# Multiple-input multiple-output causal strategies for gene selection

**DOI:** 10.1186/1471-2105-12-458

**Published:** 2011-11-25

**Authors:** Gianluca Bontempi, Benjamin Haibe-Kains, Christine Desmedt, Christos Sotiriou, John Quackenbush

**Affiliations:** 1 Machine Learning Group, Computer Science Department, Université Libre de Bruxelles, Belgium; 2 Computational Biology and Functional Genomics Laboratory, Department of Biostatistics and Computational Biology, Dana-Farber Cancer Institute, Harvard School of Public Health, USA; 3 Breast Cancer Translational Research Laboratory, Department of Medical Oncology, Institut Jules Bordet, Université Libre de Bruxelles, Belgium

## Abstract

**Background:**

Traditional strategies for selecting variables in high dimensional classification problems aim to find sets of maximally relevant variables able to explain the target variations. If these techniques may be effective in generalization accuracy they often do not reveal direct causes. The latter is essentially related to the fact that high correlation (or relevance) does not imply causation. In this study, we show how to efficiently incorporate causal information into gene selection by moving from a single-input single-output to a multiple-input multiple-output setting.

**Results:**

We show in synthetic case study that a better prioritization of causal variables can be obtained by considering a relevance score which incorporates a causal term. In addition we show, in a meta-analysis study of six publicly available breast cancer microarray datasets, that the improvement occurs also in terms of accuracy. The biological interpretation of the results confirms the potential of a causal approach to gene selection.

**Conclusions:**

Integrating causal information into gene selection algorithms is effective both in terms of prediction accuracy and biological interpretation.

## Background

Supervised analysis of genomic datasets (gene expression microarray or comparative genomic hybridization array for instance) with a large number of features and a respectively small number of samples requires the adoption of either regularization or feature selection strategies [1]. The most common feature selection strategies select or rank the variables according to a relevance score. In ranking, for instance, the score of each variable is the univariate association with the target returned by a measure of relevance, like mutual information, correlation, or p-value. If on one hand the ranking is widely used for its simple implementation and its low complexity, on the other hand it suffers from well-known limitations. A drawback is that ranking relies on univariate terms and as such it cannot take into consideration higher-order interaction terms or redundancy between features [2]. Another limitation is that ranking techniques are not able to distinguish between causes and effects. This is due to the fact that univariate correlation (or relevance) does not imply causation [3]. This problem is not solved in multivariate feature selection approaches since their cost function typically takes into consideration accuracy but disregards causal aspects. Nowadays the importance of bringing causality into play when designing feature selection methods is more widely acknowledged in the bioinformatics and the machine learning communities [4,5]. This is typically the case in microarray classification, where the goal is, for example, to distinguish between tumor classes or predict the effects of therapies on the basis of gene expression profiles [6]. In these settings the number of input variables, represented by the number of gene probes, is huge (typically several thousands) while the number of samples, represented by the patients' tumors, is very limited (a few hundreds) making the selection of relevant genes a challenging task. Moreover the inference of causal relationships between variables plays a major role in the context of genomic studies since more and more biologists and medical doctors expect data analysis to provide not only accurate prediction models (for prognostic purposes) but also insights into the mechanisms associated with disease and appropriate therapeutic targets.

It is well established that the detection of causal patterns cannot be carried out in a bivariate (single-input single-output) context and that at least a trivariate setting has to be considered [7]. This is put into evidence by the literature on graphical models where arc orientation relies on notions of conditional independence (requiring at least three terms) [8] and by the work on information theoretic methods for network inference [9]. In particular this paper will focus on the notion of *feature interaction*, a three-way mutual information that differs from zero when group of attributes are complementary [10]. The role of interaction in feature selection has already been discussed in the machine learning literature. Jakulin proposed an heuristic based on interaction for selecting attributes within the naive Bayesian classifier [11]. Meyer et al. proposed a filter algorithm which relies on the maximization of an information theoretic criterion, denoted Double Input Symmetrical Relevance (DISR), which implicitly takes into account the interaction, or complementarity between variables, in the choice of the features [12]. Watkinson et al. used a notion of synergy related to feature interaction to assign a score to a pair of genes and then measured the degree of confidence that one of the genes regulates the other [9]. A causal filter algorithm which computes interaction between inputs has been recently proposed in [5]. However it is unclear whether these techniques are capable of recovering the set of features that are both relevant and causal, in high-dimensional problems, such as in microarray analysis.

The contributions of this paper can be summarized as follows. First we introduce a new causal filter based on the interaction information and we show how to estimate this quantity in a multiple-input multiple-output setting. Second we assess the capacity of such filter to prioritize causal variables by using a synthetic case study. Third we measure from an accuracy and a biological point of view the performance of such causal filter in a number of prognostic studies in breast cancer. We advocate that a multiple-input multiple-output approach is particularly relevant in clinical studies where it is common that more than a single target variable is collected. This is the case of prognostic studies of breast cancer patients where several clinical indices, including patients' tumor size and histological grade, are collected together with the survival of the patients and the gene expressions of their tumor. It is worth to note that, in spite of their availability, these additional phenotypes are usually not taken into consideration since statistical studies focus on survival prediction and adopt single-output methods.

This paper describes an original multiple-input multiple-output score which combines a conventional relevance term with a causal term. This additional term quantifies the causal role of the features and allows the prioritization of causal variables in the resulting ranking. We carried out a synthetic study, where the set of causal dependencies is known, which shows that causal variables are highly ranked once this score is adopted. We performed a meta-analysis of six publicly available breast cancer microarray datasets to assess the improvement of using our causal relevance score in terms of accuracy over the conventional ranking. The related discussion shows also that it is possible to carry out a biological interpretation of the role of selected variables which allows to discriminate between potentially causal and relevant, yet non causal, features. The source code, documentation and data are open-source and publicly available from http://mlg.ulb.ac.be/software/ and http://compbio.dfci.harvard.edu/pubs/mimocausal/.

## Methods

### Mutual information and interaction

Let us consider a multiple-input multiple-output (MIMO) classification problem characterized by *n *input variables **X **= {**x**_*i*_, *i *= 1,..., *n*} and *m *targets **Y **= {**y**_*j*_, *j *= 1,..., *m*} where $x_{i}\in X$ is continuous and $y_{j}\in Y_{j}=\left\{c_{j1},\ldots ,c_{jC}\right\}$. Let us denote **y**_1 _as the *primary target *and the remaining *m *- 1 outputs as *secondary targets*. We make this distinction since, though we assume that the goal of classification is to predict **y**_1_, we want to take advantage of the causal information which can be extracted by multiple targets. We begin by reviewing some notions of information theory by considering three random (boldface) variables, notably two inputs **x**_1_, **x**_2 _and the primary target **y**_1_. The mutual information [13] between the continuous variables **x**_1 _and **x**_2 _is defined in terms of their probabilistic density functions *p*(*x*_1_), *p*(*x*_2_) and *p*(*x*_1_, *x*_2_) as

(1) $$I\left(x_{1};x_{2}\right)=\int \int p\left(x_{1},x_{2}\right)\log\frac{p\left(x_{1},x_{2}\right)}{p\left(x_{1}\right)p\left(x_{2}\right)}dx_{1}dx_{2}=H\left(x_{1}\right)-H\left(x_{1}|x_{2}\right)$$

where *H *is the entropy and the convention $0\log\frac{0}{0}=0$ is adopted. This quantity measures the amount of stochastic dependence between **x**_1 _and **x**_2 _and is also called two-way interaction [11]. Note that, if **x**_1 _and **x**_2 _are Gaussian distributed the following relation holds

(2) $$I\left(x_{1};x_{2}\right)=-\frac{1}{2}\log\left(1-\rho^{2}\right)$$

where *ρ *is the Pearson correlation coefficient.

Let us now consider the target **y**_1_, too. The *conditional mutual information I*(**x**_1_; **x**_2_|**y**_1_) [13] between **x**_1 _and **x**_2_, once **y**_1 _is given, is defined by

$$∭p\left(x_{1},x_{2},y_{1}\right)\log\frac{p\left(x_{1},x_{2}|y_{1}\right)}{p\left(x_{1}|y_{1}\right)p\left(x_{2}|y_{1}\right)}dx_{1}dx_{2}dy_{1}$$

The conditional mutual information is null iff **x**_1 _and **x**_2 _are conditionally independent given **y**_1_. The change of dependence between **x**_1 _and **x**_2 _due to the knowledge of **y**_1 _is measured by the three-way *interaction information *defined in [14] as

(3) $$\begin{array}{c} I\left(x_{1};x_{2};y_{1}\right)=I\left(x_{1};y_{1}\right)-I\left(x_{1};y_{1}|x_{2}\right)=\\ \;\;\;\;\;\;\;\;=-H\left(x_{1},x_{2}\right)-H\left(x_{1};y_{1}\right)-H\left(x_{2};y_{1}\right)+H\left(x_{1}\right)+H\left(x_{2}\right)+H\left(y_{1}\right)+H\left(x_{1},x_{2},y_{1}\right) \end{array}$$

This measure quantifies the amount of mutual dependence that cannot be explained by bivariate interactions. When it is different from zero, we say that **x**_1_, **x**_2 _and **y**_1 _three-interact. A non-zero interaction can be either negative, which denotes a synergy or complementarity between the variables, or positive, which indicates redundancy. Because of the symmetry of the *H *operator in (3), we have

(4) $$\begin{array}{ll} I\left(x_{1};x_{2};y_{1}\right) & =I\left(x_{1};y_{1}\right)-I\left(x_{1};y_{1}|x_{2}\right)\;\\ & =I\left(x_{2};y_{1}\right)-I\left(x_{2};y_{1}|x_{1}\right)\;\\ & =I\left(x_{1};x_{2}\right)-I\left(x_{1};x_{2}|y_{1}\right)\;\\ & \; \end{array}$$

By (4) we derive

(5) $$I\left(x_{1};y|x_{2}\right)=I\left(x_{1};y\right)-I\left(x_{1};x_{2}|y\right)$$

Since the joint information of **x**_1 _and **x**_2 _to **y**_1 _can be written as

$$I\left(\left(x_{1};x_{2}\right);y\right)=I\left(x_{2};y\right)+I\left(x_{1};y|x_{2}\right)$$

it follows that by adding *I*(**x**_2_; **y**) to both sides of (5) we obtain

(6) $$I\left(\left(x_{1};x_{2}\right);y\right)-I\left(x_{1};y\right)+I\left(x_{2};y\right)-I\left(x_{1};x_{2};y\right)=I\left(x_{1};y\right)+I\left(x_{2};y\right)+I\left(x_{1};x_{2}|y\right)-I\left(x_{1};x_{2}\right)$$

Note that the above relationships hold also when either **x**_1 _or **x**_2 _are vectorial random variables.

### Feature selection, causality and interaction

Consider a multiple-class classification problem where **x **∈ **X **⊂ ℝ^*n *^is the *n*-variate input and $y_{1}\in Y$ is the primary target variable. Let *A *= {1,..., *n*} be the set of indices of the *n *inputs. Let us formulate the feature selection problem as the problem of finding the subset X* of *v *> 0 variables such that

(7) $$X^{*}=\arg\underset{S\subset A:|S|=v}{\max}I\left(X_{S};y_{1}\right)=\arg\underset{S\subset A:|S|=v}{\max}픰\left(X_{S}\right)$$

where the score $픰\left(X_{S}\right)$ of a subset **X**_*S *_of variables is given by the mutual information it brings to the target. In other words, for a given number *v *of variables the optimal feature set is the one that maximizes the information about the target. Note that this formulation of the feature selection problem, also known as Max-Dependency [12,15], is classifier-independent.

If we want to carry out the maximization (7), both an estimation of *I *and a search strategy in the space of subsets of **X **are required. As far as the search is concerned, according to the Cover and Van Campenhout theorem [16], to be assured of finding the optimal feature set of size *v*, all feature subsets should be assessed. Given the infeasibility of exhaustive approaches for large *n*, we will consider here only forward selection search approaches. Forward selection starts with an empty set of variables and incrementally updates the solution by adding the variable that is expected to bring the best improvement (according to a given criterion). The hill-climbing search selects a subset of *v *<*n *variables in *v *steps by exploring only $\sum_{i=0}^{v}\left(n-1\right)$ configurations. For this reason the forward approach is commonly adopted in filter approaches for classification problems with high dimensionality [17,18].

If *v *= 1 the optimal set returned by (7) is composed of the most relevant variable, that is the one carrying the highest mutual information to **y**. For *v *> 1, we need to provide an incremental solution to (7) in order to obtain, given a set of *d *variables, the (*d *+ 1)^th ^feature which maximizes the increase of the dependency

(8) $$x_{d+1}^{*}=\arg\underset{x_{k}\in X-X_{S}}{\max}픰\left(\left(X_{S},x_{k}\right)\right)$$

where (**X**_*S*_, **x**_*k*_) stands for the set of variables resulting from the union of **X**_*S *_and **x**_*k*_. Since for large *d *the term $픰\left(\left(X_{S},x_{k}\right)\right)$ requires the computation of multivariate mutual information, its estimation is often prone to ill-conditioning and large variance. This led to the adoption of low variate approximations in literature, like the univariate approximation

(9) $$x_{d+1}^{*}=\arg\underset{x_{k}\in X-X_{S}}{\max}픰\left(x_{k}\right)=\arg\underset{x_{k}\in X-X_{S}}{\max}I\left(x_{k},y_{1}\right)$$

which leads to a ranking of the variables according to their mutual information with the target. More advanced approaches rely on bivariate decompositions [12] like

(10) $$x_{d+1}^{*}=\arg\underset{x_{k}\in X-X_{S}}{\max}\frac{1}{d}\underset{x_{i}\in X_{S}}{\sum }픰\left(\left(x_{i},x_{k}\right)\right)$$

where $픰\left(\left(x_{i},x_{k}\right)\right)$ quantifies the amount of information that **x**_*i *_and **x**_*k *_contain jointly about **y**_1_.

**Figure 1:**
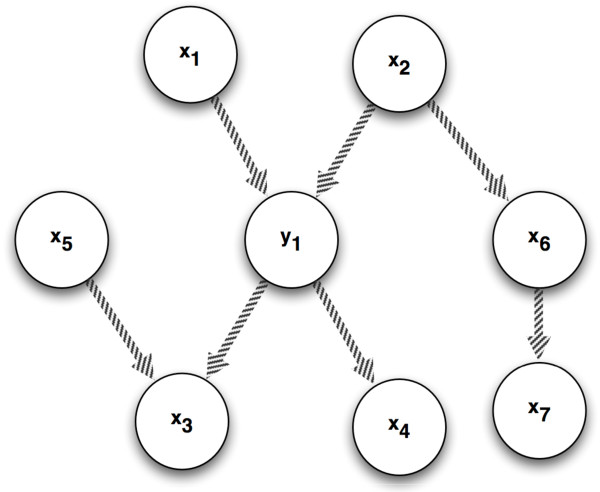
**Single-output case with different causal patterns: (i) *common effect *or *explaining away effect *configuration involving **x**_1_, **x**_2 _and **y**_1_; (ii) *spouse *configuration involving **x**_5 _and **y**_1_; (iii) *common cause *configuration involving **y**_1_, **x**_3_, **x**_4_; and (iv) *causal chain *configuration involving **x**_1_, **y**_1_, **x**_4_**.

However a feature selection procedure targeting the Max-Dependency is not able in general to discriminate between causal and non causal dependencies. For instance in a selection procedure applied to a dataset derived from a causal process like the one in Figure 1, the effect **x**_4 _could be more highly ranked than the direct causes **x**_1 _and **x**_2_.

Here we propose to modify the conventional score 픰(X) into a causal score ${픰}_{c}\left(X\right)$ able to keep into consideration the causal information returned by the adoption of a multiple output configuration. This is made possible by integrating in the score an interaction term which is strictly related to the notion of causal dependency.

#### Interaction and causal dependency

This section aims to establish the link between information theory and causality. Causality is at the same time an essential and imprecise notion in scientific discovery. In order to avoid any ambiguity, here we adopt the formalism of causal Bayesian network which is a sound and convenient framework for reasoning about causality between random variables [8]. This means that all causal dependencies between variables are expressed by a directed acyclic graph where the existence of an oriented edge from a node **x**_*i *_to a node **x**_*j *_means that **x**_*i *_directly causes **x**_*j*_. In formal terms we assume that the Causal Markov condition, the Causal Faithfulness and the Causal Sufficiency conditions hold [4]. Several works in literature showed that the structure of a causal graph can, to some extent, be inferred from observational data. The vast majority of these works rely on statistical tests of conditional independence [19]. Here we present a way to reason about causality which do not use independence tests but estimate an information theory score to prioritize potential causes.

Let us consider a triplet made of two inputs **x**_*i*_, **x**_*j *_and one target **y**_1_. As discussed in [4] six possible configurations of directed acyclic graphs involving three variables can occur. One configuration is trivial and corresponds to a completely unconnected graph. One configuration corresponds to a single arrow chain (for example only **x**_*i *_and **x**_*j *_are linked) and it is well known in literature that for a system of two variables the causal structure is not distinguishable. Another configuration corresponds to a fully connected graph and in this case the lack of independencies implies that the direction of the arrows cannot be determined. The remaining configurations can be illustrated and detected by studying the relationship [5] between the sign of *I*(**x**_*i*_; **x**_*j*_; **y**_1_) and causal patterns of the triplet, like the ones sketched in Figure 1.

A negative interaction *I*(**x**_*i*_; **x**_*j*_; **y**_1_) means that the knowledge of the value **y**_1 _increases the amount of dependence between **x**_*i *_and **x**_*j*_; this situation occurs in the presence of a collider. According to the label of the collider we can have two cases: i) the *common effect *configuration (for example the pattern involving **x**_1_, **x**_2 _and **y**_1_, also known as the *explaining-away *effect) and ii) the *spouse *configuration (the pattern involving **x**_3_, **x**_5 _and **y**_1 _in Figure 1 where **x**_3 _is the common descendant of **y**_1 _and **x**_5_). This is a consequence of the fact that, if we assume Causal Faithfulness, the graph structure entails that the two parents are independent (null mutual information) but conditionally dependent (conditional mutual information bigger than zero). Note also that both configurations are characterized by the presence of a collider.

On the contrary a positive interaction *I*(**x**_*i*_; **x**_*j*_; **y**_1_) between **x**_*i *_and **x**_*j *_means that the knowledge of **y**_1 _decreases the amount of dependence. This situation occur in two cases: i) the *common cause *configuration (for example, two dependent effects **x**_3 _and **x**_4 _become independent once the value of the common cause **y**_1 _is known as illustrated in Figure 1) and ii) the *causal chain *configuration where one of the variables (let say, **x**_1_) is the cause and the other (let say, **x**_4_) is the effect of **y**_1_. This is due to the fact that the graph entails the dependence between **x**_*i *_and **x**_*j *_as well as their conditional independence (null conditional mutual information).

**Figure 2:**
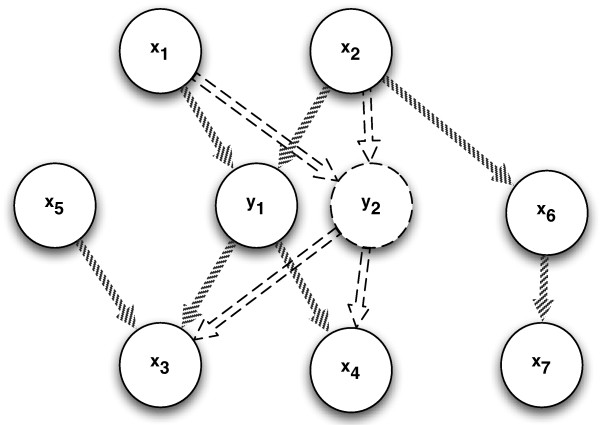
**Two-output case with different causal patterns: (i) *common effect *configuration involving **x**_3_, **y**_1 _and **y**_2_; (ii) *spouse *configuration involving **y**_2 _and **x**_6_; (iii) *common cause *configuration involving **x**_1_, **y**_1 _and **y**_2_; and (iv) *causal chain *configuration involving **x**_1_, **y**_2 _and **x**_7_**.

So far we have considered a single-output configuration. However causal patterns can be better identified if we consider a multiple-output configuration, for instance the two output configuration sketched in Figure 2. If **y**_1 _and **y**_2 _are two outputs representing different observations of the same phenomenon (for example a disease) we expect that the causal configurations concerning the first output appear also for the second one. This is a reasonable assumption in breast cancer clinical studies where the measured phenotypes (size and histological grade of the tumor for instance) can be considered as different manifestations of the state of the tumor.

Let us consider for instance the inputs **x**_1 _and **x**_2 _and the two targets **y**_1 _and **y**_2_: the *common effect *configurations between **x**_1 _and **x**_2 _and **y**_1 _holds also for the triplet **x**_1 _and **x**_2 _and **y**_2_. The same happens for the common cause pattern involving both the triplet **x**_3_, **x**_4_, **y**_1 _and **x**_3_, **x**_4_, **y**_2_. The presence of multiple outputs can therefore make more robust the identification of a causal pattern, especially in data configurations characterized by a very large number of variables.

In the following we will take advantage of these considerations to design a causal filter able to extract from observed data causal dependencies between variables.

#### The MIMO causal filter

**Figure 3:**
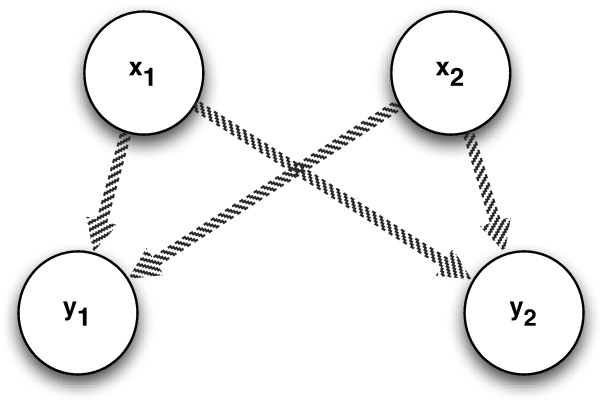
**Two-inputs two-outputs causal pattern**.

The link between causality and interaction discussed in the previous section suggests that, if we want to detect causality without estimating large variate dependencies, we may search for patterns like the one sketched in Figure 3. This dependency pattern is characterized by two causal inputs and two outputs and can be detected when the following two conditions are satisfied:

1 the interaction *I*(**x**_1_; **x**_2_; **y**_1_) is negative

2 the interaction *I*(**x**_1_; **x**_2_; **y**_2_) is negative

In what follows we implement this idea into a MIMO causal filter where input variables belonging to causal patterns like the one in Figure 3 are prioritized.

For the pair of inputs **x**_1 _and **x**_2 _and the pair of outputs **y**_1 _and **y**_2_, we define a structural score

(11) $$C\left(x_{1},x_{2}\right)=-\frac{1}{2}\left(I\left(x_{1};x_{2};y_{1}\right)+I\left(x_{1};x_{2};y_{2}\right)\right)$$

which is composed of two multiple-input interaction terms. The magnitude of this score depends on whether **x**_1 _and **x**_2 _jointly play a joint causal role on **y**_1 _and **y**_2_, or in other words, the pattern in Figure 3 is encountered. This means that the higher the term *C*(**x**_1_, **x**_2_), the higher is the evidence that the pair **x**_1_, **x**_2 _be a cause of **y**_1 _ad **y**_2_. This score plays a similar role to the score that is maximized in structural identification of Bayesian networks [20]. If in that case the score measures the likelihood of the data for a given graph structure, here the quantity *C*(**x**_1_, **x**_2_) measures the likelihood of the data for a structural pattern where the pair **x**_1_, **x**_2 _has a causal role.

In the case of bivariate output (*m *= 2) we propose then a causal version ${픰}_{c}$ of the univariate score  which accounts both for the relevance and the causal role of a pair of input variables **x**_1 _and **x**_2_

(12) $${픰}_{c}\left(\left(x_{1},x_{2}\right)\right)=I\left(x_{1};y_{1}\right)+I\left(x_{2};y_{1}\right)+\lambda C\left(x_{1},x_{2}\right)$$

where *λ *> 0 stands for the degree of *causality *imposed to the selection. If we adopt the filter approximation (10) the incremental formula takes the form

(13) $$\begin{array}{c} x_{d+1}^{*}=\arg\underset{x_{k}\in X-X_{S}}{\max}\frac{1}{d}\underset{x_{i}\in X_{S}}{\sum }{픰}_{c}\left(\left(x_{i};x_{k}\right)\right)=\\ \;\;\;\;\;\;\;\;\;=\arg\underset{x_{k}\in X-X_{S}}{\max}\left[I\left(x_{k};y_{1}\right)+\frac{\lambda }{d}\underset{x_{i}\in X_{S}}{\sum }C\left(x_{i};x_{k}\right)\right]=\\ \;\;\;\;\;\;\;\;\;\;\;=\arg\underset{x_{k}\in X-X_{S}}{\max}\left[I\left(x_{k};y_{1}\right)-\frac{\lambda }{2d}\underset{x_{i}\in X_{S}}{\sum }\left(I\left(x_{i};x_{k};y_{1}\right)+I\left(x_{i};x_{k};y_{2}\right)\right)\right] \end{array}$$

In other terms this formulation suggests to add at the (*d *+ 1)^th ^step, among all the remaining variables, the one which has the better combination of relevance and causality, where the causal term is obtained by averaging over the selected variables and the considered outputs. Note that in the case of *m *> 2 targets the structural score (11) is obtained by averaging the interaction terms over the *m *variables.

Similarly to what is done in regularization approaches [21] where specific configurations (typically those with higher complexity) are penalized by adding a complexity term to the one measuring the error, the causality parameter *λ *in (13) is expected to penalize input variables with no causal role (positive interaction). Note that for *λ *= 0 the selection rule (13) boils down to the rule (9). The following section will study the impact of the causality term on the accuracy and the stability of a filter algorithm implementing the rule (13).

## Results

In this section we perform two experiments to assess the role of the causation term in the feature selection process. The first one is based on a number of synthetic datasets generated by simulating a causal Bayesian network while the second relies on public microarray breast cancer datasets to assess the approach in a real data setting.

### Synthetic data

**Figure 4:**
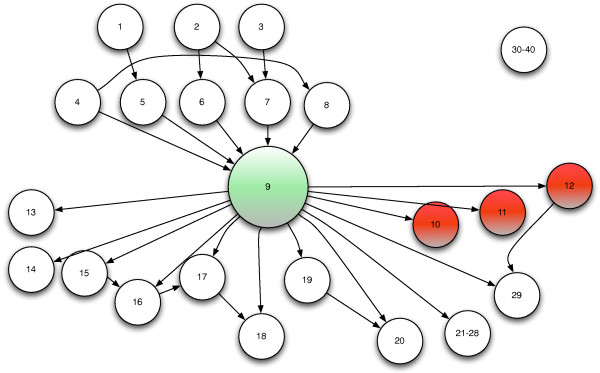
**Bayesian causal network used for synthetic experiment**. The green node 9 denotes the non observable variable. The three red nodes denote the targets of the multiple-output classification problem. The isolated node (30-40) represents a set of 11 independent variables.

This experiment focuses on the prioritization of causes in a set of classification tasks defined on the basis of simulated data generated by the causal structure depicted in Figure 4. Note that this causal structure aims to represent in a very simplified manner a stochastic dependency characterized by a number of indirect (nodes 1-3) and direct causes (nodes 4-8), a latent non measurable variable (node 9), one observable primary target (node 10), two secondary targets (nodes 11-12), a set of additional effects (nodes 13-29) and a number of independent and irrelevant variables (nodes 30-40). In order to set up an analogy with the real data experiments of the following subsection, we could make the assumption that the latent variable represents the cancer progression, the three targets denote a set of observable measures depending on the cancer state (patients' prognosis, size and histological grade of the tumor for instance), and that all other variables represent the expression of genes whose activity could play a causal role, be determined as an effect of the disease or be completely irrelevant. It is worth to note that also in the presence of a hidden variable the interaction between marginally independent causes given an effect is negative. This is due to the fact that conditioning on the hidden variable or on one of his children is equivalent in terms of d-separation between the variables [8] and consequently is equivalent in terms of the sign of the interaction. We simulate a number of multivariate datasets from the causal structure in Figure 4 and for each of them we rank the inputs of the MIMO classification problem by using the conventional ranking approach based on mutual information (Equation (9)) and our novel approach based on causality (Equation (13)). The stochastic dependency between parents and descendants of the network is modeled by a linear regression where the parameters are uniformly sampled in [-2, 2] and the noise distribution is Gaussian with zero mean and standard deviation *σ*. We carry out a series of experiments, each characterized by 150 datasets and an increasing noise standard deviation ranging between 0.01 and 0.4. All the variables are continuous apart from the variables 10, 11, and 12, which correspond to the targets **y**_1_, **y**_2_, and **y**_3 _of the classification task and are discretized to two binary values. Note that all measures are centered and scaled in order to have a zero mean and unit standard deviation; this allows for a better understanding of the impact of the noise amplitude on the ranking.

**Figure 5:**
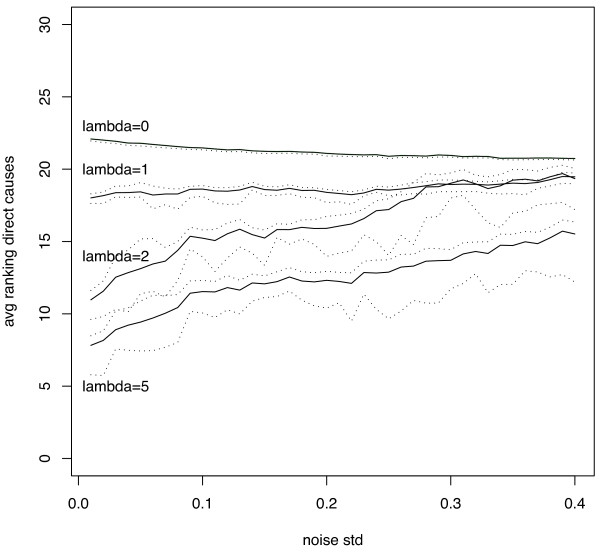
**Synthetic data experiment: average ranking of direct causes for different values of λ as a function of the noise standard deviation**. Dotted lines are used to denote the 90% confidence interval estimated on the basis of 150 trials.

**Figure 6:**
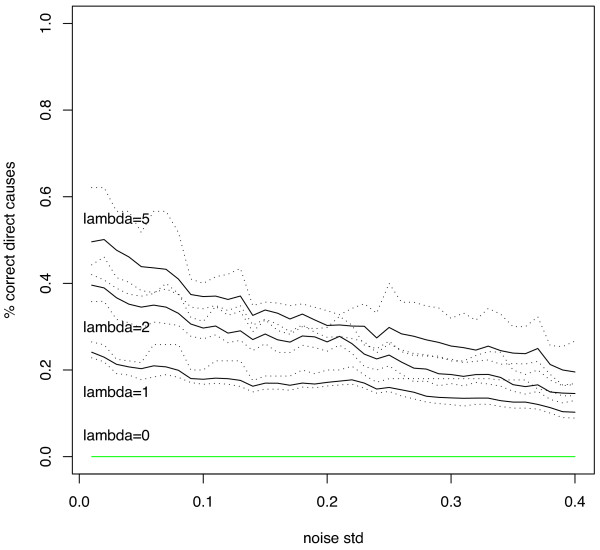
**Synthetic data experiment: probability of selecting a direct cause among the first 5 ranked variables for different values of λ as a function of the noise standard deviation**. Dotted lines are used to denote the 90% confidence interval estimated on the basis of 150 trials.

The quality of our causal prioritization strategy is assessed by measuring the average ranking of the direct causes (nodes 4-8) and the percentage of time that the direct causes are ranked among the first 5 variables. These two measures (together with a 90% confidence interval) for different values of λ are shown in Figure 5 and 6 respectively. These plots show that by increasing the value of λ, the average ranking position of direct causes decreases (direct causes are better prioritized) and that the percentage of correct selection increases (among the first ranked variables we find the direct causes with higher probability). The improvement occurs in a consistent manner for different values of the noise standard deviation though the detection of causal terms become less accurate as the noise increases. Note also that the very bad performance of the ranking (λ = 0) strategy (0% rate of correct selection) derives from the very large number of effects which tend to be ranked before the real causes.

### Real expression data

The real data experiment consists of 6 public microarray datasets derived from breast cancer clinical studies (Table 1) in order to compare the generalization accuracy of the selection returned by the conventional ranking approach based on mutual information (Equation (9)) with the accuracy of the selection returned by our novel approach based on causality (Equation (13)).

**Table 1:** Affymetrix microarray datasets and related clinical study where the gene expression have been originally published

Dataset	Patients	Reference
UPP	251 (110)	[52]

STK	159	[53]

VDX	344	[54,55]

UNT	137 (92)	[56]

MAINZ	200	[57]

TRANSBIG	198	[58]

Duplicated patients between studies have been removed in two studies, UPP and UNT; the remaining unique patients are reported in brackets. All the datasets have been generated from Affymetrix technology and normalized using fRMA [51]. We consider for analysis the 13,091 unique genes common in all datasets.

All the microarray studies analyzed hereafter are characterized by the collection of gene expression data (the inputs **X **representing *n *= 13,091 unique genes), the survival data (the primary target **y**_1_) and 2 additional clinical (secondary) variables about the state of the tumor, namely the histological grade and the tumor size. These clinical variables are well known by clinicians to be highly relevant for prognosis since large tumors of high grade are usually aggressive and lead to poor prognosis. Each experiment was conducted in a meta-analytical [22] and cross-validation [23] framework, that is the set of variables are selected by relying on the samples of several datasets and the validation is performed on a set of samples not used for the selection. In order to adopt a classification framework, the survival of the patients was transformed in a binary class such as low or high risk of the patients given their clinical outcome at five years as in [24]. We conducted two sets of meta-analysis validation experiments to compare the conventional ranking approach (λ = 0 case) and our causal version for different values of λ:

• Holdout: we carried out 100 training-and-test repetitions where for each repetition the training set is composed of half of the samples of each dataset and the test is composed of the remaining ones.

• Leave-one-dataset-out where for each dataset the features used for classification are selected without considering the patients of the dataset itself. Once the selection is over, 100 holdout repetitions are used to assess the generalization power of the selected set of features.

All the mutual information terms are computed by using the Gaussian approximation (2). This allows the meta-analysis integration at the correlation level by means of the weighted estimation approach proposed by [22]. All the experiments were repeated for three sizes of the gene signature (number of selected features): *v *= 20, 50, 100.

The quality of the selection is represented by the accuracy of a Naive Bayes classifier measured by four different criteria: the Area Under the ROC curve (AUC), the Root Mean Squared Error (RMSE), the SAR (Squared error, Accuracy, and ROC score introduced by [25]) and the precision-recall *F *score measure [26]. Table 2 reports for the holdout experiment the value of the four performance criteria for different values of *v *and λ. Table 3 refers to the leave-one-dataset-out experiments for *v *= 20, *v *= 50, and *v *= 100, respectively. Note that the W-L (Win-Loss) line reports the number of datasets for which the causal filter is significantly more (W) or less (L) accurate than the ranking filter according both to the McNemar test [27] (p-value < 0.05 adjusted for multiple testing by Holm's method [28]) and the Wilcoxon paired test on squared errors (p-value < 0.05 adjusted for multiple testing by Holm's method).

**Table 2:** Holdout: accuracy criteria (to be maximized) for different numbers *v *of variables and different values of λ

*v *= 20	λ = 0	λ = 0.2	λ = 0.4	λ = 0.6	λ = 0.8	λ = 0.9	λ = 1	λ = 2
AUC	0.688	0.688	0.694	0.699	0.703	0.704	0.705	0.707
1-RMSE	0.460	0.466	0.481	0.493	0.504	0.510	0.515	0.542
SAR	0.559	0.561	0.569	0.575	0.580	0.583	0.585	0.595
F	0.255	0.254	0.260	0.262	0.265	0.265	0.266	0.274

W-L		1-0	3-0	5-0	6-0	5-0	5-0	5-0


*v *= 50	λ = 0	λ = 0.2	λ = 0.4	λ = 0.6	λ = 0.8	λ = 0.9	λ = 1	λ = 2

AUC	0.693	0.698	0.702	0.706	0.709	0.710	0.711	0.715
1-RMSE	0.451	0.458	0.465	0.471	0.477	0.479	0.482	0.503
SAR	0.552	0.556	0.562	0.567	0.571	0.572	0.574	0.583
F	0.263	0.265	0.268	0.270	0.272	0.271	0.273	0.277

W-L		2-0	3-0	3-0	2-0	2-0	3-0	4-0


*v *= 100	λ = 0	λ = 0.2	λ = 0.4	λ = 0.6	λ = 0.8	λ = 0.9	λ = 1	λ = 2

AUC	0.699	0.704	0.708	0.711	0.714	0.715	0.715	0.716
1-RMSE	0.454	0.457	0.459	0.463	0.467	0.470	0.472	0.487
SAR	0.545	0.549	0.553	0.557	0.561	0.563	0.564	0.573
F	0.272	0.271	0.272	0.274	0.274	0.274	0.275	0.284

W-L		1-0	1-0	1-0	2-0	3-0	4-1	4-1

AUC = Area Under the Curve; 1-RMSE = one minus Root Mean Squared Error; SAR = Squared error, Accuracy, and ROC; F = precision-recall; W-L = Win -Loss reporting the number of datasets for which the causal filter is significantly more (W) or less (L) accurate than the conventional ranking filter according both to the McNemar test (p-value < 0.05 adjusted for multiple testing by Holm's method) and the Wilcoxon paired test on squared errors (p-value < 0.05 adjusted for multiple testing by Holm's method).

**Table 3:** Leave-one-dataset-out: accuracy criteria (to be maximized) for different numbers *v *of variables and different values of λ

*v *= 20	λ = 0	λ = 0.2	λ = 0.4	λ = 0.6	λ = 0.8	λ = 0.9	λ = 1	λ = 2
AUC	0.678	0.674	0.678	0.680	0.682	0.682	0.680	0.669
1-RMSE	0.447	0.448	0.467	0.469	0.482	0.528	0.544	0.556
SAR	0.553	0.552	0.560	0.561	0.566	0.582	0.586	0.586
F	0.280	0.275	0.275	0.281	0.279	0.283	0.287	0.276

W-L		1-1	5-1	2-0	4-0	5-0	4-0	4-0


*v *= 50	λ = 0.	λ = 0.2	λ = 0.4	λ = 0.6	λ = 0.8	λ = 0.9	λ = 1	λ = 2

AUC	0.681	0.687	0.692	0.693	0.698	0.700	0.700	0.693
1-RMSE	0.428	0.438	0.453	0.457	0.464	0.473	0.490	0.516
SAR	0.542	0.551	0.559	0.561	0.565	0.569	0.576	0.582
F	0.284	0.284	0.281	0.281	0.285	0.291	0.298	0.303

W-L		3-0	4-0	5-1	3-0	5-0	4-0	6-0


*v *= 100	λ = 0	λ = 0.2	λ = 0.4	λ = 0.6	λ = 0.8	λ = 0.9	λ = 1	λ = 2

AUC	0.687	0.694	0.704	0.708	0.711	0.706	0.708	0.676
1-RMSE	0.430	0.436	0.449	0.457	0.463	0.463	0.476	0.477
SAR	0.537	0.545	0.556	0.562	0.566	0.565	0.571	0.561
F	0.290	0.292	0.294	0.296	0.299	0.294	0.304	0.288

W-L		1-0	4-0	6-0	4-0	4-0	5-0	5-1

AUC = Area Under the Curve; 1-RMSE = one minus Root Mean Squared Error; SAR = Squared error, Accuracy, and ROC; F = precision-recall; W-L = Win -Loss reporting the number of datasets for which the causal filter is significantly more (W) or less (L) accurate than the conventional ranking filter according both to the McNemar test (p-value < 0.05 adjusted for multiple testing by Holm's method) and the Wilcoxon paired test on squared errors (p-value < 0.05 adjusted for multiple testing by Holm's method).

## Discussion

In the previous section we reported the accuracy results of the traditional ranking approach and our novel method based on a causal relevance score. Here we discuss the added value of our causal approach both from a quantitative and qualitative perspective.

The performance measured in cross-validation suggests that the incorporation of a causal term leads to a significant improvement of classification accuracy. This improvement is observed for different validation configurations and different sizes of the prognostic gene signature. From these results we can conclude that (i) causal feature selection is interesting also for a prediction perspective and (ii) relevant (prognostic) information is contained into secondary output variables (in our case tumor size and histological grade). Although the absolute improvement is only moderate (3% to 6% depending on the validation configurations and performance estimates), the use of our causal ranking strategy in more sophisticated modeling approach for prognosis, such as in [29], may help develop more clinically relevant prognostic classifiers in breast cancer.

**Figure 7:**
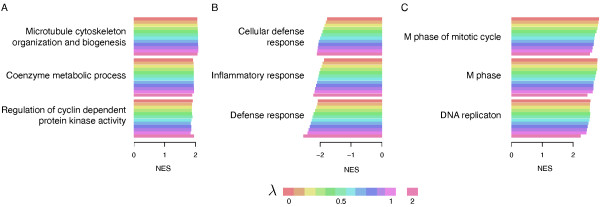
**Most enriched GO terms with respect to λ according to a pre-ranked gene set enrichment analysis (GSEA): (A) GO terms enriched in the conventional ranking and having a high degree of causality for tumorigenesis; (B) GO terms increasingly enriched with respect to larger λ, suggesting they are putative causes for tumorigenesis; (C) GO terms decreasingly enriched with respect to larger λ, suggesting they are putative effects for tumorigenesis**. The normalized enrichment score (NES) depends on the genome-ranking of the genes, which in turn depends on λ. Larger the NES of a GO term, stronger the association of this gene set with survival; the sign of NES reflects the direction of association of the GO term with survival, a positive score meaning that over-expression of the genes implies worst survival and inversely.

The other advantage of our approach is that the introduction of a causality term leads to an interpretation of the causal role of the selected genes. We illustrate this characteristic in Figure 7 by comparing, through Gene Ontological (GO) terms, gene rankings with increasing degree of causality using a pre-ranked gene set enrichment analysis (GSEA) [30]. By quantifying how the causal rank of genes diverges from the conventional one (λ = 0) with respect to λ we can identify the gene sets that are potential causes or effects of breast cancer.

Genes that remains among the top ranked ones for increasing λ can be considered as relevant (they contain predictive information about survival) and causal. Genes whose rank increases for increasing λ are putative causes: they have less relevance than other genes (for example, those being direct effects) but they are potentially causal. These genes would have been missed by conventional ranking, where they would appear as false negatives if we interpret the outcome of conventional ranking in causal terms. Genes whose rank decreases for increasing λ are putative effects in the sense that they are relevant but probably not causal. This set of genes could be erroneously considered as causal, and represent false positives if we interpret the outcome of conventional ranking in causal terms.

Since genes are not acting in isolation but rather in pathways, we analyzed the gene rankings in terms of gene set enrichment. As described in [30], the normalized enrichment score (NES) computed in GSEA enables quantification of the strength of association of a gene set (GO term) with a phenotye of interest, here poor or good prognosis (survival). In more details, given a list of genes *L *ranked by their prognostic relevance and an *a priori *defined set of genes *S *(for example genes sharing the same GO category), the goal of GSEA is to determine whether the members of *S *are randomly distributed throughout *L *or primarily found at the top or bottom; gene sets associated with the prognosis phenotype tend to show the latter distribution. NES reflects the degree to which a gene set *S *is overrepresented at the extremes (top or bottom) of the entire ranked list *L*. The score is calculated by walking down the list *L*, increasing a running-sum statistic when a gene in *S *is encountered and decreasing it when genes not in *S *are encountered. The magnitude of the increment depends on the statistic used to rank the genes in *L*. In our study the statistic of a gene is simply its rank (the most relevant genes have the largest ranks) and its sign depends on the association of its expression with survival: positive sign if over-expression is associated with poor survival and inversely. The score is the maximum deviation from zero encountered in the "walk"; it corresponds to a weighted Kolmogorov-Smirnov-like statistic [30,31]. Finally the score is normalized for each gene set to account for the size of the gene set, yielding a NES.

We computed NES for multiple genome-wide rankings generated with increasing values of λ, and displayed in Figure 7 the score of the 3 most enriched GO terms which are identified as being potentially (A) both causes and effects, (B) causes, and (C) effects of breast tumorigenesis (GSEA results for all the GO terms are provided in Additional File 1, 2 and 3). The first group of GO terms that show similar enrichment scores independently of their level of causality are implicated in cell movement and division, cellular respiration and regulation of cell cycle (Figure 7A). The first GO term involves genes encoding for the Rho family of GTPases proteins that are among key regulators of actin and microtubule cytoskeleton [32] and are often over-expressed in human breast cancers [33]. Bromberg et al. showed that, when affected by RNF5, this family of proteins may cause dysregulation of cell proliferation to promote tumor progression [34]. The second GO term represents the co-enzyme metabolic process which includes proteins showed to be early indicators of breast cancer [35]; perturbation of these co-enzymes might cause cancers by compromising the structure of important enzyme complexes implicated in mitochondrial functions [35]. Genes involved in the third GO term "regulation cyclin-dependent protein kinase activity" are key players in cell cycle regulation and inhibition of such kinases proved to block proliferation of human breast cancer cells [36]. Moreover, Moore et al. recently highlighted the role of cyclin-dependent kinases as progesterone activators that could give raise to tumors and sustain their progression in breast cancer [37].

Figure 7B displays the GO terms that are increasingly enriched with their degree of causality, involving genes that are putative causes of the tumorigenesis affecting patients' survival; these genes might have been missed by the conventional ranking approach (λ = 0). Counterintuitively, the three GO terms in this category are related to the immune system what is sought to be more an effect of the tumor growth as lymphocytes strike cancer cells as they proliferate. However, several independent research groups showed that frequent usage of aspirin significantly decrease the long-term risk of cancer death by correcting immune system dysfunction [38,39], findings that have been confirmed in breast cancer [40], what supports that the immune system might have a causal role in tumorigenesis. There is strong evidence of interplay between immune system and tumors since solid tumors are commonly infiltrated by immune cells; in contrast to infiltration of cells responsible for chronic inflammation, the presence of high numbers of lymphocytes, especially T cells, has been reported to be an indicator of good prognosis in many cancers [41], what concours with the sign of the enrichment (negative enrichment; Figure 7B). We and others have reported that gene expression signatures representing the immune response process were associated with a better prognosis in particular subtypes of breast cancer [29,42,43].

The last group of GO terms are less enriched when the degree of causality increases and the vast majority of the corresponding genes are related to cell-cycle and proliferation (Figure 7C). Cell-cycle and proliferation-related genes, such as for example Ki67, have been used for many decades to describe breast tumors: High levels of Ki67 have been correlated with worse prognosis and are also known to be associated with high tumor grade and negativity of estrogen receptor status [44,45]. We and others have shown that a quantitative measurement of proliferation genes using mRNA gene expression could provide an accurate assessment of prognosis of breast cancer patients [43,46,47]. We also have shown that only one of those genes, AURKA, which is significantly enriched in this case in the M phase GO term, was sufficient to recapitulate the prognostic performance of different prognostic signatures [48]. However the enrichment of these proliferation-related genes seems to be a downstream effect of the breast tumorigenesis instead of its cause.

Our approach allows to identify biological processes that may be direct causes of cancer. These processes are likely to be missed by conventional methods. Given the promising performance of our approach, we plan to integrate our method in analytical frameworks combining efficiently the available clinical data and *a priori *biological knowledge, potentially retrieved from biomedical literature [49] or pathway database [50], in order to unravel gene sets or network of genes causal of cancer patients' survival.

## Conclusions

It is well known in statistics that correlation does not imply causation or, in more general terms, that features that are relevant or strongly relevant for predicting a target are not necessarily direct causes. Direct effects are typical examples of variables that provide information about a target without having any causal role. In a data-driven approach to gene selection it is therefore more and more important to discriminate not only between relevant and non-relevant variables but also, within the subset of relevant variables, to discriminate between direct or indirect causes and effects. This paper proposes a computationally affordable strategy to infer causal patterns that take advantage of multiple outputs. Experimental results in terms of accuracy and clinical interpretation show the added value deriving from the inclusion of a causal term into conventional ranking.

## Abbreviations

AUC: Area Under the ROC Curve; DISR: Double Input Symmetrical Relevance; GO: Gene Ontology; GSEA: Gene Set Enrichment Analysis; MIMO: multiple-input multiple-output; NES: Normalized Enrichment Score; RMSE: Root Mean Squared Error; ROC: Receiver Operating Characteristics; SAR: Squared error, Accuracy, and ROC score; W-L: Win-Loss.

## Authors' contributions

GB and BHK were responsible for the design and execution of the study, data analysis and interpretation. CD and CS participated to the data analysis and interpretation. GB and BHK were responsible for writing the manuscript; JQ supervised the study. All authors read and approved the final manuscript.

## Supplementary Material

Additional file 1 **Spreadsheet containing the normalized enrichment scores with respect to increasing λ as computed by preranked GSEA (****gsea_res_all.csv****)**. Click here for file

Additional file 2 **Archive containing the output files computed by the preranked GSEA for λ ∈ {0.1,0.2,0.3,0.4,0.5} (****GSEA_MIMO_part1.zip****)**. Click here for file

Additional file 3 **Archive containing the output files computed by the preranked GSEA for λ ∈ {0.6,0.7,0.8,0.9,1.0,2.0} (****GSEA_MIMO_part2.zip****)**. Click here for file
