# Supplementary material for: Multiple-input multiple-output causal strategies for gene selection
Source: BMC Bioinformatics. 2011 Nov 25;12:458. doi: 10.1186/1471-2105-12-458 (PMC3323860; doi:10.1186/1471-2105-12-458)
Supplement: Additional file 2 — Archive containing the output files computed by the preranked GSEA for λ ∈ {0.1,0.2,0.3,0.4,0.5} (GSEA_MIMO_part1.zip). [file 1471-2105-12-458-S2.ZIP › mFS02_entrez_mimo.GseaPreranked.1316038034143/gsea_report_for_na_neg_1316038034143.html]

Report for na\_neg 1316038034143 [GSEA]

| GS  follow link to MSigDB | GS DETAILS | SIZE | ES | NES | NOM p-val | FDR q-val | FWER p-val | RANK AT MAX | LEADING EDGE || 1 | IMMUNE\_RESPONSE |  | 212 | -0.39 | -2.34 | 0.000 | 0.005 | 0.004 | 4483 | tags=59%, list=34%, signal=88% |
| 2 | IMMUNE\_SYSTEM\_PROCESS |  | 298 | -0.36 | -2.25 | 0.000 | 0.007 | 0.010 | 3099 | tags=42%, list=24%, signal=53% |
| 3 | DEFENSE\_RESPONSE |  | 238 | -0.35 | -2.12 | 0.000 | 0.017 | 0.038 | 4084 | tags=47%, list=31%, signal=68% |
| 4 | REGULATION\_OF\_IMMUNE\_RESPONSE |  | 28 | -0.51 | -2.08 | 0.000 | 0.021 | 0.062 | 4546 | tags=75%, list=35%, signal=115% |
| 5 | POSITIVE\_REGULATION\_OF\_IMMUNE\_RESPONSE |  | 24 | -0.55 | -2.07 | 0.000 | 0.018 | 0.068 | 4389 | tags=75%, list=34%, signal=113% |
| 6 | POSITIVE\_REGULATION\_OF\_IMMUNE\_SYSTEM\_PROCESS |  | 44 | -0.45 | -2.00 | 0.000 | 0.032 | 0.136 | 4546 | tags=66%, list=35%, signal=101% |
| 7 | INFLAMMATORY\_RESPONSE |  | 115 | -0.36 | -1.95 | 0.000 | 0.040 | 0.190 | 3033 | tags=41%, list=23%, signal=53% |
| 8 | POSITIVE\_REGULATION\_OF\_MULTICELLULAR\_ORGANISMAL\_PROCESS |  | 56 | -0.42 | -1.95 | 0.000 | 0.036 | 0.198 | 4214 | tags=59%, list=32%, signal=87% |
| 9 | RESPONSE\_TO\_WOUNDING |  | 171 | -0.34 | -1.92 | 0.000 | 0.045 | 0.270 | 3240 | tags=40%, list=25%, signal=52% |
| 10 | REGULATION\_OF\_IMMUNE\_SYSTEM\_PROCESS |  | 57 | -0.40 | -1.90 | 0.000 | 0.049 | 0.319 | 4058 | tags=58%, list=31%, signal=84% |
| 11 | CELLULAR\_DEFENSE\_RESPONSE |  | 54 | -0.40 | -1.85 | 0.000 | 0.068 | 0.438 | 3796 | tags=48%, list=29%, signal=68% |
| 12 | ADAPTIVE\_IMMUNE\_RESPONSE\_GO\_0002460 |  | 22 | -0.49 | -1.79 | 0.014 | 0.104 | 0.624 | 4058 | tags=59%, list=31%, signal=85% |
| 13 | ADAPTIVE\_IMMUNE\_RESPONSE |  | 23 | -0.46 | -1.75 | 0.009 | 0.129 | 0.728 | 4058 | tags=57%, list=31%, signal=82% |
| 14 | REGULATION\_OF\_CELL\_DIFFERENTIATION |  | 48 | -0.38 | -1.71 | 0.002 | 0.166 | 0.833 | 4546 | tags=56%, list=35%, signal=86% |
| 15 | HEMOPOIETIC\_OR\_LYMPHOID\_ORGAN\_DEVELOPMENT |  | 71 | -0.34 | -1.70 | 0.000 | 0.170 | 0.855 | 2973 | tags=39%, list=23%, signal=51% |
| 16 | REGULATION\_OF\_MULTICELLULAR\_ORGANISMAL\_PROCESS |  | 131 | -0.31 | -1.70 | 0.002 | 0.159 | 0.857 | 3062 | tags=37%, list=23%, signal=48% |
| 17 | HUMORAL\_IMMUNE\_RESPONSE |  | 30 | -0.42 | -1.69 | 0.011 | 0.156 | 0.867 | 3099 | tags=53%, list=24%, signal=70% |
| 18 | REGULATION\_OF\_DEFENSE\_RESPONSE |  | 15 | -0.52 | -1.69 | 0.025 | 0.155 | 0.875 | 4058 | tags=67%, list=31%, signal=97% |
| 19 | HEMOPOIESIS |  | 69 | -0.34 | -1.69 | 0.004 | 0.148 | 0.878 | 2973 | tags=39%, list=23%, signal=50% |
| 20 | IMMUNE\_EFFECTOR\_PROCESS |  | 34 | -0.40 | -1.66 | 0.009 | 0.173 | 0.928 | 2811 | tags=50%, list=21%, signal=64% |
| 21 | TRANSFORMING\_GROWTH\_FACTOR\_BETA\_RECEPTOR\_SIGNALING\_PATHWAY |  | 34 | -0.40 | -1.65 | 0.020 | 0.172 | 0.932 | 3055 | tags=44%, list=23%, signal=57% |
| 22 | LIPID\_CATABOLIC\_PROCESS |  | 34 | -0.39 | -1.65 | 0.013 | 0.168 | 0.936 | 4062 | tags=56%, list=31%, signal=81% |
| 23 | IMMUNE\_SYSTEM\_DEVELOPMENT |  | 75 | -0.33 | -1.64 | 0.002 | 0.167 | 0.941 | 2973 | tags=39%, list=23%, signal=50% |
| 24 | RESPONSE\_TO\_EXTERNAL\_STIMULUS |  | 278 | -0.26 | -1.63 | 0.000 | 0.168 | 0.949 | 3111 | tags=33%, list=24%, signal=43% |
| 25 | RECEPTOR\_MEDIATED\_ENDOCYTOSIS |  | 31 | -0.41 | -1.60 | 0.011 | 0.207 | 0.980 | 2244 | tags=35%, list=17%, signal=43% |
| 26 | LYMPHOCYTE\_ACTIVATION |  | 54 | -0.34 | -1.58 | 0.009 | 0.228 | 0.987 | 3366 | tags=46%, list=26%, signal=62% |
| 27 | LEUKOCYTE\_DIFFERENTIATION |  | 34 | -0.38 | -1.56 | 0.009 | 0.250 | 0.994 | 2919 | tags=44%, list=22%, signal=57% |
| 28 | JAK\_STAT\_CASCADE |  | 26 | -0.40 | -1.56 | 0.036 | 0.242 | 0.994 | 3101 | tags=46%, list=24%, signal=60% |
| 29 | CELL\_ACTIVATION |  | 64 | -0.32 | -1.56 | 0.007 | 0.241 | 0.995 | 4058 | tags=52%, list=31%, signal=74% |
| 30 | REGULATION\_OF\_ANGIOGENESIS |  | 24 | -0.42 | -1.56 | 0.037 | 0.236 | 0.996 | 1844 | tags=42%, list=14%, signal=48% |
| 31 | ENZYME\_LINKED\_RECEPTOR\_PROTEIN\_SIGNALING\_PATHWAY |  | 128 | -0.28 | -1.55 | 0.005 | 0.235 | 0.997 | 2819 | tags=30%, list=22%, signal=38% |
| 32 | ACTIN\_CYTOSKELETON\_ORGANIZATION\_AND\_BIOGENESIS |  | 90 | -0.30 | -1.54 | 0.015 | 0.249 | 0.999 | 1965 | tags=27%, list=15%, signal=31% |
| 33 | POSITIVE\_REGULATION\_OF\_RESPONSE\_TO\_STIMULUS |  | 35 | -0.36 | -1.53 | 0.031 | 0.247 | 0.999 | 3040 | tags=46%, list=23%, signal=59% |
| 34 | T\_CELL\_ACTIVATION |  | 39 | -0.35 | -1.53 | 0.031 | 0.253 | 0.999 | 4058 | tags=51%, list=31%, signal=74% |
| 35 | TRANSMEMBRANE\_RECEPTOR\_PROTEIN\_SERINE\_THREONINE\_KINASE\_SIGNALING\_PATHWAY |  | 42 | -0.35 | -1.52 | 0.025 | 0.259 | 0.999 | 3197 | tags=40%, list=24%, signal=53% |
| 36 | POSITIVE\_REGULATION\_OF\_PHOSPHATE\_METABOLIC\_PROCESS |  | 23 | -0.40 | -1.50 | 0.041 | 0.280 | 0.999 | 1376 | tags=30%, list=11%, signal=34% |
| 37 | LEUKOCYTE\_ACTIVATION |  | 59 | -0.31 | -1.49 | 0.020 | 0.302 | 0.999 | 3366 | tags=44%, list=26%, signal=59% |
| 38 | LYMPHOCYTE\_DIFFERENTIATION |  | 23 | -0.41 | -1.48 | 0.054 | 0.301 | 1.000 | 3284 | tags=52%, list=25%, signal=70% |
| 39 | POSITIVE\_REGULATION\_OF\_CELL\_DIFFERENTIATION |  | 21 | -0.41 | -1.48 | 0.059 | 0.296 | 1.000 | 4480 | tags=67%, list=34%, signal=101% |
| 40 | CELLULAR\_LIPID\_CATABOLIC\_PROCESS |  | 31 | -0.36 | -1.48 | 0.051 | 0.293 | 1.000 | 4046 | tags=52%, list=31%, signal=75% |
| 41 | NEGATIVE\_REGULATION\_OF\_SIGNAL\_TRANSDUCTION |  | 31 | -0.37 | -1.48 | 0.048 | 0.287 | 1.000 | 5191 | tags=65%, list=40%, signal=107% |
| 42 | REGULATION\_OF\_RESPONSE\_TO\_STIMULUS |  | 49 | -0.32 | -1.47 | 0.024 | 0.297 | 1.000 | 4389 | tags=59%, list=34%, signal=89% |
| 43 | WOUND\_HEALING |  | 49 | -0.33 | -1.47 | 0.038 | 0.292 | 1.000 | 4014 | tags=45%, list=31%, signal=65% |
| 44 | INNATE\_IMMUNE\_RESPONSE |  | 19 | -0.41 | -1.46 | 0.058 | 0.295 | 1.000 | 4823 | tags=74%, list=37%, signal=116% |
| 45 | B\_CELL\_ACTIVATION |  | 17 | -0.43 | -1.45 | 0.070 | 0.307 | 1.000 | 3366 | tags=59%, list=26%, signal=79% |
| 46 | MESODERM\_DEVELOPMENT |  | 22 | -0.40 | -1.45 | 0.069 | 0.301 | 1.000 | 4352 | tags=55%, list=33%, signal=82% |
| 47 | AMINE\_TRANSPORT |  | 36 | -0.35 | -1.44 | 0.046 | 0.322 | 1.000 | 2107 | tags=28%, list=16%, signal=33% |
| 48 | PROTEIN\_AMINO\_ACID\_PHOSPHORYLATION |  | 231 | -0.23 | -1.43 | 0.000 | 0.329 | 1.000 | 3151 | tags=31%, list=24%, signal=40% |
| 49 | POSITIVE\_REGULATION\_OF\_SIGNAL\_TRANSDUCTION |  | 97 | -0.27 | -1.43 | 0.014 | 0.326 | 1.000 | 4051 | tags=47%, list=31%, signal=68% |
| 50 | CYTOKINE\_AND\_CHEMOKINE\_MEDIATED\_SIGNALING\_PATHWAY |  | 19 | -0.41 | -1.42 | 0.067 | 0.334 | 1.000 | 2209 | tags=37%, list=17%, signal=44% |
| 51 | RESPONSE\_TO\_OTHER\_ORGANISM |  | 69 | -0.29 | -1.42 | 0.046 | 0.329 | 1.000 | 2921 | tags=38%, list=22%, signal=48% |
| 52 | PROTEIN\_AMINO\_ACID\_N\_LINKED\_GLYCOSYLATION |  | 27 | -0.36 | -1.42 | 0.055 | 0.324 | 1.000 | 2544 | tags=37%, list=19%, signal=46% |
| 53 | FATTY\_ACID\_METABOLIC\_PROCESS |  | 56 | -0.30 | -1.42 | 0.053 | 0.326 | 1.000 | 4046 | tags=50%, list=31%, signal=72% |
| 54 | COAGULATION |  | 41 | -0.32 | -1.41 | 0.050 | 0.324 | 1.000 | 3453 | tags=39%, list=26%, signal=53% |
| 55 | BLOOD\_COAGULATION |  | 41 | -0.32 | -1.41 | 0.075 | 0.327 | 1.000 | 3453 | tags=39%, list=26%, signal=53% |
| 56 | CELL\_SUBSTRATE\_ADHESION |  | 36 | -0.33 | -1.41 | 0.076 | 0.323 | 1.000 | 1711 | tags=31%, list=13%, signal=35% |
| 57 | REGULATION\_OF\_SIGNAL\_TRANSDUCTION |  | 173 | -0.24 | -1.40 | 0.024 | 0.331 | 1.000 | 3921 | tags=41%, list=30%, signal=58% |
| 58 | POSITIVE\_REGULATION\_OF\_PROTEIN\_AMINO\_ACID\_PHOSPHORYLATION |  | 15 | -0.43 | -1.40 | 0.086 | 0.327 | 1.000 | 1376 | tags=33%, list=11%, signal=37% |
| 59 | SMALL\_GTPASE\_MEDIATED\_SIGNAL\_TRANSDUCTION |  | 77 | -0.28 | -1.40 | 0.024 | 0.326 | 1.000 | 3701 | tags=43%, list=28%, signal=59% |
| 60 | MULTI\_ORGANISM\_PROCESS |  | 137 | -0.25 | -1.39 | 0.024 | 0.333 | 1.000 | 3460 | tags=38%, list=26%, signal=51% |
| 61 | GROWTH |  | 59 | -0.29 | -1.39 | 0.053 | 0.333 | 1.000 | 4210 | tags=46%, list=32%, signal=67% |
| 62 | CATION\_HOMEOSTASIS |  | 94 | -0.26 | -1.39 | 0.035 | 0.329 | 1.000 | 3259 | tags=36%, list=25%, signal=48% |
| 63 | REGULATION\_OF\_PROTEIN\_AMINO\_ACID\_PHOSPHORYLATION |  | 23 | -0.38 | -1.39 | 0.078 | 0.329 | 1.000 | 2653 | tags=35%, list=20%, signal=44% |
| 64 | REGULATION\_OF\_BLOOD\_PRESSURE |  | 22 | -0.37 | -1.38 | 0.098 | 0.338 | 1.000 | 3452 | tags=41%, list=26%, signal=55% |
| 65 | REGULATION\_OF\_ANATOMICAL\_STRUCTURE\_MORPHOGENESIS |  | 17 | -0.40 | -1.37 | 0.104 | 0.341 | 1.000 | 4547 | tags=53%, list=35%, signal=81% |
| 66 | PEPTIDYL\_TYROSINE\_MODIFICATION |  | 23 | -0.38 | -1.37 | 0.080 | 0.337 | 1.000 | 1577 | tags=26%, list=12%, signal=30% |
| 67 | ANATOMICAL\_STRUCTURE\_FORMATION |  | 52 | -0.29 | -1.36 | 0.072 | 0.355 | 1.000 | 1844 | tags=29%, list=14%, signal=33% |
| 68 | AMINO\_ACID\_TRANSPORT |  | 25 | -0.35 | -1.36 | 0.090 | 0.350 | 1.000 | 2107 | tags=32%, list=16%, signal=38% |
| 69 | REGULATION\_OF\_LYMPHOCYTE\_ACTIVATION |  | 31 | -0.34 | -1.36 | 0.091 | 0.353 | 1.000 | 3284 | tags=45%, list=25%, signal=60% |
| 70 | DETECTION\_OF\_STIMULUS |  | 36 | -0.32 | -1.36 | 0.086 | 0.349 | 1.000 | 5290 | tags=58%, list=40%, signal=98% |
| 71 | CELLULAR\_CATION\_HOMEOSTASIS |  | 91 | -0.26 | -1.35 | 0.058 | 0.349 | 1.000 | 3259 | tags=36%, list=25%, signal=48% |
| 72 | ACTIN\_FILAMENT\_BASED\_PROCESS |  | 99 | -0.26 | -1.35 | 0.039 | 0.345 | 1.000 | 1965 | tags=24%, list=15%, signal=28% |
| 73 | FEMALE\_PREGNANCY |  | 42 | -0.31 | -1.35 | 0.101 | 0.344 | 1.000 | 4407 | tags=55%, list=34%, signal=82% |
| 74 | MAINTENANCE\_OF\_LOCALIZATION |  | 21 | -0.38 | -1.35 | 0.117 | 0.344 | 1.000 | 2710 | tags=38%, list=21%, signal=48% |
| 75 | TRANSMEMBRANE\_RECEPTOR\_PROTEIN\_TYROSINE\_KINASE\_SIGNALING\_PATHWAY |  | 76 | -0.27 | -1.35 | 0.063 | 0.340 | 1.000 | 1386 | tags=21%, list=11%, signal=23% |
| 76 | RESPONSE\_TO\_VIRUS |  | 45 | -0.30 | -1.34 | 0.086 | 0.358 | 1.000 | 2921 | tags=42%, list=22%, signal=54% |
| 77 | HEMOSTASIS |  | 46 | -0.30 | -1.33 | 0.077 | 0.367 | 1.000 | 1871 | tags=26%, list=14%, signal=30% |
| 78 | POSITIVE\_REGULATION\_OF\_CYTOKINE\_BIOSYNTHETIC\_PROCESS |  | 21 | -0.37 | -1.33 | 0.118 | 0.373 | 1.000 | 2909 | tags=43%, list=22%, signal=55% |
| 79 | PROTEIN\_AMINO\_ACID\_DEPHOSPHORYLATION |  | 60 | -0.28 | -1.31 | 0.099 | 0.393 | 1.000 | 1498 | tags=22%, list=11%, signal=24% |
| 80 | GLYCOPROTEIN\_METABOLIC\_PROCESS |  | 82 | -0.26 | -1.31 | 0.060 | 0.389 | 1.000 | 3672 | tags=39%, list=28%, signal=54% |
| 81 | MUSCLE\_DEVELOPMENT |  | 85 | -0.26 | -1.31 | 0.066 | 0.388 | 1.000 | 3435 | tags=40%, list=26%, signal=54% |
| 82 | POSITIVE\_REGULATION\_OF\_PHOSPHORYLATION |  | 21 | -0.37 | -1.31 | 0.136 | 0.385 | 1.000 | 1376 | tags=29%, list=11%, signal=32% |
| 83 | REGULATION\_OF\_BODY\_FLUID\_LEVELS |  | 55 | -0.28 | -1.31 | 0.092 | 0.386 | 1.000 | 1871 | tags=25%, list=14%, signal=30% |
| 84 | PROTEIN\_KINASE\_CASCADE |  | 239 | -0.21 | -1.30 | 0.031 | 0.400 | 1.000 | 2264 | tags=25%, list=17%, signal=30% |
| 85 | POSITIVE\_REGULATION\_OF\_LYMPHOCYTE\_ACTIVATION |  | 23 | -0.35 | -1.30 | 0.124 | 0.402 | 1.000 | 3284 | tags=43%, list=25%, signal=58% |
| 86 | REGULATION\_OF\_T\_CELL\_ACTIVATION |  | 25 | -0.33 | -1.29 | 0.120 | 0.403 | 1.000 | 3284 | tags=44%, list=25%, signal=59% |
| 87 | RESPONSE\_TO\_DRUG |  | 21 | -0.36 | -1.29 | 0.149 | 0.399 | 1.000 | 2530 | tags=43%, list=19%, signal=53% |
| 88 | ANGIOGENESIS |  | 44 | -0.29 | -1.29 | 0.121 | 0.402 | 1.000 | 1844 | tags=30%, list=14%, signal=34% |
| 89 | PROTEIN\_PROCESSING |  | 41 | -0.29 | -1.29 | 0.111 | 0.408 | 1.000 | 4051 | tags=41%, list=31%, signal=60% |
| 90 | DEPHOSPHORYLATION |  | 67 | -0.26 | -1.28 | 0.095 | 0.407 | 1.000 | 1498 | tags=21%, list=11%, signal=23% |
| 91 | PROTEIN\_COMPLEX\_ASSEMBLY |  | 157 | -0.22 | -1.28 | 0.049 | 0.402 | 1.000 | 2436 | tags=26%, list=19%, signal=32% |
| 92 | CELL\_MATRIX\_ADHESION |  | 35 | -0.31 | -1.28 | 0.133 | 0.413 | 1.000 | 1711 | tags=29%, list=13%, signal=33% |
| 93 | POSITIVE\_REGULATION\_OF\_SECRETION |  | 18 | -0.37 | -1.28 | 0.158 | 0.409 | 1.000 | 4314 | tags=61%, list=33%, signal=91% |
| 94 | CYTOKINE\_PRODUCTION |  | 61 | -0.27 | -1.27 | 0.100 | 0.426 | 1.000 | 2909 | tags=34%, list=22%, signal=44% |
| 95 | PHOSPHOLIPID\_METABOLIC\_PROCESS |  | 63 | -0.26 | -1.26 | 0.107 | 0.436 | 1.000 | 3843 | tags=43%, list=29%, signal=60% |
| 96 | RAS\_PROTEIN\_SIGNAL\_TRANSDUCTION |  | 55 | -0.27 | -1.26 | 0.127 | 0.436 | 1.000 | 3701 | tags=44%, list=28%, signal=61% |
| 97 | REGULATION\_OF\_CYTOSKELETON\_ORGANIZATION\_AND\_BIOGENESIS |  | 26 | -0.32 | -1.25 | 0.152 | 0.442 | 1.000 | 2653 | tags=35%, list=20%, signal=43% |
| 98 | PROTEIN\_OLIGOMERIZATION |  | 37 | -0.31 | -1.25 | 0.128 | 0.439 | 1.000 | 2571 | tags=32%, list=20%, signal=40% |
| 99 | MONOCARBOXYLIC\_ACID\_METABOLIC\_PROCESS |  | 77 | -0.25 | -1.25 | 0.093 | 0.439 | 1.000 | 4406 | tags=48%, list=34%, signal=72% |
| 100 | LIPID\_METABOLIC\_PROCESS |  | 283 | -0.20 | -1.25 | 0.047 | 0.446 | 1.000 | 3925 | tags=39%, list=30%, signal=54% |
| 101 | PEPTIDYL\_TYROSINE\_PHOSPHORYLATION |  | 21 | -0.34 | -1.25 | 0.169 | 0.444 | 1.000 | 3101 | tags=33%, list=24%, signal=44% |
| 102 | ACTIN\_POLYMERIZATION\_AND\_OR\_DEPOLYMERIZATION |  | 20 | -0.35 | -1.24 | 0.191 | 0.442 | 1.000 | 2436 | tags=30%, list=19%, signal=37% |
| 103 | REGULATION\_OF\_CELL\_PROLIFERATION |  | 275 | -0.20 | -1.24 | 0.031 | 0.439 | 1.000 | 2676 | tags=26%, list=20%, signal=32% |
| 104 | ICOSANOID\_METABOLIC\_PROCESS |  | 16 | -0.38 | -1.23 | 0.227 | 0.464 | 1.000 | 3681 | tags=50%, list=28%, signal=69% |
| 105 | POSITIVE\_REGULATION\_OF\_TRANSLATION |  | 28 | -0.31 | -1.23 | 0.178 | 0.463 | 1.000 | 2909 | tags=39%, list=22%, signal=50% |
| 106 | NEGATIVE\_REGULATION\_OF\_TRANSCRIPTION |  | 166 | -0.21 | -1.23 | 0.094 | 0.465 | 1.000 | 2865 | tags=30%, list=22%, signal=37% |
| 107 | PHOSPHORYLATION |  | 262 | -0.20 | -1.22 | 0.084 | 0.472 | 1.000 | 3151 | tags=30%, list=24%, signal=38% |
| 108 | PROTEIN\_AUTOPROCESSING |  | 24 | -0.32 | -1.22 | 0.182 | 0.473 | 1.000 | 4051 | tags=46%, list=31%, signal=66% |
| 109 | VASCULATURE\_DEVELOPMENT |  | 50 | -0.26 | -1.22 | 0.150 | 0.473 | 1.000 | 1844 | tags=26%, list=14%, signal=30% |
| 110 | REGULATION\_OF\_I\_KAPPAB\_KINASE\_NF\_KAPPAB\_CASCADE |  | 72 | -0.25 | -1.22 | 0.135 | 0.471 | 1.000 | 3879 | tags=46%, list=30%, signal=65% |
| 111 | POSITIVE\_REGULATION\_OF\_TRANSFERASE\_ACTIVITY |  | 71 | -0.25 | -1.22 | 0.129 | 0.470 | 1.000 | 2431 | tags=27%, list=19%, signal=33% |
| 112 | G\_PROTEIN\_SIGNALING\_COUPLED\_TO\_CAMP\_NUCLEOTIDE\_SECOND\_MESSENGER |  | 62 | -0.25 | -1.22 | 0.164 | 0.467 | 1.000 | 2122 | tags=21%, list=16%, signal=25% |
| 113 | CAMP\_MEDIATED\_SIGNALING |  | 63 | -0.25 | -1.22 | 0.152 | 0.463 | 1.000 | 2122 | tags=21%, list=16%, signal=25% |
| 114 | ORGAN\_MORPHOGENESIS |  | 131 | -0.22 | -1.21 | 0.109 | 0.479 | 1.000 | 1485 | tags=19%, list=11%, signal=21% |
| 115 | PROTEIN\_AMINO\_ACID\_AUTOPHOSPHORYLATION |  | 24 | -0.32 | -1.21 | 0.188 | 0.481 | 1.000 | 4051 | tags=46%, list=31%, signal=66% |
| 116 | MUSCLE\_CELL\_DIFFERENTIATION |  | 21 | -0.33 | -1.20 | 0.209 | 0.479 | 1.000 | 3435 | tags=48%, list=26%, signal=64% |
| 117 | POSITIVE\_REGULATION\_OF\_CELL\_PROLIFERATION |  | 129 | -0.22 | -1.20 | 0.146 | 0.494 | 1.000 | 2061 | tags=23%, list=16%, signal=27% |
| 118 | RESPONSE\_TO\_BACTERIUM |  | 22 | -0.32 | -1.20 | 0.231 | 0.493 | 1.000 | 1844 | tags=27%, list=14%, signal=32% |
| 119 | REGULATION\_OF\_MYELOID\_CELL\_DIFFERENTIATION |  | 19 | -0.34 | -1.19 | 0.239 | 0.511 | 1.000 | 4546 | tags=58%, list=35%, signal=89% |
| 120 | CELL\_RECOGNITION |  | 16 | -0.36 | -1.19 | 0.239 | 0.509 | 1.000 | 4058 | tags=50%, list=31%, signal=72% |
| 121 | AMINO\_ACID\_CATABOLIC\_PROCESS |  | 23 | -0.32 | -1.18 | 0.233 | 0.513 | 1.000 | 2200 | tags=35%, list=17%, signal=42% |
| 122 | REGULATION\_OF\_PROTEIN\_METABOLIC\_PROCESS |  | 150 | -0.21 | -1.17 | 0.146 | 0.532 | 1.000 | 2738 | tags=28%, list=21%, signal=35% |
| 123 | GLYCEROPHOSPHOLIPID\_METABOLIC\_PROCESS |  | 39 | -0.27 | -1.17 | 0.232 | 0.530 | 1.000 | 3843 | tags=44%, list=29%, signal=62% |
| 124 | BONE\_REMODELING |  | 28 | -0.30 | -1.17 | 0.242 | 0.527 | 1.000 | 2819 | tags=32%, list=22%, signal=41% |
| 125 | POSITIVE\_REGULATION\_OF\_CELLULAR\_PROTEIN\_METABOLIC\_PROCESS |  | 61 | -0.24 | -1.17 | 0.201 | 0.524 | 1.000 | 2436 | tags=30%, list=19%, signal=36% |
| 126 | REGULATION\_OF\_MAP\_KINASE\_ACTIVITY |  | 56 | -0.25 | -1.17 | 0.210 | 0.526 | 1.000 | 2086 | tags=29%, list=16%, signal=34% |
| 127 | POSITIVE\_REGULATION\_OF\_PROTEIN\_METABOLIC\_PROCESS |  | 63 | -0.24 | -1.17 | 0.218 | 0.525 | 1.000 | 2436 | tags=30%, list=19%, signal=37% |
| 128 | GENERATION\_OF\_PRECURSOR\_METABOLITES\_AND\_ENERGY |  | 120 | -0.22 | -1.17 | 0.161 | 0.524 | 1.000 | 3160 | tags=32%, list=24%, signal=41% |
| 129 | ACTIVATION\_OF\_NF\_KAPPAB\_TRANSCRIPTION\_FACTOR |  | 15 | -0.36 | -1.17 | 0.235 | 0.524 | 1.000 | 4271 | tags=60%, list=33%, signal=89% |
| 130 | POSITIVE\_REGULATION\_OF\_PROTEIN\_MODIFICATION\_PROCESS |  | 24 | -0.31 | -1.16 | 0.246 | 0.525 | 1.000 | 1376 | tags=25%, list=11%, signal=28% |
| 131 | BEHAVIOR |  | 136 | -0.21 | -1.16 | 0.187 | 0.523 | 1.000 | 4483 | tags=43%, list=34%, signal=64% |
| 132 | REGULATION\_OF\_ORGANELLE\_ORGANIZATION\_AND\_BIOGENESIS |  | 35 | -0.28 | -1.16 | 0.262 | 0.522 | 1.000 | 1732 | tags=26%, list=13%, signal=30% |
| 133 | NEGATIVE\_REGULATION\_OF\_RNA\_METABOLIC\_PROCESS |  | 114 | -0.21 | -1.16 | 0.165 | 0.520 | 1.000 | 2865 | tags=30%, list=22%, signal=38% |
| 134 | ACTIVATION\_OF\_MAPK\_ACTIVITY |  | 33 | -0.28 | -1.16 | 0.234 | 0.520 | 1.000 | 2086 | tags=30%, list=16%, signal=36% |
| 135 | NEURON\_DIFFERENTIATION |  | 58 | -0.25 | -1.15 | 0.208 | 0.529 | 1.000 | 3461 | tags=33%, list=26%, signal=44% |
| 136 | VITAMIN\_METABOLIC\_PROCESS |  | 15 | -0.36 | -1.15 | 0.263 | 0.525 | 1.000 | 4119 | tags=60%, list=31%, signal=87% |
| 137 | GENERATION\_OF\_NEURONS |  | 65 | -0.24 | -1.15 | 0.206 | 0.536 | 1.000 | 3512 | tags=34%, list=27%, signal=46% |
| 138 | POSITIVE\_REGULATION\_OF\_I\_KAPPAB\_KINASE\_NF\_KAPPAB\_CASCADE |  | 67 | -0.23 | -1.15 | 0.207 | 0.535 | 1.000 | 3879 | tags=45%, list=30%, signal=63% |
| 139 | DEVELOPMENTAL\_MATURATION |  | 18 | -0.34 | -1.15 | 0.260 | 0.531 | 1.000 | 3178 | tags=39%, list=24%, signal=51% |
| 140 | NEGATIVE\_REGULATION\_OF\_TRANSCRIPTION\_DNA\_DEPENDENT |  | 114 | -0.21 | -1.15 | 0.209 | 0.529 | 1.000 | 2865 | tags=30%, list=22%, signal=38% |
| 141 | SKELETAL\_DEVELOPMENT |  | 91 | -0.22 | -1.14 | 0.227 | 0.537 | 1.000 | 3067 | tags=32%, list=23%, signal=41% |
| 142 | STRIATED\_MUSCLE\_DEVELOPMENT |  | 36 | -0.27 | -1.14 | 0.261 | 0.542 | 1.000 | 3625 | tags=44%, list=28%, signal=61% |
| 143 | POSITIVE\_REGULATION\_OF\_T\_CELL\_ACTIVATION |  | 20 | -0.32 | -1.14 | 0.296 | 0.547 | 1.000 | 4546 | tags=55%, list=35%, signal=84% |
| 144 | MEMBRANE\_ORGANIZATION\_AND\_BIOGENESIS |  | 124 | -0.20 | -1.13 | 0.216 | 0.544 | 1.000 | 3554 | tags=35%, list=27%, signal=48% |
| 145 | AMINO\_ACID\_METABOLIC\_PROCESS |  | 73 | -0.23 | -1.13 | 0.230 | 0.549 | 1.000 | 2224 | tags=27%, list=17%, signal=33% |
| 146 | AMINE\_CATABOLIC\_PROCESS |  | 25 | -0.30 | -1.13 | 0.284 | 0.547 | 1.000 | 2200 | tags=32%, list=17%, signal=38% |
| 147 | REGULATION\_OF\_CELLULAR\_PROTEIN\_METABOLIC\_PROCESS |  | 139 | -0.20 | -1.13 | 0.217 | 0.553 | 1.000 | 2738 | tags=27%, list=21%, signal=34% |
| 148 | FATTY\_ACID\_OXIDATION |  | 17 | -0.33 | -1.13 | 0.275 | 0.549 | 1.000 | 3355 | tags=47%, list=26%, signal=63% |
| 149 | CYTOKINE\_BIOSYNTHETIC\_PROCESS |  | 34 | -0.27 | -1.12 | 0.273 | 0.550 | 1.000 | 2909 | tags=35%, list=22%, signal=45% |
| 150 | REGULATION\_OF\_PROTEIN\_IMPORT\_INTO\_NUCLEUS |  | 15 | -0.34 | -1.12 | 0.289 | 0.550 | 1.000 | 1405 | tags=27%, list=11%, signal=30% |
| 151 | NEGATIVE\_REGULATION\_OF\_NUCLEOBASENUCLEOSIDENUCLEOTIDE\_AND\_NUCLEIC\_ACID\_METABOLIC\_PROCESS |  | 185 | -0.19 | -1.12 | 0.188 | 0.551 | 1.000 | 2865 | tags=29%, list=22%, signal=36% |
| 152 | PROTEIN\_SECRETION |  | 28 | -0.28 | -1.12 | 0.289 | 0.549 | 1.000 | 4154 | tags=46%, list=32%, signal=68% |
| 153 | NEGATIVE\_REGULATION\_OF\_CELL\_DIFFERENTIATION |  | 24 | -0.30 | -1.12 | 0.297 | 0.547 | 1.000 | 2782 | tags=29%, list=21%, signal=37% |
| 154 | ACTIN\_FILAMENT\_ORGANIZATION |  | 21 | -0.30 | -1.12 | 0.318 | 0.547 | 1.000 | 1617 | tags=29%, list=12%, signal=33% |
| 155 | REGULATION\_OF\_MAPKKK\_CASCADE |  | 19 | -0.33 | -1.12 | 0.313 | 0.549 | 1.000 | 717 | tags=21%, list=5%, signal=22% |
| 156 | RESPONSE\_TO\_BIOTIC\_STIMULUS |  | 103 | -0.21 | -1.12 | 0.252 | 0.548 | 1.000 | 2921 | tags=32%, list=22%, signal=41% |
| 157 | GLYCOPROTEIN\_BIOSYNTHETIC\_PROCESS |  | 67 | -0.23 | -1.11 | 0.249 | 0.547 | 1.000 | 3835 | tags=39%, list=29%, signal=55% |
| 158 | CYTOKINE\_SECRETION |  | 15 | -0.35 | -1.11 | 0.317 | 0.550 | 1.000 | 3413 | tags=47%, list=26%, signal=63% |
| 159 | SODIUM\_ION\_TRANSPORT |  | 17 | -0.33 | -1.11 | 0.311 | 0.549 | 1.000 | 5152 | tags=59%, list=39%, signal=97% |
| 160 | CARBOXYLIC\_ACID\_METABOLIC\_PROCESS |  | 160 | -0.19 | -1.11 | 0.228 | 0.557 | 1.000 | 3940 | tags=38%, list=30%, signal=54% |
| 161 | ORGANIC\_ACID\_METABOLIC\_PROCESS |  | 162 | -0.19 | -1.11 | 0.235 | 0.555 | 1.000 | 3940 | tags=38%, list=30%, signal=53% |
| 162 | AMINO\_ACID\_DERIVATIVE\_METABOLIC\_PROCESS |  | 23 | -0.30 | -1.11 | 0.299 | 0.553 | 1.000 | 4046 | tags=48%, list=31%, signal=69% |
| 163 | PHAGOCYTOSIS |  | 16 | -0.34 | -1.11 | 0.294 | 0.550 | 1.000 | 3547 | tags=50%, list=27%, signal=68% |
| 164 | TISSUE\_REMODELING |  | 29 | -0.28 | -1.10 | 0.308 | 0.549 | 1.000 | 2819 | tags=31%, list=22%, signal=39% |
| 165 | NEGATIVE\_REGULATION\_OF\_TRANSCRIPTION\_FROM\_RNA\_POLYMERASE\_II\_PROMOTER |  | 76 | -0.22 | -1.10 | 0.265 | 0.550 | 1.000 | 2865 | tags=30%, list=22%, signal=39% |
| 166 | POST\_TRANSLATIONAL\_PROTEIN\_MODIFICATION |  | 409 | -0.17 | -1.10 | 0.164 | 0.549 | 1.000 | 2870 | tags=25%, list=22%, signal=32% |
| 167 | NITROGEN\_COMPOUND\_CATABOLIC\_PROCESS |  | 27 | -0.29 | -1.10 | 0.300 | 0.547 | 1.000 | 2200 | tags=30%, list=17%, signal=36% |
| 168 | MYELOID\_CELL\_DIFFERENTIATION |  | 35 | -0.26 | -1.10 | 0.305 | 0.545 | 1.000 | 2973 | tags=31%, list=23%, signal=41% |
| 169 | NEGATIVE\_REGULATION\_OF\_CELL\_PROLIFERATION |  | 145 | -0.19 | -1.10 | 0.216 | 0.543 | 1.000 | 2676 | tags=26%, list=20%, signal=33% |
| 170 | CELLULAR\_LIPID\_METABOLIC\_PROCESS |  | 220 | -0.18 | -1.10 | 0.212 | 0.545 | 1.000 | 3925 | tags=38%, list=30%, signal=54% |
| 171 | TISSUE\_DEVELOPMENT |  | 126 | -0.20 | -1.10 | 0.255 | 0.544 | 1.000 | 1536 | tags=19%, list=12%, signal=21% |
| 172 | REGULATION\_OF\_DEVELOPMENTAL\_PROCESS |  | 387 | -0.17 | -1.10 | 0.183 | 0.542 | 1.000 | 4391 | tags=43%, list=34%, signal=62% |
| 173 | I\_KAPPAB\_KINASE\_NF\_KAPPAB\_CASCADE |  | 88 | -0.21 | -1.09 | 0.267 | 0.548 | 1.000 | 3879 | tags=42%, list=30%, signal=59% |
| 174 | NEGATIVE\_REGULATION\_OF\_METABOLIC\_PROCESS |  | 232 | -0.18 | -1.09 | 0.239 | 0.552 | 1.000 | 2865 | tags=27%, list=22%, signal=34% |
| 175 | MEMBRANE\_LIPID\_METABOLIC\_PROCESS |  | 85 | -0.21 | -1.09 | 0.285 | 0.554 | 1.000 | 3843 | tags=40%, list=29%, signal=56% |
| 176 | NEURON\_DEVELOPMENT |  | 49 | -0.24 | -1.09 | 0.297 | 0.558 | 1.000 | 3461 | tags=33%, list=26%, signal=44% |
| 177 | CELL\_MATURATION |  | 16 | -0.32 | -1.08 | 0.363 | 0.572 | 1.000 | 3178 | tags=38%, list=24%, signal=49% |
| 178 | ION\_HOMEOSTASIS |  | 112 | -0.20 | -1.08 | 0.284 | 0.575 | 1.000 | 3259 | tags=32%, list=25%, signal=42% |
| 179 | POSITIVE\_REGULATION\_OF\_TRANSCRIPTION |  | 124 | -0.19 | -1.08 | 0.284 | 0.575 | 1.000 | 3292 | tags=31%, list=25%, signal=41% |
| 180 | REGULATION\_OF\_BIOLOGICAL\_QUALITY |  | 364 | -0.17 | -1.07 | 0.239 | 0.574 | 1.000 | 4014 | tags=34%, list=31%, signal=47% |
| 181 | ELECTRON\_TRANSPORT\_GO\_0006118 |  | 50 | -0.24 | -1.07 | 0.337 | 0.583 | 1.000 | 2240 | tags=26%, list=17%, signal=31% |
| 182 | POSITIVE\_REGULATION\_OF\_MAP\_KINASE\_ACTIVITY |  | 39 | -0.26 | -1.07 | 0.341 | 0.581 | 1.000 | 2086 | tags=28%, list=16%, signal=33% |
| 183 | DETECTION\_OF\_EXTERNAL\_STIMULUS |  | 18 | -0.32 | -1.07 | 0.365 | 0.583 | 1.000 | 8972 | tags=100%, list=69%, signal=317% |
| 184 | CYTOKINE\_METABOLIC\_PROCESS |  | 35 | -0.26 | -1.07 | 0.352 | 0.586 | 1.000 | 2909 | tags=34%, list=22%, signal=44% |
| 185 | DEFENSE\_RESPONSE\_TO\_BACTERIUM |  | 16 | -0.32 | -1.06 | 0.365 | 0.585 | 1.000 | 4164 | tags=44%, list=32%, signal=64% |
| 186 | LOCOMOTORY\_BEHAVIOR |  | 84 | -0.21 | -1.06 | 0.335 | 0.584 | 1.000 | 2765 | tags=29%, list=21%, signal=36% |
| 187 | POSITIVE\_REGULATION\_OF\_DEVELOPMENTAL\_PROCESS |  | 197 | -0.18 | -1.06 | 0.324 | 0.594 | 1.000 | 4377 | tags=45%, list=33%, signal=67% |
| 188 | ORGANIC\_ACID\_TRANSPORT |  | 39 | -0.24 | -1.05 | 0.345 | 0.605 | 1.000 | 2107 | tags=26%, list=16%, signal=30% |
| 189 | HORMONE\_METABOLIC\_PROCESS |  | 29 | -0.27 | -1.05 | 0.354 | 0.603 | 1.000 | 3920 | tags=48%, list=30%, signal=69% |
| 190 | CELL\_MIGRATION |  | 82 | -0.21 | -1.05 | 0.359 | 0.602 | 1.000 | 2771 | tags=26%, list=21%, signal=32% |
| 191 | NEGATIVE\_REGULATION\_OF\_CELLULAR\_METABOLIC\_PROCESS |  | 229 | -0.17 | -1.05 | 0.306 | 0.600 | 1.000 | 2865 | tags=27%, list=22%, signal=34% |
| 192 | CARBOXYLIC\_ACID\_TRANSPORT |  | 39 | -0.24 | -1.05 | 0.373 | 0.602 | 1.000 | 2107 | tags=26%, list=16%, signal=30% |
| 193 | MAPKKK\_CASCADE\_GO\_0000165 |  | 90 | -0.20 | -1.05 | 0.364 | 0.600 | 1.000 | 2105 | tags=22%, list=16%, signal=26% |
| 194 | POSITIVE\_REGULATION\_OF\_METABOLIC\_PROCESS |  | 201 | -0.17 | -1.05 | 0.352 | 0.604 | 1.000 | 2275 | tags=23%, list=17%, signal=27% |
| 195 | MYOBLAST\_DIFFERENTIATION |  | 16 | -0.31 | -1.04 | 0.388 | 0.608 | 1.000 | 3435 | tags=50%, list=26%, signal=68% |
| 196 | POSITIVE\_REGULATION\_OF\_CELLULAR\_METABOLIC\_PROCESS |  | 196 | -0.18 | -1.04 | 0.337 | 0.613 | 1.000 | 2275 | tags=23%, list=17%, signal=27% |
| 197 | NEURITE\_DEVELOPMENT |  | 41 | -0.24 | -1.04 | 0.392 | 0.616 | 1.000 | 3461 | tags=32%, list=26%, signal=43% |
| 198 | DETECTION\_OF\_STIMULUS\_INVOLVED\_IN\_SENSORY\_PERCEPTION |  | 15 | -0.32 | -1.03 | 0.422 | 0.627 | 1.000 | 8972 | tags=100%, list=69%, signal=317% |
| 199 | AMINO\_ACID\_AND\_DERIVATIVE\_METABOLIC\_PROCESS |  | 96 | -0.19 | -1.03 | 0.376 | 0.626 | 1.000 | 2327 | tags=25%, list=18%, signal=30% |
| 200 | CELL\_PROLIFERATION\_GO\_0008283 |  | 466 | -0.16 | -1.03 | 0.363 | 0.627 | 1.000 | 2702 | tags=24%, list=21%, signal=29% |
| 201 | AXONOGENESIS |  | 33 | -0.25 | -1.03 | 0.420 | 0.633 | 1.000 | 3461 | tags=33%, list=26%, signal=45% |
| 202 | TRANSLATION |  | 149 | -0.18 | -1.02 | 0.423 | 0.647 | 1.000 | 2909 | tags=29%, list=22%, signal=37% |
| 203 | HEART\_DEVELOPMENT |  | 33 | -0.25 | -1.02 | 0.420 | 0.651 | 1.000 | 4081 | tags=39%, list=31%, signal=57% |
| 204 | NERVOUS\_SYSTEM\_DEVELOPMENT |  | 328 | -0.16 | -1.01 | 0.417 | 0.661 | 1.000 | 4068 | tags=34%, list=31%, signal=47% |
| 205 | POSITIVE\_REGULATION\_OF\_CATALYTIC\_ACTIVITY |  | 139 | -0.18 | -1.01 | 0.446 | 0.666 | 1.000 | 2205 | tags=21%, list=17%, signal=25% |
| 206 | AXON\_GUIDANCE |  | 18 | -0.30 | -1.01 | 0.422 | 0.663 | 1.000 | 2771 | tags=33%, list=21%, signal=42% |
| 207 | MACROMOLECULE\_BIOSYNTHETIC\_PROCESS |  | 267 | -0.16 | -1.01 | 0.435 | 0.665 | 1.000 | 3544 | tags=32%, list=27%, signal=43% |
| 208 | PEPTIDYL\_AMINO\_ACID\_MODIFICATION |  | 47 | -0.23 | -1.01 | 0.424 | 0.669 | 1.000 | 2653 | tags=28%, list=20%, signal=35% |
| 209 | REGULATION\_OF\_TRANSCRIPTION |  | 498 | -0.15 | -1.00 | 0.438 | 0.679 | 1.000 | 2973 | tags=26%, list=23%, signal=32% |
| 210 | INSULIN\_RECEPTOR\_SIGNALING\_PATHWAY |  | 16 | -0.30 | -1.00 | 0.453 | 0.682 | 1.000 | 3531 | tags=44%, list=27%, signal=60% |
| 211 | ANATOMICAL\_STRUCTURE\_MORPHOGENESIS |  | 336 | -0.16 | -0.99 | 0.468 | 0.692 | 1.000 | 3236 | tags=28%, list=25%, signal=36% |
| 212 | REGULATION\_OF\_JNK\_ACTIVITY |  | 18 | -0.29 | -0.99 | 0.462 | 0.694 | 1.000 | 2017 | tags=28%, list=15%, signal=33% |
| 213 | REGULATION\_OF\_PROTEIN\_SECRETION |  | 19 | -0.29 | -0.99 | 0.474 | 0.696 | 1.000 | 4058 | tags=47%, list=31%, signal=69% |
| 214 | POSITIVE\_REGULATION\_OF\_DNA\_BINDING |  | 18 | -0.29 | -0.99 | 0.465 | 0.703 | 1.000 | 4419 | tags=56%, list=34%, signal=84% |
| 215 | POSITIVE\_REGULATION\_OF\_NUCLEOBASENUCLEOSIDENUCLEOTIDE\_AND\_NUCLEIC\_ACID\_METABOLIC\_PROCESS |  | 134 | -0.18 | -0.98 | 0.484 | 0.718 | 1.000 | 3292 | tags=30%, list=25%, signal=39% |
| 216 | POSITIVE\_REGULATION\_OF\_TRANSCRIPTION\_FROM\_RNA\_POLYMERASE\_II\_PROMOTER |  | 60 | -0.21 | -0.98 | 0.482 | 0.715 | 1.000 | 3232 | tags=32%, list=25%, signal=42% |
| 217 | NEUROGENESIS |  | 75 | -0.19 | -0.98 | 0.505 | 0.714 | 1.000 | 3512 | tags=32%, list=27%, signal=43% |
| 218 | CELLULAR\_COMPONENT\_ASSEMBLY |  | 272 | -0.16 | -0.98 | 0.511 | 0.712 | 1.000 | 3178 | tags=28%, list=24%, signal=37% |
| 219 | CELL\_CELL\_ADHESION |  | 72 | -0.20 | -0.98 | 0.490 | 0.716 | 1.000 | 4389 | tags=43%, list=34%, signal=64% |
| 220 | REGULATION\_OF\_CYTOKINE\_BIOSYNTHETIC\_PROCESS |  | 31 | -0.24 | -0.98 | 0.478 | 0.713 | 1.000 | 2909 | tags=32%, list=22%, signal=41% |
| 221 | RESPONSE\_TO\_CHEMICAL\_STIMULUS |  | 271 | -0.16 | -0.97 | 0.504 | 0.711 | 1.000 | 2765 | tags=24%, list=21%, signal=30% |
| 222 | RHYTHMIC\_PROCESS |  | 23 | -0.26 | -0.97 | 0.506 | 0.715 | 1.000 | 2699 | tags=30%, list=21%, signal=38% |
| 223 | EPIDERMIS\_DEVELOPMENT |  | 66 | -0.20 | -0.97 | 0.490 | 0.713 | 1.000 | 1483 | tags=20%, list=11%, signal=22% |
| 224 | POSITIVE\_REGULATION\_OF\_TRANSCRIPTION\_FACTOR\_ACTIVITY |  | 17 | -0.28 | -0.97 | 0.487 | 0.714 | 1.000 | 4271 | tags=53%, list=33%, signal=78% |
| 225 | CELLULAR\_HOMEOSTASIS |  | 121 | -0.17 | -0.96 | 0.544 | 0.733 | 1.000 | 3259 | tags=31%, list=25%, signal=40% |
| 226 | ANTI\_APOPTOSIS |  | 107 | -0.18 | -0.96 | 0.545 | 0.739 | 1.000 | 2284 | tags=25%, list=17%, signal=30% |
| 227 | NEGATIVE\_REGULATION\_OF\_DEVELOPMENTAL\_PROCESS |  | 177 | -0.16 | -0.94 | 0.580 | 0.771 | 1.000 | 2819 | tags=27%, list=22%, signal=33% |
| 228 | REGULATION\_OF\_G\_PROTEIN\_COUPLED\_RECEPTOR\_PROTEIN\_SIGNALING\_PATHWAY |  | 23 | -0.26 | -0.94 | 0.532 | 0.770 | 1.000 | 1062 | tags=17%, list=8%, signal=19% |
| 229 | HOMEOSTATIC\_PROCESS |  | 179 | -0.16 | -0.94 | 0.602 | 0.771 | 1.000 | 3259 | tags=28%, list=25%, signal=37% |
| 230 | RESPONSE\_TO\_OXIDATIVE\_STRESS |  | 38 | -0.22 | -0.94 | 0.564 | 0.782 | 1.000 | 1448 | tags=24%, list=11%, signal=27% |
| 231 | CYCLIC\_NUCLEOTIDE\_MEDIATED\_SIGNALING |  | 97 | -0.18 | -0.94 | 0.578 | 0.781 | 1.000 | 1300 | tags=12%, list=10%, signal=14% |
| 232 | MACROMOLECULAR\_COMPLEX\_ASSEMBLY |  | 254 | -0.15 | -0.93 | 0.666 | 0.790 | 1.000 | 3160 | tags=28%, list=24%, signal=36% |
| 233 | CHEMICAL\_HOMEOSTASIS |  | 136 | -0.17 | -0.92 | 0.606 | 0.804 | 1.000 | 3259 | tags=29%, list=25%, signal=38% |
| 234 | SKELETAL\_MUSCLE\_DEVELOPMENT |  | 28 | -0.23 | -0.92 | 0.588 | 0.806 | 1.000 | 3625 | tags=43%, list=28%, signal=59% |
| 235 | SECRETION\_BY\_CELL |  | 100 | -0.17 | -0.92 | 0.633 | 0.804 | 1.000 | 4449 | tags=42%, list=34%, signal=63% |
| 236 | ECTODERM\_DEVELOPMENT |  | 75 | -0.18 | -0.92 | 0.595 | 0.803 | 1.000 | 1536 | tags=19%, list=12%, signal=21% |
| 237 | REGULATION\_OF\_MUSCLE\_CONTRACTION |  | 18 | -0.27 | -0.92 | 0.541 | 0.810 | 1.000 | 2922 | tags=39%, list=22%, signal=50% |
| 238 | G\_PROTEIN\_SIGNALING\_COUPLED\_TO\_CYCLIC\_NUCLEOTIDE\_SECOND\_MESSENGER |  | 96 | -0.18 | -0.91 | 0.663 | 0.815 | 1.000 | 1300 | tags=13%, list=10%, signal=14% |
| 239 | REGULATION\_OF\_CELLULAR\_COMPONENT\_ORGANIZATION\_AND\_BIOGENESIS |  | 102 | -0.17 | -0.91 | 0.667 | 0.812 | 1.000 | 3625 | tags=33%, list=28%, signal=46% |
| 240 | CELLULAR\_PROTEIN\_COMPLEX\_ASSEMBLY |  | 28 | -0.23 | -0.91 | 0.583 | 0.818 | 1.000 | 2797 | tags=29%, list=21%, signal=36% |
| 241 | NEGATIVE\_REGULATION\_OF\_CELLULAR\_COMPONENT\_ORGANIZATION\_AND\_BIOGENESIS |  | 26 | -0.24 | -0.91 | 0.600 | 0.821 | 1.000 | 1617 | tags=19%, list=12%, signal=22% |
| 242 | RESPONSE\_TO\_NUTRIENT |  | 17 | -0.27 | -0.91 | 0.574 | 0.819 | 1.000 | 394 | tags=18%, list=3%, signal=18% |
| 243 | REGULATION\_OF\_TRANSCRIPTIONDNA\_DEPENDENT |  | 412 | -0.14 | -0.90 | 0.830 | 0.828 | 1.000 | 2973 | tags=26%, list=23%, signal=32% |
| 244 | REGULATION\_OF\_TRANSLATIONAL\_INITIATION |  | 25 | -0.24 | -0.90 | 0.614 | 0.842 | 1.000 | 2738 | tags=32%, list=21%, signal=40% |
| 245 | PHOSPHOINOSITIDE\_METABOLIC\_PROCESS |  | 25 | -0.24 | -0.89 | 0.612 | 0.841 | 1.000 | 3843 | tags=44%, list=29%, signal=62% |
| 246 | REGULATION\_OF\_GROWTH |  | 48 | -0.20 | -0.89 | 0.638 | 0.852 | 1.000 | 4588 | tags=46%, list=35%, signal=70% |
| 247 | AMINE\_METABOLIC\_PROCESS |  | 128 | -0.16 | -0.89 | 0.711 | 0.850 | 1.000 | 4156 | tags=38%, list=32%, signal=54% |
| 248 | PROTEIN\_HOMOOLIGOMERIZATION |  | 19 | -0.26 | -0.89 | 0.597 | 0.851 | 1.000 | 2084 | tags=26%, list=16%, signal=31% |
| 249 | ENDOSOME\_TRANSPORT |  | 22 | -0.24 | -0.88 | 0.623 | 0.861 | 1.000 | 2295 | tags=27%, list=18%, signal=33% |
| 250 | CELL\_CELL\_SIGNALING |  | 372 | -0.14 | -0.88 | 0.872 | 0.864 | 1.000 | 4097 | tags=32%, list=31%, signal=46% |
| 251 | REGULATION\_OF\_TRANSLATION |  | 76 | -0.17 | -0.87 | 0.747 | 0.885 | 1.000 | 2909 | tags=28%, list=22%, signal=35% |
| 252 | SECOND\_MESSENGER\_MEDIATED\_SIGNALING |  | 139 | -0.15 | -0.87 | 0.810 | 0.882 | 1.000 | 1323 | tags=12%, list=10%, signal=13% |
| 253 | SPHINGOLIPID\_METABOLIC\_PROCESS |  | 23 | -0.23 | -0.86 | 0.673 | 0.884 | 1.000 | 3247 | tags=35%, list=25%, signal=46% |
| 254 | ACTIVATION\_OF\_PROTEIN\_KINASE\_ACTIVITY |  | 23 | -0.23 | -0.86 | 0.636 | 0.896 | 1.000 | 4534 | tags=39%, list=35%, signal=60% |
| 255 | VESICLE\_MEDIATED\_TRANSPORT |  | 174 | -0.14 | -0.85 | 0.850 | 0.905 | 1.000 | 1935 | tags=18%, list=15%, signal=21% |
| 256 | POSITIVE\_REGULATION\_OF\_CELLULAR\_COMPONENT\_ORGANIZATION\_AND\_BIOGENESIS |  | 28 | -0.22 | -0.85 | 0.706 | 0.908 | 1.000 | 3474 | tags=36%, list=27%, signal=49% |
| 257 | REGULATION\_OF\_PROTEIN\_MODIFICATION\_PROCESS |  | 37 | -0.20 | -0.85 | 0.694 | 0.912 | 1.000 | 1577 | tags=19%, list=12%, signal=21% |
| 258 | CELLULAR\_MORPHOGENESIS\_DURING\_DIFFERENTIATION |  | 38 | -0.20 | -0.84 | 0.720 | 0.917 | 1.000 | 3461 | tags=29%, list=26%, signal=39% |
| 259 | REGULATION\_OF\_BINDING |  | 46 | -0.19 | -0.84 | 0.721 | 0.922 | 1.000 | 1842 | tags=22%, list=14%, signal=25% |
| 260 | CENTRAL\_NERVOUS\_SYSTEM\_DEVELOPMENT |  | 105 | -0.16 | -0.83 | 0.856 | 0.938 | 1.000 | 3825 | tags=32%, list=29%, signal=45% |
| 261 | EXTRACELLULAR\_STRUCTURE\_ORGANIZATION\_AND\_BIOGENESIS |  | 23 | -0.22 | -0.82 | 0.705 | 0.945 | 1.000 | 2061 | tags=26%, list=16%, signal=31% |
| 262 | POSITIVE\_REGULATION\_OF\_BINDING |  | 19 | -0.24 | -0.82 | 0.695 | 0.945 | 1.000 | 4419 | tags=53%, list=34%, signal=79% |
| 263 | NEGATIVE\_REGULATION\_OF\_GROWTH |  | 35 | -0.20 | -0.81 | 0.776 | 0.955 | 1.000 | 4588 | tags=49%, list=35%, signal=75% |
| 264 | RESPONSE\_TO\_NUTRIENT\_LEVELS |  | 27 | -0.21 | -0.81 | 0.752 | 0.951 | 1.000 | 4361 | tags=44%, list=33%, signal=67% |
| 265 | SULFUR\_METABOLIC\_PROCESS |  | 30 | -0.20 | -0.80 | 0.773 | 0.971 | 1.000 | 3352 | tags=33%, list=26%, signal=45% |
| 266 | EXCRETION |  | 35 | -0.19 | -0.80 | 0.792 | 0.970 | 1.000 | 2067 | tags=20%, list=16%, signal=24% |
| 267 | POSITIVE\_REGULATION\_OF\_JNK\_ACTIVITY |  | 16 | -0.23 | -0.79 | 0.754 | 0.988 | 1.000 | 2017 | tags=25%, list=15%, signal=30% |
| 268 | PATTERN\_SPECIFICATION\_PROCESS |  | 27 | -0.20 | -0.78 | 0.809 | 0.988 | 1.000 | 5641 | tags=63%, list=43%, signal=110% |
| 269 | POSITIVE\_REGULATION\_OF\_CASPASE\_ACTIVITY |  | 28 | -0.20 | -0.78 | 0.791 | 0.998 | 1.000 | 1954 | tags=25%, list=15%, signal=29% |
| 270 | G\_PROTEIN\_COUPLED\_RECEPTOR\_PROTEIN\_SIGNALING\_PATHWAY |  | 300 | -0.12 | -0.77 | 0.992 | 1.000 | 1.000 | 4024 | tags=28%, list=31%, signal=40% |
| 271 | T\_CELL\_PROLIFERATION |  | 17 | -0.23 | -0.77 | 0.778 | 0.997 | 1.000 | 4823 | tags=59%, list=37%, signal=93% |
| 272 | POSITIVE\_REGULATION\_OF\_TRANSCRIPTIONDNA\_DEPENDENT |  | 105 | -0.14 | -0.77 | 0.938 | 0.996 | 1.000 | 4567 | tags=40%, list=35%, signal=61% |
| 273 | REPRODUCTIVE\_PROCESS |  | 133 | -0.14 | -0.77 | 0.950 | 0.996 | 1.000 | 3831 | tags=32%, list=29%, signal=45% |
| 274 | REGULATION\_OF\_SECRETION |  | 35 | -0.19 | -0.77 | 0.829 | 0.992 | 1.000 | 4314 | tags=46%, list=33%, signal=68% |
| 275 | TRANSLATIONAL\_INITIATION |  | 33 | -0.19 | -0.76 | 0.840 | 0.998 | 1.000 | 1255 | tags=18%, list=10%, signal=20% |
| 276 | AMINE\_BIOSYNTHETIC\_PROCESS |  | 15 | -0.24 | -0.76 | 0.775 | 0.996 | 1.000 | 496 | tags=13%, list=4%, signal=14% |
| 277 | G\_PROTEIN\_SIGNALING\_COUPLED\_TO\_IP3\_SECOND\_MESSENGERPHOSPHOLIPASE\_C\_ACTIVATING |  | 39 | -0.18 | -0.76 | 0.880 | 0.994 | 1.000 | 3068 | tags=26%, list=23%, signal=33% |
| 278 | REGULATION\_OF\_CELL\_MIGRATION |  | 23 | -0.21 | -0.75 | 0.815 | 0.994 | 1.000 | 4847 | tags=43%, list=37%, signal=69% |
| 279 | GOLGI\_VESICLE\_TRANSPORT |  | 42 | -0.17 | -0.75 | 0.867 | 0.994 | 1.000 | 4449 | tags=43%, list=34%, signal=65% |
| 280 | REGULATION\_OF\_TRANSCRIPTION\_FACTOR\_ACTIVITY |  | 30 | -0.19 | -0.75 | 0.847 | 0.991 | 1.000 | 3966 | tags=40%, list=30%, signal=57% |
| 281 | ESTABLISHMENT\_AND\_OR\_MAINTENANCE\_OF\_CELL\_POLARITY |  | 19 | -0.21 | -0.75 | 0.836 | 0.992 | 1.000 | 3686 | tags=32%, list=28%, signal=44% |
| 282 | REGULATION\_OF\_DNA\_BINDING |  | 36 | -0.18 | -0.74 | 0.862 | 0.993 | 1.000 | 3036 | tags=31%, list=23%, signal=40% |
| 283 | METAL\_ION\_TRANSPORT |  | 102 | -0.14 | -0.73 | 0.952 | 1.000 | 1.000 | 5365 | tags=48%, list=41%, signal=81% |
| 284 | RESPONSE\_TO\_EXTRACELLULAR\_STIMULUS |  | 29 | -0.18 | -0.73 | 0.896 | 1.000 | 1.000 | 2466 | tags=24%, list=19%, signal=30% |
| 285 | DI\_\_\_TRI\_VALENT\_INORGANIC\_CATION\_TRANSPORT |  | 27 | -0.18 | -0.73 | 0.882 | 1.000 | 1.000 | 1108 | tags=15%, list=8%, signal=16% |
| 286 | POSITIVE\_REGULATION\_OF\_RNA\_METABOLIC\_PROCESS |  | 107 | -0.14 | -0.73 | 0.965 | 0.999 | 1.000 | 3256 | tags=26%, list=25%, signal=35% |
| 287 | CARBOHYDRATE\_METABOLIC\_PROCESS |  | 152 | -0.13 | -0.73 | 0.984 | 0.996 | 1.000 | 3864 | tags=31%, list=30%, signal=43% |
| 288 | LIPID\_HOMEOSTASIS |  | 15 | -0.22 | -0.72 | 0.827 | 1.000 | 1.000 | 594 | tags=13%, list=5%, signal=14% |
| 289 | SECRETION |  | 157 | -0.12 | -0.71 | 0.986 | 1.000 | 1.000 | 4314 | tags=36%, list=33%, signal=53% |
| 290 | PROTEIN\_POLYMERIZATION |  | 17 | -0.21 | -0.71 | 0.860 | 1.000 | 1.000 | 243 | tags=12%, list=2%, signal=12% |
| 291 | PROTEIN\_AMINO\_ACID\_LIPIDATION |  | 21 | -0.19 | -0.70 | 0.877 | 1.000 | 1.000 | 4622 | tags=52%, list=35%, signal=81% |
| 292 | G\_PROTEIN\_SIGNALING\_ADENYLATE\_CYCLASE\_ACTIVATING\_PATHWAY |  | 24 | -0.18 | -0.70 | 0.879 | 1.000 | 1.000 | 1300 | tags=13%, list=10%, signal=14% |
| 293 | NUCLEOTIDE\_EXCISION\_REPAIR |  | 19 | -0.20 | -0.69 | 0.908 | 1.000 | 1.000 | 2011 | tags=21%, list=15%, signal=25% |
| 294 | SECRETORY\_PATHWAY |  | 72 | -0.14 | -0.69 | 0.969 | 1.000 | 1.000 | 4449 | tags=40%, list=34%, signal=61% |
| 295 | INORGANIC\_ANION\_TRANSPORT |  | 16 | -0.21 | -0.68 | 0.874 | 1.000 | 1.000 | 601 | tags=13%, list=5%, signal=13% |
| 296 | POTASSIUM\_ION\_TRANSPORT |  | 52 | -0.15 | -0.68 | 0.953 | 1.000 | 1.000 | 5434 | tags=50%, list=42%, signal=85% |
| 297 | CARBOHYDRATE\_BIOSYNTHETIC\_PROCESS |  | 35 | -0.16 | -0.67 | 0.920 | 1.000 | 1.000 | 5307 | tags=54%, list=41%, signal=91% |
| 298 | NEGATIVE\_REGULATION\_OF\_CELLULAR\_PROTEIN\_METABOLIC\_PROCESS |  | 41 | -0.15 | -0.67 | 0.957 | 1.000 | 1.000 | 2952 | tags=24%, list=23%, signal=31% |
| 299 | CATION\_TRANSPORT |  | 130 | -0.12 | -0.66 | 0.995 | 1.000 | 1.000 | 3744 | tags=28%, list=29%, signal=38% |
| 300 | PHOSPHOINOSITIDE\_MEDIATED\_SIGNALING |  | 42 | -0.15 | -0.66 | 0.949 | 1.000 | 1.000 | 2691 | tags=21%, list=21%, signal=27% |
| 301 | ION\_TRANSPORT |  | 165 | -0.11 | -0.65 | 1.000 | 1.000 | 1.000 | 4113 | tags=30%, list=31%, signal=44% |
| 302 | RHO\_PROTEIN\_SIGNAL\_TRANSDUCTION |  | 30 | -0.16 | -0.65 | 0.932 | 1.000 | 1.000 | 3701 | tags=33%, list=28%, signal=46% |
| 303 | REGULATION\_OF\_CELL\_GROWTH |  | 39 | -0.15 | -0.64 | 0.978 | 1.000 | 1.000 | 4588 | tags=41%, list=35%, signal=63% |
| 304 | REGULATION\_OF\_CYTOKINE\_PRODUCTION |  | 21 | -0.18 | -0.64 | 0.926 | 1.000 | 1.000 | 2454 | tags=24%, list=19%, signal=29% |
| 305 | NEGATIVE\_REGULATION\_OF\_MULTICELLULAR\_ORGANISMAL\_PROCESS |  | 27 | -0.17 | -0.64 | 0.931 | 1.000 | 1.000 | 2250 | tags=22%, list=17%, signal=27% |
| 306 | NEGATIVE\_REGULATION\_OF\_PROTEIN\_METABOLIC\_PROCESS |  | 44 | -0.14 | -0.62 | 0.975 | 1.000 | 1.000 | 2952 | tags=23%, list=23%, signal=29% |
| 307 | POSITIVE\_REGULATION\_OF\_TRANSPORT |  | 18 | -0.17 | -0.60 | 0.955 | 1.000 | 1.000 | 5157 | tags=56%, list=39%, signal=92% |
| 308 | CALCIUM\_ION\_TRANSPORT |  | 23 | -0.16 | -0.58 | 0.958 | 1.000 | 1.000 | 1108 | tags=13%, list=8%, signal=14% |
| 309 | CELLULAR\_CARBOHYDRATE\_CATABOLIC\_PROCESS |  | 20 | -0.16 | -0.58 | 0.961 | 1.000 | 1.000 | 4406 | tags=35%, list=34%, signal=53% |
| 310 | CARBOHYDRATE\_CATABOLIC\_PROCESS |  | 20 | -0.16 | -0.58 | 0.973 | 1.000 | 1.000 | 4406 | tags=35%, list=34%, signal=53% |
| 311 | FEMALE\_GAMETE\_GENERATION |  | 15 | -0.18 | -0.57 | 0.970 | 1.000 | 1.000 | 10767 | tags=100%, list=82%, signal=563% |
| 312 | REGULATION\_OF\_HEART\_CONTRACTION |  | 24 | -0.15 | -0.57 | 0.986 | 1.000 | 1.000 | 11088 | tags=100%, list=85%, signal=652% |
| 313 | PEROXISOME\_ORGANIZATION\_AND\_BIOGENESIS |  | 15 | -0.17 | -0.54 | 0.974 | 1.000 | 1.000 | 4814 | tags=47%, list=37%, signal=74% |
| 314 | AMINO\_SUGAR\_METABOLIC\_PROCESS |  | 15 | -0.16 | -0.54 | 0.987 | 1.000 | 1.000 | 3700 | tags=33%, list=28%, signal=46% |
| 315 | REGULATION\_OF\_ACTION\_POTENTIAL |  | 16 | -0.16 | -0.53 | 0.986 | 1.000 | 1.000 | 3589 | tags=31%, list=27%, signal=43% |
| 316 | RESPONSE\_TO\_LIGHT\_STIMULUS |  | 40 | -0.12 | -0.52 | 0.993 | 0.998 | 1.000 | 2677 | tags=20%, list=20%, signal=25% |
| 317 | MONOVALENT\_INORGANIC\_CATION\_TRANSPORT |  | 83 | -0.10 | -0.48 | 1.000 | 0.998 | 1.000 | 5434 | tags=46%, list=42%, signal=78% |
Table: Gene sets enriched in phenotype **na**[plain text format]****

  
