# Supplementary material for: Multiple-input multiple-output causal strategies for gene selection
Source: BMC Bioinformatics. 2011 Nov 25;12:458. doi: 10.1186/1471-2105-12-458 (PMC3323860; doi:10.1186/1471-2105-12-458)
Supplement: Additional file 2 — Archive containing the output files computed by the preranked GSEA for λ ∈ {0.1,0.2,0.3,0.4,0.5} (GSEA_MIMO_part1.zip). [file 1471-2105-12-458-S2.ZIP › mFS02_entrez_mimo.GseaPreranked.1316038034143/gsea_report_for_na_pos_1316038034143.html]

Report for na\_pos 1316038034143 [GSEA]

| GS  follow link to MSigDB | GS DETAILS | SIZE | ES | NES | NOM p-val | FDR q-val | FWER p-val | RANK AT MAX | LEADING EDGE || 1 | M\_PHASE\_OF\_MITOTIC\_CELL\_CYCLE |  | 72 | 0.60 | 2.80 | 0.000 | 0.000 | 0.000 | 2095 | tags=56%, list=16%, signal=66% |
| 2 | MITOSIS |  | 70 | 0.59 | 2.78 | 0.000 | 0.000 | 0.000 | 2095 | tags=54%, list=16%, signal=64% |
| 3 | M\_PHASE |  | 98 | 0.55 | 2.78 | 0.000 | 0.000 | 0.000 | 2151 | tags=51%, list=16%, signal=61% |
| 4 | MITOTIC\_CELL\_CYCLE |  | 134 | 0.51 | 2.75 | 0.000 | 0.000 | 0.000 | 2724 | tags=52%, list=21%, signal=65% |
| 5 | CELL\_CYCLE\_PROCESS |  | 169 | 0.50 | 2.74 | 0.000 | 0.000 | 0.000 | 2636 | tags=50%, list=20%, signal=61% |
| 6 | CELL\_CYCLE\_PHASE |  | 152 | 0.48 | 2.61 | 0.000 | 0.000 | 0.000 | 2636 | tags=48%, list=20%, signal=59% |
| 7 | DNA\_REPLICATION |  | 97 | 0.50 | 2.54 | 0.000 | 0.000 | 0.000 | 2679 | tags=47%, list=20%, signal=59% |
| 8 | SISTER\_CHROMATID\_SEGREGATION |  | 16 | 0.76 | 2.47 | 0.000 | 0.000 | 0.001 | 627 | tags=56%, list=5%, signal=59% |
| 9 | MITOTIC\_SISTER\_CHROMATID\_SEGREGATION |  | 15 | 0.78 | 2.47 | 0.000 | 0.000 | 0.001 | 627 | tags=60%, list=5%, signal=63% |
| 10 | DNA\_METABOLIC\_PROCESS |  | 240 | 0.42 | 2.46 | 0.000 | 0.000 | 0.001 | 3308 | tags=49%, list=25%, signal=64% |
| 11 | CELL\_CYCLE\_GO\_0007049 |  | 277 | 0.41 | 2.42 | 0.000 | 0.000 | 0.001 | 2636 | tags=43%, list=20%, signal=52% |
| 12 | CHROMOSOME\_SEGREGATION |  | 28 | 0.62 | 2.41 | 0.000 | 0.000 | 0.001 | 627 | tags=46%, list=5%, signal=49% |
| 13 | CELL\_CYCLE\_CHECKPOINT\_GO\_0000075 |  | 45 | 0.54 | 2.37 | 0.000 | 0.000 | 0.001 | 2207 | tags=56%, list=17%, signal=67% |
| 14 | REGULATION\_OF\_MITOSIS |  | 33 | 0.59 | 2.32 | 0.000 | 0.000 | 0.001 | 1847 | tags=52%, list=14%, signal=60% |
| 15 | DNA\_REPAIR |  | 118 | 0.44 | 2.32 | 0.000 | 0.000 | 0.001 | 2585 | tags=46%, list=20%, signal=57% |
| 16 | DNA\_DEPENDENT\_DNA\_REPLICATION |  | 52 | 0.51 | 2.31 | 0.000 | 0.000 | 0.001 | 2862 | tags=54%, list=22%, signal=69% |
| 17 | RESPONSE\_TO\_DNA\_DAMAGE\_STIMULUS |  | 153 | 0.42 | 2.29 | 0.000 | 0.000 | 0.003 | 2585 | tags=44%, list=20%, signal=54% |
| 18 | RNA\_SPLICING |  | 74 | 0.47 | 2.26 | 0.000 | 0.000 | 0.005 | 3550 | tags=55%, list=27%, signal=76% |
| 19 | RESPONSE\_TO\_ENDOGENOUS\_STIMULUS |  | 182 | 0.38 | 2.15 | 0.000 | 0.001 | 0.022 | 3298 | tags=46%, list=25%, signal=60% |
| 20 | RNA\_PROCESSING |  | 138 | 0.40 | 2.15 | 0.000 | 0.001 | 0.025 | 3550 | tags=52%, list=27%, signal=71% |
| 21 | NUCLEOTIDE\_BIOSYNTHETIC\_PROCESS |  | 17 | 0.65 | 2.14 | 0.000 | 0.001 | 0.025 | 1522 | tags=53%, list=12%, signal=60% |
| 22 | MITOTIC\_CELL\_CYCLE\_CHECKPOINT |  | 19 | 0.60 | 2.09 | 0.002 | 0.002 | 0.043 | 1847 | tags=53%, list=14%, signal=61% |
| 23 | MICROTUBULE\_CYTOSKELETON\_ORGANIZATION\_AND\_BIOGENESIS |  | 31 | 0.53 | 2.06 | 0.000 | 0.002 | 0.060 | 2724 | tags=55%, list=21%, signal=69% |
| 24 | DNA\_INTEGRITY\_CHECKPOINT |  | 22 | 0.58 | 2.06 | 0.000 | 0.002 | 0.060 | 2207 | tags=59%, list=17%, signal=71% |
| 25 | MRNA\_METABOLIC\_PROCESS |  | 72 | 0.43 | 2.05 | 0.000 | 0.002 | 0.077 | 2983 | tags=49%, list=23%, signal=63% |
| 26 | REGULATION\_OF\_CELL\_CYCLE |  | 161 | 0.37 | 2.04 | 0.000 | 0.003 | 0.088 | 1847 | tags=35%, list=14%, signal=40% |
| 27 | MITOCHONDRION\_ORGANIZATION\_AND\_BIOGENESIS |  | 42 | 0.48 | 2.03 | 0.000 | 0.003 | 0.099 | 3267 | tags=52%, list=25%, signal=70% |
| 28 | DNA\_REPLICATION\_INITIATION |  | 15 | 0.64 | 2.03 | 0.000 | 0.003 | 0.101 | 2862 | tags=80%, list=22%, signal=102% |
| 29 | PROTEIN\_FOLDING |  | 55 | 0.44 | 1.99 | 0.000 | 0.004 | 0.141 | 3265 | tags=51%, list=25%, signal=68% |
| 30 | NUCLEOBASENUCLEOSIDENUCLEOTIDE\_AND\_NUCLEIC\_ACID\_TRANSPORT |  | 26 | 0.52 | 1.98 | 0.002 | 0.004 | 0.158 | 2394 | tags=50%, list=18%, signal=61% |
| 31 | REGULATION\_OF\_MITOTIC\_CELL\_CYCLE |  | 19 | 0.57 | 1.97 | 0.002 | 0.005 | 0.179 | 1170 | tags=47%, list=9%, signal=52% |
| 32 | DOUBLE\_STRAND\_BREAK\_REPAIR |  | 21 | 0.56 | 1.96 | 0.004 | 0.005 | 0.196 | 1987 | tags=52%, list=15%, signal=62% |
| 33 | TRNA\_METABOLIC\_PROCESS |  | 15 | 0.61 | 1.94 | 0.004 | 0.006 | 0.229 | 2854 | tags=67%, list=22%, signal=85% |
| 34 | COENZYME\_METABOLIC\_PROCESS |  | 35 | 0.48 | 1.94 | 0.002 | 0.006 | 0.233 | 3467 | tags=49%, list=26%, signal=66% |
| 35 | MRNA\_PROCESSING\_GO\_0006397 |  | 61 | 0.42 | 1.93 | 0.002 | 0.006 | 0.238 | 3519 | tags=51%, list=27%, signal=69% |
| 36 | INTERPHASE\_OF\_MITOTIC\_CELL\_CYCLE |  | 57 | 0.42 | 1.90 | 0.000 | 0.009 | 0.338 | 3630 | tags=53%, list=28%, signal=73% |
| 37 | INTERPHASE |  | 63 | 0.41 | 1.89 | 0.000 | 0.009 | 0.370 | 3277 | tags=48%, list=25%, signal=63% |
| 38 | G1\_S\_TRANSITION\_OF\_MITOTIC\_CELL\_CYCLE |  | 23 | 0.51 | 1.89 | 0.000 | 0.009 | 0.381 | 2460 | tags=48%, list=19%, signal=59% |
| 39 | REGULATION\_OF\_CYCLIN\_DEPENDENT\_PROTEIN\_KINASE\_ACTIVITY |  | 40 | 0.45 | 1.88 | 0.002 | 0.010 | 0.389 | 2918 | tags=52%, list=22%, signal=67% |
| 40 | CHROMOSOME\_ORGANIZATION\_AND\_BIOGENESIS |  | 107 | 0.37 | 1.88 | 0.000 | 0.009 | 0.392 | 2546 | tags=37%, list=19%, signal=46% |
| 41 | COFACTOR\_BIOSYNTHETIC\_PROCESS |  | 21 | 0.53 | 1.88 | 0.002 | 0.009 | 0.396 | 1513 | tags=38%, list=12%, signal=43% |
| 42 | REGULATION\_OF\_DNA\_METABOLIC\_PROCESS |  | 40 | 0.44 | 1.85 | 0.000 | 0.011 | 0.471 | 2207 | tags=45%, list=17%, signal=54% |
| 43 | REGULATION\_OF\_DNA\_REPLICATION |  | 18 | 0.54 | 1.82 | 0.009 | 0.015 | 0.573 | 2207 | tags=50%, list=17%, signal=60% |
| 44 | MITOCHONDRIAL\_TRANSPORT |  | 18 | 0.54 | 1.80 | 0.004 | 0.016 | 0.613 | 1354 | tags=44%, list=10%, signal=50% |
| 45 | BIOPOLYMER\_CATABOLIC\_PROCESS |  | 103 | 0.35 | 1.79 | 0.000 | 0.018 | 0.648 | 2634 | tags=37%, list=20%, signal=46% |
| 46 | DNA\_DAMAGE\_RESPONSESIGNAL\_TRANSDUCTION |  | 34 | 0.46 | 1.79 | 0.002 | 0.018 | 0.655 | 2269 | tags=47%, list=17%, signal=57% |
| 47 | DNA\_DAMAGE\_CHECKPOINT |  | 19 | 0.53 | 1.78 | 0.004 | 0.018 | 0.669 | 2207 | tags=53%, list=17%, signal=63% |
| 48 | DNA\_RECOMBINATION |  | 45 | 0.41 | 1.77 | 0.005 | 0.020 | 0.713 | 1325 | tags=33%, list=10%, signal=37% |
| 49 | UBIQUITIN\_CYCLE |  | 40 | 0.42 | 1.75 | 0.005 | 0.022 | 0.751 | 2184 | tags=38%, list=17%, signal=45% |
| 50 | PROTEIN\_MODIFICATION\_BY\_SMALL\_PROTEIN\_CONJUGATION |  | 35 | 0.43 | 1.75 | 0.002 | 0.022 | 0.757 | 2184 | tags=40%, list=17%, signal=48% |
| 51 | COFACTOR\_METABOLIC\_PROCESS |  | 51 | 0.39 | 1.74 | 0.004 | 0.024 | 0.790 | 3535 | tags=43%, list=27%, signal=59% |
| 52 | PROTEIN\_CATABOLIC\_PROCESS |  | 60 | 0.38 | 1.72 | 0.006 | 0.028 | 0.852 | 2634 | tags=35%, list=20%, signal=44% |
| 53 | MACROMOLECULE\_CATABOLIC\_PROCESS |  | 120 | 0.32 | 1.71 | 0.003 | 0.028 | 0.861 | 2634 | tags=33%, list=20%, signal=41% |
| 54 | DNA\_PACKAGING |  | 29 | 0.45 | 1.71 | 0.013 | 0.028 | 0.862 | 2529 | tags=45%, list=19%, signal=55% |
| 55 | NUCLEAR\_EXPORT |  | 26 | 0.46 | 1.71 | 0.007 | 0.028 | 0.874 | 2394 | tags=42%, list=18%, signal=52% |
| 56 | TRANSCRIPTION\_INITIATION\_FROM\_RNA\_POLYMERASE\_II\_PROMOTER |  | 27 | 0.46 | 1.71 | 0.013 | 0.028 | 0.876 | 2737 | tags=44%, list=21%, signal=56% |
| 57 | MEIOSIS\_I |  | 19 | 0.50 | 1.70 | 0.004 | 0.029 | 0.889 | 1325 | tags=37%, list=10%, signal=41% |
| 58 | CELLULAR\_PROTEIN\_CATABOLIC\_PROCESS |  | 50 | 0.39 | 1.69 | 0.011 | 0.031 | 0.908 | 2419 | tags=34%, list=18%, signal=42% |
| 59 | PROTEIN\_UBIQUITINATION |  | 32 | 0.42 | 1.68 | 0.012 | 0.034 | 0.929 | 2184 | tags=38%, list=17%, signal=45% |
| 60 | BASE\_EXCISION\_REPAIR |  | 16 | 0.50 | 1.66 | 0.011 | 0.039 | 0.955 | 2471 | tags=44%, list=19%, signal=54% |
| 61 | RNA\_EXPORT\_FROM\_NUCLEUS |  | 17 | 0.49 | 1.63 | 0.027 | 0.048 | 0.981 | 2394 | tags=47%, list=18%, signal=58% |
| 62 | ONE\_CARBON\_COMPOUND\_METABOLIC\_PROCESS |  | 24 | 0.45 | 1.62 | 0.022 | 0.050 | 0.985 | 2837 | tags=50%, list=22%, signal=64% |
| 63 | MEIOTIC\_CELL\_CYCLE |  | 31 | 0.42 | 1.61 | 0.013 | 0.052 | 0.987 | 2724 | tags=42%, list=21%, signal=53% |
| 64 | PROTEIN\_DNA\_COMPLEX\_ASSEMBLY |  | 45 | 0.37 | 1.61 | 0.010 | 0.053 | 0.990 | 2737 | tags=40%, list=21%, signal=50% |
| 65 | CYTOKINESIS |  | 17 | 0.48 | 1.60 | 0.029 | 0.054 | 0.990 | 1086 | tags=35%, list=8%, signal=38% |
| 66 | NUCLEOTIDE\_METABOLIC\_PROCESS |  | 36 | 0.39 | 1.59 | 0.033 | 0.060 | 0.997 | 768 | tags=28%, list=6%, signal=29% |
| 67 | NUCLEOBASENUCLEOSIDE\_AND\_NUCLEOTIDE\_METABOLIC\_PROCESS |  | 46 | 0.37 | 1.58 | 0.006 | 0.061 | 0.998 | 768 | tags=26%, list=6%, signal=28% |
| 68 | APOPTOTIC\_NUCLEAR\_CHANGES |  | 17 | 0.47 | 1.58 | 0.039 | 0.063 | 0.998 | 2269 | tags=47%, list=17%, signal=57% |
| 69 | CHROMATIN\_ASSEMBLY\_OR\_DISASSEMBLY |  | 25 | 0.42 | 1.57 | 0.020 | 0.062 | 0.998 | 2529 | tags=48%, list=19%, signal=59% |
| 70 | CELLULAR\_MACROMOLECULE\_CATABOLIC\_PROCESS |  | 90 | 0.31 | 1.56 | 0.010 | 0.069 | 0.999 | 2569 | tags=31%, list=20%, signal=38% |
| 71 | CELLULAR\_COMPONENT\_DISASSEMBLY |  | 31 | 0.40 | 1.55 | 0.029 | 0.070 | 0.999 | 2269 | tags=39%, list=17%, signal=47% |
| 72 | NUCLEAR\_TRANSPORT |  | 77 | 0.32 | 1.55 | 0.009 | 0.072 | 0.999 | 2710 | tags=34%, list=21%, signal=42% |
| 73 | NUCLEOCYTOPLASMIC\_TRANSPORT |  | 77 | 0.32 | 1.52 | 0.017 | 0.083 | 1.000 | 2710 | tags=34%, list=21%, signal=42% |
| 74 | ORGANELLE\_ORGANIZATION\_AND\_BIOGENESIS |  | 407 | 0.24 | 1.52 | 0.000 | 0.083 | 1.000 | 3292 | tags=34%, list=25%, signal=44% |
| 75 | MICROTUBULE\_BASED\_PROCESS |  | 75 | 0.32 | 1.49 | 0.016 | 0.099 | 1.000 | 2944 | tags=37%, list=22%, signal=48% |
| 76 | TRANSCRIPTION\_INITIATION |  | 33 | 0.38 | 1.48 | 0.047 | 0.106 | 1.000 | 2310 | tags=36%, list=18%, signal=44% |
| 77 | CELL\_DIVISION |  | 19 | 0.44 | 1.47 | 0.063 | 0.112 | 1.000 | 1086 | tags=32%, list=8%, signal=34% |
| 78 | ESTABLISHMENT\_OF\_ORGANELLE\_LOCALIZATION |  | 16 | 0.46 | 1.46 | 0.064 | 0.119 | 1.000 | 950 | tags=38%, list=7%, signal=40% |
| 79 | VIRAL\_INFECTIOUS\_CYCLE |  | 29 | 0.38 | 1.46 | 0.054 | 0.119 | 1.000 | 997 | tags=31%, list=8%, signal=34% |
| 80 | NEGATIVE\_REGULATION\_OF\_DNA\_METABOLIC\_PROCESS |  | 16 | 0.44 | 1.43 | 0.080 | 0.138 | 1.000 | 2546 | tags=50%, list=19%, signal=62% |
| 81 | ORGANELLE\_LOCALIZATION |  | 21 | 0.40 | 1.43 | 0.081 | 0.137 | 1.000 | 950 | tags=29%, list=7%, signal=31% |
| 82 | APOPTOTIC\_PROGRAM |  | 56 | 0.31 | 1.40 | 0.043 | 0.162 | 1.000 | 3630 | tags=46%, list=28%, signal=64% |
| 83 | CHROMATIN\_REMODELING |  | 21 | 0.39 | 1.39 | 0.081 | 0.168 | 1.000 | 2405 | tags=43%, list=18%, signal=52% |
| 84 | NEGATIVE\_REGULATION\_OF\_BINDING |  | 16 | 0.42 | 1.39 | 0.102 | 0.177 | 1.000 | 2611 | tags=50%, list=20%, signal=62% |
| 85 | VIRAL\_REPRODUCTIVE\_PROCESS |  | 33 | 0.35 | 1.38 | 0.094 | 0.184 | 1.000 | 1156 | tags=30%, list=9%, signal=33% |
| 86 | MEIOTIC\_RECOMBINATION |  | 16 | 0.43 | 1.37 | 0.115 | 0.192 | 1.000 | 1325 | tags=31%, list=10%, signal=35% |
| 87 | ESTABLISHMENT\_AND\_OR\_MAINTENANCE\_OF\_CHROMATIN\_ARCHITECTURE |  | 65 | 0.29 | 1.37 | 0.064 | 0.193 | 1.000 | 2529 | tags=34%, list=19%, signal=42% |
| 88 | ALCOHOL\_METABOLIC\_PROCESS |  | 82 | 0.28 | 1.37 | 0.062 | 0.191 | 1.000 | 3966 | tags=39%, list=30%, signal=56% |
| 89 | G1\_PHASE |  | 15 | 0.42 | 1.36 | 0.109 | 0.189 | 1.000 | 413 | tags=27%, list=3%, signal=28% |
| 90 | REGULATION\_OF\_GENE\_EXPRESSION\_EPIGENETIC |  | 27 | 0.35 | 1.35 | 0.093 | 0.208 | 1.000 | 2795 | tags=41%, list=21%, signal=52% |
| 91 | INTRACELLULAR\_TRANSPORT |  | 248 | 0.23 | 1.33 | 0.024 | 0.227 | 1.000 | 3298 | tags=33%, list=25%, signal=43% |
| 92 | RESPONSE\_TO\_ORGANIC\_SUBSTANCE |  | 27 | 0.35 | 1.31 | 0.130 | 0.249 | 1.000 | 2874 | tags=37%, list=22%, signal=47% |
| 93 | RESPONSE\_TO\_HYPOXIA |  | 27 | 0.35 | 1.31 | 0.133 | 0.247 | 1.000 | 2302 | tags=33%, list=18%, signal=40% |
| 94 | NEGATIVE\_REGULATION\_OF\_CATALYTIC\_ACTIVITY |  | 61 | 0.28 | 1.30 | 0.102 | 0.260 | 1.000 | 2826 | tags=36%, list=22%, signal=46% |
| 95 | RESPONSE\_TO\_ABIOTIC\_STIMULUS |  | 79 | 0.27 | 1.30 | 0.084 | 0.260 | 1.000 | 3467 | tags=37%, list=26%, signal=50% |
| 96 | NUCLEAR\_ORGANIZATION\_AND\_BIOGENESIS |  | 23 | 0.36 | 1.30 | 0.140 | 0.259 | 1.000 | 2269 | tags=39%, list=17%, signal=47% |
| 97 | VIRAL\_REPRODUCTION |  | 38 | 0.31 | 1.30 | 0.124 | 0.259 | 1.000 | 1156 | tags=26%, list=9%, signal=29% |
| 98 | ESTABLISHMENT\_OF\_CELLULAR\_LOCALIZATION |  | 311 | 0.21 | 1.29 | 0.044 | 0.271 | 1.000 | 3298 | tags=31%, list=25%, signal=40% |
| 99 | DNA\_CATABOLIC\_PROCESS |  | 21 | 0.37 | 1.28 | 0.150 | 0.280 | 1.000 | 3833 | tags=52%, list=29%, signal=74% |
| 100 | RESPONSE\_TO\_STRESS |  | 467 | 0.20 | 1.28 | 0.024 | 0.278 | 1.000 | 3064 | tags=30%, list=23%, signal=37% |
| 101 | VIRAL\_GENOME\_REPLICATION |  | 20 | 0.37 | 1.27 | 0.132 | 0.282 | 1.000 | 997 | tags=30%, list=8%, signal=32% |
| 102 | RNA\_CATABOLIC\_PROCESS |  | 20 | 0.37 | 1.27 | 0.166 | 0.280 | 1.000 | 2394 | tags=45%, list=18%, signal=55% |
| 103 | CELLULAR\_LOCALIZATION |  | 323 | 0.21 | 1.26 | 0.052 | 0.300 | 1.000 | 3298 | tags=30%, list=25%, signal=40% |
| 104 | CHROMATIN\_ASSEMBLY |  | 16 | 0.39 | 1.26 | 0.190 | 0.300 | 1.000 | 2529 | tags=44%, list=19%, signal=54% |
| 105 | HETEROCYCLE\_METABOLIC\_PROCESS |  | 26 | 0.34 | 1.25 | 0.168 | 0.307 | 1.000 | 1513 | tags=23%, list=12%, signal=26% |
| 106 | CYTOSKELETON\_DEPENDENT\_INTRACELLULAR\_TRANSPORT |  | 25 | 0.34 | 1.25 | 0.173 | 0.312 | 1.000 | 3952 | tags=52%, list=30%, signal=74% |
| 107 | OXYGEN\_AND\_REACTIVE\_OXYGEN\_SPECIES\_METABOLIC\_PROCESS |  | 18 | 0.37 | 1.24 | 0.190 | 0.316 | 1.000 | 2810 | tags=44%, list=21%, signal=57% |
| 108 | RIBONUCLEOPROTEIN\_COMPLEX\_BIOGENESIS\_AND\_ASSEMBLY |  | 68 | 0.26 | 1.23 | 0.140 | 0.330 | 1.000 | 3787 | tags=41%, list=29%, signal=58% |
| 109 | CELL\_STRUCTURE\_DISASSEMBLY\_DURING\_APOPTOSIS |  | 17 | 0.37 | 1.23 | 0.214 | 0.332 | 1.000 | 2269 | tags=35%, list=17%, signal=43% |
| 110 | NEGATIVE\_REGULATION\_OF\_DNA\_BINDING |  | 15 | 0.39 | 1.23 | 0.216 | 0.332 | 1.000 | 2611 | tags=47%, list=20%, signal=58% |
| 111 | TRANSCRIPTION\_FROM\_RNA\_POLYMERASE\_II\_PROMOTER |  | 428 | 0.19 | 1.19 | 0.069 | 0.403 | 1.000 | 2759 | tags=26%, list=21%, signal=31% |
| 112 | CHROMATIN\_MODIFICATION |  | 46 | 0.27 | 1.17 | 0.218 | 0.444 | 1.000 | 2985 | tags=35%, list=23%, signal=45% |
| 113 | CELLULAR\_RESPIRATION |  | 19 | 0.34 | 1.17 | 0.256 | 0.442 | 1.000 | 2664 | tags=37%, list=20%, signal=46% |
| 114 | TRANSCRIPTION\_FROM\_RNA\_POLYMERASE\_III\_PROMOTER |  | 18 | 0.35 | 1.16 | 0.282 | 0.458 | 1.000 | 3884 | tags=56%, list=30%, signal=79% |
| 115 | CATABOLIC\_PROCESS |  | 201 | 0.21 | 1.16 | 0.164 | 0.454 | 1.000 | 2707 | tags=26%, list=21%, signal=32% |
| 116 | NITROGEN\_COMPOUND\_BIOSYNTHETIC\_PROCESS |  | 25 | 0.31 | 1.15 | 0.262 | 0.454 | 1.000 | 2302 | tags=28%, list=18%, signal=34% |
| 117 | REGULATION\_OF\_KINASE\_ACTIVITY |  | 135 | 0.22 | 1.15 | 0.198 | 0.451 | 1.000 | 2073 | tags=23%, list=16%, signal=27% |
| 118 | CELLULAR\_RESPONSE\_TO\_STIMULUS |  | 17 | 0.34 | 1.15 | 0.275 | 0.462 | 1.000 | 4226 | tags=53%, list=32%, signal=78% |
| 119 | REGULATION\_OF\_CATALYTIC\_ACTIVITY |  | 238 | 0.20 | 1.15 | 0.160 | 0.461 | 1.000 | 3086 | tags=29%, list=24%, signal=37% |
| 120 | REGULATION\_OF\_HYDROLASE\_ACTIVITY |  | 65 | 0.24 | 1.14 | 0.238 | 0.472 | 1.000 | 3079 | tags=34%, list=24%, signal=44% |
| 121 | GLUTAMATE\_SIGNALING\_PATHWAY |  | 17 | 0.34 | 1.14 | 0.279 | 0.474 | 1.000 | 3147 | tags=29%, list=24%, signal=39% |
| 122 | REGULATION\_OF\_TRANSFERASE\_ACTIVITY |  | 137 | 0.21 | 1.14 | 0.209 | 0.474 | 1.000 | 2073 | tags=23%, list=16%, signal=27% |
| 123 | CELLULAR\_CATABOLIC\_PROCESS |  | 189 | 0.20 | 1.13 | 0.193 | 0.478 | 1.000 | 2707 | tags=25%, list=21%, signal=32% |
| 124 | INDUCTION\_OF\_APOPTOSIS\_BY\_EXTRACELLULAR\_SIGNALS |  | 25 | 0.31 | 1.13 | 0.301 | 0.474 | 1.000 | 2658 | tags=36%, list=20%, signal=45% |
| 125 | RNA\_SPLICINGVIA\_TRANSESTERIFICATION\_REACTIONS |  | 27 | 0.30 | 1.12 | 0.289 | 0.503 | 1.000 | 3787 | tags=41%, list=29%, signal=57% |
| 126 | REGULATION\_OF\_PROTEIN\_KINASE\_ACTIVITY |  | 133 | 0.21 | 1.11 | 0.245 | 0.506 | 1.000 | 2541 | tags=26%, list=19%, signal=31% |
| 127 | NEGATIVE\_REGULATION\_OF\_TRANSPORT |  | 18 | 0.33 | 1.11 | 0.311 | 0.514 | 1.000 | 3479 | tags=44%, list=27%, signal=60% |
| 128 | NUCLEAR\_IMPORT |  | 47 | 0.25 | 1.11 | 0.277 | 0.516 | 1.000 | 3298 | tags=34%, list=25%, signal=45% |
| 129 | NEURON\_APOPTOSIS |  | 15 | 0.34 | 1.10 | 0.313 | 0.529 | 1.000 | 1242 | tags=27%, list=9%, signal=29% |
| 130 | INTERACTION\_WITH\_HOST |  | 15 | 0.34 | 1.10 | 0.332 | 0.537 | 1.000 | 1156 | tags=27%, list=9%, signal=29% |
| 131 | REGULATION\_OF\_MOLECULAR\_FUNCTION |  | 275 | 0.18 | 1.08 | 0.256 | 0.579 | 1.000 | 3086 | tags=28%, list=24%, signal=35% |
| 132 | DNA\_DAMAGE\_RESPONSESIGNAL\_TRANSDUCTION\_RESULTING\_IN\_INDUCTION\_OF\_APOPTOSIS |  | 15 | 0.33 | 1.06 | 0.369 | 0.611 | 1.000 | 1105 | tags=27%, list=8%, signal=29% |
| 133 | RESPONSE\_TO\_HORMONE\_STIMULUS |  | 26 | 0.28 | 1.06 | 0.376 | 0.608 | 1.000 | 3483 | tags=38%, list=27%, signal=52% |
| 134 | REGULATION\_OF\_NEUROTRANSMITTER\_LEVELS |  | 23 | 0.29 | 1.05 | 0.381 | 0.625 | 1.000 | 826 | tags=17%, list=6%, signal=19% |
| 135 | CELLULAR\_BIOSYNTHETIC\_PROCESS |  | 273 | 0.18 | 1.05 | 0.325 | 0.623 | 1.000 | 2854 | tags=26%, list=22%, signal=32% |
| 136 | GAMETE\_GENERATION |  | 92 | 0.21 | 1.04 | 0.363 | 0.647 | 1.000 | 3895 | tags=34%, list=30%, signal=48% |
| 137 | REGULATION\_OF\_PROTEIN\_STABILITY |  | 17 | 0.32 | 1.04 | 0.393 | 0.642 | 1.000 | 4128 | tags=41%, list=32%, signal=60% |
| 138 | PIGMENT\_BIOSYNTHETIC\_PROCESS |  | 17 | 0.31 | 1.04 | 0.406 | 0.645 | 1.000 | 1513 | tags=24%, list=12%, signal=27% |
| 139 | INTRACELLULAR\_PROTEIN\_TRANSPORT |  | 127 | 0.19 | 1.04 | 0.376 | 0.646 | 1.000 | 3298 | tags=29%, list=25%, signal=39% |
| 140 | LIPID\_BIOSYNTHETIC\_PROCESS |  | 84 | 0.21 | 1.04 | 0.403 | 0.642 | 1.000 | 1242 | tags=18%, list=9%, signal=20% |
| 141 | NEGATIVE\_REGULATION\_OF\_TRANSFERASE\_ACTIVITY |  | 27 | 0.27 | 1.03 | 0.423 | 0.650 | 1.000 | 2541 | tags=33%, list=19%, signal=41% |
| 142 | CYTOSKELETON\_ORGANIZATION\_AND\_BIOGENESIS |  | 182 | 0.18 | 1.03 | 0.376 | 0.655 | 1.000 | 2944 | tags=27%, list=22%, signal=35% |
| 143 | RESPONSE\_TO\_TEMPERATURE\_STIMULUS |  | 16 | 0.32 | 1.03 | 0.427 | 0.657 | 1.000 | 3467 | tags=44%, list=26%, signal=59% |
| 144 | MACROMOLECULE\_LOCALIZATION |  | 202 | 0.18 | 1.03 | 0.403 | 0.656 | 1.000 | 3139 | tags=27%, list=24%, signal=35% |
| 145 | STEROID\_BIOSYNTHETIC\_PROCESS |  | 22 | 0.29 | 1.02 | 0.428 | 0.655 | 1.000 | 4095 | tags=55%, list=31%, signal=79% |
| 146 | PROTEIN\_TARGETING |  | 94 | 0.20 | 1.01 | 0.432 | 0.693 | 1.000 | 3298 | tags=29%, list=25%, signal=38% |
| 147 | PROTEIN\_IMPORT |  | 58 | 0.22 | 1.01 | 0.443 | 0.691 | 1.000 | 3298 | tags=29%, list=25%, signal=39% |
| 148 | PROTEIN\_TRANSPORT |  | 139 | 0.19 | 1.00 | 0.476 | 0.707 | 1.000 | 3298 | tags=29%, list=25%, signal=38% |
| 149 | SECONDARY\_METABOLIC\_PROCESS |  | 23 | 0.27 | 1.00 | 0.468 | 0.704 | 1.000 | 1513 | tags=22%, list=12%, signal=25% |
| 150 | REGULATION\_OF\_PROGRAMMED\_CELL\_DEATH |  | 313 | 0.16 | 0.99 | 0.481 | 0.715 | 1.000 | 1630 | tags=17%, list=12%, signal=19% |
| 151 | DIGESTION |  | 42 | 0.23 | 0.99 | 0.441 | 0.716 | 1.000 | 2975 | tags=24%, list=23%, signal=31% |
| 152 | NEGATIVE\_REGULATION\_OF\_APOPTOSIS |  | 136 | 0.18 | 0.99 | 0.488 | 0.719 | 1.000 | 1630 | tags=19%, list=12%, signal=22% |
| 153 | PIGMENT\_METABOLIC\_PROCESS |  | 18 | 0.29 | 0.98 | 0.475 | 0.730 | 1.000 | 1513 | tags=22%, list=12%, signal=25% |
| 154 | REGULATION\_OF\_APOPTOSIS |  | 312 | 0.16 | 0.98 | 0.514 | 0.734 | 1.000 | 1630 | tags=17%, list=12%, signal=19% |
| 155 | PROGRAMMED\_CELL\_DEATH |  | 393 | 0.16 | 0.97 | 0.542 | 0.750 | 1.000 | 1653 | tags=17%, list=13%, signal=18% |
| 156 | STEROID\_METABOLIC\_PROCESS |  | 66 | 0.21 | 0.97 | 0.520 | 0.748 | 1.000 | 3759 | tags=35%, list=29%, signal=49% |
| 157 | NEGATIVE\_REGULATION\_OF\_PROGRAMMED\_CELL\_DEATH |  | 137 | 0.18 | 0.97 | 0.510 | 0.744 | 1.000 | 1630 | tags=19%, list=12%, signal=21% |
| 158 | APOPTOSIS\_GO |  | 392 | 0.16 | 0.97 | 0.542 | 0.745 | 1.000 | 1653 | tags=17%, list=13%, signal=18% |
| 159 | ENERGY\_DERIVATION\_BY\_OXIDATION\_OF\_ORGANIC\_COMPOUNDS |  | 37 | 0.24 | 0.97 | 0.496 | 0.745 | 1.000 | 1258 | tags=19%, list=10%, signal=21% |
| 160 | CARBOHYDRATE\_TRANSPORT |  | 17 | 0.29 | 0.96 | 0.517 | 0.756 | 1.000 | 2570 | tags=29%, list=20%, signal=37% |
| 161 | AROMATIC\_COMPOUND\_METABOLIC\_PROCESS |  | 26 | 0.25 | 0.95 | 0.516 | 0.770 | 1.000 | 256 | tags=15%, list=2%, signal=16% |
| 162 | PROTEIN\_RNA\_COMPLEX\_ASSEMBLY |  | 55 | 0.21 | 0.95 | 0.527 | 0.777 | 1.000 | 3787 | tags=38%, list=29%, signal=53% |
| 163 | REGULATION\_OF\_TRANSCRIPTION\_FROM\_RNA\_POLYMERASE\_II\_PROMOTER |  | 267 | 0.16 | 0.95 | 0.586 | 0.774 | 1.000 | 2737 | tags=24%, list=21%, signal=30% |
| 164 | MORPHOGENESIS\_OF\_AN\_EPITHELIUM |  | 15 | 0.30 | 0.95 | 0.511 | 0.774 | 1.000 | 3885 | tags=47%, list=30%, signal=66% |
| 165 | STEROID\_HORMONE\_RECEPTOR\_SIGNALING\_PATHWAY |  | 18 | 0.28 | 0.94 | 0.555 | 0.789 | 1.000 | 1438 | tags=22%, list=11%, signal=25% |
| 166 | COVALENT\_CHROMATIN\_MODIFICATION |  | 22 | 0.27 | 0.94 | 0.541 | 0.787 | 1.000 | 4006 | tags=45%, list=31%, signal=65% |
| 167 | MICROTUBULE\_BASED\_MOVEMENT |  | 16 | 0.29 | 0.94 | 0.549 | 0.786 | 1.000 | 2943 | tags=38%, list=22%, signal=48% |
| 168 | CELL\_PROJECTION\_BIOGENESIS |  | 20 | 0.27 | 0.93 | 0.549 | 0.794 | 1.000 | 4218 | tags=45%, list=32%, signal=66% |
| 169 | INTRACELLULAR\_RECEPTOR\_MEDIATED\_SIGNALING\_PATHWAY |  | 18 | 0.28 | 0.93 | 0.575 | 0.797 | 1.000 | 1438 | tags=22%, list=11%, signal=25% |
| 170 | PROTEIN\_IMPORT\_INTO\_NUCLEUS |  | 45 | 0.21 | 0.93 | 0.573 | 0.793 | 1.000 | 3298 | tags=31%, list=25%, signal=41% |
| 171 | PROTEIN\_AMINO\_ACID\_O\_LINKED\_GLYCOSYLATION |  | 18 | 0.27 | 0.93 | 0.547 | 0.792 | 1.000 | 2825 | tags=39%, list=22%, signal=50% |
| 172 | SPLICEOSOME\_ASSEMBLY |  | 17 | 0.28 | 0.93 | 0.555 | 0.792 | 1.000 | 3787 | tags=41%, list=29%, signal=58% |
| 173 | GENERATION\_OF\_A\_SIGNAL\_INVOLVED\_IN\_CELL\_CELL\_SIGNALING |  | 25 | 0.25 | 0.92 | 0.589 | 0.812 | 1.000 | 2502 | tags=28%, list=19%, signal=35% |
| 174 | INDUCTION\_OF\_APOPTOSIS\_BY\_INTRACELLULAR\_SIGNALS |  | 23 | 0.25 | 0.91 | 0.600 | 0.833 | 1.000 | 1507 | tags=22%, list=12%, signal=25% |
| 175 | BIOSYNTHETIC\_PROCESS |  | 402 | 0.14 | 0.90 | 0.778 | 0.832 | 1.000 | 1820 | tags=17%, list=14%, signal=19% |
| 176 | SEXUAL\_REPRODUCTION |  | 109 | 0.18 | 0.90 | 0.638 | 0.841 | 1.000 | 2762 | tags=22%, list=21%, signal=28% |
| 177 | REGULATION\_OF\_RNA\_METABOLIC\_PROCESS |  | 417 | 0.14 | 0.89 | 0.804 | 0.858 | 1.000 | 3232 | tags=26%, list=25%, signal=34% |
| 178 | POSITIVE\_REGULATION\_OF\_CELL\_CYCLE |  | 15 | 0.28 | 0.89 | 0.627 | 0.859 | 1.000 | 429 | tags=20%, list=3%, signal=21% |
| 179 | REGULATION\_OF\_TRANSPORT |  | 57 | 0.20 | 0.88 | 0.654 | 0.864 | 1.000 | 3479 | tags=33%, list=27%, signal=45% |
| 180 | ENERGY\_RESERVE\_METABOLIC\_PROCESS |  | 15 | 0.28 | 0.88 | 0.613 | 0.871 | 1.000 | 1258 | tags=20%, list=10%, signal=22% |
| 181 | TRANSMISSION\_OF\_NERVE\_IMPULSE |  | 167 | 0.16 | 0.87 | 0.752 | 0.893 | 1.000 | 2754 | tags=21%, list=21%, signal=26% |
| 182 | NEGATIVE\_REGULATION\_OF\_CELL\_ADHESION |  | 16 | 0.27 | 0.86 | 0.634 | 0.897 | 1.000 | 3457 | tags=44%, list=26%, signal=59% |
| 183 | RESPONSE\_TO\_RADIATION |  | 52 | 0.19 | 0.86 | 0.689 | 0.894 | 1.000 | 3362 | tags=31%, list=26%, signal=41% |
| 184 | REGULATION\_OF\_INTRACELLULAR\_TRANSPORT |  | 22 | 0.24 | 0.86 | 0.659 | 0.897 | 1.000 | 4527 | tags=50%, list=35%, signal=76% |
| 185 | EXOCYTOSIS |  | 22 | 0.24 | 0.85 | 0.686 | 0.902 | 1.000 | 2393 | tags=23%, list=18%, signal=28% |
| 186 | EPIDERMAL\_GROWTH\_FACTOR\_RECEPTOR\_SIGNALING\_PATHWAY |  | 18 | 0.25 | 0.84 | 0.690 | 0.922 | 1.000 | 4358 | tags=44%, list=33%, signal=67% |
| 187 | AEROBIC\_RESPIRATION |  | 15 | 0.27 | 0.84 | 0.688 | 0.925 | 1.000 | 2664 | tags=33%, list=20%, signal=42% |
| 188 | SYNAPTIC\_TRANSMISSION |  | 154 | 0.15 | 0.84 | 0.816 | 0.920 | 1.000 | 2754 | tags=20%, list=21%, signal=25% |
| 189 | REGULATION\_OF\_PHOSPHORYLATION |  | 42 | 0.19 | 0.83 | 0.749 | 0.936 | 1.000 | 4265 | tags=45%, list=33%, signal=67% |
| 190 | REGULATION\_OF\_NUCLEOCYTOPLASMIC\_TRANSPORT |  | 19 | 0.24 | 0.82 | 0.719 | 0.947 | 1.000 | 4527 | tags=53%, list=35%, signal=80% |
| 191 | DEVELOPMENT\_OF\_PRIMARY\_SEXUAL\_CHARACTERISTICS |  | 25 | 0.22 | 0.82 | 0.743 | 0.950 | 1.000 | 3071 | tags=28%, list=23%, signal=37% |
| 192 | CELLULAR\_CARBOHYDRATE\_METABOLIC\_PROCESS |  | 106 | 0.16 | 0.81 | 0.838 | 0.953 | 1.000 | 3066 | tags=24%, list=23%, signal=31% |
| 193 | CASPASE\_ACTIVATION |  | 24 | 0.22 | 0.81 | 0.718 | 0.950 | 1.000 | 3357 | tags=38%, list=26%, signal=50% |
| 194 | ESTABLISHMENT\_OF\_PROTEIN\_LOCALIZATION |  | 166 | 0.15 | 0.81 | 0.899 | 0.946 | 1.000 | 3298 | tags=26%, list=25%, signal=34% |
| 195 | HISTONE\_MODIFICATION |  | 21 | 0.23 | 0.80 | 0.727 | 0.960 | 1.000 | 4006 | tags=43%, list=31%, signal=62% |
| 196 | PHOSPHOINOSITIDE\_BIOSYNTHETIC\_PROCESS |  | 21 | 0.23 | 0.79 | 0.756 | 0.965 | 1.000 | 1193 | tags=19%, list=9%, signal=21% |
| 197 | CALCIUM\_INDEPENDENT\_CELL\_CELL\_ADHESION |  | 16 | 0.24 | 0.79 | 0.718 | 0.962 | 1.000 | 3969 | tags=38%, list=30%, signal=54% |
| 198 | CELL\_CYCLE\_ARREST\_GO\_0007050 |  | 52 | 0.18 | 0.79 | 0.827 | 0.961 | 1.000 | 3983 | tags=40%, list=30%, signal=58% |
| 199 | NEGATIVE\_REGULATION\_OF\_CELL\_CYCLE |  | 72 | 0.17 | 0.79 | 0.841 | 0.957 | 1.000 | 2197 | tags=21%, list=17%, signal=25% |
| 200 | MEMBRANE\_FUSION |  | 27 | 0.21 | 0.79 | 0.771 | 0.953 | 1.000 | 3637 | tags=37%, list=28%, signal=51% |
| 201 | NITROGEN\_COMPOUND\_METABOLIC\_PROCESS |  | 141 | 0.15 | 0.79 | 0.920 | 0.952 | 1.000 | 2417 | tags=20%, list=18%, signal=24% |
| 202 | PROTEOLYSIS |  | 170 | 0.14 | 0.78 | 0.926 | 0.953 | 1.000 | 3943 | tags=32%, list=30%, signal=45% |
| 203 | GLUCOSE\_METABOLIC\_PROCESS |  | 27 | 0.21 | 0.78 | 0.786 | 0.948 | 1.000 | 4693 | tags=44%, list=36%, signal=69% |
| 204 | REGULATION\_OF\_CATABOLIC\_PROCESS |  | 15 | 0.25 | 0.78 | 0.754 | 0.949 | 1.000 | 2634 | tags=33%, list=20%, signal=42% |
| 205 | PROTEIN\_LOCALIZATION |  | 184 | 0.14 | 0.78 | 0.941 | 0.950 | 1.000 | 3215 | tags=25%, list=25%, signal=33% |
| 206 | MEMBRANE\_LIPID\_BIOSYNTHETIC\_PROCESS |  | 41 | 0.18 | 0.77 | 0.836 | 0.952 | 1.000 | 3926 | tags=34%, list=30%, signal=49% |
| 207 | RESPONSE\_TO\_UV |  | 22 | 0.21 | 0.76 | 0.806 | 0.963 | 1.000 | 3362 | tags=36%, list=26%, signal=49% |
| 208 | LIPID\_TRANSPORT |  | 27 | 0.20 | 0.74 | 0.852 | 0.982 | 1.000 | 2032 | tags=22%, list=16%, signal=26% |
| 209 | REPRODUCTION |  | 215 | 0.13 | 0.74 | 0.980 | 0.979 | 1.000 | 3116 | tags=22%, list=24%, signal=29% |
| 210 | EMBRYONIC\_DEVELOPMENT |  | 46 | 0.17 | 0.74 | 0.877 | 0.975 | 1.000 | 3071 | tags=26%, list=23%, signal=34% |
| 211 | STRESS\_ACTIVATED\_PROTEIN\_KINASE\_SIGNALING\_PATHWAY |  | 45 | 0.17 | 0.73 | 0.911 | 0.981 | 1.000 | 4152 | tags=38%, list=32%, signal=55% |
| 212 | JNK\_CASCADE |  | 44 | 0.17 | 0.73 | 0.886 | 0.979 | 1.000 | 4152 | tags=39%, list=32%, signal=56% |
| 213 | PHOSPHOLIPID\_BIOSYNTHETIC\_PROCESS |  | 35 | 0.18 | 0.73 | 0.899 | 0.975 | 1.000 | 1650 | tags=17%, list=13%, signal=20% |
| 214 | SENSORY\_PERCEPTION |  | 163 | 0.13 | 0.72 | 0.976 | 0.978 | 1.000 | 5249 | tags=42%, list=40%, signal=70% |
| 215 | GLYCEROPHOSPHOLIPID\_BIOSYNTHETIC\_PROCESS |  | 27 | 0.19 | 0.71 | 0.886 | 0.983 | 1.000 | 1193 | tags=15%, list=9%, signal=16% |
| 216 | NEUROLOGICAL\_SYSTEM\_PROCESS |  | 328 | 0.12 | 0.70 | 1.000 | 0.984 | 1.000 | 2754 | tags=17%, list=21%, signal=21% |
| 217 | POSITIVE\_REGULATION\_OF\_HYDROLASE\_ACTIVITY |  | 45 | 0.16 | 0.69 | 0.948 | 0.989 | 1.000 | 3357 | tags=29%, list=26%, signal=39% |
| 218 | BRAIN\_DEVELOPMENT |  | 39 | 0.17 | 0.69 | 0.923 | 0.988 | 1.000 | 3071 | tags=28%, list=23%, signal=37% |
| 219 | LIPOPROTEIN\_METABOLIC\_PROCESS |  | 30 | 0.18 | 0.69 | 0.909 | 0.986 | 1.000 | 2032 | tags=20%, list=16%, signal=24% |
| 220 | HOMEOSTASIS\_OF\_NUMBER\_OF\_CELLS |  | 20 | 0.20 | 0.68 | 0.899 | 0.987 | 1.000 | 2609 | tags=25%, list=20%, signal=31% |
| 221 | ANION\_TRANSPORT |  | 27 | 0.18 | 0.68 | 0.919 | 0.985 | 1.000 | 2906 | tags=22%, list=22%, signal=29% |
| 222 | BIOGENIC\_AMINE\_METABOLIC\_PROCESS |  | 16 | 0.21 | 0.67 | 0.896 | 0.982 | 1.000 | 1650 | tags=19%, list=13%, signal=21% |
| 223 | NEGATIVE\_REGULATION\_OF\_CELLULAR\_BIOSYNTHETIC\_PROCESS |  | 25 | 0.18 | 0.67 | 0.943 | 0.978 | 1.000 | 3307 | tags=28%, list=25%, signal=37% |
| 224 | REGULATION\_OF\_CELL\_ADHESION |  | 31 | 0.17 | 0.64 | 0.954 | 0.991 | 1.000 | 4186 | tags=42%, list=32%, signal=62% |
| 225 | NEGATIVE\_REGULATION\_OF\_BIOSYNTHETIC\_PROCESS |  | 26 | 0.17 | 0.63 | 0.936 | 0.993 | 1.000 | 3307 | tags=27%, list=25%, signal=36% |
| 226 | ADENYLATE\_CYCLASE\_ACTIVATION |  | 18 | 0.18 | 0.61 | 0.956 | 0.998 | 1.000 | 4526 | tags=39%, list=35%, signal=59% |
| 227 | LIPOPROTEIN\_BIOSYNTHETIC\_PROCESS |  | 23 | 0.16 | 0.56 | 0.972 | 1.000 | 1.000 | 4060 | tags=35%, list=31%, signal=50% |
| 228 | FEEDING\_BEHAVIOR |  | 20 | 0.16 | 0.56 | 0.982 | 1.000 | 1.000 | 5180 | tags=45%, list=40%, signal=74% |
| 229 | TUBE\_DEVELOPMENT |  | 15 | 0.17 | 0.55 | 0.980 | 0.999 | 1.000 | 3885 | tags=33%, list=30%, signal=47% |
| 230 | DETECTION\_OF\_ABIOTIC\_STIMULUS |  | 16 | 0.16 | 0.53 | 0.983 | 0.997 | 1.000 | 61 | tags=6%, list=0%, signal=6% |
| 231 | NEGATIVE\_REGULATION\_OF\_TRANSLATION |  | 19 | 0.14 | 0.48 | 0.998 | 0.998 | 1.000 | 3307 | tags=26%, list=25%, signal=35% |
Table: Gene sets enriched in phenotype **na**[plain text format]****

  
