# Supplementary material for: Multiple-input multiple-output causal strategies for gene selection
Source: BMC Bioinformatics. 2011 Nov 25;12:458. doi: 10.1186/1471-2105-12-458 (PMC3323860; doi:10.1186/1471-2105-12-458)
Supplement: Additional file 2 — Archive containing the output files computed by the preranked GSEA for λ ∈ {0.1,0.2,0.3,0.4,0.5} (GSEA_MIMO_part1.zip). [file 1471-2105-12-458-S2.ZIP › mFS02_entrez_mimo.GseaPreranked.1316038034143/index.html]

Index for xtools.gsea.GseaPreranked mFS02\_entrez\_mimo.GseaPreranked.1316038034143

### GSEA Report for Dataset mFS02\_entrez\_mimo

#### Enrichment in phenotype: **na**

- 231 / 548 gene sets are upregulated in phenotype **na\_pos**- 93 gene sets are significant at FDR < 25%- 59 gene sets are significantly enriched at nominal pvalue < 1%- 80 gene sets are significantly enriched at nominal pvalue < 5%- Snapshot of enrichment results- Detailed enrichment results in html format- Detailed enrichment results in excel format (tab delimited text)- Guide to interpret results

#### Enrichment in phenotype: **na**

- 317 / 548 gene sets are upregulated in phenotype **na\_neg**- 32 gene sets are significantly enriched at FDR < 25%- 24 gene sets are significantly enriched at nominal pvalue < 1%- 54 gene sets are significantly enriched at nominal pvalue < 5%- Snapshot of enrichment results- Detailed enrichment results in html format- Detailed enrichment results in excel format (tab delimited text)- Guide to interpret results

#### Dataset details

- The dataset has 13091 features (genes)- No probe set => gene symbol collapsing was requested, so all 13091 features were used

#### Gene set details

- Gene set size filters (min=15, max=500) resulted in filtering out 277 / 825 gene sets- The remaining 548 gene sets were used in the analysis- List of gene sets used and their sizes (restricted to features in the specified dataset)

#### Gene markers for the **na\_pos** *versus* **na\_neg** comparison

- The dataset has 13091 features (genes)- Detailed rank ordered gene list for all features in the dataset

#### Global statistics and plots

- Plot of p-values *vs.* NES- Global ES histogram

#### Other

- Parameters used for this analysis

---

Report: mFS02\_entrez\_mimo.GseaPreranked.1316038034143.rpt   by user: bhaibeka

xtools.gsea.GseaPreranked [Wed, Sep 14, '11 6 PM 7]

Website: www.broadinstitute.org/GSEA
Questions & Suggestions: Email
