# Supplementary material for: Multiple-input multiple-output causal strategies for gene selection
Source: BMC Bioinformatics. 2011 Nov 25;12:458. doi: 10.1186/1471-2105-12-458 (PMC3323860; doi:10.1186/1471-2105-12-458)
Supplement: Additional file 2 — Archive containing the output files computed by the preranked GSEA for λ ∈ {0.1,0.2,0.3,0.4,0.5} (GSEA_MIMO_part1.zip). [file 1471-2105-12-458-S2.ZIP › mFS03_entrez_mimo.GseaPreranked.1316038204362/gsea_report_for_na_neg_1316038204362.html]

Report for na\_neg 1316038204362 [GSEA]

| GS  follow link to MSigDB | GS DETAILS | SIZE | ES | NES | NOM p-val | FDR q-val | FWER p-val | RANK AT MAX | LEADING EDGE || 1 | IMMUNE\_RESPONSE |  | 212 | -0.39 | -2.34 | 0.000 | 0.005 | 0.004 | 3108 | tags=44%, list=24%, signal=57% |
| 2 | IMMUNE\_SYSTEM\_PROCESS |  | 298 | -0.36 | -2.26 | 0.000 | 0.006 | 0.010 | 3108 | tags=42%, list=24%, signal=53% |
| 3 | DEFENSE\_RESPONSE |  | 238 | -0.35 | -2.16 | 0.000 | 0.012 | 0.027 | 3211 | tags=40%, list=25%, signal=52% |
| 4 | REGULATION\_OF\_IMMUNE\_RESPONSE |  | 28 | -0.52 | -2.09 | 0.000 | 0.019 | 0.058 | 4551 | tags=75%, list=35%, signal=115% |
| 5 | POSITIVE\_REGULATION\_OF\_IMMUNE\_RESPONSE |  | 24 | -0.55 | -2.06 | 0.000 | 0.019 | 0.072 | 4443 | tags=75%, list=34%, signal=113% |
| 6 | POSITIVE\_REGULATION\_OF\_IMMUNE\_SYSTEM\_PROCESS |  | 44 | -0.46 | -2.02 | 0.000 | 0.024 | 0.109 | 4159 | tags=61%, list=32%, signal=90% |
| 7 | INFLAMMATORY\_RESPONSE |  | 115 | -0.37 | -1.99 | 0.000 | 0.029 | 0.145 | 3007 | tags=41%, list=23%, signal=53% |
| 8 | POSITIVE\_REGULATION\_OF\_MULTICELLULAR\_ORGANISMAL\_PROCESS |  | 56 | -0.43 | -1.99 | 0.000 | 0.025 | 0.145 | 4159 | tags=59%, list=32%, signal=86% |
| 9 | RESPONSE\_TO\_WOUNDING |  | 171 | -0.34 | -1.95 | 0.000 | 0.032 | 0.200 | 3199 | tags=40%, list=24%, signal=52% |
| 10 | REGULATION\_OF\_IMMUNE\_SYSTEM\_PROCESS |  | 57 | -0.41 | -1.93 | 0.000 | 0.034 | 0.233 | 4159 | tags=60%, list=32%, signal=87% |
| 11 | CELLULAR\_DEFENSE\_RESPONSE |  | 54 | -0.41 | -1.89 | 0.000 | 0.050 | 0.348 | 3727 | tags=48%, list=28%, signal=67% |
| 12 | ADAPTIVE\_IMMUNE\_RESPONSE\_GO\_0002460 |  | 22 | -0.49 | -1.78 | 0.014 | 0.105 | 0.630 | 4070 | tags=59%, list=31%, signal=86% |
| 13 | ADAPTIVE\_IMMUNE\_RESPONSE |  | 23 | -0.46 | -1.75 | 0.009 | 0.131 | 0.738 | 4070 | tags=57%, list=31%, signal=82% |
| 14 | HEMOPOIETIC\_OR\_LYMPHOID\_ORGAN\_DEVELOPMENT |  | 71 | -0.35 | -1.72 | 0.000 | 0.155 | 0.819 | 2932 | tags=39%, list=22%, signal=51% |
| 15 | HEMOPOIESIS |  | 69 | -0.35 | -1.70 | 0.004 | 0.159 | 0.843 | 2932 | tags=39%, list=22%, signal=50% |
| 16 | REGULATION\_OF\_MULTICELLULAR\_ORGANISMAL\_PROCESS |  | 131 | -0.31 | -1.70 | 0.002 | 0.157 | 0.854 | 3077 | tags=37%, list=24%, signal=48% |
| 17 | HUMORAL\_IMMUNE\_RESPONSE |  | 30 | -0.42 | -1.69 | 0.011 | 0.159 | 0.870 | 3108 | tags=53%, list=24%, signal=70% |
| 18 | REGULATION\_OF\_DEFENSE\_RESPONSE |  | 15 | -0.52 | -1.68 | 0.025 | 0.161 | 0.890 | 4070 | tags=67%, list=31%, signal=97% |
| 19 | REGULATION\_OF\_CELL\_DIFFERENTIATION |  | 48 | -0.38 | -1.68 | 0.004 | 0.154 | 0.894 | 4604 | tags=56%, list=35%, signal=86% |
| 20 | IMMUNE\_EFFECTOR\_PROCESS |  | 34 | -0.40 | -1.67 | 0.009 | 0.163 | 0.919 | 2797 | tags=50%, list=21%, signal=63% |
| 21 | IMMUNE\_SYSTEM\_DEVELOPMENT |  | 75 | -0.33 | -1.66 | 0.002 | 0.158 | 0.921 | 2932 | tags=39%, list=22%, signal=50% |
| 22 | LIPID\_CATABOLIC\_PROCESS |  | 34 | -0.40 | -1.66 | 0.011 | 0.155 | 0.928 | 4034 | tags=56%, list=31%, signal=81% |
| 23 | RESPONSE\_TO\_EXTERNAL\_STIMULUS |  | 278 | -0.26 | -1.64 | 0.000 | 0.164 | 0.940 | 3208 | tags=34%, list=25%, signal=44% |
| 24 | TRANSFORMING\_GROWTH\_FACTOR\_BETA\_RECEPTOR\_SIGNALING\_PATHWAY |  | 34 | -0.40 | -1.64 | 0.020 | 0.161 | 0.942 | 3056 | tags=44%, list=23%, signal=57% |
| 25 | RECEPTOR\_MEDIATED\_ENDOCYTOSIS |  | 31 | -0.42 | -1.63 | 0.011 | 0.169 | 0.958 | 2201 | tags=35%, list=17%, signal=43% |
| 26 | LYMPHOCYTE\_ACTIVATION |  | 54 | -0.34 | -1.61 | 0.004 | 0.186 | 0.970 | 3331 | tags=46%, list=25%, signal=62% |
| 27 | JAK\_STAT\_CASCADE |  | 26 | -0.41 | -1.58 | 0.032 | 0.222 | 0.989 | 1705 | tags=35%, list=13%, signal=40% |
| 28 | LEUKOCYTE\_DIFFERENTIATION |  | 34 | -0.38 | -1.58 | 0.009 | 0.215 | 0.989 | 2888 | tags=44%, list=22%, signal=56% |
| 29 | POSITIVE\_REGULATION\_OF\_RESPONSE\_TO\_STIMULUS |  | 35 | -0.37 | -1.56 | 0.031 | 0.229 | 0.993 | 2974 | tags=46%, list=23%, signal=59% |
| 30 | CELL\_ACTIVATION |  | 64 | -0.32 | -1.55 | 0.007 | 0.236 | 0.996 | 4094 | tags=52%, list=31%, signal=75% |
| 31 | REGULATION\_OF\_ANGIOGENESIS |  | 24 | -0.41 | -1.55 | 0.037 | 0.237 | 0.998 | 2360 | tags=46%, list=18%, signal=56% |
| 32 | T\_CELL\_ACTIVATION |  | 39 | -0.36 | -1.54 | 0.032 | 0.243 | 0.999 | 3267 | tags=44%, list=25%, signal=58% |
| 33 | ENZYME\_LINKED\_RECEPTOR\_PROTEIN\_SIGNALING\_PATHWAY |  | 128 | -0.28 | -1.53 | 0.005 | 0.247 | 0.999 | 3180 | tags=33%, list=24%, signal=43% |
| 34 | TRANSMEMBRANE\_RECEPTOR\_PROTEIN\_SERINE\_THREONINE\_KINASE\_SIGNALING\_PATHWAY |  | 42 | -0.35 | -1.53 | 0.022 | 0.249 | 0.999 | 3163 | tags=40%, list=24%, signal=53% |
| 35 | ACTIN\_CYTOSKELETON\_ORGANIZATION\_AND\_BIOGENESIS |  | 90 | -0.29 | -1.52 | 0.015 | 0.256 | 0.999 | 1828 | tags=26%, list=14%, signal=29% |
| 36 | LEUKOCYTE\_ACTIVATION |  | 59 | -0.32 | -1.51 | 0.011 | 0.260 | 0.999 | 3331 | tags=44%, list=25%, signal=59% |
| 37 | POSITIVE\_REGULATION\_OF\_PHOSPHATE\_METABOLIC\_PROCESS |  | 23 | -0.40 | -1.49 | 0.041 | 0.285 | 0.999 | 1401 | tags=30%, list=11%, signal=34% |
| 38 | LYMPHOCYTE\_DIFFERENTIATION |  | 23 | -0.41 | -1.49 | 0.047 | 0.284 | 0.999 | 3267 | tags=52%, list=25%, signal=69% |
| 39 | CELLULAR\_LIPID\_CATABOLIC\_PROCESS |  | 31 | -0.37 | -1.49 | 0.051 | 0.281 | 0.999 | 4024 | tags=52%, list=31%, signal=74% |
| 40 | NEGATIVE\_REGULATION\_OF\_SIGNAL\_TRANSDUCTION |  | 31 | -0.37 | -1.49 | 0.048 | 0.280 | 1.000 | 4027 | tags=48%, list=31%, signal=70% |
| 41 | WOUND\_HEALING |  | 49 | -0.33 | -1.48 | 0.033 | 0.277 | 1.000 | 3943 | tags=45%, list=30%, signal=64% |
| 42 | POSITIVE\_REGULATION\_OF\_CELL\_DIFFERENTIATION |  | 21 | -0.41 | -1.48 | 0.059 | 0.276 | 1.000 | 4456 | tags=67%, list=34%, signal=101% |
| 43 | REGULATION\_OF\_RESPONSE\_TO\_STIMULUS |  | 49 | -0.32 | -1.47 | 0.024 | 0.289 | 1.000 | 4551 | tags=61%, list=35%, signal=93% |
| 44 | B\_CELL\_ACTIVATION |  | 17 | -0.43 | -1.46 | 0.066 | 0.290 | 1.000 | 3331 | tags=59%, list=25%, signal=79% |
| 45 | INNATE\_IMMUNE\_RESPONSE |  | 19 | -0.41 | -1.46 | 0.058 | 0.292 | 1.000 | 4830 | tags=74%, list=37%, signal=117% |
| 46 | MESODERM\_DEVELOPMENT |  | 22 | -0.39 | -1.43 | 0.073 | 0.335 | 1.000 | 4389 | tags=55%, list=34%, signal=82% |
| 47 | PROTEIN\_AMINO\_ACID\_N\_LINKED\_GLYCOSYLATION |  | 27 | -0.36 | -1.43 | 0.049 | 0.329 | 1.000 | 2507 | tags=37%, list=19%, signal=46% |
| 48 | CYTOKINE\_AND\_CHEMOKINE\_MEDIATED\_SIGNALING\_PATHWAY |  | 19 | -0.41 | -1.43 | 0.065 | 0.326 | 1.000 | 2237 | tags=37%, list=17%, signal=44% |
| 49 | FATTY\_ACID\_METABOLIC\_PROCESS |  | 56 | -0.31 | -1.43 | 0.048 | 0.320 | 1.000 | 4024 | tags=50%, list=31%, signal=72% |
| 50 | POSITIVE\_REGULATION\_OF\_SIGNAL\_TRANSDUCTION |  | 97 | -0.27 | -1.43 | 0.014 | 0.316 | 1.000 | 4081 | tags=47%, list=31%, signal=68% |
| 51 | CELL\_SUBSTRATE\_ADHESION |  | 36 | -0.33 | -1.43 | 0.074 | 0.319 | 1.000 | 1706 | tags=31%, list=13%, signal=35% |
| 52 | PROTEIN\_AMINO\_ACID\_PHOSPHORYLATION |  | 231 | -0.23 | -1.42 | 0.000 | 0.325 | 1.000 | 2999 | tags=30%, list=23%, signal=38% |
| 53 | MULTI\_ORGANISM\_PROCESS |  | 137 | -0.25 | -1.42 | 0.016 | 0.321 | 1.000 | 3087 | tags=35%, list=24%, signal=45% |
| 54 | RESPONSE\_TO\_OTHER\_ORGANISM |  | 69 | -0.29 | -1.42 | 0.051 | 0.318 | 1.000 | 2948 | tags=38%, list=23%, signal=48% |
| 55 | COAGULATION |  | 41 | -0.32 | -1.41 | 0.050 | 0.323 | 1.000 | 3444 | tags=39%, list=26%, signal=53% |
| 56 | CATION\_HOMEOSTASIS |  | 94 | -0.27 | -1.41 | 0.026 | 0.319 | 1.000 | 3218 | tags=36%, list=25%, signal=48% |
| 57 | REGULATION\_OF\_PROTEIN\_AMINO\_ACID\_PHOSPHORYLATION |  | 23 | -0.38 | -1.41 | 0.071 | 0.314 | 1.000 | 2625 | tags=35%, list=20%, signal=43% |
| 58 | BLOOD\_COAGULATION |  | 41 | -0.32 | -1.41 | 0.074 | 0.313 | 1.000 | 3444 | tags=39%, list=26%, signal=53% |
| 59 | AMINE\_TRANSPORT |  | 36 | -0.34 | -1.40 | 0.062 | 0.324 | 1.000 | 2202 | tags=28%, list=17%, signal=33% |
| 60 | REGULATION\_OF\_SIGNAL\_TRANSDUCTION |  | 173 | -0.24 | -1.39 | 0.024 | 0.331 | 1.000 | 4081 | tags=42%, list=31%, signal=60% |
| 61 | POSITIVE\_REGULATION\_OF\_PROTEIN\_AMINO\_ACID\_PHOSPHORYLATION |  | 15 | -0.42 | -1.39 | 0.090 | 0.326 | 1.000 | 1401 | tags=33%, list=11%, signal=37% |
| 62 | PEPTIDYL\_TYROSINE\_MODIFICATION |  | 23 | -0.38 | -1.39 | 0.080 | 0.322 | 1.000 | 1568 | tags=26%, list=12%, signal=30% |
| 63 | SMALL\_GTPASE\_MEDIATED\_SIGNAL\_TRANSDUCTION |  | 77 | -0.27 | -1.38 | 0.033 | 0.329 | 1.000 | 3353 | tags=39%, list=26%, signal=52% |
| 64 | REGULATION\_OF\_BLOOD\_PRESSURE |  | 22 | -0.37 | -1.38 | 0.094 | 0.332 | 1.000 | 3412 | tags=41%, list=26%, signal=55% |
| 65 | REGULATION\_OF\_LYMPHOCYTE\_ACTIVATION |  | 31 | -0.35 | -1.38 | 0.082 | 0.327 | 1.000 | 3267 | tags=45%, list=25%, signal=60% |
| 66 | CELLULAR\_CATION\_HOMEOSTASIS |  | 91 | -0.27 | -1.38 | 0.042 | 0.326 | 1.000 | 3218 | tags=36%, list=25%, signal=48% |
| 67 | MAINTENANCE\_OF\_LOCALIZATION |  | 21 | -0.39 | -1.37 | 0.095 | 0.334 | 1.000 | 2678 | tags=38%, list=20%, signal=48% |
| 68 | TRANSMEMBRANE\_RECEPTOR\_PROTEIN\_TYROSINE\_KINASE\_SIGNALING\_PATHWAY |  | 76 | -0.27 | -1.36 | 0.053 | 0.348 | 1.000 | 1374 | tags=21%, list=10%, signal=23% |
| 69 | DETECTION\_OF\_STIMULUS |  | 36 | -0.33 | -1.36 | 0.082 | 0.344 | 1.000 | 5086 | tags=56%, list=39%, signal=91% |
| 70 | GROWTH |  | 59 | -0.29 | -1.36 | 0.065 | 0.340 | 1.000 | 4126 | tags=44%, list=32%, signal=64% |
| 71 | FEMALE\_PREGNANCY |  | 42 | -0.31 | -1.36 | 0.101 | 0.337 | 1.000 | 4166 | tags=52%, list=32%, signal=77% |
| 72 | ACTIN\_FILAMENT\_BASED\_PROCESS |  | 99 | -0.26 | -1.34 | 0.046 | 0.364 | 1.000 | 1828 | tags=23%, list=14%, signal=27% |
| 73 | REGULATION\_OF\_ANATOMICAL\_STRUCTURE\_MORPHOGENESIS |  | 17 | -0.39 | -1.34 | 0.113 | 0.360 | 1.000 | 4697 | tags=53%, list=36%, signal=82% |
| 74 | GLYCOPROTEIN\_METABOLIC\_PROCESS |  | 82 | -0.26 | -1.34 | 0.049 | 0.369 | 1.000 | 3631 | tags=39%, list=28%, signal=54% |
| 75 | RESPONSE\_TO\_VIRUS |  | 45 | -0.30 | -1.34 | 0.088 | 0.365 | 1.000 | 2948 | tags=42%, list=23%, signal=54% |
| 76 | POSITIVE\_REGULATION\_OF\_CYTOKINE\_BIOSYNTHETIC\_PROCESS |  | 21 | -0.37 | -1.33 | 0.118 | 0.375 | 1.000 | 2886 | tags=43%, list=22%, signal=55% |
| 77 | AMINO\_ACID\_TRANSPORT |  | 25 | -0.35 | -1.33 | 0.109 | 0.371 | 1.000 | 2202 | tags=32%, list=17%, signal=38% |
| 78 | POSITIVE\_REGULATION\_OF\_LYMPHOCYTE\_ACTIVATION |  | 23 | -0.36 | -1.32 | 0.111 | 0.380 | 1.000 | 3267 | tags=43%, list=25%, signal=58% |
| 79 | MUSCLE\_DEVELOPMENT |  | 85 | -0.26 | -1.32 | 0.059 | 0.376 | 1.000 | 3411 | tags=40%, list=26%, signal=54% |
| 80 | REGULATION\_OF\_T\_CELL\_ACTIVATION |  | 25 | -0.34 | -1.32 | 0.100 | 0.374 | 1.000 | 3267 | tags=44%, list=25%, signal=59% |
| 81 | ANATOMICAL\_STRUCTURE\_FORMATION |  | 52 | -0.28 | -1.32 | 0.089 | 0.370 | 1.000 | 1930 | tags=29%, list=15%, signal=34% |
| 82 | PROTEIN\_KINASE\_CASCADE |  | 239 | -0.22 | -1.32 | 0.023 | 0.371 | 1.000 | 2251 | tags=25%, list=17%, signal=30% |
| 83 | PROTEIN\_COMPLEX\_ASSEMBLY |  | 157 | -0.23 | -1.30 | 0.047 | 0.394 | 1.000 | 2410 | tags=26%, list=18%, signal=32% |
| 84 | POSITIVE\_REGULATION\_OF\_PHOSPHORYLATION |  | 21 | -0.36 | -1.30 | 0.144 | 0.390 | 1.000 | 1401 | tags=29%, list=11%, signal=32% |
| 85 | PROTEIN\_AMINO\_ACID\_DEPHOSPHORYLATION |  | 60 | -0.28 | -1.30 | 0.112 | 0.393 | 1.000 | 1548 | tags=22%, list=12%, signal=24% |
| 86 | CELL\_MATRIX\_ADHESION |  | 35 | -0.31 | -1.29 | 0.114 | 0.401 | 1.000 | 1706 | tags=29%, list=13%, signal=33% |
| 87 | RESPONSE\_TO\_DRUG |  | 21 | -0.36 | -1.29 | 0.151 | 0.401 | 1.000 | 2548 | tags=43%, list=19%, signal=53% |
| 88 | HEMOSTASIS |  | 46 | -0.29 | -1.29 | 0.094 | 0.406 | 1.000 | 3444 | tags=37%, list=26%, signal=50% |
| 89 | CYTOKINE\_PRODUCTION |  | 61 | -0.27 | -1.28 | 0.086 | 0.411 | 1.000 | 2886 | tags=34%, list=22%, signal=44% |
| 90 | REGULATION\_OF\_CYTOSKELETON\_ORGANIZATION\_AND\_BIOGENESIS |  | 26 | -0.33 | -1.28 | 0.133 | 0.412 | 1.000 | 2625 | tags=35%, list=20%, signal=43% |
| 91 | PROTEIN\_PROCESSING |  | 41 | -0.29 | -1.27 | 0.120 | 0.425 | 1.000 | 4027 | tags=41%, list=31%, signal=60% |
| 92 | DEPHOSPHORYLATION |  | 67 | -0.25 | -1.27 | 0.112 | 0.429 | 1.000 | 1548 | tags=21%, list=12%, signal=24% |
| 93 | ACTIN\_POLYMERIZATION\_AND\_OR\_DEPOLYMERIZATION |  | 20 | -0.36 | -1.27 | 0.169 | 0.427 | 1.000 | 2410 | tags=30%, list=18%, signal=37% |
| 94 | REGULATION\_OF\_CELL\_PROLIFERATION |  | 275 | -0.20 | -1.27 | 0.018 | 0.425 | 1.000 | 2646 | tags=26%, list=20%, signal=32% |
| 95 | MONOCARBOXYLIC\_ACID\_METABOLIC\_PROCESS |  | 77 | -0.25 | -1.27 | 0.084 | 0.423 | 1.000 | 4046 | tags=44%, list=31%, signal=64% |
| 96 | RAS\_PROTEIN\_SIGNAL\_TRANSDUCTION |  | 55 | -0.28 | -1.26 | 0.124 | 0.425 | 1.000 | 3353 | tags=40%, list=26%, signal=54% |
| 97 | REGULATION\_OF\_BODY\_FLUID\_LEVELS |  | 55 | -0.27 | -1.26 | 0.123 | 0.421 | 1.000 | 3444 | tags=36%, list=26%, signal=49% |
| 98 | ANGIOGENESIS |  | 44 | -0.29 | -1.26 | 0.138 | 0.420 | 1.000 | 2543 | tags=34%, list=19%, signal=42% |
| 99 | POSITIVE\_REGULATION\_OF\_SECRETION |  | 18 | -0.37 | -1.26 | 0.180 | 0.416 | 1.000 | 4357 | tags=61%, list=33%, signal=91% |
| 100 | LIPID\_METABOLIC\_PROCESS |  | 283 | -0.20 | -1.26 | 0.036 | 0.417 | 1.000 | 3897 | tags=39%, list=30%, signal=54% |
| 101 | PEPTIDYL\_TYROSINE\_PHOSPHORYLATION |  | 21 | -0.34 | -1.26 | 0.169 | 0.418 | 1.000 | 1568 | tags=24%, list=12%, signal=27% |
| 102 | PROTEIN\_OLIGOMERIZATION |  | 37 | -0.31 | -1.25 | 0.133 | 0.432 | 1.000 | 2616 | tags=32%, list=20%, signal=40% |
| 103 | POSITIVE\_REGULATION\_OF\_TRANSLATION |  | 28 | -0.31 | -1.23 | 0.170 | 0.461 | 1.000 | 2886 | tags=39%, list=22%, signal=50% |
| 104 | PHOSPHOLIPID\_METABOLIC\_PROCESS |  | 63 | -0.25 | -1.23 | 0.129 | 0.467 | 1.000 | 3769 | tags=41%, list=29%, signal=58% |
| 105 | ICOSANOID\_METABOLIC\_PROCESS |  | 16 | -0.38 | -1.23 | 0.227 | 0.465 | 1.000 | 3688 | tags=50%, list=28%, signal=70% |
| 106 | POSITIVE\_REGULATION\_OF\_TRANSFERASE\_ACTIVITY |  | 71 | -0.25 | -1.23 | 0.122 | 0.463 | 1.000 | 2412 | tags=27%, list=18%, signal=33% |
| 107 | PHOSPHORYLATION |  | 262 | -0.20 | -1.22 | 0.079 | 0.466 | 1.000 | 2999 | tags=29%, list=23%, signal=36% |
| 108 | ORGAN\_MORPHOGENESIS |  | 131 | -0.22 | -1.22 | 0.092 | 0.472 | 1.000 | 1465 | tags=19%, list=11%, signal=21% |
| 109 | G\_PROTEIN\_SIGNALING\_COUPLED\_TO\_CAMP\_NUCLEOTIDE\_SECOND\_MESSENGER |  | 62 | -0.25 | -1.22 | 0.162 | 0.471 | 1.000 | 2084 | tags=21%, list=16%, signal=25% |
| 110 | CAMP\_MEDIATED\_SIGNALING |  | 63 | -0.25 | -1.22 | 0.152 | 0.468 | 1.000 | 2084 | tags=21%, list=16%, signal=24% |
| 111 | POSITIVE\_REGULATION\_OF\_CELL\_PROLIFERATION |  | 129 | -0.22 | -1.21 | 0.120 | 0.480 | 1.000 | 1915 | tags=22%, list=15%, signal=26% |
| 112 | NEGATIVE\_REGULATION\_OF\_TRANSCRIPTION |  | 166 | -0.21 | -1.21 | 0.116 | 0.477 | 1.000 | 2673 | tags=28%, list=20%, signal=34% |
| 113 | MUSCLE\_CELL\_DIFFERENTIATION |  | 21 | -0.33 | -1.21 | 0.206 | 0.473 | 1.000 | 3411 | tags=48%, list=26%, signal=64% |
| 114 | PROTEIN\_AUTOPROCESSING |  | 24 | -0.32 | -1.20 | 0.197 | 0.485 | 1.000 | 4027 | tags=46%, list=31%, signal=66% |
| 115 | REGULATION\_OF\_I\_KAPPAB\_KINASE\_NF\_KAPPAB\_CASCADE |  | 72 | -0.24 | -1.20 | 0.146 | 0.482 | 1.000 | 4081 | tags=47%, list=31%, signal=68% |
| 116 | RESPONSE\_TO\_BACTERIUM |  | 22 | -0.32 | -1.20 | 0.229 | 0.487 | 1.000 | 1836 | tags=27%, list=14%, signal=32% |
| 117 | PROTEIN\_AMINO\_ACID\_AUTOPHOSPHORYLATION |  | 24 | -0.32 | -1.19 | 0.214 | 0.512 | 1.000 | 4027 | tags=46%, list=31%, signal=66% |
| 118 | CELL\_RECOGNITION |  | 16 | -0.36 | -1.19 | 0.239 | 0.516 | 1.000 | 4070 | tags=50%, list=31%, signal=72% |
| 119 | POSITIVE\_REGULATION\_OF\_CELLULAR\_PROTEIN\_METABOLIC\_PROCESS |  | 61 | -0.24 | -1.18 | 0.194 | 0.515 | 1.000 | 2410 | tags=30%, list=18%, signal=36% |
| 120 | NEURON\_DIFFERENTIATION |  | 58 | -0.25 | -1.18 | 0.172 | 0.512 | 1.000 | 3431 | tags=33%, list=26%, signal=44% |
| 121 | ACTIVATION\_OF\_NF\_KAPPAB\_TRANSCRIPTION\_FACTOR |  | 15 | -0.37 | -1.18 | 0.219 | 0.511 | 1.000 | 4236 | tags=60%, list=32%, signal=89% |
| 122 | POSITIVE\_REGULATION\_OF\_PROTEIN\_METABOLIC\_PROCESS |  | 63 | -0.25 | -1.18 | 0.194 | 0.509 | 1.000 | 2410 | tags=30%, list=18%, signal=37% |
| 123 | VASCULATURE\_DEVELOPMENT |  | 50 | -0.25 | -1.18 | 0.188 | 0.506 | 1.000 | 1930 | tags=26%, list=15%, signal=30% |
| 124 | GENERATION\_OF\_NEURONS |  | 65 | -0.24 | -1.18 | 0.174 | 0.504 | 1.000 | 3659 | tags=35%, list=28%, signal=49% |
| 125 | REGULATION\_OF\_PROTEIN\_METABOLIC\_PROCESS |  | 150 | -0.21 | -1.18 | 0.143 | 0.506 | 1.000 | 2925 | tags=29%, list=22%, signal=37% |
| 126 | BONE\_REMODELING |  | 28 | -0.31 | -1.18 | 0.231 | 0.504 | 1.000 | 2807 | tags=32%, list=21%, signal=41% |
| 127 | REGULATION\_OF\_MYELOID\_CELL\_DIFFERENTIATION |  | 19 | -0.34 | -1.18 | 0.239 | 0.502 | 1.000 | 4551 | tags=58%, list=35%, signal=89% |
| 128 | BEHAVIOR |  | 136 | -0.21 | -1.17 | 0.179 | 0.522 | 1.000 | 4566 | tags=43%, list=35%, signal=66% |
| 129 | REGULATION\_OF\_ORGANELLE\_ORGANIZATION\_AND\_BIOGENESIS |  | 35 | -0.28 | -1.16 | 0.251 | 0.526 | 1.000 | 1745 | tags=26%, list=13%, signal=30% |
| 130 | SKELETAL\_DEVELOPMENT |  | 91 | -0.22 | -1.16 | 0.202 | 0.531 | 1.000 | 3037 | tags=32%, list=23%, signal=41% |
| 131 | DEVELOPMENTAL\_MATURATION |  | 18 | -0.34 | -1.16 | 0.249 | 0.529 | 1.000 | 3121 | tags=39%, list=24%, signal=51% |
| 132 | POSITIVE\_REGULATION\_OF\_PROTEIN\_MODIFICATION\_PROCESS |  | 24 | -0.31 | -1.16 | 0.251 | 0.530 | 1.000 | 1401 | tags=25%, list=11%, signal=28% |
| 133 | VITAMIN\_METABOLIC\_PROCESS |  | 15 | -0.36 | -1.16 | 0.261 | 0.529 | 1.000 | 4083 | tags=60%, list=31%, signal=87% |
| 134 | POSITIVE\_REGULATION\_OF\_T\_CELL\_ACTIVATION |  | 20 | -0.32 | -1.16 | 0.275 | 0.526 | 1.000 | 4551 | tags=55%, list=35%, signal=84% |
| 135 | GENERATION\_OF\_PRECURSOR\_METABOLITES\_AND\_ENERGY |  | 120 | -0.21 | -1.15 | 0.184 | 0.541 | 1.000 | 3208 | tags=32%, list=25%, signal=42% |
| 136 | REGULATION\_OF\_MAP\_KINASE\_ACTIVITY |  | 56 | -0.25 | -1.15 | 0.232 | 0.539 | 1.000 | 2149 | tags=29%, list=16%, signal=34% |
| 137 | AMINO\_ACID\_CATABOLIC\_PROCESS |  | 23 | -0.31 | -1.15 | 0.258 | 0.536 | 1.000 | 2327 | tags=35%, list=18%, signal=42% |
| 138 | STRIATED\_MUSCLE\_DEVELOPMENT |  | 36 | -0.27 | -1.15 | 0.259 | 0.537 | 1.000 | 3581 | tags=44%, list=27%, signal=61% |
| 139 | REGULATION\_OF\_PROTEIN\_IMPORT\_INTO\_NUCLEUS |  | 15 | -0.35 | -1.14 | 0.270 | 0.538 | 1.000 | 1393 | tags=27%, list=11%, signal=30% |
| 140 | ACTIVATION\_OF\_MAPK\_ACTIVITY |  | 33 | -0.28 | -1.14 | 0.248 | 0.539 | 1.000 | 2149 | tags=30%, list=16%, signal=36% |
| 141 | FATTY\_ACID\_OXIDATION |  | 17 | -0.34 | -1.14 | 0.268 | 0.543 | 1.000 | 3324 | tags=47%, list=25%, signal=63% |
| 142 | POSITIVE\_REGULATION\_OF\_I\_KAPPAB\_KINASE\_NF\_KAPPAB\_CASCADE |  | 67 | -0.23 | -1.14 | 0.218 | 0.542 | 1.000 | 4081 | tags=46%, list=31%, signal=67% |
| 143 | REGULATION\_OF\_CELLULAR\_PROTEIN\_METABOLIC\_PROCESS |  | 139 | -0.20 | -1.14 | 0.197 | 0.540 | 1.000 | 2925 | tags=29%, list=22%, signal=37% |
| 144 | CYTOKINE\_BIOSYNTHETIC\_PROCESS |  | 34 | -0.28 | -1.13 | 0.268 | 0.546 | 1.000 | 2886 | tags=35%, list=22%, signal=45% |
| 145 | GLYCOPROTEIN\_BIOSYNTHETIC\_PROCESS |  | 67 | -0.23 | -1.13 | 0.236 | 0.548 | 1.000 | 3630 | tags=37%, list=28%, signal=51% |
| 146 | PROTEIN\_SECRETION |  | 28 | -0.28 | -1.13 | 0.284 | 0.547 | 1.000 | 4135 | tags=46%, list=32%, signal=68% |
| 147 | NEGATIVE\_REGULATION\_OF\_RNA\_METABOLIC\_PROCESS |  | 114 | -0.21 | -1.13 | 0.212 | 0.546 | 1.000 | 2843 | tags=29%, list=22%, signal=37% |
| 148 | MEMBRANE\_ORGANIZATION\_AND\_BIOGENESIS |  | 124 | -0.20 | -1.13 | 0.216 | 0.547 | 1.000 | 1949 | tags=23%, list=15%, signal=26% |
| 149 | NEGATIVE\_REGULATION\_OF\_CELL\_DIFFERENTIATION |  | 24 | -0.30 | -1.13 | 0.290 | 0.544 | 1.000 | 2750 | tags=29%, list=21%, signal=37% |
| 150 | GLYCEROPHOSPHOLIPID\_METABOLIC\_PROCESS |  | 39 | -0.26 | -1.12 | 0.290 | 0.543 | 1.000 | 4600 | tags=51%, list=35%, signal=79% |
| 151 | NEGATIVE\_REGULATION\_OF\_CELL\_PROLIFERATION |  | 145 | -0.20 | -1.12 | 0.188 | 0.541 | 1.000 | 2646 | tags=26%, list=20%, signal=32% |
| 152 | REGULATION\_OF\_MAPKKK\_CASCADE |  | 19 | -0.33 | -1.12 | 0.311 | 0.552 | 1.000 | 711 | tags=21%, list=5%, signal=22% |
| 153 | MYELOID\_CELL\_DIFFERENTIATION |  | 35 | -0.27 | -1.12 | 0.280 | 0.549 | 1.000 | 2932 | tags=31%, list=22%, signal=40% |
| 154 | CARBOXYLIC\_ACID\_METABOLIC\_PROCESS |  | 160 | -0.19 | -1.12 | 0.207 | 0.547 | 1.000 | 4046 | tags=39%, list=31%, signal=56% |
| 155 | ORGANIC\_ACID\_METABOLIC\_PROCESS |  | 162 | -0.19 | -1.12 | 0.212 | 0.544 | 1.000 | 4046 | tags=39%, list=31%, signal=56% |
| 156 | NEGATIVE\_REGULATION\_OF\_NUCLEOBASENUCLEOSIDENUCLEOTIDE\_AND\_NUCLEIC\_ACID\_METABOLIC\_PROCESS |  | 185 | -0.19 | -1.12 | 0.197 | 0.545 | 1.000 | 2673 | tags=27%, list=20%, signal=33% |
| 157 | CYTOKINE\_SECRETION |  | 15 | -0.35 | -1.12 | 0.316 | 0.543 | 1.000 | 3420 | tags=47%, list=26%, signal=63% |
| 158 | NEGATIVE\_REGULATION\_OF\_TRANSCRIPTION\_DNA\_DEPENDENT |  | 114 | -0.21 | -1.11 | 0.242 | 0.542 | 1.000 | 2843 | tags=29%, list=22%, signal=37% |
| 159 | NEURON\_DEVELOPMENT |  | 49 | -0.24 | -1.11 | 0.252 | 0.541 | 1.000 | 3431 | tags=33%, list=26%, signal=44% |
| 160 | POST\_TRANSLATIONAL\_PROTEIN\_MODIFICATION |  | 409 | -0.17 | -1.11 | 0.146 | 0.543 | 1.000 | 2962 | tags=26%, list=23%, signal=33% |
| 161 | AMINO\_ACID\_DERIVATIVE\_METABOLIC\_PROCESS |  | 23 | -0.30 | -1.11 | 0.291 | 0.543 | 1.000 | 4024 | tags=48%, list=31%, signal=69% |
| 162 | RESPONSE\_TO\_BIOTIC\_STIMULUS |  | 103 | -0.21 | -1.11 | 0.262 | 0.540 | 1.000 | 2948 | tags=32%, list=23%, signal=41% |
| 163 | TISSUE\_REMODELING |  | 29 | -0.28 | -1.11 | 0.299 | 0.538 | 1.000 | 2807 | tags=31%, list=21%, signal=39% |
| 164 | NEGATIVE\_REGULATION\_OF\_METABOLIC\_PROCESS |  | 232 | -0.18 | -1.10 | 0.210 | 0.548 | 1.000 | 2673 | tags=25%, list=20%, signal=31% |
| 165 | REGULATION\_OF\_DEVELOPMENTAL\_PROCESS |  | 387 | -0.17 | -1.10 | 0.174 | 0.553 | 1.000 | 4456 | tags=43%, list=34%, signal=64% |
| 166 | ION\_HOMEOSTASIS |  | 112 | -0.20 | -1.10 | 0.257 | 0.553 | 1.000 | 3218 | tags=32%, list=25%, signal=42% |
| 167 | TISSUE\_DEVELOPMENT |  | 126 | -0.20 | -1.10 | 0.255 | 0.553 | 1.000 | 1529 | tags=19%, list=12%, signal=21% |
| 168 | DEFENSE\_RESPONSE\_TO\_BACTERIUM |  | 16 | -0.33 | -1.10 | 0.343 | 0.554 | 1.000 | 4086 | tags=44%, list=31%, signal=64% |
| 169 | SODIUM\_ION\_TRANSPORT |  | 17 | -0.33 | -1.10 | 0.324 | 0.551 | 1.000 | 5088 | tags=59%, list=39%, signal=96% |
| 170 | AMINO\_ACID\_METABOLIC\_PROCESS |  | 73 | -0.22 | -1.09 | 0.282 | 0.553 | 1.000 | 2327 | tags=27%, list=18%, signal=33% |
| 171 | PHAGOCYTOSIS |  | 16 | -0.33 | -1.09 | 0.315 | 0.551 | 1.000 | 4171 | tags=56%, list=32%, signal=82% |
| 172 | AMINE\_CATABOLIC\_PROCESS |  | 25 | -0.29 | -1.09 | 0.323 | 0.549 | 1.000 | 2327 | tags=32%, list=18%, signal=39% |
| 173 | CELLULAR\_LIPID\_METABOLIC\_PROCESS |  | 220 | -0.18 | -1.09 | 0.219 | 0.551 | 1.000 | 3897 | tags=38%, list=30%, signal=53% |
| 174 | CELL\_MATURATION |  | 16 | -0.33 | -1.09 | 0.349 | 0.551 | 1.000 | 3121 | tags=38%, list=24%, signal=49% |
| 175 | I\_KAPPAB\_KINASE\_NF\_KAPPAB\_CASCADE |  | 88 | -0.21 | -1.09 | 0.278 | 0.553 | 1.000 | 3726 | tags=40%, list=28%, signal=55% |
| 176 | ACTIN\_FILAMENT\_ORGANIZATION |  | 21 | -0.29 | -1.08 | 0.354 | 0.559 | 1.000 | 1745 | tags=29%, list=13%, signal=33% |
| 177 | LOCOMOTORY\_BEHAVIOR |  | 84 | -0.21 | -1.08 | 0.309 | 0.563 | 1.000 | 2765 | tags=29%, list=21%, signal=36% |
| 178 | ELECTRON\_TRANSPORT\_GO\_0006118 |  | 50 | -0.24 | -1.08 | 0.319 | 0.573 | 1.000 | 2233 | tags=26%, list=17%, signal=31% |
| 179 | CYTOKINE\_METABOLIC\_PROCESS |  | 35 | -0.26 | -1.07 | 0.334 | 0.579 | 1.000 | 2886 | tags=34%, list=22%, signal=44% |
| 180 | POSITIVE\_REGULATION\_OF\_DEVELOPMENTAL\_PROCESS |  | 197 | -0.18 | -1.07 | 0.297 | 0.579 | 1.000 | 4364 | tags=45%, list=33%, signal=67% |
| 181 | MEMBRANE\_LIPID\_METABOLIC\_PROCESS |  | 85 | -0.21 | -1.07 | 0.322 | 0.577 | 1.000 | 3769 | tags=39%, list=29%, signal=54% |
| 182 | NEURITE\_DEVELOPMENT |  | 41 | -0.24 | -1.07 | 0.347 | 0.583 | 1.000 | 3431 | tags=32%, list=26%, signal=43% |
| 183 | REGULATION\_OF\_BIOLOGICAL\_QUALITY |  | 364 | -0.17 | -1.07 | 0.261 | 0.580 | 1.000 | 3631 | tags=30%, list=28%, signal=41% |
| 184 | NITROGEN\_COMPOUND\_CATABOLIC\_PROCESS |  | 27 | -0.28 | -1.07 | 0.338 | 0.582 | 1.000 | 2327 | tags=30%, list=18%, signal=36% |
| 185 | NEGATIVE\_REGULATION\_OF\_CELLULAR\_METABOLIC\_PROCESS |  | 229 | -0.17 | -1.06 | 0.274 | 0.582 | 1.000 | 2673 | tags=25%, list=20%, signal=31% |
| 186 | DETECTION\_OF\_EXTERNAL\_STIMULUS |  | 18 | -0.31 | -1.06 | 0.373 | 0.579 | 1.000 | 8986 | tags=100%, list=69%, signal=318% |
| 187 | POSITIVE\_REGULATION\_OF\_TRANSCRIPTION |  | 124 | -0.19 | -1.06 | 0.306 | 0.580 | 1.000 | 3342 | tags=31%, list=26%, signal=41% |
| 188 | HORMONE\_METABOLIC\_PROCESS |  | 29 | -0.27 | -1.06 | 0.347 | 0.589 | 1.000 | 3897 | tags=48%, list=30%, signal=69% |
| 189 | NEGATIVE\_REGULATION\_OF\_TRANSCRIPTION\_FROM\_RNA\_POLYMERASE\_II\_PROMOTER |  | 76 | -0.21 | -1.05 | 0.358 | 0.604 | 1.000 | 2843 | tags=29%, list=22%, signal=37% |
| 190 | POSITIVE\_REGULATION\_OF\_MAP\_KINASE\_ACTIVITY |  | 39 | -0.25 | -1.05 | 0.368 | 0.603 | 1.000 | 2149 | tags=28%, list=16%, signal=34% |
| 191 | AXONOGENESIS |  | 33 | -0.26 | -1.05 | 0.397 | 0.601 | 1.000 | 3431 | tags=33%, list=26%, signal=45% |
| 192 | CELL\_PROLIFERATION\_GO\_0008283 |  | 466 | -0.16 | -1.05 | 0.288 | 0.602 | 1.000 | 2646 | tags=24%, list=20%, signal=29% |
| 193 | POSITIVE\_REGULATION\_OF\_CELLULAR\_METABOLIC\_PROCESS |  | 196 | -0.18 | -1.05 | 0.321 | 0.600 | 1.000 | 2275 | tags=23%, list=17%, signal=27% |
| 194 | MYOBLAST\_DIFFERENTIATION |  | 16 | -0.31 | -1.05 | 0.385 | 0.598 | 1.000 | 3411 | tags=50%, list=26%, signal=68% |
| 195 | CELL\_MIGRATION |  | 82 | -0.21 | -1.04 | 0.368 | 0.607 | 1.000 | 2803 | tags=26%, list=21%, signal=32% |
| 196 | POSITIVE\_REGULATION\_OF\_METABOLIC\_PROCESS |  | 201 | -0.17 | -1.04 | 0.374 | 0.620 | 1.000 | 2310 | tags=23%, list=18%, signal=27% |
| 197 | MAPKKK\_CASCADE\_GO\_0000165 |  | 90 | -0.20 | -1.03 | 0.395 | 0.623 | 1.000 | 2149 | tags=22%, list=16%, signal=26% |
| 198 | TRANSLATION |  | 149 | -0.18 | -1.03 | 0.398 | 0.623 | 1.000 | 2886 | tags=29%, list=22%, signal=37% |
| 199 | AMINO\_ACID\_AND\_DERIVATIVE\_METABOLIC\_PROCESS |  | 96 | -0.19 | -1.03 | 0.376 | 0.623 | 1.000 | 2327 | tags=25%, list=18%, signal=30% |
| 200 | DETECTION\_OF\_STIMULUS\_INVOLVED\_IN\_SENSORY\_PERCEPTION |  | 15 | -0.31 | -1.03 | 0.430 | 0.625 | 1.000 | 8986 | tags=100%, list=69%, signal=319% |
| 201 | HEART\_DEVELOPMENT |  | 33 | -0.25 | -1.03 | 0.407 | 0.634 | 1.000 | 4012 | tags=39%, list=31%, signal=57% |
| 202 | PEPTIDYL\_AMINO\_ACID\_MODIFICATION |  | 47 | -0.23 | -1.02 | 0.398 | 0.637 | 1.000 | 2625 | tags=28%, list=20%, signal=34% |
| 203 | ORGANIC\_ACID\_TRANSPORT |  | 39 | -0.24 | -1.02 | 0.392 | 0.643 | 1.000 | 2202 | tags=26%, list=17%, signal=31% |
| 204 | POSITIVE\_REGULATION\_OF\_CATALYTIC\_ACTIVITY |  | 139 | -0.18 | -1.02 | 0.416 | 0.643 | 1.000 | 2202 | tags=21%, list=17%, signal=25% |
| 205 | CARBOXYLIC\_ACID\_TRANSPORT |  | 39 | -0.24 | -1.02 | 0.424 | 0.648 | 1.000 | 2202 | tags=26%, list=17%, signal=31% |
| 206 | NERVOUS\_SYSTEM\_DEVELOPMENT |  | 328 | -0.16 | -1.01 | 0.420 | 0.654 | 1.000 | 3936 | tags=32%, list=30%, signal=45% |
| 207 | AXON\_GUIDANCE |  | 18 | -0.30 | -1.01 | 0.422 | 0.653 | 1.000 | 2803 | tags=33%, list=21%, signal=42% |
| 208 | ANATOMICAL\_STRUCTURE\_MORPHOGENESIS |  | 336 | -0.16 | -1.01 | 0.435 | 0.663 | 1.000 | 3431 | tags=29%, list=26%, signal=39% |
| 209 | REGULATION\_OF\_TRANSCRIPTION |  | 498 | -0.15 | -1.01 | 0.432 | 0.662 | 1.000 | 3356 | tags=29%, list=26%, signal=38% |
| 210 | CELLULAR\_COMPONENT\_ASSEMBLY |  | 272 | -0.16 | -1.00 | 0.422 | 0.661 | 1.000 | 3137 | tags=28%, list=24%, signal=36% |
| 211 | NEUROGENESIS |  | 75 | -0.20 | -1.00 | 0.474 | 0.683 | 1.000 | 3659 | tags=33%, list=28%, signal=46% |
| 212 | MACROMOLECULE\_BIOSYNTHETIC\_PROCESS |  | 267 | -0.16 | -0.99 | 0.479 | 0.682 | 1.000 | 3662 | tags=33%, list=28%, signal=44% |
| 213 | REGULATION\_OF\_JNK\_ACTIVITY |  | 18 | -0.29 | -0.99 | 0.461 | 0.689 | 1.000 | 1979 | tags=28%, list=15%, signal=33% |
| 214 | RESPONSE\_TO\_CHEMICAL\_STIMULUS |  | 271 | -0.16 | -0.99 | 0.474 | 0.695 | 1.000 | 1871 | tags=18%, list=14%, signal=21% |
| 215 | POSITIVE\_REGULATION\_OF\_DNA\_BINDING |  | 18 | -0.29 | -0.99 | 0.465 | 0.694 | 1.000 | 4446 | tags=56%, list=34%, signal=84% |
| 216 | POSITIVE\_REGULATION\_OF\_TRANSCRIPTION\_FACTOR\_ACTIVITY |  | 17 | -0.29 | -0.99 | 0.469 | 0.691 | 1.000 | 4236 | tags=53%, list=32%, signal=78% |
| 217 | REGULATION\_OF\_PROTEIN\_SECRETION |  | 19 | -0.29 | -0.99 | 0.475 | 0.688 | 1.000 | 4070 | tags=47%, list=31%, signal=69% |
| 218 | CELL\_CELL\_ADHESION |  | 72 | -0.20 | -0.98 | 0.475 | 0.690 | 1.000 | 4236 | tags=42%, list=32%, signal=61% |
| 219 | REGULATION\_OF\_CYTOKINE\_BIOSYNTHETIC\_PROCESS |  | 31 | -0.24 | -0.98 | 0.460 | 0.691 | 1.000 | 2886 | tags=32%, list=22%, signal=41% |
| 220 | POSITIVE\_REGULATION\_OF\_TRANSCRIPTION\_FROM\_RNA\_POLYMERASE\_II\_PROMOTER |  | 60 | -0.21 | -0.98 | 0.482 | 0.698 | 1.000 | 3000 | tags=30%, list=23%, signal=39% |
| 221 | ANTI\_APOPTOSIS |  | 107 | -0.18 | -0.98 | 0.477 | 0.697 | 1.000 | 2250 | tags=25%, list=17%, signal=30% |
| 222 | RHYTHMIC\_PROCESS |  | 23 | -0.26 | -0.97 | 0.511 | 0.714 | 1.000 | 2076 | tags=26%, list=16%, signal=31% |
| 223 | POSITIVE\_REGULATION\_OF\_NUCLEOBASENUCLEOSIDENUCLEOTIDE\_AND\_NUCLEIC\_ACID\_METABOLIC\_PROCESS |  | 134 | -0.17 | -0.97 | 0.509 | 0.722 | 1.000 | 3342 | tags=30%, list=26%, signal=40% |
| 224 | NEGATIVE\_REGULATION\_OF\_DEVELOPMENTAL\_PROCESS |  | 177 | -0.17 | -0.95 | 0.556 | 0.754 | 1.000 | 2807 | tags=27%, list=21%, signal=33% |
| 225 | EPIDERMIS\_DEVELOPMENT |  | 66 | -0.20 | -0.95 | 0.544 | 0.758 | 1.000 | 1529 | tags=20%, list=12%, signal=22% |
| 226 | CELLULAR\_HOMEOSTASIS |  | 121 | -0.17 | -0.95 | 0.575 | 0.757 | 1.000 | 4334 | tags=41%, list=33%, signal=61% |
| 227 | MACROMOLECULAR\_COMPLEX\_ASSEMBLY |  | 254 | -0.15 | -0.95 | 0.607 | 0.755 | 1.000 | 3137 | tags=28%, list=24%, signal=36% |
| 228 | CHEMICAL\_HOMEOSTASIS |  | 136 | -0.17 | -0.95 | 0.562 | 0.759 | 1.000 | 3218 | tags=29%, list=25%, signal=38% |
| 229 | REGULATION\_OF\_G\_PROTEIN\_COUPLED\_RECEPTOR\_PROTEIN\_SIGNALING\_PATHWAY |  | 23 | -0.26 | -0.94 | 0.537 | 0.767 | 1.000 | 1067 | tags=17%, list=8%, signal=19% |
| 230 | INSULIN\_RECEPTOR\_SIGNALING\_PATHWAY |  | 16 | -0.28 | -0.94 | 0.514 | 0.769 | 1.000 | 3763 | tags=44%, list=29%, signal=61% |
| 231 | SECRETION\_BY\_CELL |  | 100 | -0.18 | -0.94 | 0.589 | 0.774 | 1.000 | 4420 | tags=42%, list=34%, signal=63% |
| 232 | CYCLIC\_NUCLEOTIDE\_MEDIATED\_SIGNALING |  | 97 | -0.18 | -0.93 | 0.583 | 0.779 | 1.000 | 1272 | tags=12%, list=10%, signal=14% |
| 233 | CELLULAR\_PROTEIN\_COMPLEX\_ASSEMBLY |  | 28 | -0.24 | -0.93 | 0.559 | 0.782 | 1.000 | 2775 | tags=29%, list=21%, signal=36% |
| 234 | SKELETAL\_MUSCLE\_DEVELOPMENT |  | 28 | -0.24 | -0.93 | 0.566 | 0.779 | 1.000 | 3581 | tags=43%, list=27%, signal=59% |
| 235 | RESPONSE\_TO\_OXIDATIVE\_STRESS |  | 38 | -0.22 | -0.93 | 0.574 | 0.778 | 1.000 | 1466 | tags=24%, list=11%, signal=27% |
| 236 | HOMEOSTATIC\_PROCESS |  | 179 | -0.16 | -0.93 | 0.660 | 0.786 | 1.000 | 3301 | tags=28%, list=25%, signal=38% |
| 237 | ECTODERM\_DEVELOPMENT |  | 75 | -0.18 | -0.92 | 0.598 | 0.799 | 1.000 | 1529 | tags=19%, list=12%, signal=21% |
| 238 | G\_PROTEIN\_SIGNALING\_COUPLED\_TO\_CYCLIC\_NUCLEOTIDE\_SECOND\_MESSENGER |  | 96 | -0.18 | -0.91 | 0.663 | 0.814 | 1.000 | 1272 | tags=13%, list=10%, signal=14% |
| 239 | RESPONSE\_TO\_NUTRIENT |  | 17 | -0.27 | -0.91 | 0.574 | 0.823 | 1.000 | 397 | tags=18%, list=3%, signal=18% |
| 240 | ACTIVATION\_OF\_PROTEIN\_KINASE\_ACTIVITY |  | 23 | -0.24 | -0.89 | 0.564 | 0.859 | 1.000 | 4449 | tags=39%, list=34%, signal=59% |
| 241 | REGULATION\_OF\_MUSCLE\_CONTRACTION |  | 18 | -0.26 | -0.89 | 0.584 | 0.857 | 1.000 | 2973 | tags=39%, list=23%, signal=50% |
| 242 | ENDOSOME\_TRANSPORT |  | 22 | -0.24 | -0.89 | 0.610 | 0.857 | 1.000 | 2254 | tags=27%, list=17%, signal=33% |
| 243 | AMINE\_METABOLIC\_PROCESS |  | 128 | -0.16 | -0.89 | 0.712 | 0.860 | 1.000 | 4024 | tags=36%, list=31%, signal=51% |
| 244 | REGULATION\_OF\_CELLULAR\_COMPONENT\_ORGANIZATION\_AND\_BIOGENESIS |  | 102 | -0.17 | -0.89 | 0.737 | 0.862 | 1.000 | 3605 | tags=32%, list=28%, signal=44% |
| 245 | REGULATION\_OF\_TRANSLATION |  | 76 | -0.18 | -0.88 | 0.725 | 0.872 | 1.000 | 2886 | tags=28%, list=22%, signal=35% |
| 246 | CELL\_CELL\_SIGNALING |  | 372 | -0.14 | -0.88 | 0.875 | 0.879 | 1.000 | 4142 | tags=33%, list=32%, signal=47% |
| 247 | REGULATION\_OF\_TRANSLATIONAL\_INITIATION |  | 25 | -0.23 | -0.87 | 0.648 | 0.879 | 1.000 | 2823 | tags=32%, list=22%, signal=41% |
| 248 | REGULATION\_OF\_GROWTH |  | 48 | -0.20 | -0.87 | 0.687 | 0.886 | 1.000 | 4632 | tags=46%, list=35%, signal=71% |
| 249 | NEGATIVE\_REGULATION\_OF\_CELLULAR\_COMPONENT\_ORGANIZATION\_AND\_BIOGENESIS |  | 26 | -0.23 | -0.87 | 0.663 | 0.883 | 1.000 | 1745 | tags=19%, list=13%, signal=22% |
| 250 | SPHINGOLIPID\_METABOLIC\_PROCESS |  | 23 | -0.23 | -0.86 | 0.683 | 0.895 | 1.000 | 3240 | tags=35%, list=25%, signal=46% |
| 251 | PHOSPHOINOSITIDE\_METABOLIC\_PROCESS |  | 25 | -0.23 | -0.86 | 0.673 | 0.895 | 1.000 | 4807 | tags=56%, list=37%, signal=88% |
| 252 | CELLULAR\_MORPHOGENESIS\_DURING\_DIFFERENTIATION |  | 38 | -0.20 | -0.86 | 0.684 | 0.892 | 1.000 | 3431 | tags=29%, list=26%, signal=39% |
| 253 | REGULATION\_OF\_PROTEIN\_MODIFICATION\_PROCESS |  | 37 | -0.21 | -0.86 | 0.676 | 0.894 | 1.000 | 1568 | tags=19%, list=12%, signal=21% |
| 254 | POSITIVE\_REGULATION\_OF\_CELLULAR\_COMPONENT\_ORGANIZATION\_AND\_BIOGENESIS |  | 28 | -0.22 | -0.86 | 0.695 | 0.897 | 1.000 | 3505 | tags=36%, list=27%, signal=49% |
| 255 | PROTEIN\_HOMOOLIGOMERIZATION |  | 19 | -0.25 | -0.85 | 0.652 | 0.904 | 1.000 | 2199 | tags=26%, list=17%, signal=32% |
| 256 | REGULATION\_OF\_BINDING |  | 46 | -0.19 | -0.85 | 0.706 | 0.901 | 1.000 | 1810 | tags=22%, list=14%, signal=25% |
| 257 | VESICLE\_MEDIATED\_TRANSPORT |  | 174 | -0.14 | -0.85 | 0.863 | 0.905 | 1.000 | 1949 | tags=18%, list=15%, signal=21% |
| 258 | SECOND\_MESSENGER\_MEDIATED\_SIGNALING |  | 139 | -0.15 | -0.85 | 0.857 | 0.903 | 1.000 | 1348 | tags=12%, list=10%, signal=13% |
| 259 | CENTRAL\_NERVOUS\_SYSTEM\_DEVELOPMENT |  | 105 | -0.16 | -0.83 | 0.856 | 0.940 | 1.000 | 3823 | tags=32%, list=29%, signal=45% |
| 260 | RESPONSE\_TO\_NUTRIENT\_LEVELS |  | 27 | -0.21 | -0.83 | 0.719 | 0.938 | 1.000 | 4334 | tags=44%, list=33%, signal=66% |
| 261 | POSITIVE\_REGULATION\_OF\_BINDING |  | 19 | -0.24 | -0.82 | 0.695 | 0.944 | 1.000 | 4446 | tags=53%, list=34%, signal=80% |
| 262 | SULFUR\_METABOLIC\_PROCESS |  | 30 | -0.21 | -0.81 | 0.756 | 0.951 | 1.000 | 2964 | tags=30%, list=23%, signal=39% |
| 263 | EXTRACELLULAR\_STRUCTURE\_ORGANIZATION\_AND\_BIOGENESIS |  | 23 | -0.21 | -0.79 | 0.738 | 0.982 | 1.000 | 3581 | tags=39%, list=27%, signal=54% |
| 264 | NEGATIVE\_REGULATION\_OF\_GROWTH |  | 35 | -0.19 | -0.79 | 0.801 | 0.979 | 1.000 | 4632 | tags=49%, list=35%, signal=75% |
| 265 | POSITIVE\_REGULATION\_OF\_CASPASE\_ACTIVITY |  | 28 | -0.20 | -0.79 | 0.761 | 0.977 | 1.000 | 1936 | tags=25%, list=15%, signal=29% |
| 266 | PATTERN\_SPECIFICATION\_PROCESS |  | 27 | -0.20 | -0.79 | 0.788 | 0.976 | 1.000 | 5556 | tags=63%, list=42%, signal=109% |
| 267 | POSITIVE\_REGULATION\_OF\_JNK\_ACTIVITY |  | 16 | -0.23 | -0.79 | 0.754 | 0.982 | 1.000 | 1979 | tags=25%, list=15%, signal=29% |
| 268 | REPRODUCTIVE\_PROCESS |  | 133 | -0.14 | -0.78 | 0.930 | 0.986 | 1.000 | 3940 | tags=34%, list=30%, signal=48% |
| 269 | T\_CELL\_PROLIFERATION |  | 17 | -0.23 | -0.78 | 0.766 | 0.983 | 1.000 | 4830 | tags=59%, list=37%, signal=93% |
| 270 | POSITIVE\_REGULATION\_OF\_TRANSCRIPTIONDNA\_DEPENDENT |  | 105 | -0.14 | -0.78 | 0.935 | 0.984 | 1.000 | 4644 | tags=41%, list=35%, signal=63% |
| 271 | EXCRETION |  | 35 | -0.19 | -0.78 | 0.829 | 0.983 | 1.000 | 2101 | tags=20%, list=16%, signal=24% |
| 272 | REGULATION\_OF\_TRANSCRIPTION\_FACTOR\_ACTIVITY |  | 30 | -0.19 | -0.77 | 0.820 | 0.982 | 1.000 | 4468 | tags=47%, list=34%, signal=71% |
| 273 | G\_PROTEIN\_COUPLED\_RECEPTOR\_PROTEIN\_SIGNALING\_PATHWAY |  | 300 | -0.12 | -0.77 | 0.992 | 0.980 | 1.000 | 4635 | tags=35%, list=35%, signal=52% |
| 274 | GOLGI\_VESICLE\_TRANSPORT |  | 42 | -0.18 | -0.77 | 0.838 | 0.978 | 1.000 | 4420 | tags=43%, list=34%, signal=64% |
| 275 | AMINE\_BIOSYNTHETIC\_PROCESS |  | 15 | -0.24 | -0.77 | 0.766 | 0.985 | 1.000 | 462 | tags=13%, list=4%, signal=14% |
| 276 | REGULATION\_OF\_CELL\_MIGRATION |  | 23 | -0.21 | -0.76 | 0.801 | 0.982 | 1.000 | 5108 | tags=48%, list=39%, signal=78% |
| 277 | REGULATION\_OF\_DNA\_BINDING |  | 36 | -0.18 | -0.76 | 0.824 | 0.978 | 1.000 | 4468 | tags=47%, list=34%, signal=71% |
| 278 | TRANSLATIONAL\_INITIATION |  | 33 | -0.19 | -0.76 | 0.840 | 0.978 | 1.000 | 1266 | tags=18%, list=10%, signal=20% |
| 279 | G\_PROTEIN\_SIGNALING\_COUPLED\_TO\_IP3\_SECOND\_MESSENGERPHOSPHOLIPASE\_C\_ACTIVATING |  | 39 | -0.18 | -0.76 | 0.876 | 0.978 | 1.000 | 2651 | tags=23%, list=20%, signal=29% |
| 280 | METAL\_ION\_TRANSPORT |  | 102 | -0.14 | -0.75 | 0.944 | 0.984 | 1.000 | 5349 | tags=49%, list=41%, signal=82% |
| 281 | REGULATION\_OF\_SECRETION |  | 35 | -0.18 | -0.75 | 0.851 | 0.986 | 1.000 | 4357 | tags=46%, list=33%, signal=68% |
| 282 | RESPONSE\_TO\_EXTRACELLULAR\_STIMULUS |  | 29 | -0.19 | -0.75 | 0.881 | 0.986 | 1.000 | 2432 | tags=24%, list=19%, signal=30% |
| 283 | SECRETION |  | 157 | -0.13 | -0.74 | 0.978 | 0.990 | 1.000 | 4424 | tags=38%, list=34%, signal=56% |
| 284 | CARBOHYDRATE\_METABOLIC\_PROCESS |  | 152 | -0.13 | -0.74 | 0.975 | 0.991 | 1.000 | 3855 | tags=31%, list=29%, signal=43% |
| 285 | POSITIVE\_REGULATION\_OF\_RNA\_METABOLIC\_PROCESS |  | 107 | -0.14 | -0.73 | 0.965 | 0.995 | 1.000 | 4644 | tags=40%, list=35%, signal=62% |
| 286 | DI\_\_\_TRI\_VALENT\_INORGANIC\_CATION\_TRANSPORT |  | 27 | -0.18 | -0.72 | 0.886 | 0.995 | 1.000 | 1109 | tags=15%, list=8%, signal=16% |
| 287 | ESTABLISHMENT\_AND\_OR\_MAINTENANCE\_OF\_CELL\_POLARITY |  | 19 | -0.21 | -0.72 | 0.862 | 0.998 | 1.000 | 3776 | tags=32%, list=29%, signal=44% |
| 288 | PROTEIN\_POLYMERIZATION |  | 17 | -0.21 | -0.71 | 0.858 | 0.999 | 1.000 | 269 | tags=12%, list=2%, signal=12% |
| 289 | POTASSIUM\_ION\_TRANSPORT |  | 52 | -0.15 | -0.71 | 0.928 | 0.998 | 1.000 | 5349 | tags=50%, list=41%, signal=84% |
| 290 | LIPID\_HOMEOSTASIS |  | 15 | -0.22 | -0.71 | 0.845 | 0.998 | 1.000 | 632 | tags=13%, list=5%, signal=14% |
| 291 | G\_PROTEIN\_SIGNALING\_ADENYLATE\_CYCLASE\_ACTIVATING\_PATHWAY |  | 24 | -0.19 | -0.71 | 0.870 | 0.995 | 1.000 | 1272 | tags=13%, list=10%, signal=14% |
| 292 | PROTEIN\_AMINO\_ACID\_LIPIDATION |  | 21 | -0.19 | -0.70 | 0.877 | 0.996 | 1.000 | 4600 | tags=52%, list=35%, signal=81% |
| 293 | SECRETORY\_PATHWAY |  | 72 | -0.14 | -0.70 | 0.953 | 0.995 | 1.000 | 4420 | tags=40%, list=34%, signal=60% |
| 294 | NUCLEOTIDE\_EXCISION\_REPAIR |  | 19 | -0.19 | -0.69 | 0.916 | 1.000 | 1.000 | 2051 | tags=21%, list=16%, signal=25% |
| 295 | NEGATIVE\_REGULATION\_OF\_CELLULAR\_PROTEIN\_METABOLIC\_PROCESS |  | 41 | -0.15 | -0.68 | 0.952 | 1.000 | 1.000 | 2925 | tags=24%, list=22%, signal=31% |
| 296 | INORGANIC\_ANION\_TRANSPORT |  | 16 | -0.20 | -0.68 | 0.887 | 1.000 | 1.000 | 606 | tags=13%, list=5%, signal=13% |
| 297 | CATION\_TRANSPORT |  | 130 | -0.12 | -0.67 | 0.991 | 1.000 | 1.000 | 5230 | tags=45%, list=40%, signal=75% |
| 298 | PHOSPHOINOSITIDE\_MEDIATED\_SIGNALING |  | 42 | -0.15 | -0.66 | 0.940 | 1.000 | 1.000 | 2651 | tags=21%, list=20%, signal=27% |
| 299 | REGULATION\_OF\_CYTOKINE\_PRODUCTION |  | 21 | -0.18 | -0.64 | 0.926 | 1.000 | 1.000 | 2480 | tags=24%, list=19%, signal=29% |
| 300 | ION\_TRANSPORT |  | 165 | -0.11 | -0.64 | 1.000 | 1.000 | 1.000 | 5258 | tags=45%, list=40%, signal=74% |
| 301 | NEGATIVE\_REGULATION\_OF\_MULTICELLULAR\_ORGANISMAL\_PROCESS |  | 27 | -0.17 | -0.64 | 0.929 | 1.000 | 1.000 | 2235 | tags=22%, list=17%, signal=27% |
| 302 | NEGATIVE\_REGULATION\_OF\_PROTEIN\_METABOLIC\_PROCESS |  | 44 | -0.14 | -0.64 | 0.965 | 1.000 | 1.000 | 2925 | tags=23%, list=22%, signal=29% |
| 303 | CARBOHYDRATE\_BIOSYNTHETIC\_PROCESS |  | 35 | -0.15 | -0.64 | 0.961 | 1.000 | 1.000 | 5288 | tags=51%, list=40%, signal=86% |
| 304 | POSITIVE\_REGULATION\_OF\_TRANSPORT |  | 18 | -0.18 | -0.63 | 0.933 | 1.000 | 1.000 | 5086 | tags=56%, list=39%, signal=91% |
| 305 | REGULATION\_OF\_CELL\_GROWTH |  | 39 | -0.14 | -0.62 | 0.983 | 1.000 | 1.000 | 4632 | tags=41%, list=35%, signal=63% |
| 306 | CARBOHYDRATE\_CATABOLIC\_PROCESS |  | 20 | -0.17 | -0.61 | 0.953 | 1.000 | 1.000 | 4339 | tags=35%, list=33%, signal=52% |
| 307 | CELLULAR\_CARBOHYDRATE\_CATABOLIC\_PROCESS |  | 20 | -0.17 | -0.61 | 0.942 | 1.000 | 1.000 | 4339 | tags=35%, list=33%, signal=52% |
| 308 | RHO\_PROTEIN\_SIGNAL\_TRANSDUCTION |  | 30 | -0.15 | -0.59 | 0.970 | 1.000 | 1.000 | 3876 | tags=33%, list=30%, signal=47% |
| 309 | CALCIUM\_ION\_TRANSPORT |  | 23 | -0.16 | -0.58 | 0.962 | 1.000 | 1.000 | 1109 | tags=13%, list=8%, signal=14% |
| 310 | FEMALE\_GAMETE\_GENERATION |  | 15 | -0.18 | -0.56 | 0.972 | 1.000 | 1.000 | 10786 | tags=100%, list=82%, signal=567% |
| 311 | REGULATION\_OF\_HEART\_CONTRACTION |  | 24 | -0.15 | -0.56 | 0.990 | 1.000 | 1.000 | 11125 | tags=100%, list=85%, signal=665% |
| 312 | AMINO\_SUGAR\_METABOLIC\_PROCESS |  | 15 | -0.17 | -0.55 | 0.983 | 1.000 | 1.000 | 3711 | tags=33%, list=28%, signal=46% |
| 313 | REGULATION\_OF\_ACTION\_POTENTIAL |  | 16 | -0.16 | -0.53 | 0.986 | 1.000 | 1.000 | 3600 | tags=31%, list=27%, signal=43% |
| 314 | PEROXISOME\_ORGANIZATION\_AND\_BIOGENESIS |  | 15 | -0.17 | -0.52 | 0.978 | 0.998 | 1.000 | 4888 | tags=47%, list=37%, signal=74% |
| 315 | MONOVALENT\_INORGANIC\_CATION\_TRANSPORT |  | 83 | -0.10 | -0.51 | 1.000 | 0.996 | 1.000 | 5349 | tags=46%, list=41%, signal=77% |
Table: Gene sets enriched in phenotype **na**[plain text format]****

  
