# Supplementary material for: Multiple-input multiple-output causal strategies for gene selection
Source: BMC Bioinformatics. 2011 Nov 25;12:458. doi: 10.1186/1471-2105-12-458 (PMC3323860; doi:10.1186/1471-2105-12-458)
Supplement: Additional file 2 — Archive containing the output files computed by the preranked GSEA for λ ∈ {0.1,0.2,0.3,0.4,0.5} (GSEA_MIMO_part1.zip). [file 1471-2105-12-458-S2.ZIP › mFS03_entrez_mimo.GseaPreranked.1316038204362/gsea_report_for_na_pos_1316038204362.html]

Report for na\_pos 1316038204362 [GSEA]

| GS  follow link to MSigDB | GS DETAILS | SIZE | ES | NES | NOM p-val | FDR q-val | FWER p-val | RANK AT MAX | LEADING EDGE || 1 | M\_PHASE\_OF\_MITOTIC\_CELL\_CYCLE |  | 72 | 0.59 | 2.78 | 0.000 | 0.000 | 0.000 | 2133 | tags=56%, list=16%, signal=66% |
| 2 | M\_PHASE |  | 98 | 0.55 | 2.78 | 0.000 | 0.000 | 0.000 | 2133 | tags=51%, list=16%, signal=60% |
| 3 | MITOSIS |  | 70 | 0.58 | 2.76 | 0.000 | 0.000 | 0.000 | 2133 | tags=54%, list=16%, signal=65% |
| 4 | MITOTIC\_CELL\_CYCLE |  | 134 | 0.51 | 2.75 | 0.000 | 0.000 | 0.000 | 2724 | tags=52%, list=21%, signal=65% |
| 5 | CELL\_CYCLE\_PROCESS |  | 169 | 0.49 | 2.73 | 0.000 | 0.000 | 0.000 | 2724 | tags=50%, list=21%, signal=63% |
| 6 | CELL\_CYCLE\_PHASE |  | 152 | 0.48 | 2.60 | 0.000 | 0.000 | 0.000 | 2724 | tags=49%, list=21%, signal=61% |
| 7 | DNA\_REPLICATION |  | 97 | 0.50 | 2.53 | 0.000 | 0.000 | 0.000 | 2697 | tags=47%, list=21%, signal=59% |
| 8 | SISTER\_CHROMATID\_SEGREGATION |  | 16 | 0.76 | 2.46 | 0.000 | 0.000 | 0.001 | 664 | tags=56%, list=5%, signal=59% |
| 9 | MITOTIC\_SISTER\_CHROMATID\_SEGREGATION |  | 15 | 0.78 | 2.46 | 0.000 | 0.000 | 0.001 | 664 | tags=60%, list=5%, signal=63% |
| 10 | DNA\_METABOLIC\_PROCESS |  | 240 | 0.42 | 2.44 | 0.000 | 0.000 | 0.001 | 2697 | tags=43%, list=21%, signal=54% |
| 11 | CELL\_CYCLE\_GO\_0007049 |  | 277 | 0.41 | 2.42 | 0.000 | 0.000 | 0.001 | 2724 | tags=43%, list=21%, signal=54% |
| 12 | CHROMOSOME\_SEGREGATION |  | 28 | 0.62 | 2.39 | 0.000 | 0.000 | 0.001 | 664 | tags=46%, list=5%, signal=49% |
| 13 | CELL\_CYCLE\_CHECKPOINT\_GO\_0000075 |  | 45 | 0.53 | 2.33 | 0.000 | 0.000 | 0.001 | 2544 | tags=58%, list=19%, signal=71% |
| 14 | DNA\_DEPENDENT\_DNA\_REPLICATION |  | 52 | 0.52 | 2.33 | 0.000 | 0.000 | 0.001 | 2836 | tags=54%, list=22%, signal=68% |
| 15 | DNA\_REPAIR |  | 118 | 0.44 | 2.31 | 0.000 | 0.000 | 0.001 | 2595 | tags=46%, list=20%, signal=57% |
| 16 | REGULATION\_OF\_MITOSIS |  | 33 | 0.58 | 2.31 | 0.000 | 0.000 | 0.001 | 1865 | tags=52%, list=14%, signal=60% |
| 17 | RESPONSE\_TO\_DNA\_DAMAGE\_STIMULUS |  | 153 | 0.42 | 2.28 | 0.000 | 0.000 | 0.003 | 2595 | tags=44%, list=20%, signal=54% |
| 18 | RNA\_SPLICING |  | 74 | 0.46 | 2.21 | 0.000 | 0.000 | 0.009 | 3662 | tags=55%, list=28%, signal=76% |
| 19 | RESPONSE\_TO\_ENDOGENOUS\_STIMULUS |  | 182 | 0.38 | 2.16 | 0.000 | 0.001 | 0.019 | 3287 | tags=46%, list=25%, signal=60% |
| 20 | NUCLEOTIDE\_BIOSYNTHETIC\_PROCESS |  | 17 | 0.65 | 2.15 | 0.000 | 0.001 | 0.025 | 1476 | tags=53%, list=11%, signal=60% |
| 21 | RNA\_PROCESSING |  | 138 | 0.40 | 2.12 | 0.000 | 0.001 | 0.027 | 3458 | tags=51%, list=26%, signal=68% |
| 22 | MITOTIC\_CELL\_CYCLE\_CHECKPOINT |  | 19 | 0.60 | 2.08 | 0.002 | 0.002 | 0.053 | 1865 | tags=53%, list=14%, signal=61% |
| 23 | MICROTUBULE\_CYTOSKELETON\_ORGANIZATION\_AND\_BIOGENESIS |  | 31 | 0.53 | 2.07 | 0.000 | 0.002 | 0.060 | 2724 | tags=55%, list=21%, signal=69% |
| 24 | DNA\_INTEGRITY\_CHECKPOINT |  | 22 | 0.58 | 2.07 | 0.000 | 0.002 | 0.060 | 2172 | tags=59%, list=17%, signal=71% |
| 25 | DNA\_REPLICATION\_INITIATION |  | 15 | 0.64 | 2.03 | 0.000 | 0.003 | 0.099 | 2172 | tags=73%, list=17%, signal=88% |
| 26 | MRNA\_METABOLIC\_PROCESS |  | 72 | 0.42 | 2.02 | 0.000 | 0.003 | 0.111 | 3053 | tags=49%, list=23%, signal=63% |
| 27 | MITOCHONDRION\_ORGANIZATION\_AND\_BIOGENESIS |  | 42 | 0.47 | 2.00 | 0.000 | 0.004 | 0.130 | 4023 | tags=60%, list=31%, signal=86% |
| 28 | PROTEIN\_FOLDING |  | 55 | 0.44 | 2.00 | 0.000 | 0.004 | 0.131 | 3248 | tags=51%, list=25%, signal=67% |
| 29 | REGULATION\_OF\_CELL\_CYCLE |  | 161 | 0.36 | 1.99 | 0.000 | 0.004 | 0.142 | 1954 | tags=35%, list=15%, signal=40% |
| 30 | REGULATION\_OF\_MITOTIC\_CELL\_CYCLE |  | 19 | 0.57 | 1.98 | 0.002 | 0.004 | 0.164 | 1215 | tags=47%, list=9%, signal=52% |
| 31 | DOUBLE\_STRAND\_BREAK\_REPAIR |  | 21 | 0.56 | 1.97 | 0.004 | 0.005 | 0.183 | 1962 | tags=52%, list=15%, signal=62% |
| 32 | NUCLEOBASENUCLEOSIDENUCLEOTIDE\_AND\_NUCLEIC\_ACID\_TRANSPORT |  | 26 | 0.51 | 1.95 | 0.002 | 0.006 | 0.210 | 2508 | tags=50%, list=19%, signal=62% |
| 33 | COENZYME\_METABOLIC\_PROCESS |  | 35 | 0.48 | 1.94 | 0.002 | 0.006 | 0.233 | 3450 | tags=49%, list=26%, signal=66% |
| 34 | TRNA\_METABOLIC\_PROCESS |  | 15 | 0.61 | 1.94 | 0.004 | 0.006 | 0.233 | 2816 | tags=67%, list=22%, signal=85% |
| 35 | MRNA\_PROCESSING\_GO\_0006397 |  | 61 | 0.42 | 1.92 | 0.002 | 0.007 | 0.264 | 3533 | tags=51%, list=27%, signal=69% |
| 36 | INTERPHASE\_OF\_MITOTIC\_CELL\_CYCLE |  | 57 | 0.42 | 1.90 | 0.000 | 0.009 | 0.344 | 3264 | tags=49%, list=25%, signal=65% |
| 37 | G1\_S\_TRANSITION\_OF\_MITOTIC\_CELL\_CYCLE |  | 23 | 0.51 | 1.89 | 0.000 | 0.009 | 0.369 | 2429 | tags=48%, list=19%, signal=59% |
| 38 | INTERPHASE |  | 63 | 0.41 | 1.89 | 0.000 | 0.009 | 0.375 | 3264 | tags=48%, list=25%, signal=63% |
| 39 | COFACTOR\_BIOSYNTHETIC\_PROCESS |  | 21 | 0.53 | 1.88 | 0.002 | 0.010 | 0.391 | 1495 | tags=38%, list=11%, signal=43% |
| 40 | CHROMOSOME\_ORGANIZATION\_AND\_BIOGENESIS |  | 107 | 0.37 | 1.88 | 0.000 | 0.010 | 0.408 | 2560 | tags=37%, list=20%, signal=46% |
| 41 | REGULATION\_OF\_CYCLIN\_DEPENDENT\_PROTEIN\_KINASE\_ACTIVITY |  | 40 | 0.45 | 1.88 | 0.004 | 0.010 | 0.410 | 2935 | tags=52%, list=22%, signal=67% |
| 42 | REGULATION\_OF\_DNA\_METABOLIC\_PROCESS |  | 40 | 0.44 | 1.86 | 0.000 | 0.011 | 0.456 | 2172 | tags=45%, list=17%, signal=54% |
| 43 | REGULATION\_OF\_DNA\_REPLICATION |  | 18 | 0.54 | 1.83 | 0.009 | 0.014 | 0.551 | 2172 | tags=50%, list=17%, signal=60% |
| 44 | MITOCHONDRIAL\_TRANSPORT |  | 18 | 0.54 | 1.81 | 0.004 | 0.016 | 0.603 | 1367 | tags=44%, list=10%, signal=50% |
| 45 | DNA\_DAMAGE\_CHECKPOINT |  | 19 | 0.53 | 1.78 | 0.004 | 0.019 | 0.667 | 2172 | tags=53%, list=17%, signal=63% |
| 46 | PROTEIN\_MODIFICATION\_BY\_SMALL\_PROTEIN\_CONJUGATION |  | 35 | 0.44 | 1.78 | 0.002 | 0.019 | 0.680 | 2141 | tags=40%, list=16%, signal=48% |
| 47 | UBIQUITIN\_CYCLE |  | 40 | 0.43 | 1.78 | 0.005 | 0.019 | 0.692 | 2141 | tags=38%, list=16%, signal=45% |
| 48 | DNA\_DAMAGE\_RESPONSESIGNAL\_TRANSDUCTION |  | 34 | 0.45 | 1.77 | 0.002 | 0.019 | 0.695 | 2302 | tags=47%, list=18%, signal=57% |
| 49 | BIOPOLYMER\_CATABOLIC\_PROCESS |  | 103 | 0.35 | 1.77 | 0.000 | 0.020 | 0.704 | 2544 | tags=36%, list=19%, signal=44% |
| 50 | COFACTOR\_METABOLIC\_PROCESS |  | 51 | 0.39 | 1.75 | 0.004 | 0.023 | 0.762 | 3509 | tags=43%, list=27%, signal=59% |
| 51 | DNA\_RECOMBINATION |  | 45 | 0.41 | 1.75 | 0.005 | 0.022 | 0.766 | 1359 | tags=33%, list=10%, signal=37% |
| 52 | TRANSCRIPTION\_INITIATION\_FROM\_RNA\_POLYMERASE\_II\_PROMOTER |  | 27 | 0.46 | 1.72 | 0.013 | 0.028 | 0.841 | 2694 | tags=44%, list=21%, signal=56% |
| 53 | DNA\_PACKAGING |  | 29 | 0.45 | 1.71 | 0.013 | 0.029 | 0.860 | 2501 | tags=45%, list=19%, signal=55% |
| 54 | PROTEIN\_CATABOLIC\_PROCESS |  | 60 | 0.38 | 1.71 | 0.006 | 0.029 | 0.872 | 2368 | tags=33%, list=18%, signal=41% |
| 55 | PROTEIN\_UBIQUITINATION |  | 32 | 0.43 | 1.70 | 0.011 | 0.030 | 0.883 | 2141 | tags=38%, list=16%, signal=45% |
| 56 | CELLULAR\_PROTEIN\_CATABOLIC\_PROCESS |  | 50 | 0.39 | 1.70 | 0.011 | 0.029 | 0.884 | 2368 | tags=34%, list=18%, signal=41% |
| 57 | MACROMOLECULE\_CATABOLIC\_PROCESS |  | 120 | 0.32 | 1.70 | 0.003 | 0.030 | 0.902 | 2544 | tags=33%, list=19%, signal=40% |
| 58 | NUCLEAR\_EXPORT |  | 26 | 0.46 | 1.69 | 0.007 | 0.032 | 0.917 | 2508 | tags=42%, list=19%, signal=52% |
| 59 | MEIOSIS\_I |  | 19 | 0.49 | 1.68 | 0.010 | 0.033 | 0.927 | 1359 | tags=37%, list=10%, signal=41% |
| 60 | BASE\_EXCISION\_REPAIR |  | 16 | 0.50 | 1.65 | 0.013 | 0.042 | 0.965 | 2540 | tags=44%, list=19%, signal=54% |
| 61 | ONE\_CARBON\_COMPOUND\_METABOLIC\_PROCESS |  | 24 | 0.45 | 1.62 | 0.022 | 0.051 | 0.986 | 2824 | tags=50%, list=22%, signal=64% |
| 62 | PROTEIN\_DNA\_COMPLEX\_ASSEMBLY |  | 45 | 0.37 | 1.62 | 0.010 | 0.051 | 0.986 | 2694 | tags=40%, list=21%, signal=50% |
| 63 | CYTOKINESIS |  | 17 | 0.49 | 1.62 | 0.027 | 0.051 | 0.986 | 1072 | tags=35%, list=8%, signal=38% |
| 64 | RNA\_EXPORT\_FROM\_NUCLEUS |  | 17 | 0.48 | 1.61 | 0.033 | 0.054 | 0.990 | 4503 | tags=71%, list=34%, signal=107% |
| 65 | MEIOTIC\_CELL\_CYCLE |  | 31 | 0.42 | 1.60 | 0.013 | 0.054 | 0.990 | 2724 | tags=42%, list=21%, signal=53% |
| 66 | CELLULAR\_MACROMOLECULE\_CATABOLIC\_PROCESS |  | 90 | 0.31 | 1.58 | 0.007 | 0.064 | 0.998 | 2508 | tags=31%, list=19%, signal=38% |
| 67 | APOPTOTIC\_NUCLEAR\_CHANGES |  | 17 | 0.47 | 1.58 | 0.039 | 0.064 | 0.998 | 2305 | tags=47%, list=18%, signal=57% |
| 68 | CHROMATIN\_ASSEMBLY\_OR\_DISASSEMBLY |  | 25 | 0.42 | 1.57 | 0.020 | 0.064 | 0.998 | 2501 | tags=48%, list=19%, signal=59% |
| 69 | NUCLEOTIDE\_METABOLIC\_PROCESS |  | 36 | 0.39 | 1.57 | 0.034 | 0.066 | 0.998 | 800 | tags=28%, list=6%, signal=30% |
| 70 | NUCLEOBASENUCLEOSIDE\_AND\_NUCLEOTIDE\_METABOLIC\_PROCESS |  | 46 | 0.37 | 1.57 | 0.006 | 0.065 | 0.998 | 800 | tags=26%, list=6%, signal=28% |
| 71 | CELLULAR\_COMPONENT\_DISASSEMBLY |  | 31 | 0.40 | 1.55 | 0.029 | 0.074 | 0.999 | 2305 | tags=39%, list=18%, signal=47% |
| 72 | NUCLEAR\_TRANSPORT |  | 77 | 0.31 | 1.53 | 0.012 | 0.081 | 1.000 | 3287 | tags=38%, list=25%, signal=50% |
| 73 | ORGANELLE\_ORGANIZATION\_AND\_BIOGENESIS |  | 407 | 0.24 | 1.52 | 0.000 | 0.087 | 1.000 | 3246 | tags=33%, list=25%, signal=43% |
| 74 | NUCLEOCYTOPLASMIC\_TRANSPORT |  | 77 | 0.31 | 1.51 | 0.021 | 0.092 | 1.000 | 3287 | tags=38%, list=25%, signal=50% |
| 75 | TRANSCRIPTION\_INITIATION |  | 33 | 0.38 | 1.49 | 0.044 | 0.103 | 1.000 | 2694 | tags=39%, list=21%, signal=49% |
| 76 | CELL\_DIVISION |  | 19 | 0.44 | 1.48 | 0.057 | 0.104 | 1.000 | 1072 | tags=32%, list=8%, signal=34% |
| 77 | MICROTUBULE\_BASED\_PROCESS |  | 75 | 0.31 | 1.48 | 0.018 | 0.108 | 1.000 | 3006 | tags=37%, list=23%, signal=48% |
| 78 | ESTABLISHMENT\_OF\_ORGANELLE\_LOCALIZATION |  | 16 | 0.46 | 1.46 | 0.064 | 0.120 | 1.000 | 940 | tags=38%, list=7%, signal=40% |
| 79 | VIRAL\_INFECTIOUS\_CYCLE |  | 29 | 0.38 | 1.46 | 0.054 | 0.121 | 1.000 | 1012 | tags=31%, list=8%, signal=34% |
| 80 | ORGANELLE\_LOCALIZATION |  | 21 | 0.40 | 1.43 | 0.081 | 0.142 | 1.000 | 940 | tags=29%, list=7%, signal=31% |
| 81 | NEGATIVE\_REGULATION\_OF\_DNA\_METABOLIC\_PROCESS |  | 16 | 0.43 | 1.42 | 0.088 | 0.145 | 1.000 | 2560 | tags=50%, list=20%, signal=62% |
| 82 | APOPTOTIC\_PROGRAM |  | 56 | 0.31 | 1.39 | 0.047 | 0.174 | 1.000 | 3666 | tags=46%, list=28%, signal=64% |
| 83 | NEGATIVE\_REGULATION\_OF\_BINDING |  | 16 | 0.43 | 1.39 | 0.097 | 0.173 | 1.000 | 2571 | tags=50%, list=20%, signal=62% |
| 84 | CHROMATIN\_REMODELING |  | 21 | 0.39 | 1.39 | 0.088 | 0.178 | 1.000 | 2481 | tags=43%, list=19%, signal=53% |
| 85 | ESTABLISHMENT\_AND\_OR\_MAINTENANCE\_OF\_CHROMATIN\_ARCHITECTURE |  | 65 | 0.30 | 1.38 | 0.059 | 0.182 | 1.000 | 2501 | tags=34%, list=19%, signal=42% |
| 86 | VIRAL\_REPRODUCTIVE\_PROCESS |  | 33 | 0.35 | 1.37 | 0.098 | 0.189 | 1.000 | 1194 | tags=30%, list=9%, signal=33% |
| 87 | ALCOHOL\_METABOLIC\_PROCESS |  | 82 | 0.28 | 1.37 | 0.062 | 0.190 | 1.000 | 3945 | tags=39%, list=30%, signal=56% |
| 88 | G1\_PHASE |  | 15 | 0.42 | 1.35 | 0.131 | 0.215 | 1.000 | 437 | tags=27%, list=3%, signal=28% |
| 89 | REGULATION\_OF\_GENE\_EXPRESSION\_EPIGENETIC |  | 27 | 0.35 | 1.35 | 0.094 | 0.213 | 1.000 | 2766 | tags=41%, list=21%, signal=52% |
| 90 | MEIOTIC\_RECOMBINATION |  | 16 | 0.42 | 1.34 | 0.124 | 0.213 | 1.000 | 1359 | tags=31%, list=10%, signal=35% |
| 91 | INTRACELLULAR\_TRANSPORT |  | 248 | 0.23 | 1.33 | 0.026 | 0.234 | 1.000 | 3654 | tags=35%, list=28%, signal=48% |
| 92 | RESPONSE\_TO\_ORGANIC\_SUBSTANCE |  | 27 | 0.35 | 1.32 | 0.123 | 0.237 | 1.000 | 2853 | tags=37%, list=22%, signal=47% |
| 93 | RESPONSE\_TO\_HYPOXIA |  | 27 | 0.35 | 1.32 | 0.133 | 0.241 | 1.000 | 2314 | tags=33%, list=18%, signal=40% |
| 94 | NUCLEAR\_ORGANIZATION\_AND\_BIOGENESIS |  | 23 | 0.36 | 1.29 | 0.145 | 0.277 | 1.000 | 2305 | tags=39%, list=18%, signal=47% |
| 95 | RESPONSE\_TO\_ABIOTIC\_STIMULUS |  | 79 | 0.26 | 1.29 | 0.086 | 0.276 | 1.000 | 3450 | tags=37%, list=26%, signal=50% |
| 96 | VIRAL\_REPRODUCTION |  | 38 | 0.31 | 1.29 | 0.126 | 0.278 | 1.000 | 1194 | tags=26%, list=9%, signal=29% |
| 97 | NEGATIVE\_REGULATION\_OF\_CATALYTIC\_ACTIVITY |  | 61 | 0.28 | 1.28 | 0.113 | 0.284 | 1.000 | 2872 | tags=36%, list=22%, signal=46% |
| 98 | RESPONSE\_TO\_STRESS |  | 467 | 0.20 | 1.28 | 0.024 | 0.285 | 1.000 | 3036 | tags=30%, list=23%, signal=37% |
| 99 | VIRAL\_GENOME\_REPLICATION |  | 20 | 0.37 | 1.27 | 0.132 | 0.291 | 1.000 | 1763 | tags=35%, list=13%, signal=40% |
| 100 | ESTABLISHMENT\_OF\_CELLULAR\_LOCALIZATION |  | 311 | 0.21 | 1.27 | 0.047 | 0.289 | 1.000 | 3287 | tags=31%, list=25%, signal=40% |
| 101 | CYTOSKELETON\_DEPENDENT\_INTRACELLULAR\_TRANSPORT |  | 25 | 0.34 | 1.27 | 0.157 | 0.295 | 1.000 | 3948 | tags=52%, list=30%, signal=74% |
| 102 | HETEROCYCLE\_METABOLIC\_PROCESS |  | 26 | 0.34 | 1.26 | 0.164 | 0.310 | 1.000 | 1495 | tags=23%, list=11%, signal=26% |
| 103 | CHROMATIN\_ASSEMBLY |  | 16 | 0.39 | 1.25 | 0.192 | 0.311 | 1.000 | 2501 | tags=44%, list=19%, signal=54% |
| 104 | RNA\_CATABOLIC\_PROCESS |  | 20 | 0.36 | 1.25 | 0.189 | 0.319 | 1.000 | 2508 | tags=45%, list=19%, signal=56% |
| 105 | CELLULAR\_LOCALIZATION |  | 323 | 0.20 | 1.25 | 0.059 | 0.319 | 1.000 | 3287 | tags=30%, list=25%, signal=39% |
| 106 | DNA\_CATABOLIC\_PROCESS |  | 21 | 0.36 | 1.24 | 0.172 | 0.317 | 1.000 | 3036 | tags=43%, list=23%, signal=56% |
| 107 | NEGATIVE\_REGULATION\_OF\_DNA\_BINDING |  | 15 | 0.39 | 1.23 | 0.209 | 0.333 | 1.000 | 2571 | tags=47%, list=20%, signal=58% |
| 108 | OXYGEN\_AND\_REACTIVE\_OXYGEN\_SPECIES\_METABOLIC\_PROCESS |  | 18 | 0.37 | 1.23 | 0.196 | 0.334 | 1.000 | 2825 | tags=44%, list=22%, signal=57% |
| 109 | CELL\_STRUCTURE\_DISASSEMBLY\_DURING\_APOPTOSIS |  | 17 | 0.37 | 1.23 | 0.212 | 0.333 | 1.000 | 2305 | tags=35%, list=18%, signal=43% |
| 110 | RIBONUCLEOPROTEIN\_COMPLEX\_BIOGENESIS\_AND\_ASSEMBLY |  | 68 | 0.26 | 1.22 | 0.155 | 0.353 | 1.000 | 3834 | tags=41%, list=29%, signal=58% |
| 111 | TRANSCRIPTION\_FROM\_RNA\_POLYMERASE\_II\_PROMOTER |  | 428 | 0.19 | 1.18 | 0.076 | 0.424 | 1.000 | 2712 | tags=25%, list=21%, signal=31% |
| 112 | CELLULAR\_RESPIRATION |  | 19 | 0.34 | 1.17 | 0.256 | 0.447 | 1.000 | 2664 | tags=37%, list=20%, signal=46% |
| 113 | CHROMATIN\_MODIFICATION |  | 46 | 0.27 | 1.17 | 0.218 | 0.446 | 1.000 | 3715 | tags=41%, list=28%, signal=57% |
| 114 | TRANSCRIPTION\_FROM\_RNA\_POLYMERASE\_III\_PROMOTER |  | 18 | 0.35 | 1.16 | 0.280 | 0.455 | 1.000 | 3852 | tags=56%, list=29%, signal=79% |
| 115 | REGULATION\_OF\_KINASE\_ACTIVITY |  | 135 | 0.22 | 1.15 | 0.198 | 0.465 | 1.000 | 2077 | tags=23%, list=16%, signal=27% |
| 116 | REGULATION\_OF\_HYDROLASE\_ACTIVITY |  | 65 | 0.25 | 1.15 | 0.228 | 0.462 | 1.000 | 3067 | tags=34%, list=23%, signal=44% |
| 117 | REGULATION\_OF\_CATALYTIC\_ACTIVITY |  | 238 | 0.20 | 1.15 | 0.149 | 0.461 | 1.000 | 3075 | tags=29%, list=23%, signal=37% |
| 118 | CELLULAR\_RESPONSE\_TO\_STIMULUS |  | 17 | 0.34 | 1.15 | 0.273 | 0.463 | 1.000 | 4213 | tags=53%, list=32%, signal=78% |
| 119 | NITROGEN\_COMPOUND\_BIOSYNTHETIC\_PROCESS |  | 25 | 0.30 | 1.15 | 0.273 | 0.464 | 1.000 | 2314 | tags=28%, list=18%, signal=34% |
| 120 | CELLULAR\_CATABOLIC\_PROCESS |  | 189 | 0.20 | 1.14 | 0.168 | 0.464 | 1.000 | 2681 | tags=25%, list=20%, signal=31% |
| 121 | INDUCTION\_OF\_APOPTOSIS\_BY\_EXTRACELLULAR\_SIGNALS |  | 25 | 0.31 | 1.14 | 0.296 | 0.473 | 1.000 | 2642 | tags=36%, list=20%, signal=45% |
| 122 | REGULATION\_OF\_TRANSFERASE\_ACTIVITY |  | 137 | 0.21 | 1.14 | 0.209 | 0.479 | 1.000 | 2077 | tags=23%, list=16%, signal=27% |
| 123 | CATABOLIC\_PROCESS |  | 201 | 0.20 | 1.13 | 0.189 | 0.476 | 1.000 | 2759 | tags=26%, list=21%, signal=32% |
| 124 | GLUTAMATE\_SIGNALING\_PATHWAY |  | 17 | 0.34 | 1.13 | 0.291 | 0.492 | 1.000 | 3114 | tags=29%, list=24%, signal=39% |
| 125 | NUCLEAR\_IMPORT |  | 47 | 0.26 | 1.11 | 0.270 | 0.514 | 1.000 | 3287 | tags=34%, list=25%, signal=45% |
| 126 | REGULATION\_OF\_PROTEIN\_KINASE\_ACTIVITY |  | 133 | 0.21 | 1.11 | 0.247 | 0.510 | 1.000 | 4351 | tags=43%, list=33%, signal=64% |
| 127 | NEGATIVE\_REGULATION\_OF\_TRANSPORT |  | 18 | 0.33 | 1.11 | 0.311 | 0.521 | 1.000 | 3489 | tags=44%, list=27%, signal=61% |
| 128 | NEURON\_APOPTOSIS |  | 15 | 0.34 | 1.10 | 0.311 | 0.530 | 1.000 | 1244 | tags=27%, list=10%, signal=29% |
| 129 | INTERACTION\_WITH\_HOST |  | 15 | 0.34 | 1.10 | 0.326 | 0.541 | 1.000 | 1194 | tags=27%, list=9%, signal=29% |
| 130 | RNA\_SPLICINGVIA\_TRANSESTERIFICATION\_REACTIONS |  | 27 | 0.29 | 1.10 | 0.315 | 0.537 | 1.000 | 3834 | tags=41%, list=29%, signal=57% |
| 131 | REGULATION\_OF\_MOLECULAR\_FUNCTION |  | 275 | 0.18 | 1.08 | 0.248 | 0.569 | 1.000 | 3075 | tags=28%, list=23%, signal=35% |
| 132 | RESPONSE\_TO\_HORMONE\_STIMULUS |  | 26 | 0.28 | 1.07 | 0.356 | 0.592 | 1.000 | 3506 | tags=38%, list=27%, signal=52% |
| 133 | CELLULAR\_BIOSYNTHETIC\_PROCESS |  | 273 | 0.18 | 1.07 | 0.295 | 0.601 | 1.000 | 2827 | tags=26%, list=22%, signal=32% |
| 134 | DNA\_DAMAGE\_RESPONSESIGNAL\_TRANSDUCTION\_RESULTING\_IN\_INDUCTION\_OF\_APOPTOSIS |  | 15 | 0.33 | 1.06 | 0.368 | 0.612 | 1.000 | 1101 | tags=27%, list=8%, signal=29% |
| 135 | REGULATION\_OF\_PROTEIN\_STABILITY |  | 17 | 0.32 | 1.05 | 0.387 | 0.636 | 1.000 | 4117 | tags=41%, list=31%, signal=60% |
| 136 | LIPID\_BIOSYNTHETIC\_PROCESS |  | 84 | 0.22 | 1.05 | 0.384 | 0.634 | 1.000 | 1876 | tags=21%, list=14%, signal=25% |
| 137 | INTRACELLULAR\_PROTEIN\_TRANSPORT |  | 127 | 0.19 | 1.05 | 0.365 | 0.640 | 1.000 | 3287 | tags=29%, list=25%, signal=39% |
| 138 | PIGMENT\_BIOSYNTHETIC\_PROCESS |  | 17 | 0.31 | 1.04 | 0.403 | 0.641 | 1.000 | 1495 | tags=24%, list=11%, signal=27% |
| 139 | GAMETE\_GENERATION |  | 92 | 0.21 | 1.04 | 0.368 | 0.648 | 1.000 | 3874 | tags=34%, list=30%, signal=48% |
| 140 | REGULATION\_OF\_NEUROTRANSMITTER\_LEVELS |  | 23 | 0.28 | 1.03 | 0.403 | 0.656 | 1.000 | 867 | tags=17%, list=7%, signal=19% |
| 141 | MACROMOLECULE\_LOCALIZATION |  | 202 | 0.18 | 1.02 | 0.406 | 0.679 | 1.000 | 3287 | tags=28%, list=25%, signal=37% |
| 142 | CYTOSKELETON\_ORGANIZATION\_AND\_BIOGENESIS |  | 182 | 0.18 | 1.02 | 0.389 | 0.675 | 1.000 | 2735 | tags=26%, list=21%, signal=32% |
| 143 | NEGATIVE\_REGULATION\_OF\_TRANSFERASE\_ACTIVITY |  | 27 | 0.27 | 1.02 | 0.444 | 0.676 | 1.000 | 2588 | tags=33%, list=20%, signal=41% |
| 144 | RESPONSE\_TO\_TEMPERATURE\_STIMULUS |  | 16 | 0.32 | 1.02 | 0.441 | 0.679 | 1.000 | 3450 | tags=44%, list=26%, signal=59% |
| 145 | STEROID\_BIOSYNTHETIC\_PROCESS |  | 22 | 0.29 | 1.02 | 0.435 | 0.681 | 1.000 | 4105 | tags=55%, list=31%, signal=79% |
| 146 | PROTEIN\_IMPORT |  | 58 | 0.22 | 1.01 | 0.435 | 0.682 | 1.000 | 3287 | tags=29%, list=25%, signal=39% |
| 147 | PROTEIN\_TARGETING |  | 94 | 0.20 | 1.01 | 0.413 | 0.680 | 1.000 | 3287 | tags=29%, list=25%, signal=38% |
| 148 | REGULATION\_OF\_PROGRAMMED\_CELL\_DEATH |  | 313 | 0.17 | 1.01 | 0.445 | 0.678 | 1.000 | 1589 | tags=17%, list=12%, signal=19% |
| 149 | PROTEIN\_TRANSPORT |  | 139 | 0.19 | 1.01 | 0.464 | 0.686 | 1.000 | 3287 | tags=29%, list=25%, signal=38% |
| 150 | NEGATIVE\_REGULATION\_OF\_APOPTOSIS |  | 136 | 0.19 | 1.01 | 0.454 | 0.682 | 1.000 | 1589 | tags=19%, list=12%, signal=22% |
| 151 | SECONDARY\_METABOLIC\_PROCESS |  | 23 | 0.27 | 1.00 | 0.464 | 0.692 | 1.000 | 1495 | tags=22%, list=11%, signal=24% |
| 152 | REGULATION\_OF\_APOPTOSIS |  | 312 | 0.17 | 1.00 | 0.465 | 0.698 | 1.000 | 1589 | tags=17%, list=12%, signal=19% |
| 153 | DIGESTION |  | 42 | 0.23 | 0.99 | 0.434 | 0.705 | 1.000 | 2960 | tags=24%, list=23%, signal=31% |
| 154 | PROGRAMMED\_CELL\_DEATH |  | 393 | 0.16 | 0.99 | 0.476 | 0.708 | 1.000 | 1611 | tags=17%, list=12%, signal=18% |
| 155 | NEGATIVE\_REGULATION\_OF\_PROGRAMMED\_CELL\_DEATH |  | 137 | 0.19 | 0.99 | 0.474 | 0.708 | 1.000 | 1589 | tags=19%, list=12%, signal=21% |
| 156 | APOPTOSIS\_GO |  | 392 | 0.16 | 0.99 | 0.495 | 0.709 | 1.000 | 1611 | tags=17%, list=12%, signal=18% |
| 157 | PIGMENT\_METABOLIC\_PROCESS |  | 18 | 0.29 | 0.99 | 0.472 | 0.709 | 1.000 | 1495 | tags=22%, list=11%, signal=25% |
| 158 | CARBOHYDRATE\_TRANSPORT |  | 17 | 0.29 | 0.97 | 0.500 | 0.751 | 1.000 | 2535 | tags=29%, list=19%, signal=36% |
| 159 | ENERGY\_DERIVATION\_BY\_OXIDATION\_OF\_ORGANIC\_COMPOUNDS |  | 37 | 0.24 | 0.96 | 0.503 | 0.755 | 1.000 | 1265 | tags=19%, list=10%, signal=21% |
| 160 | MORPHOGENESIS\_OF\_AN\_EPITHELIUM |  | 15 | 0.30 | 0.96 | 0.501 | 0.771 | 1.000 | 3858 | tags=47%, list=29%, signal=66% |
| 161 | STEROID\_METABOLIC\_PROCESS |  | 66 | 0.21 | 0.95 | 0.542 | 0.773 | 1.000 | 3780 | tags=35%, list=29%, signal=49% |
| 162 | MICROTUBULE\_BASED\_MOVEMENT |  | 16 | 0.30 | 0.95 | 0.534 | 0.774 | 1.000 | 2899 | tags=38%, list=22%, signal=48% |
| 163 | CELL\_PROJECTION\_BIOGENESIS |  | 20 | 0.28 | 0.95 | 0.521 | 0.780 | 1.000 | 4211 | tags=45%, list=32%, signal=66% |
| 164 | AROMATIC\_COMPOUND\_METABOLIC\_PROCESS |  | 26 | 0.25 | 0.95 | 0.527 | 0.776 | 1.000 | 270 | tags=15%, list=2%, signal=16% |
| 165 | STEROID\_HORMONE\_RECEPTOR\_SIGNALING\_PATHWAY |  | 18 | 0.28 | 0.94 | 0.548 | 0.782 | 1.000 | 1410 | tags=22%, list=11%, signal=25% |
| 166 | PROTEIN\_AMINO\_ACID\_O\_LINKED\_GLYCOSYLATION |  | 18 | 0.27 | 0.94 | 0.530 | 0.782 | 1.000 | 2791 | tags=39%, list=21%, signal=49% |
| 167 | PROTEIN\_RNA\_COMPLEX\_ASSEMBLY |  | 55 | 0.21 | 0.94 | 0.552 | 0.790 | 1.000 | 3834 | tags=38%, list=29%, signal=54% |
| 168 | REGULATION\_OF\_TRANSCRIPTION\_FROM\_RNA\_POLYMERASE\_II\_PROMOTER |  | 267 | 0.16 | 0.94 | 0.611 | 0.785 | 1.000 | 2824 | tags=24%, list=22%, signal=30% |
| 169 | COVALENT\_CHROMATIN\_MODIFICATION |  | 22 | 0.27 | 0.94 | 0.542 | 0.782 | 1.000 | 4035 | tags=45%, list=31%, signal=66% |
| 170 | INTRACELLULAR\_RECEPTOR\_MEDIATED\_SIGNALING\_PATHWAY |  | 18 | 0.28 | 0.93 | 0.570 | 0.784 | 1.000 | 1410 | tags=22%, list=11%, signal=25% |
| 171 | PROTEIN\_IMPORT\_INTO\_NUCLEUS |  | 45 | 0.22 | 0.93 | 0.562 | 0.781 | 1.000 | 3287 | tags=31%, list=25%, signal=41% |
| 172 | GENERATION\_OF\_A\_SIGNAL\_INVOLVED\_IN\_CELL\_CELL\_SIGNALING |  | 25 | 0.25 | 0.92 | 0.583 | 0.810 | 1.000 | 2476 | tags=28%, list=19%, signal=34% |
| 173 | SPLICEOSOME\_ASSEMBLY |  | 17 | 0.27 | 0.91 | 0.592 | 0.845 | 1.000 | 3834 | tags=41%, list=29%, signal=58% |
| 174 | BIOSYNTHETIC\_PROCESS |  | 402 | 0.14 | 0.90 | 0.781 | 0.847 | 1.000 | 2863 | tags=23%, list=22%, signal=29% |
| 175 | INDUCTION\_OF\_APOPTOSIS\_BY\_INTRACELLULAR\_SIGNALS |  | 23 | 0.25 | 0.90 | 0.606 | 0.860 | 1.000 | 1534 | tags=22%, list=12%, signal=25% |
| 176 | REGULATION\_OF\_TRANSPORT |  | 57 | 0.20 | 0.89 | 0.632 | 0.866 | 1.000 | 4277 | tags=42%, list=33%, signal=62% |
| 177 | REGULATION\_OF\_RNA\_METABOLIC\_PROCESS |  | 417 | 0.14 | 0.89 | 0.803 | 0.865 | 1.000 | 3207 | tags=26%, list=24%, signal=34% |
| 178 | SEXUAL\_REPRODUCTION |  | 109 | 0.17 | 0.89 | 0.677 | 0.868 | 1.000 | 3874 | tags=30%, list=30%, signal=43% |
| 179 | POSITIVE\_REGULATION\_OF\_CELL\_CYCLE |  | 15 | 0.28 | 0.88 | 0.635 | 0.873 | 1.000 | 446 | tags=20%, list=3%, signal=21% |
| 180 | ENERGY\_RESERVE\_METABOLIC\_PROCESS |  | 15 | 0.28 | 0.88 | 0.614 | 0.878 | 1.000 | 1265 | tags=20%, list=10%, signal=22% |
| 181 | TRANSMISSION\_OF\_NERVE\_IMPULSE |  | 167 | 0.16 | 0.87 | 0.732 | 0.885 | 1.000 | 2717 | tags=21%, list=21%, signal=26% |
| 182 | NEGATIVE\_REGULATION\_OF\_CELL\_ADHESION |  | 16 | 0.27 | 0.87 | 0.634 | 0.889 | 1.000 | 3441 | tags=44%, list=26%, signal=59% |
| 183 | RESPONSE\_TO\_RADIATION |  | 52 | 0.20 | 0.87 | 0.680 | 0.887 | 1.000 | 3314 | tags=31%, list=25%, signal=41% |
| 184 | REGULATION\_OF\_INTRACELLULAR\_TRANSPORT |  | 22 | 0.24 | 0.86 | 0.663 | 0.911 | 1.000 | 4554 | tags=50%, list=35%, signal=77% |
| 185 | REGULATION\_OF\_TRANSCRIPTIONDNA\_DEPENDENT |  | 412 | 0.14 | 0.85 | 0.894 | 0.914 | 1.000 | 2868 | tags=23%, list=22%, signal=29% |
| 186 | SYNAPTIC\_TRANSMISSION |  | 154 | 0.15 | 0.84 | 0.802 | 0.925 | 1.000 | 2716 | tags=20%, list=21%, signal=25% |
| 187 | REGULATION\_OF\_PHOSPHORYLATION |  | 42 | 0.20 | 0.84 | 0.730 | 0.926 | 1.000 | 4245 | tags=45%, list=32%, signal=67% |
| 188 | AEROBIC\_RESPIRATION |  | 15 | 0.27 | 0.84 | 0.681 | 0.922 | 1.000 | 2664 | tags=33%, list=20%, signal=42% |
| 189 | EPIDERMAL\_GROWTH\_FACTOR\_RECEPTOR\_SIGNALING\_PATHWAY |  | 18 | 0.25 | 0.84 | 0.690 | 0.919 | 1.000 | 4318 | tags=44%, list=33%, signal=66% |
| 190 | CASPASE\_ACTIVATION |  | 24 | 0.22 | 0.83 | 0.694 | 0.936 | 1.000 | 3309 | tags=38%, list=25%, signal=50% |
| 191 | REGULATION\_OF\_NUCLEOCYTOPLASMIC\_TRANSPORT |  | 19 | 0.24 | 0.83 | 0.713 | 0.932 | 1.000 | 1341 | tags=21%, list=10%, signal=23% |
| 192 | ESTABLISHMENT\_OF\_PROTEIN\_LOCALIZATION |  | 166 | 0.15 | 0.82 | 0.870 | 0.946 | 1.000 | 3287 | tags=26%, list=25%, signal=34% |
| 193 | DEVELOPMENT\_OF\_PRIMARY\_SEXUAL\_CHARACTERISTICS |  | 25 | 0.22 | 0.82 | 0.743 | 0.948 | 1.000 | 3059 | tags=28%, list=23%, signal=36% |
| 194 | PHOSPHOINOSITIDE\_BIOSYNTHETIC\_PROCESS |  | 21 | 0.23 | 0.80 | 0.746 | 0.972 | 1.000 | 1169 | tags=19%, list=9%, signal=21% |
| 195 | HISTONE\_MODIFICATION |  | 21 | 0.23 | 0.80 | 0.729 | 0.970 | 1.000 | 4035 | tags=43%, list=31%, signal=62% |
| 196 | CALCIUM\_INDEPENDENT\_CELL\_CELL\_ADHESION |  | 16 | 0.25 | 0.80 | 0.713 | 0.968 | 1.000 | 3909 | tags=38%, list=30%, signal=53% |
| 197 | MEMBRANE\_FUSION |  | 27 | 0.21 | 0.80 | 0.766 | 0.966 | 1.000 | 3618 | tags=37%, list=28%, signal=51% |
| 198 | EXOCYTOSIS |  | 22 | 0.22 | 0.80 | 0.754 | 0.961 | 1.000 | 2562 | tags=23%, list=20%, signal=28% |
| 199 | PROTEOLYSIS |  | 170 | 0.14 | 0.79 | 0.913 | 0.963 | 1.000 | 3925 | tags=32%, list=30%, signal=45% |
| 200 | PROTEIN\_LOCALIZATION |  | 184 | 0.14 | 0.78 | 0.936 | 0.974 | 1.000 | 3287 | tags=26%, list=25%, signal=34% |
| 201 | NEGATIVE\_REGULATION\_OF\_CELL\_CYCLE |  | 72 | 0.16 | 0.78 | 0.855 | 0.972 | 1.000 | 2440 | tags=22%, list=19%, signal=27% |
| 202 | CELL\_CYCLE\_ARREST\_GO\_0007050 |  | 52 | 0.17 | 0.78 | 0.860 | 0.973 | 1.000 | 4178 | tags=42%, list=32%, signal=62% |
| 203 | NITROGEN\_COMPOUND\_METABOLIC\_PROCESS |  | 141 | 0.14 | 0.77 | 0.937 | 0.972 | 1.000 | 2460 | tags=20%, list=19%, signal=24% |
| 204 | MEMBRANE\_LIPID\_BIOSYNTHETIC\_PROCESS |  | 41 | 0.18 | 0.77 | 0.836 | 0.970 | 1.000 | 1633 | tags=17%, list=12%, signal=19% |
| 205 | GLUCOSE\_METABOLIC\_PROCESS |  | 27 | 0.20 | 0.77 | 0.799 | 0.966 | 1.000 | 4715 | tags=44%, list=36%, signal=69% |
| 206 | RESPONSE\_TO\_UV |  | 22 | 0.21 | 0.77 | 0.785 | 0.964 | 1.000 | 3314 | tags=36%, list=25%, signal=49% |
| 207 | CELLULAR\_CARBOHYDRATE\_METABOLIC\_PROCESS |  | 106 | 0.15 | 0.76 | 0.911 | 0.967 | 1.000 | 2744 | tags=21%, list=21%, signal=26% |
| 208 | EMBRYONIC\_DEVELOPMENT |  | 46 | 0.17 | 0.76 | 0.861 | 0.972 | 1.000 | 3059 | tags=26%, list=23%, signal=34% |
| 209 | REGULATION\_OF\_CATABOLIC\_PROCESS |  | 15 | 0.24 | 0.75 | 0.795 | 0.979 | 1.000 | 2759 | tags=33%, list=21%, signal=42% |
| 210 | LIPID\_TRANSPORT |  | 27 | 0.20 | 0.74 | 0.852 | 0.979 | 1.000 | 2025 | tags=22%, list=15%, signal=26% |
| 211 | REPRODUCTION |  | 215 | 0.13 | 0.74 | 0.982 | 0.984 | 1.000 | 3541 | tags=26%, list=27%, signal=34% |
| 212 | PHOSPHOLIPID\_BIOSYNTHETIC\_PROCESS |  | 35 | 0.18 | 0.73 | 0.894 | 0.980 | 1.000 | 1633 | tags=17%, list=12%, signal=20% |
| 213 | STRESS\_ACTIVATED\_PROTEIN\_KINASE\_SIGNALING\_PATHWAY |  | 45 | 0.17 | 0.73 | 0.912 | 0.981 | 1.000 | 4141 | tags=38%, list=32%, signal=55% |
| 214 | JNK\_CASCADE |  | 44 | 0.17 | 0.73 | 0.887 | 0.978 | 1.000 | 4141 | tags=39%, list=32%, signal=56% |
| 215 | SENSORY\_PERCEPTION |  | 163 | 0.13 | 0.72 | 0.976 | 0.982 | 1.000 | 5083 | tags=40%, list=39%, signal=64% |
| 216 | GLYCEROPHOSPHOLIPID\_BIOSYNTHETIC\_PROCESS |  | 27 | 0.19 | 0.72 | 0.876 | 0.981 | 1.000 | 1169 | tags=15%, list=9%, signal=16% |
| 217 | POSITIVE\_REGULATION\_OF\_HYDROLASE\_ACTIVITY |  | 45 | 0.16 | 0.71 | 0.928 | 0.979 | 1.000 | 3309 | tags=29%, list=25%, signal=39% |
| 218 | NEUROLOGICAL\_SYSTEM\_PROCESS |  | 328 | 0.12 | 0.71 | 1.000 | 0.975 | 1.000 | 2717 | tags=17%, list=21%, signal=21% |
| 219 | BRAIN\_DEVELOPMENT |  | 39 | 0.17 | 0.70 | 0.912 | 0.986 | 1.000 | 3059 | tags=28%, list=23%, signal=37% |
| 220 | LIPOPROTEIN\_METABOLIC\_PROCESS |  | 30 | 0.18 | 0.69 | 0.908 | 0.988 | 1.000 | 2025 | tags=20%, list=15%, signal=24% |
| 221 | HOMEOSTASIS\_OF\_NUMBER\_OF\_CELLS |  | 20 | 0.20 | 0.68 | 0.897 | 0.986 | 1.000 | 2568 | tags=25%, list=20%, signal=31% |
| 222 | NEGATIVE\_REGULATION\_OF\_CELLULAR\_BIOSYNTHETIC\_PROCESS |  | 25 | 0.18 | 0.68 | 0.934 | 0.985 | 1.000 | 3295 | tags=28%, list=25%, signal=37% |
| 223 | BIOGENIC\_AMINE\_METABOLIC\_PROCESS |  | 16 | 0.21 | 0.67 | 0.899 | 0.987 | 1.000 | 1633 | tags=19%, list=12%, signal=21% |
| 224 | REGULATION\_OF\_CELL\_ADHESION |  | 31 | 0.17 | 0.66 | 0.940 | 0.987 | 1.000 | 4174 | tags=42%, list=32%, signal=61% |
| 225 | ANION\_TRANSPORT |  | 27 | 0.17 | 0.65 | 0.942 | 0.991 | 1.000 | 2964 | tags=22%, list=23%, signal=29% |
| 226 | NEGATIVE\_REGULATION\_OF\_BIOSYNTHETIC\_PROCESS |  | 26 | 0.17 | 0.64 | 0.934 | 0.993 | 1.000 | 3295 | tags=27%, list=25%, signal=36% |
| 227 | ADENYLATE\_CYCLASE\_ACTIVATION |  | 18 | 0.18 | 0.61 | 0.956 | 1.000 | 1.000 | 4523 | tags=39%, list=35%, signal=59% |
| 228 | FEEDING\_BEHAVIOR |  | 20 | 0.17 | 0.58 | 0.978 | 1.000 | 1.000 | 5118 | tags=45%, list=39%, signal=74% |
| 229 | LIPOPROTEIN\_BIOSYNTHETIC\_PROCESS |  | 23 | 0.16 | 0.57 | 0.970 | 1.000 | 1.000 | 4036 | tags=35%, list=31%, signal=50% |
| 230 | TUBE\_DEVELOPMENT |  | 15 | 0.17 | 0.56 | 0.974 | 1.000 | 1.000 | 3858 | tags=33%, list=29%, signal=47% |
| 231 | DETECTION\_OF\_ABIOTIC\_STIMULUS |  | 16 | 0.16 | 0.52 | 0.983 | 1.000 | 1.000 | 62 | tags=6%, list=0%, signal=6% |
| 232 | RESPONSE\_TO\_LIGHT\_STIMULUS |  | 40 | 0.12 | 0.52 | 0.996 | 0.999 | 1.000 | 3314 | tags=25%, list=25%, signal=33% |
| 233 | NEGATIVE\_REGULATION\_OF\_TRANSLATION |  | 19 | 0.15 | 0.50 | 0.992 | 0.997 | 1.000 | 3295 | tags=26%, list=25%, signal=35% |
Table: Gene sets enriched in phenotype **na**[plain text format]****

  
