# Supplementary material for: Multiple-input multiple-output causal strategies for gene selection
Source: BMC Bioinformatics. 2011 Nov 25;12:458. doi: 10.1186/1471-2105-12-458 (PMC3323860; doi:10.1186/1471-2105-12-458)
Supplement: Additional file 2 — Archive containing the output files computed by the preranked GSEA for λ ∈ {0.1,0.2,0.3,0.4,0.5} (GSEA_MIMO_part1.zip). [file 1471-2105-12-458-S2.ZIP › mFS04_entrez_mimo.GseaPreranked.1316038376454/gsea_report_for_na_neg_1316038376454.html]

Report for na\_neg 1316038376454 [GSEA]

| GS  follow link to MSigDB | GS DETAILS | SIZE | ES | NES | NOM p-val | FDR q-val | FWER p-val | RANK AT MAX | LEADING EDGE || 1 | IMMUNE\_RESPONSE |  | 212 | -0.40 | -2.36 | 0.000 | 0.004 | 0.003 | 2909 | tags=43%, list=22%, signal=54% |
| 2 | IMMUNE\_SYSTEM\_PROCESS |  | 298 | -0.36 | -2.28 | 0.000 | 0.004 | 0.006 | 2909 | tags=40%, list=22%, signal=51% |
| 3 | DEFENSE\_RESPONSE |  | 238 | -0.36 | -2.21 | 0.000 | 0.006 | 0.014 | 3284 | tags=41%, list=25%, signal=53% |
| 4 | REGULATION\_OF\_IMMUNE\_RESPONSE |  | 28 | -0.52 | -2.09 | 0.000 | 0.017 | 0.052 | 4560 | tags=75%, list=35%, signal=115% |
| 5 | POSITIVE\_REGULATION\_OF\_IMMUNE\_RESPONSE |  | 24 | -0.56 | -2.09 | 0.000 | 0.015 | 0.057 | 2909 | tags=58%, list=22%, signal=75% |
| 6 | POSITIVE\_REGULATION\_OF\_IMMUNE\_SYSTEM\_PROCESS |  | 44 | -0.46 | -2.06 | 0.000 | 0.017 | 0.077 | 4106 | tags=61%, list=31%, signal=89% |
| 7 | INFLAMMATORY\_RESPONSE |  | 115 | -0.38 | -2.02 | 0.000 | 0.021 | 0.109 | 2982 | tags=41%, list=23%, signal=52% |
| 8 | POSITIVE\_REGULATION\_OF\_MULTICELLULAR\_ORGANISMAL\_PROCESS |  | 56 | -0.44 | -2.02 | 0.000 | 0.019 | 0.114 | 4106 | tags=59%, list=31%, signal=85% |
| 9 | RESPONSE\_TO\_WOUNDING |  | 171 | -0.35 | -1.98 | 0.000 | 0.025 | 0.161 | 3451 | tags=42%, list=26%, signal=56% |
| 10 | REGULATION\_OF\_IMMUNE\_SYSTEM\_PROCESS |  | 57 | -0.42 | -1.96 | 0.000 | 0.025 | 0.175 | 4106 | tags=60%, list=31%, signal=87% |
| 11 | CELLULAR\_DEFENSE\_RESPONSE |  | 54 | -0.42 | -1.92 | 0.000 | 0.035 | 0.261 | 3652 | tags=48%, list=28%, signal=67% |
| 12 | ADAPTIVE\_IMMUNE\_RESPONSE\_GO\_0002460 |  | 22 | -0.49 | -1.78 | 0.014 | 0.105 | 0.630 | 4084 | tags=59%, list=31%, signal=86% |
| 13 | ADAPTIVE\_IMMUNE\_RESPONSE |  | 23 | -0.46 | -1.74 | 0.009 | 0.133 | 0.739 | 4084 | tags=57%, list=31%, signal=82% |
| 14 | HEMOPOIETIC\_OR\_LYMPHOID\_ORGAN\_DEVELOPMENT |  | 71 | -0.35 | -1.73 | 0.000 | 0.136 | 0.773 | 2903 | tags=39%, list=22%, signal=50% |
| 15 | HEMOPOIESIS |  | 69 | -0.35 | -1.72 | 0.004 | 0.142 | 0.813 | 2903 | tags=39%, list=22%, signal=50% |
| 16 | REGULATION\_OF\_MULTICELLULAR\_ORGANISMAL\_PROCESS |  | 131 | -0.31 | -1.71 | 0.002 | 0.148 | 0.839 | 4106 | tags=47%, list=31%, signal=67% |
| 17 | HUMORAL\_IMMUNE\_RESPONSE |  | 30 | -0.42 | -1.68 | 0.013 | 0.168 | 0.885 | 3131 | tags=53%, list=24%, signal=70% |
| 18 | IMMUNE\_SYSTEM\_DEVELOPMENT |  | 75 | -0.33 | -1.68 | 0.002 | 0.165 | 0.895 | 2903 | tags=39%, list=22%, signal=49% |
| 19 | REGULATION\_OF\_DEFENSE\_RESPONSE |  | 15 | -0.52 | -1.67 | 0.025 | 0.159 | 0.900 | 4084 | tags=67%, list=31%, signal=97% |
| 20 | IMMUNE\_EFFECTOR\_PROCESS |  | 34 | -0.40 | -1.67 | 0.009 | 0.156 | 0.908 | 2800 | tags=50%, list=21%, signal=63% |
| 21 | LIPID\_CATABOLIC\_PROCESS |  | 34 | -0.40 | -1.67 | 0.011 | 0.155 | 0.919 | 4015 | tags=56%, list=31%, signal=80% |
| 22 | RECEPTOR\_MEDIATED\_ENDOCYTOSIS |  | 31 | -0.42 | -1.66 | 0.009 | 0.156 | 0.927 | 2148 | tags=35%, list=16%, signal=42% |
| 23 | RESPONSE\_TO\_EXTERNAL\_STIMULUS |  | 278 | -0.27 | -1.66 | 0.000 | 0.150 | 0.928 | 3295 | tags=35%, list=25%, signal=46% |
| 24 | LYMPHOCYTE\_ACTIVATION |  | 54 | -0.35 | -1.64 | 0.004 | 0.165 | 0.947 | 3284 | tags=46%, list=25%, signal=62% |
| 25 | REGULATION\_OF\_CELL\_DIFFERENTIATION |  | 48 | -0.37 | -1.63 | 0.007 | 0.160 | 0.948 | 4694 | tags=56%, list=36%, signal=87% |
| 26 | TRANSFORMING\_GROWTH\_FACTOR\_BETA\_RECEPTOR\_SIGNALING\_PATHWAY |  | 34 | -0.39 | -1.63 | 0.020 | 0.157 | 0.951 | 3067 | tags=44%, list=23%, signal=57% |
| 27 | JAK\_STAT\_CASCADE |  | 26 | -0.42 | -1.61 | 0.028 | 0.177 | 0.968 | 1683 | tags=35%, list=13%, signal=40% |
| 28 | POSITIVE\_REGULATION\_OF\_RESPONSE\_TO\_STIMULUS |  | 35 | -0.38 | -1.59 | 0.026 | 0.191 | 0.984 | 2909 | tags=46%, list=22%, signal=59% |
| 29 | LEUKOCYTE\_DIFFERENTIATION |  | 34 | -0.38 | -1.59 | 0.009 | 0.191 | 0.985 | 2867 | tags=44%, list=22%, signal=56% |
| 30 | T\_CELL\_ACTIVATION |  | 39 | -0.36 | -1.56 | 0.029 | 0.228 | 0.995 | 3247 | tags=44%, list=25%, signal=58% |
| 31 | REGULATION\_OF\_ANGIOGENESIS |  | 24 | -0.41 | -1.55 | 0.039 | 0.239 | 0.998 | 2323 | tags=46%, list=18%, signal=56% |
| 32 | ENZYME\_LINKED\_RECEPTOR\_PROTEIN\_SIGNALING\_PATHWAY |  | 128 | -0.28 | -1.55 | 0.005 | 0.235 | 0.998 | 3159 | tags=33%, list=24%, signal=43% |
| 33 | LEUKOCYTE\_ACTIVATION |  | 59 | -0.32 | -1.54 | 0.009 | 0.237 | 0.999 | 3284 | tags=44%, list=25%, signal=59% |
| 34 | CELL\_ACTIVATION |  | 64 | -0.32 | -1.54 | 0.007 | 0.236 | 0.999 | 4161 | tags=52%, list=32%, signal=75% |
| 35 | TRANSMEMBRANE\_RECEPTOR\_PROTEIN\_SERINE\_THREONINE\_KINASE\_SIGNALING\_PATHWAY |  | 42 | -0.35 | -1.53 | 0.022 | 0.233 | 0.999 | 3139 | tags=40%, list=24%, signal=53% |
| 36 | LYMPHOCYTE\_DIFFERENTIATION |  | 23 | -0.41 | -1.50 | 0.041 | 0.281 | 0.999 | 3247 | tags=52%, list=25%, signal=69% |
| 37 | ACTIN\_CYTOSKELETON\_ORGANIZATION\_AND\_BIOGENESIS |  | 90 | -0.29 | -1.50 | 0.018 | 0.273 | 0.999 | 1883 | tags=26%, list=14%, signal=30% |
| 38 | CELLULAR\_LIPID\_CATABOLIC\_PROCESS |  | 31 | -0.37 | -1.50 | 0.046 | 0.275 | 0.999 | 4002 | tags=52%, list=31%, signal=74% |
| 39 | POSITIVE\_REGULATION\_OF\_PHOSPHATE\_METABOLIC\_PROCESS |  | 23 | -0.40 | -1.49 | 0.044 | 0.285 | 0.999 | 1426 | tags=30%, list=11%, signal=34% |
| 40 | B\_CELL\_ACTIVATION |  | 17 | -0.43 | -1.48 | 0.061 | 0.290 | 1.000 | 3284 | tags=59%, list=25%, signal=78% |
| 41 | POSITIVE\_REGULATION\_OF\_CELL\_DIFFERENTIATION |  | 21 | -0.41 | -1.47 | 0.059 | 0.293 | 1.000 | 4443 | tags=67%, list=34%, signal=101% |
| 42 | REGULATION\_OF\_RESPONSE\_TO\_STIMULUS |  | 49 | -0.32 | -1.47 | 0.024 | 0.289 | 1.000 | 4106 | tags=55%, list=31%, signal=80% |
| 43 | WOUND\_HEALING |  | 49 | -0.32 | -1.46 | 0.038 | 0.299 | 1.000 | 3974 | tags=45%, list=30%, signal=64% |
| 44 | INNATE\_IMMUNE\_RESPONSE |  | 19 | -0.41 | -1.46 | 0.058 | 0.297 | 1.000 | 4824 | tags=74%, list=37%, signal=117% |
| 45 | PROTEIN\_AMINO\_ACID\_N\_LINKED\_GLYCOSYLATION |  | 27 | -0.36 | -1.45 | 0.049 | 0.318 | 1.000 | 2472 | tags=37%, list=19%, signal=46% |
| 46 | CYTOKINE\_AND\_CHEMOKINE\_MEDIATED\_SIGNALING\_PATHWAY |  | 19 | -0.41 | -1.44 | 0.063 | 0.319 | 1.000 | 2269 | tags=37%, list=17%, signal=45% |
| 47 | NEGATIVE\_REGULATION\_OF\_SIGNAL\_TRANSDUCTION |  | 31 | -0.36 | -1.44 | 0.057 | 0.314 | 1.000 | 4753 | tags=55%, list=36%, signal=86% |
| 48 | FATTY\_ACID\_METABOLIC\_PROCESS |  | 56 | -0.31 | -1.44 | 0.048 | 0.313 | 1.000 | 4002 | tags=50%, list=31%, signal=72% |
| 49 | CELL\_SUBSTRATE\_ADHESION |  | 36 | -0.34 | -1.44 | 0.063 | 0.307 | 1.000 | 1709 | tags=31%, list=13%, signal=35% |
| 50 | MULTI\_ORGANISM\_PROCESS |  | 137 | -0.25 | -1.43 | 0.014 | 0.307 | 1.000 | 4220 | tags=47%, list=32%, signal=68% |
| 51 | REGULATION\_OF\_PROTEIN\_AMINO\_ACID\_PHOSPHORYLATION |  | 23 | -0.39 | -1.43 | 0.069 | 0.310 | 1.000 | 2599 | tags=35%, list=20%, signal=43% |
| 52 | CATION\_HOMEOSTASIS |  | 94 | -0.27 | -1.43 | 0.023 | 0.304 | 1.000 | 3181 | tags=36%, list=24%, signal=47% |
| 53 | PROTEIN\_AMINO\_ACID\_PHOSPHORYLATION |  | 231 | -0.23 | -1.43 | 0.000 | 0.302 | 1.000 | 2985 | tags=30%, list=23%, signal=38% |
| 54 | PEPTIDYL\_TYROSINE\_MODIFICATION |  | 23 | -0.39 | -1.42 | 0.063 | 0.310 | 1.000 | 1531 | tags=26%, list=12%, signal=29% |
| 55 | MESODERM\_DEVELOPMENT |  | 22 | -0.39 | -1.42 | 0.077 | 0.307 | 1.000 | 4422 | tags=55%, list=34%, signal=82% |
| 56 | POSITIVE\_REGULATION\_OF\_SIGNAL\_TRANSDUCTION |  | 97 | -0.27 | -1.41 | 0.021 | 0.324 | 1.000 | 4082 | tags=46%, list=31%, signal=67% |
| 57 | SMALL\_GTPASE\_MEDIATED\_SIGNAL\_TRANSDUCTION |  | 77 | -0.28 | -1.41 | 0.024 | 0.320 | 1.000 | 3324 | tags=39%, list=25%, signal=52% |
| 58 | RESPONSE\_TO\_OTHER\_ORGANISM |  | 69 | -0.29 | -1.41 | 0.055 | 0.316 | 1.000 | 2809 | tags=36%, list=21%, signal=46% |
| 59 | COAGULATION |  | 41 | -0.32 | -1.41 | 0.050 | 0.311 | 1.000 | 3438 | tags=39%, list=26%, signal=53% |
| 60 | BLOOD\_COAGULATION |  | 41 | -0.32 | -1.40 | 0.079 | 0.313 | 1.000 | 3438 | tags=39%, list=26%, signal=53% |
| 61 | MAINTENANCE\_OF\_LOCALIZATION |  | 21 | -0.39 | -1.40 | 0.082 | 0.314 | 1.000 | 2636 | tags=38%, list=20%, signal=48% |
| 62 | CELLULAR\_CATION\_HOMEOSTASIS |  | 91 | -0.27 | -1.40 | 0.037 | 0.310 | 1.000 | 3181 | tags=36%, list=24%, signal=48% |
| 63 | REGULATION\_OF\_LYMPHOCYTE\_ACTIVATION |  | 31 | -0.35 | -1.40 | 0.073 | 0.307 | 1.000 | 3247 | tags=45%, list=25%, signal=60% |
| 64 | REGULATION\_OF\_SIGNAL\_TRANSDUCTION |  | 173 | -0.24 | -1.40 | 0.024 | 0.304 | 1.000 | 3339 | tags=35%, list=26%, signal=46% |
| 65 | POSITIVE\_REGULATION\_OF\_PROTEIN\_AMINO\_ACID\_PHOSPHORYLATION |  | 15 | -0.42 | -1.38 | 0.092 | 0.326 | 1.000 | 1426 | tags=33%, list=11%, signal=37% |
| 66 | GROWTH |  | 59 | -0.29 | -1.38 | 0.058 | 0.324 | 1.000 | 4096 | tags=44%, list=31%, signal=64% |
| 67 | REGULATION\_OF\_BLOOD\_PRESSURE |  | 22 | -0.37 | -1.38 | 0.101 | 0.324 | 1.000 | 3394 | tags=41%, list=26%, signal=55% |
| 68 | DETECTION\_OF\_STIMULUS |  | 36 | -0.33 | -1.38 | 0.073 | 0.320 | 1.000 | 5033 | tags=56%, list=38%, signal=90% |
| 69 | FEMALE\_PREGNANCY |  | 42 | -0.31 | -1.38 | 0.083 | 0.316 | 1.000 | 4099 | tags=52%, list=31%, signal=76% |
| 70 | TRANSMEMBRANE\_RECEPTOR\_PROTEIN\_TYROSINE\_KINASE\_SIGNALING\_PATHWAY |  | 76 | -0.27 | -1.37 | 0.051 | 0.317 | 1.000 | 1368 | tags=21%, list=10%, signal=23% |
| 71 | AMINE\_TRANSPORT |  | 36 | -0.33 | -1.36 | 0.080 | 0.328 | 1.000 | 2282 | tags=28%, list=17%, signal=34% |
| 72 | GLYCOPROTEIN\_METABOLIC\_PROCESS |  | 82 | -0.26 | -1.35 | 0.040 | 0.361 | 1.000 | 3632 | tags=39%, list=28%, signal=54% |
| 73 | POSITIVE\_REGULATION\_OF\_LYMPHOCYTE\_ACTIVATION |  | 23 | -0.36 | -1.34 | 0.098 | 0.369 | 1.000 | 3247 | tags=43%, list=25%, signal=58% |
| 74 | REGULATION\_OF\_T\_CELL\_ACTIVATION |  | 25 | -0.35 | -1.34 | 0.092 | 0.366 | 1.000 | 3247 | tags=44%, list=25%, signal=58% |
| 75 | POSITIVE\_REGULATION\_OF\_CYTOKINE\_BIOSYNTHETIC\_PROCESS |  | 21 | -0.37 | -1.33 | 0.118 | 0.373 | 1.000 | 2863 | tags=43%, list=22%, signal=55% |
| 76 | PROTEIN\_KINASE\_CASCADE |  | 239 | -0.22 | -1.33 | 0.015 | 0.371 | 1.000 | 2247 | tags=25%, list=17%, signal=30% |
| 77 | ACTIN\_FILAMENT\_BASED\_PROCESS |  | 99 | -0.26 | -1.33 | 0.051 | 0.366 | 1.000 | 3060 | tags=31%, list=23%, signal=41% |
| 78 | RESPONSE\_TO\_VIRUS |  | 45 | -0.30 | -1.33 | 0.093 | 0.363 | 1.000 | 2988 | tags=42%, list=23%, signal=55% |
| 79 | MUSCLE\_DEVELOPMENT |  | 85 | -0.26 | -1.33 | 0.059 | 0.366 | 1.000 | 3392 | tags=40%, list=26%, signal=54% |
| 80 | PROTEIN\_COMPLEX\_ASSEMBLY |  | 157 | -0.23 | -1.32 | 0.039 | 0.372 | 1.000 | 2382 | tags=26%, list=18%, signal=32% |
| 81 | REGULATION\_OF\_CYTOSKELETON\_ORGANIZATION\_AND\_BIOGENESIS |  | 26 | -0.34 | -1.31 | 0.116 | 0.391 | 1.000 | 2599 | tags=35%, list=20%, signal=43% |
| 82 | CELL\_MATRIX\_ADHESION |  | 35 | -0.31 | -1.31 | 0.112 | 0.393 | 1.000 | 1709 | tags=29%, list=13%, signal=33% |
| 83 | REGULATION\_OF\_ANATOMICAL\_STRUCTURE\_MORPHOGENESIS |  | 17 | -0.38 | -1.31 | 0.129 | 0.390 | 1.000 | 4856 | tags=53%, list=37%, signal=84% |
| 84 | AMINO\_ACID\_TRANSPORT |  | 25 | -0.34 | -1.30 | 0.140 | 0.394 | 1.000 | 2282 | tags=32%, list=17%, signal=39% |
| 85 | CYTOKINE\_PRODUCTION |  | 61 | -0.28 | -1.30 | 0.080 | 0.394 | 1.000 | 2863 | tags=34%, list=22%, signal=44% |
| 86 | ACTIN\_POLYMERIZATION\_AND\_OR\_DEPOLYMERIZATION |  | 20 | -0.37 | -1.30 | 0.152 | 0.396 | 1.000 | 2377 | tags=30%, list=18%, signal=37% |
| 87 | POSITIVE\_REGULATION\_OF\_PHOSPHORYLATION |  | 21 | -0.36 | -1.30 | 0.147 | 0.394 | 1.000 | 1426 | tags=29%, list=11%, signal=32% |
| 88 | RESPONSE\_TO\_DRUG |  | 21 | -0.36 | -1.29 | 0.151 | 0.395 | 1.000 | 2557 | tags=43%, list=20%, signal=53% |
| 89 | PROTEIN\_AMINO\_ACID\_DEPHOSPHORYLATION |  | 60 | -0.27 | -1.29 | 0.116 | 0.398 | 1.000 | 1590 | tags=22%, list=12%, signal=25% |
| 90 | RAS\_PROTEIN\_SIGNAL\_TRANSDUCTION |  | 55 | -0.28 | -1.29 | 0.107 | 0.395 | 1.000 | 3324 | tags=40%, list=25%, signal=53% |
| 91 | PEPTIDYL\_TYROSINE\_PHOSPHORYLATION |  | 21 | -0.35 | -1.29 | 0.154 | 0.393 | 1.000 | 1531 | tags=24%, list=12%, signal=27% |
| 92 | ANATOMICAL\_STRUCTURE\_FORMATION |  | 52 | -0.27 | -1.29 | 0.109 | 0.389 | 1.000 | 2535 | tags=33%, list=19%, signal=40% |
| 93 | MONOCARBOXYLIC\_ACID\_METABOLIC\_PROCESS |  | 77 | -0.26 | -1.29 | 0.075 | 0.389 | 1.000 | 4005 | tags=44%, list=31%, signal=63% |
| 94 | HEMOSTASIS |  | 46 | -0.29 | -1.28 | 0.094 | 0.387 | 1.000 | 3438 | tags=37%, list=26%, signal=50% |
| 95 | REGULATION\_OF\_CELL\_PROLIFERATION |  | 275 | -0.20 | -1.27 | 0.018 | 0.420 | 1.000 | 2863 | tags=28%, list=22%, signal=35% |
| 96 | PROTEIN\_PROCESSING |  | 41 | -0.29 | -1.26 | 0.130 | 0.427 | 1.000 | 4006 | tags=41%, list=31%, signal=60% |
| 97 | REGULATION\_OF\_BODY\_FLUID\_LEVELS |  | 55 | -0.27 | -1.26 | 0.123 | 0.426 | 1.000 | 3438 | tags=36%, list=26%, signal=49% |
| 98 | DEPHOSPHORYLATION |  | 67 | -0.25 | -1.26 | 0.119 | 0.425 | 1.000 | 1590 | tags=21%, list=12%, signal=24% |
| 99 | ANGIOGENESIS |  | 44 | -0.29 | -1.26 | 0.141 | 0.426 | 1.000 | 2535 | tags=34%, list=19%, signal=42% |
| 100 | POSITIVE\_REGULATION\_OF\_TRANSFERASE\_ACTIVITY |  | 71 | -0.25 | -1.25 | 0.108 | 0.446 | 1.000 | 2381 | tags=27%, list=18%, signal=33% |
| 101 | LIPID\_METABOLIC\_PROCESS |  | 283 | -0.20 | -1.24 | 0.052 | 0.449 | 1.000 | 3890 | tags=39%, list=30%, signal=54% |
| 102 | PROTEIN\_OLIGOMERIZATION |  | 37 | -0.30 | -1.24 | 0.145 | 0.453 | 1.000 | 2668 | tags=32%, list=20%, signal=41% |
| 103 | POSITIVE\_REGULATION\_OF\_TRANSLATION |  | 28 | -0.31 | -1.24 | 0.170 | 0.454 | 1.000 | 2863 | tags=39%, list=22%, signal=50% |
| 104 | POSITIVE\_REGULATION\_OF\_SECRETION |  | 18 | -0.36 | -1.24 | 0.198 | 0.450 | 1.000 | 4406 | tags=61%, list=34%, signal=92% |
| 105 | PHOSPHORYLATION |  | 262 | -0.20 | -1.23 | 0.063 | 0.450 | 1.000 | 2985 | tags=29%, list=23%, signal=36% |
| 106 | PHOSPHOLIPID\_METABOLIC\_PROCESS |  | 63 | -0.25 | -1.23 | 0.127 | 0.455 | 1.000 | 3775 | tags=41%, list=29%, signal=58% |
| 107 | POSITIVE\_REGULATION\_OF\_CELL\_PROLIFERATION |  | 129 | -0.22 | -1.23 | 0.101 | 0.454 | 1.000 | 1887 | tags=22%, list=14%, signal=26% |
| 108 | REGULATION\_OF\_I\_KAPPAB\_KINASE\_NF\_KAPPAB\_CASCADE |  | 72 | -0.25 | -1.23 | 0.130 | 0.451 | 1.000 | 3681 | tags=43%, list=28%, signal=60% |
| 109 | NEGATIVE\_REGULATION\_OF\_TRANSCRIPTION |  | 166 | -0.21 | -1.23 | 0.097 | 0.452 | 1.000 | 2737 | tags=28%, list=21%, signal=35% |
| 110 | ICOSANOID\_METABOLIC\_PROCESS |  | 16 | -0.38 | -1.22 | 0.227 | 0.452 | 1.000 | 3067 | tags=44%, list=23%, signal=57% |
| 111 | G\_PROTEIN\_SIGNALING\_COUPLED\_TO\_CAMP\_NUCLEOTIDE\_SECOND\_MESSENGER |  | 62 | -0.26 | -1.22 | 0.155 | 0.449 | 1.000 | 2042 | tags=21%, list=16%, signal=25% |
| 112 | CAMP\_MEDIATED\_SIGNALING |  | 63 | -0.26 | -1.22 | 0.148 | 0.448 | 1.000 | 2042 | tags=21%, list=16%, signal=24% |
| 113 | ORGAN\_MORPHOGENESIS |  | 131 | -0.22 | -1.21 | 0.102 | 0.467 | 1.000 | 1485 | tags=19%, list=11%, signal=21% |
| 114 | MUSCLE\_CELL\_DIFFERENTIATION |  | 21 | -0.33 | -1.21 | 0.204 | 0.464 | 1.000 | 3392 | tags=48%, list=26%, signal=64% |
| 115 | GENERATION\_OF\_NEURONS |  | 65 | -0.25 | -1.21 | 0.143 | 0.478 | 1.000 | 3616 | tags=35%, list=28%, signal=49% |
| 116 | RESPONSE\_TO\_BACTERIUM |  | 22 | -0.32 | -1.20 | 0.227 | 0.484 | 1.000 | 1830 | tags=27%, list=14%, signal=32% |
| 117 | NEURON\_DIFFERENTIATION |  | 58 | -0.26 | -1.20 | 0.158 | 0.481 | 1.000 | 3407 | tags=33%, list=26%, signal=44% |
| 118 | POSITIVE\_REGULATION\_OF\_CELLULAR\_PROTEIN\_METABOLIC\_PROCESS |  | 61 | -0.25 | -1.20 | 0.170 | 0.479 | 1.000 | 2377 | tags=30%, list=18%, signal=36% |
| 119 | POSITIVE\_REGULATION\_OF\_PROTEIN\_METABOLIC\_PROCESS |  | 63 | -0.25 | -1.20 | 0.180 | 0.481 | 1.000 | 2377 | tags=30%, list=18%, signal=37% |
| 120 | ACTIVATION\_OF\_NF\_KAPPAB\_TRANSCRIPTION\_FACTOR |  | 15 | -0.37 | -1.19 | 0.213 | 0.492 | 1.000 | 4206 | tags=60%, list=32%, signal=88% |
| 121 | REGULATION\_OF\_PROTEIN\_METABOLIC\_PROCESS |  | 150 | -0.21 | -1.19 | 0.120 | 0.493 | 1.000 | 2912 | tags=29%, list=22%, signal=37% |
| 122 | PROTEIN\_AUTOPROCESSING |  | 24 | -0.31 | -1.19 | 0.215 | 0.490 | 1.000 | 4006 | tags=46%, list=31%, signal=66% |
| 123 | CELL\_RECOGNITION |  | 16 | -0.36 | -1.19 | 0.239 | 0.493 | 1.000 | 4600 | tags=56%, list=35%, signal=87% |
| 124 | SKELETAL\_DEVELOPMENT |  | 91 | -0.22 | -1.18 | 0.173 | 0.503 | 1.000 | 2990 | tags=32%, list=23%, signal=41% |
| 125 | BEHAVIOR |  | 136 | -0.21 | -1.18 | 0.147 | 0.504 | 1.000 | 4109 | tags=38%, list=31%, signal=55% |
| 126 | BONE\_REMODELING |  | 28 | -0.30 | -1.17 | 0.237 | 0.510 | 1.000 | 2802 | tags=32%, list=21%, signal=41% |
| 127 | PROTEIN\_AMINO\_ACID\_AUTOPHOSPHORYLATION |  | 24 | -0.31 | -1.17 | 0.228 | 0.507 | 1.000 | 4006 | tags=46%, list=31%, signal=66% |
| 128 | DEVELOPMENTAL\_MATURATION |  | 18 | -0.34 | -1.17 | 0.238 | 0.506 | 1.000 | 3060 | tags=39%, list=23%, signal=51% |
| 129 | REGULATION\_OF\_ORGANELLE\_ORGANIZATION\_AND\_BIOGENESIS |  | 35 | -0.28 | -1.17 | 0.249 | 0.507 | 1.000 | 2599 | tags=31%, list=20%, signal=39% |
| 130 | VASCULATURE\_DEVELOPMENT |  | 50 | -0.25 | -1.17 | 0.197 | 0.506 | 1.000 | 2535 | tags=30%, list=19%, signal=37% |
| 131 | REGULATION\_OF\_PROTEIN\_IMPORT\_INTO\_NUCLEUS |  | 15 | -0.36 | -1.17 | 0.246 | 0.505 | 1.000 | 1374 | tags=27%, list=10%, signal=30% |
| 132 | POSITIVE\_REGULATION\_OF\_T\_CELL\_ACTIVATION |  | 20 | -0.32 | -1.16 | 0.269 | 0.521 | 1.000 | 4560 | tags=55%, list=35%, signal=84% |
| 133 | REGULATION\_OF\_MYELOID\_CELL\_DIFFERENTIATION |  | 19 | -0.33 | -1.16 | 0.249 | 0.517 | 1.000 | 4560 | tags=58%, list=35%, signal=89% |
| 134 | VITAMIN\_METABOLIC\_PROCESS |  | 15 | -0.36 | -1.16 | 0.261 | 0.525 | 1.000 | 4051 | tags=60%, list=31%, signal=87% |
| 135 | POSITIVE\_REGULATION\_OF\_I\_KAPPAB\_KINASE\_NF\_KAPPAB\_CASCADE |  | 67 | -0.23 | -1.15 | 0.200 | 0.529 | 1.000 | 3681 | tags=42%, list=28%, signal=58% |
| 136 | GENERATION\_OF\_PRECURSOR\_METABOLITES\_AND\_ENERGY |  | 120 | -0.21 | -1.15 | 0.182 | 0.526 | 1.000 | 3092 | tags=31%, list=24%, signal=40% |
| 137 | FATTY\_ACID\_OXIDATION |  | 17 | -0.34 | -1.15 | 0.259 | 0.526 | 1.000 | 3282 | tags=47%, list=25%, signal=63% |
| 138 | POSITIVE\_REGULATION\_OF\_PROTEIN\_MODIFICATION\_PROCESS |  | 24 | -0.31 | -1.15 | 0.254 | 0.524 | 1.000 | 1426 | tags=25%, list=11%, signal=28% |
| 139 | REGULATION\_OF\_CELLULAR\_PROTEIN\_METABOLIC\_PROCESS |  | 139 | -0.20 | -1.15 | 0.173 | 0.525 | 1.000 | 2912 | tags=29%, list=22%, signal=37% |
| 140 | STRIATED\_MUSCLE\_DEVELOPMENT |  | 36 | -0.28 | -1.15 | 0.254 | 0.523 | 1.000 | 3551 | tags=44%, list=27%, signal=61% |
| 141 | NEGATIVE\_REGULATION\_OF\_CELL\_PROLIFERATION |  | 145 | -0.20 | -1.15 | 0.160 | 0.526 | 1.000 | 2613 | tags=26%, list=20%, signal=32% |
| 142 | NEGATIVE\_REGULATION\_OF\_RNA\_METABOLIC\_PROCESS |  | 114 | -0.21 | -1.14 | 0.187 | 0.531 | 1.000 | 2821 | tags=29%, list=22%, signal=37% |
| 143 | CYTOKINE\_BIOSYNTHETIC\_PROCESS |  | 34 | -0.28 | -1.14 | 0.255 | 0.530 | 1.000 | 2863 | tags=35%, list=22%, signal=45% |
| 144 | PROTEIN\_SECRETION |  | 28 | -0.28 | -1.13 | 0.282 | 0.540 | 1.000 | 4116 | tags=46%, list=31%, signal=68% |
| 145 | REGULATION\_OF\_MAP\_KINASE\_ACTIVITY |  | 56 | -0.24 | -1.13 | 0.257 | 0.544 | 1.000 | 2198 | tags=29%, list=17%, signal=34% |
| 146 | MYELOID\_CELL\_DIFFERENTIATION |  | 35 | -0.27 | -1.13 | 0.267 | 0.542 | 1.000 | 2903 | tags=31%, list=22%, signal=40% |
| 147 | GLYCOPROTEIN\_BIOSYNTHETIC\_PROCESS |  | 67 | -0.23 | -1.13 | 0.236 | 0.540 | 1.000 | 3632 | tags=37%, list=28%, signal=51% |
| 148 | ACTIVATION\_OF\_MAPK\_ACTIVITY |  | 33 | -0.28 | -1.13 | 0.259 | 0.536 | 1.000 | 2198 | tags=30%, list=17%, signal=36% |
| 149 | NEURON\_DEVELOPMENT |  | 49 | -0.25 | -1.13 | 0.228 | 0.533 | 1.000 | 3407 | tags=33%, list=26%, signal=44% |
| 150 | GLYCEROPHOSPHOLIPID\_METABOLIC\_PROCESS |  | 39 | -0.26 | -1.13 | 0.287 | 0.533 | 1.000 | 4753 | tags=54%, list=36%, signal=84% |
| 151 | NEGATIVE\_REGULATION\_OF\_TRANSCRIPTION\_DNA\_DEPENDENT |  | 114 | -0.21 | -1.13 | 0.230 | 0.533 | 1.000 | 2821 | tags=29%, list=22%, signal=37% |
| 152 | I\_KAPPAB\_KINASE\_NF\_KAPPAB\_CASCADE |  | 88 | -0.22 | -1.13 | 0.223 | 0.533 | 1.000 | 3681 | tags=40%, list=28%, signal=55% |
| 153 | NEGATIVE\_REGULATION\_OF\_NUCLEOBASENUCLEOSIDENUCLEOTIDE\_AND\_NUCLEIC\_ACID\_METABOLIC\_PROCESS |  | 185 | -0.19 | -1.12 | 0.187 | 0.533 | 1.000 | 2737 | tags=28%, list=21%, signal=34% |
| 154 | NEGATIVE\_REGULATION\_OF\_CELL\_DIFFERENTIATION |  | 24 | -0.30 | -1.12 | 0.297 | 0.533 | 1.000 | 2737 | tags=29%, list=21%, signal=37% |
| 155 | RESPONSE\_TO\_BIOTIC\_STIMULUS |  | 103 | -0.21 | -1.12 | 0.238 | 0.532 | 1.000 | 2809 | tags=31%, list=21%, signal=39% |
| 156 | POST\_TRANSLATIONAL\_PROTEIN\_MODIFICATION |  | 409 | -0.17 | -1.12 | 0.120 | 0.531 | 1.000 | 2985 | tags=26%, list=23%, signal=33% |
| 157 | ORGANIC\_ACID\_METABOLIC\_PROCESS |  | 162 | -0.19 | -1.12 | 0.210 | 0.528 | 1.000 | 4041 | tags=39%, list=31%, signal=56% |
| 158 | CARBOXYLIC\_ACID\_METABOLIC\_PROCESS |  | 160 | -0.19 | -1.12 | 0.205 | 0.525 | 1.000 | 4041 | tags=39%, list=31%, signal=56% |
| 159 | DEFENSE\_RESPONSE\_TO\_BACTERIUM |  | 16 | -0.34 | -1.12 | 0.313 | 0.522 | 1.000 | 4013 | tags=44%, list=31%, signal=63% |
| 160 | REGULATION\_OF\_MAPKKK\_CASCADE |  | 19 | -0.33 | -1.12 | 0.311 | 0.522 | 1.000 | 2441 | tags=32%, list=19%, signal=39% |
| 161 | ION\_HOMEOSTASIS |  | 112 | -0.21 | -1.12 | 0.219 | 0.519 | 1.000 | 3181 | tags=32%, list=24%, signal=42% |
| 162 | MEMBRANE\_ORGANIZATION\_AND\_BIOGENESIS |  | 124 | -0.20 | -1.12 | 0.222 | 0.517 | 1.000 | 1974 | tags=23%, list=15%, signal=26% |
| 163 | AMINO\_ACID\_CATABOLIC\_PROCESS |  | 23 | -0.30 | -1.12 | 0.297 | 0.523 | 1.000 | 2438 | tags=35%, list=19%, signal=43% |
| 164 | AMINO\_ACID\_DERIVATIVE\_METABOLIC\_PROCESS |  | 23 | -0.30 | -1.11 | 0.287 | 0.521 | 1.000 | 4002 | tags=48%, list=31%, signal=69% |
| 165 | CYTOKINE\_SECRETION |  | 15 | -0.35 | -1.11 | 0.318 | 0.527 | 1.000 | 3432 | tags=47%, list=26%, signal=63% |
| 166 | REGULATION\_OF\_DEVELOPMENTAL\_PROCESS |  | 387 | -0.17 | -1.11 | 0.165 | 0.528 | 1.000 | 4406 | tags=43%, list=34%, signal=63% |
| 167 | NEGATIVE\_REGULATION\_OF\_METABOLIC\_PROCESS |  | 232 | -0.18 | -1.11 | 0.205 | 0.525 | 1.000 | 2737 | tags=26%, list=21%, signal=32% |
| 168 | TISSUE\_REMODELING |  | 29 | -0.28 | -1.11 | 0.307 | 0.525 | 1.000 | 2802 | tags=31%, list=21%, signal=39% |
| 169 | CELL\_MATURATION |  | 16 | -0.33 | -1.10 | 0.334 | 0.542 | 1.000 | 3060 | tags=38%, list=23%, signal=49% |
| 170 | PHAGOCYTOSIS |  | 16 | -0.34 | -1.10 | 0.309 | 0.542 | 1.000 | 4139 | tags=56%, list=32%, signal=82% |
| 171 | NEURITE\_DEVELOPMENT |  | 41 | -0.25 | -1.09 | 0.325 | 0.572 | 1.000 | 3407 | tags=32%, list=26%, signal=43% |
| 172 | CYTOKINE\_METABOLIC\_PROCESS |  | 35 | -0.26 | -1.08 | 0.314 | 0.580 | 1.000 | 2863 | tags=34%, list=22%, signal=44% |
| 173 | LOCOMOTORY\_BEHAVIOR |  | 84 | -0.21 | -1.08 | 0.310 | 0.581 | 1.000 | 2809 | tags=29%, list=21%, signal=36% |
| 174 | ELECTRON\_TRANSPORT\_GO\_0006118 |  | 50 | -0.24 | -1.08 | 0.308 | 0.581 | 1.000 | 2227 | tags=26%, list=17%, signal=31% |
| 175 | SODIUM\_ION\_TRANSPORT |  | 17 | -0.32 | -1.08 | 0.348 | 0.586 | 1.000 | 5046 | tags=59%, list=39%, signal=96% |
| 176 | REGULATION\_OF\_BIOLOGICAL\_QUALITY |  | 364 | -0.17 | -1.07 | 0.239 | 0.583 | 1.000 | 3616 | tags=30%, list=28%, signal=41% |
| 177 | TISSUE\_DEVELOPMENT |  | 126 | -0.20 | -1.07 | 0.295 | 0.584 | 1.000 | 1579 | tags=19%, list=12%, signal=21% |
| 178 | CELLULAR\_LIPID\_METABOLIC\_PROCESS |  | 220 | -0.18 | -1.07 | 0.271 | 0.583 | 1.000 | 3890 | tags=37%, list=30%, signal=52% |
| 179 | POSITIVE\_REGULATION\_OF\_DEVELOPMENTAL\_PROCESS |  | 197 | -0.18 | -1.07 | 0.292 | 0.580 | 1.000 | 4084 | tags=42%, list=31%, signal=60% |
| 180 | MEMBRANE\_LIPID\_METABOLIC\_PROCESS |  | 85 | -0.21 | -1.07 | 0.324 | 0.584 | 1.000 | 3775 | tags=39%, list=29%, signal=54% |
| 181 | NEGATIVE\_REGULATION\_OF\_CELLULAR\_METABOLIC\_PROCESS |  | 229 | -0.18 | -1.07 | 0.254 | 0.581 | 1.000 | 2737 | tags=26%, list=21%, signal=32% |
| 182 | NEGATIVE\_REGULATION\_OF\_TRANSCRIPTION\_FROM\_RNA\_POLYMERASE\_II\_PROMOTER |  | 76 | -0.21 | -1.07 | 0.329 | 0.586 | 1.000 | 2821 | tags=29%, list=22%, signal=37% |
| 183 | DETECTION\_OF\_EXTERNAL\_STIMULUS |  | 18 | -0.31 | -1.06 | 0.373 | 0.589 | 1.000 | 8986 | tags=100%, list=69%, signal=318% |
| 184 | AMINO\_ACID\_METABOLIC\_PROCESS |  | 73 | -0.21 | -1.06 | 0.330 | 0.598 | 1.000 | 2218 | tags=26%, list=17%, signal=31% |
| 185 | AXONOGENESIS |  | 33 | -0.26 | -1.06 | 0.374 | 0.595 | 1.000 | 3407 | tags=33%, list=26%, signal=45% |
| 186 | HORMONE\_METABOLIC\_PROCESS |  | 29 | -0.27 | -1.06 | 0.347 | 0.594 | 1.000 | 3890 | tags=48%, list=30%, signal=69% |
| 187 | AMINE\_CATABOLIC\_PROCESS |  | 25 | -0.28 | -1.06 | 0.361 | 0.591 | 1.000 | 2438 | tags=32%, list=19%, signal=39% |
| 188 | CELL\_PROLIFERATION\_GO\_0008283 |  | 466 | -0.16 | -1.05 | 0.279 | 0.604 | 1.000 | 2613 | tags=24%, list=20%, signal=28% |
| 189 | MYOBLAST\_DIFFERENTIATION |  | 16 | -0.31 | -1.05 | 0.383 | 0.610 | 1.000 | 3392 | tags=50%, list=26%, signal=67% |
| 190 | CELL\_MIGRATION |  | 82 | -0.21 | -1.05 | 0.363 | 0.608 | 1.000 | 2599 | tags=24%, list=20%, signal=30% |
| 191 | POSITIVE\_REGULATION\_OF\_METABOLIC\_PROCESS |  | 201 | -0.17 | -1.05 | 0.352 | 0.607 | 1.000 | 2377 | tags=23%, list=18%, signal=28% |
| 192 | ACTIN\_FILAMENT\_ORGANIZATION |  | 21 | -0.28 | -1.04 | 0.410 | 0.613 | 1.000 | 1883 | tags=29%, list=14%, signal=33% |
| 193 | POSITIVE\_REGULATION\_OF\_CELLULAR\_METABOLIC\_PROCESS |  | 196 | -0.18 | -1.04 | 0.331 | 0.611 | 1.000 | 2377 | tags=23%, list=18%, signal=28% |
| 194 | POSITIVE\_REGULATION\_OF\_TRANSCRIPTION |  | 124 | -0.19 | -1.04 | 0.343 | 0.609 | 1.000 | 3400 | tags=31%, list=26%, signal=41% |
| 195 | PEPTIDYL\_AMINO\_ACID\_MODIFICATION |  | 47 | -0.23 | -1.04 | 0.374 | 0.613 | 1.000 | 2599 | tags=28%, list=20%, signal=34% |
| 196 | POSITIVE\_REGULATION\_OF\_MAP\_KINASE\_ACTIVITY |  | 39 | -0.25 | -1.04 | 0.386 | 0.615 | 1.000 | 2198 | tags=28%, list=17%, signal=34% |
| 197 | HEART\_DEVELOPMENT |  | 33 | -0.26 | -1.04 | 0.382 | 0.615 | 1.000 | 3928 | tags=39%, list=30%, signal=56% |
| 198 | POSITIVE\_REGULATION\_OF\_CATALYTIC\_ACTIVITY |  | 139 | -0.18 | -1.03 | 0.381 | 0.621 | 1.000 | 2429 | tags=22%, list=19%, signal=27% |
| 199 | NITROGEN\_COMPOUND\_CATABOLIC\_PROCESS |  | 27 | -0.27 | -1.03 | 0.393 | 0.620 | 1.000 | 2438 | tags=30%, list=19%, signal=36% |
| 200 | DETECTION\_OF\_STIMULUS\_INVOLVED\_IN\_SENSORY\_PERCEPTION |  | 15 | -0.31 | -1.03 | 0.430 | 0.625 | 1.000 | 8986 | tags=100%, list=69%, signal=319% |
| 201 | MAPKKK\_CASCADE\_GO\_0000165 |  | 90 | -0.20 | -1.02 | 0.410 | 0.636 | 1.000 | 1984 | tags=21%, list=15%, signal=25% |
| 202 | TRANSLATION |  | 149 | -0.18 | -1.02 | 0.417 | 0.635 | 1.000 | 2912 | tags=29%, list=22%, signal=37% |
| 203 | NEUROGENESIS |  | 75 | -0.20 | -1.02 | 0.441 | 0.646 | 1.000 | 3616 | tags=33%, list=28%, signal=46% |
| 204 | ANATOMICAL\_STRUCTURE\_MORPHOGENESIS |  | 336 | -0.16 | -1.02 | 0.400 | 0.646 | 1.000 | 3407 | tags=29%, list=26%, signal=39% |
| 205 | RESPONSE\_TO\_CHEMICAL\_STIMULUS |  | 271 | -0.16 | -1.01 | 0.411 | 0.653 | 1.000 | 1824 | tags=18%, list=14%, signal=21% |
| 206 | NERVOUS\_SYSTEM\_DEVELOPMENT |  | 328 | -0.16 | -1.00 | 0.437 | 0.675 | 1.000 | 4050 | tags=33%, list=31%, signal=47% |
| 207 | CELLULAR\_COMPONENT\_ASSEMBLY |  | 272 | -0.16 | -1.00 | 0.428 | 0.678 | 1.000 | 3107 | tags=28%, list=24%, signal=36% |
| 208 | REGULATION\_OF\_TRANSCRIPTION |  | 498 | -0.15 | -1.00 | 0.435 | 0.675 | 1.000 | 3324 | tags=29%, list=25%, signal=37% |
| 209 | AXON\_GUIDANCE |  | 18 | -0.29 | -1.00 | 0.441 | 0.682 | 1.000 | 2837 | tags=33%, list=22%, signal=42% |
| 210 | POSITIVE\_REGULATION\_OF\_TRANSCRIPTION\_FACTOR\_ACTIVITY |  | 17 | -0.29 | -1.00 | 0.446 | 0.679 | 1.000 | 4206 | tags=53%, list=32%, signal=78% |
| 211 | MACROMOLECULE\_BIOSYNTHETIC\_PROCESS |  | 267 | -0.16 | -1.00 | 0.466 | 0.676 | 1.000 | 2990 | tags=27%, list=23%, signal=34% |
| 212 | ANTI\_APOPTOSIS |  | 107 | -0.18 | -0.99 | 0.443 | 0.685 | 1.000 | 2229 | tags=25%, list=17%, signal=30% |
| 213 | ORGANIC\_ACID\_TRANSPORT |  | 39 | -0.23 | -0.99 | 0.455 | 0.688 | 1.000 | 2282 | tags=26%, list=17%, signal=31% |
| 214 | REGULATION\_OF\_CYTOKINE\_BIOSYNTHETIC\_PROCESS |  | 31 | -0.24 | -0.99 | 0.444 | 0.685 | 1.000 | 2863 | tags=32%, list=22%, signal=41% |
| 215 | REGULATION\_OF\_JNK\_ACTIVITY |  | 18 | -0.29 | -0.99 | 0.464 | 0.689 | 1.000 | 1952 | tags=28%, list=15%, signal=33% |
| 216 | CARBOXYLIC\_ACID\_TRANSPORT |  | 39 | -0.23 | -0.99 | 0.468 | 0.689 | 1.000 | 2282 | tags=26%, list=17%, signal=31% |
| 217 | AMINO\_ACID\_AND\_DERIVATIVE\_METABOLIC\_PROCESS |  | 96 | -0.19 | -0.99 | 0.475 | 0.690 | 1.000 | 2438 | tags=25%, list=19%, signal=30% |
| 218 | REGULATION\_OF\_PROTEIN\_SECRETION |  | 19 | -0.28 | -0.98 | 0.484 | 0.711 | 1.000 | 4084 | tags=47%, list=31%, signal=69% |
| 219 | RHYTHMIC\_PROCESS |  | 23 | -0.26 | -0.98 | 0.502 | 0.710 | 1.000 | 2051 | tags=26%, list=16%, signal=31% |
| 220 | POSITIVE\_REGULATION\_OF\_DNA\_BINDING |  | 18 | -0.28 | -0.97 | 0.479 | 0.713 | 1.000 | 4509 | tags=56%, list=34%, signal=85% |
| 221 | CHEMICAL\_HOMEOSTASIS |  | 136 | -0.17 | -0.97 | 0.521 | 0.729 | 1.000 | 3181 | tags=29%, list=24%, signal=37% |
| 222 | NEGATIVE\_REGULATION\_OF\_DEVELOPMENTAL\_PROCESS |  | 177 | -0.17 | -0.96 | 0.533 | 0.730 | 1.000 | 2863 | tags=27%, list=22%, signal=34% |
| 223 | POSITIVE\_REGULATION\_OF\_TRANSCRIPTION\_FROM\_RNA\_POLYMERASE\_II\_PROMOTER |  | 60 | -0.20 | -0.95 | 0.535 | 0.760 | 1.000 | 3273 | tags=32%, list=25%, signal=42% |
| 224 | CELLULAR\_PROTEIN\_COMPLEX\_ASSEMBLY |  | 28 | -0.24 | -0.95 | 0.536 | 0.761 | 1.000 | 2764 | tags=29%, list=21%, signal=36% |
| 225 | POSITIVE\_REGULATION\_OF\_NUCLEOBASENUCLEOSIDENUCLEOTIDE\_AND\_NUCLEIC\_ACID\_METABOLIC\_PROCESS |  | 134 | -0.17 | -0.95 | 0.548 | 0.763 | 1.000 | 3113 | tags=28%, list=24%, signal=36% |
| 226 | MACROMOLECULAR\_COMPLEX\_ASSEMBLY |  | 254 | -0.15 | -0.95 | 0.607 | 0.762 | 1.000 | 3292 | tags=29%, list=25%, signal=38% |
| 227 | CELLULAR\_HOMEOSTASIS |  | 121 | -0.17 | -0.95 | 0.582 | 0.761 | 1.000 | 4342 | tags=41%, list=33%, signal=61% |
| 228 | CELL\_CELL\_ADHESION |  | 72 | -0.19 | -0.95 | 0.549 | 0.759 | 1.000 | 4325 | tags=42%, list=33%, signal=62% |
| 229 | SECRETION\_BY\_CELL |  | 100 | -0.18 | -0.95 | 0.573 | 0.757 | 1.000 | 4406 | tags=42%, list=34%, signal=63% |
| 230 | REGULATION\_OF\_G\_PROTEIN\_COUPLED\_RECEPTOR\_PROTEIN\_SIGNALING\_PATHWAY |  | 23 | -0.26 | -0.94 | 0.537 | 0.765 | 1.000 | 1070 | tags=17%, list=8%, signal=19% |
| 231 | SKELETAL\_MUSCLE\_DEVELOPMENT |  | 28 | -0.24 | -0.93 | 0.560 | 0.780 | 1.000 | 3551 | tags=43%, list=27%, signal=59% |
| 232 | INSULIN\_RECEPTOR\_SIGNALING\_PATHWAY |  | 16 | -0.28 | -0.93 | 0.521 | 0.783 | 1.000 | 3159 | tags=38%, list=24%, signal=49% |
| 233 | EPIDERMIS\_DEVELOPMENT |  | 66 | -0.19 | -0.93 | 0.578 | 0.790 | 1.000 | 1579 | tags=20%, list=12%, signal=22% |
| 234 | CYCLIC\_NUCLEOTIDE\_MEDIATED\_SIGNALING |  | 97 | -0.18 | -0.93 | 0.599 | 0.789 | 1.000 | 1256 | tags=12%, list=10%, signal=14% |
| 235 | RESPONSE\_TO\_OXIDATIVE\_STRESS |  | 38 | -0.22 | -0.92 | 0.586 | 0.793 | 1.000 | 1067 | tags=21%, list=8%, signal=23% |
| 236 | HOMEOSTATIC\_PROCESS |  | 179 | -0.16 | -0.92 | 0.671 | 0.796 | 1.000 | 3245 | tags=28%, list=25%, signal=37% |
| 237 | ACTIVATION\_OF\_PROTEIN\_KINASE\_ACTIVITY |  | 23 | -0.25 | -0.92 | 0.522 | 0.798 | 1.000 | 4384 | tags=39%, list=33%, signal=59% |
| 238 | G\_PROTEIN\_SIGNALING\_COUPLED\_TO\_CYCLIC\_NUCLEOTIDE\_SECOND\_MESSENGER |  | 96 | -0.18 | -0.91 | 0.676 | 0.832 | 1.000 | 1256 | tags=13%, list=10%, signal=14% |
| 239 | RESPONSE\_TO\_NUTRIENT |  | 17 | -0.27 | -0.91 | 0.576 | 0.829 | 1.000 | 411 | tags=18%, list=3%, signal=18% |
| 240 | ENDOSOME\_TRANSPORT |  | 22 | -0.25 | -0.90 | 0.586 | 0.843 | 1.000 | 2225 | tags=27%, list=17%, signal=33% |
| 241 | ECTODERM\_DEVELOPMENT |  | 75 | -0.18 | -0.90 | 0.643 | 0.844 | 1.000 | 1579 | tags=19%, list=12%, signal=21% |
| 242 | AMINE\_METABOLIC\_PROCESS |  | 128 | -0.16 | -0.89 | 0.715 | 0.866 | 1.000 | 4041 | tags=36%, list=31%, signal=51% |
| 243 | CELL\_CELL\_SIGNALING |  | 372 | -0.14 | -0.88 | 0.853 | 0.874 | 1.000 | 4115 | tags=33%, list=31%, signal=46% |
| 244 | REGULATION\_OF\_PROTEIN\_MODIFICATION\_PROCESS |  | 37 | -0.21 | -0.88 | 0.647 | 0.876 | 1.000 | 1531 | tags=19%, list=12%, signal=21% |
| 245 | REGULATION\_OF\_CELLULAR\_COMPONENT\_ORGANIZATION\_AND\_BIOGENESIS |  | 102 | -0.17 | -0.87 | 0.766 | 0.887 | 1.000 | 3656 | tags=32%, list=28%, signal=45% |
| 246 | POSITIVE\_REGULATION\_OF\_CELLULAR\_COMPONENT\_ORGANIZATION\_AND\_BIOGENESIS |  | 28 | -0.22 | -0.87 | 0.672 | 0.884 | 1.000 | 4191 | tags=43%, list=32%, signal=63% |
| 247 | REGULATION\_OF\_TRANSLATION |  | 76 | -0.18 | -0.87 | 0.741 | 0.882 | 1.000 | 2912 | tags=28%, list=22%, signal=35% |
| 248 | PHOSPHOINOSITIDE\_METABOLIC\_PROCESS |  | 25 | -0.23 | -0.87 | 0.655 | 0.880 | 1.000 | 4753 | tags=56%, list=36%, signal=88% |
| 249 | REGULATION\_OF\_TRANSLATIONAL\_INITIATION |  | 25 | -0.23 | -0.87 | 0.656 | 0.881 | 1.000 | 1156 | tags=20%, list=9%, signal=22% |
| 250 | CELLULAR\_MORPHOGENESIS\_DURING\_DIFFERENTIATION |  | 38 | -0.20 | -0.87 | 0.672 | 0.882 | 1.000 | 3407 | tags=29%, list=26%, signal=39% |
| 251 | REGULATION\_OF\_BINDING |  | 46 | -0.19 | -0.87 | 0.685 | 0.883 | 1.000 | 1766 | tags=22%, list=13%, signal=25% |
| 252 | REGULATION\_OF\_MUSCLE\_CONTRACTION |  | 18 | -0.26 | -0.87 | 0.634 | 0.881 | 1.000 | 3032 | tags=39%, list=23%, signal=51% |
| 253 | REGULATION\_OF\_GROWTH |  | 48 | -0.19 | -0.86 | 0.717 | 0.892 | 1.000 | 4096 | tags=40%, list=31%, signal=57% |
| 254 | SPHINGOLIPID\_METABOLIC\_PROCESS |  | 23 | -0.23 | -0.86 | 0.686 | 0.894 | 1.000 | 3245 | tags=35%, list=25%, signal=46% |
| 255 | SULFUR\_METABOLIC\_PROCESS |  | 30 | -0.21 | -0.85 | 0.722 | 0.915 | 1.000 | 2918 | tags=30%, list=22%, signal=39% |
| 256 | VESICLE\_MEDIATED\_TRANSPORT |  | 174 | -0.14 | -0.84 | 0.868 | 0.921 | 1.000 | 2252 | tags=20%, list=17%, signal=23% |
| 257 | RESPONSE\_TO\_NUTRIENT\_LEVELS |  | 27 | -0.21 | -0.84 | 0.683 | 0.919 | 1.000 | 2399 | tags=26%, list=18%, signal=32% |
| 258 | NEGATIVE\_REGULATION\_OF\_CELLULAR\_COMPONENT\_ORGANIZATION\_AND\_BIOGENESIS |  | 26 | -0.22 | -0.83 | 0.727 | 0.936 | 1.000 | 1883 | tags=19%, list=14%, signal=22% |
| 259 | SECOND\_MESSENGER\_MEDIATED\_SIGNALING |  | 139 | -0.15 | -0.83 | 0.879 | 0.933 | 1.000 | 1362 | tags=12%, list=10%, signal=14% |
| 260 | CENTRAL\_NERVOUS\_SYSTEM\_DEVELOPMENT |  | 105 | -0.15 | -0.82 | 0.861 | 0.949 | 1.000 | 3830 | tags=32%, list=29%, signal=45% |
| 261 | POSITIVE\_REGULATION\_OF\_CASPASE\_ACTIVITY |  | 28 | -0.21 | -0.81 | 0.735 | 0.956 | 1.000 | 1917 | tags=25%, list=15%, signal=29% |
| 262 | PROTEIN\_HOMOOLIGOMERIZATION |  | 19 | -0.24 | -0.81 | 0.720 | 0.952 | 1.000 | 2307 | tags=26%, list=18%, signal=32% |
| 263 | POSITIVE\_REGULATION\_OF\_BINDING |  | 19 | -0.23 | -0.80 | 0.738 | 0.964 | 1.000 | 4509 | tags=53%, list=34%, signal=80% |
| 264 | NITROGEN\_COMPOUND\_METABOLIC\_PROCESS |  | 141 | -0.14 | -0.80 | 0.899 | 0.963 | 1.000 | 2990 | tags=25%, list=23%, signal=32% |
| 265 | EXTRACELLULAR\_STRUCTURE\_ORGANIZATION\_AND\_BIOGENESIS |  | 23 | -0.22 | -0.80 | 0.727 | 0.961 | 1.000 | 3551 | tags=39%, list=27%, signal=54% |
| 266 | GOLGI\_VESICLE\_TRANSPORT |  | 42 | -0.18 | -0.80 | 0.803 | 0.961 | 1.000 | 4378 | tags=43%, list=33%, signal=64% |
| 267 | T\_CELL\_PROLIFERATION |  | 17 | -0.24 | -0.79 | 0.742 | 0.967 | 1.000 | 4824 | tags=59%, list=37%, signal=93% |
| 268 | REGULATION\_OF\_TRANSCRIPTION\_FACTOR\_ACTIVITY |  | 30 | -0.20 | -0.79 | 0.808 | 0.968 | 1.000 | 4430 | tags=47%, list=34%, signal=70% |
| 269 | REGULATION\_OF\_CELL\_MIGRATION |  | 23 | -0.22 | -0.79 | 0.774 | 0.966 | 1.000 | 4981 | tags=48%, list=38%, signal=77% |
| 270 | POSITIVE\_REGULATION\_OF\_JNK\_ACTIVITY |  | 16 | -0.23 | -0.78 | 0.754 | 0.974 | 1.000 | 1952 | tags=25%, list=15%, signal=29% |
| 271 | POSITIVE\_REGULATION\_OF\_TRANSCRIPTIONDNA\_DEPENDENT |  | 105 | -0.15 | -0.78 | 0.924 | 0.970 | 1.000 | 4560 | tags=40%, list=35%, signal=61% |
| 272 | PATTERN\_SPECIFICATION\_PROCESS |  | 27 | -0.19 | -0.78 | 0.813 | 0.977 | 1.000 | 5537 | tags=63%, list=42%, signal=109% |
| 273 | REPRODUCTIVE\_PROCESS |  | 133 | -0.14 | -0.77 | 0.947 | 0.986 | 1.000 | 4099 | tags=35%, list=31%, signal=51% |
| 274 | AMINE\_BIOSYNTHETIC\_PROCESS |  | 15 | -0.24 | -0.77 | 0.763 | 0.983 | 1.000 | 436 | tags=13%, list=3%, signal=14% |
| 275 | METAL\_ION\_TRANSPORT |  | 102 | -0.14 | -0.77 | 0.928 | 0.983 | 1.000 | 5295 | tags=49%, list=40%, signal=82% |
| 276 | TRANSLATIONAL\_INITIATION |  | 33 | -0.19 | -0.76 | 0.840 | 0.981 | 1.000 | 1271 | tags=18%, list=10%, signal=20% |
| 277 | G\_PROTEIN\_SIGNALING\_COUPLED\_TO\_IP3\_SECOND\_MESSENGERPHOSPHOLIPASE\_C\_ACTIVATING |  | 39 | -0.18 | -0.76 | 0.867 | 0.977 | 1.000 | 2624 | tags=23%, list=20%, signal=29% |
| 278 | REGULATION\_OF\_DNA\_BINDING |  | 36 | -0.18 | -0.76 | 0.824 | 0.978 | 1.000 | 2651 | tags=28%, list=20%, signal=35% |
| 279 | RESPONSE\_TO\_EXTRACELLULAR\_STIMULUS |  | 29 | -0.19 | -0.76 | 0.866 | 0.977 | 1.000 | 2399 | tags=24%, list=18%, signal=29% |
| 280 | EXCRETION |  | 35 | -0.18 | -0.75 | 0.855 | 0.984 | 1.000 | 2140 | tags=20%, list=16%, signal=24% |
| 281 | G\_PROTEIN\_COUPLED\_RECEPTOR\_PROTEIN\_SIGNALING\_PATHWAY |  | 300 | -0.12 | -0.75 | 0.997 | 0.984 | 1.000 | 4659 | tags=35%, list=36%, signal=53% |
| 282 | SECRETION |  | 157 | -0.13 | -0.74 | 0.978 | 0.989 | 1.000 | 4406 | tags=38%, list=34%, signal=56% |
| 283 | POSITIVE\_REGULATION\_OF\_RNA\_METABOLIC\_PROCESS |  | 107 | -0.14 | -0.74 | 0.963 | 0.993 | 1.000 | 4560 | tags=39%, list=35%, signal=60% |
| 284 | CARBOHYDRATE\_METABOLIC\_PROCESS |  | 152 | -0.13 | -0.74 | 0.975 | 0.990 | 1.000 | 3862 | tags=31%, list=30%, signal=43% |
| 285 | REGULATION\_OF\_SECRETION |  | 35 | -0.18 | -0.73 | 0.866 | 0.992 | 1.000 | 1284 | tags=17%, list=10%, signal=19% |
| 286 | POTASSIUM\_ION\_TRANSPORT |  | 52 | -0.16 | -0.73 | 0.902 | 0.990 | 1.000 | 5295 | tags=50%, list=40%, signal=84% |
| 287 | NEGATIVE\_REGULATION\_OF\_GROWTH |  | 35 | -0.18 | -0.73 | 0.883 | 0.987 | 1.000 | 4096 | tags=40%, list=31%, signal=58% |
| 288 | PROTEIN\_POLYMERIZATION |  | 17 | -0.21 | -0.72 | 0.858 | 0.994 | 1.000 | 290 | tags=12%, list=2%, signal=12% |
| 289 | DI\_\_\_TRI\_VALENT\_INORGANIC\_CATION\_TRANSPORT |  | 27 | -0.18 | -0.72 | 0.891 | 0.992 | 1.000 | 1115 | tags=15%, list=9%, signal=16% |
| 290 | SECRETORY\_PATHWAY |  | 72 | -0.14 | -0.71 | 0.948 | 0.995 | 1.000 | 4406 | tags=40%, list=34%, signal=60% |
| 291 | G\_PROTEIN\_SIGNALING\_ADENYLATE\_CYCLASE\_ACTIVATING\_PATHWAY |  | 24 | -0.19 | -0.71 | 0.865 | 0.992 | 1.000 | 1256 | tags=13%, list=10%, signal=14% |
| 292 | PROTEIN\_AMINO\_ACID\_LIPIDATION |  | 21 | -0.20 | -0.71 | 0.875 | 0.993 | 1.000 | 4567 | tags=52%, list=35%, signal=80% |
| 293 | NEGATIVE\_REGULATION\_OF\_CELLULAR\_PROTEIN\_METABOLIC\_PROCESS |  | 41 | -0.16 | -0.70 | 0.946 | 0.995 | 1.000 | 2909 | tags=24%, list=22%, signal=31% |
| 294 | LIPID\_HOMEOSTASIS |  | 15 | -0.21 | -0.70 | 0.861 | 0.993 | 1.000 | 672 | tags=13%, list=5%, signal=14% |
| 295 | ESTABLISHMENT\_AND\_OR\_MAINTENANCE\_OF\_CELL\_POLARITY |  | 19 | -0.20 | -0.69 | 0.896 | 0.998 | 1.000 | 3877 | tags=32%, list=30%, signal=45% |
| 296 | CATION\_TRANSPORT |  | 130 | -0.12 | -0.68 | 0.991 | 1.000 | 1.000 | 5087 | tags=44%, list=39%, signal=71% |
| 297 | NUCLEOTIDE\_EXCISION\_REPAIR |  | 19 | -0.19 | -0.68 | 0.920 | 0.997 | 1.000 | 2081 | tags=21%, list=16%, signal=25% |
| 298 | PHOSPHOINOSITIDE\_MEDIATED\_SIGNALING |  | 42 | -0.16 | -0.67 | 0.937 | 0.999 | 1.000 | 2624 | tags=21%, list=20%, signal=27% |
| 299 | INORGANIC\_ANION\_TRANSPORT |  | 16 | -0.20 | -0.67 | 0.896 | 0.997 | 1.000 | 601 | tags=13%, list=5%, signal=13% |
| 300 | POSITIVE\_REGULATION\_OF\_TRANSPORT |  | 18 | -0.19 | -0.66 | 0.902 | 0.998 | 1.000 | 5033 | tags=56%, list=38%, signal=90% |
| 301 | CARBOHYDRATE\_BIOSYNTHETIC\_PROCESS |  | 35 | -0.16 | -0.65 | 0.934 | 1.000 | 1.000 | 5289 | tags=51%, list=40%, signal=86% |
| 302 | ION\_TRANSPORT |  | 165 | -0.11 | -0.65 | 1.000 | 0.998 | 1.000 | 5099 | tags=43%, list=39%, signal=70% |
| 303 | NEGATIVE\_REGULATION\_OF\_PROTEIN\_METABOLIC\_PROCESS |  | 44 | -0.15 | -0.65 | 0.965 | 0.995 | 1.000 | 2909 | tags=23%, list=22%, signal=29% |
| 304 | REGULATION\_OF\_CYTOKINE\_PRODUCTION |  | 21 | -0.18 | -0.65 | 0.926 | 0.995 | 1.000 | 2515 | tags=24%, list=19%, signal=29% |
| 305 | NEGATIVE\_REGULATION\_OF\_MULTICELLULAR\_ORGANISMAL\_PROCESS |  | 27 | -0.17 | -0.64 | 0.925 | 0.993 | 1.000 | 2215 | tags=22%, list=17%, signal=27% |
| 306 | CARBOHYDRATE\_CATABOLIC\_PROCESS |  | 20 | -0.18 | -0.63 | 0.942 | 0.997 | 1.000 | 4282 | tags=35%, list=33%, signal=52% |
| 307 | CELLULAR\_CARBOHYDRATE\_CATABOLIC\_PROCESS |  | 20 | -0.18 | -0.63 | 0.923 | 0.994 | 1.000 | 4282 | tags=35%, list=33%, signal=52% |
| 308 | CALCIUM\_ION\_TRANSPORT |  | 23 | -0.16 | -0.57 | 0.966 | 1.000 | 1.000 | 1115 | tags=13%, list=9%, signal=14% |
| 309 | FEMALE\_GAMETE\_GENERATION |  | 15 | -0.18 | -0.56 | 0.972 | 1.000 | 1.000 | 10802 | tags=100%, list=83%, signal=571% |
| 310 | AMINO\_SUGAR\_METABOLIC\_PROCESS |  | 15 | -0.17 | -0.56 | 0.972 | 1.000 | 1.000 | 3733 | tags=33%, list=29%, signal=47% |
| 311 | REGULATION\_OF\_CELL\_GROWTH |  | 39 | -0.13 | -0.56 | 0.993 | 1.000 | 1.000 | 4096 | tags=33%, list=31%, signal=48% |
| 312 | REGULATION\_OF\_HEART\_CONTRACTION |  | 24 | -0.15 | -0.54 | 0.994 | 1.000 | 1.000 | 11186 | tags=100%, list=85%, signal=686% |
| 313 | REGULATION\_OF\_ACTION\_POTENTIAL |  | 16 | -0.16 | -0.52 | 0.986 | 1.000 | 1.000 | 3604 | tags=31%, list=28%, signal=43% |
| 314 | MONOVALENT\_INORGANIC\_CATION\_TRANSPORT |  | 83 | -0.10 | -0.52 | 1.000 | 0.998 | 1.000 | 5295 | tags=46%, list=40%, signal=76% |
| 315 | PEROXISOME\_ORGANIZATION\_AND\_BIOGENESIS |  | 15 | -0.16 | -0.50 | 0.986 | 0.996 | 1.000 | 4964 | tags=47%, list=38%, signal=75% |
Table: Gene sets enriched in phenotype **na**[plain text format]****

  
