# Supplementary material for: Multiple-input multiple-output causal strategies for gene selection
Source: BMC Bioinformatics. 2011 Nov 25;12:458. doi: 10.1186/1471-2105-12-458 (PMC3323860; doi:10.1186/1471-2105-12-458)
Supplement: Additional file 2 — Archive containing the output files computed by the preranked GSEA for λ ∈ {0.1,0.2,0.3,0.4,0.5} (GSEA_MIMO_part1.zip). [file 1471-2105-12-458-S2.ZIP › mFS04_entrez_mimo.GseaPreranked.1316038376454/gsea_report_for_na_pos_1316038376454.html]

Report for na\_pos 1316038376454 [GSEA]

| GS  follow link to MSigDB | GS DETAILS | SIZE | ES | NES | NOM p-val | FDR q-val | FWER p-val | RANK AT MAX | LEADING EDGE || 1 | M\_PHASE\_OF\_MITOTIC\_CELL\_CYCLE |  | 72 | 0.58 | 2.75 | 0.000 | 0.000 | 0.000 | 2211 | tags=56%, list=17%, signal=66% |
| 2 | M\_PHASE |  | 98 | 0.54 | 2.74 | 0.000 | 0.000 | 0.000 | 2460 | tags=53%, list=19%, signal=65% |
| 3 | MITOTIC\_CELL\_CYCLE |  | 134 | 0.51 | 2.73 | 0.000 | 0.000 | 0.000 | 2746 | tags=52%, list=21%, signal=65% |
| 4 | MITOSIS |  | 70 | 0.58 | 2.73 | 0.000 | 0.000 | 0.000 | 2211 | tags=54%, list=17%, signal=65% |
| 5 | CELL\_CYCLE\_PROCESS |  | 169 | 0.49 | 2.71 | 0.000 | 0.000 | 0.000 | 2746 | tags=50%, list=21%, signal=63% |
| 6 | CELL\_CYCLE\_PHASE |  | 152 | 0.47 | 2.58 | 0.000 | 0.000 | 0.000 | 2746 | tags=49%, list=21%, signal=61% |
| 7 | DNA\_REPLICATION |  | 97 | 0.50 | 2.55 | 0.000 | 0.000 | 0.000 | 2803 | tags=48%, list=21%, signal=61% |
| 8 | SISTER\_CHROMATID\_SEGREGATION |  | 16 | 0.75 | 2.44 | 0.000 | 0.000 | 0.001 | 716 | tags=56%, list=5%, signal=59% |
| 9 | DNA\_METABOLIC\_PROCESS |  | 240 | 0.42 | 2.44 | 0.000 | 0.000 | 0.001 | 2644 | tags=43%, list=20%, signal=53% |
| 10 | MITOTIC\_SISTER\_CHROMATID\_SEGREGATION |  | 15 | 0.77 | 2.44 | 0.000 | 0.000 | 0.001 | 716 | tags=60%, list=5%, signal=63% |
| 11 | CELL\_CYCLE\_GO\_0007049 |  | 277 | 0.41 | 2.42 | 0.000 | 0.000 | 0.001 | 2527 | tags=42%, list=19%, signal=51% |
| 12 | CHROMOSOME\_SEGREGATION |  | 28 | 0.61 | 2.37 | 0.000 | 0.000 | 0.001 | 716 | tags=46%, list=5%, signal=49% |
| 13 | CELL\_CYCLE\_CHECKPOINT\_GO\_0000075 |  | 45 | 0.53 | 2.34 | 0.000 | 0.000 | 0.001 | 2506 | tags=58%, list=19%, signal=71% |
| 14 | DNA\_DEPENDENT\_DNA\_REPLICATION |  | 52 | 0.52 | 2.34 | 0.000 | 0.000 | 0.001 | 2803 | tags=54%, list=21%, signal=68% |
| 15 | REGULATION\_OF\_MITOSIS |  | 33 | 0.58 | 2.30 | 0.000 | 0.000 | 0.002 | 1880 | tags=52%, list=14%, signal=60% |
| 16 | DNA\_REPAIR |  | 118 | 0.44 | 2.29 | 0.000 | 0.000 | 0.002 | 2644 | tags=46%, list=20%, signal=57% |
| 17 | RESPONSE\_TO\_DNA\_DAMAGE\_STIMULUS |  | 153 | 0.41 | 2.26 | 0.000 | 0.000 | 0.004 | 2644 | tags=44%, list=20%, signal=54% |
| 18 | RNA\_SPLICING |  | 74 | 0.45 | 2.20 | 0.000 | 0.000 | 0.010 | 3558 | tags=54%, list=27%, signal=74% |
| 19 | RESPONSE\_TO\_ENDOGENOUS\_STIMULUS |  | 182 | 0.38 | 2.16 | 0.000 | 0.001 | 0.017 | 3279 | tags=46%, list=25%, signal=60% |
| 20 | NUCLEOTIDE\_BIOSYNTHETIC\_PROCESS |  | 17 | 0.65 | 2.15 | 0.000 | 0.001 | 0.022 | 1444 | tags=53%, list=11%, signal=59% |
| 21 | RNA\_PROCESSING |  | 138 | 0.40 | 2.14 | 0.000 | 0.001 | 0.025 | 3424 | tags=51%, list=26%, signal=68% |
| 22 | MICROTUBULE\_CYTOSKELETON\_ORGANIZATION\_AND\_BIOGENESIS |  | 31 | 0.53 | 2.07 | 0.000 | 0.002 | 0.058 | 2721 | tags=55%, list=21%, signal=69% |
| 23 | DNA\_INTEGRITY\_CHECKPOINT |  | 22 | 0.58 | 2.07 | 0.000 | 0.002 | 0.058 | 2131 | tags=59%, list=16%, signal=70% |
| 24 | MITOTIC\_CELL\_CYCLE\_CHECKPOINT |  | 19 | 0.60 | 2.07 | 0.002 | 0.002 | 0.060 | 1880 | tags=53%, list=14%, signal=61% |
| 25 | DNA\_REPLICATION\_INITIATION |  | 15 | 0.65 | 2.04 | 0.000 | 0.003 | 0.095 | 2803 | tags=80%, list=21%, signal=102% |
| 26 | MRNA\_METABOLIC\_PROCESS |  | 72 | 0.42 | 2.02 | 0.000 | 0.003 | 0.111 | 3191 | tags=50%, list=24%, signal=66% |
| 27 | PROTEIN\_FOLDING |  | 55 | 0.44 | 2.01 | 0.000 | 0.004 | 0.125 | 3228 | tags=51%, list=25%, signal=67% |
| 28 | REGULATION\_OF\_CELL\_CYCLE |  | 161 | 0.36 | 1.99 | 0.000 | 0.004 | 0.141 | 2131 | tags=36%, list=16%, signal=43% |
| 29 | REGULATION\_OF\_MITOTIC\_CELL\_CYCLE |  | 19 | 0.58 | 1.99 | 0.002 | 0.004 | 0.149 | 1260 | tags=47%, list=10%, signal=52% |
| 30 | MITOCHONDRION\_ORGANIZATION\_AND\_BIOGENESIS |  | 42 | 0.46 | 1.98 | 0.000 | 0.004 | 0.157 | 3671 | tags=55%, list=28%, signal=76% |
| 31 | DOUBLE\_STRAND\_BREAK\_REPAIR |  | 21 | 0.56 | 1.98 | 0.004 | 0.004 | 0.161 | 1923 | tags=52%, list=15%, signal=61% |
| 32 | COENZYME\_METABOLIC\_PROCESS |  | 35 | 0.48 | 1.94 | 0.002 | 0.006 | 0.231 | 3435 | tags=49%, list=26%, signal=66% |
| 33 | TRNA\_METABOLIC\_PROCESS |  | 15 | 0.61 | 1.94 | 0.004 | 0.006 | 0.232 | 2773 | tags=67%, list=21%, signal=84% |
| 34 | NUCLEOBASENUCLEOSIDENUCLEOTIDE\_AND\_NUCLEIC\_ACID\_TRANSPORT |  | 26 | 0.50 | 1.92 | 0.002 | 0.008 | 0.279 | 2617 | tags=50%, list=20%, signal=62% |
| 35 | MRNA\_PROCESSING\_GO\_0006397 |  | 61 | 0.42 | 1.92 | 0.002 | 0.008 | 0.285 | 3191 | tags=48%, list=24%, signal=63% |
| 36 | INTERPHASE\_OF\_MITOTIC\_CELL\_CYCLE |  | 57 | 0.42 | 1.90 | 0.000 | 0.009 | 0.344 | 3441 | tags=51%, list=26%, signal=69% |
| 37 | G1\_S\_TRANSITION\_OF\_MITOTIC\_CELL\_CYCLE |  | 23 | 0.51 | 1.89 | 0.000 | 0.010 | 0.380 | 2423 | tags=48%, list=19%, signal=59% |
| 38 | INTERPHASE |  | 63 | 0.41 | 1.88 | 0.000 | 0.010 | 0.384 | 3264 | tags=48%, list=25%, signal=63% |
| 39 | COFACTOR\_BIOSYNTHETIC\_PROCESS |  | 21 | 0.53 | 1.88 | 0.002 | 0.010 | 0.389 | 1481 | tags=38%, list=11%, signal=43% |
| 40 | REGULATION\_OF\_CYCLIN\_DEPENDENT\_PROTEIN\_KINASE\_ACTIVITY |  | 40 | 0.45 | 1.88 | 0.002 | 0.009 | 0.390 | 2633 | tags=50%, list=20%, signal=62% |
| 41 | CHROMOSOME\_ORGANIZATION\_AND\_BIOGENESIS |  | 107 | 0.37 | 1.87 | 0.000 | 0.010 | 0.418 | 2569 | tags=37%, list=20%, signal=46% |
| 42 | REGULATION\_OF\_DNA\_METABOLIC\_PROCESS |  | 40 | 0.44 | 1.86 | 0.000 | 0.011 | 0.446 | 2131 | tags=45%, list=16%, signal=54% |
| 43 | REGULATION\_OF\_DNA\_REPLICATION |  | 18 | 0.54 | 1.84 | 0.009 | 0.013 | 0.523 | 2131 | tags=50%, list=16%, signal=60% |
| 44 | MITOCHONDRIAL\_TRANSPORT |  | 18 | 0.54 | 1.81 | 0.004 | 0.016 | 0.596 | 1390 | tags=44%, list=11%, signal=50% |
| 45 | PROTEIN\_MODIFICATION\_BY\_SMALL\_PROTEIN\_CONJUGATION |  | 35 | 0.45 | 1.80 | 0.002 | 0.016 | 0.618 | 2106 | tags=40%, list=16%, signal=48% |
| 46 | UBIQUITIN\_CYCLE |  | 40 | 0.43 | 1.80 | 0.005 | 0.017 | 0.636 | 2106 | tags=38%, list=16%, signal=45% |
| 47 | DNA\_DAMAGE\_CHECKPOINT |  | 19 | 0.53 | 1.79 | 0.004 | 0.018 | 0.659 | 2131 | tags=53%, list=16%, signal=63% |
| 48 | DNA\_DAMAGE\_RESPONSESIGNAL\_TRANSDUCTION |  | 34 | 0.45 | 1.76 | 0.002 | 0.022 | 0.736 | 2337 | tags=47%, list=18%, signal=57% |
| 49 | COFACTOR\_METABOLIC\_PROCESS |  | 51 | 0.39 | 1.75 | 0.004 | 0.022 | 0.751 | 3495 | tags=43%, list=27%, signal=59% |
| 50 | BIOPOLYMER\_CATABOLIC\_PROCESS |  | 103 | 0.34 | 1.74 | 0.000 | 0.024 | 0.781 | 3131 | tags=40%, list=24%, signal=52% |
| 51 | TRANSCRIPTION\_INITIATION\_FROM\_RNA\_POLYMERASE\_II\_PROMOTER |  | 27 | 0.47 | 1.73 | 0.013 | 0.025 | 0.804 | 2633 | tags=44%, list=20%, signal=56% |
| 52 | PROTEIN\_UBIQUITINATION |  | 32 | 0.43 | 1.73 | 0.009 | 0.026 | 0.817 | 2106 | tags=38%, list=16%, signal=45% |
| 53 | DNA\_RECOMBINATION |  | 45 | 0.40 | 1.73 | 0.005 | 0.025 | 0.817 | 1388 | tags=33%, list=11%, signal=37% |
| 54 | PROTEIN\_CATABOLIC\_PROCESS |  | 60 | 0.38 | 1.72 | 0.004 | 0.027 | 0.843 | 2329 | tags=33%, list=18%, signal=40% |
| 55 | CELLULAR\_PROTEIN\_CATABOLIC\_PROCESS |  | 50 | 0.39 | 1.71 | 0.007 | 0.029 | 0.874 | 2329 | tags=34%, list=18%, signal=41% |
| 56 | DNA\_PACKAGING |  | 29 | 0.44 | 1.69 | 0.016 | 0.033 | 0.919 | 2569 | tags=45%, list=20%, signal=56% |
| 57 | NUCLEAR\_EXPORT |  | 26 | 0.45 | 1.67 | 0.011 | 0.037 | 0.940 | 2617 | tags=42%, list=20%, signal=53% |
| 58 | MACROMOLECULE\_CATABOLIC\_PROCESS |  | 120 | 0.31 | 1.66 | 0.003 | 0.038 | 0.946 | 2617 | tags=33%, list=20%, signal=40% |
| 59 | MEIOSIS\_I |  | 19 | 0.48 | 1.66 | 0.014 | 0.040 | 0.955 | 1388 | tags=37%, list=11%, signal=41% |
| 60 | BASE\_EXCISION\_REPAIR |  | 16 | 0.49 | 1.64 | 0.015 | 0.045 | 0.970 | 2601 | tags=44%, list=20%, signal=55% |
| 61 | PROTEIN\_DNA\_COMPLEX\_ASSEMBLY |  | 45 | 0.38 | 1.64 | 0.010 | 0.045 | 0.971 | 2633 | tags=40%, list=20%, signal=50% |
| 62 | CYTOKINESIS |  | 17 | 0.49 | 1.62 | 0.027 | 0.050 | 0.984 | 1063 | tags=35%, list=8%, signal=38% |
| 63 | RNA\_EXPORT\_FROM\_NUCLEUS |  | 17 | 0.49 | 1.62 | 0.027 | 0.050 | 0.986 | 4516 | tags=71%, list=34%, signal=108% |
| 64 | ONE\_CARBON\_COMPOUND\_METABOLIC\_PROCESS |  | 24 | 0.45 | 1.62 | 0.022 | 0.049 | 0.986 | 2816 | tags=50%, list=22%, signal=64% |
| 65 | MEIOTIC\_CELL\_CYCLE |  | 31 | 0.41 | 1.59 | 0.015 | 0.059 | 0.993 | 2721 | tags=42%, list=21%, signal=53% |
| 66 | APOPTOTIC\_NUCLEAR\_CHANGES |  | 17 | 0.47 | 1.55 | 0.044 | 0.077 | 0.999 | 2423 | tags=47%, list=19%, signal=58% |
| 67 | CHROMATIN\_ASSEMBLY\_OR\_DISASSEMBLY |  | 25 | 0.42 | 1.55 | 0.027 | 0.078 | 0.999 | 2569 | tags=48%, list=20%, signal=60% |
| 68 | NUCLEAR\_TRANSPORT |  | 77 | 0.32 | 1.54 | 0.009 | 0.079 | 0.999 | 3279 | tags=38%, list=25%, signal=50% |
| 69 | CELLULAR\_MACROMOLECULE\_CATABOLIC\_PROCESS |  | 90 | 0.31 | 1.53 | 0.015 | 0.085 | 1.000 | 2617 | tags=31%, list=20%, signal=39% |
| 70 | NUCLEOBASENUCLEOSIDE\_AND\_NUCLEOTIDE\_METABOLIC\_PROCESS |  | 46 | 0.36 | 1.53 | 0.008 | 0.084 | 1.000 | 896 | tags=26%, list=7%, signal=28% |
| 71 | NUCLEOTIDE\_METABOLIC\_PROCESS |  | 36 | 0.38 | 1.53 | 0.036 | 0.084 | 1.000 | 896 | tags=28%, list=7%, signal=30% |
| 72 | ORGANELLE\_ORGANIZATION\_AND\_BIOGENESIS |  | 407 | 0.24 | 1.53 | 0.000 | 0.084 | 1.000 | 3236 | tags=33%, list=25%, signal=43% |
| 73 | NUCLEOCYTOPLASMIC\_TRANSPORT |  | 77 | 0.32 | 1.52 | 0.017 | 0.086 | 1.000 | 3279 | tags=38%, list=25%, signal=50% |
| 74 | CELLULAR\_COMPONENT\_DISASSEMBLY |  | 31 | 0.39 | 1.51 | 0.038 | 0.089 | 1.000 | 2423 | tags=39%, list=19%, signal=47% |
| 75 | TRANSCRIPTION\_INITIATION |  | 33 | 0.38 | 1.50 | 0.036 | 0.093 | 1.000 | 2633 | tags=39%, list=20%, signal=49% |
| 76 | CELL\_DIVISION |  | 19 | 0.44 | 1.49 | 0.057 | 0.100 | 1.000 | 1063 | tags=32%, list=8%, signal=34% |
| 77 | MICROTUBULE\_BASED\_PROCESS |  | 75 | 0.31 | 1.48 | 0.018 | 0.108 | 1.000 | 2854 | tags=36%, list=22%, signal=46% |
| 78 | ESTABLISHMENT\_OF\_ORGANELLE\_LOCALIZATION |  | 16 | 0.46 | 1.46 | 0.064 | 0.120 | 1.000 | 931 | tags=38%, list=7%, signal=40% |
| 79 | VIRAL\_INFECTIOUS\_CYCLE |  | 29 | 0.38 | 1.45 | 0.055 | 0.128 | 1.000 | 1045 | tags=31%, list=8%, signal=34% |
| 80 | ORGANELLE\_LOCALIZATION |  | 21 | 0.40 | 1.43 | 0.081 | 0.143 | 1.000 | 931 | tags=29%, list=7%, signal=31% |
| 81 | NEGATIVE\_REGULATION\_OF\_DNA\_METABOLIC\_PROCESS |  | 16 | 0.43 | 1.42 | 0.092 | 0.150 | 1.000 | 2562 | tags=50%, list=20%, signal=62% |
| 82 | NEGATIVE\_REGULATION\_OF\_BINDING |  | 16 | 0.43 | 1.40 | 0.097 | 0.169 | 1.000 | 2532 | tags=50%, list=19%, signal=62% |
| 83 | APOPTOTIC\_PROGRAM |  | 56 | 0.31 | 1.38 | 0.056 | 0.190 | 1.000 | 3714 | tags=46%, list=28%, signal=65% |
| 84 | ALCOHOL\_METABOLIC\_PROCESS |  | 82 | 0.28 | 1.37 | 0.061 | 0.193 | 1.000 | 3920 | tags=39%, list=30%, signal=55% |
| 85 | CHROMATIN\_REMODELING |  | 21 | 0.39 | 1.37 | 0.100 | 0.198 | 1.000 | 2569 | tags=43%, list=20%, signal=53% |
| 86 | VIRAL\_REPRODUCTIVE\_PROCESS |  | 33 | 0.35 | 1.36 | 0.106 | 0.199 | 1.000 | 1237 | tags=30%, list=9%, signal=33% |
| 87 | ESTABLISHMENT\_AND\_OR\_MAINTENANCE\_OF\_CHROMATIN\_ARCHITECTURE |  | 65 | 0.29 | 1.36 | 0.067 | 0.201 | 1.000 | 2569 | tags=34%, list=20%, signal=42% |
| 88 | REGULATION\_OF\_GENE\_EXPRESSION\_EPIGENETIC |  | 27 | 0.35 | 1.35 | 0.094 | 0.214 | 1.000 | 2732 | tags=41%, list=21%, signal=51% |
| 89 | INTRACELLULAR\_TRANSPORT |  | 248 | 0.23 | 1.33 | 0.024 | 0.235 | 1.000 | 3653 | tags=35%, list=28%, signal=48% |
| 90 | G1\_PHASE |  | 15 | 0.41 | 1.33 | 0.142 | 0.234 | 1.000 | 465 | tags=27%, list=4%, signal=28% |
| 91 | RESPONSE\_TO\_ORGANIC\_SUBSTANCE |  | 27 | 0.35 | 1.33 | 0.119 | 0.233 | 1.000 | 2826 | tags=37%, list=22%, signal=47% |
| 92 | RESPONSE\_TO\_HYPOXIA |  | 27 | 0.35 | 1.32 | 0.128 | 0.233 | 1.000 | 2325 | tags=33%, list=18%, signal=40% |
| 93 | MEIOTIC\_RECOMBINATION |  | 16 | 0.41 | 1.32 | 0.132 | 0.234 | 1.000 | 1388 | tags=31%, list=11%, signal=35% |
| 94 | RESPONSE\_TO\_ABIOTIC\_STIMULUS |  | 79 | 0.26 | 1.28 | 0.092 | 0.289 | 1.000 | 3435 | tags=37%, list=26%, signal=49% |
| 95 | VIRAL\_GENOME\_REPLICATION |  | 20 | 0.37 | 1.28 | 0.118 | 0.292 | 1.000 | 1741 | tags=35%, list=13%, signal=40% |
| 96 | ESTABLISHMENT\_OF\_CELLULAR\_LOCALIZATION |  | 311 | 0.21 | 1.28 | 0.044 | 0.290 | 1.000 | 3279 | tags=31%, list=25%, signal=40% |
| 97 | CYTOSKELETON\_DEPENDENT\_INTRACELLULAR\_TRANSPORT |  | 25 | 0.35 | 1.28 | 0.144 | 0.291 | 1.000 | 3943 | tags=52%, list=30%, signal=74% |
| 98 | VIRAL\_REPRODUCTION |  | 38 | 0.31 | 1.27 | 0.130 | 0.291 | 1.000 | 1237 | tags=26%, list=9%, signal=29% |
| 99 | RESPONSE\_TO\_STRESS |  | 467 | 0.20 | 1.27 | 0.024 | 0.289 | 1.000 | 3018 | tags=29%, list=23%, signal=37% |
| 100 | NEGATIVE\_REGULATION\_OF\_CATALYTIC\_ACTIVITY |  | 61 | 0.28 | 1.27 | 0.119 | 0.292 | 1.000 | 2676 | tags=34%, list=20%, signal=43% |
| 101 | HETEROCYCLE\_METABOLIC\_PROCESS |  | 26 | 0.34 | 1.26 | 0.161 | 0.306 | 1.000 | 1481 | tags=23%, list=11%, signal=26% |
| 102 | NUCLEAR\_ORGANIZATION\_AND\_BIOGENESIS |  | 23 | 0.35 | 1.26 | 0.170 | 0.303 | 1.000 | 2423 | tags=39%, list=19%, signal=48% |
| 103 | CELLULAR\_LOCALIZATION |  | 323 | 0.20 | 1.25 | 0.057 | 0.314 | 1.000 | 3279 | tags=30%, list=25%, signal=39% |
| 104 | NEGATIVE\_REGULATION\_OF\_DNA\_BINDING |  | 15 | 0.39 | 1.24 | 0.203 | 0.334 | 1.000 | 2532 | tags=47%, list=19%, signal=58% |
| 105 | DNA\_CATABOLIC\_PROCESS |  | 21 | 0.36 | 1.23 | 0.188 | 0.339 | 1.000 | 2506 | tags=38%, list=19%, signal=47% |
| 106 | RNA\_CATABOLIC\_PROCESS |  | 20 | 0.36 | 1.23 | 0.207 | 0.343 | 1.000 | 3125 | tags=50%, list=24%, signal=66% |
| 107 | CHROMATIN\_ASSEMBLY |  | 16 | 0.38 | 1.23 | 0.218 | 0.344 | 1.000 | 2569 | tags=44%, list=20%, signal=54% |
| 108 | RIBONUCLEOPROTEIN\_COMPLEX\_BIOGENESIS\_AND\_ASSEMBLY |  | 68 | 0.26 | 1.22 | 0.148 | 0.345 | 1.000 | 2787 | tags=32%, list=21%, signal=41% |
| 109 | CELL\_STRUCTURE\_DISASSEMBLY\_DURING\_APOPTOSIS |  | 17 | 0.36 | 1.21 | 0.228 | 0.372 | 1.000 | 2423 | tags=35%, list=19%, signal=43% |
| 110 | OXYGEN\_AND\_REACTIVE\_OXYGEN\_SPECIES\_METABOLIC\_PROCESS |  | 18 | 0.35 | 1.18 | 0.227 | 0.423 | 1.000 | 2993 | tags=44%, list=23%, signal=58% |
| 111 | CELLULAR\_RESPIRATION |  | 19 | 0.34 | 1.17 | 0.244 | 0.444 | 1.000 | 2660 | tags=37%, list=20%, signal=46% |
| 112 | TRANSCRIPTION\_FROM\_RNA\_POLYMERASE\_II\_PROMOTER |  | 428 | 0.18 | 1.16 | 0.096 | 0.455 | 1.000 | 2822 | tags=26%, list=22%, signal=32% |
| 113 | TRANSCRIPTION\_FROM\_RNA\_POLYMERASE\_III\_PROMOTER |  | 18 | 0.35 | 1.16 | 0.280 | 0.453 | 1.000 | 3817 | tags=56%, list=29%, signal=78% |
| 114 | REGULATION\_OF\_HYDROLASE\_ACTIVITY |  | 65 | 0.25 | 1.16 | 0.224 | 0.454 | 1.000 | 3269 | tags=35%, list=25%, signal=47% |
| 115 | CELLULAR\_CATABOLIC\_PROCESS |  | 189 | 0.20 | 1.15 | 0.152 | 0.463 | 1.000 | 2660 | tags=25%, list=20%, signal=31% |
| 116 | CELLULAR\_RESPONSE\_TO\_STIMULUS |  | 17 | 0.34 | 1.15 | 0.268 | 0.460 | 1.000 | 4193 | tags=53%, list=32%, signal=78% |
| 117 | INDUCTION\_OF\_APOPTOSIS\_BY\_EXTRACELLULAR\_SIGNALS |  | 25 | 0.31 | 1.15 | 0.282 | 0.470 | 1.000 | 2631 | tags=36%, list=20%, signal=45% |
| 118 | CHROMATIN\_MODIFICATION |  | 46 | 0.26 | 1.15 | 0.241 | 0.469 | 1.000 | 2816 | tags=33%, list=22%, signal=41% |
| 119 | CATABOLIC\_PROCESS |  | 201 | 0.20 | 1.14 | 0.172 | 0.469 | 1.000 | 2660 | tags=25%, list=20%, signal=31% |
| 120 | REGULATION\_OF\_KINASE\_ACTIVITY |  | 135 | 0.21 | 1.14 | 0.210 | 0.465 | 1.000 | 2102 | tags=23%, list=16%, signal=27% |
| 121 | NITROGEN\_COMPOUND\_BIOSYNTHETIC\_PROCESS |  | 25 | 0.30 | 1.14 | 0.276 | 0.463 | 1.000 | 1611 | tags=24%, list=12%, signal=27% |
| 122 | REGULATION\_OF\_CATALYTIC\_ACTIVITY |  | 238 | 0.19 | 1.14 | 0.166 | 0.469 | 1.000 | 3269 | tags=30%, list=25%, signal=39% |
| 123 | REGULATION\_OF\_TRANSFERASE\_ACTIVITY |  | 137 | 0.21 | 1.13 | 0.223 | 0.492 | 1.000 | 1333 | tags=18%, list=10%, signal=20% |
| 124 | GLUTAMATE\_SIGNALING\_PATHWAY |  | 17 | 0.34 | 1.12 | 0.292 | 0.502 | 1.000 | 3068 | tags=29%, list=23%, signal=38% |
| 125 | NUCLEAR\_IMPORT |  | 47 | 0.26 | 1.12 | 0.265 | 0.499 | 1.000 | 3279 | tags=34%, list=25%, signal=45% |
| 126 | REGULATION\_OF\_PROTEIN\_KINASE\_ACTIVITY |  | 133 | 0.21 | 1.11 | 0.252 | 0.519 | 1.000 | 4363 | tags=43%, list=33%, signal=64% |
| 127 | NEGATIVE\_REGULATION\_OF\_TRANSPORT |  | 18 | 0.33 | 1.11 | 0.311 | 0.516 | 1.000 | 3501 | tags=44%, list=27%, signal=61% |
| 128 | NEURON\_APOPTOSIS |  | 15 | 0.34 | 1.11 | 0.304 | 0.513 | 1.000 | 1236 | tags=27%, list=9%, signal=29% |
| 129 | INTERACTION\_WITH\_HOST |  | 15 | 0.34 | 1.09 | 0.332 | 0.547 | 1.000 | 1237 | tags=27%, list=9%, signal=29% |
| 130 | RNA\_SPLICINGVIA\_TRANSESTERIFICATION\_REACTIONS |  | 27 | 0.29 | 1.08 | 0.336 | 0.589 | 1.000 | 3884 | tags=41%, list=30%, signal=58% |
| 131 | RESPONSE\_TO\_HORMONE\_STIMULUS |  | 26 | 0.28 | 1.08 | 0.354 | 0.588 | 1.000 | 3533 | tags=38%, list=27%, signal=53% |
| 132 | LIPID\_BIOSYNTHETIC\_PROCESS |  | 84 | 0.22 | 1.07 | 0.351 | 0.610 | 1.000 | 1837 | tags=21%, list=14%, signal=25% |
| 133 | REGULATION\_OF\_MOLECULAR\_FUNCTION |  | 275 | 0.18 | 1.06 | 0.302 | 0.623 | 1.000 | 3269 | tags=29%, list=25%, signal=37% |
| 134 | CELLULAR\_BIOSYNTHETIC\_PROCESS |  | 273 | 0.18 | 1.06 | 0.321 | 0.621 | 1.000 | 2850 | tags=26%, list=22%, signal=32% |
| 135 | DNA\_DAMAGE\_RESPONSESIGNAL\_TRANSDUCTION\_RESULTING\_IN\_INDUCTION\_OF\_APOPTOSIS |  | 15 | 0.33 | 1.06 | 0.370 | 0.617 | 1.000 | 1101 | tags=27%, list=8%, signal=29% |
| 136 | INTRACELLULAR\_PROTEIN\_TRANSPORT |  | 127 | 0.20 | 1.05 | 0.358 | 0.630 | 1.000 | 3279 | tags=29%, list=25%, signal=38% |
| 137 | REGULATION\_OF\_PROTEIN\_STABILITY |  | 17 | 0.32 | 1.05 | 0.385 | 0.626 | 1.000 | 4112 | tags=41%, list=31%, signal=60% |
| 138 | PIGMENT\_BIOSYNTHETIC\_PROCESS |  | 17 | 0.31 | 1.04 | 0.402 | 0.638 | 1.000 | 1481 | tags=24%, list=11%, signal=26% |
| 139 | CYTOSKELETON\_ORGANIZATION\_AND\_BIOGENESIS |  | 182 | 0.18 | 1.03 | 0.365 | 0.660 | 1.000 | 2878 | tags=27%, list=22%, signal=34% |
| 140 | GAMETE\_GENERATION |  | 92 | 0.21 | 1.03 | 0.376 | 0.660 | 1.000 | 3858 | tags=34%, list=29%, signal=47% |
| 141 | MACROMOLECULE\_LOCALIZATION |  | 202 | 0.18 | 1.03 | 0.387 | 0.660 | 1.000 | 3279 | tags=28%, list=25%, signal=37% |
| 142 | NEGATIVE\_REGULATION\_OF\_APOPTOSIS |  | 136 | 0.19 | 1.02 | 0.412 | 0.673 | 1.000 | 1556 | tags=19%, list=12%, signal=21% |
| 143 | PROTEIN\_IMPORT |  | 58 | 0.22 | 1.02 | 0.429 | 0.683 | 1.000 | 3279 | tags=29%, list=25%, signal=39% |
| 144 | PROTEIN\_TARGETING |  | 94 | 0.20 | 1.02 | 0.408 | 0.681 | 1.000 | 3279 | tags=29%, list=25%, signal=38% |
| 145 | PROTEIN\_TRANSPORT |  | 139 | 0.19 | 1.02 | 0.452 | 0.685 | 1.000 | 3279 | tags=29%, list=25%, signal=38% |
| 146 | NEGATIVE\_REGULATION\_OF\_TRANSFERASE\_ACTIVITY |  | 27 | 0.27 | 1.01 | 0.458 | 0.683 | 1.000 | 2621 | tags=33%, list=20%, signal=42% |
| 147 | RESPONSE\_TO\_TEMPERATURE\_STIMULUS |  | 16 | 0.32 | 1.01 | 0.448 | 0.682 | 1.000 | 3435 | tags=44%, list=26%, signal=59% |
| 148 | REGULATION\_OF\_NEUROTRANSMITTER\_LEVELS |  | 23 | 0.28 | 1.01 | 0.427 | 0.685 | 1.000 | 922 | tags=17%, list=7%, signal=19% |
| 149 | STEROID\_BIOSYNTHETIC\_PROCESS |  | 22 | 0.28 | 1.01 | 0.445 | 0.688 | 1.000 | 4120 | tags=55%, list=31%, signal=79% |
| 150 | NEGATIVE\_REGULATION\_OF\_PROGRAMMED\_CELL\_DEATH |  | 137 | 0.19 | 1.01 | 0.449 | 0.687 | 1.000 | 1556 | tags=19%, list=12%, signal=21% |
| 151 | SECONDARY\_METABOLIC\_PROCESS |  | 23 | 0.27 | 1.00 | 0.460 | 0.687 | 1.000 | 1481 | tags=22%, list=11%, signal=24% |
| 152 | DIGESTION |  | 42 | 0.24 | 1.00 | 0.422 | 0.690 | 1.000 | 2938 | tags=24%, list=22%, signal=31% |
| 153 | PROGRAMMED\_CELL\_DEATH |  | 393 | 0.16 | 0.99 | 0.476 | 0.713 | 1.000 | 1658 | tags=17%, list=13%, signal=19% |
| 154 | REGULATION\_OF\_PROGRAMMED\_CELL\_DEATH |  | 313 | 0.16 | 0.99 | 0.496 | 0.713 | 1.000 | 1640 | tags=17%, list=13%, signal=19% |
| 155 | APOPTOSIS\_GO |  | 392 | 0.16 | 0.99 | 0.495 | 0.714 | 1.000 | 1658 | tags=17%, list=13%, signal=19% |
| 156 | PIGMENT\_METABOLIC\_PROCESS |  | 18 | 0.29 | 0.99 | 0.470 | 0.710 | 1.000 | 1481 | tags=22%, list=11%, signal=25% |
| 157 | CARBOHYDRATE\_TRANSPORT |  | 17 | 0.30 | 0.98 | 0.484 | 0.732 | 1.000 | 2501 | tags=29%, list=19%, signal=36% |
| 158 | REGULATION\_OF\_APOPTOSIS |  | 312 | 0.16 | 0.97 | 0.528 | 0.734 | 1.000 | 1640 | tags=17%, list=13%, signal=19% |
| 159 | MICROTUBULE\_BASED\_MOVEMENT |  | 16 | 0.30 | 0.97 | 0.518 | 0.746 | 1.000 | 2854 | tags=38%, list=22%, signal=48% |
| 160 | ENERGY\_DERIVATION\_BY\_OXIDATION\_OF\_ORGANIC\_COMPOUNDS |  | 37 | 0.24 | 0.97 | 0.494 | 0.744 | 1.000 | 1257 | tags=19%, list=10%, signal=21% |
| 161 | MORPHOGENESIS\_OF\_AN\_EPITHELIUM |  | 15 | 0.30 | 0.97 | 0.485 | 0.742 | 1.000 | 3828 | tags=47%, list=29%, signal=66% |
| 162 | CELL\_PROJECTION\_BIOGENESIS |  | 20 | 0.28 | 0.96 | 0.493 | 0.759 | 1.000 | 4197 | tags=45%, list=32%, signal=66% |
| 163 | PROTEIN\_AMINO\_ACID\_O\_LINKED\_GLYCOSYLATION |  | 18 | 0.28 | 0.96 | 0.515 | 0.757 | 1.000 | 2763 | tags=39%, list=21%, signal=49% |
| 164 | STEROID\_METABOLIC\_PROCESS |  | 66 | 0.21 | 0.95 | 0.542 | 0.759 | 1.000 | 4219 | tags=39%, list=32%, signal=58% |
| 165 | REGULATION\_OF\_TRANSCRIPTION\_FROM\_RNA\_POLYMERASE\_II\_PROMOTER |  | 267 | 0.16 | 0.95 | 0.571 | 0.761 | 1.000 | 2633 | tags=23%, list=20%, signal=28% |
| 166 | STEROID\_HORMONE\_RECEPTOR\_SIGNALING\_PATHWAY |  | 18 | 0.28 | 0.95 | 0.539 | 0.759 | 1.000 | 1374 | tags=22%, list=10%, signal=25% |
| 167 | INTRACELLULAR\_RECEPTOR\_MEDIATED\_SIGNALING\_PATHWAY |  | 18 | 0.28 | 0.94 | 0.552 | 0.777 | 1.000 | 1374 | tags=22%, list=10%, signal=25% |
| 168 | AROMATIC\_COMPOUND\_METABOLIC\_PROCESS |  | 26 | 0.24 | 0.94 | 0.537 | 0.773 | 1.000 | 286 | tags=15%, list=2%, signal=16% |
| 169 | PROTEIN\_IMPORT\_INTO\_NUCLEUS |  | 45 | 0.22 | 0.94 | 0.550 | 0.776 | 1.000 | 3279 | tags=31%, list=25%, signal=41% |
| 170 | COVALENT\_CHROMATIN\_MODIFICATION |  | 22 | 0.27 | 0.94 | 0.544 | 0.776 | 1.000 | 4070 | tags=45%, list=31%, signal=66% |
| 171 | GENERATION\_OF\_A\_SIGNAL\_INVOLVED\_IN\_CELL\_CELL\_SIGNALING |  | 25 | 0.26 | 0.93 | 0.573 | 0.793 | 1.000 | 2444 | tags=28%, list=19%, signal=34% |
| 172 | PROTEIN\_RNA\_COMPLEX\_ASSEMBLY |  | 55 | 0.21 | 0.92 | 0.581 | 0.805 | 1.000 | 3884 | tags=38%, list=30%, signal=54% |
| 173 | BIOSYNTHETIC\_PROCESS |  | 402 | 0.15 | 0.91 | 0.754 | 0.834 | 1.000 | 2850 | tags=23%, list=22%, signal=29% |
| 174 | REGULATION\_OF\_TRANSPORT |  | 57 | 0.20 | 0.91 | 0.597 | 0.834 | 1.000 | 4249 | tags=42%, list=32%, signal=62% |
| 175 | REGULATION\_OF\_RNA\_METABOLIC\_PROCESS |  | 417 | 0.14 | 0.89 | 0.812 | 0.879 | 1.000 | 3828 | tags=31%, list=29%, signal=43% |
| 176 | INDUCTION\_OF\_APOPTOSIS\_BY\_INTRACELLULAR\_SIGNALS |  | 23 | 0.24 | 0.89 | 0.622 | 0.876 | 1.000 | 1558 | tags=22%, list=12%, signal=25% |
| 177 | SPLICEOSOME\_ASSEMBLY |  | 17 | 0.27 | 0.88 | 0.612 | 0.877 | 1.000 | 3884 | tags=41%, list=30%, signal=58% |
| 178 | TRANSMISSION\_OF\_NERVE\_IMPULSE |  | 167 | 0.16 | 0.88 | 0.718 | 0.879 | 1.000 | 2677 | tags=21%, list=20%, signal=26% |
| 179 | ENERGY\_RESERVE\_METABOLIC\_PROCESS |  | 15 | 0.28 | 0.88 | 0.612 | 0.882 | 1.000 | 1257 | tags=20%, list=10%, signal=22% |
| 180 | SEXUAL\_REPRODUCTION |  | 109 | 0.17 | 0.88 | 0.697 | 0.878 | 1.000 | 3858 | tags=30%, list=29%, signal=43% |
| 181 | POSITIVE\_REGULATION\_OF\_CELL\_CYCLE |  | 15 | 0.28 | 0.88 | 0.641 | 0.877 | 1.000 | 467 | tags=20%, list=4%, signal=21% |
| 182 | NEGATIVE\_REGULATION\_OF\_CELL\_ADHESION |  | 16 | 0.27 | 0.87 | 0.628 | 0.877 | 1.000 | 3436 | tags=44%, list=26%, signal=59% |
| 183 | RESPONSE\_TO\_RADIATION |  | 52 | 0.20 | 0.87 | 0.677 | 0.879 | 1.000 | 3279 | tags=31%, list=25%, signal=41% |
| 184 | SYNAPTIC\_TRANSMISSION |  | 154 | 0.15 | 0.86 | 0.779 | 0.911 | 1.000 | 2671 | tags=20%, list=20%, signal=25% |
| 185 | REGULATION\_OF\_INTRACELLULAR\_TRANSPORT |  | 22 | 0.24 | 0.86 | 0.663 | 0.907 | 1.000 | 4580 | tags=50%, list=35%, signal=77% |
| 186 | REGULATION\_OF\_PHOSPHORYLATION |  | 42 | 0.20 | 0.85 | 0.708 | 0.904 | 1.000 | 4225 | tags=45%, list=32%, signal=67% |
| 187 | REGULATION\_OF\_TRANSCRIPTIONDNA\_DEPENDENT |  | 412 | 0.14 | 0.85 | 0.888 | 0.900 | 1.000 | 3828 | tags=31%, list=29%, signal=42% |
| 188 | CASPASE\_ACTIVATION |  | 24 | 0.23 | 0.85 | 0.668 | 0.913 | 1.000 | 3269 | tags=38%, list=25%, signal=50% |
| 189 | AEROBIC\_RESPIRATION |  | 15 | 0.27 | 0.85 | 0.675 | 0.908 | 1.000 | 2660 | tags=33%, list=20%, signal=42% |
| 190 | REGULATION\_OF\_NUCLEOCYTOPLASMIC\_TRANSPORT |  | 19 | 0.24 | 0.84 | 0.694 | 0.915 | 1.000 | 1333 | tags=21%, list=10%, signal=23% |
| 191 | ESTABLISHMENT\_OF\_PROTEIN\_LOCALIZATION |  | 166 | 0.15 | 0.83 | 0.855 | 0.935 | 1.000 | 3279 | tags=26%, list=25%, signal=34% |
| 192 | EPIDERMAL\_GROWTH\_FACTOR\_RECEPTOR\_SIGNALING\_PATHWAY |  | 18 | 0.24 | 0.82 | 0.705 | 0.945 | 1.000 | 4351 | tags=44%, list=33%, signal=66% |
| 193 | DEVELOPMENT\_OF\_PRIMARY\_SEXUAL\_CHARACTERISTICS |  | 25 | 0.22 | 0.82 | 0.742 | 0.944 | 1.000 | 3043 | tags=28%, list=23%, signal=36% |
| 194 | PHOSPHOINOSITIDE\_BIOSYNTHETIC\_PROCESS |  | 21 | 0.23 | 0.81 | 0.726 | 0.955 | 1.000 | 1137 | tags=19%, list=9%, signal=21% |
| 195 | PROTEOLYSIS |  | 170 | 0.15 | 0.81 | 0.888 | 0.956 | 1.000 | 3697 | tags=30%, list=28%, signal=41% |
| 196 | MEMBRANE\_FUSION |  | 27 | 0.21 | 0.80 | 0.758 | 0.962 | 1.000 | 3604 | tags=37%, list=28%, signal=51% |
| 197 | HISTONE\_MODIFICATION |  | 21 | 0.23 | 0.80 | 0.727 | 0.958 | 1.000 | 4070 | tags=43%, list=31%, signal=62% |
| 198 | CALCIUM\_INDEPENDENT\_CELL\_CELL\_ADHESION |  | 16 | 0.25 | 0.80 | 0.709 | 0.953 | 1.000 | 3853 | tags=38%, list=29%, signal=53% |
| 199 | CELL\_CYCLE\_ARREST\_GO\_0007050 |  | 52 | 0.18 | 0.79 | 0.827 | 0.965 | 1.000 | 3813 | tags=38%, list=29%, signal=54% |
| 200 | PROTEIN\_LOCALIZATION |  | 184 | 0.14 | 0.79 | 0.928 | 0.964 | 1.000 | 3279 | tags=26%, list=25%, signal=34% |
| 201 | NEGATIVE\_REGULATION\_OF\_CELL\_CYCLE |  | 72 | 0.16 | 0.79 | 0.849 | 0.962 | 1.000 | 2423 | tags=22%, list=19%, signal=27% |
| 202 | MEMBRANE\_LIPID\_BIOSYNTHETIC\_PROCESS |  | 41 | 0.18 | 0.78 | 0.817 | 0.963 | 1.000 | 1611 | tags=17%, list=12%, signal=19% |
| 203 | EMBRYONIC\_DEVELOPMENT |  | 46 | 0.18 | 0.78 | 0.833 | 0.969 | 1.000 | 3043 | tags=26%, list=23%, signal=34% |
| 204 | RESPONSE\_TO\_UV |  | 22 | 0.22 | 0.78 | 0.780 | 0.966 | 1.000 | 3279 | tags=36%, list=25%, signal=48% |
| 205 | EXOCYTOSIS |  | 22 | 0.21 | 0.77 | 0.794 | 0.970 | 1.000 | 10283 | tags=100%, list=79%, signal=465% |
| 206 | CELLULAR\_CARBOHYDRATE\_METABOLIC\_PROCESS |  | 106 | 0.15 | 0.76 | 0.916 | 0.978 | 1.000 | 3466 | tags=25%, list=26%, signal=34% |
| 207 | GLUCOSE\_METABOLIC\_PROCESS |  | 27 | 0.20 | 0.76 | 0.822 | 0.974 | 1.000 | 4736 | tags=44%, list=36%, signal=69% |
| 208 | LIPID\_TRANSPORT |  | 27 | 0.20 | 0.75 | 0.844 | 0.980 | 1.000 | 1998 | tags=22%, list=15%, signal=26% |
| 209 | PHOSPHOLIPID\_BIOSYNTHETIC\_PROCESS |  | 35 | 0.18 | 0.74 | 0.880 | 0.984 | 1.000 | 1611 | tags=17%, list=12%, signal=19% |
| 210 | REPRODUCTION |  | 215 | 0.13 | 0.74 | 0.980 | 0.984 | 1.000 | 3508 | tags=26%, list=27%, signal=34% |
| 211 | POSITIVE\_REGULATION\_OF\_HYDROLASE\_ACTIVITY |  | 45 | 0.17 | 0.73 | 0.908 | 0.988 | 1.000 | 3269 | tags=29%, list=25%, signal=38% |
| 212 | REGULATION\_OF\_CATABOLIC\_PROCESS |  | 15 | 0.23 | 0.73 | 0.813 | 0.985 | 1.000 | 4162 | tags=47%, list=32%, signal=68% |
| 213 | STRESS\_ACTIVATED\_PROTEIN\_KINASE\_SIGNALING\_PATHWAY |  | 45 | 0.17 | 0.73 | 0.914 | 0.983 | 1.000 | 4125 | tags=38%, list=32%, signal=55% |
| 214 | JNK\_CASCADE |  | 44 | 0.17 | 0.73 | 0.887 | 0.980 | 1.000 | 4125 | tags=39%, list=32%, signal=56% |
| 215 | GLYCEROPHOSPHOLIPID\_BIOSYNTHETIC\_PROCESS |  | 27 | 0.19 | 0.73 | 0.871 | 0.977 | 1.000 | 1137 | tags=15%, list=9%, signal=16% |
| 216 | SENSORY\_PERCEPTION |  | 163 | 0.13 | 0.72 | 0.976 | 0.975 | 1.000 | 5182 | tags=42%, list=40%, signal=68% |
| 217 | NEUROLOGICAL\_SYSTEM\_PROCESS |  | 328 | 0.11 | 0.70 | 1.000 | 0.994 | 1.000 | 2735 | tags=17%, list=21%, signal=21% |
| 218 | LIPOPROTEIN\_METABOLIC\_PROCESS |  | 30 | 0.18 | 0.70 | 0.900 | 0.990 | 1.000 | 1998 | tags=20%, list=15%, signal=24% |
| 219 | HOMEOSTASIS\_OF\_NUMBER\_OF\_CELLS |  | 20 | 0.20 | 0.69 | 0.891 | 0.991 | 1.000 | 2527 | tags=25%, list=19%, signal=31% |
| 220 | NEGATIVE\_REGULATION\_OF\_CELLULAR\_BIOSYNTHETIC\_PROCESS |  | 25 | 0.18 | 0.69 | 0.927 | 0.987 | 1.000 | 3287 | tags=28%, list=25%, signal=37% |
| 221 | REGULATION\_OF\_CELL\_ADHESION |  | 31 | 0.18 | 0.68 | 0.922 | 0.988 | 1.000 | 4152 | tags=42%, list=32%, signal=61% |
| 222 | BIOGENIC\_AMINE\_METABOLIC\_PROCESS |  | 16 | 0.21 | 0.67 | 0.901 | 0.993 | 1.000 | 1611 | tags=19%, list=12%, signal=21% |
| 223 | BRAIN\_DEVELOPMENT |  | 39 | 0.16 | 0.67 | 0.953 | 0.989 | 1.000 | 3157 | tags=28%, list=24%, signal=37% |
| 224 | ANION\_TRANSPORT |  | 27 | 0.18 | 0.66 | 0.934 | 0.990 | 1.000 | 1545 | tags=15%, list=12%, signal=17% |
| 225 | NEGATIVE\_REGULATION\_OF\_BIOSYNTHETIC\_PROCESS |  | 26 | 0.17 | 0.65 | 0.925 | 0.992 | 1.000 | 3287 | tags=27%, list=25%, signal=36% |
| 226 | ADENYLATE\_CYCLASE\_ACTIVATION |  | 18 | 0.18 | 0.61 | 0.956 | 1.000 | 1.000 | 4518 | tags=39%, list=35%, signal=59% |
| 227 | FEEDING\_BEHAVIOR |  | 20 | 0.17 | 0.58 | 0.976 | 1.000 | 1.000 | 5086 | tags=45%, list=39%, signal=73% |
| 228 | LIPOPROTEIN\_BIOSYNTHETIC\_PROCESS |  | 23 | 0.16 | 0.58 | 0.968 | 1.000 | 1.000 | 4009 | tags=35%, list=31%, signal=50% |
| 229 | TUBE\_DEVELOPMENT |  | 15 | 0.18 | 0.57 | 0.965 | 1.000 | 1.000 | 3828 | tags=33%, list=29%, signal=47% |
| 230 | RHO\_PROTEIN\_SIGNAL\_TRANSDUCTION |  | 30 | 0.15 | 0.56 | 0.976 | 1.000 | 1.000 | 2826 | tags=23%, list=22%, signal=30% |
| 231 | RESPONSE\_TO\_LIGHT\_STIMULUS |  | 40 | 0.12 | 0.52 | 0.996 | 1.000 | 1.000 | 3279 | tags=25%, list=25%, signal=33% |
| 232 | DETECTION\_OF\_ABIOTIC\_STIMULUS |  | 16 | 0.16 | 0.52 | 0.985 | 0.999 | 1.000 | 64 | tags=6%, list=0%, signal=6% |
| 233 | NEGATIVE\_REGULATION\_OF\_TRANSLATION |  | 19 | 0.15 | 0.51 | 0.990 | 0.995 | 1.000 | 3287 | tags=26%, list=25%, signal=35% |
Table: Gene sets enriched in phenotype **na**[plain text format]****

  
