# Supplementary material for: Multiple-input multiple-output causal strategies for gene selection
Source: BMC Bioinformatics. 2011 Nov 25;12:458. doi: 10.1186/1471-2105-12-458 (PMC3323860; doi:10.1186/1471-2105-12-458)
Supplement: Additional file 2 — Archive containing the output files computed by the preranked GSEA for λ ∈ {0.1,0.2,0.3,0.4,0.5} (GSEA_MIMO_part1.zip). [file 1471-2105-12-458-S2.ZIP › mFS05_entrez_mimo.GseaPreranked.1316038549256/gsea_report_for_na_neg_1316038549256.html]

Report for na\_neg 1316038549256 [GSEA]

| GS  follow link to MSigDB | GS DETAILS | SIZE | ES | NES | NOM p-val | FDR q-val | FWER p-val | RANK AT MAX | LEADING EDGE || 1 | IMMUNE\_RESPONSE |  | 212 | -0.40 | -2.38 | 0.000 | 0.003 | 0.002 | 2901 | tags=43%, list=22%, signal=54% |
| 2 | IMMUNE\_SYSTEM\_PROCESS |  | 298 | -0.36 | -2.30 | 0.000 | 0.004 | 0.006 | 2901 | tags=40%, list=22%, signal=51% |
| 3 | DEFENSE\_RESPONSE |  | 238 | -0.37 | -2.24 | 0.000 | 0.005 | 0.011 | 3260 | tags=41%, list=25%, signal=53% |
| 4 | POSITIVE\_REGULATION\_OF\_IMMUNE\_RESPONSE |  | 24 | -0.56 | -2.10 | 0.000 | 0.016 | 0.048 | 2901 | tags=58%, list=22%, signal=75% |
| 5 | REGULATION\_OF\_IMMUNE\_RESPONSE |  | 28 | -0.52 | -2.10 | 0.000 | 0.013 | 0.048 | 4576 | tags=75%, list=35%, signal=115% |
| 6 | POSITIVE\_REGULATION\_OF\_IMMUNE\_SYSTEM\_PROCESS |  | 44 | -0.47 | -2.06 | 0.000 | 0.016 | 0.070 | 4123 | tags=61%, list=31%, signal=89% |
| 7 | INFLAMMATORY\_RESPONSE |  | 115 | -0.38 | -2.05 | 0.000 | 0.016 | 0.082 | 2973 | tags=41%, list=23%, signal=52% |
| 8 | POSITIVE\_REGULATION\_OF\_MULTICELLULAR\_ORGANISMAL\_PROCESS |  | 56 | -0.44 | -2.03 | 0.000 | 0.017 | 0.104 | 3237 | tags=50%, list=25%, signal=66% |
| 9 | RESPONSE\_TO\_WOUNDING |  | 171 | -0.35 | -2.01 | 0.000 | 0.020 | 0.126 | 3425 | tags=42%, list=26%, signal=56% |
| 10 | REGULATION\_OF\_IMMUNE\_SYSTEM\_PROCESS |  | 57 | -0.42 | -1.97 | 0.000 | 0.024 | 0.168 | 4123 | tags=60%, list=31%, signal=87% |
| 11 | CELLULAR\_DEFENSE\_RESPONSE |  | 54 | -0.43 | -1.96 | 0.000 | 0.024 | 0.186 | 3582 | tags=48%, list=27%, signal=66% |
| 12 | ADAPTIVE\_IMMUNE\_RESPONSE\_GO\_0002460 |  | 22 | -0.49 | -1.78 | 0.014 | 0.105 | 0.629 | 4107 | tags=59%, list=31%, signal=86% |
| 13 | HEMOPOIETIC\_OR\_LYMPHOID\_ORGAN\_DEVELOPMENT |  | 71 | -0.35 | -1.74 | 0.000 | 0.135 | 0.740 | 2880 | tags=39%, list=22%, signal=50% |
| 14 | ADAPTIVE\_IMMUNE\_RESPONSE |  | 23 | -0.46 | -1.74 | 0.009 | 0.128 | 0.749 | 3237 | tags=48%, list=25%, signal=63% |
| 15 | HEMOPOIESIS |  | 69 | -0.35 | -1.73 | 0.004 | 0.128 | 0.776 | 2880 | tags=39%, list=22%, signal=50% |
| 16 | REGULATION\_OF\_MULTICELLULAR\_ORGANISMAL\_PROCESS |  | 131 | -0.31 | -1.72 | 0.002 | 0.132 | 0.810 | 3237 | tags=39%, list=25%, signal=51% |
| 17 | IMMUNE\_SYSTEM\_DEVELOPMENT |  | 75 | -0.33 | -1.69 | 0.002 | 0.163 | 0.872 | 2880 | tags=39%, list=22%, signal=49% |
| 18 | RESPONSE\_TO\_EXTERNAL\_STIMULUS |  | 278 | -0.27 | -1.68 | 0.000 | 0.160 | 0.886 | 2384 | tags=28%, list=18%, signal=34% |
| 19 | RECEPTOR\_MEDIATED\_ENDOCYTOSIS |  | 31 | -0.43 | -1.68 | 0.009 | 0.152 | 0.889 | 2098 | tags=35%, list=16%, signal=42% |
| 20 | IMMUNE\_EFFECTOR\_PROCESS |  | 34 | -0.40 | -1.68 | 0.009 | 0.147 | 0.893 | 4107 | tags=65%, list=31%, signal=94% |
| 21 | HUMORAL\_IMMUNE\_RESPONSE |  | 30 | -0.42 | -1.68 | 0.013 | 0.143 | 0.897 | 3153 | tags=53%, list=24%, signal=70% |
| 22 | REGULATION\_OF\_DEFENSE\_RESPONSE |  | 15 | -0.52 | -1.67 | 0.027 | 0.148 | 0.918 | 4107 | tags=67%, list=31%, signal=97% |
| 23 | LIPID\_CATABOLIC\_PROCESS |  | 34 | -0.40 | -1.67 | 0.011 | 0.142 | 0.919 | 3993 | tags=56%, list=31%, signal=80% |
| 24 | LYMPHOCYTE\_ACTIVATION |  | 54 | -0.36 | -1.66 | 0.002 | 0.139 | 0.920 | 3237 | tags=46%, list=25%, signal=61% |
| 25 | JAK\_STAT\_CASCADE |  | 26 | -0.42 | -1.64 | 0.028 | 0.159 | 0.947 | 1655 | tags=35%, list=13%, signal=40% |
| 26 | REGULATION\_OF\_CELL\_DIFFERENTIATION |  | 48 | -0.36 | -1.62 | 0.007 | 0.175 | 0.964 | 4859 | tags=58%, list=37%, signal=92% |
| 27 | TRANSFORMING\_GROWTH\_FACTOR\_BETA\_RECEPTOR\_SIGNALING\_PATHWAY |  | 34 | -0.39 | -1.62 | 0.020 | 0.169 | 0.964 | 3078 | tags=44%, list=24%, signal=58% |
| 28 | POSITIVE\_REGULATION\_OF\_RESPONSE\_TO\_STIMULUS |  | 35 | -0.38 | -1.61 | 0.021 | 0.176 | 0.971 | 2901 | tags=46%, list=22%, signal=59% |
| 29 | LEUKOCYTE\_DIFFERENTIATION |  | 34 | -0.39 | -1.60 | 0.007 | 0.184 | 0.983 | 2853 | tags=44%, list=22%, signal=56% |
| 30 | T\_CELL\_ACTIVATION |  | 39 | -0.37 | -1.58 | 0.027 | 0.197 | 0.987 | 3217 | tags=44%, list=25%, signal=58% |
| 31 | LEUKOCYTE\_ACTIVATION |  | 59 | -0.33 | -1.56 | 0.009 | 0.215 | 0.992 | 3237 | tags=44%, list=25%, signal=58% |
| 32 | CELL\_ACTIVATION |  | 64 | -0.32 | -1.55 | 0.007 | 0.227 | 0.997 | 3545 | tags=45%, list=27%, signal=62% |
| 33 | REGULATION\_OF\_ANGIOGENESIS |  | 24 | -0.41 | -1.54 | 0.039 | 0.232 | 0.999 | 2287 | tags=46%, list=17%, signal=55% |
| 34 | TRANSMEMBRANE\_RECEPTOR\_PROTEIN\_SERINE\_THREONINE\_KINASE\_SIGNALING\_PATHWAY |  | 42 | -0.35 | -1.54 | 0.022 | 0.229 | 0.999 | 3104 | tags=40%, list=24%, signal=53% |
| 35 | ENZYME\_LINKED\_RECEPTOR\_PROTEIN\_SIGNALING\_PATHWAY |  | 128 | -0.28 | -1.54 | 0.005 | 0.230 | 0.999 | 1441 | tags=22%, list=11%, signal=24% |
| 36 | ACTIN\_CYTOSKELETON\_ORGANIZATION\_AND\_BIOGENESIS |  | 90 | -0.29 | -1.51 | 0.015 | 0.259 | 0.999 | 3005 | tags=33%, list=23%, signal=43% |
| 37 | LYMPHOCYTE\_DIFFERENTIATION |  | 23 | -0.41 | -1.51 | 0.036 | 0.256 | 0.999 | 3217 | tags=52%, list=25%, signal=69% |
| 38 | CELLULAR\_LIPID\_CATABOLIC\_PROCESS |  | 31 | -0.37 | -1.49 | 0.046 | 0.280 | 0.999 | 3986 | tags=52%, list=30%, signal=74% |
| 39 | B\_CELL\_ACTIVATION |  | 17 | -0.44 | -1.49 | 0.052 | 0.273 | 0.999 | 3237 | tags=59%, list=25%, signal=78% |
| 40 | REGULATION\_OF\_RESPONSE\_TO\_STIMULUS |  | 49 | -0.33 | -1.48 | 0.024 | 0.292 | 1.000 | 4107 | tags=55%, list=31%, signal=80% |
| 41 | POSITIVE\_REGULATION\_OF\_PHOSPHATE\_METABOLIC\_PROCESS |  | 23 | -0.39 | -1.47 | 0.044 | 0.299 | 1.000 | 1464 | tags=30%, list=11%, signal=34% |
| 42 | NEGATIVE\_REGULATION\_OF\_SIGNAL\_TRANSDUCTION |  | 31 | -0.36 | -1.46 | 0.050 | 0.307 | 1.000 | 4690 | tags=55%, list=36%, signal=85% |
| 43 | MULTI\_ORGANISM\_PROCESS |  | 137 | -0.26 | -1.46 | 0.007 | 0.303 | 1.000 | 4173 | tags=47%, list=32%, signal=68% |
| 44 | PROTEIN\_AMINO\_ACID\_N\_LINKED\_GLYCOSYLATION |  | 27 | -0.37 | -1.46 | 0.045 | 0.300 | 1.000 | 2441 | tags=37%, list=19%, signal=45% |
| 45 | INNATE\_IMMUNE\_RESPONSE |  | 19 | -0.41 | -1.46 | 0.058 | 0.295 | 1.000 | 4825 | tags=74%, list=37%, signal=117% |
| 46 | POSITIVE\_REGULATION\_OF\_CELL\_DIFFERENTIATION |  | 21 | -0.40 | -1.46 | 0.066 | 0.290 | 1.000 | 4470 | tags=67%, list=34%, signal=101% |
| 47 | CELL\_SUBSTRATE\_ADHESION |  | 36 | -0.34 | -1.45 | 0.059 | 0.307 | 1.000 | 1716 | tags=31%, list=13%, signal=35% |
| 48 | CYTOKINE\_AND\_CHEMOKINE\_MEDIATED\_SIGNALING\_PATHWAY |  | 19 | -0.41 | -1.44 | 0.063 | 0.305 | 1.000 | 2299 | tags=37%, list=18%, signal=45% |
| 49 | CATION\_HOMEOSTASIS |  | 94 | -0.27 | -1.44 | 0.023 | 0.299 | 1.000 | 3156 | tags=36%, list=24%, signal=47% |
| 50 | REGULATION\_OF\_PROTEIN\_AMINO\_ACID\_PHOSPHORYLATION |  | 23 | -0.39 | -1.44 | 0.065 | 0.295 | 1.000 | 2562 | tags=35%, list=20%, signal=43% |
| 51 | FATTY\_ACID\_METABOLIC\_PROCESS |  | 56 | -0.31 | -1.44 | 0.048 | 0.294 | 1.000 | 3986 | tags=50%, list=30%, signal=72% |
| 52 | PEPTIDYL\_TYROSINE\_MODIFICATION |  | 23 | -0.39 | -1.44 | 0.060 | 0.294 | 1.000 | 1513 | tags=26%, list=12%, signal=29% |
| 53 | REGULATION\_OF\_SIGNAL\_TRANSDUCTION |  | 173 | -0.24 | -1.43 | 0.022 | 0.304 | 1.000 | 3366 | tags=35%, list=26%, signal=47% |
| 54 | MAINTENANCE\_OF\_LOCALIZATION |  | 21 | -0.40 | -1.42 | 0.071 | 0.306 | 1.000 | 2604 | tags=38%, list=20%, signal=47% |
| 55 | POSITIVE\_REGULATION\_OF\_SIGNAL\_TRANSDUCTION |  | 97 | -0.27 | -1.42 | 0.014 | 0.301 | 1.000 | 3706 | tags=42%, list=28%, signal=59% |
| 56 | MESODERM\_DEVELOPMENT |  | 22 | -0.39 | -1.42 | 0.077 | 0.300 | 1.000 | 4013 | tags=50%, list=31%, signal=72% |
| 57 | WOUND\_HEALING |  | 49 | -0.31 | -1.42 | 0.049 | 0.298 | 1.000 | 3425 | tags=39%, list=26%, signal=52% |
| 58 | REGULATION\_OF\_LYMPHOCYTE\_ACTIVATION |  | 31 | -0.36 | -1.41 | 0.068 | 0.303 | 1.000 | 3217 | tags=45%, list=25%, signal=60% |
| 59 | CELLULAR\_CATION\_HOMEOSTASIS |  | 91 | -0.28 | -1.41 | 0.037 | 0.300 | 1.000 | 3156 | tags=36%, list=24%, signal=47% |
| 60 | SMALL\_GTPASE\_MEDIATED\_SIGNAL\_TRANSDUCTION |  | 77 | -0.28 | -1.40 | 0.024 | 0.308 | 1.000 | 2718 | tags=34%, list=21%, signal=42% |
| 61 | COAGULATION |  | 41 | -0.32 | -1.40 | 0.052 | 0.307 | 1.000 | 3425 | tags=39%, list=26%, signal=53% |
| 62 | RESPONSE\_TO\_OTHER\_ORGANISM |  | 69 | -0.29 | -1.40 | 0.057 | 0.308 | 1.000 | 2838 | tags=36%, list=22%, signal=46% |
| 63 | BLOOD\_COAGULATION |  | 41 | -0.32 | -1.40 | 0.079 | 0.305 | 1.000 | 3425 | tags=39%, list=26%, signal=53% |
| 64 | PROTEIN\_AMINO\_ACID\_PHOSPHORYLATION |  | 231 | -0.23 | -1.39 | 0.005 | 0.310 | 1.000 | 3060 | tags=30%, list=23%, signal=38% |
| 65 | DETECTION\_OF\_STIMULUS |  | 36 | -0.33 | -1.39 | 0.071 | 0.308 | 1.000 | 4971 | tags=56%, list=38%, signal=89% |
| 66 | TRANSMEMBRANE\_RECEPTOR\_PROTEIN\_TYROSINE\_KINASE\_SIGNALING\_PATHWAY |  | 76 | -0.28 | -1.38 | 0.046 | 0.321 | 1.000 | 1359 | tags=21%, list=10%, signal=23% |
| 67 | POSITIVE\_REGULATION\_OF\_PROTEIN\_AMINO\_ACID\_PHOSPHORYLATION |  | 15 | -0.42 | -1.36 | 0.098 | 0.351 | 1.000 | 1464 | tags=33%, list=11%, signal=37% |
| 68 | REGULATION\_OF\_T\_CELL\_ACTIVATION |  | 25 | -0.35 | -1.36 | 0.083 | 0.358 | 1.000 | 3217 | tags=44%, list=25%, signal=58% |
| 69 | PROTEIN\_KINASE\_CASCADE |  | 239 | -0.22 | -1.36 | 0.015 | 0.353 | 1.000 | 2406 | tags=26%, list=18%, signal=32% |
| 70 | POSITIVE\_REGULATION\_OF\_LYMPHOCYTE\_ACTIVATION |  | 23 | -0.37 | -1.36 | 0.091 | 0.349 | 1.000 | 3217 | tags=43%, list=25%, signal=58% |
| 71 | GLYCOPROTEIN\_METABOLIC\_PROCESS |  | 82 | -0.26 | -1.35 | 0.033 | 0.356 | 1.000 | 3778 | tags=40%, list=29%, signal=56% |
| 72 | FEMALE\_PREGNANCY |  | 42 | -0.31 | -1.35 | 0.103 | 0.357 | 1.000 | 4158 | tags=52%, list=32%, signal=77% |
| 73 | REGULATION\_OF\_CYTOSKELETON\_ORGANIZATION\_AND\_BIOGENESIS |  | 26 | -0.35 | -1.34 | 0.103 | 0.361 | 1.000 | 2562 | tags=35%, list=20%, signal=43% |
| 74 | PROTEIN\_COMPLEX\_ASSEMBLY |  | 157 | -0.23 | -1.34 | 0.032 | 0.371 | 1.000 | 2899 | tags=30%, list=22%, signal=38% |
| 75 | REGULATION\_OF\_BLOOD\_PRESSURE |  | 22 | -0.36 | -1.34 | 0.123 | 0.370 | 1.000 | 3503 | tags=41%, list=27%, signal=56% |
| 76 | GROWTH |  | 59 | -0.28 | -1.33 | 0.089 | 0.367 | 1.000 | 4391 | tags=46%, list=34%, signal=69% |
| 77 | POSITIVE\_REGULATION\_OF\_CYTOKINE\_BIOSYNTHETIC\_PROCESS |  | 21 | -0.37 | -1.33 | 0.118 | 0.368 | 1.000 | 2856 | tags=43%, list=22%, signal=55% |
| 78 | AMINE\_TRANSPORT |  | 36 | -0.32 | -1.33 | 0.110 | 0.368 | 1.000 | 2362 | tags=28%, list=18%, signal=34% |
| 79 | RESPONSE\_TO\_VIRUS |  | 45 | -0.30 | -1.32 | 0.101 | 0.375 | 1.000 | 3032 | tags=42%, list=23%, signal=55% |
| 80 | ACTIN\_POLYMERIZATION\_AND\_OR\_DEPOLYMERIZATION |  | 20 | -0.37 | -1.32 | 0.136 | 0.371 | 1.000 | 2348 | tags=30%, list=18%, signal=37% |
| 81 | MUSCLE\_DEVELOPMENT |  | 85 | -0.26 | -1.32 | 0.059 | 0.366 | 1.000 | 3519 | tags=41%, list=27%, signal=56% |
| 82 | CELL\_MATRIX\_ADHESION |  | 35 | -0.31 | -1.31 | 0.112 | 0.381 | 1.000 | 1716 | tags=29%, list=13%, signal=33% |
| 83 | CYTOKINE\_PRODUCTION |  | 61 | -0.28 | -1.31 | 0.069 | 0.388 | 1.000 | 2856 | tags=34%, list=22%, signal=44% |
| 84 | PEPTIDYL\_TYROSINE\_PHOSPHORYLATION |  | 21 | -0.35 | -1.30 | 0.142 | 0.390 | 1.000 | 1513 | tags=24%, list=12%, signal=27% |
| 85 | ACTIN\_FILAMENT\_BASED\_PROCESS |  | 99 | -0.25 | -1.30 | 0.074 | 0.392 | 1.000 | 3005 | tags=30%, list=23%, signal=39% |
| 86 | MONOCARBOXYLIC\_ACID\_METABOLIC\_PROCESS |  | 77 | -0.26 | -1.29 | 0.068 | 0.404 | 1.000 | 3986 | tags=44%, list=30%, signal=63% |
| 87 | RAS\_PROTEIN\_SIGNAL\_TRANSDUCTION |  | 55 | -0.28 | -1.29 | 0.105 | 0.403 | 1.000 | 3345 | tags=40%, list=26%, signal=54% |
| 88 | HEMOSTASIS |  | 46 | -0.29 | -1.28 | 0.094 | 0.418 | 1.000 | 3425 | tags=37%, list=26%, signal=50% |
| 89 | ANATOMICAL\_STRUCTURE\_FORMATION |  | 52 | -0.27 | -1.28 | 0.115 | 0.416 | 1.000 | 2522 | tags=33%, list=19%, signal=40% |
| 90 | POSITIVE\_REGULATION\_OF\_PHOSPHORYLATION |  | 21 | -0.36 | -1.28 | 0.158 | 0.413 | 1.000 | 1464 | tags=29%, list=11%, signal=32% |
| 91 | PROTEIN\_AMINO\_ACID\_DEPHOSPHORYLATION |  | 60 | -0.27 | -1.28 | 0.121 | 0.413 | 1.000 | 1629 | tags=22%, list=12%, signal=25% |
| 92 | RESPONSE\_TO\_DRUG |  | 21 | -0.36 | -1.28 | 0.158 | 0.411 | 1.000 | 2620 | tags=43%, list=20%, signal=53% |
| 93 | REGULATION\_OF\_CELL\_PROLIFERATION |  | 275 | -0.20 | -1.27 | 0.018 | 0.417 | 1.000 | 2657 | tags=26%, list=20%, signal=32% |
| 94 | AMINO\_ACID\_TRANSPORT |  | 25 | -0.33 | -1.27 | 0.158 | 0.414 | 1.000 | 2362 | tags=32%, list=18%, signal=39% |
| 95 | REGULATION\_OF\_ANATOMICAL\_STRUCTURE\_MORPHOGENESIS |  | 17 | -0.37 | -1.26 | 0.149 | 0.436 | 1.000 | 5028 | tags=53%, list=38%, signal=86% |
| 96 | REGULATION\_OF\_BODY\_FLUID\_LEVELS |  | 55 | -0.27 | -1.26 | 0.123 | 0.434 | 1.000 | 3425 | tags=36%, list=26%, signal=49% |
| 97 | POSITIVE\_REGULATION\_OF\_TRANSFERASE\_ACTIVITY |  | 71 | -0.26 | -1.26 | 0.104 | 0.434 | 1.000 | 2362 | tags=27%, list=18%, signal=32% |
| 98 | ANGIOGENESIS |  | 44 | -0.29 | -1.25 | 0.143 | 0.441 | 1.000 | 2522 | tags=34%, list=19%, signal=42% |
| 99 | DEPHOSPHORYLATION |  | 67 | -0.25 | -1.25 | 0.124 | 0.444 | 1.000 | 1629 | tags=21%, list=12%, signal=24% |
| 100 | POSITIVE\_REGULATION\_OF\_CELL\_PROLIFERATION |  | 129 | -0.22 | -1.24 | 0.094 | 0.450 | 1.000 | 1864 | tags=22%, list=14%, signal=26% |
| 101 | REGULATION\_OF\_I\_KAPPAB\_KINASE\_NF\_KAPPAB\_CASCADE |  | 72 | -0.25 | -1.24 | 0.123 | 0.456 | 1.000 | 3698 | tags=43%, list=28%, signal=60% |
| 102 | POSITIVE\_REGULATION\_OF\_TRANSLATION |  | 28 | -0.31 | -1.24 | 0.170 | 0.459 | 1.000 | 2856 | tags=39%, list=22%, signal=50% |
| 103 | PROTEIN\_OLIGOMERIZATION |  | 37 | -0.30 | -1.23 | 0.150 | 0.476 | 1.000 | 2726 | tags=32%, list=21%, signal=41% |
| 104 | NEGATIVE\_REGULATION\_OF\_TRANSCRIPTION |  | 166 | -0.21 | -1.23 | 0.097 | 0.473 | 1.000 | 2822 | tags=29%, list=22%, signal=36% |
| 105 | G\_PROTEIN\_SIGNALING\_COUPLED\_TO\_CAMP\_NUCLEOTIDE\_SECOND\_MESSENGER |  | 62 | -0.26 | -1.23 | 0.155 | 0.472 | 1.000 | 2010 | tags=21%, list=15%, signal=25% |
| 106 | PHOSPHOLIPID\_METABOLIC\_PROCESS |  | 63 | -0.25 | -1.23 | 0.132 | 0.470 | 1.000 | 3794 | tags=41%, list=29%, signal=58% |
| 107 | CAMP\_MEDIATED\_SIGNALING |  | 63 | -0.26 | -1.22 | 0.146 | 0.467 | 1.000 | 2010 | tags=21%, list=15%, signal=24% |
| 108 | ICOSANOID\_METABOLIC\_PROCESS |  | 16 | -0.38 | -1.22 | 0.226 | 0.465 | 1.000 | 3078 | tags=44%, list=24%, signal=57% |
| 109 | LIPID\_METABOLIC\_PROCESS |  | 283 | -0.20 | -1.22 | 0.070 | 0.465 | 1.000 | 3993 | tags=39%, list=31%, signal=55% |
| 110 | MUSCLE\_CELL\_DIFFERENTIATION |  | 21 | -0.34 | -1.22 | 0.205 | 0.467 | 1.000 | 3352 | tags=48%, list=26%, signal=64% |
| 111 | NEURON\_DIFFERENTIATION |  | 58 | -0.26 | -1.22 | 0.149 | 0.464 | 1.000 | 3371 | tags=33%, list=26%, signal=44% |
| 112 | POSITIVE\_REGULATION\_OF\_CELLULAR\_PROTEIN\_METABOLIC\_PROCESS |  | 61 | -0.25 | -1.21 | 0.154 | 0.474 | 1.000 | 2348 | tags=30%, list=18%, signal=36% |
| 113 | CELL\_RECOGNITION |  | 16 | -0.36 | -1.21 | 0.226 | 0.477 | 1.000 | 4522 | tags=56%, list=35%, signal=86% |
| 114 | ORGAN\_MORPHOGENESIS |  | 131 | -0.22 | -1.21 | 0.116 | 0.486 | 1.000 | 1508 | tags=19%, list=12%, signal=21% |
| 115 | BEHAVIOR |  | 136 | -0.21 | -1.20 | 0.135 | 0.482 | 1.000 | 4142 | tags=39%, list=32%, signal=56% |
| 116 | GENERATION\_OF\_NEURONS |  | 65 | -0.25 | -1.20 | 0.143 | 0.482 | 1.000 | 3984 | tags=38%, list=30%, signal=55% |
| 117 | PHOSPHORYLATION |  | 262 | -0.20 | -1.20 | 0.097 | 0.479 | 1.000 | 2897 | tags=27%, list=22%, signal=35% |
| 118 | ACTIVATION\_OF\_NF\_KAPPAB\_TRANSCRIPTION\_FACTOR |  | 15 | -0.37 | -1.20 | 0.209 | 0.479 | 1.000 | 4173 | tags=60%, list=32%, signal=88% |
| 119 | RESPONSE\_TO\_BACTERIUM |  | 22 | -0.32 | -1.20 | 0.229 | 0.478 | 1.000 | 1827 | tags=27%, list=14%, signal=32% |
| 120 | REGULATION\_OF\_ORGANELLE\_ORGANIZATION\_AND\_BIOGENESIS |  | 35 | -0.29 | -1.20 | 0.217 | 0.484 | 1.000 | 2562 | tags=31%, list=20%, signal=39% |
| 121 | POSITIVE\_REGULATION\_OF\_PROTEIN\_METABOLIC\_PROCESS |  | 63 | -0.25 | -1.20 | 0.183 | 0.481 | 1.000 | 2608 | tags=32%, list=20%, signal=39% |
| 122 | POSITIVE\_REGULATION\_OF\_SECRETION |  | 18 | -0.35 | -1.19 | 0.230 | 0.483 | 1.000 | 4521 | tags=61%, list=35%, signal=93% |
| 123 | SKELETAL\_DEVELOPMENT |  | 91 | -0.23 | -1.19 | 0.155 | 0.483 | 1.000 | 2963 | tags=32%, list=23%, signal=41% |
| 124 | REGULATION\_OF\_PROTEIN\_IMPORT\_INTO\_NUCLEUS |  | 15 | -0.36 | -1.19 | 0.223 | 0.484 | 1.000 | 1354 | tags=27%, list=10%, signal=30% |
| 125 | DEVELOPMENTAL\_MATURATION |  | 18 | -0.35 | -1.18 | 0.229 | 0.494 | 1.000 | 3005 | tags=39%, list=23%, signal=50% |
| 126 | BONE\_REMODELING |  | 28 | -0.30 | -1.17 | 0.242 | 0.522 | 1.000 | 2793 | tags=32%, list=21%, signal=41% |
| 127 | REGULATION\_OF\_PROTEIN\_METABOLIC\_PROCESS |  | 150 | -0.21 | -1.17 | 0.152 | 0.524 | 1.000 | 2403 | tags=25%, list=18%, signal=31% |
| 128 | VASCULATURE\_DEVELOPMENT |  | 50 | -0.25 | -1.17 | 0.199 | 0.522 | 1.000 | 2522 | tags=30%, list=19%, signal=37% |
| 129 | GENERATION\_OF\_PRECURSOR\_METABOLITES\_AND\_ENERGY |  | 120 | -0.22 | -1.17 | 0.160 | 0.521 | 1.000 | 3058 | tags=31%, list=23%, signal=40% |
| 130 | POSITIVE\_REGULATION\_OF\_I\_KAPPAB\_KINASE\_NF\_KAPPAB\_CASCADE |  | 67 | -0.24 | -1.17 | 0.180 | 0.520 | 1.000 | 3698 | tags=42%, list=28%, signal=58% |
| 131 | POSITIVE\_REGULATION\_OF\_T\_CELL\_ACTIVATION |  | 20 | -0.33 | -1.16 | 0.263 | 0.523 | 1.000 | 4576 | tags=55%, list=35%, signal=84% |
| 132 | PROTEIN\_PROCESSING |  | 41 | -0.27 | -1.16 | 0.223 | 0.528 | 1.000 | 4260 | tags=41%, list=33%, signal=61% |
| 133 | FATTY\_ACID\_OXIDATION |  | 17 | -0.34 | -1.15 | 0.256 | 0.540 | 1.000 | 3243 | tags=47%, list=25%, signal=62% |
| 134 | STRIATED\_MUSCLE\_DEVELOPMENT |  | 36 | -0.28 | -1.15 | 0.250 | 0.544 | 1.000 | 3519 | tags=44%, list=27%, signal=61% |
| 135 | NEURON\_DEVELOPMENT |  | 49 | -0.25 | -1.15 | 0.208 | 0.545 | 1.000 | 3371 | tags=33%, list=26%, signal=44% |
| 136 | CYTOKINE\_BIOSYNTHETIC\_PROCESS |  | 34 | -0.28 | -1.14 | 0.249 | 0.554 | 1.000 | 2856 | tags=35%, list=22%, signal=45% |
| 137 | DEFENSE\_RESPONSE\_TO\_BACTERIUM |  | 16 | -0.34 | -1.14 | 0.274 | 0.551 | 1.000 | 3940 | tags=44%, list=30%, signal=63% |
| 138 | REGULATION\_OF\_MYELOID\_CELL\_DIFFERENTIATION |  | 19 | -0.33 | -1.14 | 0.260 | 0.551 | 1.000 | 4576 | tags=58%, list=35%, signal=89% |
| 139 | NEGATIVE\_REGULATION\_OF\_RNA\_METABOLIC\_PROCESS |  | 114 | -0.21 | -1.14 | 0.187 | 0.549 | 1.000 | 2822 | tags=29%, list=22%, signal=37% |
| 140 | MYELOID\_CELL\_DIFFERENTIATION |  | 35 | -0.27 | -1.14 | 0.258 | 0.547 | 1.000 | 2880 | tags=31%, list=22%, signal=40% |
| 141 | GLYCEROPHOSPHOLIPID\_METABOLIC\_PROCESS |  | 39 | -0.27 | -1.14 | 0.272 | 0.543 | 1.000 | 4690 | tags=54%, list=36%, signal=84% |
| 142 | I\_KAPPAB\_KINASE\_NF\_KAPPAB\_CASCADE |  | 88 | -0.22 | -1.14 | 0.203 | 0.540 | 1.000 | 3698 | tags=40%, list=28%, signal=55% |
| 143 | GLYCOPROTEIN\_BIOSYNTHETIC\_PROCESS |  | 67 | -0.24 | -1.14 | 0.227 | 0.537 | 1.000 | 3778 | tags=39%, list=29%, signal=54% |
| 144 | POSITIVE\_REGULATION\_OF\_PROTEIN\_MODIFICATION\_PROCESS |  | 24 | -0.30 | -1.14 | 0.261 | 0.537 | 1.000 | 1464 | tags=25%, list=11%, signal=28% |
| 145 | REGULATION\_OF\_MAP\_KINASE\_ACTIVITY |  | 56 | -0.24 | -1.14 | 0.255 | 0.534 | 1.000 | 1920 | tags=27%, list=15%, signal=31% |
| 146 | PROTEIN\_SECRETION |  | 28 | -0.28 | -1.14 | 0.282 | 0.532 | 1.000 | 4107 | tags=46%, list=31%, signal=68% |
| 147 | ION\_HOMEOSTASIS |  | 112 | -0.21 | -1.13 | 0.189 | 0.533 | 1.000 | 3156 | tags=32%, list=24%, signal=42% |
| 148 | NEGATIVE\_REGULATION\_OF\_CELL\_PROLIFERATION |  | 145 | -0.20 | -1.13 | 0.178 | 0.534 | 1.000 | 2653 | tags=26%, list=20%, signal=33% |
| 149 | REGULATION\_OF\_MAPKKK\_CASCADE |  | 19 | -0.33 | -1.13 | 0.304 | 0.531 | 1.000 | 2387 | tags=32%, list=18%, signal=39% |
| 150 | REGULATION\_OF\_CELLULAR\_PROTEIN\_METABOLIC\_PROCESS |  | 139 | -0.20 | -1.13 | 0.214 | 0.531 | 1.000 | 3058 | tags=29%, list=23%, signal=38% |
| 151 | VITAMIN\_METABOLIC\_PROCESS |  | 15 | -0.35 | -1.13 | 0.286 | 0.534 | 1.000 | 4130 | tags=60%, list=32%, signal=88% |
| 152 | NEGATIVE\_REGULATION\_OF\_TRANSCRIPTION\_DNA\_DEPENDENT |  | 114 | -0.21 | -1.13 | 0.230 | 0.534 | 1.000 | 2822 | tags=29%, list=22%, signal=37% |
| 153 | MEMBRANE\_ORGANIZATION\_AND\_BIOGENESIS |  | 124 | -0.20 | -1.12 | 0.220 | 0.540 | 1.000 | 2098 | tags=23%, list=16%, signal=28% |
| 154 | RESPONSE\_TO\_BIOTIC\_STIMULUS |  | 103 | -0.21 | -1.12 | 0.240 | 0.539 | 1.000 | 2185 | tags=26%, list=17%, signal=31% |
| 155 | NEGATIVE\_REGULATION\_OF\_NUCLEOBASENUCLEOSIDENUCLEOTIDE\_AND\_NUCLEIC\_ACID\_METABOLIC\_PROCESS |  | 185 | -0.19 | -1.12 | 0.190 | 0.541 | 1.000 | 2822 | tags=28%, list=22%, signal=35% |
| 156 | ORGANIC\_ACID\_METABOLIC\_PROCESS |  | 162 | -0.19 | -1.12 | 0.210 | 0.540 | 1.000 | 3986 | tags=38%, list=30%, signal=54% |
| 157 | ACTIVATION\_OF\_MAPK\_ACTIVITY |  | 33 | -0.27 | -1.12 | 0.275 | 0.540 | 1.000 | 2250 | tags=30%, list=17%, signal=37% |
| 158 | CARBOXYLIC\_ACID\_METABOLIC\_PROCESS |  | 160 | -0.19 | -1.12 | 0.207 | 0.537 | 1.000 | 3986 | tags=39%, list=30%, signal=55% |
| 159 | AMINO\_ACID\_DERIVATIVE\_METABOLIC\_PROCESS |  | 23 | -0.30 | -1.12 | 0.287 | 0.536 | 1.000 | 3986 | tags=48%, list=30%, signal=69% |
| 160 | POST\_TRANSLATIONAL\_PROTEIN\_MODIFICATION |  | 409 | -0.17 | -1.11 | 0.138 | 0.538 | 1.000 | 2900 | tags=26%, list=22%, signal=32% |
| 161 | PROTEIN\_AUTOPROCESSING |  | 24 | -0.29 | -1.11 | 0.296 | 0.539 | 1.000 | 5182 | tags=58%, list=40%, signal=96% |
| 162 | CELL\_MATURATION |  | 16 | -0.33 | -1.11 | 0.326 | 0.545 | 1.000 | 3005 | tags=38%, list=23%, signal=49% |
| 163 | NEGATIVE\_REGULATION\_OF\_METABOLIC\_PROCESS |  | 232 | -0.18 | -1.11 | 0.206 | 0.547 | 1.000 | 2689 | tags=25%, list=21%, signal=31% |
| 164 | PHAGOCYTOSIS |  | 16 | -0.34 | -1.11 | 0.294 | 0.544 | 1.000 | 4101 | tags=56%, list=31%, signal=82% |
| 165 | CYTOKINE\_SECRETION |  | 15 | -0.35 | -1.11 | 0.321 | 0.543 | 1.000 | 3442 | tags=47%, list=26%, signal=63% |
| 166 | TISSUE\_REMODELING |  | 29 | -0.28 | -1.10 | 0.309 | 0.544 | 1.000 | 2793 | tags=31%, list=21%, signal=39% |
| 167 | NEURITE\_DEVELOPMENT |  | 41 | -0.25 | -1.10 | 0.310 | 0.543 | 1.000 | 3371 | tags=32%, list=26%, signal=43% |
| 168 | PROTEIN\_AMINO\_ACID\_AUTOPHOSPHORYLATION |  | 24 | -0.29 | -1.10 | 0.342 | 0.550 | 1.000 | 5182 | tags=58%, list=40%, signal=96% |
| 169 | NEGATIVE\_REGULATION\_OF\_CELL\_DIFFERENTIATION |  | 24 | -0.29 | -1.09 | 0.329 | 0.566 | 1.000 | 2822 | tags=29%, list=22%, signal=37% |
| 170 | REGULATION\_OF\_DEVELOPMENTAL\_PROCESS |  | 387 | -0.17 | -1.09 | 0.200 | 0.564 | 1.000 | 4449 | tags=43%, list=34%, signal=63% |
| 171 | AMINO\_ACID\_CATABOLIC\_PROCESS |  | 23 | -0.30 | -1.09 | 0.334 | 0.572 | 1.000 | 1923 | tags=30%, list=15%, signal=36% |
| 172 | ELECTRON\_TRANSPORT\_GO\_0006118 |  | 50 | -0.24 | -1.09 | 0.298 | 0.570 | 1.000 | 2211 | tags=26%, list=17%, signal=31% |
| 173 | CYTOKINE\_METABOLIC\_PROCESS |  | 35 | -0.26 | -1.09 | 0.303 | 0.569 | 1.000 | 2856 | tags=34%, list=22%, signal=44% |
| 174 | LOCOMOTORY\_BEHAVIOR |  | 84 | -0.21 | -1.08 | 0.310 | 0.577 | 1.000 | 2838 | tags=29%, list=22%, signal=36% |
| 175 | REGULATION\_OF\_BIOLOGICAL\_QUALITY |  | 364 | -0.17 | -1.08 | 0.237 | 0.587 | 1.000 | 3678 | tags=31%, list=28%, signal=42% |
| 176 | NEGATIVE\_REGULATION\_OF\_TRANSCRIPTION\_FROM\_RNA\_POLYMERASE\_II\_PROMOTER |  | 76 | -0.21 | -1.07 | 0.318 | 0.592 | 1.000 | 2807 | tags=29%, list=21%, signal=37% |
| 177 | AXONOGENESIS |  | 33 | -0.26 | -1.07 | 0.358 | 0.597 | 1.000 | 3371 | tags=33%, list=26%, signal=45% |
| 178 | CELLULAR\_LIPID\_METABOLIC\_PROCESS |  | 220 | -0.18 | -1.07 | 0.292 | 0.601 | 1.000 | 3899 | tags=37%, list=30%, signal=52% |
| 179 | NEGATIVE\_REGULATION\_OF\_CELLULAR\_METABOLIC\_PROCESS |  | 229 | -0.17 | -1.07 | 0.271 | 0.599 | 1.000 | 2689 | tags=25%, list=21%, signal=31% |
| 180 | CELL\_PROLIFERATION\_GO\_0008283 |  | 466 | -0.16 | -1.06 | 0.265 | 0.602 | 1.000 | 2657 | tags=24%, list=20%, signal=29% |
| 181 | POSITIVE\_REGULATION\_OF\_DEVELOPMENTAL\_PROCESS |  | 197 | -0.18 | -1.06 | 0.316 | 0.601 | 1.000 | 4253 | tags=43%, list=32%, signal=63% |
| 182 | SODIUM\_ION\_TRANSPORT |  | 17 | -0.32 | -1.06 | 0.359 | 0.599 | 1.000 | 4981 | tags=59%, list=38%, signal=95% |
| 183 | MEMBRANE\_LIPID\_METABOLIC\_PROCESS |  | 85 | -0.21 | -1.06 | 0.336 | 0.596 | 1.000 | 3794 | tags=39%, list=29%, signal=54% |
| 184 | DETECTION\_OF\_EXTERNAL\_STIMULUS |  | 18 | -0.31 | -1.06 | 0.378 | 0.599 | 1.000 | 9000 | tags=100%, list=69%, signal=320% |
| 185 | CELL\_MIGRATION |  | 82 | -0.21 | -1.06 | 0.349 | 0.596 | 1.000 | 2562 | tags=24%, list=20%, signal=30% |
| 186 | HORMONE\_METABOLIC\_PROCESS |  | 29 | -0.27 | -1.06 | 0.347 | 0.595 | 1.000 | 3879 | tags=48%, list=30%, signal=68% |
| 187 | PEPTIDYL\_AMINO\_ACID\_MODIFICATION |  | 47 | -0.24 | -1.06 | 0.354 | 0.592 | 1.000 | 2562 | tags=28%, list=20%, signal=34% |
| 188 | POSITIVE\_REGULATION\_OF\_CELLULAR\_METABOLIC\_PROCESS |  | 196 | -0.18 | -1.06 | 0.314 | 0.595 | 1.000 | 2355 | tags=23%, list=18%, signal=28% |
| 189 | AMINO\_ACID\_METABOLIC\_PROCESS |  | 73 | -0.21 | -1.06 | 0.337 | 0.592 | 1.000 | 2224 | tags=26%, list=17%, signal=31% |
| 190 | MYOBLAST\_DIFFERENTIATION |  | 16 | -0.32 | -1.06 | 0.377 | 0.593 | 1.000 | 3352 | tags=50%, list=26%, signal=67% |
| 191 | TISSUE\_DEVELOPMENT |  | 126 | -0.19 | -1.05 | 0.326 | 0.591 | 1.000 | 1615 | tags=19%, list=12%, signal=22% |
| 192 | POSITIVE\_REGULATION\_OF\_CATALYTIC\_ACTIVITY |  | 139 | -0.18 | -1.05 | 0.318 | 0.601 | 1.000 | 2410 | tags=22%, list=18%, signal=27% |
| 193 | ACTIN\_FILAMENT\_ORGANIZATION |  | 21 | -0.28 | -1.04 | 0.410 | 0.614 | 1.000 | 2562 | tags=33%, list=20%, signal=41% |
| 194 | POSITIVE\_REGULATION\_OF\_METABOLIC\_PROCESS |  | 201 | -0.17 | -1.04 | 0.367 | 0.624 | 1.000 | 2403 | tags=23%, list=18%, signal=28% |
| 195 | AMINE\_CATABOLIC\_PROCESS |  | 25 | -0.27 | -1.04 | 0.385 | 0.627 | 1.000 | 1923 | tags=28%, list=15%, signal=33% |
| 196 | POSITIVE\_REGULATION\_OF\_TRANSCRIPTION |  | 124 | -0.19 | -1.03 | 0.370 | 0.637 | 1.000 | 2904 | tags=27%, list=22%, signal=34% |
| 197 | DETECTION\_OF\_STIMULUS\_INVOLVED\_IN\_SENSORY\_PERCEPTION |  | 15 | -0.31 | -1.03 | 0.434 | 0.648 | 1.000 | 9000 | tags=100%, list=69%, signal=320% |
| 198 | RESPONSE\_TO\_CHEMICAL\_STIMULUS |  | 271 | -0.17 | -1.03 | 0.389 | 0.648 | 1.000 | 1806 | tags=18%, list=14%, signal=21% |
| 199 | POSITIVE\_REGULATION\_OF\_MAP\_KINASE\_ACTIVITY |  | 39 | -0.25 | -1.02 | 0.409 | 0.647 | 1.000 | 2250 | tags=28%, list=17%, signal=34% |
| 200 | NERVOUS\_SYSTEM\_DEVELOPMENT |  | 328 | -0.16 | -1.02 | 0.394 | 0.651 | 1.000 | 4112 | tags=34%, list=31%, signal=49% |
| 201 | HEART\_DEVELOPMENT |  | 33 | -0.25 | -1.02 | 0.415 | 0.655 | 1.000 | 3950 | tags=39%, list=30%, signal=56% |
| 202 | NITROGEN\_COMPOUND\_CATABOLIC\_PROCESS |  | 27 | -0.27 | -1.02 | 0.407 | 0.656 | 1.000 | 1923 | tags=26%, list=15%, signal=30% |
| 203 | MAPKKK\_CASCADE\_GO\_0000165 |  | 90 | -0.20 | -1.02 | 0.421 | 0.655 | 1.000 | 2008 | tags=21%, list=15%, signal=25% |
| 204 | TRANSLATION |  | 149 | -0.18 | -1.02 | 0.453 | 0.655 | 1.000 | 2759 | tags=28%, list=21%, signal=34% |
| 205 | ANTI\_APOPTOSIS |  | 107 | -0.19 | -1.01 | 0.398 | 0.654 | 1.000 | 2194 | tags=25%, list=17%, signal=30% |
| 206 | CELLULAR\_COMPONENT\_ASSEMBLY |  | 272 | -0.16 | -1.01 | 0.389 | 0.654 | 1.000 | 3271 | tags=29%, list=25%, signal=38% |
| 207 | POSITIVE\_REGULATION\_OF\_TRANSCRIPTION\_FACTOR\_ACTIVITY |  | 17 | -0.30 | -1.01 | 0.431 | 0.658 | 1.000 | 4173 | tags=53%, list=32%, signal=78% |
| 208 | ORGANIC\_ACID\_TRANSPORT |  | 39 | -0.23 | -1.00 | 0.432 | 0.674 | 1.000 | 1014 | tags=18%, list=8%, signal=19% |
| 209 | CARBOXYLIC\_ACID\_TRANSPORT |  | 39 | -0.23 | -1.00 | 0.455 | 0.682 | 1.000 | 1014 | tags=18%, list=8%, signal=19% |
| 210 | ANATOMICAL\_STRUCTURE\_MORPHOGENESIS |  | 336 | -0.16 | -1.00 | 0.455 | 0.680 | 1.000 | 3402 | tags=29%, list=26%, signal=38% |
| 211 | NEUROGENESIS |  | 75 | -0.20 | -1.00 | 0.470 | 0.677 | 1.000 | 3675 | tags=33%, list=28%, signal=46% |
| 212 | REGULATION\_OF\_CYTOKINE\_BIOSYNTHETIC\_PROCESS |  | 31 | -0.24 | -1.00 | 0.442 | 0.684 | 1.000 | 2856 | tags=32%, list=22%, signal=41% |
| 213 | MACROMOLECULE\_BIOSYNTHETIC\_PROCESS |  | 267 | -0.16 | -0.99 | 0.479 | 0.683 | 1.000 | 3058 | tags=27%, list=23%, signal=35% |
| 214 | REGULATION\_OF\_TRANSCRIPTION |  | 498 | -0.15 | -0.99 | 0.467 | 0.685 | 1.000 | 3072 | tags=27%, list=23%, signal=33% |
| 215 | REGULATION\_OF\_JNK\_ACTIVITY |  | 18 | -0.29 | -0.99 | 0.464 | 0.693 | 1.000 | 1919 | tags=28%, list=15%, signal=33% |
| 216 | AMINO\_ACID\_AND\_DERIVATIVE\_METABOLIC\_PROCESS |  | 96 | -0.19 | -0.99 | 0.471 | 0.693 | 1.000 | 2275 | tags=24%, list=17%, signal=29% |
| 217 | AXON\_GUIDANCE |  | 18 | -0.29 | -0.98 | 0.476 | 0.705 | 1.000 | 2873 | tags=33%, list=22%, signal=43% |
| 218 | RHYTHMIC\_PROCESS |  | 23 | -0.26 | -0.98 | 0.497 | 0.703 | 1.000 | 2029 | tags=26%, list=15%, signal=31% |
| 219 | CHEMICAL\_HOMEOSTASIS |  | 136 | -0.18 | -0.98 | 0.484 | 0.701 | 1.000 | 3156 | tags=29%, list=24%, signal=37% |
| 220 | NEGATIVE\_REGULATION\_OF\_DEVELOPMENTAL\_PROCESS |  | 177 | -0.17 | -0.98 | 0.506 | 0.713 | 1.000 | 3260 | tags=31%, list=25%, signal=40% |
| 221 | CELLULAR\_PROTEIN\_COMPLEX\_ASSEMBLY |  | 28 | -0.25 | -0.97 | 0.507 | 0.720 | 1.000 | 2744 | tags=29%, list=21%, signal=36% |
| 222 | POSITIVE\_REGULATION\_OF\_DNA\_BINDING |  | 18 | -0.28 | -0.97 | 0.490 | 0.729 | 1.000 | 4541 | tags=56%, list=35%, signal=85% |
| 223 | REGULATION\_OF\_PROTEIN\_SECRETION |  | 19 | -0.28 | -0.96 | 0.502 | 0.732 | 1.000 | 4107 | tags=47%, list=31%, signal=69% |
| 224 | MACROMOLECULAR\_COMPLEX\_ASSEMBLY |  | 254 | -0.16 | -0.96 | 0.567 | 0.736 | 1.000 | 3271 | tags=29%, list=25%, signal=38% |
| 225 | CELLULAR\_HOMEOSTASIS |  | 121 | -0.17 | -0.94 | 0.585 | 0.779 | 1.000 | 3230 | tags=30%, list=25%, signal=39% |
| 226 | SKELETAL\_MUSCLE\_DEVELOPMENT |  | 28 | -0.24 | -0.94 | 0.557 | 0.792 | 1.000 | 3519 | tags=43%, list=27%, signal=58% |
| 227 | REGULATION\_OF\_G\_PROTEIN\_COUPLED\_RECEPTOR\_PROTEIN\_SIGNALING\_PATHWAY |  | 23 | -0.25 | -0.94 | 0.544 | 0.790 | 1.000 | 1081 | tags=17%, list=8%, signal=19% |
| 228 | ACTIVATION\_OF\_PROTEIN\_KINASE\_ACTIVITY |  | 23 | -0.25 | -0.94 | 0.514 | 0.791 | 1.000 | 4367 | tags=39%, list=33%, signal=59% |
| 229 | POSITIVE\_REGULATION\_OF\_TRANSCRIPTION\_FROM\_RNA\_POLYMERASE\_II\_PROMOTER |  | 60 | -0.20 | -0.94 | 0.571 | 0.789 | 1.000 | 3295 | tags=32%, list=25%, signal=42% |
| 230 | CELL\_CELL\_ADHESION |  | 72 | -0.19 | -0.94 | 0.571 | 0.786 | 1.000 | 4791 | tags=47%, list=37%, signal=74% |
| 231 | HOMEOSTATIC\_PROCESS |  | 179 | -0.16 | -0.93 | 0.629 | 0.788 | 1.000 | 3156 | tags=27%, list=24%, signal=36% |
| 232 | POSITIVE\_REGULATION\_OF\_NUCLEOBASENUCLEOSIDENUCLEOTIDE\_AND\_NUCLEIC\_ACID\_METABOLIC\_PROCESS |  | 134 | -0.17 | -0.93 | 0.604 | 0.796 | 1.000 | 2076 | tags=20%, list=16%, signal=24% |
| 233 | SECRETION\_BY\_CELL |  | 100 | -0.17 | -0.93 | 0.622 | 0.802 | 1.000 | 4470 | tags=42%, list=34%, signal=63% |
| 234 | INSULIN\_RECEPTOR\_SIGNALING\_PATHWAY |  | 16 | -0.27 | -0.92 | 0.536 | 0.801 | 1.000 | 3227 | tags=38%, list=25%, signal=50% |
| 235 | CYCLIC\_NUCLEOTIDE\_MEDIATED\_SIGNALING |  | 97 | -0.18 | -0.92 | 0.617 | 0.802 | 1.000 | 1237 | tags=12%, list=9%, signal=14% |
| 236 | EPIDERMIS\_DEVELOPMENT |  | 66 | -0.19 | -0.91 | 0.614 | 0.826 | 1.000 | 1615 | tags=20%, list=12%, signal=22% |
| 237 | RESPONSE\_TO\_NUTRIENT |  | 17 | -0.27 | -0.91 | 0.576 | 0.840 | 1.000 | 414 | tags=18%, list=3%, signal=18% |
| 238 | G\_PROTEIN\_SIGNALING\_COUPLED\_TO\_CYCLIC\_NUCLEOTIDE\_SECOND\_MESSENGER |  | 96 | -0.18 | -0.90 | 0.678 | 0.848 | 1.000 | 1237 | tags=13%, list=9%, signal=14% |
| 239 | REGULATION\_OF\_PHOSPHORYLATION |  | 42 | -0.20 | -0.90 | 0.608 | 0.847 | 1.000 | 2562 | tags=26%, list=20%, signal=32% |
| 240 | ENDOSOME\_TRANSPORT |  | 22 | -0.25 | -0.90 | 0.604 | 0.857 | 1.000 | 2239 | tags=27%, list=17%, signal=33% |
| 241 | POSITIVE\_REGULATION\_OF\_CELLULAR\_COMPONENT\_ORGANIZATION\_AND\_BIOGENESIS |  | 28 | -0.23 | -0.89 | 0.628 | 0.857 | 1.000 | 4181 | tags=43%, list=32%, signal=63% |
| 242 | REGULATION\_OF\_PROTEIN\_MODIFICATION\_PROCESS |  | 37 | -0.21 | -0.89 | 0.629 | 0.864 | 1.000 | 1513 | tags=19%, list=12%, signal=21% |
| 243 | PHOSPHOINOSITIDE\_METABOLIC\_PROCESS |  | 25 | -0.24 | -0.88 | 0.637 | 0.874 | 1.000 | 4690 | tags=56%, list=36%, signal=87% |
| 244 | REGULATION\_OF\_BINDING |  | 46 | -0.20 | -0.88 | 0.655 | 0.872 | 1.000 | 2608 | tags=28%, list=20%, signal=35% |
| 245 | AMINE\_METABOLIC\_PROCESS |  | 128 | -0.16 | -0.88 | 0.722 | 0.874 | 1.000 | 3986 | tags=35%, list=30%, signal=50% |
| 246 | ECTODERM\_DEVELOPMENT |  | 75 | -0.17 | -0.88 | 0.691 | 0.876 | 1.000 | 1615 | tags=19%, list=12%, signal=21% |
| 247 | REGULATION\_OF\_CELLULAR\_COMPONENT\_ORGANIZATION\_AND\_BIOGENESIS |  | 102 | -0.17 | -0.88 | 0.754 | 0.873 | 1.000 | 3557 | tags=31%, list=27%, signal=43% |
| 248 | REGULATION\_OF\_TRANSLATIONAL\_INITIATION |  | 25 | -0.23 | -0.88 | 0.637 | 0.874 | 1.000 | 1147 | tags=20%, list=9%, signal=22% |
| 249 | CELLULAR\_MORPHOGENESIS\_DURING\_DIFFERENTIATION |  | 38 | -0.20 | -0.88 | 0.662 | 0.871 | 1.000 | 3371 | tags=29%, list=26%, signal=39% |
| 250 | SULFUR\_METABOLIC\_PROCESS |  | 30 | -0.22 | -0.87 | 0.684 | 0.878 | 1.000 | 2877 | tags=30%, list=22%, signal=38% |
| 251 | CELL\_CELL\_SIGNALING |  | 372 | -0.13 | -0.87 | 0.878 | 0.878 | 1.000 | 4038 | tags=32%, list=31%, signal=45% |
| 252 | SPHINGOLIPID\_METABOLIC\_PROCESS |  | 23 | -0.23 | -0.86 | 0.688 | 0.908 | 1.000 | 3230 | tags=35%, list=25%, signal=46% |
| 253 | RESPONSE\_TO\_NUTRIENT\_LEVELS |  | 27 | -0.22 | -0.86 | 0.644 | 0.906 | 1.000 | 2368 | tags=26%, list=18%, signal=32% |
| 254 | VESICLE\_MEDIATED\_TRANSPORT |  | 174 | -0.14 | -0.85 | 0.857 | 0.916 | 1.000 | 2239 | tags=20%, list=17%, signal=23% |
| 255 | CENTRAL\_NERVOUS\_SYSTEM\_DEVELOPMENT |  | 105 | -0.16 | -0.84 | 0.847 | 0.934 | 1.000 | 4087 | tags=35%, list=31%, signal=51% |
| 256 | REGULATION\_OF\_TRANSLATION |  | 76 | -0.17 | -0.84 | 0.806 | 0.932 | 1.000 | 3011 | tags=28%, list=23%, signal=36% |
| 257 | REGULATION\_OF\_GROWTH |  | 48 | -0.19 | -0.84 | 0.767 | 0.933 | 1.000 | 4391 | tags=42%, list=34%, signal=62% |
| 258 | REGULATION\_OF\_MUSCLE\_CONTRACTION |  | 18 | -0.25 | -0.83 | 0.706 | 0.936 | 1.000 | 3105 | tags=39%, list=24%, signal=51% |
| 259 | POSITIVE\_REGULATION\_OF\_CASPASE\_ACTIVITY |  | 28 | -0.21 | -0.83 | 0.716 | 0.940 | 1.000 | 1909 | tags=25%, list=15%, signal=29% |
| 260 | GOLGI\_VESICLE\_TRANSPORT |  | 42 | -0.19 | -0.83 | 0.758 | 0.941 | 1.000 | 4349 | tags=43%, list=33%, signal=64% |
| 261 | PROTEIN\_LOCALIZATION |  | 184 | -0.14 | -0.82 | 0.920 | 0.945 | 1.000 | 3629 | tags=29%, list=28%, signal=40% |
| 262 | NITROGEN\_COMPOUND\_METABOLIC\_PROCESS |  | 141 | -0.15 | -0.82 | 0.877 | 0.948 | 1.000 | 2963 | tags=25%, list=23%, signal=32% |
| 263 | SECOND\_MESSENGER\_MEDIATED\_SIGNALING |  | 139 | -0.15 | -0.81 | 0.903 | 0.953 | 1.000 | 1376 | tags=12%, list=11%, signal=14% |
| 264 | EXTRACELLULAR\_STRUCTURE\_ORGANIZATION\_AND\_BIOGENESIS |  | 23 | -0.22 | -0.81 | 0.715 | 0.954 | 1.000 | 3519 | tags=39%, list=27%, signal=53% |
| 265 | T\_CELL\_PROLIFERATION |  | 17 | -0.24 | -0.81 | 0.722 | 0.960 | 1.000 | 4825 | tags=59%, list=37%, signal=93% |
| 266 | REGULATION\_OF\_TRANSCRIPTION\_FACTOR\_ACTIVITY |  | 30 | -0.20 | -0.81 | 0.785 | 0.957 | 1.000 | 4405 | tags=47%, list=34%, signal=70% |
| 267 | POSITIVE\_REGULATION\_OF\_BINDING |  | 19 | -0.23 | -0.80 | 0.753 | 0.968 | 1.000 | 4541 | tags=53%, list=35%, signal=80% |
| 268 | PROTEIN\_HOMOOLIGOMERIZATION |  | 19 | -0.23 | -0.79 | 0.740 | 0.970 | 1.000 | 1528 | tags=21%, list=12%, signal=24% |
| 269 | NEGATIVE\_REGULATION\_OF\_CELLULAR\_COMPONENT\_ORGANIZATION\_AND\_BIOGENESIS |  | 26 | -0.21 | -0.79 | 0.788 | 0.968 | 1.000 | 2014 | tags=19%, list=15%, signal=23% |
| 270 | POSITIVE\_REGULATION\_OF\_JNK\_ACTIVITY |  | 16 | -0.23 | -0.79 | 0.753 | 0.977 | 1.000 | 1919 | tags=25%, list=15%, signal=29% |
| 271 | POSITIVE\_REGULATION\_OF\_TRANSCRIPTIONDNA\_DEPENDENT |  | 105 | -0.14 | -0.78 | 0.933 | 0.985 | 1.000 | 4576 | tags=40%, list=35%, signal=61% |
| 272 | REGULATION\_OF\_DNA\_BINDING |  | 36 | -0.18 | -0.78 | 0.806 | 0.982 | 1.000 | 2608 | tags=28%, list=20%, signal=35% |
| 273 | METAL\_ION\_TRANSPORT |  | 102 | -0.15 | -0.78 | 0.920 | 0.980 | 1.000 | 4995 | tags=45%, list=38%, signal=72% |
| 274 | RESPONSE\_TO\_EXTRACELLULAR\_STIMULUS |  | 29 | -0.20 | -0.78 | 0.848 | 0.979 | 1.000 | 2368 | tags=24%, list=18%, signal=29% |
| 275 | REPRODUCTIVE\_PROCESS |  | 133 | -0.14 | -0.77 | 0.938 | 0.979 | 1.000 | 3840 | tags=33%, list=29%, signal=46% |
| 276 | AMINE\_BIOSYNTHETIC\_PROCESS |  | 15 | -0.24 | -0.77 | 0.763 | 0.976 | 1.000 | 412 | tags=13%, list=3%, signal=14% |
| 277 | REGULATION\_OF\_CELL\_MIGRATION |  | 23 | -0.21 | -0.77 | 0.801 | 0.981 | 1.000 | 5028 | tags=48%, list=38%, signal=78% |
| 278 | G\_PROTEIN\_SIGNALING\_COUPLED\_TO\_IP3\_SECOND\_MESSENGERPHOSPHOLIPASE\_C\_ACTIVATING |  | 39 | -0.18 | -0.76 | 0.869 | 0.981 | 1.000 | 2613 | tags=23%, list=20%, signal=29% |
| 279 | CARBOHYDRATE\_METABOLIC\_PROCESS |  | 152 | -0.13 | -0.76 | 0.954 | 0.989 | 1.000 | 4112 | tags=34%, list=31%, signal=48% |
| 280 | PATTERN\_SPECIFICATION\_PROCESS |  | 27 | -0.19 | -0.75 | 0.854 | 0.987 | 1.000 | 5548 | tags=63%, list=42%, signal=109% |
| 281 | G\_PROTEIN\_COUPLED\_RECEPTOR\_PROTEIN\_SIGNALING\_PATHWAY |  | 300 | -0.12 | -0.75 | 0.997 | 0.989 | 1.000 | 4212 | tags=30%, list=32%, signal=44% |
| 282 | EXCRETION |  | 35 | -0.18 | -0.73 | 0.868 | 1.000 | 1.000 | 2168 | tags=20%, list=17%, signal=24% |
| 283 | NEGATIVE\_REGULATION\_OF\_GROWTH |  | 35 | -0.18 | -0.73 | 0.883 | 1.000 | 1.000 | 4391 | tags=43%, list=34%, signal=64% |
| 284 | POSITIVE\_REGULATION\_OF\_RNA\_METABOLIC\_PROCESS |  | 107 | -0.14 | -0.73 | 0.963 | 1.000 | 1.000 | 4576 | tags=39%, list=35%, signal=60% |
| 285 | PROTEIN\_POLYMERIZATION |  | 17 | -0.21 | -0.72 | 0.856 | 1.000 | 1.000 | 318 | tags=12%, list=2%, signal=12% |
| 286 | REGULATION\_OF\_SECRETION |  | 35 | -0.18 | -0.72 | 0.882 | 1.000 | 1.000 | 1317 | tags=17%, list=10%, signal=19% |
| 287 | G\_PROTEIN\_SIGNALING\_ADENYLATE\_CYCLASE\_ACTIVATING\_PATHWAY |  | 24 | -0.19 | -0.72 | 0.860 | 1.000 | 1.000 | 1237 | tags=13%, list=9%, signal=14% |
| 288 | DI\_\_\_TRI\_VALENT\_INORGANIC\_CATION\_TRANSPORT |  | 27 | -0.18 | -0.72 | 0.893 | 1.000 | 1.000 | 1112 | tags=15%, list=8%, signal=16% |
| 289 | CATION\_TRANSPORT |  | 130 | -0.13 | -0.71 | 0.984 | 1.000 | 1.000 | 4995 | tags=44%, list=38%, signal=70% |
| 290 | NEGATIVE\_REGULATION\_OF\_CELLULAR\_PROTEIN\_METABOLIC\_PROCESS |  | 41 | -0.16 | -0.71 | 0.933 | 1.000 | 1.000 | 2901 | tags=24%, list=22%, signal=31% |
| 291 | PROTEIN\_AMINO\_ACID\_LIPIDATION |  | 21 | -0.20 | -0.71 | 0.877 | 1.000 | 1.000 | 4544 | tags=52%, list=35%, signal=80% |
| 292 | POTASSIUM\_ION\_TRANSPORT |  | 52 | -0.15 | -0.70 | 0.936 | 1.000 | 1.000 | 5372 | tags=50%, list=41%, signal=84% |
| 293 | SECRETION |  | 157 | -0.12 | -0.70 | 0.990 | 1.000 | 1.000 | 4548 | tags=38%, list=35%, signal=58% |
| 294 | SECRETORY\_PATHWAY |  | 72 | -0.14 | -0.69 | 0.962 | 1.000 | 1.000 | 4470 | tags=40%, list=34%, signal=61% |
| 295 | POSITIVE\_REGULATION\_OF\_TRANSPORT |  | 18 | -0.20 | -0.69 | 0.882 | 1.000 | 1.000 | 4970 | tags=56%, list=38%, signal=89% |
| 296 | LIPID\_HOMEOSTASIS |  | 15 | -0.21 | -0.69 | 0.872 | 1.000 | 1.000 | 718 | tags=13%, list=5%, signal=14% |
| 297 | BRAIN\_DEVELOPMENT |  | 39 | -0.16 | -0.69 | 0.931 | 0.999 | 1.000 | 4087 | tags=38%, list=31%, signal=56% |
| 298 | CARBOHYDRATE\_BIOSYNTHETIC\_PROCESS |  | 35 | -0.16 | -0.68 | 0.917 | 1.000 | 1.000 | 4226 | tags=37%, list=32%, signal=55% |
| 299 | ION\_TRANSPORT |  | 165 | -0.12 | -0.68 | 0.998 | 1.000 | 1.000 | 4995 | tags=42%, list=38%, signal=68% |
| 300 | PHOSPHOINOSITIDE\_MEDIATED\_SIGNALING |  | 42 | -0.16 | -0.67 | 0.938 | 0.998 | 1.000 | 2613 | tags=21%, list=20%, signal=27% |
| 301 | ESTABLISHMENT\_AND\_OR\_MAINTENANCE\_OF\_CELL\_POLARITY |  | 19 | -0.19 | -0.67 | 0.914 | 0.997 | 1.000 | 3271 | tags=26%, list=25%, signal=35% |
| 302 | NUCLEOTIDE\_EXCISION\_REPAIR |  | 19 | -0.19 | -0.67 | 0.922 | 0.995 | 1.000 | 2127 | tags=21%, list=16%, signal=25% |
| 303 | INORGANIC\_ANION\_TRANSPORT |  | 16 | -0.20 | -0.67 | 0.901 | 0.992 | 1.000 | 597 | tags=13%, list=5%, signal=13% |
| 304 | NEGATIVE\_REGULATION\_OF\_PROTEIN\_METABOLIC\_PROCESS |  | 44 | -0.15 | -0.66 | 0.959 | 0.992 | 1.000 | 2901 | tags=23%, list=22%, signal=29% |
| 305 | REGULATION\_OF\_CYTOKINE\_PRODUCTION |  | 21 | -0.18 | -0.65 | 0.922 | 0.995 | 1.000 | 3237 | tags=29%, list=25%, signal=38% |
| 306 | NEGATIVE\_REGULATION\_OF\_MULTICELLULAR\_ORGANISMAL\_PROCESS |  | 27 | -0.17 | -0.65 | 0.918 | 0.993 | 1.000 | 2189 | tags=22%, list=17%, signal=27% |
| 307 | CARBOHYDRATE\_CATABOLIC\_PROCESS |  | 20 | -0.18 | -0.65 | 0.928 | 0.990 | 1.000 | 4226 | tags=35%, list=32%, signal=52% |
| 308 | CELLULAR\_CARBOHYDRATE\_CATABOLIC\_PROCESS |  | 20 | -0.18 | -0.65 | 0.906 | 0.987 | 1.000 | 4226 | tags=35%, list=32%, signal=52% |
| 309 | CALCIUM\_ION\_TRANSPORT |  | 23 | -0.16 | -0.57 | 0.966 | 1.000 | 1.000 | 1112 | tags=13%, list=8%, signal=14% |
| 310 | AMINO\_SUGAR\_METABOLIC\_PROCESS |  | 15 | -0.17 | -0.57 | 0.963 | 1.000 | 1.000 | 3751 | tags=33%, list=29%, signal=47% |
| 311 | FEMALE\_GAMETE\_GENERATION |  | 15 | -0.17 | -0.56 | 0.978 | 1.000 | 1.000 | 10819 | tags=100%, list=83%, signal=576% |
| 312 | REGULATION\_OF\_CELL\_GROWTH |  | 39 | -0.13 | -0.55 | 0.993 | 1.000 | 1.000 | 4391 | tags=36%, list=34%, signal=54% |
| 313 | REGULATION\_OF\_HEART\_CONTRACTION |  | 24 | -0.14 | -0.53 | 0.996 | 1.000 | 1.000 | 11217 | tags=100%, list=86%, signal=697% |
| 314 | RESPONSE\_TO\_LIGHT\_STIMULUS |  | 40 | -0.12 | -0.53 | 0.993 | 1.000 | 1.000 | 2182 | tags=18%, list=17%, signal=21% |
| 315 | REGULATION\_OF\_ACTION\_POTENTIAL |  | 16 | -0.16 | -0.52 | 0.988 | 1.000 | 1.000 | 3616 | tags=31%, list=28%, signal=43% |
| 316 | MONOVALENT\_INORGANIC\_CATION\_TRANSPORT |  | 83 | -0.10 | -0.50 | 1.000 | 0.999 | 1.000 | 4995 | tags=41%, list=38%, signal=66% |
| 317 | PEROXISOME\_ORGANIZATION\_AND\_BIOGENESIS |  | 15 | -0.15 | -0.48 | 0.994 | 0.998 | 1.000 | 5047 | tags=47%, list=39%, signal=76% |
Table: Gene sets enriched in phenotype **na**[plain text format]****

  
