# Supplementary material for: Multiple-input multiple-output causal strategies for gene selection
Source: BMC Bioinformatics. 2011 Nov 25;12:458. doi: 10.1186/1471-2105-12-458 (PMC3323860; doi:10.1186/1471-2105-12-458)
Supplement: Additional file 2 — Archive containing the output files computed by the preranked GSEA for λ ∈ {0.1,0.2,0.3,0.4,0.5} (GSEA_MIMO_part1.zip). [file 1471-2105-12-458-S2.ZIP › mFS00_entrez_mimo.GseaPreranked.1316037675010/gsea_report_for_na_neg_1316037675010.html]

Report for na\_neg 1316037675010 [GSEA]

| GS  follow link to MSigDB | GS DETAILS | SIZE | ES | NES | NOM p-val | FDR q-val | FWER p-val | RANK AT MAX | LEADING EDGE || 1 | IMMUNE\_RESPONSE |  | 212 | -0.39 | -2.29 | 0.000 | 0.008 | 0.006 | 4560 | tags=59%, list=35%, signal=90% |
| 2 | IMMUNE\_SYSTEM\_PROCESS |  | 298 | -0.35 | -2.19 | 0.000 | 0.011 | 0.017 | 3063 | tags=41%, list=23%, signal=52% |
| 3 | DEFENSE\_RESPONSE |  | 238 | -0.34 | -2.07 | 0.000 | 0.030 | 0.066 | 3737 | tags=45%, list=29%, signal=61% |
| 4 | REGULATION\_OF\_IMMUNE\_RESPONSE |  | 28 | -0.51 | -2.07 | 0.000 | 0.023 | 0.068 | 4526 | tags=75%, list=35%, signal=114% |
| 5 | POSITIVE\_REGULATION\_OF\_IMMUNE\_RESPONSE |  | 24 | -0.55 | -2.05 | 0.000 | 0.021 | 0.079 | 4365 | tags=75%, list=33%, signal=112% |
| 6 | POSITIVE\_REGULATION\_OF\_IMMUNE\_SYSTEM\_PROCESS |  | 44 | -0.44 | -1.97 | 0.000 | 0.041 | 0.171 | 4526 | tags=66%, list=35%, signal=100% |
| 7 | POSITIVE\_REGULATION\_OF\_MULTICELLULAR\_ORGANISMAL\_PROCESS |  | 56 | -0.42 | -1.93 | 0.000 | 0.049 | 0.232 | 3451 | tags=52%, list=26%, signal=70% |
| 8 | REGULATION\_OF\_IMMUNE\_SYSTEM\_PROCESS |  | 57 | -0.40 | -1.89 | 0.000 | 0.068 | 0.344 | 4007 | tags=58%, list=31%, signal=83% |
| 9 | RESPONSE\_TO\_WOUNDING |  | 171 | -0.33 | -1.88 | 0.000 | 0.064 | 0.358 | 2997 | tags=38%, list=23%, signal=49% |
| 10 | INFLAMMATORY\_RESPONSE |  | 115 | -0.35 | -1.87 | 0.000 | 0.063 | 0.381 | 3075 | tags=41%, list=23%, signal=53% |
| 11 | ADAPTIVE\_IMMUNE\_RESPONSE\_GO\_0002460 |  | 22 | -0.50 | -1.83 | 0.014 | 0.084 | 0.505 | 4007 | tags=59%, list=31%, signal=85% |
| 12 | ADAPTIVE\_IMMUNE\_RESPONSE |  | 23 | -0.47 | -1.79 | 0.004 | 0.104 | 0.622 | 4007 | tags=57%, list=31%, signal=81% |
| 13 | CELLULAR\_DEFENSE\_RESPONSE |  | 54 | -0.39 | -1.77 | 0.000 | 0.108 | 0.669 | 4072 | tags=50%, list=31%, signal=72% |
| 14 | REGULATION\_OF\_CELL\_DIFFERENTIATION |  | 48 | -0.39 | -1.73 | 0.000 | 0.140 | 0.786 | 4529 | tags=56%, list=35%, signal=86% |
| 15 | REGULATION\_OF\_DEFENSE\_RESPONSE |  | 15 | -0.53 | -1.71 | 0.021 | 0.155 | 0.834 | 3998 | tags=67%, list=31%, signal=96% |
| 16 | HUMORAL\_IMMUNE\_RESPONSE |  | 30 | -0.43 | -1.70 | 0.011 | 0.161 | 0.857 | 3114 | tags=53%, list=24%, signal=70% |
| 17 | TRANSFORMING\_GROWTH\_FACTOR\_BETA\_RECEPTOR\_SIGNALING\_PATHWAY |  | 34 | -0.41 | -1.68 | 0.018 | 0.171 | 0.888 | 3053 | tags=44%, list=23%, signal=57% |
| 18 | REGULATION\_OF\_MULTICELLULAR\_ORGANISMAL\_PROCESS |  | 131 | -0.30 | -1.65 | 0.002 | 0.199 | 0.932 | 3592 | tags=41%, list=27%, signal=56% |
| 19 | HEMOPOIETIC\_OR\_LYMPHOID\_ORGAN\_DEVELOPMENT |  | 71 | -0.33 | -1.65 | 0.000 | 0.193 | 0.934 | 3044 | tags=39%, list=23%, signal=51% |
| 20 | IMMUNE\_EFFECTOR\_PROCESS |  | 34 | -0.40 | -1.65 | 0.009 | 0.185 | 0.936 | 2829 | tags=50%, list=22%, signal=64% |
| 21 | HEMOPOIESIS |  | 69 | -0.33 | -1.64 | 0.007 | 0.190 | 0.946 | 3044 | tags=39%, list=23%, signal=51% |
| 22 | LIPID\_CATABOLIC\_PROCESS |  | 34 | -0.39 | -1.63 | 0.015 | 0.188 | 0.951 | 4103 | tags=56%, list=31%, signal=81% |
| 23 | ENZYME\_LINKED\_RECEPTOR\_PROTEIN\_SIGNALING\_PATHWAY |  | 128 | -0.29 | -1.60 | 0.002 | 0.229 | 0.982 | 2509 | tags=29%, list=19%, signal=35% |
| 24 | RESPONSE\_TO\_EXTERNAL\_STIMULUS |  | 278 | -0.26 | -1.60 | 0.000 | 0.221 | 0.983 | 3075 | tags=33%, list=23%, signal=42% |
| 25 | IMMUNE\_SYSTEM\_DEVELOPMENT |  | 75 | -0.32 | -1.60 | 0.002 | 0.214 | 0.984 | 3044 | tags=39%, list=23%, signal=50% |
| 26 | REGULATION\_OF\_ANGIOGENESIS |  | 24 | -0.42 | -1.58 | 0.035 | 0.232 | 0.987 | 1859 | tags=42%, list=14%, signal=48% |
| 27 | ACTIN\_CYTOSKELETON\_ORGANIZATION\_AND\_BIOGENESIS |  | 90 | -0.30 | -1.56 | 0.013 | 0.263 | 0.996 | 1898 | tags=27%, list=14%, signal=31% |
| 28 | NEGATIVE\_REGULATION\_OF\_SIGNAL\_TRANSDUCTION |  | 31 | -0.39 | -1.55 | 0.039 | 0.270 | 0.999 | 4984 | tags=65%, list=38%, signal=104% |
| 29 | RECEPTOR\_MEDIATED\_ENDOCYTOSIS |  | 31 | -0.40 | -1.55 | 0.018 | 0.262 | 0.999 | 1859 | tags=32%, list=14%, signal=38% |
| 30 | LEUKOCYTE\_DIFFERENTIATION |  | 34 | -0.37 | -1.53 | 0.017 | 0.283 | 0.999 | 2962 | tags=44%, list=23%, signal=57% |
| 31 | TRANSMEMBRANE\_RECEPTOR\_PROTEIN\_SERINE\_THREONINE\_KINASE\_SIGNALING\_PATHWAY |  | 42 | -0.35 | -1.52 | 0.022 | 0.287 | 0.999 | 3246 | tags=40%, list=25%, signal=54% |
| 32 | JAK\_STAT\_CASCADE |  | 26 | -0.39 | -1.51 | 0.043 | 0.300 | 0.999 | 3063 | tags=46%, list=23%, signal=60% |
| 33 | POSITIVE\_REGULATION\_OF\_RESPONSE\_TO\_STIMULUS |  | 35 | -0.36 | -1.51 | 0.036 | 0.294 | 0.999 | 4365 | tags=60%, list=33%, signal=90% |
| 34 | LYMPHOCYTE\_ACTIVATION |  | 54 | -0.32 | -1.51 | 0.022 | 0.291 | 0.999 | 3026 | tags=43%, list=23%, signal=55% |
| 35 | WOUND\_HEALING |  | 49 | -0.33 | -1.50 | 0.022 | 0.297 | 0.999 | 3530 | tags=41%, list=27%, signal=56% |
| 36 | CELL\_ACTIVATION |  | 64 | -0.31 | -1.50 | 0.011 | 0.293 | 0.999 | 4137 | tags=52%, list=32%, signal=75% |
| 37 | T\_CELL\_ACTIVATION |  | 39 | -0.35 | -1.49 | 0.045 | 0.300 | 0.999 | 4007 | tags=51%, list=31%, signal=74% |
| 38 | POSITIVE\_REGULATION\_OF\_PHOSPHATE\_METABOLIC\_PROCESS |  | 23 | -0.40 | -1.48 | 0.044 | 0.299 | 0.999 | 1335 | tags=30%, list=10%, signal=34% |
| 39 | MESODERM\_DEVELOPMENT |  | 22 | -0.40 | -1.48 | 0.054 | 0.294 | 0.999 | 3011 | tags=41%, list=23%, signal=53% |
| 40 | POSITIVE\_REGULATION\_OF\_CELL\_DIFFERENTIATION |  | 21 | -0.41 | -1.48 | 0.060 | 0.294 | 1.000 | 4529 | tags=67%, list=35%, signal=102% |
| 41 | INNATE\_IMMUNE\_RESPONSE |  | 19 | -0.42 | -1.47 | 0.056 | 0.296 | 1.000 | 4379 | tags=68%, list=33%, signal=103% |
| 42 | AMINE\_TRANSPORT |  | 36 | -0.36 | -1.47 | 0.027 | 0.289 | 1.000 | 2023 | tags=28%, list=15%, signal=33% |
| 43 | REGULATION\_OF\_RESPONSE\_TO\_STIMULUS |  | 49 | -0.32 | -1.47 | 0.024 | 0.286 | 1.000 | 4365 | tags=59%, list=33%, signal=88% |
| 44 | CELLULAR\_LIPID\_CATABOLIC\_PROCESS |  | 31 | -0.36 | -1.47 | 0.053 | 0.285 | 1.000 | 4074 | tags=52%, list=31%, signal=75% |
| 45 | PROTEIN\_AMINO\_ACID\_PHOSPHORYLATION |  | 231 | -0.24 | -1.47 | 0.000 | 0.282 | 1.000 | 3118 | tags=32%, list=24%, signal=41% |
| 46 | LYMPHOCYTE\_DIFFERENTIATION |  | 23 | -0.40 | -1.46 | 0.054 | 0.290 | 1.000 | 3316 | tags=52%, list=25%, signal=70% |
| 47 | LEUKOCYTE\_ACTIVATION |  | 59 | -0.30 | -1.45 | 0.036 | 0.296 | 1.000 | 4007 | tags=51%, list=31%, signal=73% |
| 48 | GROWTH |  | 59 | -0.30 | -1.45 | 0.041 | 0.294 | 1.000 | 3903 | tags=44%, list=30%, signal=63% |
| 49 | TRANSMEMBRANE\_RECEPTOR\_PROTEIN\_TYROSINE\_KINASE\_SIGNALING\_PATHWAY |  | 76 | -0.29 | -1.44 | 0.039 | 0.307 | 1.000 | 2509 | tags=29%, list=19%, signal=36% |
| 50 | SMALL\_GTPASE\_MEDIATED\_SIGNAL\_TRANSDUCTION |  | 77 | -0.28 | -1.42 | 0.021 | 0.331 | 1.000 | 3202 | tags=39%, list=24%, signal=51% |
| 51 | COAGULATION |  | 41 | -0.33 | -1.42 | 0.041 | 0.327 | 1.000 | 1786 | tags=27%, list=14%, signal=31% |
| 52 | BLOOD\_COAGULATION |  | 41 | -0.33 | -1.42 | 0.070 | 0.325 | 1.000 | 1786 | tags=27%, list=14%, signal=31% |
| 53 | B\_CELL\_ACTIVATION |  | 17 | -0.41 | -1.41 | 0.084 | 0.332 | 1.000 | 3451 | tags=59%, list=26%, signal=80% |
| 54 | RESPONSE\_TO\_OTHER\_ORGANISM |  | 69 | -0.29 | -1.41 | 0.053 | 0.342 | 1.000 | 2935 | tags=38%, list=22%, signal=48% |
| 55 | CYTOKINE\_AND\_CHEMOKINE\_MEDIATED\_SIGNALING\_PATHWAY |  | 19 | -0.40 | -1.40 | 0.071 | 0.338 | 1.000 | 2148 | tags=37%, list=16%, signal=44% |
| 56 | AMINO\_ACID\_TRANSPORT |  | 25 | -0.36 | -1.40 | 0.074 | 0.341 | 1.000 | 2023 | tags=32%, list=15%, signal=38% |
| 57 | PROTEIN\_AMINO\_ACID\_N\_LINKED\_GLYCOSYLATION |  | 27 | -0.35 | -1.40 | 0.062 | 0.342 | 1.000 | 2616 | tags=37%, list=20%, signal=46% |
| 58 | FATTY\_ACID\_METABOLIC\_PROCESS |  | 56 | -0.30 | -1.39 | 0.057 | 0.349 | 1.000 | 4074 | tags=50%, list=31%, signal=72% |
| 59 | POSITIVE\_REGULATION\_OF\_PROTEIN\_AMINO\_ACID\_PHOSPHORYLATION |  | 15 | -0.42 | -1.39 | 0.092 | 0.353 | 1.000 | 1335 | tags=33%, list=10%, signal=37% |
| 60 | CELL\_SUBSTRATE\_ADHESION |  | 36 | -0.32 | -1.38 | 0.089 | 0.351 | 1.000 | 1720 | tags=31%, list=13%, signal=35% |
| 61 | REGULATION\_OF\_BLOOD\_PRESSURE |  | 22 | -0.37 | -1.38 | 0.096 | 0.348 | 1.000 | 3554 | tags=41%, list=27%, signal=56% |
| 62 | MULTI\_ORGANISM\_PROCESS |  | 137 | -0.24 | -1.38 | 0.028 | 0.350 | 1.000 | 3552 | tags=39%, list=27%, signal=53% |
| 63 | ANATOMICAL\_STRUCTURE\_FORMATION |  | 52 | -0.29 | -1.38 | 0.065 | 0.346 | 1.000 | 1544 | tags=27%, list=12%, signal=30% |
| 64 | POSITIVE\_REGULATION\_OF\_SIGNAL\_TRANSDUCTION |  | 97 | -0.26 | -1.37 | 0.030 | 0.353 | 1.000 | 3726 | tags=43%, list=28%, signal=60% |
| 65 | FEMALE\_PREGNANCY |  | 42 | -0.31 | -1.37 | 0.090 | 0.353 | 1.000 | 4424 | tags=55%, list=34%, signal=82% |
| 66 | ACTIN\_FILAMENT\_BASED\_PROCESS |  | 99 | -0.26 | -1.37 | 0.037 | 0.348 | 1.000 | 1898 | tags=24%, list=14%, signal=28% |
| 67 | HEMOSTASIS |  | 46 | -0.31 | -1.36 | 0.063 | 0.356 | 1.000 | 1786 | tags=26%, list=14%, signal=30% |
| 68 | REGULATION\_OF\_SIGNAL\_TRANSDUCTION |  | 173 | -0.23 | -1.36 | 0.029 | 0.354 | 1.000 | 3593 | tags=38%, list=27%, signal=51% |
| 69 | RESPONSE\_TO\_VIRUS |  | 45 | -0.30 | -1.35 | 0.076 | 0.368 | 1.000 | 2851 | tags=42%, list=22%, signal=54% |
| 70 | REGULATION\_OF\_BODY\_FLUID\_LEVELS |  | 55 | -0.29 | -1.34 | 0.066 | 0.378 | 1.000 | 1786 | tags=25%, list=14%, signal=29% |
| 71 | CATION\_HOMEOSTASIS |  | 94 | -0.25 | -1.34 | 0.049 | 0.389 | 1.000 | 3325 | tags=36%, list=25%, signal=48% |
| 72 | PEPTIDYL\_TYROSINE\_MODIFICATION |  | 23 | -0.36 | -1.33 | 0.102 | 0.400 | 1.000 | 2974 | tags=35%, list=23%, signal=45% |
| 73 | POSITIVE\_REGULATION\_OF\_CYTOKINE\_BIOSYNTHETIC\_PROCESS |  | 21 | -0.37 | -1.33 | 0.119 | 0.403 | 1.000 | 2950 | tags=43%, list=23%, signal=55% |
| 74 | PROTEIN\_PROCESSING |  | 41 | -0.30 | -1.32 | 0.098 | 0.408 | 1.000 | 4075 | tags=41%, list=31%, signal=60% |
| 75 | REGULATION\_OF\_ANATOMICAL\_STRUCTURE\_MORPHOGENESIS |  | 17 | -0.38 | -1.32 | 0.129 | 0.410 | 1.000 | 4679 | tags=53%, list=36%, signal=82% |
| 76 | REGULATION\_OF\_LYMPHOCYTE\_ACTIVATION |  | 31 | -0.33 | -1.31 | 0.125 | 0.412 | 1.000 | 3316 | tags=45%, list=25%, signal=60% |
| 77 | REGULATION\_OF\_PROTEIN\_AMINO\_ACID\_PHOSPHORYLATION |  | 23 | -0.36 | -1.31 | 0.127 | 0.409 | 1.000 | 2715 | tags=35%, list=21%, signal=44% |
| 78 | CELLULAR\_CATION\_HOMEOSTASIS |  | 91 | -0.26 | -1.31 | 0.070 | 0.407 | 1.000 | 4276 | tags=46%, list=33%, signal=68% |
| 79 | ANGIOGENESIS |  | 44 | -0.30 | -1.30 | 0.114 | 0.418 | 1.000 | 1859 | tags=30%, list=14%, signal=34% |
| 80 | POSITIVE\_REGULATION\_OF\_PHOSPHORYLATION |  | 21 | -0.36 | -1.30 | 0.147 | 0.427 | 1.000 | 1335 | tags=29%, list=10%, signal=32% |
| 81 | PROTEIN\_AMINO\_ACID\_DEPHOSPHORYLATION |  | 60 | -0.27 | -1.30 | 0.112 | 0.424 | 1.000 | 1551 | tags=22%, list=12%, signal=24% |
| 82 | RAS\_PROTEIN\_SIGNAL\_TRANSDUCTION |  | 55 | -0.28 | -1.30 | 0.104 | 0.419 | 1.000 | 3202 | tags=40%, list=24%, signal=53% |
| 83 | MAINTENANCE\_OF\_LOCALIZATION |  | 21 | -0.36 | -1.29 | 0.149 | 0.420 | 1.000 | 2786 | tags=38%, list=21%, signal=48% |
| 84 | DETECTION\_OF\_STIMULUS |  | 36 | -0.31 | -1.29 | 0.108 | 0.422 | 1.000 | 5466 | tags=58%, list=42%, signal=100% |
| 85 | RESPONSE\_TO\_DRUG |  | 21 | -0.36 | -1.29 | 0.153 | 0.422 | 1.000 | 2511 | tags=43%, list=19%, signal=53% |
| 86 | PROTEIN\_COMPLEX\_ASSEMBLY |  | 157 | -0.22 | -1.29 | 0.047 | 0.418 | 1.000 | 2496 | tags=27%, list=19%, signal=33% |
| 87 | MUSCLE\_DEVELOPMENT |  | 85 | -0.25 | -1.28 | 0.076 | 0.422 | 1.000 | 3503 | tags=40%, list=27%, signal=54% |
| 88 | REGULATION\_OF\_CYTOSKELETON\_ORGANIZATION\_AND\_BIOGENESIS |  | 26 | -0.33 | -1.28 | 0.133 | 0.425 | 1.000 | 2939 | tags=38%, list=22%, signal=49% |
| 89 | POSITIVE\_REGULATION\_OF\_SECRETION |  | 18 | -0.37 | -1.28 | 0.159 | 0.427 | 1.000 | 4283 | tags=61%, list=33%, signal=91% |
| 90 | GLYCOPROTEIN\_METABOLIC\_PROCESS |  | 82 | -0.25 | -1.28 | 0.078 | 0.423 | 1.000 | 3873 | tags=40%, list=30%, signal=57% |
| 91 | VASCULATURE\_DEVELOPMENT |  | 50 | -0.27 | -1.28 | 0.115 | 0.421 | 1.000 | 4276 | tags=50%, list=33%, signal=74% |
| 92 | ACTIN\_POLYMERIZATION\_AND\_OR\_DEPOLYMERIZATION |  | 20 | -0.36 | -1.27 | 0.171 | 0.431 | 1.000 | 1409 | tags=25%, list=11%, signal=28% |
| 93 | PROTEIN\_AUTOPROCESSING |  | 24 | -0.34 | -1.27 | 0.156 | 0.432 | 1.000 | 4075 | tags=46%, list=31%, signal=66% |
| 94 | CYTOKINE\_PRODUCTION |  | 61 | -0.27 | -1.26 | 0.107 | 0.442 | 1.000 | 4007 | tags=44%, list=31%, signal=63% |
| 95 | DEPHOSPHORYLATION |  | 67 | -0.25 | -1.26 | 0.117 | 0.437 | 1.000 | 1551 | tags=21%, list=12%, signal=24% |
| 96 | LIPID\_METABOLIC\_PROCESS |  | 283 | -0.20 | -1.26 | 0.036 | 0.437 | 1.000 | 3469 | tags=35%, list=26%, signal=46% |
| 97 | PROTEIN\_OLIGOMERIZATION |  | 37 | -0.31 | -1.25 | 0.128 | 0.444 | 1.000 | 2496 | tags=32%, list=19%, signal=40% |
| 98 | ICOSANOID\_METABOLIC\_PROCESS |  | 16 | -0.39 | -1.25 | 0.207 | 0.441 | 1.000 | 3662 | tags=50%, list=28%, signal=69% |
| 99 | CELL\_MATRIX\_ADHESION |  | 35 | -0.30 | -1.25 | 0.150 | 0.441 | 1.000 | 1720 | tags=29%, list=13%, signal=33% |
| 100 | PROTEIN\_AMINO\_ACID\_AUTOPHOSPHORYLATION |  | 24 | -0.34 | -1.25 | 0.152 | 0.437 | 1.000 | 4075 | tags=46%, list=31%, signal=66% |
| 101 | PHOSPHORYLATION |  | 262 | -0.20 | -1.25 | 0.050 | 0.433 | 1.000 | 3118 | tags=30%, list=24%, signal=39% |
| 102 | AMINO\_ACID\_CATABOLIC\_PROCESS |  | 23 | -0.34 | -1.25 | 0.154 | 0.432 | 1.000 | 1950 | tags=35%, list=15%, signal=41% |
| 103 | PROTEIN\_KINASE\_CASCADE |  | 239 | -0.21 | -1.25 | 0.056 | 0.431 | 1.000 | 1989 | tags=23%, list=15%, signal=27% |
| 104 | POSITIVE\_REGULATION\_OF\_LYMPHOCYTE\_ACTIVATION |  | 23 | -0.33 | -1.24 | 0.162 | 0.437 | 1.000 | 3316 | tags=43%, list=25%, signal=58% |
| 105 | MONOCARBOXYLIC\_ACID\_METABOLIC\_PROCESS |  | 77 | -0.25 | -1.24 | 0.098 | 0.437 | 1.000 | 4168 | tags=45%, list=32%, signal=66% |
| 106 | REGULATION\_OF\_T\_CELL\_ACTIVATION |  | 25 | -0.32 | -1.24 | 0.162 | 0.438 | 1.000 | 3316 | tags=44%, list=25%, signal=59% |
| 107 | ORGAN\_MORPHOGENESIS |  | 131 | -0.22 | -1.24 | 0.080 | 0.438 | 1.000 | 1544 | tags=20%, list=12%, signal=22% |
| 108 | REGULATION\_OF\_CELL\_PROLIFERATION |  | 275 | -0.20 | -1.23 | 0.044 | 0.459 | 1.000 | 2950 | tags=28%, list=23%, signal=36% |
| 109 | POSITIVE\_REGULATION\_OF\_TRANSLATION |  | 28 | -0.31 | -1.23 | 0.185 | 0.457 | 1.000 | 2950 | tags=39%, list=23%, signal=51% |
| 110 | PHOSPHOLIPID\_METABOLIC\_PROCESS |  | 63 | -0.25 | -1.22 | 0.134 | 0.457 | 1.000 | 3949 | tags=43%, list=30%, signal=61% |
| 111 | PEPTIDYL\_TYROSINE\_PHOSPHORYLATION |  | 21 | -0.33 | -1.22 | 0.199 | 0.455 | 1.000 | 2974 | tags=33%, list=23%, signal=43% |
| 112 | G\_PROTEIN\_SIGNALING\_COUPLED\_TO\_CAMP\_NUCLEOTIDE\_SECOND\_MESSENGER |  | 62 | -0.26 | -1.22 | 0.157 | 0.453 | 1.000 | 2200 | tags=21%, list=17%, signal=25% |
| 113 | CAMP\_MEDIATED\_SIGNALING |  | 63 | -0.26 | -1.22 | 0.148 | 0.449 | 1.000 | 2200 | tags=21%, list=17%, signal=25% |
| 114 | AMINE\_CATABOLIC\_PROCESS |  | 25 | -0.32 | -1.20 | 0.190 | 0.494 | 1.000 | 1950 | tags=32%, list=15%, signal=38% |
| 115 | NEGATIVE\_REGULATION\_OF\_TRANSCRIPTION |  | 166 | -0.21 | -1.20 | 0.128 | 0.494 | 1.000 | 2750 | tags=28%, list=21%, signal=35% |
| 116 | REGULATION\_OF\_MYELOID\_CELL\_DIFFERENTIATION |  | 19 | -0.34 | -1.20 | 0.227 | 0.501 | 1.000 | 4529 | tags=58%, list=35%, signal=88% |
| 117 | POSITIVE\_REGULATION\_OF\_TRANSFERASE\_ACTIVITY |  | 71 | -0.24 | -1.20 | 0.153 | 0.499 | 1.000 | 2466 | tags=27%, list=19%, signal=33% |
| 118 | REGULATION\_OF\_I\_KAPPAB\_KINASE\_NF\_KAPPAB\_CASCADE |  | 72 | -0.24 | -1.19 | 0.164 | 0.506 | 1.000 | 3851 | tags=46%, list=29%, signal=65% |
| 119 | MUSCLE\_CELL\_DIFFERENTIATION |  | 21 | -0.33 | -1.19 | 0.228 | 0.517 | 1.000 | 3503 | tags=48%, list=27%, signal=65% |
| 120 | POSITIVE\_REGULATION\_OF\_CELL\_PROLIFERATION |  | 129 | -0.21 | -1.18 | 0.156 | 0.515 | 1.000 | 2031 | tags=23%, list=16%, signal=27% |
| 121 | SODIUM\_ION\_TRANSPORT |  | 17 | -0.35 | -1.18 | 0.262 | 0.512 | 1.000 | 4731 | tags=53%, list=36%, signal=83% |
| 122 | RESPONSE\_TO\_BACTERIUM |  | 22 | -0.32 | -1.18 | 0.243 | 0.511 | 1.000 | 1859 | tags=27%, list=14%, signal=32% |
| 123 | NITROGEN\_COMPOUND\_CATABOLIC\_PROCESS |  | 27 | -0.31 | -1.17 | 0.239 | 0.529 | 1.000 | 1950 | tags=30%, list=15%, signal=35% |
| 124 | BEHAVIOR |  | 136 | -0.21 | -1.17 | 0.167 | 0.528 | 1.000 | 4526 | tags=44%, list=35%, signal=67% |
| 125 | REGULATION\_OF\_MAP\_KINASE\_ACTIVITY |  | 56 | -0.25 | -1.17 | 0.209 | 0.527 | 1.000 | 2093 | tags=29%, list=16%, signal=34% |
| 126 | REGULATION\_OF\_PROTEIN\_METABOLIC\_PROCESS |  | 150 | -0.21 | -1.17 | 0.150 | 0.524 | 1.000 | 2984 | tags=30%, list=23%, signal=38% |
| 127 | REGULATION\_OF\_ORGANELLE\_ORGANIZATION\_AND\_BIOGENESIS |  | 35 | -0.28 | -1.17 | 0.251 | 0.534 | 1.000 | 3217 | tags=37%, list=25%, signal=49% |
| 128 | ACTIVATION\_OF\_MAPK\_ACTIVITY |  | 33 | -0.29 | -1.16 | 0.234 | 0.543 | 1.000 | 2093 | tags=30%, list=16%, signal=36% |
| 129 | BONE\_REMODELING |  | 28 | -0.30 | -1.16 | 0.255 | 0.548 | 1.000 | 2306 | tags=29%, list=18%, signal=35% |
| 130 | GLYCEROPHOSPHOLIPID\_METABOLIC\_PROCESS |  | 39 | -0.27 | -1.16 | 0.258 | 0.546 | 1.000 | 3949 | tags=44%, list=30%, signal=62% |
| 131 | POSITIVE\_REGULATION\_OF\_PROTEIN\_MODIFICATION\_PROCESS |  | 24 | -0.31 | -1.15 | 0.254 | 0.557 | 1.000 | 1335 | tags=25%, list=10%, signal=28% |
| 132 | CELL\_RECOGNITION |  | 16 | -0.35 | -1.15 | 0.261 | 0.563 | 1.000 | 3998 | tags=50%, list=31%, signal=72% |
| 133 | GENERATION\_OF\_PRECURSOR\_METABOLITES\_AND\_ENERGY |  | 120 | -0.21 | -1.14 | 0.187 | 0.565 | 1.000 | 2978 | tags=30%, list=23%, signal=38% |
| 134 | POSITIVE\_REGULATION\_OF\_CELLULAR\_PROTEIN\_METABOLIC\_PROCESS |  | 61 | -0.23 | -1.14 | 0.243 | 0.572 | 1.000 | 2477 | tags=30%, list=19%, signal=36% |
| 135 | MEMBRANE\_ORGANIZATION\_AND\_BIOGENESIS |  | 124 | -0.20 | -1.14 | 0.206 | 0.571 | 1.000 | 2032 | tags=23%, list=16%, signal=27% |
| 136 | ORGANIC\_ACID\_METABOLIC\_PROCESS |  | 162 | -0.19 | -1.14 | 0.184 | 0.570 | 1.000 | 2609 | tags=27%, list=20%, signal=33% |
| 137 | POSITIVE\_REGULATION\_OF\_PROTEIN\_METABOLIC\_PROCESS |  | 63 | -0.24 | -1.14 | 0.246 | 0.567 | 1.000 | 2477 | tags=30%, list=19%, signal=37% |
| 138 | CARBOXYLIC\_ACID\_METABOLIC\_PROCESS |  | 160 | -0.20 | -1.14 | 0.191 | 0.566 | 1.000 | 2609 | tags=27%, list=20%, signal=33% |
| 139 | VITAMIN\_METABOLIC\_PROCESS |  | 15 | -0.35 | -1.13 | 0.283 | 0.567 | 1.000 | 3604 | tags=53%, list=28%, signal=74% |
| 140 | DEVELOPMENTAL\_MATURATION |  | 18 | -0.33 | -1.13 | 0.279 | 0.565 | 1.000 | 3859 | tags=44%, list=29%, signal=63% |
| 141 | SKELETAL\_DEVELOPMENT |  | 91 | -0.22 | -1.13 | 0.244 | 0.562 | 1.000 | 2637 | tags=29%, list=20%, signal=36% |
| 142 | REGULATION\_OF\_CELLULAR\_PROTEIN\_METABOLIC\_PROCESS |  | 139 | -0.20 | -1.13 | 0.207 | 0.562 | 1.000 | 2984 | tags=29%, list=23%, signal=38% |
| 143 | POST\_TRANSLATIONAL\_PROTEIN\_MODIFICATION |  | 409 | -0.17 | -1.13 | 0.109 | 0.559 | 1.000 | 3077 | tags=27%, list=24%, signal=35% |
| 144 | TISSUE\_DEVELOPMENT |  | 126 | -0.21 | -1.13 | 0.201 | 0.563 | 1.000 | 2406 | tags=25%, list=18%, signal=30% |
| 145 | STRIATED\_MUSCLE\_DEVELOPMENT |  | 36 | -0.27 | -1.13 | 0.277 | 0.566 | 1.000 | 3700 | tags=44%, list=28%, signal=62% |
| 146 | NEGATIVE\_REGULATION\_OF\_RNA\_METABOLIC\_PROCESS |  | 114 | -0.20 | -1.12 | 0.239 | 0.574 | 1.000 | 2601 | tags=27%, list=20%, signal=34% |
| 147 | POSITIVE\_REGULATION\_OF\_I\_KAPPAB\_KINASE\_NF\_KAPPAB\_CASCADE |  | 67 | -0.23 | -1.12 | 0.242 | 0.572 | 1.000 | 3851 | tags=45%, list=29%, signal=63% |
| 148 | MEMBRANE\_LIPID\_METABOLIC\_PROCESS |  | 85 | -0.22 | -1.12 | 0.249 | 0.569 | 1.000 | 3284 | tags=35%, list=25%, signal=47% |
| 149 | CYTOKINE\_BIOSYNTHETIC\_PROCESS |  | 34 | -0.27 | -1.12 | 0.277 | 0.570 | 1.000 | 2950 | tags=35%, list=23%, signal=45% |
| 150 | ACTIVATION\_OF\_NF\_KAPPAB\_TRANSCRIPTION\_FACTOR |  | 15 | -0.35 | -1.12 | 0.291 | 0.567 | 1.000 | 4328 | tags=60%, list=33%, signal=90% |
| 151 | GENERATION\_OF\_NEURONS |  | 65 | -0.23 | -1.11 | 0.251 | 0.576 | 1.000 | 3859 | tags=37%, list=29%, signal=52% |
| 152 | FATTY\_ACID\_OXIDATION |  | 17 | -0.33 | -1.11 | 0.287 | 0.579 | 1.000 | 3421 | tags=47%, list=26%, signal=64% |
| 153 | NEGATIVE\_REGULATION\_OF\_NUCLEOBASENUCLEOSIDENUCLEOTIDE\_AND\_NUCLEIC\_ACID\_METABOLIC\_PROCESS |  | 185 | -0.19 | -1.11 | 0.210 | 0.579 | 1.000 | 2750 | tags=28%, list=21%, signal=34% |
| 154 | NEGATIVE\_REGULATION\_OF\_TRANSCRIPTION\_DNA\_DEPENDENT |  | 114 | -0.20 | -1.11 | 0.251 | 0.578 | 1.000 | 2601 | tags=27%, list=20%, signal=34% |
| 155 | REGULATION\_OF\_BIOLOGICAL\_QUALITY |  | 364 | -0.17 | -1.11 | 0.172 | 0.576 | 1.000 | 3737 | tags=32%, list=29%, signal=43% |
| 156 | AMINO\_ACID\_DERIVATIVE\_METABOLIC\_PROCESS |  | 23 | -0.29 | -1.10 | 0.304 | 0.586 | 1.000 | 4074 | tags=48%, list=31%, signal=69% |
| 157 | PROTEIN\_SECRETION |  | 28 | -0.27 | -1.10 | 0.312 | 0.587 | 1.000 | 4492 | tags=50%, list=34%, signal=76% |
| 158 | GLYCOPROTEIN\_BIOSYNTHETIC\_PROCESS |  | 67 | -0.23 | -1.10 | 0.280 | 0.584 | 1.000 | 3873 | tags=39%, list=30%, signal=55% |
| 159 | NEGATIVE\_REGULATION\_OF\_CELL\_DIFFERENTIATION |  | 24 | -0.29 | -1.10 | 0.325 | 0.583 | 1.000 | 2862 | tags=29%, list=22%, signal=37% |
| 160 | TISSUE\_REMODELING |  | 29 | -0.28 | -1.10 | 0.316 | 0.579 | 1.000 | 2306 | tags=28%, list=18%, signal=33% |
| 161 | CYTOKINE\_SECRETION |  | 15 | -0.34 | -1.10 | 0.331 | 0.579 | 1.000 | 3998 | tags=53%, list=31%, signal=77% |
| 162 | REGULATION\_OF\_DEVELOPMENTAL\_PROCESS |  | 387 | -0.17 | -1.10 | 0.183 | 0.578 | 1.000 | 4286 | tags=42%, list=33%, signal=60% |
| 163 | RESPONSE\_TO\_BIOTIC\_STIMULUS |  | 103 | -0.21 | -1.10 | 0.292 | 0.575 | 1.000 | 2935 | tags=32%, list=22%, signal=41% |
| 164 | PHAGOCYTOSIS |  | 16 | -0.33 | -1.10 | 0.314 | 0.575 | 1.000 | 3484 | tags=50%, list=27%, signal=68% |
| 165 | POSITIVE\_REGULATION\_OF\_T\_CELL\_ACTIVATION |  | 20 | -0.31 | -1.09 | 0.346 | 0.575 | 1.000 | 4526 | tags=55%, list=35%, signal=84% |
| 166 | REGULATION\_OF\_MAPKKK\_CASCADE |  | 19 | -0.32 | -1.09 | 0.334 | 0.579 | 1.000 | 752 | tags=21%, list=6%, signal=22% |
| 167 | ORGANIC\_ACID\_TRANSPORT |  | 39 | -0.25 | -1.09 | 0.294 | 0.580 | 1.000 | 2023 | tags=26%, list=15%, signal=30% |
| 168 | CARBOXYLIC\_ACID\_TRANSPORT |  | 39 | -0.25 | -1.09 | 0.320 | 0.587 | 1.000 | 2023 | tags=26%, list=15%, signal=30% |
| 169 | NEGATIVE\_REGULATION\_OF\_METABOLIC\_PROCESS |  | 232 | -0.17 | -1.09 | 0.239 | 0.584 | 1.000 | 2750 | tags=26%, list=21%, signal=32% |
| 170 | ACTIN\_FILAMENT\_ORGANIZATION |  | 21 | -0.30 | -1.09 | 0.352 | 0.581 | 1.000 | 1687 | tags=29%, list=13%, signal=33% |
| 171 | NEURON\_DIFFERENTIATION |  | 58 | -0.23 | -1.08 | 0.301 | 0.579 | 1.000 | 3530 | tags=33%, list=27%, signal=45% |
| 172 | POSITIVE\_REGULATION\_OF\_TRANSCRIPTION |  | 124 | -0.20 | -1.08 | 0.272 | 0.577 | 1.000 | 3243 | tags=31%, list=25%, signal=40% |
| 173 | INSULIN\_RECEPTOR\_SIGNALING\_PATHWAY |  | 16 | -0.32 | -1.08 | 0.318 | 0.577 | 1.000 | 3233 | tags=44%, list=25%, signal=58% |
| 174 | REGULATION\_OF\_PROTEIN\_IMPORT\_INTO\_NUCLEUS |  | 15 | -0.33 | -1.08 | 0.338 | 0.588 | 1.000 | 1440 | tags=27%, list=11%, signal=30% |
| 175 | AMINO\_ACID\_METABOLIC\_PROCESS |  | 73 | -0.22 | -1.08 | 0.313 | 0.587 | 1.000 | 2348 | tags=27%, list=18%, signal=33% |
| 176 | CELLULAR\_LIPID\_METABOLIC\_PROCESS |  | 220 | -0.18 | -1.07 | 0.269 | 0.592 | 1.000 | 3975 | tags=38%, list=30%, signal=54% |
| 177 | POSITIVE\_REGULATION\_OF\_MAP\_KINASE\_ACTIVITY |  | 39 | -0.26 | -1.07 | 0.337 | 0.593 | 1.000 | 2093 | tags=28%, list=16%, signal=33% |
| 178 | DETECTION\_OF\_EXTERNAL\_STIMULUS |  | 18 | -0.32 | -1.07 | 0.362 | 0.595 | 1.000 | 8958 | tags=100%, list=68%, signal=316% |
| 179 | NEGATIVE\_REGULATION\_OF\_TRANSCRIPTION\_FROM\_RNA\_POLYMERASE\_II\_PROMOTER |  | 76 | -0.21 | -1.07 | 0.324 | 0.593 | 1.000 | 2582 | tags=28%, list=20%, signal=34% |
| 180 | I\_KAPPAB\_KINASE\_NF\_KAPPAB\_CASCADE |  | 88 | -0.21 | -1.07 | 0.312 | 0.593 | 1.000 | 3726 | tags=41%, list=28%, signal=57% |
| 181 | CELL\_MIGRATION |  | 82 | -0.21 | -1.06 | 0.345 | 0.599 | 1.000 | 2715 | tags=26%, list=21%, signal=32% |
| 182 | ION\_HOMEOSTASIS |  | 112 | -0.20 | -1.06 | 0.317 | 0.597 | 1.000 | 3902 | tags=38%, list=30%, signal=54% |
| 183 | MYELOID\_CELL\_DIFFERENTIATION |  | 35 | -0.25 | -1.06 | 0.372 | 0.612 | 1.000 | 809 | tags=17%, list=6%, signal=18% |
| 184 | CYTOKINE\_METABOLIC\_PROCESS |  | 35 | -0.26 | -1.06 | 0.371 | 0.609 | 1.000 | 2950 | tags=34%, list=23%, signal=44% |
| 185 | CELL\_MATURATION |  | 16 | -0.32 | -1.05 | 0.386 | 0.621 | 1.000 | 3290 | tags=38%, list=25%, signal=50% |
| 186 | NEGATIVE\_REGULATION\_OF\_CELLULAR\_METABOLIC\_PROCESS |  | 229 | -0.17 | -1.04 | 0.315 | 0.637 | 1.000 | 2750 | tags=26%, list=21%, signal=32% |
| 187 | POSITIVE\_REGULATION\_OF\_DEVELOPMENTAL\_PROCESS |  | 197 | -0.18 | -1.04 | 0.369 | 0.634 | 1.000 | 4253 | tags=44%, list=32%, signal=64% |
| 188 | ELECTRON\_TRANSPORT\_GO\_0006118 |  | 50 | -0.23 | -1.04 | 0.380 | 0.640 | 1.000 | 2270 | tags=26%, list=17%, signal=31% |
| 189 | MACROMOLECULE\_BIOSYNTHETIC\_PROCESS |  | 267 | -0.17 | -1.04 | 0.372 | 0.639 | 1.000 | 3686 | tags=34%, list=28%, signal=46% |
| 190 | NEGATIVE\_REGULATION\_OF\_CELL\_PROLIFERATION |  | 145 | -0.18 | -1.04 | 0.367 | 0.639 | 1.000 | 3026 | tags=28%, list=23%, signal=36% |
| 191 | TRANSLATION |  | 149 | -0.18 | -1.04 | 0.387 | 0.637 | 1.000 | 2950 | tags=30%, list=23%, signal=38% |
| 192 | DETECTION\_OF\_STIMULUS\_INVOLVED\_IN\_SENSORY\_PERCEPTION |  | 15 | -0.32 | -1.04 | 0.418 | 0.642 | 1.000 | 8958 | tags=100%, list=68%, signal=316% |
| 193 | MYOBLAST\_DIFFERENTIATION |  | 16 | -0.31 | -1.03 | 0.406 | 0.654 | 1.000 | 3503 | tags=50%, list=27%, signal=68% |
| 194 | HORMONE\_METABOLIC\_PROCESS |  | 29 | -0.26 | -1.03 | 0.386 | 0.653 | 1.000 | 3983 | tags=48%, list=30%, signal=69% |
| 195 | POSITIVE\_REGULATION\_OF\_CATALYTIC\_ACTIVITY |  | 139 | -0.18 | -1.03 | 0.392 | 0.652 | 1.000 | 4283 | tags=38%, list=33%, signal=56% |
| 196 | POSITIVE\_REGULATION\_OF\_CELLULAR\_METABOLIC\_PROCESS |  | 196 | -0.18 | -1.03 | 0.367 | 0.651 | 1.000 | 3243 | tags=30%, list=25%, signal=39% |
| 197 | NEURON\_DEVELOPMENT |  | 49 | -0.22 | -1.03 | 0.403 | 0.653 | 1.000 | 4376 | tags=41%, list=33%, signal=61% |
| 198 | CELL\_PROLIFERATION\_GO\_0008283 |  | 466 | -0.15 | -1.02 | 0.394 | 0.660 | 1.000 | 2749 | tags=24%, list=21%, signal=30% |
| 199 | DEFENSE\_RESPONSE\_TO\_BACTERIUM |  | 16 | -0.31 | -1.02 | 0.431 | 0.661 | 1.000 | 1859 | tags=25%, list=14%, signal=29% |
| 200 | POSITIVE\_REGULATION\_OF\_METABOLIC\_PROCESS |  | 201 | -0.17 | -1.02 | 0.409 | 0.663 | 1.000 | 3243 | tags=30%, list=25%, signal=39% |
| 201 | AMINO\_ACID\_AND\_DERIVATIVE\_METABOLIC\_PROCESS |  | 96 | -0.19 | -1.01 | 0.407 | 0.669 | 1.000 | 2363 | tags=25%, list=18%, signal=30% |
| 202 | POSITIVE\_REGULATION\_OF\_TRANSCRIPTION\_FROM\_RNA\_POLYMERASE\_II\_PROMOTER |  | 60 | -0.21 | -1.01 | 0.431 | 0.672 | 1.000 | 3178 | tags=32%, list=24%, signal=42% |
| 203 | AXON\_GUIDANCE |  | 18 | -0.30 | -1.01 | 0.424 | 0.682 | 1.000 | 2704 | tags=33%, list=21%, signal=42% |
| 204 | REGULATION\_OF\_JNK\_ACTIVITY |  | 18 | -0.29 | -1.00 | 0.443 | 0.686 | 1.000 | 2093 | tags=28%, list=16%, signal=33% |
| 205 | HEART\_DEVELOPMENT |  | 33 | -0.25 | -1.00 | 0.445 | 0.690 | 1.000 | 4230 | tags=39%, list=32%, signal=58% |
| 206 | REGULATION\_OF\_TRANSCRIPTION |  | 498 | -0.15 | -1.00 | 0.438 | 0.692 | 1.000 | 3437 | tags=30%, list=26%, signal=39% |
| 207 | NERVOUS\_SYSTEM\_DEVELOPMENT |  | 328 | -0.16 | -1.00 | 0.461 | 0.692 | 1.000 | 3919 | tags=32%, list=30%, signal=45% |
| 208 | CELL\_CELL\_ADHESION |  | 72 | -0.20 | -1.00 | 0.455 | 0.691 | 1.000 | 4328 | tags=43%, list=33%, signal=64% |
| 209 | ANTI\_APOPTOSIS |  | 107 | -0.18 | -1.00 | 0.444 | 0.695 | 1.000 | 3199 | tags=34%, list=24%, signal=44% |
| 210 | LOCOMOTORY\_BEHAVIOR |  | 84 | -0.20 | -0.99 | 0.446 | 0.696 | 1.000 | 2834 | tags=29%, list=22%, signal=36% |
| 211 | AXONOGENESIS |  | 33 | -0.24 | -0.99 | 0.475 | 0.693 | 1.000 | 4376 | tags=42%, list=33%, signal=64% |
| 212 | RHYTHMIC\_PROCESS |  | 23 | -0.26 | -0.99 | 0.465 | 0.690 | 1.000 | 2637 | tags=30%, list=20%, signal=38% |
| 213 | ANATOMICAL\_STRUCTURE\_MORPHOGENESIS |  | 336 | -0.16 | -0.99 | 0.468 | 0.690 | 1.000 | 2800 | tags=24%, list=21%, signal=30% |
| 214 | REGULATION\_OF\_PROTEIN\_SECRETION |  | 19 | -0.29 | -0.99 | 0.468 | 0.687 | 1.000 | 3998 | tags=47%, list=31%, signal=68% |
| 215 | POSITIVE\_REGULATION\_OF\_NUCLEOBASENUCLEOSIDENUCLEOTIDE\_AND\_NUCLEIC\_ACID\_METABOLIC\_PROCESS |  | 134 | -0.18 | -0.99 | 0.479 | 0.699 | 1.000 | 3243 | tags=30%, list=25%, signal=39% |
| 216 | NEGATIVE\_REGULATION\_OF\_DEVELOPMENTAL\_PROCESS |  | 177 | -0.17 | -0.99 | 0.489 | 0.696 | 1.000 | 3199 | tags=31%, list=24%, signal=40% |
| 217 | MAPKKK\_CASCADE\_GO\_0000165 |  | 90 | -0.19 | -0.99 | 0.487 | 0.696 | 1.000 | 2234 | tags=22%, list=17%, signal=27% |
| 218 | EPIDERMIS\_DEVELOPMENT |  | 66 | -0.20 | -0.99 | 0.475 | 0.695 | 1.000 | 1473 | tags=20%, list=11%, signal=22% |
| 219 | POSITIVE\_REGULATION\_OF\_DNA\_BINDING |  | 18 | -0.28 | -0.98 | 0.476 | 0.713 | 1.000 | 4339 | tags=56%, list=33%, signal=83% |
| 220 | PEPTIDYL\_AMINO\_ACID\_MODIFICATION |  | 47 | -0.22 | -0.98 | 0.481 | 0.710 | 1.000 | 2974 | tags=30%, list=23%, signal=38% |
| 221 | REGULATION\_OF\_MUSCLE\_CONTRACTION |  | 18 | -0.29 | -0.97 | 0.459 | 0.714 | 1.000 | 2810 | tags=39%, list=21%, signal=49% |
| 222 | REGULATION\_OF\_CYTOKINE\_BIOSYNTHETIC\_PROCESS |  | 31 | -0.24 | -0.97 | 0.483 | 0.727 | 1.000 | 2950 | tags=32%, list=23%, signal=42% |
| 223 | NEURITE\_DEVELOPMENT |  | 41 | -0.22 | -0.96 | 0.497 | 0.733 | 1.000 | 3530 | tags=32%, list=27%, signal=43% |
| 224 | CELLULAR\_COMPONENT\_ASSEMBLY |  | 272 | -0.16 | -0.96 | 0.571 | 0.752 | 1.000 | 3022 | tags=27%, list=23%, signal=34% |
| 225 | RESPONSE\_TO\_OXIDATIVE\_STRESS |  | 38 | -0.22 | -0.96 | 0.527 | 0.753 | 1.000 | 1402 | tags=24%, list=11%, signal=26% |
| 226 | CYCLIC\_NUCLEOTIDE\_MEDIATED\_SIGNALING |  | 97 | -0.18 | -0.95 | 0.560 | 0.757 | 1.000 | 1340 | tags=12%, list=10%, signal=14% |
| 227 | PROTEIN\_HOMOOLIGOMERIZATION |  | 19 | -0.28 | -0.95 | 0.497 | 0.758 | 1.000 | 1890 | tags=26%, list=14%, signal=31% |
| 228 | RESPONSE\_TO\_CHEMICAL\_STIMULUS |  | 271 | -0.15 | -0.95 | 0.573 | 0.766 | 1.000 | 1916 | tags=18%, list=15%, signal=21% |
| 229 | REGULATION\_OF\_G\_PROTEIN\_COUPLED\_RECEPTOR\_PROTEIN\_SIGNALING\_PATHWAY |  | 23 | -0.26 | -0.94 | 0.530 | 0.770 | 1.000 | 1055 | tags=17%, list=8%, signal=19% |
| 230 | NEUROGENESIS |  | 75 | -0.19 | -0.94 | 0.575 | 0.769 | 1.000 | 3530 | tags=32%, list=27%, signal=44% |
| 231 | REGULATION\_OF\_TRANSLATIONAL\_INITIATION |  | 25 | -0.25 | -0.94 | 0.527 | 0.766 | 1.000 | 2578 | tags=32%, list=20%, signal=40% |
| 232 | CELLULAR\_HOMEOSTASIS |  | 121 | -0.17 | -0.94 | 0.604 | 0.781 | 1.000 | 3644 | tags=34%, list=28%, signal=47% |
| 233 | AMINE\_METABOLIC\_PROCESS |  | 128 | -0.17 | -0.93 | 0.589 | 0.790 | 1.000 | 4100 | tags=38%, list=31%, signal=55% |
| 234 | G\_PROTEIN\_SIGNALING\_COUPLED\_TO\_CYCLIC\_NUCLEOTIDE\_SECOND\_MESSENGER |  | 96 | -0.18 | -0.93 | 0.608 | 0.790 | 1.000 | 1340 | tags=13%, list=10%, signal=14% |
| 235 | REGULATION\_OF\_GROWTH |  | 48 | -0.21 | -0.93 | 0.548 | 0.787 | 1.000 | 3903 | tags=40%, list=30%, signal=56% |
| 236 | POSITIVE\_REGULATION\_OF\_TRANSCRIPTION\_FACTOR\_ACTIVITY |  | 17 | -0.27 | -0.92 | 0.546 | 0.805 | 1.000 | 4328 | tags=53%, list=33%, signal=79% |
| 237 | SECRETION\_BY\_CELL |  | 100 | -0.17 | -0.92 | 0.633 | 0.803 | 1.000 | 4610 | tags=44%, list=35%, signal=67% |
| 238 | REGULATION\_OF\_TRANSLATION |  | 76 | -0.18 | -0.92 | 0.623 | 0.801 | 1.000 | 2950 | tags=29%, list=23%, signal=37% |
| 239 | MACROMOLECULAR\_COMPLEX\_ASSEMBLY |  | 254 | -0.15 | -0.92 | 0.704 | 0.798 | 1.000 | 3022 | tags=26%, list=23%, signal=34% |
| 240 | CHEMICAL\_HOMEOSTASIS |  | 136 | -0.16 | -0.91 | 0.639 | 0.810 | 1.000 | 4283 | tags=39%, list=33%, signal=57% |
| 241 | SKELETAL\_MUSCLE\_DEVELOPMENT |  | 28 | -0.23 | -0.91 | 0.621 | 0.816 | 1.000 | 3700 | tags=43%, list=28%, signal=60% |
| 242 | HOMEOSTATIC\_PROCESS |  | 179 | -0.16 | -0.91 | 0.693 | 0.814 | 1.000 | 3737 | tags=32%, list=29%, signal=45% |
| 243 | ECTODERM\_DEVELOPMENT |  | 75 | -0.18 | -0.91 | 0.629 | 0.817 | 1.000 | 1595 | tags=19%, list=12%, signal=21% |
| 244 | RESPONSE\_TO\_NUTRIENT |  | 17 | -0.27 | -0.90 | 0.582 | 0.834 | 1.000 | 419 | tags=18%, list=3%, signal=18% |
| 245 | PHOSPHOINOSITIDE\_METABOLIC\_PROCESS |  | 25 | -0.24 | -0.90 | 0.602 | 0.832 | 1.000 | 3462 | tags=40%, list=26%, signal=54% |
| 246 | NEGATIVE\_REGULATION\_OF\_CELLULAR\_COMPONENT\_ORGANIZATION\_AND\_BIOGENESIS |  | 26 | -0.23 | -0.89 | 0.625 | 0.853 | 1.000 | 1687 | tags=19%, list=13%, signal=22% |
| 247 | REGULATION\_OF\_CELLULAR\_COMPONENT\_ORGANIZATION\_AND\_BIOGENESIS |  | 102 | -0.17 | -0.89 | 0.728 | 0.851 | 1.000 | 3565 | tags=32%, list=27%, signal=44% |
| 248 | SECOND\_MESSENGER\_MEDIATED\_SIGNALING |  | 139 | -0.16 | -0.88 | 0.764 | 0.857 | 1.000 | 1340 | tags=12%, list=10%, signal=13% |
| 249 | SPHINGOLIPID\_METABOLIC\_PROCESS |  | 23 | -0.24 | -0.88 | 0.639 | 0.865 | 1.000 | 3251 | tags=35%, list=25%, signal=46% |
| 250 | NEGATIVE\_REGULATION\_OF\_GROWTH |  | 35 | -0.21 | -0.88 | 0.671 | 0.874 | 1.000 | 4716 | tags=51%, list=36%, signal=80% |
| 251 | CELLULAR\_PROTEIN\_COMPLEX\_ASSEMBLY |  | 28 | -0.22 | -0.87 | 0.653 | 0.875 | 1.000 | 2841 | tags=29%, list=22%, signal=36% |
| 252 | ENDOSOME\_TRANSPORT |  | 22 | -0.24 | -0.87 | 0.648 | 0.888 | 1.000 | 2372 | tags=27%, list=18%, signal=33% |
| 253 | VESICLE\_MEDIATED\_TRANSPORT |  | 174 | -0.15 | -0.86 | 0.828 | 0.889 | 1.000 | 1907 | tags=18%, list=15%, signal=21% |
| 254 | CELL\_CELL\_SIGNALING |  | 372 | -0.13 | -0.86 | 0.892 | 0.888 | 1.000 | 4102 | tags=32%, list=31%, signal=45% |
| 255 | REGULATION\_OF\_CELL\_MIGRATION |  | 23 | -0.23 | -0.85 | 0.682 | 0.922 | 1.000 | 4276 | tags=39%, list=33%, signal=58% |
| 256 | EXCRETION |  | 35 | -0.20 | -0.84 | 0.719 | 0.922 | 1.000 | 2024 | tags=20%, list=15%, signal=24% |
| 257 | G\_PROTEIN\_SIGNALING\_COUPLED\_TO\_IP3\_SECOND\_MESSENGERPHOSPHOLIPASE\_C\_ACTIVATING |  | 39 | -0.20 | -0.83 | 0.739 | 0.938 | 1.000 | 3571 | tags=31%, list=27%, signal=42% |
| 258 | EXTRACELLULAR\_STRUCTURE\_ORGANIZATION\_AND\_BIOGENESIS |  | 23 | -0.22 | -0.83 | 0.692 | 0.938 | 1.000 | 2054 | tags=26%, list=16%, signal=31% |
| 259 | POSITIVE\_REGULATION\_OF\_CELLULAR\_COMPONENT\_ORGANIZATION\_AND\_BIOGENESIS |  | 28 | -0.21 | -0.83 | 0.719 | 0.938 | 1.000 | 3444 | tags=36%, list=26%, signal=48% |
| 260 | NITROGEN\_COMPOUND\_METABOLIC\_PROCESS |  | 141 | -0.15 | -0.83 | 0.870 | 0.939 | 1.000 | 4100 | tags=36%, list=31%, signal=52% |
| 261 | ESTABLISHMENT\_AND\_OR\_MAINTENANCE\_OF\_CELL\_POLARITY |  | 19 | -0.24 | -0.82 | 0.718 | 0.950 | 1.000 | 3496 | tags=32%, list=27%, signal=43% |
| 262 | POSITIVE\_REGULATION\_OF\_BINDING |  | 19 | -0.24 | -0.82 | 0.704 | 0.955 | 1.000 | 4339 | tags=53%, list=33%, signal=79% |
| 263 | REGULATION\_OF\_PROTEIN\_MODIFICATION\_PROCESS |  | 37 | -0.19 | -0.81 | 0.770 | 0.969 | 1.000 | 1614 | tags=19%, list=12%, signal=22% |
| 264 | REGULATION\_OF\_BINDING |  | 46 | -0.18 | -0.81 | 0.769 | 0.968 | 1.000 | 1916 | tags=22%, list=15%, signal=25% |
| 265 | RESPONSE\_TO\_NUTRIENT\_LEVELS |  | 27 | -0.20 | -0.80 | 0.777 | 0.972 | 1.000 | 2971 | tags=30%, list=23%, signal=38% |
| 266 | POSITIVE\_REGULATION\_OF\_JNK\_ACTIVITY |  | 16 | -0.24 | -0.80 | 0.733 | 0.975 | 1.000 | 2093 | tags=25%, list=16%, signal=30% |
| 267 | CENTRAL\_NERVOUS\_SYSTEM\_DEVELOPMENT |  | 105 | -0.15 | -0.80 | 0.905 | 0.974 | 1.000 | 3696 | tags=30%, list=28%, signal=42% |
| 268 | PROTEIN\_LOCALIZATION |  | 184 | -0.14 | -0.79 | 0.947 | 0.975 | 1.000 | 3599 | tags=29%, list=27%, signal=40% |
| 269 | CELLULAR\_MORPHOGENESIS\_DURING\_DIFFERENTIATION |  | 38 | -0.18 | -0.79 | 0.808 | 0.977 | 1.000 | 3530 | tags=29%, list=27%, signal=40% |
| 270 | REPRODUCTIVE\_PROCESS |  | 133 | -0.14 | -0.79 | 0.925 | 0.974 | 1.000 | 3685 | tags=31%, list=28%, signal=42% |
| 271 | PATTERN\_SPECIFICATION\_PROCESS |  | 27 | -0.20 | -0.78 | 0.807 | 0.978 | 1.000 | 5202 | tags=52%, list=40%, signal=86% |
| 272 | G\_PROTEIN\_COUPLED\_RECEPTOR\_PROTEIN\_SIGNALING\_PATHWAY |  | 300 | -0.12 | -0.78 | 0.990 | 0.978 | 1.000 | 4260 | tags=30%, list=33%, signal=43% |
| 273 | SULFUR\_METABOLIC\_PROCESS |  | 30 | -0.20 | -0.78 | 0.793 | 0.977 | 1.000 | 3314 | tags=33%, list=25%, signal=45% |
| 274 | ACTIVATION\_OF\_PROTEIN\_KINASE\_ACTIVITY |  | 23 | -0.21 | -0.77 | 0.765 | 0.986 | 1.000 | 4679 | tags=39%, list=36%, signal=61% |
| 275 | NEGATIVE\_REGULATION\_OF\_CELLULAR\_PROTEIN\_METABOLIC\_PROCESS |  | 41 | -0.17 | -0.77 | 0.855 | 0.984 | 1.000 | 2984 | tags=27%, list=23%, signal=35% |
| 276 | POSITIVE\_REGULATION\_OF\_TRANSCRIPTIONDNA\_DEPENDENT |  | 105 | -0.14 | -0.77 | 0.942 | 0.988 | 1.000 | 3243 | tags=27%, list=25%, signal=35% |
| 277 | AMINE\_BIOSYNTHETIC\_PROCESS |  | 15 | -0.23 | -0.76 | 0.778 | 0.999 | 1.000 | 554 | tags=13%, list=4%, signal=14% |
| 278 | TRANSLATIONAL\_INITIATION |  | 33 | -0.19 | -0.76 | 0.846 | 0.997 | 1.000 | 2578 | tags=27%, list=20%, signal=34% |
| 279 | T\_CELL\_PROLIFERATION |  | 17 | -0.22 | -0.75 | 0.803 | 0.998 | 1.000 | 4815 | tags=59%, list=37%, signal=93% |
| 280 | LIPID\_HOMEOSTASIS |  | 15 | -0.23 | -0.74 | 0.802 | 1.000 | 1.000 | 524 | tags=13%, list=4%, signal=14% |
| 281 | REGULATION\_OF\_DNA\_BINDING |  | 36 | -0.17 | -0.74 | 0.875 | 1.000 | 1.000 | 3030 | tags=31%, list=23%, signal=40% |
| 282 | POSITIVE\_REGULATION\_OF\_CASPASE\_ACTIVITY |  | 28 | -0.19 | -0.74 | 0.860 | 1.000 | 1.000 | 2006 | tags=25%, list=15%, signal=29% |
| 283 | DI\_\_\_TRI\_VALENT\_INORGANIC\_CATION\_TRANSPORT |  | 27 | -0.19 | -0.74 | 0.871 | 1.000 | 1.000 | 1117 | tags=15%, list=9%, signal=16% |
| 284 | POSITIVE\_REGULATION\_OF\_RNA\_METABOLIC\_PROCESS |  | 107 | -0.14 | -0.73 | 0.965 | 1.000 | 1.000 | 3243 | tags=26%, list=25%, signal=35% |
| 285 | NEGATIVE\_REGULATION\_OF\_PROTEIN\_METABOLIC\_PROCESS |  | 44 | -0.16 | -0.73 | 0.919 | 1.000 | 1.000 | 2984 | tags=25%, list=23%, signal=32% |
| 286 | CARBOHYDRATE\_METABOLIC\_PROCESS |  | 152 | -0.13 | -0.73 | 0.986 | 1.000 | 1.000 | 4183 | tags=34%, list=32%, signal=50% |
| 287 | GOLGI\_VESICLE\_TRANSPORT |  | 42 | -0.16 | -0.72 | 0.891 | 1.000 | 1.000 | 5000 | tags=50%, list=38%, signal=81% |
| 288 | REGULATION\_OF\_TRANSCRIPTION\_FACTOR\_ACTIVITY |  | 30 | -0.18 | -0.72 | 0.881 | 1.000 | 1.000 | 3030 | tags=30%, list=23%, signal=39% |
| 289 | RESPONSE\_TO\_EXTRACELLULAR\_STIMULUS |  | 29 | -0.18 | -0.72 | 0.905 | 1.000 | 1.000 | 2971 | tags=28%, list=23%, signal=36% |
| 290 | PROTEIN\_POLYMERIZATION |  | 17 | -0.21 | -0.71 | 0.860 | 1.000 | 1.000 | 197 | tags=12%, list=2%, signal=12% |
| 291 | REGULATION\_OF\_SECRETION |  | 35 | -0.17 | -0.71 | 0.893 | 1.000 | 1.000 | 1315 | tags=17%, list=10%, signal=19% |
| 292 | PHOSPHOINOSITIDE\_MEDIATED\_SIGNALING |  | 42 | -0.16 | -0.71 | 0.894 | 0.998 | 1.000 | 3571 | tags=29%, list=27%, signal=39% |
| 293 | SECRETION |  | 157 | -0.12 | -0.70 | 0.993 | 1.000 | 1.000 | 4545 | tags=38%, list=35%, signal=57% |
| 294 | PROTEIN\_AMINO\_ACID\_LIPIDATION |  | 21 | -0.19 | -0.70 | 0.883 | 1.000 | 1.000 | 4663 | tags=52%, list=36%, signal=81% |
| 295 | REGULATION\_OF\_CELL\_GROWTH |  | 39 | -0.16 | -0.70 | 0.904 | 1.000 | 1.000 | 4716 | tags=44%, list=36%, signal=68% |
| 296 | G\_PROTEIN\_SIGNALING\_ADENYLATE\_CYCLASE\_ACTIVATING\_PATHWAY |  | 24 | -0.18 | -0.70 | 0.883 | 0.998 | 1.000 | 1340 | tags=13%, list=10%, signal=14% |
| 297 | METAL\_ION\_TRANSPORT |  | 102 | -0.13 | -0.69 | 0.988 | 0.995 | 1.000 | 5101 | tags=41%, list=39%, signal=67% |
| 298 | INORGANIC\_ANION\_TRANSPORT |  | 16 | -0.21 | -0.69 | 0.863 | 0.994 | 1.000 | 617 | tags=13%, list=5%, signal=13% |
| 299 | SECRETORY\_PATHWAY |  | 72 | -0.14 | -0.68 | 0.975 | 0.998 | 1.000 | 5000 | tags=47%, list=38%, signal=76% |
| 300 | NUCLEOTIDE\_EXCISION\_REPAIR |  | 19 | -0.19 | -0.67 | 0.920 | 1.000 | 1.000 | 2006 | tags=21%, list=15%, signal=25% |
| 301 | RHO\_PROTEIN\_SIGNAL\_TRANSDUCTION |  | 30 | -0.17 | -0.67 | 0.917 | 1.000 | 1.000 | 3673 | tags=33%, list=28%, signal=46% |
| 302 | CATION\_TRANSPORT |  | 130 | -0.12 | -0.66 | 0.995 | 1.000 | 1.000 | 4204 | tags=31%, list=32%, signal=45% |
| 303 | ION\_TRANSPORT |  | 165 | -0.11 | -0.65 | 1.000 | 1.000 | 1.000 | 4129 | tags=30%, list=32%, signal=43% |
| 304 | CARBOHYDRATE\_BIOSYNTHETIC\_PROCESS |  | 35 | -0.15 | -0.64 | 0.956 | 1.000 | 1.000 | 5343 | tags=54%, list=41%, signal=91% |
| 305 | POSITIVE\_REGULATION\_OF\_TRANSPORT |  | 18 | -0.18 | -0.63 | 0.933 | 1.000 | 1.000 | 5314 | tags=61%, list=41%, signal=103% |
| 306 | NEGATIVE\_REGULATION\_OF\_MULTICELLULAR\_ORGANISMAL\_PROCESS |  | 27 | -0.16 | -0.63 | 0.935 | 1.000 | 1.000 | 4283 | tags=41%, list=33%, signal=60% |
| 307 | REGULATION\_OF\_CYTOKINE\_PRODUCTION |  | 21 | -0.17 | -0.61 | 0.944 | 1.000 | 1.000 | 2466 | tags=24%, list=19%, signal=29% |
| 308 | POTASSIUM\_ION\_TRANSPORT |  | 52 | -0.13 | -0.60 | 0.992 | 1.000 | 1.000 | 5761 | tags=52%, list=44%, signal=92% |
| 309 | REGULATION\_OF\_HEART\_CONTRACTION |  | 24 | -0.16 | -0.59 | 0.977 | 1.000 | 1.000 | 11013 | tags=100%, list=84%, signal=629% |
| 310 | CALCIUM\_ION\_TRANSPORT |  | 23 | -0.16 | -0.59 | 0.954 | 1.000 | 1.000 | 1117 | tags=13%, list=9%, signal=14% |
| 311 | PEROXISOME\_ORGANIZATION\_AND\_BIOGENESIS |  | 15 | -0.19 | -0.59 | 0.955 | 1.000 | 1.000 | 4645 | tags=47%, list=35%, signal=72% |
| 312 | FEMALE\_GAMETE\_GENERATION |  | 15 | -0.18 | -0.58 | 0.963 | 1.000 | 1.000 | 10707 | tags=100%, list=82%, signal=548% |
| 313 | AMINO\_SUGAR\_METABOLIC\_PROCESS |  | 15 | -0.17 | -0.55 | 0.983 | 1.000 | 1.000 | 4707 | tags=47%, list=36%, signal=73% |
| 314 | REGULATION\_OF\_ACTION\_POTENTIAL |  | 16 | -0.16 | -0.53 | 0.986 | 1.000 | 1.000 | 3592 | tags=31%, list=27%, signal=43% |
| 315 | RESPONSE\_TO\_LIGHT\_STIMULUS |  | 40 | -0.12 | -0.51 | 0.993 | 1.000 | 1.000 | 2723 | tags=20%, list=21%, signal=25% |
| 316 | CARBOHYDRATE\_CATABOLIC\_PROCESS |  | 20 | -0.14 | -0.51 | 0.994 | 1.000 | 1.000 | 4542 | tags=35%, list=35%, signal=54% |
| 317 | CELLULAR\_CARBOHYDRATE\_CATABOLIC\_PROCESS |  | 20 | -0.14 | -0.51 | 0.985 | 0.999 | 1.000 | 4542 | tags=35%, list=35%, signal=54% |
| 318 | MONOVALENT\_INORGANIC\_CATION\_TRANSPORT |  | 83 | -0.09 | -0.45 | 1.000 | 0.999 | 1.000 | 5364 | tags=42%, list=41%, signal=71% |
Table: Gene sets enriched in phenotype **na**[plain text format]****

  
