# Supplementary material for: Multiple-input multiple-output causal strategies for gene selection
Source: BMC Bioinformatics. 2011 Nov 25;12:458. doi: 10.1186/1471-2105-12-458 (PMC3323860; doi:10.1186/1471-2105-12-458)
Supplement: Additional file 2 — Archive containing the output files computed by the preranked GSEA for λ ∈ {0.1,0.2,0.3,0.4,0.5} (GSEA_MIMO_part1.zip). [file 1471-2105-12-458-S2.ZIP › mFS00_entrez_mimo.GseaPreranked.1316037675010/gsea_report_for_na_pos_1316037675010.html]

Report for na\_pos 1316037675010 [GSEA]

| GS  follow link to MSigDB | GS DETAILS | SIZE | ES | NES | NOM p-val | FDR q-val | FWER p-val | RANK AT MAX | LEADING EDGE || 1 | M\_PHASE\_OF\_MITOTIC\_CELL\_CYCLE |  | 72 | 0.60 | 2.83 | 0.000 | 0.000 | 0.000 | 2048 | tags=56%, list=16%, signal=65% |
| 2 | MITOSIS |  | 70 | 0.59 | 2.81 | 0.000 | 0.000 | 0.000 | 2048 | tags=54%, list=16%, signal=64% |
| 3 | M\_PHASE |  | 98 | 0.55 | 2.78 | 0.000 | 0.000 | 0.000 | 2048 | tags=50%, list=16%, signal=59% |
| 4 | MITOTIC\_CELL\_CYCLE |  | 134 | 0.52 | 2.77 | 0.000 | 0.000 | 0.000 | 2513 | tags=51%, list=19%, signal=62% |
| 5 | CELL\_CYCLE\_PROCESS |  | 169 | 0.50 | 2.75 | 0.000 | 0.000 | 0.000 | 2726 | tags=50%, list=21%, signal=63% |
| 6 | CELL\_CYCLE\_PHASE |  | 152 | 0.49 | 2.65 | 0.000 | 0.000 | 0.000 | 2513 | tags=47%, list=19%, signal=58% |
| 7 | DNA\_REPLICATION |  | 97 | 0.50 | 2.55 | 0.000 | 0.000 | 0.000 | 2532 | tags=46%, list=19%, signal=57% |
| 8 | DNA\_METABOLIC\_PROCESS |  | 240 | 0.43 | 2.49 | 0.000 | 0.000 | 0.000 | 3374 | tags=50%, list=26%, signal=66% |
| 9 | SISTER\_CHROMATID\_SEGREGATION |  | 16 | 0.77 | 2.48 | 0.000 | 0.000 | 0.000 | 597 | tags=56%, list=5%, signal=59% |
| 10 | MITOTIC\_SISTER\_CHROMATID\_SEGREGATION |  | 15 | 0.78 | 2.48 | 0.000 | 0.000 | 0.000 | 597 | tags=60%, list=5%, signal=63% |
| 11 | CHROMOSOME\_SEGREGATION |  | 28 | 0.63 | 2.43 | 0.000 | 0.000 | 0.001 | 597 | tags=46%, list=5%, signal=49% |
| 12 | CELL\_CYCLE\_GO\_0007049 |  | 277 | 0.41 | 2.41 | 0.000 | 0.000 | 0.001 | 2726 | tags=43%, list=21%, signal=53% |
| 13 | CELL\_CYCLE\_CHECKPOINT\_GO\_0000075 |  | 45 | 0.54 | 2.37 | 0.000 | 0.000 | 0.001 | 1974 | tags=53%, list=15%, signal=63% |
| 14 | DNA\_DEPENDENT\_DNA\_REPLICATION |  | 52 | 0.52 | 2.35 | 0.000 | 0.000 | 0.001 | 2507 | tags=52%, list=19%, signal=64% |
| 15 | REGULATION\_OF\_MITOSIS |  | 33 | 0.59 | 2.34 | 0.000 | 0.000 | 0.001 | 1838 | tags=52%, list=14%, signal=60% |
| 16 | DNA\_REPAIR |  | 118 | 0.44 | 2.29 | 0.000 | 0.000 | 0.002 | 2513 | tags=45%, list=19%, signal=55% |
| 17 | RESPONSE\_TO\_DNA\_DAMAGE\_STIMULUS |  | 153 | 0.41 | 2.26 | 0.000 | 0.000 | 0.004 | 2640 | tags=44%, list=20%, signal=54% |
| 18 | RNA\_SPLICING |  | 74 | 0.47 | 2.26 | 0.000 | 0.000 | 0.004 | 3672 | tags=57%, list=28%, signal=78% |
| 19 | RNA\_PROCESSING |  | 138 | 0.41 | 2.17 | 0.000 | 0.001 | 0.015 | 2943 | tags=47%, list=22%, signal=60% |
| 20 | RESPONSE\_TO\_ENDOGENOUS\_STIMULUS |  | 182 | 0.38 | 2.15 | 0.000 | 0.001 | 0.024 | 3342 | tags=46%, list=26%, signal=61% |
| 21 | NUCLEOTIDE\_BIOSYNTHETIC\_PROCESS |  | 17 | 0.64 | 2.13 | 0.000 | 0.001 | 0.025 | 1596 | tags=53%, list=12%, signal=60% |
| 22 | MITOTIC\_CELL\_CYCLE\_CHECKPOINT |  | 19 | 0.61 | 2.11 | 0.002 | 0.001 | 0.034 | 1838 | tags=53%, list=14%, signal=61% |
| 23 | DNA\_INTEGRITY\_CHECKPOINT |  | 22 | 0.59 | 2.08 | 0.000 | 0.002 | 0.053 | 1672 | tags=55%, list=13%, signal=62% |
| 24 | MITOCHONDRION\_ORGANIZATION\_AND\_BIOGENESIS |  | 42 | 0.48 | 2.07 | 0.000 | 0.002 | 0.060 | 3604 | tags=57%, list=28%, signal=79% |
| 25 | MRNA\_METABOLIC\_PROCESS |  | 72 | 0.43 | 2.06 | 0.000 | 0.002 | 0.060 | 2943 | tags=49%, list=22%, signal=62% |
| 26 | MICROTUBULE\_CYTOSKELETON\_ORGANIZATION\_AND\_BIOGENESIS |  | 31 | 0.52 | 2.05 | 0.000 | 0.002 | 0.074 | 2726 | tags=55%, list=21%, signal=69% |
| 27 | NUCLEOBASENUCLEOSIDENUCLEOTIDE\_AND\_NUCLEIC\_ACID\_TRANSPORT |  | 26 | 0.53 | 2.05 | 0.000 | 0.002 | 0.081 | 2187 | tags=50%, list=17%, signal=60% |
| 28 | REGULATION\_OF\_CELL\_CYCLE |  | 161 | 0.37 | 2.05 | 0.000 | 0.002 | 0.082 | 1850 | tags=35%, list=14%, signal=40% |
| 29 | DNA\_REPLICATION\_INITIATION |  | 15 | 0.64 | 2.02 | 0.000 | 0.003 | 0.109 | 2930 | tags=80%, list=22%, signal=103% |
| 30 | PROTEIN\_FOLDING |  | 55 | 0.44 | 1.97 | 0.000 | 0.005 | 0.170 | 3086 | tags=49%, list=24%, signal=64% |
| 31 | REGULATION\_OF\_MITOTIC\_CELL\_CYCLE |  | 19 | 0.57 | 1.96 | 0.002 | 0.005 | 0.186 | 1080 | tags=47%, list=8%, signal=52% |
| 32 | MRNA\_PROCESSING\_GO\_0006397 |  | 61 | 0.43 | 1.95 | 0.002 | 0.005 | 0.199 | 2943 | tags=46%, list=22%, signal=59% |
| 33 | DOUBLE\_STRAND\_BREAK\_REPAIR |  | 21 | 0.55 | 1.95 | 0.004 | 0.006 | 0.215 | 2028 | tags=52%, list=15%, signal=62% |
| 34 | INTERPHASE\_OF\_MITOTIC\_CELL\_CYCLE |  | 57 | 0.42 | 1.93 | 0.000 | 0.006 | 0.237 | 3589 | tags=53%, list=27%, signal=72% |
| 35 | INTERPHASE |  | 63 | 0.42 | 1.92 | 0.000 | 0.007 | 0.283 | 3589 | tags=51%, list=27%, signal=70% |
| 36 | COENZYME\_METABOLIC\_PROCESS |  | 35 | 0.48 | 1.91 | 0.002 | 0.007 | 0.294 | 3515 | tags=49%, list=27%, signal=66% |
| 37 | CHROMOSOME\_ORGANIZATION\_AND\_BIOGENESIS |  | 107 | 0.37 | 1.91 | 0.000 | 0.008 | 0.312 | 3074 | tags=42%, list=23%, signal=55% |
| 38 | REGULATION\_OF\_CYCLIN\_DEPENDENT\_PROTEIN\_KINASE\_ACTIVITY |  | 40 | 0.45 | 1.90 | 0.002 | 0.008 | 0.325 | 1974 | tags=45%, list=15%, signal=53% |
| 39 | G1\_S\_TRANSITION\_OF\_MITOTIC\_CELL\_CYCLE |  | 23 | 0.51 | 1.90 | 0.000 | 0.008 | 0.351 | 2513 | tags=48%, list=19%, signal=59% |
| 40 | TRNA\_METABOLIC\_PROCESS |  | 15 | 0.60 | 1.89 | 0.005 | 0.008 | 0.357 | 2939 | tags=67%, list=22%, signal=86% |
| 41 | REGULATION\_OF\_DNA\_METABOLIC\_PROCESS |  | 40 | 0.44 | 1.87 | 0.000 | 0.010 | 0.434 | 2532 | tags=48%, list=19%, signal=59% |
| 42 | COFACTOR\_BIOSYNTHETIC\_PROCESS |  | 21 | 0.52 | 1.84 | 0.006 | 0.012 | 0.497 | 1544 | tags=38%, list=12%, signal=43% |
| 43 | DNA\_RECOMBINATION |  | 45 | 0.42 | 1.80 | 0.005 | 0.017 | 0.619 | 1296 | tags=33%, list=10%, signal=37% |
| 44 | REGULATION\_OF\_DNA\_REPLICATION |  | 18 | 0.53 | 1.80 | 0.011 | 0.017 | 0.632 | 2297 | tags=50%, list=18%, signal=61% |
| 45 | DNA\_DAMAGE\_CHECKPOINT |  | 19 | 0.53 | 1.79 | 0.004 | 0.018 | 0.652 | 2297 | tags=53%, list=18%, signal=64% |
| 46 | DNA\_DAMAGE\_RESPONSESIGNAL\_TRANSDUCTION |  | 34 | 0.46 | 1.79 | 0.002 | 0.018 | 0.652 | 2297 | tags=47%, list=18%, signal=57% |
| 47 | BIOPOLYMER\_CATABOLIC\_PROCESS |  | 103 | 0.35 | 1.78 | 0.000 | 0.018 | 0.675 | 2640 | tags=37%, list=20%, signal=46% |
| 48 | MITOCHONDRIAL\_TRANSPORT |  | 18 | 0.53 | 1.78 | 0.005 | 0.018 | 0.678 | 1324 | tags=44%, list=10%, signal=49% |
| 49 | PROTEIN\_CATABOLIC\_PROCESS |  | 60 | 0.39 | 1.76 | 0.004 | 0.021 | 0.738 | 2510 | tags=35%, list=19%, signal=43% |
| 50 | MEIOSIS\_I |  | 19 | 0.51 | 1.75 | 0.004 | 0.023 | 0.771 | 1296 | tags=37%, list=10%, signal=41% |
| 51 | NUCLEAR\_EXPORT |  | 26 | 0.47 | 1.74 | 0.005 | 0.024 | 0.799 | 2187 | tags=42%, list=17%, signal=51% |
| 52 | ONE\_CARBON\_COMPOUND\_METABOLIC\_PROCESS |  | 24 | 0.48 | 1.71 | 0.017 | 0.029 | 0.857 | 2026 | tags=46%, list=15%, signal=54% |
| 53 | DNA\_PACKAGING |  | 29 | 0.45 | 1.71 | 0.013 | 0.028 | 0.860 | 2600 | tags=45%, list=20%, signal=56% |
| 54 | UBIQUITIN\_CYCLE |  | 40 | 0.41 | 1.71 | 0.011 | 0.030 | 0.882 | 2260 | tags=38%, list=17%, signal=45% |
| 55 | PROTEIN\_MODIFICATION\_BY\_SMALL\_PROTEIN\_CONJUGATION |  | 35 | 0.42 | 1.70 | 0.007 | 0.031 | 0.897 | 2260 | tags=40%, list=17%, signal=48% |
| 56 | COFACTOR\_METABOLIC\_PROCESS |  | 51 | 0.38 | 1.70 | 0.004 | 0.030 | 0.898 | 3573 | tags=43%, list=27%, signal=59% |
| 57 | TRANSCRIPTION\_INITIATION\_FROM\_RNA\_POLYMERASE\_II\_PROMOTER |  | 27 | 0.45 | 1.69 | 0.017 | 0.031 | 0.913 | 3267 | tags=48%, list=25%, signal=64% |
| 58 | MACROMOLECULE\_CATABOLIC\_PROCESS |  | 120 | 0.32 | 1.69 | 0.003 | 0.032 | 0.917 | 2680 | tags=33%, list=20%, signal=42% |
| 59 | BASE\_EXCISION\_REPAIR |  | 16 | 0.50 | 1.67 | 0.011 | 0.036 | 0.941 | 2353 | tags=44%, list=18%, signal=53% |
| 60 | RNA\_EXPORT\_FROM\_NUCLEUS |  | 17 | 0.50 | 1.67 | 0.024 | 0.036 | 0.946 | 2187 | tags=47%, list=17%, signal=56% |
| 61 | CELLULAR\_PROTEIN\_CATABOLIC\_PROCESS |  | 50 | 0.38 | 1.66 | 0.013 | 0.038 | 0.955 | 2510 | tags=34%, list=19%, signal=42% |
| 62 | MEIOTIC\_CELL\_CYCLE |  | 31 | 0.43 | 1.63 | 0.011 | 0.045 | 0.977 | 1296 | tags=32%, list=10%, signal=36% |
| 63 | PROTEIN\_UBIQUITINATION |  | 32 | 0.41 | 1.62 | 0.014 | 0.049 | 0.985 | 2260 | tags=38%, list=17%, signal=45% |
| 64 | NUCLEOTIDE\_METABOLIC\_PROCESS |  | 36 | 0.39 | 1.59 | 0.028 | 0.060 | 0.995 | 790 | tags=28%, list=6%, signal=29% |
| 65 | CYTOKINESIS |  | 17 | 0.48 | 1.59 | 0.034 | 0.061 | 0.997 | 1100 | tags=35%, list=8%, signal=38% |
| 66 | NUCLEOBASENUCLEOSIDE\_AND\_NUCLEOTIDE\_METABOLIC\_PROCESS |  | 46 | 0.37 | 1.58 | 0.006 | 0.063 | 0.998 | 790 | tags=26%, list=6%, signal=28% |
| 67 | PROTEIN\_DNA\_COMPLEX\_ASSEMBLY |  | 45 | 0.37 | 1.58 | 0.012 | 0.062 | 0.998 | 2849 | tags=40%, list=22%, signal=51% |
| 68 | NUCLEAR\_TRANSPORT |  | 77 | 0.32 | 1.57 | 0.007 | 0.063 | 0.998 | 2611 | tags=34%, list=20%, signal=42% |
| 69 | CHROMATIN\_ASSEMBLY\_OR\_DISASSEMBLY |  | 25 | 0.42 | 1.57 | 0.020 | 0.063 | 0.998 | 2582 | tags=48%, list=20%, signal=60% |
| 70 | APOPTOTIC\_NUCLEAR\_CHANGES |  | 17 | 0.47 | 1.57 | 0.041 | 0.062 | 0.998 | 2202 | tags=47%, list=17%, signal=57% |
| 71 | CELLULAR\_COMPONENT\_DISASSEMBLY |  | 31 | 0.41 | 1.57 | 0.024 | 0.062 | 0.998 | 2202 | tags=39%, list=17%, signal=46% |
| 72 | NUCLEOCYTOPLASMIC\_TRANSPORT |  | 77 | 0.32 | 1.55 | 0.014 | 0.071 | 0.999 | 2611 | tags=34%, list=20%, signal=42% |
| 73 | ORGANELLE\_ORGANIZATION\_AND\_BIOGENESIS |  | 407 | 0.24 | 1.53 | 0.000 | 0.081 | 1.000 | 3082 | tags=32%, list=24%, signal=41% |
| 74 | CELLULAR\_MACROMOLECULE\_CATABOLIC\_PROCESS |  | 90 | 0.30 | 1.52 | 0.020 | 0.086 | 1.000 | 2306 | tags=29%, list=18%, signal=35% |
| 75 | ESTABLISHMENT\_OF\_ORGANELLE\_LOCALIZATION |  | 16 | 0.47 | 1.49 | 0.050 | 0.100 | 1.000 | 1748 | tags=44%, list=13%, signal=50% |
| 76 | VIRAL\_INFECTIOUS\_CYCLE |  | 29 | 0.38 | 1.47 | 0.054 | 0.114 | 1.000 | 942 | tags=31%, list=7%, signal=33% |
| 77 | MICROTUBULE\_BASED\_PROCESS |  | 75 | 0.31 | 1.47 | 0.021 | 0.114 | 1.000 | 2821 | tags=36%, list=22%, signal=46% |
| 78 | TRANSCRIPTION\_INITIATION |  | 33 | 0.37 | 1.47 | 0.049 | 0.114 | 1.000 | 2389 | tags=36%, list=18%, signal=44% |
| 79 | CELL\_DIVISION |  | 19 | 0.43 | 1.45 | 0.064 | 0.122 | 1.000 | 1100 | tags=32%, list=8%, signal=34% |
| 80 | NEGATIVE\_REGULATION\_OF\_DNA\_METABOLIC\_PROCESS |  | 16 | 0.44 | 1.45 | 0.071 | 0.121 | 1.000 | 2532 | tags=50%, list=19%, signal=62% |
| 81 | ORGANELLE\_LOCALIZATION |  | 21 | 0.40 | 1.44 | 0.077 | 0.129 | 1.000 | 1748 | tags=33%, list=13%, signal=38% |
| 82 | MEIOTIC\_RECOMBINATION |  | 16 | 0.44 | 1.42 | 0.096 | 0.148 | 1.000 | 1296 | tags=31%, list=10%, signal=35% |
| 83 | G1\_PHASE |  | 15 | 0.44 | 1.41 | 0.077 | 0.148 | 1.000 | 365 | tags=27%, list=3%, signal=27% |
| 84 | APOPTOTIC\_PROGRAM |  | 56 | 0.31 | 1.40 | 0.043 | 0.156 | 1.000 | 3625 | tags=46%, list=28%, signal=64% |
| 85 | ESTABLISHMENT\_AND\_OR\_MAINTENANCE\_OF\_CHROMATIN\_ARCHITECTURE |  | 65 | 0.30 | 1.40 | 0.054 | 0.159 | 1.000 | 3020 | tags=38%, list=23%, signal=50% |
| 86 | VIRAL\_REPRODUCTIVE\_PROCESS |  | 33 | 0.36 | 1.39 | 0.091 | 0.163 | 1.000 | 1077 | tags=30%, list=8%, signal=33% |
| 87 | ALCOHOL\_METABOLIC\_PROCESS |  | 82 | 0.28 | 1.39 | 0.052 | 0.162 | 1.000 | 2889 | tags=30%, list=22%, signal=39% |
| 88 | NEGATIVE\_REGULATION\_OF\_BINDING |  | 16 | 0.42 | 1.37 | 0.106 | 0.180 | 1.000 | 2688 | tags=50%, list=21%, signal=63% |
| 89 | CHROMATIN\_REMODELING |  | 21 | 0.39 | 1.37 | 0.102 | 0.188 | 1.000 | 3020 | tags=48%, list=23%, signal=62% |
| 90 | DNA\_CATABOLIC\_PROCESS |  | 21 | 0.39 | 1.35 | 0.115 | 0.202 | 1.000 | 3549 | tags=52%, list=27%, signal=72% |
| 91 | RESPONSE\_TO\_ABIOTIC\_STIMULUS |  | 79 | 0.27 | 1.33 | 0.068 | 0.223 | 1.000 | 2816 | tags=32%, list=22%, signal=40% |
| 92 | REGULATION\_OF\_GENE\_EXPRESSION\_EPIGENETIC |  | 27 | 0.35 | 1.33 | 0.097 | 0.221 | 1.000 | 2860 | tags=41%, list=22%, signal=52% |
| 93 | INTRACELLULAR\_TRANSPORT |  | 248 | 0.23 | 1.33 | 0.027 | 0.226 | 1.000 | 3334 | tags=33%, list=25%, signal=44% |
| 94 | RNA\_CATABOLIC\_PROCESS |  | 20 | 0.38 | 1.32 | 0.132 | 0.229 | 1.000 | 2187 | tags=45%, list=17%, signal=54% |
| 95 | VIRAL\_REPRODUCTION |  | 38 | 0.32 | 1.32 | 0.111 | 0.230 | 1.000 | 1077 | tags=26%, list=8%, signal=29% |
| 96 | NEGATIVE\_REGULATION\_OF\_CATALYTIC\_ACTIVITY |  | 61 | 0.29 | 1.32 | 0.097 | 0.232 | 1.000 | 2814 | tags=36%, list=21%, signal=46% |
| 97 | RESPONSE\_TO\_STRESS |  | 467 | 0.21 | 1.31 | 0.012 | 0.240 | 1.000 | 3365 | tags=33%, list=26%, signal=43% |
| 98 | NUCLEAR\_ORGANIZATION\_AND\_BIOGENESIS |  | 23 | 0.37 | 1.31 | 0.134 | 0.242 | 1.000 | 2202 | tags=39%, list=17%, signal=47% |
| 99 | RESPONSE\_TO\_HYPOXIA |  | 27 | 0.34 | 1.30 | 0.135 | 0.243 | 1.000 | 2285 | tags=33%, list=17%, signal=40% |
| 100 | VIRAL\_GENOME\_REPLICATION |  | 20 | 0.37 | 1.29 | 0.116 | 0.263 | 1.000 | 942 | tags=30%, list=7%, signal=32% |
| 101 | ESTABLISHMENT\_OF\_CELLULAR\_LOCALIZATION |  | 311 | 0.21 | 1.28 | 0.044 | 0.271 | 1.000 | 3334 | tags=31%, list=25%, signal=41% |
| 102 | RESPONSE\_TO\_ORGANIC\_SUBSTANCE |  | 27 | 0.34 | 1.27 | 0.150 | 0.279 | 1.000 | 2929 | tags=37%, list=22%, signal=48% |
| 103 | CHROMATIN\_ASSEMBLY |  | 16 | 0.40 | 1.27 | 0.175 | 0.278 | 1.000 | 2582 | tags=44%, list=20%, signal=54% |
| 104 | OXYGEN\_AND\_REACTIVE\_OXYGEN\_SPECIES\_METABOLIC\_PROCESS |  | 18 | 0.38 | 1.27 | 0.165 | 0.285 | 1.000 | 2786 | tags=44%, list=21%, signal=56% |
| 105 | CYTOSKELETON\_DEPENDENT\_INTRACELLULAR\_TRANSPORT |  | 25 | 0.34 | 1.25 | 0.168 | 0.301 | 1.000 | 4188 | tags=56%, list=32%, signal=82% |
| 106 | CELLULAR\_LOCALIZATION |  | 323 | 0.20 | 1.25 | 0.057 | 0.300 | 1.000 | 3334 | tags=31%, list=25%, signal=40% |
| 107 | CHROMATIN\_MODIFICATION |  | 46 | 0.29 | 1.24 | 0.158 | 0.318 | 1.000 | 3020 | tags=37%, list=23%, signal=48% |
| 108 | HETEROCYCLE\_METABOLIC\_PROCESS |  | 26 | 0.33 | 1.22 | 0.191 | 0.349 | 1.000 | 1544 | tags=23%, list=12%, signal=26% |
| 109 | CELL\_STRUCTURE\_DISASSEMBLY\_DURING\_APOPTOSIS |  | 17 | 0.37 | 1.22 | 0.218 | 0.347 | 1.000 | 2202 | tags=35%, list=17%, signal=42% |
| 110 | RIBONUCLEOPROTEIN\_COMPLEX\_BIOGENESIS\_AND\_ASSEMBLY |  | 68 | 0.26 | 1.22 | 0.155 | 0.344 | 1.000 | 3820 | tags=41%, list=29%, signal=58% |
| 111 | NEGATIVE\_REGULATION\_OF\_DNA\_BINDING |  | 15 | 0.38 | 1.22 | 0.234 | 0.346 | 1.000 | 2688 | tags=47%, list=21%, signal=59% |
| 112 | REGULATION\_OF\_KINASE\_ACTIVITY |  | 135 | 0.23 | 1.20 | 0.144 | 0.367 | 1.000 | 2196 | tags=24%, list=17%, signal=29% |
| 113 | GLUTAMATE\_SIGNALING\_PATHWAY |  | 17 | 0.35 | 1.19 | 0.229 | 0.398 | 1.000 | 3997 | tags=35%, list=31%, signal=51% |
| 114 | TRANSCRIPTION\_FROM\_RNA\_POLYMERASE\_II\_PROMOTER |  | 428 | 0.19 | 1.18 | 0.072 | 0.400 | 1.000 | 2864 | tags=26%, list=22%, signal=33% |
| 115 | REGULATION\_OF\_TRANSFERASE\_ACTIVITY |  | 137 | 0.22 | 1.18 | 0.146 | 0.396 | 1.000 | 2196 | tags=24%, list=17%, signal=29% |
| 116 | NITROGEN\_COMPOUND\_BIOSYNTHETIC\_PROCESS |  | 25 | 0.31 | 1.17 | 0.247 | 0.418 | 1.000 | 2285 | tags=28%, list=17%, signal=34% |
| 117 | REGULATION\_OF\_PROTEIN\_KINASE\_ACTIVITY |  | 133 | 0.22 | 1.16 | 0.163 | 0.428 | 1.000 | 2196 | tags=24%, list=17%, signal=29% |
| 118 | CELLULAR\_RESPONSE\_TO\_STIMULUS |  | 17 | 0.35 | 1.16 | 0.262 | 0.425 | 1.000 | 4930 | tags=65%, list=38%, signal=104% |
| 119 | CELLULAR\_RESPIRATION |  | 19 | 0.34 | 1.16 | 0.257 | 0.430 | 1.000 | 2652 | tags=37%, list=20%, signal=46% |
| 120 | RNA\_SPLICINGVIA\_TRANSESTERIFICATION\_REACTIONS |  | 27 | 0.31 | 1.16 | 0.252 | 0.429 | 1.000 | 3672 | tags=41%, list=28%, signal=57% |
| 121 | TRANSCRIPTION\_FROM\_RNA\_POLYMERASE\_III\_PROMOTER |  | 18 | 0.35 | 1.16 | 0.280 | 0.426 | 1.000 | 3945 | tags=56%, list=30%, signal=79% |
| 122 | REGULATION\_OF\_CATALYTIC\_ACTIVITY |  | 238 | 0.20 | 1.16 | 0.145 | 0.423 | 1.000 | 2885 | tags=27%, list=22%, signal=34% |
| 123 | REGULATION\_OF\_HYDROLASE\_ACTIVITY |  | 65 | 0.24 | 1.14 | 0.233 | 0.447 | 1.000 | 2814 | tags=32%, list=21%, signal=41% |
| 124 | NUCLEAR\_IMPORT |  | 47 | 0.26 | 1.14 | 0.257 | 0.457 | 1.000 | 2611 | tags=30%, list=20%, signal=37% |
| 125 | CATABOLIC\_PROCESS |  | 201 | 0.20 | 1.13 | 0.205 | 0.480 | 1.000 | 2680 | tags=25%, list=20%, signal=31% |
| 126 | INDUCTION\_OF\_APOPTOSIS\_BY\_EXTRACELLULAR\_SIGNALS |  | 25 | 0.31 | 1.13 | 0.310 | 0.478 | 1.000 | 2678 | tags=36%, list=20%, signal=45% |
| 127 | NEGATIVE\_REGULATION\_OF\_TRANSPORT |  | 18 | 0.33 | 1.11 | 0.311 | 0.515 | 1.000 | 3476 | tags=44%, list=27%, signal=60% |
| 128 | INTERACTION\_WITH\_HOST |  | 15 | 0.35 | 1.10 | 0.321 | 0.523 | 1.000 | 1077 | tags=27%, list=8%, signal=29% |
| 129 | REGULATION\_OF\_MOLECULAR\_FUNCTION |  | 275 | 0.18 | 1.10 | 0.228 | 0.526 | 1.000 | 2885 | tags=27%, list=22%, signal=33% |
| 130 | CELLULAR\_CATABOLIC\_PROCESS |  | 189 | 0.19 | 1.10 | 0.247 | 0.525 | 1.000 | 2680 | tags=25%, list=20%, signal=31% |
| 131 | CELLULAR\_BIOSYNTHETIC\_PROCESS |  | 273 | 0.18 | 1.09 | 0.258 | 0.544 | 1.000 | 1731 | tags=19%, list=13%, signal=21% |
| 132 | NEURON\_APOPTOSIS |  | 15 | 0.33 | 1.09 | 0.329 | 0.548 | 1.000 | 1253 | tags=27%, list=10%, signal=29% |
| 133 | DNA\_DAMAGE\_RESPONSESIGNAL\_TRANSDUCTION\_RESULTING\_IN\_INDUCTION\_OF\_APOPTOSIS |  | 15 | 0.33 | 1.07 | 0.367 | 0.597 | 1.000 | 1113 | tags=27%, list=9%, signal=29% |
| 134 | GAMETE\_GENERATION |  | 92 | 0.21 | 1.07 | 0.319 | 0.594 | 1.000 | 3926 | tags=34%, list=30%, signal=48% |
| 135 | REGULATION\_OF\_NEUROTRANSMITTER\_LEVELS |  | 23 | 0.29 | 1.05 | 0.385 | 0.628 | 1.000 | 920 | tags=17%, list=7%, signal=19% |
| 136 | RESPONSE\_TO\_TEMPERATURE\_STIMULUS |  | 16 | 0.33 | 1.04 | 0.410 | 0.643 | 1.000 | 3515 | tags=44%, list=27%, signal=60% |
| 137 | REGULATION\_OF\_PROTEIN\_STABILITY |  | 17 | 0.32 | 1.03 | 0.411 | 0.664 | 1.000 | 4129 | tags=41%, list=32%, signal=60% |
| 138 | STEROID\_BIOSYNTHETIC\_PROCESS |  | 22 | 0.29 | 1.03 | 0.414 | 0.663 | 1.000 | 4112 | tags=55%, list=31%, signal=79% |
| 139 | LIPID\_BIOSYNTHETIC\_PROCESS |  | 84 | 0.21 | 1.03 | 0.417 | 0.671 | 1.000 | 1253 | tags=18%, list=10%, signal=20% |
| 140 | DIGESTION |  | 42 | 0.24 | 1.02 | 0.398 | 0.693 | 1.000 | 3367 | tags=26%, list=26%, signal=35% |
| 141 | NEGATIVE\_REGULATION\_OF\_TRANSFERASE\_ACTIVITY |  | 27 | 0.27 | 1.02 | 0.457 | 0.695 | 1.000 | 2604 | tags=33%, list=20%, signal=42% |
| 142 | RESPONSE\_TO\_HORMONE\_STIMULUS |  | 26 | 0.26 | 1.01 | 0.441 | 0.712 | 1.000 | 3515 | tags=38%, list=27%, signal=52% |
| 143 | PIGMENT\_BIOSYNTHETIC\_PROCESS |  | 17 | 0.30 | 1.00 | 0.458 | 0.716 | 1.000 | 1544 | tags=24%, list=12%, signal=27% |
| 144 | MACROMOLECULE\_LOCALIZATION |  | 202 | 0.18 | 1.00 | 0.454 | 0.713 | 1.000 | 3222 | tags=28%, list=25%, signal=36% |
| 145 | INTRACELLULAR\_PROTEIN\_TRANSPORT |  | 127 | 0.19 | 1.00 | 0.460 | 0.711 | 1.000 | 3222 | tags=28%, list=25%, signal=37% |
| 146 | NEGATIVE\_REGULATION\_OF\_APOPTOSIS |  | 136 | 0.19 | 1.00 | 0.467 | 0.709 | 1.000 | 1837 | tags=21%, list=14%, signal=24% |
| 147 | PROTEIN\_IMPORT |  | 58 | 0.22 | 0.99 | 0.461 | 0.728 | 1.000 | 3334 | tags=29%, list=25%, signal=39% |
| 148 | CYTOSKELETON\_ORGANIZATION\_AND\_BIOGENESIS |  | 182 | 0.18 | 0.99 | 0.496 | 0.739 | 1.000 | 2855 | tags=26%, list=22%, signal=33% |
| 149 | STEROID\_METABOLIC\_PROCESS |  | 66 | 0.21 | 0.98 | 0.504 | 0.744 | 1.000 | 3772 | tags=35%, list=29%, signal=49% |
| 150 | NEGATIVE\_REGULATION\_OF\_PROGRAMMED\_CELL\_DEATH |  | 137 | 0.18 | 0.98 | 0.487 | 0.743 | 1.000 | 1837 | tags=20%, list=14%, signal=24% |
| 151 | PROTEIN\_TARGETING |  | 94 | 0.19 | 0.98 | 0.490 | 0.740 | 1.000 | 3334 | tags=29%, list=25%, signal=38% |
| 152 | SPLICEOSOME\_ASSEMBLY |  | 17 | 0.29 | 0.97 | 0.497 | 0.760 | 1.000 | 3672 | tags=41%, list=28%, signal=57% |
| 153 | PROTEIN\_TRANSPORT |  | 139 | 0.18 | 0.97 | 0.536 | 0.761 | 1.000 | 3222 | tags=28%, list=25%, signal=37% |
| 154 | SECONDARY\_METABOLIC\_PROCESS |  | 23 | 0.26 | 0.97 | 0.501 | 0.757 | 1.000 | 1544 | tags=22%, list=12%, signal=25% |
| 155 | AROMATIC\_COMPOUND\_METABOLIC\_PROCESS |  | 26 | 0.25 | 0.97 | 0.489 | 0.761 | 1.000 | 225 | tags=15%, list=2%, signal=16% |
| 156 | ENERGY\_DERIVATION\_BY\_OXIDATION\_OF\_ORGANIC\_COMPOUNDS |  | 37 | 0.24 | 0.96 | 0.499 | 0.762 | 1.000 | 1261 | tags=19%, list=10%, signal=21% |
| 157 | REGULATION\_OF\_PROGRAMMED\_CELL\_DEATH |  | 313 | 0.16 | 0.96 | 0.591 | 0.777 | 1.000 | 1705 | tags=17%, list=13%, signal=19% |
| 158 | COVALENT\_CHROMATIN\_MODIFICATION |  | 22 | 0.27 | 0.95 | 0.515 | 0.777 | 1.000 | 3943 | tags=45%, list=30%, signal=65% |
| 159 | PROGRAMMED\_CELL\_DEATH |  | 393 | 0.15 | 0.95 | 0.595 | 0.779 | 1.000 | 1837 | tags=17%, list=14%, signal=20% |
| 160 | PIGMENT\_METABOLIC\_PROCESS |  | 18 | 0.28 | 0.95 | 0.517 | 0.780 | 1.000 | 1544 | tags=22%, list=12%, signal=25% |
| 161 | APOPTOSIS\_GO |  | 392 | 0.15 | 0.95 | 0.613 | 0.778 | 1.000 | 1837 | tags=17%, list=14%, signal=20% |
| 162 | PROTEIN\_IMPORT\_INTO\_NUCLEUS |  | 45 | 0.22 | 0.95 | 0.537 | 0.781 | 1.000 | 2611 | tags=27%, list=20%, signal=33% |
| 163 | REGULATION\_OF\_APOPTOSIS |  | 312 | 0.16 | 0.94 | 0.620 | 0.784 | 1.000 | 1705 | tags=17%, list=13%, signal=19% |
| 164 | REGULATION\_OF\_TRANSCRIPTION\_FROM\_RNA\_POLYMERASE\_II\_PROMOTER |  | 267 | 0.16 | 0.94 | 0.596 | 0.783 | 1.000 | 3534 | tags=30%, list=27%, signal=40% |
| 165 | CARBOHYDRATE\_TRANSPORT |  | 17 | 0.28 | 0.94 | 0.558 | 0.791 | 1.000 | 2620 | tags=29%, list=20%, signal=37% |
| 166 | PROTEIN\_RNA\_COMPLEX\_ASSEMBLY |  | 55 | 0.21 | 0.93 | 0.560 | 0.793 | 1.000 | 3820 | tags=38%, list=29%, signal=54% |
| 167 | MICROTUBULE\_BASED\_MOVEMENT |  | 16 | 0.29 | 0.93 | 0.564 | 0.808 | 1.000 | 4188 | tags=50%, list=32%, signal=73% |
| 168 | STEROID\_HORMONE\_RECEPTOR\_SIGNALING\_PATHWAY |  | 18 | 0.27 | 0.92 | 0.566 | 0.808 | 1.000 | 1514 | tags=22%, list=12%, signal=25% |
| 169 | SEXUAL\_REPRODUCTION |  | 109 | 0.18 | 0.92 | 0.577 | 0.804 | 1.000 | 3926 | tags=30%, list=30%, signal=43% |
| 170 | REGULATION\_OF\_RNA\_METABOLIC\_PROCESS |  | 417 | 0.15 | 0.92 | 0.696 | 0.803 | 1.000 | 3464 | tags=29%, list=26%, signal=38% |
| 171 | INDUCTION\_OF\_APOPTOSIS\_BY\_INTRACELLULAR\_SIGNALS |  | 23 | 0.25 | 0.92 | 0.581 | 0.801 | 1.000 | 1454 | tags=22%, list=11%, signal=24% |
| 172 | GENERATION\_OF\_A\_SIGNAL\_INVOLVED\_IN\_CELL\_CELL\_SIGNALING |  | 25 | 0.25 | 0.92 | 0.589 | 0.814 | 1.000 | 2558 | tags=28%, list=20%, signal=35% |
| 173 | INTRACELLULAR\_RECEPTOR\_MEDIATED\_SIGNALING\_PATHWAY |  | 18 | 0.27 | 0.92 | 0.588 | 0.809 | 1.000 | 1514 | tags=22%, list=12%, signal=25% |
| 174 | PROTEIN\_AMINO\_ACID\_O\_LINKED\_GLYCOSYLATION |  | 18 | 0.27 | 0.91 | 0.559 | 0.810 | 1.000 | 3414 | tags=44%, list=26%, signal=60% |
| 175 | BIOSYNTHETIC\_PROCESS |  | 402 | 0.14 | 0.90 | 0.787 | 0.834 | 1.000 | 1731 | tags=16%, list=13%, signal=18% |
| 176 | POSITIVE\_REGULATION\_OF\_CELL\_CYCLE |  | 15 | 0.28 | 0.90 | 0.606 | 0.844 | 1.000 | 402 | tags=20%, list=3%, signal=21% |
| 177 | CELL\_PROJECTION\_BIOGENESIS |  | 20 | 0.26 | 0.89 | 0.606 | 0.844 | 1.000 | 4255 | tags=45%, list=33%, signal=67% |
| 178 | EXOCYTOSIS |  | 22 | 0.25 | 0.89 | 0.640 | 0.852 | 1.000 | 2310 | tags=23%, list=18%, signal=28% |
| 179 | MORPHOGENESIS\_OF\_AN\_EPITHELIUM |  | 15 | 0.28 | 0.89 | 0.588 | 0.851 | 1.000 | 3944 | tags=47%, list=30%, signal=67% |
| 180 | REGULATION\_OF\_TRANSCRIPTIONDNA\_DEPENDENT |  | 412 | 0.14 | 0.89 | 0.817 | 0.847 | 1.000 | 3464 | tags=28%, list=26%, signal=37% |
| 181 | ENERGY\_RESERVE\_METABOLIC\_PROCESS |  | 15 | 0.28 | 0.88 | 0.613 | 0.860 | 1.000 | 1261 | tags=20%, list=10%, signal=22% |
| 182 | CELLULAR\_CARBOHYDRATE\_METABOLIC\_PROCESS |  | 106 | 0.17 | 0.88 | 0.707 | 0.857 | 1.000 | 2889 | tags=24%, list=22%, signal=30% |
| 183 | REGULATION\_OF\_TRANSPORT |  | 57 | 0.20 | 0.87 | 0.672 | 0.864 | 1.000 | 3476 | tags=33%, list=27%, signal=45% |
| 184 | REGULATION\_OF\_INTRACELLULAR\_TRANSPORT |  | 22 | 0.25 | 0.87 | 0.646 | 0.864 | 1.000 | 4460 | tags=50%, list=34%, signal=76% |
| 185 | RESPONSE\_TO\_RADIATION |  | 52 | 0.19 | 0.87 | 0.682 | 0.870 | 1.000 | 3835 | tags=35%, list=29%, signal=49% |
| 186 | TRANSMISSION\_OF\_NERVE\_IMPULSE |  | 167 | 0.16 | 0.86 | 0.774 | 0.886 | 1.000 | 2838 | tags=21%, list=22%, signal=26% |
| 187 | EPIDERMAL\_GROWTH\_FACTOR\_RECEPTOR\_SIGNALING\_PATHWAY |  | 18 | 0.25 | 0.85 | 0.683 | 0.899 | 1.000 | 4449 | tags=44%, list=34%, signal=67% |
| 188 | NEGATIVE\_REGULATION\_OF\_CELL\_CYCLE |  | 72 | 0.18 | 0.85 | 0.744 | 0.902 | 1.000 | 1589 | tags=18%, list=12%, signal=20% |
| 189 | NEGATIVE\_REGULATION\_OF\_CELL\_ADHESION |  | 16 | 0.26 | 0.84 | 0.657 | 0.899 | 1.000 | 3497 | tags=44%, list=27%, signal=60% |
| 190 | MEMBRANE\_LIPID\_BIOSYNTHETIC\_PROCESS |  | 41 | 0.19 | 0.83 | 0.746 | 0.919 | 1.000 | 3758 | tags=34%, list=29%, signal=48% |
| 191 | SYNAPTIC\_TRANSMISSION |  | 154 | 0.15 | 0.83 | 0.829 | 0.915 | 1.000 | 2838 | tags=20%, list=22%, signal=25% |
| 192 | AEROBIC\_RESPIRATION |  | 15 | 0.26 | 0.83 | 0.700 | 0.912 | 1.000 | 2652 | tags=33%, list=20%, signal=42% |
| 193 | DEVELOPMENT\_OF\_PRIMARY\_SEXUAL\_CHARACTERISTICS |  | 25 | 0.22 | 0.82 | 0.736 | 0.925 | 1.000 | 3088 | tags=28%, list=24%, signal=37% |
| 194 | REGULATION\_OF\_NUCLEOCYTOPLASMIC\_TRANSPORT |  | 19 | 0.24 | 0.82 | 0.720 | 0.923 | 1.000 | 4460 | tags=53%, list=34%, signal=80% |
| 195 | CALCIUM\_INDEPENDENT\_CELL\_CELL\_ADHESION |  | 16 | 0.25 | 0.82 | 0.699 | 0.924 | 1.000 | 3242 | tags=31%, list=25%, signal=41% |
| 196 | HISTONE\_MODIFICATION |  | 21 | 0.23 | 0.82 | 0.703 | 0.921 | 1.000 | 3943 | tags=43%, list=30%, signal=61% |
| 197 | CELL\_CYCLE\_ARREST\_GO\_0007050 |  | 52 | 0.18 | 0.80 | 0.807 | 0.941 | 1.000 | 3975 | tags=40%, list=30%, signal=58% |
| 198 | EMBRYONIC\_DEVELOPMENT |  | 46 | 0.18 | 0.80 | 0.799 | 0.941 | 1.000 | 3088 | tags=28%, list=24%, signal=37% |
| 199 | GLUCOSE\_METABOLIC\_PROCESS |  | 27 | 0.21 | 0.79 | 0.777 | 0.950 | 1.000 | 4651 | tags=44%, list=36%, signal=69% |
| 200 | ESTABLISHMENT\_OF\_PROTEIN\_LOCALIZATION |  | 166 | 0.14 | 0.79 | 0.935 | 0.955 | 1.000 | 3222 | tags=25%, list=25%, signal=33% |
| 201 | CASPASE\_ACTIVATION |  | 24 | 0.21 | 0.78 | 0.778 | 0.953 | 1.000 | 4407 | tags=50%, list=34%, signal=75% |
| 202 | MEMBRANE\_FUSION |  | 27 | 0.21 | 0.78 | 0.783 | 0.949 | 1.000 | 3671 | tags=37%, list=28%, signal=51% |
| 203 | PHOSPHOINOSITIDE\_BIOSYNTHETIC\_PROCESS |  | 21 | 0.23 | 0.78 | 0.772 | 0.951 | 1.000 | 1225 | tags=19%, list=9%, signal=21% |
| 204 | REGULATION\_OF\_PHOSPHORYLATION |  | 42 | 0.18 | 0.78 | 0.838 | 0.948 | 1.000 | 4326 | tags=45%, list=33%, signal=67% |
| 205 | REGULATION\_OF\_CATABOLIC\_PROCESS |  | 15 | 0.25 | 0.78 | 0.756 | 0.945 | 1.000 | 2680 | tags=33%, list=20%, signal=42% |
| 206 | REPRODUCTION |  | 215 | 0.13 | 0.77 | 0.971 | 0.946 | 1.000 | 3109 | tags=22%, list=24%, signal=29% |
| 207 | PHOSPHOLIPID\_BIOSYNTHETIC\_PROCESS |  | 35 | 0.19 | 0.77 | 0.839 | 0.942 | 1.000 | 3758 | tags=34%, list=29%, signal=48% |
| 208 | RESPONSE\_TO\_UV |  | 22 | 0.21 | 0.77 | 0.789 | 0.945 | 1.000 | 3835 | tags=41%, list=29%, signal=58% |
| 209 | PROTEOLYSIS |  | 170 | 0.14 | 0.76 | 0.946 | 0.947 | 1.000 | 3728 | tags=29%, list=28%, signal=41% |
| 210 | LIPID\_TRANSPORT |  | 27 | 0.20 | 0.73 | 0.859 | 0.981 | 1.000 | 2057 | tags=22%, list=16%, signal=26% |
| 211 | STRESS\_ACTIVATED\_PROTEIN\_KINASE\_SIGNALING\_PATHWAY |  | 45 | 0.17 | 0.72 | 0.916 | 0.986 | 1.000 | 4179 | tags=38%, list=32%, signal=55% |
| 212 | ANION\_TRANSPORT |  | 27 | 0.19 | 0.72 | 0.865 | 0.983 | 1.000 | 2824 | tags=22%, list=22%, signal=28% |
| 213 | JNK\_CASCADE |  | 44 | 0.17 | 0.72 | 0.905 | 0.982 | 1.000 | 4179 | tags=39%, list=32%, signal=57% |
| 214 | SENSORY\_PERCEPTION |  | 163 | 0.13 | 0.71 | 0.978 | 0.984 | 1.000 | 5270 | tags=42%, list=40%, signal=69% |
| 215 | POSITIVE\_REGULATION\_OF\_HYDROLASE\_ACTIVITY |  | 45 | 0.16 | 0.70 | 0.935 | 0.984 | 1.000 | 2696 | tags=24%, list=21%, signal=31% |
| 216 | GLYCEROPHOSPHOLIPID\_BIOSYNTHETIC\_PROCESS |  | 27 | 0.18 | 0.70 | 0.901 | 0.984 | 1.000 | 1225 | tags=15%, list=9%, signal=16% |
| 217 | NEUROLOGICAL\_SYSTEM\_PROCESS |  | 328 | 0.11 | 0.69 | 1.000 | 0.989 | 1.000 | 2838 | tags=17%, list=22%, signal=22% |
| 218 | LIPOPROTEIN\_METABOLIC\_PROCESS |  | 30 | 0.18 | 0.68 | 0.921 | 0.992 | 1.000 | 2057 | tags=20%, list=16%, signal=24% |
| 219 | BRAIN\_DEVELOPMENT |  | 39 | 0.16 | 0.68 | 0.945 | 0.988 | 1.000 | 3088 | tags=28%, list=24%, signal=37% |
| 220 | BIOGENIC\_AMINE\_METABOLIC\_PROCESS |  | 16 | 0.21 | 0.68 | 0.892 | 0.984 | 1.000 | 1690 | tags=19%, list=13%, signal=22% |
| 221 | NEGATIVE\_REGULATION\_OF\_CELLULAR\_BIOSYNTHETIC\_PROCESS |  | 25 | 0.18 | 0.67 | 0.941 | 0.981 | 1.000 | 2734 | tags=24%, list=21%, signal=30% |
| 222 | HOMEOSTASIS\_OF\_NUMBER\_OF\_CELLS |  | 20 | 0.19 | 0.65 | 0.926 | 0.991 | 1.000 | 2682 | tags=25%, list=20%, signal=31% |
| 223 | NEGATIVE\_REGULATION\_OF\_BIOSYNTHETIC\_PROCESS |  | 26 | 0.17 | 0.63 | 0.936 | 0.996 | 1.000 | 2734 | tags=23%, list=21%, signal=29% |
| 224 | ADENYLATE\_CYCLASE\_ACTIVATION |  | 18 | 0.18 | 0.60 | 0.956 | 1.000 | 1.000 | 4540 | tags=39%, list=35%, signal=59% |
| 225 | REGULATION\_OF\_CELL\_ADHESION |  | 31 | 0.16 | 0.60 | 0.974 | 1.000 | 1.000 | 4206 | tags=42%, list=32%, signal=62% |
| 226 | FEEDING\_BEHAVIOR |  | 20 | 0.16 | 0.57 | 0.980 | 1.000 | 1.000 | 2696 | tags=20%, list=21%, signal=25% |
| 227 | DETECTION\_OF\_ABIOTIC\_STIMULUS |  | 16 | 0.17 | 0.55 | 0.970 | 1.000 | 1.000 | 63 | tags=6%, list=0%, signal=6% |
| 228 | LIPOPROTEIN\_BIOSYNTHETIC\_PROCESS |  | 23 | 0.15 | 0.55 | 0.979 | 0.999 | 1.000 | 4113 | tags=35%, list=31%, signal=51% |
| 229 | TUBE\_DEVELOPMENT |  | 15 | 0.16 | 0.50 | 0.990 | 1.000 | 1.000 | 1253 | tags=13%, list=10%, signal=15% |
| 230 | NEGATIVE\_REGULATION\_OF\_TRANSLATION |  | 19 | 0.14 | 0.46 | 1.000 | 0.999 | 1.000 | 3346 | tags=26%, list=26%, signal=35% |
Table: Gene sets enriched in phenotype **na**[plain text format]****

  
