# Supplementary material for: Multiple-input multiple-output causal strategies for gene selection
Source: BMC Bioinformatics. 2011 Nov 25;12:458. doi: 10.1186/1471-2105-12-458 (PMC3323860; doi:10.1186/1471-2105-12-458)
Supplement: Additional file 2 — Archive containing the output files computed by the preranked GSEA for λ ∈ {0.1,0.2,0.3,0.4,0.5} (GSEA_MIMO_part1.zip). [file 1471-2105-12-458-S2.ZIP › mFS01_entrez_mimo.GseaPreranked.1316037859455/gsea_report_for_na_neg_1316037859455.html]

Report for na\_neg 1316037859455 [GSEA]

| GS  follow link to MSigDB | GS DETAILS | SIZE | ES | NES | NOM p-val | FDR q-val | FWER p-val | RANK AT MAX | LEADING EDGE || 1 | IMMUNE\_RESPONSE |  | 212 | -0.39 | -2.32 | 0.000 | 0.006 | 0.005 | 4458 | tags=58%, list=34%, signal=87% |
| 2 | IMMUNE\_SYSTEM\_PROCESS |  | 298 | -0.35 | -2.22 | 0.000 | 0.008 | 0.013 | 3111 | tags=42%, list=24%, signal=53% |
| 3 | DEFENSE\_RESPONSE |  | 238 | -0.34 | -2.09 | 0.000 | 0.024 | 0.053 | 4075 | tags=47%, list=31%, signal=68% |
| 4 | POSITIVE\_REGULATION\_OF\_IMMUNE\_RESPONSE |  | 24 | -0.55 | -2.08 | 0.000 | 0.021 | 0.062 | 4328 | tags=75%, list=33%, signal=112% |
| 5 | REGULATION\_OF\_IMMUNE\_RESPONSE |  | 28 | -0.51 | -2.07 | 0.000 | 0.018 | 0.066 | 4535 | tags=75%, list=35%, signal=115% |
| 6 | POSITIVE\_REGULATION\_OF\_IMMUNE\_SYSTEM\_PROCESS |  | 44 | -0.45 | -1.99 | 0.000 | 0.033 | 0.142 | 4328 | tags=64%, list=33%, signal=95% |
| 7 | POSITIVE\_REGULATION\_OF\_MULTICELLULAR\_ORGANISMAL\_PROCESS |  | 56 | -0.42 | -1.94 | 0.000 | 0.045 | 0.209 | 4328 | tags=61%, list=33%, signal=90% |
| 8 | INFLAMMATORY\_RESPONSE |  | 115 | -0.36 | -1.92 | 0.000 | 0.051 | 0.269 | 3054 | tags=41%, list=23%, signal=53% |
| 9 | RESPONSE\_TO\_WOUNDING |  | 171 | -0.33 | -1.91 | 0.000 | 0.046 | 0.276 | 3054 | tags=39%, list=23%, signal=50% |
| 10 | REGULATION\_OF\_IMMUNE\_SYSTEM\_PROCESS |  | 57 | -0.40 | -1.89 | 0.000 | 0.051 | 0.324 | 4328 | tags=61%, list=33%, signal=91% |
| 11 | CELLULAR\_DEFENSE\_RESPONSE |  | 54 | -0.40 | -1.81 | 0.000 | 0.094 | 0.550 | 3650 | tags=46%, list=28%, signal=64% |
| 12 | ADAPTIVE\_IMMUNE\_RESPONSE\_GO\_0002460 |  | 22 | -0.50 | -1.80 | 0.014 | 0.094 | 0.582 | 4034 | tags=59%, list=31%, signal=85% |
| 13 | ADAPTIVE\_IMMUNE\_RESPONSE |  | 23 | -0.47 | -1.76 | 0.006 | 0.116 | 0.693 | 4034 | tags=57%, list=31%, signal=82% |
| 14 | REGULATION\_OF\_CELL\_DIFFERENTIATION |  | 48 | -0.38 | -1.72 | 0.000 | 0.150 | 0.807 | 4535 | tags=56%, list=35%, signal=86% |
| 15 | REGULATION\_OF\_DEFENSE\_RESPONSE |  | 15 | -0.53 | -1.70 | 0.023 | 0.172 | 0.857 | 4034 | tags=67%, list=31%, signal=96% |
| 16 | HUMORAL\_IMMUNE\_RESPONSE |  | 30 | -0.42 | -1.69 | 0.011 | 0.168 | 0.868 | 3111 | tags=53%, list=24%, signal=70% |
| 17 | HEMOPOIETIC\_OR\_LYMPHOID\_ORGAN\_DEVELOPMENT |  | 71 | -0.34 | -1.67 | 0.000 | 0.181 | 0.902 | 3006 | tags=39%, list=23%, signal=51% |
| 18 | REGULATION\_OF\_MULTICELLULAR\_ORGANISMAL\_PROCESS |  | 131 | -0.30 | -1.67 | 0.002 | 0.178 | 0.914 | 3103 | tags=37%, list=24%, signal=49% |
| 19 | TRANSFORMING\_GROWTH\_FACTOR\_BETA\_RECEPTOR\_SIGNALING\_PATHWAY |  | 34 | -0.40 | -1.66 | 0.020 | 0.177 | 0.921 | 3053 | tags=44%, list=23%, signal=57% |
| 20 | HEMOPOIESIS |  | 69 | -0.34 | -1.66 | 0.004 | 0.169 | 0.921 | 3006 | tags=39%, list=23%, signal=51% |
| 21 | IMMUNE\_EFFECTOR\_PROCESS |  | 34 | -0.40 | -1.65 | 0.009 | 0.171 | 0.932 | 2820 | tags=50%, list=22%, signal=64% |
| 22 | LIPID\_CATABOLIC\_PROCESS |  | 34 | -0.39 | -1.64 | 0.013 | 0.179 | 0.943 | 4089 | tags=56%, list=31%, signal=81% |
| 23 | IMMUNE\_SYSTEM\_DEVELOPMENT |  | 75 | -0.32 | -1.62 | 0.002 | 0.198 | 0.965 | 3006 | tags=39%, list=23%, signal=50% |
| 24 | RESPONSE\_TO\_EXTERNAL\_STIMULUS |  | 278 | -0.26 | -1.62 | 0.000 | 0.193 | 0.966 | 3112 | tags=33%, list=24%, signal=43% |
| 25 | RECEPTOR\_MEDIATED\_ENDOCYTOSIS |  | 31 | -0.40 | -1.57 | 0.016 | 0.254 | 0.990 | 2302 | tags=35%, list=18%, signal=43% |
| 26 | REGULATION\_OF\_ANGIOGENESIS |  | 24 | -0.42 | -1.57 | 0.035 | 0.248 | 0.991 | 1849 | tags=42%, list=14%, signal=48% |
| 27 | ACTIN\_CYTOSKELETON\_ORGANIZATION\_AND\_BIOGENESIS |  | 90 | -0.30 | -1.55 | 0.013 | 0.265 | 0.996 | 1916 | tags=27%, list=15%, signal=31% |
| 28 | LYMPHOCYTE\_ACTIVATION |  | 54 | -0.33 | -1.55 | 0.013 | 0.264 | 0.997 | 3404 | tags=46%, list=26%, signal=62% |
| 29 | ENZYME\_LINKED\_RECEPTOR\_PROTEIN\_SIGNALING\_PATHWAY |  | 128 | -0.28 | -1.55 | 0.005 | 0.259 | 0.998 | 2836 | tags=30%, list=22%, signal=39% |
| 30 | LEUKOCYTE\_DIFFERENTIATION |  | 34 | -0.37 | -1.54 | 0.015 | 0.257 | 0.999 | 3297 | tags=47%, list=25%, signal=63% |
| 31 | NEGATIVE\_REGULATION\_OF\_SIGNAL\_TRANSDUCTION |  | 31 | -0.38 | -1.54 | 0.041 | 0.249 | 0.999 | 4979 | tags=65%, list=38%, signal=104% |
| 32 | CELL\_ACTIVATION |  | 64 | -0.32 | -1.54 | 0.007 | 0.247 | 0.999 | 4077 | tags=52%, list=31%, signal=75% |
| 33 | JAK\_STAT\_CASCADE |  | 26 | -0.40 | -1.54 | 0.038 | 0.243 | 0.999 | 3069 | tags=46%, list=23%, signal=60% |
| 34 | POSITIVE\_REGULATION\_OF\_RESPONSE\_TO\_STIMULUS |  | 35 | -0.36 | -1.52 | 0.031 | 0.256 | 0.999 | 4328 | tags=60%, list=33%, signal=89% |
| 35 | TRANSMEMBRANE\_RECEPTOR\_PROTEIN\_SERINE\_THREONINE\_KINASE\_SIGNALING\_PATHWAY |  | 42 | -0.35 | -1.52 | 0.025 | 0.264 | 0.999 | 3224 | tags=40%, list=25%, signal=54% |
| 36 | T\_CELL\_ACTIVATION |  | 39 | -0.35 | -1.52 | 0.036 | 0.258 | 0.999 | 4034 | tags=51%, list=31%, signal=74% |
| 37 | POSITIVE\_REGULATION\_OF\_PHOSPHATE\_METABOLIC\_PROCESS |  | 23 | -0.40 | -1.50 | 0.041 | 0.278 | 0.999 | 1337 | tags=30%, list=10%, signal=34% |
| 38 | REGULATION\_OF\_RESPONSE\_TO\_STIMULUS |  | 49 | -0.33 | -1.49 | 0.022 | 0.295 | 0.999 | 4328 | tags=59%, list=33%, signal=88% |
| 39 | WOUND\_HEALING |  | 49 | -0.33 | -1.48 | 0.036 | 0.297 | 1.000 | 3556 | tags=41%, list=27%, signal=56% |
| 40 | POSITIVE\_REGULATION\_OF\_CELL\_DIFFERENTIATION |  | 21 | -0.41 | -1.48 | 0.057 | 0.291 | 1.000 | 4509 | tags=67%, list=34%, signal=102% |
| 41 | LYMPHOCYTE\_DIFFERENTIATION |  | 23 | -0.40 | -1.47 | 0.054 | 0.295 | 1.000 | 3297 | tags=52%, list=25%, signal=70% |
| 42 | CELLULAR\_LIPID\_CATABOLIC\_PROCESS |  | 31 | -0.36 | -1.47 | 0.051 | 0.288 | 1.000 | 4065 | tags=52%, list=31%, signal=75% |
| 43 | AMINE\_TRANSPORT |  | 36 | -0.36 | -1.47 | 0.027 | 0.284 | 1.000 | 2021 | tags=28%, list=15%, signal=33% |
| 44 | LEUKOCYTE\_ACTIVATION |  | 59 | -0.31 | -1.47 | 0.029 | 0.287 | 1.000 | 4034 | tags=51%, list=31%, signal=73% |
| 45 | MESODERM\_DEVELOPMENT |  | 22 | -0.40 | -1.47 | 0.060 | 0.281 | 1.000 | 4335 | tags=55%, list=33%, signal=81% |
| 46 | INNATE\_IMMUNE\_RESPONSE |  | 19 | -0.41 | -1.47 | 0.056 | 0.277 | 1.000 | 4815 | tags=74%, list=37%, signal=116% |
| 47 | PROTEIN\_AMINO\_ACID\_PHOSPHORYLATION |  | 231 | -0.24 | -1.46 | 0.000 | 0.272 | 1.000 | 3072 | tags=31%, list=23%, signal=40% |
| 48 | B\_CELL\_ACTIVATION |  | 17 | -0.42 | -1.44 | 0.079 | 0.317 | 1.000 | 3404 | tags=59%, list=26%, signal=79% |
| 49 | GROWTH |  | 59 | -0.30 | -1.43 | 0.041 | 0.320 | 1.000 | 3925 | tags=44%, list=30%, signal=63% |
| 50 | RESPONSE\_TO\_OTHER\_ORGANISM |  | 69 | -0.29 | -1.42 | 0.046 | 0.332 | 1.000 | 2894 | tags=38%, list=22%, signal=48% |
| 51 | COAGULATION |  | 41 | -0.33 | -1.42 | 0.043 | 0.335 | 1.000 | 1797 | tags=27%, list=14%, signal=31% |
| 52 | BLOOD\_COAGULATION |  | 41 | -0.33 | -1.41 | 0.072 | 0.336 | 1.000 | 1797 | tags=27%, list=14%, signal=31% |
| 53 | SMALL\_GTPASE\_MEDIATED\_SIGNAL\_TRANSDUCTION |  | 77 | -0.28 | -1.41 | 0.024 | 0.334 | 1.000 | 3244 | tags=39%, list=25%, signal=51% |
| 54 | CYTOKINE\_AND\_CHEMOKINE\_MEDIATED\_SIGNALING\_PATHWAY |  | 19 | -0.40 | -1.41 | 0.067 | 0.328 | 1.000 | 2180 | tags=37%, list=17%, signal=44% |
| 55 | PROTEIN\_AMINO\_ACID\_N\_LINKED\_GLYCOSYLATION |  | 27 | -0.35 | -1.41 | 0.060 | 0.329 | 1.000 | 2585 | tags=37%, list=20%, signal=46% |
| 56 | FATTY\_ACID\_METABOLIC\_PROCESS |  | 56 | -0.30 | -1.40 | 0.055 | 0.344 | 1.000 | 4065 | tags=50%, list=31%, signal=72% |
| 57 | CELL\_SUBSTRATE\_ADHESION |  | 36 | -0.33 | -1.39 | 0.082 | 0.345 | 1.000 | 1713 | tags=31%, list=13%, signal=35% |
| 58 | POSITIVE\_REGULATION\_OF\_PROTEIN\_AMINO\_ACID\_PHOSPHORYLATION |  | 15 | -0.43 | -1.39 | 0.088 | 0.340 | 1.000 | 1337 | tags=33%, list=10%, signal=37% |
| 59 | POSITIVE\_REGULATION\_OF\_SIGNAL\_TRANSDUCTION |  | 97 | -0.26 | -1.39 | 0.021 | 0.337 | 1.000 | 4080 | tags=47%, list=31%, signal=68% |
| 60 | AMINO\_ACID\_TRANSPORT |  | 25 | -0.36 | -1.39 | 0.074 | 0.333 | 1.000 | 2021 | tags=32%, list=15%, signal=38% |
| 61 | TRANSMEMBRANE\_RECEPTOR\_PROTEIN\_TYROSINE\_KINASE\_SIGNALING\_PATHWAY |  | 76 | -0.28 | -1.39 | 0.046 | 0.335 | 1.000 | 2625 | tags=29%, list=20%, signal=36% |
| 62 | FEMALE\_PREGNANCY |  | 42 | -0.32 | -1.39 | 0.076 | 0.333 | 1.000 | 4334 | tags=55%, list=33%, signal=82% |
| 63 | MULTI\_ORGANISM\_PROCESS |  | 137 | -0.25 | -1.39 | 0.024 | 0.328 | 1.000 | 3457 | tags=38%, list=26%, signal=51% |
| 64 | REGULATION\_OF\_SIGNAL\_TRANSDUCTION |  | 173 | -0.24 | -1.38 | 0.027 | 0.341 | 1.000 | 3824 | tags=40%, list=29%, signal=56% |
| 65 | REGULATION\_OF\_BLOOD\_PRESSURE |  | 22 | -0.37 | -1.38 | 0.101 | 0.338 | 1.000 | 3500 | tags=41%, list=27%, signal=56% |
| 66 | ANATOMICAL\_STRUCTURE\_FORMATION |  | 52 | -0.29 | -1.37 | 0.065 | 0.345 | 1.000 | 1849 | tags=29%, list=14%, signal=33% |
| 67 | ACTIN\_FILAMENT\_BASED\_PROCESS |  | 99 | -0.26 | -1.37 | 0.035 | 0.342 | 1.000 | 1916 | tags=24%, list=15%, signal=28% |
| 68 | CATION\_HOMEOSTASIS |  | 94 | -0.26 | -1.36 | 0.040 | 0.351 | 1.000 | 3289 | tags=36%, list=25%, signal=48% |
| 69 | HEMOSTASIS |  | 46 | -0.31 | -1.36 | 0.063 | 0.354 | 1.000 | 1797 | tags=26%, list=14%, signal=30% |
| 70 | REGULATION\_OF\_ANATOMICAL\_STRUCTURE\_MORPHOGENESIS |  | 17 | -0.39 | -1.35 | 0.111 | 0.365 | 1.000 | 4602 | tags=53%, list=35%, signal=82% |
| 71 | REGULATION\_OF\_PROTEIN\_AMINO\_ACID\_PHOSPHORYLATION |  | 23 | -0.37 | -1.35 | 0.104 | 0.370 | 1.000 | 2682 | tags=35%, list=20%, signal=44% |
| 72 | RESPONSE\_TO\_VIRUS |  | 45 | -0.30 | -1.34 | 0.084 | 0.369 | 1.000 | 2886 | tags=42%, list=22%, signal=54% |
| 73 | PEPTIDYL\_TYROSINE\_MODIFICATION |  | 23 | -0.37 | -1.34 | 0.098 | 0.370 | 1.000 | 1592 | tags=26%, list=12%, signal=30% |
| 74 | REGULATION\_OF\_BODY\_FLUID\_LEVELS |  | 55 | -0.29 | -1.34 | 0.070 | 0.371 | 1.000 | 1797 | tags=25%, list=14%, signal=29% |
| 75 | REGULATION\_OF\_LYMPHOCYTE\_ACTIVATION |  | 31 | -0.34 | -1.34 | 0.107 | 0.368 | 1.000 | 3297 | tags=45%, list=25%, signal=60% |
| 76 | DETECTION\_OF\_STIMULUS |  | 36 | -0.32 | -1.33 | 0.095 | 0.376 | 1.000 | 5345 | tags=58%, list=41%, signal=98% |
| 77 | CELLULAR\_CATION\_HOMEOSTASIS |  | 91 | -0.26 | -1.33 | 0.067 | 0.377 | 1.000 | 3289 | tags=36%, list=25%, signal=48% |
| 78 | MAINTENANCE\_OF\_LOCALIZATION |  | 21 | -0.37 | -1.33 | 0.139 | 0.373 | 1.000 | 2745 | tags=38%, list=21%, signal=48% |
| 79 | POSITIVE\_REGULATION\_OF\_CYTOKINE\_BIOSYNTHETIC\_PROCESS |  | 21 | -0.37 | -1.32 | 0.119 | 0.374 | 1.000 | 2931 | tags=43%, list=22%, signal=55% |
| 80 | POSITIVE\_REGULATION\_OF\_PHOSPHORYLATION |  | 21 | -0.37 | -1.31 | 0.138 | 0.401 | 1.000 | 1337 | tags=29%, list=10%, signal=32% |
| 81 | PROTEIN\_AMINO\_ACID\_DEPHOSPHORYLATION |  | 60 | -0.28 | -1.31 | 0.108 | 0.403 | 1.000 | 1518 | tags=22%, list=12%, signal=24% |
| 82 | PROTEIN\_PROCESSING |  | 41 | -0.30 | -1.30 | 0.105 | 0.411 | 1.000 | 4069 | tags=41%, list=31%, signal=60% |
| 83 | RAS\_PROTEIN\_SIGNAL\_TRANSDUCTION |  | 55 | -0.28 | -1.30 | 0.102 | 0.408 | 1.000 | 3394 | tags=42%, list=26%, signal=56% |
| 84 | GLYCOPROTEIN\_METABOLIC\_PROCESS |  | 82 | -0.25 | -1.30 | 0.067 | 0.405 | 1.000 | 3702 | tags=39%, list=28%, signal=54% |
| 85 | ANGIOGENESIS |  | 44 | -0.30 | -1.30 | 0.116 | 0.402 | 1.000 | 1849 | tags=30%, list=14%, signal=34% |
| 86 | MUSCLE\_DEVELOPMENT |  | 85 | -0.26 | -1.30 | 0.071 | 0.399 | 1.000 | 3472 | tags=40%, list=27%, signal=54% |
| 87 | RESPONSE\_TO\_DRUG |  | 21 | -0.36 | -1.29 | 0.151 | 0.405 | 1.000 | 2523 | tags=43%, list=19%, signal=53% |
| 88 | PROTEIN\_COMPLEX\_ASSEMBLY |  | 157 | -0.22 | -1.28 | 0.052 | 0.434 | 1.000 | 2530 | tags=27%, list=19%, signal=33% |
| 89 | DEPHOSPHORYLATION |  | 67 | -0.25 | -1.27 | 0.107 | 0.436 | 1.000 | 1518 | tags=21%, list=12%, signal=24% |
| 90 | POSITIVE\_REGULATION\_OF\_SECRETION |  | 18 | -0.37 | -1.27 | 0.161 | 0.434 | 1.000 | 4297 | tags=61%, list=33%, signal=91% |
| 91 | POSITIVE\_REGULATION\_OF\_LYMPHOCYTE\_ACTIVATION |  | 23 | -0.34 | -1.27 | 0.136 | 0.432 | 1.000 | 3297 | tags=43%, list=25%, signal=58% |
| 92 | PROTEIN\_KINASE\_CASCADE |  | 239 | -0.21 | -1.27 | 0.041 | 0.432 | 1.000 | 2299 | tags=25%, list=18%, signal=30% |
| 93 | REGULATION\_OF\_T\_CELL\_ACTIVATION |  | 25 | -0.33 | -1.27 | 0.142 | 0.430 | 1.000 | 3297 | tags=44%, list=25%, signal=59% |
| 94 | CELL\_MATRIX\_ADHESION |  | 35 | -0.30 | -1.26 | 0.140 | 0.436 | 1.000 | 1713 | tags=29%, list=13%, signal=33% |
| 95 | ACTIN\_POLYMERIZATION\_AND\_OR\_DEPOLYMERIZATION |  | 20 | -0.36 | -1.26 | 0.178 | 0.439 | 1.000 | 1496 | tags=25%, list=11%, signal=28% |
| 96 | PHOSPHORYLATION |  | 262 | -0.20 | -1.26 | 0.050 | 0.447 | 1.000 | 3072 | tags=30%, list=23%, signal=38% |
| 97 | CYTOKINE\_PRODUCTION |  | 61 | -0.27 | -1.25 | 0.111 | 0.452 | 1.000 | 2931 | tags=34%, list=22%, signal=44% |
| 98 | PROTEIN\_OLIGOMERIZATION |  | 37 | -0.31 | -1.25 | 0.133 | 0.449 | 1.000 | 2530 | tags=32%, list=19%, signal=40% |
| 99 | PHOSPHOLIPID\_METABOLIC\_PROCESS |  | 63 | -0.26 | -1.25 | 0.114 | 0.454 | 1.000 | 3888 | tags=43%, list=30%, signal=61% |
| 100 | LIPID\_METABOLIC\_PROCESS |  | 283 | -0.20 | -1.24 | 0.055 | 0.465 | 1.000 | 3944 | tags=39%, list=30%, signal=54% |
| 101 | PROTEIN\_AUTOPROCESSING |  | 24 | -0.33 | -1.24 | 0.174 | 0.462 | 1.000 | 4069 | tags=46%, list=31%, signal=66% |
| 102 | ICOSANOID\_METABOLIC\_PROCESS |  | 16 | -0.38 | -1.24 | 0.215 | 0.459 | 1.000 | 3675 | tags=50%, list=28%, signal=69% |
| 103 | MONOCARBOXYLIC\_ACID\_METABOLIC\_PROCESS |  | 77 | -0.25 | -1.23 | 0.103 | 0.466 | 1.000 | 4201 | tags=45%, list=32%, signal=67% |
| 104 | REGULATION\_OF\_CYTOSKELETON\_ORGANIZATION\_AND\_BIOGENESIS |  | 26 | -0.32 | -1.23 | 0.170 | 0.469 | 1.000 | 2682 | tags=35%, list=20%, signal=43% |
| 105 | PEPTIDYL\_TYROSINE\_PHOSPHORYLATION |  | 21 | -0.33 | -1.23 | 0.188 | 0.466 | 1.000 | 3038 | tags=33%, list=23%, signal=43% |
| 106 | POSITIVE\_REGULATION\_OF\_TRANSLATION |  | 28 | -0.31 | -1.23 | 0.180 | 0.467 | 1.000 | 2931 | tags=39%, list=22%, signal=51% |
| 107 | VASCULATURE\_DEVELOPMENT |  | 50 | -0.26 | -1.23 | 0.142 | 0.465 | 1.000 | 4374 | tags=50%, list=33%, signal=75% |
| 108 | REGULATION\_OF\_CELL\_PROLIFERATION |  | 275 | -0.20 | -1.22 | 0.046 | 0.466 | 1.000 | 2743 | tags=27%, list=21%, signal=33% |
| 109 | PROTEIN\_AMINO\_ACID\_AUTOPHOSPHORYLATION |  | 24 | -0.33 | -1.22 | 0.165 | 0.464 | 1.000 | 4069 | tags=46%, list=31%, signal=66% |
| 110 | POSITIVE\_REGULATION\_OF\_CELL\_PROLIFERATION |  | 129 | -0.22 | -1.22 | 0.111 | 0.463 | 1.000 | 1983 | tags=23%, list=15%, signal=27% |
| 111 | REGULATION\_OF\_I\_KAPPAB\_KINASE\_NF\_KAPPAB\_CASCADE |  | 72 | -0.25 | -1.22 | 0.132 | 0.465 | 1.000 | 3816 | tags=46%, list=29%, signal=64% |
| 112 | CAMP\_MEDIATED\_SIGNALING |  | 63 | -0.25 | -1.22 | 0.152 | 0.465 | 1.000 | 2156 | tags=21%, list=16%, signal=25% |
| 113 | G\_PROTEIN\_SIGNALING\_COUPLED\_TO\_CAMP\_NUCLEOTIDE\_SECOND\_MESSENGER |  | 62 | -0.25 | -1.22 | 0.164 | 0.461 | 1.000 | 2156 | tags=21%, list=16%, signal=25% |
| 114 | AMINO\_ACID\_CATABOLIC\_PROCESS |  | 23 | -0.33 | -1.22 | 0.194 | 0.458 | 1.000 | 2079 | tags=35%, list=16%, signal=41% |
| 115 | NEGATIVE\_REGULATION\_OF\_TRANSCRIPTION |  | 166 | -0.21 | -1.22 | 0.111 | 0.457 | 1.000 | 2718 | tags=28%, list=21%, signal=35% |
| 116 | POSITIVE\_REGULATION\_OF\_TRANSFERASE\_ACTIVITY |  | 71 | -0.25 | -1.21 | 0.138 | 0.470 | 1.000 | 2444 | tags=27%, list=19%, signal=33% |
| 117 | MUSCLE\_CELL\_DIFFERENTIATION |  | 21 | -0.33 | -1.20 | 0.215 | 0.495 | 1.000 | 3472 | tags=48%, list=27%, signal=65% |
| 118 | ORGAN\_MORPHOGENESIS |  | 131 | -0.21 | -1.20 | 0.119 | 0.492 | 1.000 | 1501 | tags=19%, list=11%, signal=21% |
| 119 | REGULATION\_OF\_MYELOID\_CELL\_DIFFERENTIATION |  | 19 | -0.34 | -1.19 | 0.232 | 0.498 | 1.000 | 4535 | tags=58%, list=35%, signal=88% |
| 120 | RESPONSE\_TO\_BACTERIUM |  | 22 | -0.32 | -1.19 | 0.243 | 0.511 | 1.000 | 1849 | tags=27%, list=14%, signal=32% |
| 121 | REGULATION\_OF\_PROTEIN\_METABOLIC\_PROCESS |  | 150 | -0.21 | -1.18 | 0.141 | 0.519 | 1.000 | 2705 | tags=28%, list=21%, signal=35% |
| 122 | CELL\_RECOGNITION |  | 16 | -0.36 | -1.18 | 0.241 | 0.516 | 1.000 | 4034 | tags=50%, list=31%, signal=72% |
| 123 | REGULATION\_OF\_MAP\_KINASE\_ACTIVITY |  | 56 | -0.25 | -1.18 | 0.198 | 0.514 | 1.000 | 2061 | tags=29%, list=16%, signal=34% |
| 124 | REGULATION\_OF\_ORGANELLE\_ORGANIZATION\_AND\_BIOGENESIS |  | 35 | -0.28 | -1.17 | 0.249 | 0.535 | 1.000 | 3207 | tags=37%, list=24%, signal=49% |
| 125 | GLYCEROPHOSPHOLIPID\_METABOLIC\_PROCESS |  | 39 | -0.27 | -1.17 | 0.237 | 0.536 | 1.000 | 3888 | tags=44%, list=30%, signal=62% |
| 126 | ACTIVATION\_OF\_MAPK\_ACTIVITY |  | 33 | -0.29 | -1.17 | 0.223 | 0.532 | 1.000 | 2061 | tags=30%, list=16%, signal=36% |
| 127 | AMINE\_CATABOLIC\_PROCESS |  | 25 | -0.31 | -1.17 | 0.225 | 0.529 | 1.000 | 2079 | tags=32%, list=16%, signal=38% |
| 128 | POSITIVE\_REGULATION\_OF\_PROTEIN\_MODIFICATION\_PROCESS |  | 24 | -0.31 | -1.16 | 0.246 | 0.540 | 1.000 | 1337 | tags=25%, list=10%, signal=28% |
| 129 | BONE\_REMODELING |  | 28 | -0.30 | -1.16 | 0.251 | 0.536 | 1.000 | 2300 | tags=29%, list=18%, signal=35% |
| 130 | POSITIVE\_REGULATION\_OF\_CELLULAR\_PROTEIN\_METABOLIC\_PROCESS |  | 61 | -0.24 | -1.16 | 0.220 | 0.542 | 1.000 | 2450 | tags=30%, list=19%, signal=36% |
| 131 | BEHAVIOR |  | 136 | -0.21 | -1.16 | 0.207 | 0.539 | 1.000 | 4458 | tags=43%, list=34%, signal=64% |
| 132 | POSITIVE\_REGULATION\_OF\_PROTEIN\_METABOLIC\_PROCESS |  | 63 | -0.24 | -1.15 | 0.224 | 0.543 | 1.000 | 2450 | tags=30%, list=19%, signal=37% |
| 133 | VITAMIN\_METABOLIC\_PROCESS |  | 15 | -0.36 | -1.15 | 0.263 | 0.542 | 1.000 | 4150 | tags=60%, list=32%, signal=88% |
| 134 | GENERATION\_OF\_PRECURSOR\_METABOLITES\_AND\_ENERGY |  | 120 | -0.21 | -1.15 | 0.180 | 0.540 | 1.000 | 3189 | tags=32%, list=24%, signal=41% |
| 135 | POSITIVE\_REGULATION\_OF\_I\_KAPPAB\_KINASE\_NF\_KAPPAB\_CASCADE |  | 67 | -0.23 | -1.15 | 0.207 | 0.547 | 1.000 | 3816 | tags=45%, list=29%, signal=63% |
| 136 | GENERATION\_OF\_NEURONS |  | 65 | -0.24 | -1.14 | 0.220 | 0.566 | 1.000 | 3494 | tags=34%, list=27%, signal=46% |
| 137 | ACTIVATION\_OF\_NF\_KAPPAB\_TRANSCRIPTION\_FACTOR |  | 15 | -0.35 | -1.14 | 0.259 | 0.565 | 1.000 | 4294 | tags=60%, list=33%, signal=89% |
| 138 | NITROGEN\_COMPOUND\_CATABOLIC\_PROCESS |  | 27 | -0.30 | -1.14 | 0.272 | 0.562 | 1.000 | 2079 | tags=30%, list=16%, signal=35% |
| 139 | NEGATIVE\_REGULATION\_OF\_RNA\_METABOLIC\_PROCESS |  | 114 | -0.21 | -1.14 | 0.192 | 0.559 | 1.000 | 2914 | tags=30%, list=22%, signal=38% |
| 140 | DEVELOPMENTAL\_MATURATION |  | 18 | -0.33 | -1.13 | 0.273 | 0.561 | 1.000 | 3234 | tags=39%, list=25%, signal=52% |
| 141 | STRIATED\_MUSCLE\_DEVELOPMENT |  | 36 | -0.27 | -1.13 | 0.263 | 0.560 | 1.000 | 3660 | tags=44%, list=28%, signal=62% |
| 142 | REGULATION\_OF\_CELLULAR\_PROTEIN\_METABOLIC\_PROCESS |  | 139 | -0.20 | -1.13 | 0.204 | 0.557 | 1.000 | 2705 | tags=27%, list=21%, signal=34% |
| 143 | SKELETAL\_DEVELOPMENT |  | 91 | -0.21 | -1.13 | 0.252 | 0.567 | 1.000 | 2660 | tags=29%, list=20%, signal=36% |
| 144 | PHAGOCYTOSIS |  | 16 | -0.34 | -1.13 | 0.286 | 0.570 | 1.000 | 3477 | tags=50%, list=27%, signal=68% |
| 145 | SODIUM\_ION\_TRANSPORT |  | 17 | -0.33 | -1.13 | 0.300 | 0.567 | 1.000 | 5225 | tags=59%, list=40%, signal=98% |
| 146 | NEGATIVE\_REGULATION\_OF\_TRANSCRIPTION\_DNA\_DEPENDENT |  | 114 | -0.21 | -1.12 | 0.236 | 0.565 | 1.000 | 2914 | tags=30%, list=22%, signal=38% |
| 147 | NEURON\_DIFFERENTIATION |  | 58 | -0.24 | -1.12 | 0.233 | 0.562 | 1.000 | 3494 | tags=33%, list=27%, signal=44% |
| 148 | NEGATIVE\_REGULATION\_OF\_NUCLEOBASENUCLEOSIDENUCLEOTIDE\_AND\_NUCLEIC\_ACID\_METABOLIC\_PROCESS |  | 185 | -0.19 | -1.12 | 0.190 | 0.564 | 1.000 | 2718 | tags=28%, list=21%, signal=34% |
| 149 | CYTOKINE\_BIOSYNTHETIC\_PROCESS |  | 34 | -0.27 | -1.12 | 0.277 | 0.565 | 1.000 | 2931 | tags=35%, list=22%, signal=45% |
| 150 | FATTY\_ACID\_OXIDATION |  | 17 | -0.33 | -1.12 | 0.282 | 0.564 | 1.000 | 3385 | tags=47%, list=26%, signal=63% |
| 151 | ORGANIC\_ACID\_METABOLIC\_PROCESS |  | 162 | -0.19 | -1.12 | 0.212 | 0.563 | 1.000 | 2665 | tags=27%, list=20%, signal=33% |
| 152 | POST\_TRANSLATIONAL\_PROTEIN\_MODIFICATION |  | 409 | -0.17 | -1.12 | 0.127 | 0.559 | 1.000 | 3072 | tags=27%, list=23%, signal=34% |
| 153 | MEMBRANE\_ORGANIZATION\_AND\_BIOGENESIS |  | 124 | -0.20 | -1.12 | 0.224 | 0.556 | 1.000 | 3589 | tags=35%, list=27%, signal=48% |
| 154 | RESPONSE\_TO\_BIOTIC\_STIMULUS |  | 103 | -0.21 | -1.12 | 0.248 | 0.554 | 1.000 | 2894 | tags=32%, list=22%, signal=41% |
| 155 | CARBOXYLIC\_ACID\_METABOLIC\_PROCESS |  | 160 | -0.19 | -1.12 | 0.213 | 0.552 | 1.000 | 2665 | tags=27%, list=20%, signal=33% |
| 156 | POSITIVE\_REGULATION\_OF\_T\_CELL\_ACTIVATION |  | 20 | -0.31 | -1.12 | 0.325 | 0.551 | 1.000 | 4535 | tags=55%, list=35%, signal=84% |
| 157 | NEGATIVE\_REGULATION\_OF\_CELL\_DIFFERENTIATION |  | 24 | -0.29 | -1.11 | 0.311 | 0.564 | 1.000 | 2821 | tags=29%, list=22%, signal=37% |
| 158 | ACTIN\_FILAMENT\_ORGANIZATION |  | 21 | -0.30 | -1.11 | 0.335 | 0.562 | 1.000 | 1647 | tags=29%, list=13%, signal=33% |
| 159 | PROTEIN\_SECRETION |  | 28 | -0.28 | -1.11 | 0.300 | 0.561 | 1.000 | 4166 | tags=46%, list=32%, signal=68% |
| 160 | AMINO\_ACID\_METABOLIC\_PROCESS |  | 73 | -0.22 | -1.11 | 0.256 | 0.559 | 1.000 | 2290 | tags=27%, list=17%, signal=33% |
| 161 | GLYCOPROTEIN\_BIOSYNTHETIC\_PROCESS |  | 67 | -0.23 | -1.11 | 0.260 | 0.557 | 1.000 | 3860 | tags=39%, list=29%, signal=55% |
| 162 | AMINO\_ACID\_DERIVATIVE\_METABOLIC\_PROCESS |  | 23 | -0.29 | -1.10 | 0.299 | 0.558 | 1.000 | 4065 | tags=48%, list=31%, signal=69% |
| 163 | TISSUE\_REMODELING |  | 29 | -0.28 | -1.10 | 0.312 | 0.557 | 1.000 | 2300 | tags=28%, list=18%, signal=33% |
| 164 | REGULATION\_OF\_DEVELOPMENTAL\_PROCESS |  | 387 | -0.17 | -1.10 | 0.171 | 0.555 | 1.000 | 4413 | tags=43%, list=34%, signal=63% |
| 165 | REGULATION\_OF\_PROTEIN\_IMPORT\_INTO\_NUCLEUS |  | 15 | -0.34 | -1.10 | 0.319 | 0.556 | 1.000 | 1427 | tags=27%, list=11%, signal=30% |
| 166 | NEGATIVE\_REGULATION\_OF\_METABOLIC\_PROCESS |  | 232 | -0.18 | -1.10 | 0.213 | 0.554 | 1.000 | 2718 | tags=26%, list=21%, signal=32% |
| 167 | REGULATION\_OF\_MAPKKK\_CASCADE |  | 19 | -0.32 | -1.10 | 0.330 | 0.554 | 1.000 | 736 | tags=21%, list=6%, signal=22% |
| 168 | CYTOKINE\_SECRETION |  | 15 | -0.34 | -1.10 | 0.333 | 0.559 | 1.000 | 3414 | tags=47%, list=26%, signal=63% |
| 169 | I\_KAPPAB\_KINASE\_NF\_KAPPAB\_CASCADE |  | 88 | -0.21 | -1.09 | 0.267 | 0.563 | 1.000 | 3816 | tags=42%, list=29%, signal=59% |
| 170 | POSITIVE\_REGULATION\_OF\_TRANSCRIPTION |  | 124 | -0.20 | -1.09 | 0.265 | 0.569 | 1.000 | 3241 | tags=31%, list=25%, signal=40% |
| 171 | TISSUE\_DEVELOPMENT |  | 126 | -0.20 | -1.09 | 0.274 | 0.569 | 1.000 | 1565 | tags=19%, list=12%, signal=21% |
| 172 | CELLULAR\_LIPID\_METABOLIC\_PROCESS |  | 220 | -0.18 | -1.09 | 0.226 | 0.566 | 1.000 | 3944 | tags=38%, list=30%, signal=54% |
| 173 | MEMBRANE\_LIPID\_METABOLIC\_PROCESS |  | 85 | -0.21 | -1.09 | 0.292 | 0.567 | 1.000 | 3364 | tags=35%, list=26%, signal=47% |
| 174 | ORGANIC\_ACID\_TRANSPORT |  | 39 | -0.25 | -1.09 | 0.300 | 0.567 | 1.000 | 2021 | tags=26%, list=15%, signal=30% |
| 175 | CARBOXYLIC\_ACID\_TRANSPORT |  | 39 | -0.25 | -1.08 | 0.327 | 0.576 | 1.000 | 2021 | tags=26%, list=15%, signal=30% |
| 176 | REGULATION\_OF\_BIOLOGICAL\_QUALITY |  | 364 | -0.17 | -1.08 | 0.223 | 0.573 | 1.000 | 3693 | tags=31%, list=28%, signal=42% |
| 177 | POSITIVE\_REGULATION\_OF\_MAP\_KINASE\_ACTIVITY |  | 39 | -0.26 | -1.08 | 0.327 | 0.576 | 1.000 | 2061 | tags=28%, list=16%, signal=33% |
| 178 | NEGATIVE\_REGULATION\_OF\_TRANSCRIPTION\_FROM\_RNA\_POLYMERASE\_II\_PROMOTER |  | 76 | -0.22 | -1.08 | 0.306 | 0.572 | 1.000 | 2914 | tags=30%, list=22%, signal=39% |
| 179 | MYELOID\_CELL\_DIFFERENTIATION |  | 35 | -0.26 | -1.08 | 0.335 | 0.574 | 1.000 | 3006 | tags=31%, list=23%, signal=41% |
| 180 | NEGATIVE\_REGULATION\_OF\_CELL\_PROLIFERATION |  | 145 | -0.19 | -1.07 | 0.289 | 0.584 | 1.000 | 2710 | tags=26%, list=21%, signal=33% |
| 181 | DETECTION\_OF\_EXTERNAL\_STIMULUS |  | 18 | -0.32 | -1.07 | 0.361 | 0.588 | 1.000 | 8968 | tags=100%, list=69%, signal=317% |
| 182 | CELL\_MATURATION |  | 16 | -0.32 | -1.07 | 0.372 | 0.586 | 1.000 | 3234 | tags=38%, list=25%, signal=50% |
| 183 | NEGATIVE\_REGULATION\_OF\_CELLULAR\_METABOLIC\_PROCESS |  | 229 | -0.17 | -1.06 | 0.276 | 0.603 | 1.000 | 2718 | tags=26%, list=21%, signal=32% |
| 184 | ION\_HOMEOSTASIS |  | 112 | -0.20 | -1.06 | 0.323 | 0.601 | 1.000 | 4008 | tags=39%, list=31%, signal=56% |
| 185 | ELECTRON\_TRANSPORT\_GO\_0006118 |  | 50 | -0.23 | -1.06 | 0.347 | 0.599 | 1.000 | 2254 | tags=26%, list=17%, signal=31% |
| 186 | CYTOKINE\_METABOLIC\_PROCESS |  | 35 | -0.26 | -1.06 | 0.367 | 0.597 | 1.000 | 2931 | tags=34%, list=22%, signal=44% |
| 187 | CELL\_MIGRATION |  | 82 | -0.21 | -1.06 | 0.352 | 0.595 | 1.000 | 2743 | tags=26%, list=21%, signal=32% |
| 188 | NEURON\_DEVELOPMENT |  | 49 | -0.23 | -1.06 | 0.350 | 0.599 | 1.000 | 3494 | tags=33%, list=27%, signal=44% |
| 189 | INSULIN\_RECEPTOR\_SIGNALING\_PATHWAY |  | 16 | -0.31 | -1.06 | 0.345 | 0.596 | 1.000 | 3315 | tags=44%, list=25%, signal=59% |
| 190 | POSITIVE\_REGULATION\_OF\_DEVELOPMENTAL\_PROCESS |  | 197 | -0.18 | -1.06 | 0.332 | 0.594 | 1.000 | 4244 | tags=44%, list=32%, signal=64% |
| 191 | HORMONE\_METABOLIC\_PROCESS |  | 29 | -0.26 | -1.05 | 0.358 | 0.615 | 1.000 | 3933 | tags=48%, list=30%, signal=69% |
| 192 | LOCOMOTORY\_BEHAVIOR |  | 84 | -0.21 | -1.04 | 0.380 | 0.640 | 1.000 | 2778 | tags=29%, list=21%, signal=36% |
| 193 | MYOBLAST\_DIFFERENTIATION |  | 16 | -0.31 | -1.04 | 0.394 | 0.639 | 1.000 | 3472 | tags=50%, list=27%, signal=68% |
| 194 | POSITIVE\_REGULATION\_OF\_CELLULAR\_METABOLIC\_PROCESS |  | 196 | -0.18 | -1.03 | 0.356 | 0.640 | 1.000 | 3241 | tags=30%, list=25%, signal=39% |
| 195 | DETECTION\_OF\_STIMULUS\_INVOLVED\_IN\_SENSORY\_PERCEPTION |  | 15 | -0.32 | -1.03 | 0.420 | 0.639 | 1.000 | 8968 | tags=100%, list=69%, signal=317% |
| 196 | POSITIVE\_REGULATION\_OF\_CATALYTIC\_ACTIVITY |  | 139 | -0.18 | -1.03 | 0.395 | 0.650 | 1.000 | 3795 | tags=33%, list=29%, signal=46% |
| 197 | AMINO\_ACID\_AND\_DERIVATIVE\_METABOLIC\_PROCESS |  | 96 | -0.19 | -1.03 | 0.384 | 0.653 | 1.000 | 2350 | tags=25%, list=18%, signal=30% |
| 198 | POSITIVE\_REGULATION\_OF\_METABOLIC\_PROCESS |  | 201 | -0.17 | -1.03 | 0.404 | 0.650 | 1.000 | 3241 | tags=30%, list=25%, signal=39% |
| 199 | NERVOUS\_SYSTEM\_DEVELOPMENT |  | 328 | -0.16 | -1.02 | 0.385 | 0.653 | 1.000 | 3910 | tags=32%, list=30%, signal=45% |
| 200 | DEFENSE\_RESPONSE\_TO\_BACTERIUM |  | 16 | -0.31 | -1.02 | 0.423 | 0.651 | 1.000 | 1849 | tags=25%, list=14%, signal=29% |
| 201 | CELL\_PROLIFERATION\_GO\_0008283 |  | 466 | -0.15 | -1.02 | 0.397 | 0.656 | 1.000 | 2830 | tags=25%, list=22%, signal=31% |
| 202 | MAPKKK\_CASCADE\_GO\_0000165 |  | 90 | -0.20 | -1.02 | 0.416 | 0.654 | 1.000 | 2166 | tags=22%, list=17%, signal=26% |
| 203 | MACROMOLECULE\_BIOSYNTHETIC\_PROCESS |  | 267 | -0.17 | -1.02 | 0.416 | 0.652 | 1.000 | 3888 | tags=35%, list=30%, signal=49% |
| 204 | AXONOGENESIS |  | 33 | -0.25 | -1.02 | 0.443 | 0.655 | 1.000 | 4371 | tags=42%, list=33%, signal=64% |
| 205 | TRANSLATION |  | 149 | -0.18 | -1.01 | 0.469 | 0.669 | 1.000 | 2931 | tags=29%, list=22%, signal=37% |
| 206 | HEART\_DEVELOPMENT |  | 33 | -0.25 | -1.01 | 0.429 | 0.666 | 1.000 | 4153 | tags=39%, list=32%, signal=58% |
| 207 | AXON\_GUIDANCE |  | 18 | -0.30 | -1.01 | 0.424 | 0.665 | 1.000 | 2743 | tags=33%, list=21%, signal=42% |
| 208 | NEURITE\_DEVELOPMENT |  | 41 | -0.23 | -1.01 | 0.434 | 0.669 | 1.000 | 3494 | tags=32%, list=27%, signal=43% |
| 209 | CELL\_CELL\_ADHESION |  | 72 | -0.20 | -1.00 | 0.455 | 0.691 | 1.000 | 4328 | tags=43%, list=33%, signal=64% |
| 210 | REGULATION\_OF\_TRANSCRIPTION |  | 498 | -0.15 | -1.00 | 0.443 | 0.690 | 1.000 | 3279 | tags=29%, list=25%, signal=37% |
| 211 | POSITIVE\_REGULATION\_OF\_NUCLEOBASENUCLEOSIDENUCLEOTIDE\_AND\_NUCLEIC\_ACID\_METABOLIC\_PROCESS |  | 134 | -0.18 | -0.99 | 0.467 | 0.694 | 1.000 | 3241 | tags=30%, list=25%, signal=39% |
| 212 | REGULATION\_OF\_JNK\_ACTIVITY |  | 18 | -0.29 | -0.99 | 0.454 | 0.691 | 1.000 | 2061 | tags=28%, list=16%, signal=33% |
| 213 | POSITIVE\_REGULATION\_OF\_TRANSCRIPTION\_FROM\_RNA\_POLYMERASE\_II\_PROMOTER |  | 60 | -0.21 | -0.99 | 0.462 | 0.690 | 1.000 | 3205 | tags=32%, list=24%, signal=42% |
| 214 | RHYTHMIC\_PROCESS |  | 23 | -0.26 | -0.99 | 0.480 | 0.699 | 1.000 | 2660 | tags=30%, list=20%, signal=38% |
| 215 | REGULATION\_OF\_PROTEIN\_SECRETION |  | 19 | -0.29 | -0.99 | 0.476 | 0.700 | 1.000 | 4034 | tags=47%, list=31%, signal=68% |
| 216 | ANATOMICAL\_STRUCTURE\_MORPHOGENESIS |  | 336 | -0.15 | -0.98 | 0.510 | 0.707 | 1.000 | 3507 | tags=30%, list=27%, signal=40% |
| 217 | EPIDERMIS\_DEVELOPMENT |  | 66 | -0.20 | -0.98 | 0.481 | 0.706 | 1.000 | 1466 | tags=20%, list=11%, signal=22% |
| 218 | PEPTIDYL\_AMINO\_ACID\_MODIFICATION |  | 47 | -0.22 | -0.98 | 0.469 | 0.703 | 1.000 | 2682 | tags=28%, list=20%, signal=35% |
| 219 | POSITIVE\_REGULATION\_OF\_DNA\_BINDING |  | 18 | -0.29 | -0.98 | 0.470 | 0.705 | 1.000 | 4368 | tags=56%, list=33%, signal=83% |
| 220 | NEUROGENESIS |  | 75 | -0.19 | -0.97 | 0.514 | 0.719 | 1.000 | 3494 | tags=32%, list=27%, signal=43% |
| 221 | ANTI\_APOPTOSIS |  | 107 | -0.18 | -0.97 | 0.519 | 0.730 | 1.000 | 2503 | tags=27%, list=19%, signal=33% |
| 222 | REGULATION\_OF\_CYTOKINE\_BIOSYNTHETIC\_PROCESS |  | 31 | -0.24 | -0.97 | 0.486 | 0.727 | 1.000 | 2931 | tags=32%, list=22%, signal=41% |
| 223 | RESPONSE\_TO\_CHEMICAL\_STIMULUS |  | 271 | -0.15 | -0.96 | 0.540 | 0.749 | 1.000 | 2778 | tags=24%, list=21%, signal=30% |
| 224 | CELLULAR\_COMPONENT\_ASSEMBLY |  | 272 | -0.16 | -0.96 | 0.564 | 0.746 | 1.000 | 3022 | tags=27%, list=23%, signal=34% |
| 225 | NEGATIVE\_REGULATION\_OF\_DEVELOPMENTAL\_PROCESS |  | 177 | -0.17 | -0.96 | 0.547 | 0.751 | 1.000 | 3404 | tags=32%, list=26%, signal=42% |
| 226 | REGULATION\_OF\_G\_PROTEIN\_COUPLED\_RECEPTOR\_PROTEIN\_SIGNALING\_PATHWAY |  | 23 | -0.26 | -0.94 | 0.530 | 0.779 | 1.000 | 1052 | tags=17%, list=8%, signal=19% |
| 227 | RESPONSE\_TO\_OXIDATIVE\_STRESS |  | 38 | -0.22 | -0.94 | 0.549 | 0.776 | 1.000 | 1429 | tags=24%, list=11%, signal=27% |
| 228 | REGULATION\_OF\_MUSCLE\_CONTRACTION |  | 18 | -0.28 | -0.94 | 0.502 | 0.775 | 1.000 | 2873 | tags=39%, list=22%, signal=50% |
| 229 | POSITIVE\_REGULATION\_OF\_TRANSCRIPTION\_FACTOR\_ACTIVITY |  | 17 | -0.28 | -0.94 | 0.533 | 0.774 | 1.000 | 4294 | tags=53%, list=33%, signal=79% |
| 230 | CYCLIC\_NUCLEOTIDE\_MEDIATED\_SIGNALING |  | 97 | -0.18 | -0.94 | 0.571 | 0.777 | 1.000 | 1318 | tags=12%, list=10%, signal=14% |
| 231 | CELLULAR\_HOMEOSTASIS |  | 121 | -0.17 | -0.94 | 0.595 | 0.778 | 1.000 | 3289 | tags=31%, list=25%, signal=40% |
| 232 | MACROMOLECULAR\_COMPLEX\_ASSEMBLY |  | 254 | -0.15 | -0.92 | 0.693 | 0.820 | 1.000 | 3022 | tags=26%, list=23%, signal=34% |
| 233 | HOMEOSTATIC\_PROCESS |  | 179 | -0.16 | -0.92 | 0.679 | 0.821 | 1.000 | 3289 | tags=28%, list=25%, signal=38% |
| 234 | PROTEIN\_HOMOOLIGOMERIZATION |  | 19 | -0.27 | -0.92 | 0.537 | 0.819 | 1.000 | 1980 | tags=26%, list=15%, signal=31% |
| 235 | REGULATION\_OF\_TRANSLATIONAL\_INITIATION |  | 25 | -0.24 | -0.92 | 0.577 | 0.816 | 1.000 | 2652 | tags=32%, list=20%, signal=40% |
| 236 | G\_PROTEIN\_SIGNALING\_COUPLED\_TO\_CYCLIC\_NUCLEOTIDE\_SECOND\_MESSENGER |  | 96 | -0.18 | -0.92 | 0.653 | 0.815 | 1.000 | 1318 | tags=13%, list=10%, signal=14% |
| 237 | SKELETAL\_MUSCLE\_DEVELOPMENT |  | 28 | -0.23 | -0.92 | 0.606 | 0.813 | 1.000 | 3660 | tags=43%, list=28%, signal=59% |
| 238 | SECRETION\_BY\_CELL |  | 100 | -0.17 | -0.92 | 0.647 | 0.813 | 1.000 | 4549 | tags=43%, list=35%, signal=65% |
| 239 | ECTODERM\_DEVELOPMENT |  | 75 | -0.18 | -0.91 | 0.615 | 0.815 | 1.000 | 1565 | tags=19%, list=12%, signal=21% |
| 240 | REGULATION\_OF\_GROWTH |  | 48 | -0.21 | -0.91 | 0.589 | 0.823 | 1.000 | 3925 | tags=40%, list=30%, signal=56% |
| 241 | RESPONSE\_TO\_NUTRIENT |  | 17 | -0.27 | -0.90 | 0.578 | 0.832 | 1.000 | 401 | tags=18%, list=3%, signal=18% |
| 242 | CHEMICAL\_HOMEOSTASIS |  | 136 | -0.16 | -0.90 | 0.671 | 0.835 | 1.000 | 3289 | tags=29%, list=25%, signal=38% |
| 243 | NEGATIVE\_REGULATION\_OF\_CELLULAR\_COMPONENT\_ORGANIZATION\_AND\_BIOGENESIS |  | 26 | -0.24 | -0.90 | 0.612 | 0.832 | 1.000 | 1647 | tags=19%, list=13%, signal=22% |
| 244 | AMINE\_METABOLIC\_PROCESS |  | 128 | -0.16 | -0.90 | 0.683 | 0.836 | 1.000 | 4125 | tags=38%, list=32%, signal=54% |
| 245 | REGULATION\_OF\_CELLULAR\_COMPONENT\_ORGANIZATION\_AND\_BIOGENESIS |  | 102 | -0.17 | -0.90 | 0.711 | 0.838 | 1.000 | 3660 | tags=33%, list=28%, signal=46% |
| 246 | CELLULAR\_PROTEIN\_COMPLEX\_ASSEMBLY |  | 28 | -0.23 | -0.89 | 0.619 | 0.844 | 1.000 | 2811 | tags=29%, list=21%, signal=36% |
| 247 | PHOSPHOINOSITIDE\_METABOLIC\_PROCESS |  | 25 | -0.24 | -0.89 | 0.633 | 0.859 | 1.000 | 3888 | tags=44%, list=30%, signal=62% |
| 248 | SECOND\_MESSENGER\_MEDIATED\_SIGNALING |  | 139 | -0.16 | -0.88 | 0.777 | 0.872 | 1.000 | 1318 | tags=12%, list=10%, signal=13% |
| 249 | REGULATION\_OF\_TRANSLATION |  | 76 | -0.18 | -0.87 | 0.736 | 0.880 | 1.000 | 2705 | tags=26%, list=21%, signal=33% |
| 250 | SPHINGOLIPID\_METABOLIC\_PROCESS |  | 23 | -0.24 | -0.87 | 0.658 | 0.881 | 1.000 | 3247 | tags=35%, list=25%, signal=46% |
| 251 | ENDOSOME\_TRANSPORT |  | 22 | -0.24 | -0.87 | 0.638 | 0.881 | 1.000 | 2338 | tags=27%, list=18%, signal=33% |
| 252 | CELL\_CELL\_SIGNALING |  | 372 | -0.13 | -0.86 | 0.892 | 0.896 | 1.000 | 4138 | tags=32%, list=32%, signal=46% |
| 253 | VESICLE\_MEDIATED\_TRANSPORT |  | 174 | -0.15 | -0.86 | 0.831 | 0.893 | 1.000 | 1914 | tags=18%, list=15%, signal=21% |
| 254 | POSITIVE\_REGULATION\_OF\_CELLULAR\_COMPONENT\_ORGANIZATION\_AND\_BIOGENESIS |  | 28 | -0.21 | -0.84 | 0.715 | 0.941 | 1.000 | 3460 | tags=36%, list=26%, signal=48% |
| 255 | NEGATIVE\_REGULATION\_OF\_GROWTH |  | 35 | -0.20 | -0.84 | 0.725 | 0.937 | 1.000 | 4770 | tags=51%, list=36%, signal=81% |
| 256 | EXTRACELLULAR\_STRUCTURE\_ORGANIZATION\_AND\_BIOGENESIS |  | 23 | -0.22 | -0.83 | 0.698 | 0.955 | 1.000 | 2056 | tags=26%, list=16%, signal=31% |
| 257 | REGULATION\_OF\_PROTEIN\_MODIFICATION\_PROCESS |  | 37 | -0.20 | -0.83 | 0.734 | 0.957 | 1.000 | 1592 | tags=19%, list=12%, signal=21% |
| 258 | ACTIVATION\_OF\_PROTEIN\_KINASE\_ACTIVITY |  | 23 | -0.22 | -0.82 | 0.687 | 0.954 | 1.000 | 4602 | tags=39%, list=35%, signal=60% |
| 259 | EXCRETION |  | 35 | -0.20 | -0.82 | 0.765 | 0.962 | 1.000 | 2048 | tags=20%, list=16%, signal=24% |
| 260 | REGULATION\_OF\_BINDING |  | 46 | -0.18 | -0.82 | 0.742 | 0.959 | 1.000 | 1882 | tags=22%, list=14%, signal=25% |
| 261 | CELLULAR\_MORPHOGENESIS\_DURING\_DIFFERENTIATION |  | 38 | -0.19 | -0.82 | 0.755 | 0.955 | 1.000 | 3494 | tags=29%, list=27%, signal=39% |
| 262 | POSITIVE\_REGULATION\_OF\_BINDING |  | 19 | -0.24 | -0.82 | 0.702 | 0.953 | 1.000 | 4368 | tags=53%, list=33%, signal=79% |
| 263 | CENTRAL\_NERVOUS\_SYSTEM\_DEVELOPMENT |  | 105 | -0.15 | -0.80 | 0.891 | 0.974 | 1.000 | 3662 | tags=30%, list=28%, signal=42% |
| 264 | NITROGEN\_COMPOUND\_METABOLIC\_PROCESS |  | 141 | -0.14 | -0.80 | 0.904 | 0.976 | 1.000 | 4125 | tags=35%, list=32%, signal=51% |
| 265 | PROTEIN\_LOCALIZATION |  | 184 | -0.14 | -0.80 | 0.947 | 0.978 | 1.000 | 3559 | tags=29%, list=27%, signal=39% |
| 266 | RESPONSE\_TO\_NUTRIENT\_LEVELS |  | 27 | -0.20 | -0.80 | 0.783 | 0.975 | 1.000 | 2491 | tags=26%, list=19%, signal=32% |
| 267 | SULFUR\_METABOLIC\_PROCESS |  | 30 | -0.20 | -0.79 | 0.782 | 0.979 | 1.000 | 3326 | tags=33%, list=25%, signal=45% |
| 268 | REGULATION\_OF\_CELL\_MIGRATION |  | 23 | -0.22 | -0.79 | 0.774 | 0.979 | 1.000 | 4371 | tags=39%, list=33%, signal=59% |
| 269 | POSITIVE\_REGULATION\_OF\_JNK\_ACTIVITY |  | 16 | -0.23 | -0.79 | 0.754 | 0.982 | 1.000 | 2061 | tags=25%, list=16%, signal=30% |
| 270 | POSITIVE\_REGULATION\_OF\_TRANSCRIPTIONDNA\_DEPENDENT |  | 105 | -0.14 | -0.78 | 0.931 | 0.988 | 1.000 | 3210 | tags=27%, list=25%, signal=35% |
| 271 | G\_PROTEIN\_SIGNALING\_COUPLED\_TO\_IP3\_SECOND\_MESSENGERPHOSPHOLIPASE\_C\_ACTIVATING |  | 39 | -0.19 | -0.78 | 0.849 | 0.985 | 1.000 | 3022 | tags=26%, list=23%, signal=33% |
| 272 | ESTABLISHMENT\_AND\_OR\_MAINTENANCE\_OF\_CELL\_POLARITY |  | 19 | -0.22 | -0.78 | 0.790 | 0.982 | 1.000 | 3592 | tags=32%, list=27%, signal=43% |
| 273 | PATTERN\_SPECIFICATION\_PROCESS |  | 27 | -0.19 | -0.78 | 0.813 | 0.982 | 1.000 | 5143 | tags=52%, list=39%, signal=85% |
| 274 | REPRODUCTIVE\_PROCESS |  | 133 | -0.14 | -0.77 | 0.935 | 0.983 | 1.000 | 3670 | tags=31%, list=28%, signal=42% |
| 275 | G\_PROTEIN\_COUPLED\_RECEPTOR\_PROTEIN\_SIGNALING\_PATHWAY |  | 300 | -0.12 | -0.77 | 0.992 | 0.987 | 1.000 | 4094 | tags=29%, list=31%, signal=41% |
| 276 | T\_CELL\_PROLIFERATION |  | 17 | -0.23 | -0.76 | 0.791 | 0.995 | 1.000 | 4815 | tags=59%, list=37%, signal=93% |
| 277 | POSITIVE\_REGULATION\_OF\_CASPASE\_ACTIVITY |  | 28 | -0.19 | -0.76 | 0.824 | 0.998 | 1.000 | 1976 | tags=25%, list=15%, signal=29% |
| 278 | AMINE\_BIOSYNTHETIC\_PROCESS |  | 15 | -0.23 | -0.76 | 0.781 | 0.995 | 1.000 | 527 | tags=13%, list=4%, signal=14% |
| 279 | TRANSLATIONAL\_INITIATION |  | 33 | -0.18 | -0.75 | 0.853 | 1.000 | 1.000 | 1277 | tags=18%, list=10%, signal=20% |
| 280 | POSITIVE\_REGULATION\_OF\_RNA\_METABOLIC\_PROCESS |  | 107 | -0.14 | -0.74 | 0.955 | 1.000 | 1.000 | 3210 | tags=26%, list=25%, signal=34% |
| 281 | REGULATION\_OF\_DNA\_BINDING |  | 36 | -0.17 | -0.74 | 0.873 | 1.000 | 1.000 | 3034 | tags=31%, list=23%, signal=40% |
| 282 | REGULATION\_OF\_TRANSCRIPTION\_FACTOR\_ACTIVITY |  | 30 | -0.18 | -0.73 | 0.870 | 1.000 | 1.000 | 3991 | tags=40%, list=30%, signal=57% |
| 283 | DI\_\_\_TRI\_VALENT\_INORGANIC\_CATION\_TRANSPORT |  | 27 | -0.18 | -0.73 | 0.878 | 1.000 | 1.000 | 1114 | tags=15%, list=9%, signal=16% |
| 284 | GOLGI\_VESICLE\_TRANSPORT |  | 42 | -0.17 | -0.73 | 0.882 | 1.000 | 1.000 | 4493 | tags=43%, list=34%, signal=65% |
| 285 | LIPID\_HOMEOSTASIS |  | 15 | -0.22 | -0.73 | 0.824 | 1.000 | 1.000 | 561 | tags=13%, list=4%, signal=14% |
| 286 | REGULATION\_OF\_SECRETION |  | 35 | -0.18 | -0.72 | 0.879 | 1.000 | 1.000 | 4436 | tags=46%, list=34%, signal=69% |
| 287 | RESPONSE\_TO\_EXTRACELLULAR\_STIMULUS |  | 29 | -0.18 | -0.72 | 0.900 | 1.000 | 1.000 | 2491 | tags=24%, list=19%, signal=30% |
| 288 | CARBOHYDRATE\_METABOLIC\_PROCESS |  | 152 | -0.13 | -0.71 | 0.989 | 1.000 | 1.000 | 3882 | tags=31%, list=30%, signal=43% |
| 289 | PROTEIN\_POLYMERIZATION |  | 17 | -0.21 | -0.71 | 0.860 | 1.000 | 1.000 | 220 | tags=12%, list=2%, signal=12% |
| 290 | METAL\_ION\_TRANSPORT |  | 102 | -0.13 | -0.70 | 0.978 | 1.000 | 1.000 | 5346 | tags=46%, list=41%, signal=77% |
| 291 | PROTEIN\_AMINO\_ACID\_LIPIDATION |  | 21 | -0.19 | -0.70 | 0.881 | 1.000 | 1.000 | 4633 | tags=52%, list=35%, signal=81% |
| 292 | G\_PROTEIN\_SIGNALING\_ADENYLATE\_CYCLASE\_ACTIVATING\_PATHWAY |  | 24 | -0.18 | -0.70 | 0.881 | 1.000 | 1.000 | 1318 | tags=13%, list=10%, signal=14% |
| 293 | SECRETION |  | 157 | -0.12 | -0.70 | 0.993 | 1.000 | 1.000 | 4597 | tags=38%, list=35%, signal=58% |
| 294 | NEGATIVE\_REGULATION\_OF\_CELLULAR\_PROTEIN\_METABOLIC\_PROCESS |  | 41 | -0.16 | -0.69 | 0.948 | 1.000 | 1.000 | 3207 | tags=27%, list=24%, signal=35% |
| 295 | INORGANIC\_ANION\_TRANSPORT |  | 16 | -0.21 | -0.69 | 0.872 | 1.000 | 1.000 | 603 | tags=13%, list=5%, signal=13% |
| 296 | NUCLEOTIDE\_EXCISION\_REPAIR |  | 19 | -0.20 | -0.69 | 0.911 | 1.000 | 1.000 | 1981 | tags=21%, list=15%, signal=25% |
| 297 | PHOSPHOINOSITIDE\_MEDIATED\_SIGNALING |  | 42 | -0.16 | -0.67 | 0.933 | 1.000 | 1.000 | 3022 | tags=24%, list=23%, signal=31% |
| 298 | CATION\_TRANSPORT |  | 130 | -0.12 | -0.67 | 0.993 | 1.000 | 1.000 | 4127 | tags=31%, list=32%, signal=44% |
| 299 | SECRETORY\_PATHWAY |  | 72 | -0.14 | -0.67 | 0.980 | 1.000 | 1.000 | 4493 | tags=40%, list=34%, signal=61% |
| 300 | REGULATION\_OF\_CELL\_GROWTH |  | 39 | -0.15 | -0.66 | 0.956 | 1.000 | 1.000 | 4770 | tags=44%, list=36%, signal=68% |
| 301 | ION\_TRANSPORT |  | 165 | -0.11 | -0.66 | 1.000 | 1.000 | 1.000 | 4127 | tags=30%, list=32%, signal=44% |
| 302 | CARBOHYDRATE\_BIOSYNTHETIC\_PROCESS |  | 35 | -0.16 | -0.66 | 0.932 | 1.000 | 1.000 | 5329 | tags=54%, list=41%, signal=91% |
| 303 | RHO\_PROTEIN\_SIGNAL\_TRANSDUCTION |  | 30 | -0.17 | -0.66 | 0.928 | 1.000 | 1.000 | 3688 | tags=33%, list=28%, signal=46% |
| 304 | POTASSIUM\_ION\_TRANSPORT |  | 52 | -0.14 | -0.65 | 0.968 | 1.000 | 1.000 | 5521 | tags=50%, list=42%, signal=86% |
| 305 | NEGATIVE\_REGULATION\_OF\_PROTEIN\_METABOLIC\_PROCESS |  | 44 | -0.14 | -0.65 | 0.965 | 1.000 | 1.000 | 3207 | tags=25%, list=24%, signal=33% |
| 306 | POSITIVE\_REGULATION\_OF\_TRANSPORT |  | 18 | -0.19 | -0.64 | 0.912 | 0.999 | 1.000 | 5266 | tags=61%, list=40%, signal=102% |
| 307 | NEGATIVE\_REGULATION\_OF\_MULTICELLULAR\_ORGANISMAL\_PROCESS |  | 27 | -0.16 | -0.63 | 0.937 | 1.000 | 1.000 | 2282 | tags=22%, list=17%, signal=27% |
| 308 | REGULATION\_OF\_CYTOKINE\_PRODUCTION |  | 21 | -0.17 | -0.62 | 0.936 | 1.000 | 1.000 | 2444 | tags=24%, list=19%, signal=29% |
| 309 | CALCIUM\_ION\_TRANSPORT |  | 23 | -0.16 | -0.59 | 0.958 | 1.000 | 1.000 | 1114 | tags=13%, list=9%, signal=14% |
| 310 | FEMALE\_GAMETE\_GENERATION |  | 15 | -0.18 | -0.58 | 0.968 | 1.000 | 1.000 | 10731 | tags=100%, list=82%, signal=554% |
| 311 | REGULATION\_OF\_HEART\_CONTRACTION |  | 24 | -0.16 | -0.58 | 0.981 | 1.000 | 1.000 | 11059 | tags=100%, list=84%, signal=643% |
| 312 | PEROXISOME\_ORGANIZATION\_AND\_BIOGENESIS |  | 15 | -0.18 | -0.56 | 0.970 | 1.000 | 1.000 | 4732 | tags=47%, list=36%, signal=73% |
| 313 | CARBOHYDRATE\_CATABOLIC\_PROCESS |  | 20 | -0.16 | -0.55 | 0.981 | 1.000 | 1.000 | 4479 | tags=35%, list=34%, signal=53% |
| 314 | CELLULAR\_CARBOHYDRATE\_CATABOLIC\_PROCESS |  | 20 | -0.16 | -0.55 | 0.974 | 1.000 | 1.000 | 4479 | tags=35%, list=34%, signal=53% |
| 315 | AMINO\_SUGAR\_METABOLIC\_PROCESS |  | 15 | -0.16 | -0.53 | 0.987 | 1.000 | 1.000 | 5232 | tags=53%, list=40%, signal=89% |
| 316 | REGULATION\_OF\_ACTION\_POTENTIAL |  | 16 | -0.16 | -0.53 | 0.986 | 1.000 | 1.000 | 3586 | tags=31%, list=27%, signal=43% |
| 317 | RESPONSE\_TO\_LIGHT\_STIMULUS |  | 40 | -0.12 | -0.51 | 0.993 | 0.999 | 1.000 | 2705 | tags=20%, list=21%, signal=25% |
| 318 | MONOVALENT\_INORGANIC\_CATION\_TRANSPORT |  | 83 | -0.09 | -0.46 | 1.000 | 0.999 | 1.000 | 5521 | tags=46%, list=42%, signal=79% |
Table: Gene sets enriched in phenotype **na**[plain text format]****

  
