# Supplementary material for: Multiple-input multiple-output causal strategies for gene selection
Source: BMC Bioinformatics. 2011 Nov 25;12:458. doi: 10.1186/1471-2105-12-458 (PMC3323860; doi:10.1186/1471-2105-12-458)
Supplement: Additional file 2 — Archive containing the output files computed by the preranked GSEA for λ ∈ {0.1,0.2,0.3,0.4,0.5} (GSEA_MIMO_part1.zip). [file 1471-2105-12-458-S2.ZIP › mFS01_entrez_mimo.GseaPreranked.1316037859455/gsea_report_for_na_pos_1316037859455.html]

Report for na\_pos 1316037859455 [GSEA]

| GS  follow link to MSigDB | GS DETAILS | SIZE | ES | NES | NOM p-val | FDR q-val | FWER p-val | RANK AT MAX | LEADING EDGE || 1 | M\_PHASE\_OF\_MITOTIC\_CELL\_CYCLE |  | 72 | 0.60 | 2.82 | 0.000 | 0.000 | 0.000 | 2079 | tags=56%, list=16%, signal=66% |
| 2 | MITOSIS |  | 70 | 0.59 | 2.80 | 0.000 | 0.000 | 0.000 | 2079 | tags=54%, list=16%, signal=64% |
| 3 | M\_PHASE |  | 98 | 0.55 | 2.77 | 0.000 | 0.000 | 0.000 | 2184 | tags=51%, list=17%, signal=61% |
| 4 | MITOTIC\_CELL\_CYCLE |  | 134 | 0.51 | 2.75 | 0.000 | 0.000 | 0.000 | 2729 | tags=52%, list=21%, signal=65% |
| 5 | CELL\_CYCLE\_PROCESS |  | 169 | 0.50 | 2.74 | 0.000 | 0.000 | 0.000 | 2645 | tags=50%, list=20%, signal=61% |
| 6 | CELL\_CYCLE\_PHASE |  | 152 | 0.48 | 2.62 | 0.000 | 0.000 | 0.000 | 2645 | tags=48%, list=20%, signal=59% |
| 7 | DNA\_REPLICATION |  | 97 | 0.50 | 2.54 | 0.000 | 0.000 | 0.000 | 3313 | tags=53%, list=25%, signal=70% |
| 8 | DNA\_METABOLIC\_PROCESS |  | 240 | 0.43 | 2.49 | 0.000 | 0.000 | 0.000 | 3333 | tags=50%, list=25%, signal=65% |
| 9 | SISTER\_CHROMATID\_SEGREGATION |  | 16 | 0.77 | 2.48 | 0.000 | 0.000 | 0.000 | 605 | tags=56%, list=5%, signal=59% |
| 10 | MITOTIC\_SISTER\_CHROMATID\_SEGREGATION |  | 15 | 0.78 | 2.48 | 0.000 | 0.000 | 0.000 | 605 | tags=60%, list=5%, signal=63% |
| 11 | CHROMOSOME\_SEGREGATION |  | 28 | 0.62 | 2.43 | 0.000 | 0.000 | 0.001 | 605 | tags=46%, list=5%, signal=49% |
| 12 | CELL\_CYCLE\_GO\_0007049 |  | 277 | 0.41 | 2.42 | 0.000 | 0.000 | 0.001 | 2645 | tags=43%, list=20%, signal=52% |
| 13 | CELL\_CYCLE\_CHECKPOINT\_GO\_0000075 |  | 45 | 0.54 | 2.36 | 0.000 | 0.000 | 0.001 | 2249 | tags=56%, list=17%, signal=67% |
| 14 | DNA\_DEPENDENT\_DNA\_REPLICATION |  | 52 | 0.52 | 2.33 | 0.000 | 0.000 | 0.001 | 2571 | tags=52%, list=20%, signal=64% |
| 15 | REGULATION\_OF\_MITOSIS |  | 33 | 0.59 | 2.33 | 0.000 | 0.000 | 0.001 | 1848 | tags=52%, list=14%, signal=60% |
| 16 | DNA\_REPAIR |  | 118 | 0.44 | 2.31 | 0.000 | 0.000 | 0.001 | 2483 | tags=45%, list=19%, signal=55% |
| 17 | RESPONSE\_TO\_DNA\_DAMAGE\_STIMULUS |  | 153 | 0.42 | 2.28 | 0.000 | 0.000 | 0.004 | 2612 | tags=44%, list=20%, signal=54% |
| 18 | RNA\_SPLICING |  | 74 | 0.46 | 2.24 | 0.000 | 0.000 | 0.006 | 3583 | tags=55%, list=27%, signal=76% |
| 19 | RESPONSE\_TO\_ENDOGENOUS\_STIMULUS |  | 182 | 0.38 | 2.16 | 0.000 | 0.001 | 0.019 | 3333 | tags=46%, list=25%, signal=61% |
| 20 | RNA\_PROCESSING |  | 138 | 0.40 | 2.15 | 0.000 | 0.001 | 0.025 | 3010 | tags=47%, list=23%, signal=61% |
| 21 | NUCLEOTIDE\_BIOSYNTHETIC\_PROCESS |  | 17 | 0.64 | 2.14 | 0.000 | 0.001 | 0.025 | 1561 | tags=53%, list=12%, signal=60% |
| 22 | MITOTIC\_CELL\_CYCLE\_CHECKPOINT |  | 19 | 0.61 | 2.10 | 0.002 | 0.001 | 0.038 | 1848 | tags=53%, list=14%, signal=61% |
| 23 | MRNA\_METABOLIC\_PROCESS |  | 72 | 0.43 | 2.07 | 0.000 | 0.002 | 0.058 | 2937 | tags=49%, list=22%, signal=62% |
| 24 | DNA\_INTEGRITY\_CHECKPOINT |  | 22 | 0.58 | 2.07 | 0.000 | 0.002 | 0.060 | 1655 | tags=55%, list=13%, signal=62% |
| 25 | MICROTUBULE\_CYTOSKELETON\_ORGANIZATION\_AND\_BIOGENESIS |  | 31 | 0.52 | 2.06 | 0.000 | 0.002 | 0.069 | 2729 | tags=55%, list=21%, signal=69% |
| 26 | REGULATION\_OF\_CELL\_CYCLE |  | 161 | 0.37 | 2.04 | 0.000 | 0.003 | 0.084 | 1848 | tags=35%, list=14%, signal=40% |
| 27 | MITOCHONDRION\_ORGANIZATION\_AND\_BIOGENESIS |  | 42 | 0.47 | 2.03 | 0.000 | 0.003 | 0.103 | 3733 | tags=57%, list=29%, signal=80% |
| 28 | DNA\_REPLICATION\_INITIATION |  | 15 | 0.64 | 2.02 | 0.000 | 0.003 | 0.109 | 2249 | tags=73%, list=17%, signal=88% |
| 29 | NUCLEOBASENUCLEOSIDENUCLEOTIDE\_AND\_NUCLEIC\_ACID\_TRANSPORT |  | 26 | 0.52 | 2.01 | 0.000 | 0.003 | 0.121 | 2289 | tags=50%, list=17%, signal=60% |
| 30 | PROTEIN\_FOLDING |  | 55 | 0.44 | 1.98 | 0.000 | 0.004 | 0.154 | 3289 | tags=51%, list=25%, signal=68% |
| 31 | REGULATION\_OF\_MITOTIC\_CELL\_CYCLE |  | 19 | 0.57 | 1.96 | 0.002 | 0.005 | 0.184 | 1122 | tags=47%, list=9%, signal=52% |
| 32 | MRNA\_PROCESSING\_GO\_0006397 |  | 61 | 0.43 | 1.96 | 0.002 | 0.005 | 0.195 | 2937 | tags=46%, list=22%, signal=59% |
| 33 | DOUBLE\_STRAND\_BREAK\_REPAIR |  | 21 | 0.56 | 1.95 | 0.004 | 0.005 | 0.206 | 2008 | tags=52%, list=15%, signal=62% |
| 34 | COENZYME\_METABOLIC\_PROCESS |  | 35 | 0.48 | 1.94 | 0.002 | 0.006 | 0.237 | 3492 | tags=49%, list=27%, signal=66% |
| 35 | TRNA\_METABOLIC\_PROCESS |  | 15 | 0.61 | 1.93 | 0.004 | 0.006 | 0.237 | 2902 | tags=67%, list=22%, signal=86% |
| 36 | INTERPHASE\_OF\_MITOTIC\_CELL\_CYCLE |  | 57 | 0.42 | 1.92 | 0.000 | 0.007 | 0.267 | 3585 | tags=53%, list=27%, signal=72% |
| 37 | INTERPHASE |  | 63 | 0.41 | 1.90 | 0.000 | 0.008 | 0.327 | 3585 | tags=51%, list=27%, signal=70% |
| 38 | G1\_S\_TRANSITION\_OF\_MITOTIC\_CELL\_CYCLE |  | 23 | 0.51 | 1.89 | 0.000 | 0.009 | 0.372 | 2483 | tags=48%, list=19%, signal=59% |
| 39 | REGULATION\_OF\_CYCLIN\_DEPENDENT\_PROTEIN\_KINASE\_ACTIVITY |  | 40 | 0.45 | 1.89 | 0.002 | 0.009 | 0.380 | 2910 | tags=52%, list=22%, signal=67% |
| 40 | CHROMOSOME\_ORGANIZATION\_AND\_BIOGENESIS |  | 107 | 0.37 | 1.88 | 0.000 | 0.009 | 0.396 | 3249 | tags=43%, list=25%, signal=57% |
| 41 | COFACTOR\_BIOSYNTHETIC\_PROCESS |  | 21 | 0.53 | 1.87 | 0.002 | 0.010 | 0.424 | 1533 | tags=38%, list=12%, signal=43% |
| 42 | REGULATION\_OF\_DNA\_METABOLIC\_PROCESS |  | 40 | 0.44 | 1.85 | 0.000 | 0.011 | 0.469 | 2544 | tags=48%, list=19%, signal=59% |
| 43 | REGULATION\_OF\_DNA\_REPLICATION |  | 18 | 0.53 | 1.81 | 0.011 | 0.016 | 0.593 | 2249 | tags=50%, list=17%, signal=60% |
| 44 | BIOPOLYMER\_CATABOLIC\_PROCESS |  | 103 | 0.35 | 1.80 | 0.000 | 0.017 | 0.632 | 2612 | tags=37%, list=20%, signal=46% |
| 45 | DNA\_DAMAGE\_RESPONSESIGNAL\_TRANSDUCTION |  | 34 | 0.46 | 1.80 | 0.002 | 0.017 | 0.636 | 2249 | tags=47%, list=17%, signal=57% |
| 46 | MITOCHONDRIAL\_TRANSPORT |  | 18 | 0.54 | 1.79 | 0.004 | 0.017 | 0.645 | 1339 | tags=44%, list=10%, signal=49% |
| 47 | DNA\_DAMAGE\_CHECKPOINT |  | 19 | 0.53 | 1.78 | 0.004 | 0.018 | 0.668 | 2249 | tags=53%, list=17%, signal=63% |
| 48 | DNA\_RECOMBINATION |  | 45 | 0.42 | 1.78 | 0.005 | 0.018 | 0.675 | 1314 | tags=33%, list=10%, signal=37% |
| 49 | PROTEIN\_CATABOLIC\_PROCESS |  | 60 | 0.39 | 1.77 | 0.004 | 0.020 | 0.714 | 2495 | tags=35%, list=19%, signal=43% |
| 50 | COFACTOR\_METABOLIC\_PROCESS |  | 51 | 0.39 | 1.73 | 0.004 | 0.026 | 0.809 | 3558 | tags=43%, list=27%, signal=59% |
| 51 | UBIQUITIN\_CYCLE |  | 40 | 0.42 | 1.73 | 0.007 | 0.025 | 0.809 | 2213 | tags=38%, list=17%, signal=45% |
| 52 | NUCLEAR\_EXPORT |  | 26 | 0.47 | 1.73 | 0.007 | 0.025 | 0.811 | 2289 | tags=42%, list=17%, signal=51% |
| 53 | PROTEIN\_MODIFICATION\_BY\_SMALL\_PROTEIN\_CONJUGATION |  | 35 | 0.43 | 1.73 | 0.004 | 0.025 | 0.825 | 2213 | tags=40%, list=17%, signal=48% |
| 54 | MEIOSIS\_I |  | 19 | 0.50 | 1.72 | 0.004 | 0.026 | 0.839 | 1314 | tags=37%, list=10%, signal=41% |
| 55 | MACROMOLECULE\_CATABOLIC\_PROCESS |  | 120 | 0.32 | 1.72 | 0.003 | 0.027 | 0.851 | 2625 | tags=33%, list=20%, signal=41% |
| 56 | DNA\_PACKAGING |  | 29 | 0.44 | 1.71 | 0.013 | 0.027 | 0.864 | 2560 | tags=45%, list=20%, signal=56% |
| 57 | TRANSCRIPTION\_INITIATION\_FROM\_RNA\_POLYMERASE\_II\_PROMOTER |  | 27 | 0.46 | 1.70 | 0.015 | 0.029 | 0.893 | 3233 | tags=48%, list=25%, signal=64% |
| 58 | CELLULAR\_PROTEIN\_CATABOLIC\_PROCESS |  | 50 | 0.39 | 1.68 | 0.011 | 0.034 | 0.928 | 2469 | tags=34%, list=19%, signal=42% |
| 59 | ONE\_CARBON\_COMPOUND\_METABOLIC\_PROCESS |  | 24 | 0.47 | 1.67 | 0.022 | 0.036 | 0.941 | 2154 | tags=46%, list=16%, signal=55% |
| 60 | BASE\_EXCISION\_REPAIR |  | 16 | 0.50 | 1.66 | 0.011 | 0.037 | 0.949 | 2414 | tags=44%, list=18%, signal=54% |
| 61 | PROTEIN\_UBIQUITINATION |  | 32 | 0.41 | 1.65 | 0.014 | 0.040 | 0.962 | 2213 | tags=38%, list=17%, signal=45% |
| 62 | RNA\_EXPORT\_FROM\_NUCLEUS |  | 17 | 0.49 | 1.65 | 0.027 | 0.041 | 0.966 | 2289 | tags=47%, list=17%, signal=57% |
| 63 | MEIOTIC\_CELL\_CYCLE |  | 31 | 0.42 | 1.62 | 0.013 | 0.048 | 0.984 | 2729 | tags=42%, list=21%, signal=53% |
| 64 | CYTOKINESIS |  | 17 | 0.48 | 1.59 | 0.034 | 0.059 | 0.995 | 1088 | tags=35%, list=8%, signal=38% |
| 65 | NUCLEOTIDE\_METABOLIC\_PROCESS |  | 36 | 0.39 | 1.59 | 0.026 | 0.058 | 0.995 | 774 | tags=28%, list=6%, signal=29% |
| 66 | PROTEIN\_DNA\_COMPLEX\_ASSEMBLY |  | 45 | 0.37 | 1.59 | 0.012 | 0.058 | 0.995 | 2796 | tags=40%, list=21%, signal=51% |
| 67 | NUCLEOBASENUCLEOSIDE\_AND\_NUCLEOTIDE\_METABOLIC\_PROCESS |  | 46 | 0.37 | 1.58 | 0.006 | 0.059 | 0.998 | 774 | tags=26%, list=6%, signal=28% |
| 68 | APOPTOTIC\_NUCLEAR\_CHANGES |  | 17 | 0.47 | 1.57 | 0.041 | 0.063 | 0.998 | 2235 | tags=47%, list=17%, signal=57% |
| 69 | CHROMATIN\_ASSEMBLY\_OR\_DISASSEMBLY |  | 25 | 0.42 | 1.57 | 0.023 | 0.063 | 0.998 | 2560 | tags=48%, list=20%, signal=60% |
| 70 | NUCLEAR\_TRANSPORT |  | 77 | 0.32 | 1.56 | 0.007 | 0.067 | 0.998 | 2666 | tags=34%, list=20%, signal=42% |
| 71 | CELLULAR\_COMPONENT\_DISASSEMBLY |  | 31 | 0.40 | 1.56 | 0.027 | 0.066 | 0.999 | 2235 | tags=39%, list=17%, signal=47% |
| 72 | CELLULAR\_MACROMOLECULE\_CATABOLIC\_PROCESS |  | 90 | 0.31 | 1.54 | 0.014 | 0.076 | 0.999 | 2625 | tags=31%, list=20%, signal=39% |
| 73 | NUCLEOCYTOPLASMIC\_TRANSPORT |  | 77 | 0.32 | 1.54 | 0.015 | 0.076 | 0.999 | 2666 | tags=34%, list=20%, signal=42% |
| 74 | ORGANELLE\_ORGANIZATION\_AND\_BIOGENESIS |  | 407 | 0.24 | 1.53 | 0.000 | 0.081 | 1.000 | 3310 | tags=34%, list=25%, signal=44% |
| 75 | TRANSCRIPTION\_INITIATION |  | 33 | 0.37 | 1.47 | 0.047 | 0.112 | 1.000 | 2348 | tags=36%, list=18%, signal=44% |
| 76 | MICROTUBULE\_BASED\_PROCESS |  | 75 | 0.31 | 1.47 | 0.021 | 0.111 | 1.000 | 2988 | tags=37%, list=23%, signal=48% |
| 77 | ESTABLISHMENT\_OF\_ORGANELLE\_LOCALIZATION |  | 16 | 0.46 | 1.47 | 0.061 | 0.110 | 1.000 | 1805 | tags=44%, list=14%, signal=51% |
| 78 | VIRAL\_INFECTIOUS\_CYCLE |  | 29 | 0.38 | 1.46 | 0.054 | 0.116 | 1.000 | 970 | tags=31%, list=7%, signal=33% |
| 79 | CELL\_DIVISION |  | 19 | 0.43 | 1.46 | 0.064 | 0.117 | 1.000 | 1088 | tags=32%, list=8%, signal=34% |
| 80 | NEGATIVE\_REGULATION\_OF\_DNA\_METABOLIC\_PROCESS |  | 16 | 0.44 | 1.44 | 0.075 | 0.132 | 1.000 | 2544 | tags=50%, list=19%, signal=62% |
| 81 | ORGANELLE\_LOCALIZATION |  | 21 | 0.40 | 1.43 | 0.081 | 0.135 | 1.000 | 958 | tags=29%, list=7%, signal=31% |
| 82 | APOPTOTIC\_PROGRAM |  | 56 | 0.31 | 1.41 | 0.036 | 0.154 | 1.000 | 3606 | tags=46%, list=28%, signal=64% |
| 83 | MEIOTIC\_RECOMBINATION |  | 16 | 0.43 | 1.39 | 0.101 | 0.174 | 1.000 | 1314 | tags=31%, list=10%, signal=35% |
| 84 | G1\_PHASE |  | 15 | 0.43 | 1.39 | 0.096 | 0.174 | 1.000 | 387 | tags=27%, list=3%, signal=27% |
| 85 | VIRAL\_REPRODUCTIVE\_PROCESS |  | 33 | 0.35 | 1.38 | 0.095 | 0.175 | 1.000 | 1113 | tags=30%, list=9%, signal=33% |
| 86 | NEGATIVE\_REGULATION\_OF\_BINDING |  | 16 | 0.42 | 1.38 | 0.104 | 0.178 | 1.000 | 2648 | tags=50%, list=20%, signal=63% |
| 87 | CHROMATIN\_REMODELING |  | 21 | 0.39 | 1.37 | 0.096 | 0.183 | 1.000 | 2446 | tags=43%, list=19%, signal=53% |
| 88 | ALCOHOL\_METABOLIC\_PROCESS |  | 82 | 0.28 | 1.37 | 0.061 | 0.185 | 1.000 | 4675 | tags=48%, list=36%, signal=74% |
| 89 | ESTABLISHMENT\_AND\_OR\_MAINTENANCE\_OF\_CHROMATIN\_ARCHITECTURE |  | 65 | 0.29 | 1.37 | 0.062 | 0.187 | 1.000 | 2923 | tags=37%, list=22%, signal=47% |
| 90 | REGULATION\_OF\_GENE\_EXPRESSION\_EPIGENETIC |  | 27 | 0.35 | 1.35 | 0.093 | 0.209 | 1.000 | 2819 | tags=41%, list=22%, signal=52% |
| 91 | DNA\_CATABOLIC\_PROCESS |  | 21 | 0.39 | 1.34 | 0.120 | 0.217 | 1.000 | 3602 | tags=52%, list=28%, signal=72% |
| 92 | INTRACELLULAR\_TRANSPORT |  | 248 | 0.23 | 1.33 | 0.026 | 0.227 | 1.000 | 3349 | tags=33%, list=26%, signal=44% |
| 93 | RESPONSE\_TO\_ABIOTIC\_STIMULUS |  | 79 | 0.27 | 1.32 | 0.075 | 0.230 | 1.000 | 2792 | tags=32%, list=21%, signal=40% |
| 94 | NEGATIVE\_REGULATION\_OF\_CATALYTIC\_ACTIVITY |  | 61 | 0.29 | 1.32 | 0.093 | 0.229 | 1.000 | 2779 | tags=36%, list=21%, signal=46% |
| 95 | RESPONSE\_TO\_HYPOXIA |  | 27 | 0.34 | 1.31 | 0.133 | 0.245 | 1.000 | 2290 | tags=33%, list=17%, signal=40% |
| 96 | VIRAL\_REPRODUCTION |  | 38 | 0.31 | 1.31 | 0.124 | 0.244 | 1.000 | 1113 | tags=26%, list=9%, signal=29% |
| 97 | NUCLEAR\_ORGANIZATION\_AND\_BIOGENESIS |  | 23 | 0.37 | 1.30 | 0.136 | 0.248 | 1.000 | 2235 | tags=39%, list=17%, signal=47% |
| 98 | RNA\_CATABOLIC\_PROCESS |  | 20 | 0.37 | 1.30 | 0.149 | 0.252 | 1.000 | 2289 | tags=45%, list=17%, signal=54% |
| 99 | RESPONSE\_TO\_ORGANIC\_SUBSTANCE |  | 27 | 0.35 | 1.30 | 0.134 | 0.250 | 1.000 | 2909 | tags=37%, list=22%, signal=48% |
| 100 | RESPONSE\_TO\_STRESS |  | 467 | 0.20 | 1.29 | 0.017 | 0.263 | 1.000 | 3333 | tags=32%, list=25%, signal=42% |
| 101 | VIRAL\_GENOME\_REPLICATION |  | 20 | 0.37 | 1.28 | 0.118 | 0.271 | 1.000 | 970 | tags=30%, list=7%, signal=32% |
| 102 | ESTABLISHMENT\_OF\_CELLULAR\_LOCALIZATION |  | 311 | 0.21 | 1.28 | 0.044 | 0.269 | 1.000 | 3349 | tags=31%, list=26%, signal=41% |
| 103 | CHROMATIN\_ASSEMBLY |  | 16 | 0.39 | 1.26 | 0.186 | 0.297 | 1.000 | 2560 | tags=44%, list=20%, signal=54% |
| 104 | OXYGEN\_AND\_REACTIVE\_OXYGEN\_SPECIES\_METABOLIC\_PROCESS |  | 18 | 0.37 | 1.25 | 0.181 | 0.307 | 1.000 | 2801 | tags=44%, list=21%, signal=56% |
| 105 | CELLULAR\_LOCALIZATION |  | 323 | 0.20 | 1.25 | 0.059 | 0.305 | 1.000 | 3349 | tags=31%, list=26%, signal=40% |
| 106 | HETEROCYCLE\_METABOLIC\_PROCESS |  | 26 | 0.34 | 1.24 | 0.176 | 0.317 | 1.000 | 1533 | tags=23%, list=12%, signal=26% |
| 107 | CYTOSKELETON\_DEPENDENT\_INTRACELLULAR\_TRANSPORT |  | 25 | 0.33 | 1.23 | 0.193 | 0.338 | 1.000 | 4289 | tags=56%, list=33%, signal=83% |
| 108 | RIBONUCLEOPROTEIN\_COMPLEX\_BIOGENESIS\_AND\_ASSEMBLY |  | 68 | 0.26 | 1.23 | 0.151 | 0.341 | 1.000 | 3803 | tags=41%, list=29%, signal=58% |
| 109 | CELL\_STRUCTURE\_DISASSEMBLY\_DURING\_APOPTOSIS |  | 17 | 0.37 | 1.22 | 0.216 | 0.339 | 1.000 | 2235 | tags=35%, list=17%, signal=43% |
| 110 | NEGATIVE\_REGULATION\_OF\_DNA\_BINDING |  | 15 | 0.38 | 1.22 | 0.226 | 0.339 | 1.000 | 2648 | tags=47%, list=20%, signal=58% |
| 111 | TRANSCRIPTION\_FROM\_RNA\_POLYMERASE\_II\_PROMOTER |  | 428 | 0.19 | 1.20 | 0.060 | 0.375 | 1.000 | 2796 | tags=26%, list=21%, signal=32% |
| 112 | REGULATION\_OF\_KINASE\_ACTIVITY |  | 135 | 0.22 | 1.19 | 0.151 | 0.393 | 1.000 | 2109 | tags=24%, list=16%, signal=28% |
| 113 | CHROMATIN\_MODIFICATION |  | 46 | 0.27 | 1.18 | 0.209 | 0.407 | 1.000 | 2923 | tags=35%, list=22%, signal=45% |
| 114 | REGULATION\_OF\_TRANSFERASE\_ACTIVITY |  | 137 | 0.22 | 1.17 | 0.163 | 0.425 | 1.000 | 2109 | tags=23%, list=16%, signal=28% |
| 115 | CELLULAR\_RESPIRATION |  | 19 | 0.34 | 1.17 | 0.255 | 0.431 | 1.000 | 2657 | tags=37%, list=20%, signal=46% |
| 116 | REGULATION\_OF\_HYDROLASE\_ACTIVITY |  | 65 | 0.25 | 1.17 | 0.215 | 0.428 | 1.000 | 2779 | tags=32%, list=21%, signal=41% |
| 117 | NITROGEN\_COMPOUND\_BIOSYNTHETIC\_PROCESS |  | 25 | 0.31 | 1.17 | 0.256 | 0.425 | 1.000 | 2290 | tags=28%, list=17%, signal=34% |
| 118 | TRANSCRIPTION\_FROM\_RNA\_POLYMERASE\_III\_PROMOTER |  | 18 | 0.35 | 1.16 | 0.282 | 0.440 | 1.000 | 3912 | tags=56%, list=30%, signal=79% |
| 119 | GLUTAMATE\_SIGNALING\_PATHWAY |  | 17 | 0.35 | 1.16 | 0.263 | 0.438 | 1.000 | 3170 | tags=29%, list=24%, signal=39% |
| 120 | CELLULAR\_RESPONSE\_TO\_STIMULUS |  | 17 | 0.34 | 1.15 | 0.269 | 0.438 | 1.000 | 4949 | tags=65%, list=38%, signal=104% |
| 121 | REGULATION\_OF\_PROTEIN\_KINASE\_ACTIVITY |  | 133 | 0.22 | 1.15 | 0.189 | 0.443 | 1.000 | 2109 | tags=23%, list=16%, signal=28% |
| 122 | CATABOLIC\_PROCESS |  | 201 | 0.20 | 1.14 | 0.176 | 0.456 | 1.000 | 2657 | tags=25%, list=20%, signal=31% |
| 123 | REGULATION\_OF\_CATALYTIC\_ACTIVITY |  | 238 | 0.19 | 1.14 | 0.163 | 0.454 | 1.000 | 2923 | tags=27%, list=22%, signal=35% |
| 124 | RNA\_SPLICINGVIA\_TRANSESTERIFICATION\_REACTIONS |  | 27 | 0.30 | 1.14 | 0.268 | 0.452 | 1.000 | 3725 | tags=41%, list=28%, signal=57% |
| 125 | INDUCTION\_OF\_APOPTOSIS\_BY\_EXTRACELLULAR\_SIGNALS |  | 25 | 0.31 | 1.13 | 0.307 | 0.474 | 1.000 | 2664 | tags=36%, list=20%, signal=45% |
| 126 | NUCLEAR\_IMPORT |  | 47 | 0.26 | 1.12 | 0.266 | 0.489 | 1.000 | 2666 | tags=30%, list=20%, signal=37% |
| 127 | CELLULAR\_CATABOLIC\_PROCESS |  | 189 | 0.20 | 1.12 | 0.215 | 0.493 | 1.000 | 2736 | tags=25%, list=21%, signal=32% |
| 128 | NEGATIVE\_REGULATION\_OF\_TRANSPORT |  | 18 | 0.33 | 1.11 | 0.311 | 0.508 | 1.000 | 3478 | tags=44%, list=27%, signal=60% |
| 129 | INTERACTION\_WITH\_HOST |  | 15 | 0.34 | 1.10 | 0.330 | 0.533 | 1.000 | 1113 | tags=27%, list=9%, signal=29% |
| 130 | NEURON\_APOPTOSIS |  | 15 | 0.34 | 1.10 | 0.319 | 0.532 | 1.000 | 1244 | tags=27%, list=10%, signal=29% |
| 131 | REGULATION\_OF\_MOLECULAR\_FUNCTION |  | 275 | 0.18 | 1.09 | 0.237 | 0.536 | 1.000 | 3100 | tags=28%, list=24%, signal=36% |
| 132 | CELLULAR\_BIOSYNTHETIC\_PROCESS |  | 273 | 0.18 | 1.09 | 0.260 | 0.546 | 1.000 | 1740 | tags=19%, list=13%, signal=22% |
| 133 | DNA\_DAMAGE\_RESPONSESIGNAL\_TRANSDUCTION\_RESULTING\_IN\_INDUCTION\_OF\_APOPTOSIS |  | 15 | 0.33 | 1.07 | 0.363 | 0.595 | 1.000 | 1106 | tags=27%, list=8%, signal=29% |
| 134 | REGULATION\_OF\_NEUROTRANSMITTER\_LEVELS |  | 23 | 0.29 | 1.05 | 0.381 | 0.629 | 1.000 | 877 | tags=17%, list=7%, signal=19% |
| 135 | GAMETE\_GENERATION |  | 92 | 0.21 | 1.05 | 0.347 | 0.627 | 1.000 | 3906 | tags=34%, list=30%, signal=48% |
| 136 | RESPONSE\_TO\_TEMPERATURE\_STIMULUS |  | 16 | 0.32 | 1.03 | 0.418 | 0.666 | 1.000 | 3492 | tags=44%, list=27%, signal=60% |
| 137 | LIPID\_BIOSYNTHETIC\_PROCESS |  | 84 | 0.21 | 1.03 | 0.407 | 0.666 | 1.000 | 1244 | tags=18%, list=10%, signal=20% |
| 138 | STEROID\_BIOSYNTHETIC\_PROCESS |  | 22 | 0.29 | 1.03 | 0.413 | 0.661 | 1.000 | 4087 | tags=55%, list=31%, signal=79% |
| 139 | REGULATION\_OF\_PROTEIN\_STABILITY |  | 17 | 0.32 | 1.03 | 0.415 | 0.658 | 1.000 | 4128 | tags=41%, list=32%, signal=60% |
| 140 | RESPONSE\_TO\_HORMONE\_STIMULUS |  | 26 | 0.27 | 1.03 | 0.415 | 0.654 | 1.000 | 3492 | tags=38%, list=27%, signal=52% |
| 141 | PIGMENT\_BIOSYNTHETIC\_PROCESS |  | 17 | 0.31 | 1.03 | 0.419 | 0.652 | 1.000 | 1533 | tags=24%, list=12%, signal=27% |
| 142 | NEGATIVE\_REGULATION\_OF\_TRANSFERASE\_ACTIVITY |  | 27 | 0.27 | 1.03 | 0.436 | 0.656 | 1.000 | 2555 | tags=33%, list=20%, signal=41% |
| 143 | INTRACELLULAR\_PROTEIN\_TRANSPORT |  | 127 | 0.19 | 1.03 | 0.413 | 0.658 | 1.000 | 3313 | tags=29%, list=25%, signal=39% |
| 144 | MACROMOLECULE\_LOCALIZATION |  | 202 | 0.18 | 1.02 | 0.425 | 0.678 | 1.000 | 3218 | tags=28%, list=25%, signal=36% |
| 145 | DIGESTION |  | 42 | 0.24 | 1.00 | 0.415 | 0.705 | 1.000 | 3388 | tags=26%, list=26%, signal=35% |
| 146 | CYTOSKELETON\_ORGANIZATION\_AND\_BIOGENESIS |  | 182 | 0.18 | 1.00 | 0.452 | 0.704 | 1.000 | 2988 | tags=27%, list=23%, signal=35% |
| 147 | PROTEIN\_IMPORT |  | 58 | 0.22 | 1.00 | 0.447 | 0.703 | 1.000 | 3313 | tags=29%, list=25%, signal=39% |
| 148 | PROTEIN\_TARGETING |  | 94 | 0.20 | 1.00 | 0.460 | 0.704 | 1.000 | 3313 | tags=29%, list=25%, signal=38% |
| 149 | SECONDARY\_METABOLIC\_PROCESS |  | 23 | 0.27 | 0.99 | 0.476 | 0.724 | 1.000 | 1533 | tags=22%, list=12%, signal=25% |
| 150 | NEGATIVE\_REGULATION\_OF\_APOPTOSIS |  | 136 | 0.18 | 0.99 | 0.486 | 0.723 | 1.000 | 1874 | tags=21%, list=14%, signal=24% |
| 151 | PROTEIN\_TRANSPORT |  | 139 | 0.19 | 0.99 | 0.499 | 0.722 | 1.000 | 3313 | tags=29%, list=25%, signal=38% |
| 152 | STEROID\_METABOLIC\_PROCESS |  | 66 | 0.21 | 0.98 | 0.504 | 0.732 | 1.000 | 3753 | tags=35%, list=29%, signal=49% |
| 153 | REGULATION\_OF\_PROGRAMMED\_CELL\_DEATH |  | 313 | 0.16 | 0.97 | 0.533 | 0.747 | 1.000 | 1668 | tags=17%, list=13%, signal=19% |
| 154 | PIGMENT\_METABOLIC\_PROCESS |  | 18 | 0.29 | 0.97 | 0.490 | 0.744 | 1.000 | 1533 | tags=22%, list=12%, signal=25% |
| 155 | NEGATIVE\_REGULATION\_OF\_PROGRAMMED\_CELL\_DEATH |  | 137 | 0.18 | 0.97 | 0.512 | 0.752 | 1.000 | 1874 | tags=20%, list=14%, signal=24% |
| 156 | ENERGY\_DERIVATION\_BY\_OXIDATION\_OF\_ORGANIC\_COMPOUNDS |  | 37 | 0.24 | 0.97 | 0.493 | 0.755 | 1.000 | 1257 | tags=19%, list=10%, signal=21% |
| 157 | REGULATION\_OF\_APOPTOSIS |  | 312 | 0.16 | 0.96 | 0.564 | 0.765 | 1.000 | 1668 | tags=17%, list=13%, signal=19% |
| 158 | AROMATIC\_COMPOUND\_METABOLIC\_PROCESS |  | 26 | 0.25 | 0.96 | 0.505 | 0.764 | 1.000 | 239 | tags=15%, list=2%, signal=16% |
| 159 | PROGRAMMED\_CELL\_DEATH |  | 393 | 0.15 | 0.95 | 0.590 | 0.774 | 1.000 | 1738 | tags=17%, list=13%, signal=19% |
| 160 | APOPTOSIS\_GO |  | 392 | 0.15 | 0.95 | 0.604 | 0.778 | 1.000 | 1738 | tags=17%, list=13%, signal=19% |
| 161 | CARBOHYDRATE\_TRANSPORT |  | 17 | 0.29 | 0.95 | 0.538 | 0.773 | 1.000 | 2601 | tags=29%, list=20%, signal=37% |
| 162 | SPLICEOSOME\_ASSEMBLY |  | 17 | 0.29 | 0.95 | 0.520 | 0.772 | 1.000 | 3725 | tags=41%, list=28%, signal=57% |
| 163 | COVALENT\_CHROMATIN\_MODIFICATION |  | 22 | 0.27 | 0.94 | 0.530 | 0.781 | 1.000 | 3972 | tags=45%, list=30%, signal=65% |
| 164 | REGULATION\_OF\_TRANSCRIPTION\_FROM\_RNA\_POLYMERASE\_II\_PROMOTER |  | 267 | 0.16 | 0.94 | 0.597 | 0.785 | 1.000 | 3165 | tags=27%, list=24%, signal=35% |
| 165 | PROTEIN\_RNA\_COMPLEX\_ASSEMBLY |  | 55 | 0.21 | 0.94 | 0.545 | 0.784 | 1.000 | 3803 | tags=38%, list=29%, signal=54% |
| 166 | STEROID\_HORMONE\_RECEPTOR\_SIGNALING\_PATHWAY |  | 18 | 0.28 | 0.93 | 0.561 | 0.799 | 1.000 | 1479 | tags=22%, list=11%, signal=25% |
| 167 | PROTEIN\_IMPORT\_INTO\_NUCLEUS |  | 45 | 0.21 | 0.93 | 0.574 | 0.807 | 1.000 | 2666 | tags=27%, list=20%, signal=33% |
| 168 | MICROTUBULE\_BASED\_MOVEMENT |  | 16 | 0.29 | 0.92 | 0.564 | 0.809 | 1.000 | 2988 | tags=38%, list=23%, signal=49% |
| 169 | INTRACELLULAR\_RECEPTOR\_MEDIATED\_SIGNALING\_PATHWAY |  | 18 | 0.28 | 0.92 | 0.580 | 0.810 | 1.000 | 1479 | tags=22%, list=11%, signal=25% |
| 170 | MORPHOGENESIS\_OF\_AN\_EPITHELIUM |  | 15 | 0.29 | 0.92 | 0.543 | 0.811 | 1.000 | 3911 | tags=47%, list=30%, signal=66% |
| 171 | BIOSYNTHETIC\_PROCESS |  | 402 | 0.15 | 0.92 | 0.731 | 0.812 | 1.000 | 1792 | tags=17%, list=14%, signal=19% |
| 172 | PROTEIN\_AMINO\_ACID\_O\_LINKED\_GLYCOSYLATION |  | 18 | 0.27 | 0.91 | 0.558 | 0.816 | 1.000 | 2849 | tags=39%, list=22%, signal=50% |
| 173 | INDUCTION\_OF\_APOPTOSIS\_BY\_INTRACELLULAR\_SIGNALS |  | 23 | 0.25 | 0.91 | 0.589 | 0.812 | 1.000 | 1484 | tags=22%, list=11%, signal=24% |
| 174 | CELL\_PROJECTION\_BIOGENESIS |  | 20 | 0.26 | 0.91 | 0.590 | 0.815 | 1.000 | 4233 | tags=45%, list=32%, signal=66% |
| 175 | SEXUAL\_REPRODUCTION |  | 109 | 0.18 | 0.91 | 0.611 | 0.814 | 1.000 | 2777 | tags=22%, list=21%, signal=28% |
| 176 | GENERATION\_OF\_A\_SIGNAL\_INVOLVED\_IN\_CELL\_CELL\_SIGNALING |  | 25 | 0.25 | 0.91 | 0.591 | 0.811 | 1.000 | 2540 | tags=28%, list=19%, signal=35% |
| 177 | REGULATION\_OF\_RNA\_METABOLIC\_PROCESS |  | 417 | 0.15 | 0.90 | 0.755 | 0.819 | 1.000 | 3200 | tags=26%, list=24%, signal=34% |
| 178 | EXOCYTOSIS |  | 22 | 0.25 | 0.90 | 0.632 | 0.831 | 1.000 | 2274 | tags=23%, list=17%, signal=27% |
| 179 | POSITIVE\_REGULATION\_OF\_CELL\_CYCLE |  | 15 | 0.28 | 0.89 | 0.613 | 0.837 | 1.000 | 414 | tags=20%, list=3%, signal=21% |
| 180 | REGULATION\_OF\_TRANSPORT |  | 57 | 0.20 | 0.88 | 0.657 | 0.859 | 1.000 | 3478 | tags=33%, list=27%, signal=45% |
| 181 | ENERGY\_RESERVE\_METABOLIC\_PROCESS |  | 15 | 0.28 | 0.88 | 0.615 | 0.865 | 1.000 | 1257 | tags=20%, list=10%, signal=22% |
| 182 | REGULATION\_OF\_INTRACELLULAR\_TRANSPORT |  | 22 | 0.24 | 0.87 | 0.651 | 0.887 | 1.000 | 4494 | tags=50%, list=34%, signal=76% |
| 183 | REGULATION\_OF\_TRANSCRIPTIONDNA\_DEPENDENT |  | 412 | 0.14 | 0.86 | 0.872 | 0.883 | 1.000 | 3200 | tags=26%, list=24%, signal=33% |
| 184 | TRANSMISSION\_OF\_NERVE\_IMPULSE |  | 167 | 0.16 | 0.86 | 0.761 | 0.883 | 1.000 | 2790 | tags=21%, list=21%, signal=26% |
| 185 | CELLULAR\_CARBOHYDRATE\_METABOLIC\_PROCESS |  | 106 | 0.17 | 0.86 | 0.736 | 0.884 | 1.000 | 2948 | tags=24%, list=23%, signal=30% |
| 186 | RESPONSE\_TO\_RADIATION |  | 52 | 0.19 | 0.86 | 0.697 | 0.883 | 1.000 | 3837 | tags=35%, list=29%, signal=49% |
| 187 | NEGATIVE\_REGULATION\_OF\_CELL\_ADHESION |  | 16 | 0.27 | 0.85 | 0.644 | 0.891 | 1.000 | 3481 | tags=44%, list=27%, signal=60% |
| 188 | EPIDERMAL\_GROWTH\_FACTOR\_RECEPTOR\_SIGNALING\_PATHWAY |  | 18 | 0.25 | 0.84 | 0.690 | 0.906 | 1.000 | 4404 | tags=44%, list=34%, signal=67% |
| 189 | AEROBIC\_RESPIRATION |  | 15 | 0.27 | 0.84 | 0.686 | 0.912 | 1.000 | 2657 | tags=33%, list=20%, signal=42% |
| 190 | SYNAPTIC\_TRANSMISSION |  | 154 | 0.15 | 0.84 | 0.822 | 0.912 | 1.000 | 2790 | tags=20%, list=21%, signal=25% |
| 191 | REGULATION\_OF\_NUCLEOCYTOPLASMIC\_TRANSPORT |  | 19 | 0.24 | 0.82 | 0.718 | 0.934 | 1.000 | 4494 | tags=53%, list=34%, signal=80% |
| 192 | DEVELOPMENT\_OF\_PRIMARY\_SEXUAL\_CHARACTERISTICS |  | 25 | 0.22 | 0.82 | 0.742 | 0.938 | 1.000 | 3079 | tags=28%, list=24%, signal=37% |
| 193 | CELL\_CYCLE\_ARREST\_GO\_0007050 |  | 52 | 0.18 | 0.81 | 0.783 | 0.944 | 1.000 | 3930 | tags=40%, list=30%, signal=57% |
| 194 | NEGATIVE\_REGULATION\_OF\_CELL\_CYCLE |  | 72 | 0.17 | 0.81 | 0.806 | 0.942 | 1.000 | 2141 | tags=21%, list=16%, signal=25% |
| 195 | HISTONE\_MODIFICATION |  | 21 | 0.23 | 0.81 | 0.722 | 0.945 | 1.000 | 3972 | tags=43%, list=30%, signal=61% |
| 196 | MEMBRANE\_LIPID\_BIOSYNTHETIC\_PROCESS |  | 41 | 0.19 | 0.80 | 0.789 | 0.943 | 1.000 | 3840 | tags=34%, list=29%, signal=48% |
| 197 | REGULATION\_OF\_PHOSPHORYLATION |  | 42 | 0.19 | 0.80 | 0.790 | 0.942 | 1.000 | 4294 | tags=45%, list=33%, signal=67% |
| 198 | ESTABLISHMENT\_OF\_PROTEIN\_LOCALIZATION |  | 166 | 0.14 | 0.80 | 0.914 | 0.939 | 1.000 | 3218 | tags=25%, list=25%, signal=33% |
| 199 | CALCIUM\_INDEPENDENT\_CELL\_CELL\_ADHESION |  | 16 | 0.25 | 0.80 | 0.715 | 0.939 | 1.000 | 3251 | tags=31%, list=25%, signal=42% |
| 200 | CASPASE\_ACTIVATION |  | 24 | 0.22 | 0.80 | 0.755 | 0.937 | 1.000 | 3394 | tags=38%, list=26%, signal=51% |
| 201 | GLUCOSE\_METABOLIC\_PROCESS |  | 27 | 0.21 | 0.80 | 0.772 | 0.936 | 1.000 | 4675 | tags=44%, list=36%, signal=69% |
| 202 | PHOSPHOINOSITIDE\_BIOSYNTHETIC\_PROCESS |  | 21 | 0.23 | 0.79 | 0.760 | 0.942 | 1.000 | 1208 | tags=19%, list=9%, signal=21% |
| 203 | REGULATION\_OF\_CATABOLIC\_PROCESS |  | 15 | 0.25 | 0.79 | 0.743 | 0.940 | 1.000 | 2625 | tags=33%, list=20%, signal=42% |
| 204 | MEMBRANE\_FUSION |  | 27 | 0.21 | 0.78 | 0.781 | 0.939 | 1.000 | 3655 | tags=37%, list=28%, signal=51% |
| 205 | EMBRYONIC\_DEVELOPMENT |  | 46 | 0.18 | 0.78 | 0.826 | 0.939 | 1.000 | 3195 | tags=28%, list=24%, signal=37% |
| 206 | PROTEOLYSIS |  | 170 | 0.14 | 0.77 | 0.935 | 0.945 | 1.000 | 3712 | tags=29%, list=28%, signal=41% |
| 207 | RESPONSE\_TO\_UV |  | 22 | 0.21 | 0.76 | 0.800 | 0.955 | 1.000 | 3837 | tags=41%, list=29%, signal=58% |
| 208 | REPRODUCTION |  | 215 | 0.13 | 0.76 | 0.975 | 0.957 | 1.000 | 3100 | tags=22%, list=24%, signal=29% |
| 209 | PHOSPHOLIPID\_BIOSYNTHETIC\_PROCESS |  | 35 | 0.19 | 0.75 | 0.878 | 0.969 | 1.000 | 3840 | tags=34%, list=29%, signal=48% |
| 210 | LIPID\_TRANSPORT |  | 27 | 0.20 | 0.74 | 0.852 | 0.970 | 1.000 | 2038 | tags=22%, list=16%, signal=26% |
| 211 | STRESS\_ACTIVATED\_PROTEIN\_KINASE\_SIGNALING\_PATHWAY |  | 45 | 0.17 | 0.72 | 0.914 | 0.984 | 1.000 | 4157 | tags=38%, list=32%, signal=55% |
| 212 | JNK\_CASCADE |  | 44 | 0.17 | 0.72 | 0.898 | 0.983 | 1.000 | 4157 | tags=39%, list=32%, signal=56% |
| 213 | SENSORY\_PERCEPTION |  | 163 | 0.13 | 0.72 | 0.976 | 0.982 | 1.000 | 5232 | tags=42%, list=40%, signal=69% |
| 214 | POSITIVE\_REGULATION\_OF\_HYDROLASE\_ACTIVITY |  | 45 | 0.16 | 0.71 | 0.930 | 0.980 | 1.000 | 2687 | tags=24%, list=21%, signal=31% |
| 215 | GLYCEROPHOSPHOLIPID\_BIOSYNTHETIC\_PROCESS |  | 27 | 0.19 | 0.71 | 0.890 | 0.983 | 1.000 | 1208 | tags=15%, list=9%, signal=16% |
| 216 | NEUROLOGICAL\_SYSTEM\_PROCESS |  | 328 | 0.11 | 0.70 | 1.000 | 0.986 | 1.000 | 2790 | tags=17%, list=21%, signal=22% |
| 217 | ANION\_TRANSPORT |  | 27 | 0.19 | 0.70 | 0.905 | 0.983 | 1.000 | 2869 | tags=22%, list=22%, signal=28% |
| 218 | BRAIN\_DEVELOPMENT |  | 39 | 0.16 | 0.68 | 0.934 | 0.987 | 1.000 | 3079 | tags=28%, list=24%, signal=37% |
| 219 | LIPOPROTEIN\_METABOLIC\_PROCESS |  | 30 | 0.18 | 0.68 | 0.910 | 0.983 | 1.000 | 2038 | tags=20%, list=16%, signal=24% |
| 220 | BIOGENIC\_AMINE\_METABOLIC\_PROCESS |  | 16 | 0.21 | 0.68 | 0.892 | 0.984 | 1.000 | 1669 | tags=19%, list=13%, signal=21% |
| 221 | NEGATIVE\_REGULATION\_OF\_CELLULAR\_BIOSYNTHETIC\_PROCESS |  | 25 | 0.18 | 0.66 | 0.948 | 0.988 | 1.000 | 3332 | tags=28%, list=25%, signal=37% |
| 222 | HOMEOSTASIS\_OF\_NUMBER\_OF\_CELLS |  | 20 | 0.19 | 0.66 | 0.924 | 0.984 | 1.000 | 2645 | tags=25%, list=20%, signal=31% |
| 223 | NEGATIVE\_REGULATION\_OF\_BIOSYNTHETIC\_PROCESS |  | 26 | 0.17 | 0.62 | 0.943 | 1.000 | 1.000 | 3332 | tags=27%, list=25%, signal=36% |
| 224 | REGULATION\_OF\_CELL\_ADHESION |  | 31 | 0.16 | 0.62 | 0.970 | 0.997 | 1.000 | 4194 | tags=42%, list=32%, signal=62% |
| 225 | ADENYLATE\_CYCLASE\_ACTIVATION |  | 18 | 0.18 | 0.61 | 0.956 | 0.997 | 1.000 | 4521 | tags=39%, list=35%, signal=59% |
| 226 | LIPOPROTEIN\_BIOSYNTHETIC\_PROCESS |  | 23 | 0.16 | 0.56 | 0.975 | 1.000 | 1.000 | 4086 | tags=35%, list=31%, signal=50% |
| 227 | FEEDING\_BEHAVIOR |  | 20 | 0.16 | 0.55 | 0.984 | 1.000 | 1.000 | 2687 | tags=20%, list=21%, signal=25% |
| 228 | DETECTION\_OF\_ABIOTIC\_STIMULUS |  | 16 | 0.17 | 0.54 | 0.979 | 1.000 | 1.000 | 63 | tags=6%, list=0%, signal=6% |
| 229 | TUBE\_DEVELOPMENT |  | 15 | 0.16 | 0.52 | 0.984 | 0.998 | 1.000 | 3911 | tags=33%, list=30%, signal=47% |
| 230 | NEGATIVE\_REGULATION\_OF\_TRANSLATION |  | 19 | 0.14 | 0.47 | 1.000 | 0.998 | 1.000 | 3332 | tags=26%, list=25%, signal=35% |
Table: Gene sets enriched in phenotype **na**[plain text format]****

  
