# Supplementary material for: Multiple-input multiple-output causal strategies for gene selection
Source: BMC Bioinformatics. 2011 Nov 25;12:458. doi: 10.1186/1471-2105-12-458 (PMC3323860; doi:10.1186/1471-2105-12-458)
Supplement: Additional file 3 — Archive containing the output files computed by the preranked GSEA for λ ∈ {0.6,0.7,0.8,0.9,1.0,2.0} (GSEA_MIMO_part2.zip). [file 1471-2105-12-458-S3.ZIP › mFS09_entrez_mimo.GseaPreranked.1316039282297/gsea_report_for_na_neg_1316039282297.html]

Report for na\_neg 1316039282297 [GSEA]

| GS  follow link to MSigDB | GS DETAILS | SIZE | ES | NES | NOM p-val | FDR q-val | FWER p-val | RANK AT MAX | LEADING EDGE || 1 | IMMUNE\_RESPONSE |  | 212 | -0.41 | -2.41 | 0.000 | 0.003 | 0.002 | 3492 | tags=48%, list=27%, signal=65% |
| 2 | DEFENSE\_RESPONSE |  | 238 | -0.39 | -2.37 | 0.000 | 0.002 | 0.003 | 3505 | tags=44%, list=27%, signal=59% |
| 3 | IMMUNE\_SYSTEM\_PROCESS |  | 298 | -0.37 | -2.31 | 0.000 | 0.002 | 0.005 | 2846 | tags=40%, list=22%, signal=49% |
| 4 | POSITIVE\_REGULATION\_OF\_IMMUNE\_RESPONSE |  | 24 | -0.58 | -2.17 | 0.000 | 0.007 | 0.021 | 2832 | tags=58%, list=22%, signal=74% |
| 5 | INFLAMMATORY\_RESPONSE |  | 115 | -0.40 | -2.16 | 0.000 | 0.007 | 0.026 | 3090 | tags=43%, list=24%, signal=55% |
| 6 | POSITIVE\_REGULATION\_OF\_MULTICELLULAR\_ORGANISMAL\_PROCESS |  | 56 | -0.45 | -2.09 | 0.000 | 0.012 | 0.056 | 3211 | tags=50%, list=25%, signal=66% |
| 7 | REGULATION\_OF\_IMMUNE\_RESPONSE |  | 28 | -0.52 | -2.08 | 0.000 | 0.011 | 0.060 | 2832 | tags=54%, list=22%, signal=68% |
| 8 | CELLULAR\_DEFENSE\_RESPONSE |  | 54 | -0.45 | -2.07 | 0.000 | 0.011 | 0.067 | 3413 | tags=48%, list=26%, signal=65% |
| 9 | POSITIVE\_REGULATION\_OF\_IMMUNE\_SYSTEM\_PROCESS |  | 44 | -0.47 | -2.06 | 0.000 | 0.010 | 0.071 | 3211 | tags=50%, list=25%, signal=66% |
| 10 | RESPONSE\_TO\_WOUNDING |  | 171 | -0.36 | -2.06 | 0.000 | 0.010 | 0.073 | 3277 | tags=41%, list=25%, signal=54% |
| 11 | REGULATION\_OF\_IMMUNE\_SYSTEM\_PROCESS |  | 57 | -0.43 | -2.03 | 0.000 | 0.013 | 0.106 | 3284 | tags=51%, list=25%, signal=68% |
| 12 | ADAPTIVE\_IMMUNE\_RESPONSE\_GO\_0002460 |  | 22 | -0.50 | -1.83 | 0.014 | 0.071 | 0.487 | 3090 | tags=50%, list=24%, signal=65% |
| 13 | HEMOPOIETIC\_OR\_LYMPHOID\_ORGAN\_DEVELOPMENT |  | 71 | -0.36 | -1.80 | 0.000 | 0.085 | 0.585 | 2779 | tags=39%, list=21%, signal=50% |
| 14 | ADAPTIVE\_IMMUNE\_RESPONSE |  | 23 | -0.48 | -1.80 | 0.004 | 0.081 | 0.593 | 3090 | tags=48%, list=24%, signal=62% |
| 15 | HEMOPOIESIS |  | 69 | -0.36 | -1.79 | 0.002 | 0.081 | 0.622 | 2779 | tags=39%, list=21%, signal=49% |
| 16 | REGULATION\_OF\_MULTICELLULAR\_ORGANISMAL\_PROCESS |  | 131 | -0.32 | -1.77 | 0.000 | 0.090 | 0.683 | 3430 | tags=41%, list=26%, signal=55% |
| 17 | RECEPTOR\_MEDIATED\_ENDOCYTOSIS |  | 31 | -0.45 | -1.75 | 0.009 | 0.093 | 0.714 | 1908 | tags=35%, list=15%, signal=41% |
| 18 | IMMUNE\_SYSTEM\_DEVELOPMENT |  | 75 | -0.35 | -1.74 | 0.000 | 0.096 | 0.744 | 2779 | tags=39%, list=21%, signal=49% |
| 19 | IMMUNE\_EFFECTOR\_PROCESS |  | 34 | -0.42 | -1.74 | 0.007 | 0.094 | 0.755 | 3284 | tags=56%, list=25%, signal=74% |
| 20 | RESPONSE\_TO\_EXTERNAL\_STIMULUS |  | 278 | -0.28 | -1.73 | 0.000 | 0.100 | 0.799 | 2246 | tags=28%, list=17%, signal=33% |
| 21 | LYMPHOCYTE\_ACTIVATION |  | 54 | -0.37 | -1.72 | 0.000 | 0.101 | 0.815 | 3211 | tags=46%, list=25%, signal=61% |
| 22 | JAK\_STAT\_CASCADE |  | 26 | -0.43 | -1.68 | 0.019 | 0.135 | 0.898 | 1682 | tags=35%, list=13%, signal=40% |
| 23 | REGULATION\_OF\_DEFENSE\_RESPONSE |  | 15 | -0.52 | -1.67 | 0.025 | 0.138 | 0.914 | 3477 | tags=60%, list=27%, signal=82% |
| 24 | POSITIVE\_REGULATION\_OF\_RESPONSE\_TO\_STIMULUS |  | 35 | -0.39 | -1.67 | 0.017 | 0.135 | 0.919 | 2832 | tags=46%, list=22%, signal=58% |
| 25 | LIPID\_CATABOLIC\_PROCESS |  | 34 | -0.39 | -1.64 | 0.013 | 0.150 | 0.940 | 3945 | tags=56%, list=30%, signal=80% |
| 26 | T\_CELL\_ACTIVATION |  | 39 | -0.38 | -1.64 | 0.013 | 0.148 | 0.942 | 3211 | tags=44%, list=25%, signal=58% |
| 27 | CELL\_ACTIVATION |  | 64 | -0.34 | -1.63 | 0.002 | 0.150 | 0.949 | 3576 | tags=47%, list=27%, signal=64% |
| 28 | HUMORAL\_IMMUNE\_RESPONSE |  | 30 | -0.41 | -1.63 | 0.020 | 0.144 | 0.950 | 3238 | tags=53%, list=25%, signal=71% |
| 29 | LEUKOCYTE\_DIFFERENTIATION |  | 34 | -0.39 | -1.63 | 0.004 | 0.141 | 0.953 | 2779 | tags=44%, list=21%, signal=56% |
| 30 | LEUKOCYTE\_ACTIVATION |  | 59 | -0.34 | -1.61 | 0.007 | 0.155 | 0.967 | 3211 | tags=44%, list=25%, signal=58% |
| 31 | REGULATION\_OF\_CELL\_DIFFERENTIATION |  | 48 | -0.35 | -1.58 | 0.009 | 0.196 | 0.989 | 3281 | tags=40%, list=25%, signal=53% |
| 32 | LYMPHOCYTE\_DIFFERENTIATION |  | 23 | -0.42 | -1.55 | 0.029 | 0.227 | 0.998 | 3139 | tags=52%, list=24%, signal=69% |
| 33 | MULTI\_ORGANISM\_PROCESS |  | 137 | -0.27 | -1.55 | 0.000 | 0.220 | 0.998 | 3638 | tags=42%, list=28%, signal=58% |
| 34 | B\_CELL\_ACTIVATION |  | 17 | -0.45 | -1.54 | 0.045 | 0.228 | 0.999 | 3090 | tags=59%, list=24%, signal=77% |
| 35 | ENZYME\_LINKED\_RECEPTOR\_PROTEIN\_SIGNALING\_PATHWAY |  | 128 | -0.28 | -1.53 | 0.005 | 0.233 | 0.999 | 1451 | tags=22%, list=11%, signal=24% |
| 36 | TRANSFORMING\_GROWTH\_FACTOR\_BETA\_RECEPTOR\_SIGNALING\_PATHWAY |  | 34 | -0.37 | -1.53 | 0.025 | 0.235 | 0.999 | 3213 | tags=44%, list=25%, signal=58% |
| 37 | NEGATIVE\_REGULATION\_OF\_SIGNAL\_TRANSDUCTION |  | 31 | -0.38 | -1.51 | 0.046 | 0.254 | 0.999 | 2741 | tags=35%, list=21%, signal=45% |
| 38 | CELL\_SUBSTRATE\_ADHESION |  | 36 | -0.35 | -1.50 | 0.032 | 0.259 | 0.999 | 2455 | tags=36%, list=19%, signal=44% |
| 39 | ACTIN\_CYTOSKELETON\_ORGANIZATION\_AND\_BIOGENESIS |  | 90 | -0.29 | -1.50 | 0.017 | 0.253 | 0.999 | 2900 | tags=32%, list=22%, signal=41% |
| 40 | TRANSMEMBRANE\_RECEPTOR\_PROTEIN\_SERINE\_THREONINE\_KINASE\_SIGNALING\_PATHWAY |  | 42 | -0.34 | -1.48 | 0.040 | 0.289 | 1.000 | 3213 | tags=40%, list=25%, signal=53% |
| 41 | REGULATION\_OF\_RESPONSE\_TO\_STIMULUS |  | 49 | -0.33 | -1.48 | 0.024 | 0.285 | 1.000 | 3477 | tags=47%, list=27%, signal=64% |
| 42 | PEPTIDYL\_TYROSINE\_MODIFICATION |  | 23 | -0.41 | -1.47 | 0.045 | 0.285 | 1.000 | 1427 | tags=26%, list=11%, signal=29% |
| 43 | MAINTENANCE\_OF\_LOCALIZATION |  | 21 | -0.41 | -1.47 | 0.050 | 0.286 | 1.000 | 2631 | tags=38%, list=20%, signal=48% |
| 44 | CELLULAR\_LIPID\_CATABOLIC\_PROCESS |  | 31 | -0.36 | -1.47 | 0.057 | 0.286 | 1.000 | 3945 | tags=52%, list=30%, signal=74% |
| 45 | PROTEIN\_AMINO\_ACID\_N\_LINKED\_GLYCOSYLATION |  | 27 | -0.37 | -1.46 | 0.045 | 0.297 | 1.000 | 2515 | tags=37%, list=19%, signal=46% |
| 46 | REGULATION\_OF\_LYMPHOCYTE\_ACTIVATION |  | 31 | -0.37 | -1.45 | 0.056 | 0.294 | 1.000 | 3211 | tags=45%, list=25%, signal=60% |
| 47 | POSITIVE\_REGULATION\_OF\_SIGNAL\_TRANSDUCTION |  | 97 | -0.27 | -1.45 | 0.011 | 0.293 | 1.000 | 2843 | tags=34%, list=22%, signal=43% |
| 48 | REGULATION\_OF\_PROTEIN\_AMINO\_ACID\_PHOSPHORYLATION |  | 23 | -0.40 | -1.45 | 0.065 | 0.289 | 1.000 | 2455 | tags=35%, list=19%, signal=43% |
| 49 | DETECTION\_OF\_STIMULUS |  | 36 | -0.35 | -1.45 | 0.044 | 0.285 | 1.000 | 4761 | tags=56%, list=36%, signal=87% |
| 50 | REGULATION\_OF\_SIGNAL\_TRANSDUCTION |  | 173 | -0.25 | -1.44 | 0.019 | 0.297 | 1.000 | 3225 | tags=34%, list=25%, signal=44% |
| 51 | CATION\_HOMEOSTASIS |  | 94 | -0.27 | -1.43 | 0.023 | 0.300 | 1.000 | 3087 | tags=35%, list=24%, signal=46% |
| 52 | CYTOKINE\_AND\_CHEMOKINE\_MEDIATED\_SIGNALING\_PATHWAY |  | 19 | -0.41 | -1.42 | 0.067 | 0.312 | 1.000 | 3592 | tags=47%, list=27%, signal=65% |
| 53 | INNATE\_IMMUNE\_RESPONSE |  | 19 | -0.40 | -1.42 | 0.072 | 0.309 | 1.000 | 4940 | tags=74%, list=38%, signal=118% |
| 54 | RESPONSE\_TO\_OTHER\_ORGANISM |  | 69 | -0.29 | -1.42 | 0.048 | 0.312 | 1.000 | 2102 | tags=30%, list=16%, signal=36% |
| 55 | REGULATION\_OF\_CYTOSKELETON\_ORGANIZATION\_AND\_BIOGENESIS |  | 26 | -0.37 | -1.42 | 0.060 | 0.308 | 1.000 | 2580 | tags=35%, list=20%, signal=43% |
| 56 | SMALL\_GTPASE\_MEDIATED\_SIGNAL\_TRANSDUCTION |  | 77 | -0.28 | -1.42 | 0.023 | 0.303 | 1.000 | 3476 | tags=40%, list=27%, signal=54% |
| 57 | POSITIVE\_REGULATION\_OF\_PHOSPHATE\_METABOLIC\_PROCESS |  | 23 | -0.38 | -1.42 | 0.057 | 0.301 | 1.000 | 1621 | tags=30%, list=12%, signal=35% |
| 58 | PROTEIN\_KINASE\_CASCADE |  | 239 | -0.23 | -1.41 | 0.010 | 0.304 | 1.000 | 2490 | tags=27%, list=19%, signal=33% |
| 59 | REGULATION\_OF\_T\_CELL\_ACTIVATION |  | 25 | -0.36 | -1.41 | 0.050 | 0.303 | 1.000 | 3211 | tags=44%, list=25%, signal=58% |
| 60 | CELLULAR\_CATION\_HOMEOSTASIS |  | 91 | -0.27 | -1.41 | 0.037 | 0.303 | 1.000 | 3087 | tags=35%, list=24%, signal=46% |
| 61 | PROTEIN\_COMPLEX\_ASSEMBLY |  | 157 | -0.24 | -1.40 | 0.017 | 0.306 | 1.000 | 2958 | tags=31%, list=23%, signal=40% |
| 62 | MESODERM\_DEVELOPMENT |  | 22 | -0.38 | -1.40 | 0.090 | 0.302 | 1.000 | 4699 | tags=59%, list=36%, signal=92% |
| 63 | POSITIVE\_REGULATION\_OF\_LYMPHOCYTE\_ACTIVATION |  | 23 | -0.38 | -1.40 | 0.067 | 0.298 | 1.000 | 3211 | tags=43%, list=25%, signal=58% |
| 64 | REGULATION\_OF\_ANGIOGENESIS |  | 24 | -0.37 | -1.40 | 0.086 | 0.300 | 1.000 | 2163 | tags=42%, list=17%, signal=50% |
| 65 | POSITIVE\_REGULATION\_OF\_CELL\_DIFFERENTIATION |  | 21 | -0.39 | -1.39 | 0.098 | 0.302 | 1.000 | 3139 | tags=48%, list=24%, signal=63% |
| 66 | FEMALE\_PREGNANCY |  | 42 | -0.32 | -1.39 | 0.073 | 0.301 | 1.000 | 3790 | tags=50%, list=29%, signal=70% |
| 67 | CELL\_MATRIX\_ADHESION |  | 35 | -0.33 | -1.39 | 0.070 | 0.307 | 1.000 | 2455 | tags=34%, list=19%, signal=42% |
| 68 | GROWTH |  | 59 | -0.29 | -1.38 | 0.058 | 0.320 | 1.000 | 4190 | tags=44%, list=32%, signal=65% |
| 69 | TRANSMEMBRANE\_RECEPTOR\_PROTEIN\_TYROSINE\_KINASE\_SIGNALING\_PATHWAY |  | 76 | -0.27 | -1.37 | 0.050 | 0.322 | 1.000 | 1420 | tags=21%, list=11%, signal=23% |
| 70 | FATTY\_ACID\_METABOLIC\_PROCESS |  | 56 | -0.29 | -1.37 | 0.061 | 0.320 | 1.000 | 3945 | tags=48%, list=30%, signal=69% |
| 71 | WOUND\_HEALING |  | 49 | -0.30 | -1.36 | 0.071 | 0.343 | 1.000 | 3785 | tags=41%, list=29%, signal=57% |
| 72 | COAGULATION |  | 41 | -0.31 | -1.35 | 0.076 | 0.350 | 1.000 | 3785 | tags=41%, list=29%, signal=58% |
| 73 | BLOOD\_COAGULATION |  | 41 | -0.31 | -1.35 | 0.106 | 0.348 | 1.000 | 3785 | tags=41%, list=29%, signal=58% |
| 74 | CYTOKINE\_PRODUCTION |  | 61 | -0.29 | -1.35 | 0.051 | 0.346 | 1.000 | 2786 | tags=34%, list=21%, signal=44% |
| 75 | PEPTIDYL\_TYROSINE\_PHOSPHORYLATION |  | 21 | -0.37 | -1.34 | 0.117 | 0.348 | 1.000 | 1427 | tags=24%, list=11%, signal=27% |
| 76 | POSITIVE\_REGULATION\_OF\_CYTOKINE\_BIOSYNTHETIC\_PROCESS |  | 21 | -0.37 | -1.34 | 0.116 | 0.347 | 1.000 | 2786 | tags=43%, list=21%, signal=54% |
| 77 | PROTEIN\_AMINO\_ACID\_PHOSPHORYLATION |  | 231 | -0.22 | -1.34 | 0.015 | 0.346 | 1.000 | 3929 | tags=36%, list=30%, signal=51% |
| 78 | RESPONSE\_TO\_VIRUS |  | 45 | -0.30 | -1.33 | 0.099 | 0.367 | 1.000 | 2102 | tags=33%, list=16%, signal=40% |
| 79 | ACTIN\_POLYMERIZATION\_AND\_OR\_DEPOLYMERIZATION |  | 20 | -0.37 | -1.32 | 0.139 | 0.384 | 1.000 | 2580 | tags=30%, list=20%, signal=37% |
| 80 | REGULATION\_OF\_ANATOMICAL\_STRUCTURE\_MORPHOGENESIS |  | 17 | -0.38 | -1.31 | 0.129 | 0.391 | 1.000 | 3253 | tags=35%, list=25%, signal=47% |
| 81 | REGULATION\_OF\_CELL\_PROLIFERATION |  | 275 | -0.21 | -1.31 | 0.013 | 0.387 | 1.000 | 2527 | tags=25%, list=19%, signal=31% |
| 82 | GLYCOPROTEIN\_METABOLIC\_PROCESS |  | 82 | -0.26 | -1.31 | 0.060 | 0.385 | 1.000 | 3556 | tags=37%, list=27%, signal=50% |
| 83 | MUSCLE\_DEVELOPMENT |  | 85 | -0.26 | -1.31 | 0.068 | 0.384 | 1.000 | 3387 | tags=40%, list=26%, signal=54% |
| 84 | POSITIVE\_REGULATION\_OF\_PROTEIN\_AMINO\_ACID\_PHOSPHORYLATION |  | 15 | -0.40 | -1.30 | 0.124 | 0.399 | 1.000 | 1621 | tags=33%, list=12%, signal=38% |
| 85 | RAS\_PROTEIN\_SIGNAL\_TRANSDUCTION |  | 55 | -0.28 | -1.30 | 0.104 | 0.401 | 1.000 | 3193 | tags=38%, list=24%, signal=50% |
| 86 | ACTIN\_FILAMENT\_BASED\_PROCESS |  | 99 | -0.25 | -1.29 | 0.078 | 0.400 | 1.000 | 2900 | tags=29%, list=22%, signal=37% |
| 87 | MONOCARBOXYLIC\_ACID\_METABOLIC\_PROCESS |  | 77 | -0.26 | -1.28 | 0.075 | 0.416 | 1.000 | 4026 | tags=44%, list=31%, signal=63% |
| 88 | POSITIVE\_REGULATION\_OF\_CELL\_PROLIFERATION |  | 129 | -0.23 | -1.28 | 0.068 | 0.427 | 1.000 | 1719 | tags=22%, list=13%, signal=25% |
| 89 | REGULATION\_OF\_BODY\_FLUID\_LEVELS |  | 55 | -0.27 | -1.26 | 0.122 | 0.464 | 1.000 | 3785 | tags=40%, list=29%, signal=56% |
| 90 | PHOSPHOLIPID\_METABOLIC\_PROCESS |  | 63 | -0.26 | -1.26 | 0.106 | 0.468 | 1.000 | 2875 | tags=33%, list=22%, signal=43% |
| 91 | AMINO\_ACID\_TRANSPORT |  | 25 | -0.33 | -1.25 | 0.168 | 0.468 | 1.000 | 487 | tags=20%, list=4%, signal=21% |
| 92 | POSITIVE\_REGULATION\_OF\_TRANSLATION |  | 28 | -0.31 | -1.25 | 0.163 | 0.470 | 1.000 | 2786 | tags=39%, list=21%, signal=50% |
| 93 | POSITIVE\_REGULATION\_OF\_CELLULAR\_PROTEIN\_METABOLIC\_PROCESS |  | 61 | -0.26 | -1.25 | 0.135 | 0.469 | 1.000 | 2490 | tags=31%, list=19%, signal=38% |
| 94 | BEHAVIOR |  | 136 | -0.22 | -1.25 | 0.111 | 0.466 | 1.000 | 4034 | tags=38%, list=31%, signal=55% |
| 95 | RESPONSE\_TO\_BACTERIUM |  | 22 | -0.34 | -1.25 | 0.171 | 0.463 | 1.000 | 3917 | tags=45%, list=30%, signal=65% |
| 96 | REGULATION\_OF\_ORGANELLE\_ORGANIZATION\_AND\_BIOGENESIS |  | 35 | -0.30 | -1.24 | 0.174 | 0.471 | 1.000 | 2947 | tags=34%, list=23%, signal=44% |
| 97 | DEFENSE\_RESPONSE\_TO\_BACTERIUM |  | 16 | -0.37 | -1.24 | 0.176 | 0.472 | 1.000 | 3611 | tags=44%, list=28%, signal=60% |
| 98 | ACTIVATION\_OF\_NF\_KAPPAB\_TRANSCRIPTION\_FACTOR |  | 15 | -0.38 | -1.24 | 0.176 | 0.467 | 1.000 | 4049 | tags=60%, list=31%, signal=87% |
| 99 | REGULATION\_OF\_PROTEIN\_IMPORT\_INTO\_NUCLEUS |  | 15 | -0.38 | -1.24 | 0.183 | 0.465 | 1.000 | 1303 | tags=27%, list=10%, signal=30% |
| 100 | REGULATION\_OF\_I\_KAPPAB\_KINASE\_NF\_KAPPAB\_CASCADE |  | 72 | -0.25 | -1.23 | 0.128 | 0.471 | 1.000 | 3986 | tags=44%, list=30%, signal=64% |
| 101 | CELL\_RECOGNITION |  | 16 | -0.37 | -1.23 | 0.211 | 0.471 | 1.000 | 4482 | tags=56%, list=34%, signal=85% |
| 102 | REGULATION\_OF\_BLOOD\_PRESSURE |  | 22 | -0.33 | -1.23 | 0.187 | 0.473 | 1.000 | 3189 | tags=36%, list=24%, signal=48% |
| 103 | POSITIVE\_REGULATION\_OF\_PHOSPHORYLATION |  | 21 | -0.34 | -1.23 | 0.195 | 0.472 | 1.000 | 1621 | tags=29%, list=12%, signal=33% |
| 104 | POSITIVE\_REGULATION\_OF\_PROTEIN\_METABOLIC\_PROCESS |  | 63 | -0.26 | -1.23 | 0.148 | 0.468 | 1.000 | 2947 | tags=35%, list=23%, signal=45% |
| 105 | HEMOSTASIS |  | 46 | -0.28 | -1.23 | 0.133 | 0.465 | 1.000 | 3785 | tags=39%, list=29%, signal=55% |
| 106 | G\_PROTEIN\_SIGNALING\_COUPLED\_TO\_CAMP\_NUCLEOTIDE\_SECOND\_MESSENGER |  | 62 | -0.26 | -1.23 | 0.154 | 0.466 | 1.000 | 1931 | tags=21%, list=15%, signal=24% |
| 107 | ANATOMICAL\_STRUCTURE\_FORMATION |  | 52 | -0.26 | -1.22 | 0.159 | 0.464 | 1.000 | 1806 | tags=27%, list=14%, signal=31% |
| 108 | MUSCLE\_CELL\_DIFFERENTIATION |  | 21 | -0.34 | -1.22 | 0.205 | 0.464 | 1.000 | 3281 | tags=48%, list=25%, signal=63% |
| 109 | CAMP\_MEDIATED\_SIGNALING |  | 63 | -0.26 | -1.22 | 0.150 | 0.463 | 1.000 | 1931 | tags=21%, list=15%, signal=24% |
| 110 | ICOSANOID\_METABOLIC\_PROCESS |  | 16 | -0.38 | -1.22 | 0.226 | 0.470 | 1.000 | 3149 | tags=44%, list=24%, signal=58% |
| 111 | PROTEIN\_AMINO\_ACID\_DEPHOSPHORYLATION |  | 60 | -0.26 | -1.21 | 0.160 | 0.471 | 1.000 | 1812 | tags=22%, list=14%, signal=25% |
| 112 | NEGATIVE\_REGULATION\_OF\_TRANSCRIPTION |  | 166 | -0.21 | -1.21 | 0.114 | 0.470 | 1.000 | 2604 | tags=27%, list=20%, signal=33% |
| 113 | LIPID\_METABOLIC\_PROCESS |  | 283 | -0.19 | -1.21 | 0.088 | 0.474 | 1.000 | 4010 | tags=39%, list=31%, signal=55% |
| 114 | DEVELOPMENTAL\_MATURATION |  | 18 | -0.35 | -1.21 | 0.197 | 0.474 | 1.000 | 2861 | tags=39%, list=22%, signal=50% |
| 115 | GENERATION\_OF\_NEURONS |  | 65 | -0.25 | -1.20 | 0.143 | 0.483 | 1.000 | 3285 | tags=32%, list=25%, signal=43% |
| 116 | AMINE\_TRANSPORT |  | 36 | -0.29 | -1.20 | 0.210 | 0.490 | 1.000 | 2671 | tags=28%, list=20%, signal=35% |
| 117 | DEPHOSPHORYLATION |  | 67 | -0.24 | -1.20 | 0.173 | 0.491 | 1.000 | 1496 | tags=19%, list=11%, signal=22% |
| 118 | MYELOID\_CELL\_DIFFERENTIATION |  | 35 | -0.28 | -1.19 | 0.206 | 0.494 | 1.000 | 2767 | tags=31%, list=21%, signal=40% |
| 119 | I\_KAPPAB\_KINASE\_NF\_KAPPAB\_CASCADE |  | 88 | -0.23 | -1.19 | 0.156 | 0.490 | 1.000 | 2843 | tags=32%, list=22%, signal=40% |
| 120 | PROTEIN\_OLIGOMERIZATION |  | 37 | -0.29 | -1.19 | 0.205 | 0.490 | 1.000 | 2958 | tags=32%, list=23%, signal=42% |
| 121 | NEURON\_DIFFERENTIATION |  | 58 | -0.25 | -1.19 | 0.164 | 0.492 | 1.000 | 3709 | tags=34%, list=28%, signal=48% |
| 122 | ORGAN\_MORPHOGENESIS |  | 131 | -0.21 | -1.19 | 0.131 | 0.492 | 1.000 | 1405 | tags=18%, list=11%, signal=20% |
| 123 | RESPONSE\_TO\_BIOTIC\_STIMULUS |  | 103 | -0.23 | -1.18 | 0.156 | 0.498 | 1.000 | 2102 | tags=26%, list=16%, signal=31% |
| 124 | POSITIVE\_REGULATION\_OF\_T\_CELL\_ACTIVATION |  | 20 | -0.33 | -1.18 | 0.244 | 0.495 | 1.000 | 3211 | tags=40%, list=25%, signal=53% |
| 125 | REGULATION\_OF\_PROTEIN\_METABOLIC\_PROCESS |  | 150 | -0.21 | -1.18 | 0.135 | 0.492 | 1.000 | 2608 | tags=27%, list=20%, signal=33% |
| 126 | REGULATION\_OF\_MAPKKK\_CASCADE |  | 19 | -0.34 | -1.18 | 0.248 | 0.494 | 1.000 | 2195 | tags=32%, list=17%, signal=38% |
| 127 | ANGIOGENESIS |  | 44 | -0.27 | -1.18 | 0.205 | 0.492 | 1.000 | 3196 | tags=39%, list=24%, signal=51% |
| 128 | CYTOKINE\_BIOSYNTHETIC\_PROCESS |  | 34 | -0.29 | -1.18 | 0.217 | 0.488 | 1.000 | 2786 | tags=35%, list=21%, signal=45% |
| 129 | POSITIVE\_REGULATION\_OF\_I\_KAPPAB\_KINASE\_NF\_KAPPAB\_CASCADE |  | 67 | -0.24 | -1.17 | 0.179 | 0.501 | 1.000 | 2843 | tags=33%, list=22%, signal=42% |
| 130 | RESPONSE\_TO\_DRUG |  | 21 | -0.33 | -1.17 | 0.240 | 0.501 | 1.000 | 3072 | tags=43%, list=23%, signal=56% |
| 131 | NEGATIVE\_REGULATION\_OF\_CELL\_PROLIFERATION |  | 145 | -0.20 | -1.17 | 0.149 | 0.507 | 1.000 | 2527 | tags=26%, list=19%, signal=31% |
| 132 | STRIATED\_MUSCLE\_DEVELOPMENT |  | 36 | -0.28 | -1.16 | 0.237 | 0.517 | 1.000 | 3387 | tags=44%, list=26%, signal=60% |
| 133 | BONE\_REMODELING |  | 28 | -0.30 | -1.16 | 0.250 | 0.513 | 1.000 | 2741 | tags=32%, list=21%, signal=41% |
| 134 | PHOSPHORYLATION |  | 262 | -0.19 | -1.16 | 0.125 | 0.514 | 1.000 | 2959 | tags=27%, list=23%, signal=34% |
| 135 | REGULATION\_OF\_MAP\_KINASE\_ACTIVITY |  | 56 | -0.25 | -1.16 | 0.222 | 0.515 | 1.000 | 1875 | tags=27%, list=14%, signal=31% |
| 136 | PROTEIN\_PROCESSING |  | 41 | -0.27 | -1.16 | 0.229 | 0.517 | 1.000 | 3929 | tags=39%, list=30%, signal=56% |
| 137 | POSITIVE\_REGULATION\_OF\_TRANSFERASE\_ACTIVITY |  | 71 | -0.23 | -1.15 | 0.221 | 0.531 | 1.000 | 2451 | tags=25%, list=19%, signal=31% |
| 138 | REGULATION\_OF\_CELLULAR\_PROTEIN\_METABOLIC\_PROCESS |  | 139 | -0.20 | -1.14 | 0.184 | 0.541 | 1.000 | 2580 | tags=26%, list=20%, signal=32% |
| 139 | LOCOMOTORY\_BEHAVIOR |  | 84 | -0.23 | -1.14 | 0.239 | 0.540 | 1.000 | 2448 | tags=26%, list=19%, signal=32% |
| 140 | ION\_HOMEOSTASIS |  | 112 | -0.21 | -1.14 | 0.183 | 0.541 | 1.000 | 3087 | tags=31%, list=24%, signal=41% |
| 141 | GENERATION\_OF\_PRECURSOR\_METABOLITES\_AND\_ENERGY |  | 120 | -0.21 | -1.14 | 0.193 | 0.539 | 1.000 | 2198 | tags=24%, list=17%, signal=29% |
| 142 | NEURON\_DEVELOPMENT |  | 49 | -0.25 | -1.14 | 0.217 | 0.541 | 1.000 | 3709 | tags=35%, list=28%, signal=48% |
| 143 | ACTIVATION\_OF\_MAPK\_ACTIVITY |  | 33 | -0.28 | -1.14 | 0.257 | 0.540 | 1.000 | 1749 | tags=27%, list=13%, signal=31% |
| 144 | HORMONE\_METABOLIC\_PROCESS |  | 29 | -0.29 | -1.13 | 0.261 | 0.557 | 1.000 | 3295 | tags=45%, list=25%, signal=60% |
| 145 | REGULATION\_OF\_DEVELOPMENTAL\_PROCESS |  | 387 | -0.17 | -1.13 | 0.123 | 0.554 | 1.000 | 3284 | tags=31%, list=25%, signal=40% |
| 146 | NEGATIVE\_REGULATION\_OF\_METABOLIC\_PROCESS |  | 232 | -0.18 | -1.13 | 0.184 | 0.556 | 1.000 | 2604 | tags=25%, list=20%, signal=31% |
| 147 | CELL\_MATURATION |  | 16 | -0.34 | -1.12 | 0.305 | 0.560 | 1.000 | 2861 | tags=38%, list=22%, signal=48% |
| 148 | CYTOKINE\_METABOLIC\_PROCESS |  | 35 | -0.27 | -1.12 | 0.269 | 0.557 | 1.000 | 2786 | tags=34%, list=21%, signal=43% |
| 149 | DETECTION\_OF\_EXTERNAL\_STIMULUS |  | 18 | -0.33 | -1.12 | 0.292 | 0.554 | 1.000 | 3855 | tags=39%, list=29%, signal=55% |
| 150 | POSITIVE\_REGULATION\_OF\_SECRETION |  | 18 | -0.33 | -1.12 | 0.293 | 0.554 | 1.000 | 4736 | tags=61%, list=36%, signal=96% |
| 151 | GLYCEROPHOSPHOLIPID\_METABOLIC\_PROCESS |  | 39 | -0.26 | -1.12 | 0.299 | 0.557 | 1.000 | 4522 | tags=51%, list=35%, signal=78% |
| 152 | NEGATIVE\_REGULATION\_OF\_NUCLEOBASENUCLEOSIDENUCLEOTIDE\_AND\_NUCLEIC\_ACID\_METABOLIC\_PROCESS |  | 185 | -0.19 | -1.12 | 0.195 | 0.554 | 1.000 | 2604 | tags=26%, list=20%, signal=33% |
| 153 | NEURITE\_DEVELOPMENT |  | 41 | -0.25 | -1.12 | 0.287 | 0.554 | 1.000 | 3709 | tags=34%, list=28%, signal=47% |
| 154 | PEPTIDYL\_AMINO\_ACID\_MODIFICATION |  | 47 | -0.25 | -1.11 | 0.259 | 0.562 | 1.000 | 2455 | tags=28%, list=19%, signal=34% |
| 155 | POST\_TRANSLATIONAL\_PROTEIN\_MODIFICATION |  | 409 | -0.17 | -1.11 | 0.145 | 0.567 | 1.000 | 2959 | tags=26%, list=23%, signal=32% |
| 156 | POSITIVE\_REGULATION\_OF\_DEVELOPMENTAL\_PROCESS |  | 197 | -0.19 | -1.10 | 0.246 | 0.576 | 1.000 | 3931 | tags=40%, list=30%, signal=56% |
| 157 | MEMBRANE\_ORGANIZATION\_AND\_BIOGENESIS |  | 124 | -0.20 | -1.10 | 0.241 | 0.575 | 1.000 | 2282 | tags=24%, list=17%, signal=29% |
| 158 | VASCULATURE\_DEVELOPMENT |  | 50 | -0.24 | -1.10 | 0.297 | 0.588 | 1.000 | 1806 | tags=24%, list=14%, signal=28% |
| 159 | TISSUE\_REMODELING |  | 29 | -0.27 | -1.09 | 0.324 | 0.589 | 1.000 | 2741 | tags=31%, list=21%, signal=39% |
| 160 | GLYCOPROTEIN\_BIOSYNTHETIC\_PROCESS |  | 67 | -0.23 | -1.09 | 0.291 | 0.587 | 1.000 | 4208 | tags=42%, list=32%, signal=61% |
| 161 | MEMBRANE\_LIPID\_METABOLIC\_PROCESS |  | 85 | -0.21 | -1.09 | 0.282 | 0.586 | 1.000 | 3525 | tags=36%, list=27%, signal=50% |
| 162 | ELECTRON\_TRANSPORT\_GO\_0006118 |  | 50 | -0.24 | -1.09 | 0.286 | 0.584 | 1.000 | 2198 | tags=26%, list=17%, signal=31% |
| 163 | SKELETAL\_DEVELOPMENT |  | 91 | -0.21 | -1.09 | 0.307 | 0.582 | 1.000 | 3323 | tags=33%, list=25%, signal=44% |
| 164 | CYTOKINE\_SECRETION |  | 15 | -0.34 | -1.09 | 0.341 | 0.584 | 1.000 | 2763 | tags=40%, list=21%, signal=51% |
| 165 | NEGATIVE\_REGULATION\_OF\_CELLULAR\_METABOLIC\_PROCESS |  | 229 | -0.18 | -1.09 | 0.224 | 0.589 | 1.000 | 2604 | tags=25%, list=20%, signal=31% |
| 166 | POSITIVE\_REGULATION\_OF\_PROTEIN\_MODIFICATION\_PROCESS |  | 24 | -0.29 | -1.09 | 0.316 | 0.586 | 1.000 | 1621 | tags=25%, list=12%, signal=28% |
| 167 | AXONOGENESIS |  | 33 | -0.27 | -1.08 | 0.333 | 0.591 | 1.000 | 3709 | tags=36%, list=28%, signal=51% |
| 168 | POSITIVE\_REGULATION\_OF\_CELLULAR\_METABOLIC\_PROCESS |  | 196 | -0.18 | -1.08 | 0.252 | 0.589 | 1.000 | 2964 | tags=28%, list=23%, signal=36% |
| 169 | ORGANIC\_ACID\_METABOLIC\_PROCESS |  | 162 | -0.19 | -1.08 | 0.272 | 0.587 | 1.000 | 3945 | tags=37%, list=30%, signal=52% |
| 170 | ANTI\_APOPTOSIS |  | 107 | -0.20 | -1.08 | 0.292 | 0.590 | 1.000 | 2100 | tags=25%, list=16%, signal=30% |
| 171 | POSITIVE\_REGULATION\_OF\_METABOLIC\_PROCESS |  | 201 | -0.18 | -1.08 | 0.273 | 0.589 | 1.000 | 2964 | tags=28%, list=23%, signal=35% |
| 172 | CARBOXYLIC\_ACID\_METABOLIC\_PROCESS |  | 160 | -0.19 | -1.08 | 0.281 | 0.587 | 1.000 | 3945 | tags=38%, list=30%, signal=53% |
| 173 | NEGATIVE\_REGULATION\_OF\_RNA\_METABOLIC\_PROCESS |  | 114 | -0.20 | -1.08 | 0.319 | 0.588 | 1.000 | 2850 | tags=28%, list=22%, signal=36% |
| 174 | AMINO\_ACID\_DERIVATIVE\_METABOLIC\_PROCESS |  | 23 | -0.29 | -1.07 | 0.341 | 0.593 | 1.000 | 4034 | tags=48%, list=31%, signal=69% |
| 175 | CELLULAR\_LIPID\_METABOLIC\_PROCESS |  | 220 | -0.18 | -1.07 | 0.288 | 0.600 | 1.000 | 3945 | tags=38%, list=30%, signal=53% |
| 176 | POSITIVE\_REGULATION\_OF\_TRANSCRIPTION |  | 124 | -0.19 | -1.07 | 0.305 | 0.608 | 1.000 | 2964 | tags=27%, list=23%, signal=35% |
| 177 | POSITIVE\_REGULATION\_OF\_MAP\_KINASE\_ACTIVITY |  | 39 | -0.25 | -1.06 | 0.350 | 0.607 | 1.000 | 1749 | tags=26%, list=13%, signal=30% |
| 178 | NEGATIVE\_REGULATION\_OF\_TRANSCRIPTION\_DNA\_DEPENDENT |  | 114 | -0.20 | -1.06 | 0.322 | 0.610 | 1.000 | 2850 | tags=28%, list=22%, signal=36% |
| 179 | POSITIVE\_REGULATION\_OF\_TRANSCRIPTION\_FACTOR\_ACTIVITY |  | 17 | -0.31 | -1.06 | 0.358 | 0.607 | 1.000 | 4049 | tags=53%, list=31%, signal=77% |
| 180 | POSITIVE\_REGULATION\_OF\_CATALYTIC\_ACTIVITY |  | 139 | -0.19 | -1.06 | 0.292 | 0.604 | 1.000 | 2451 | tags=22%, list=19%, signal=27% |
| 181 | CELL\_PROLIFERATION\_GO\_0008283 |  | 466 | -0.16 | -1.06 | 0.274 | 0.611 | 1.000 | 2543 | tags=23%, list=19%, signal=27% |
| 182 | NEGATIVE\_REGULATION\_OF\_TRANSCRIPTION\_FROM\_RNA\_POLYMERASE\_II\_PROMOTER |  | 76 | -0.21 | -1.05 | 0.355 | 0.616 | 1.000 | 2850 | tags=29%, list=22%, signal=37% |
| 183 | MYOBLAST\_DIFFERENTIATION |  | 16 | -0.32 | -1.05 | 0.380 | 0.615 | 1.000 | 3281 | tags=50%, list=25%, signal=67% |
| 184 | REGULATION\_OF\_BIOLOGICAL\_QUALITY |  | 364 | -0.16 | -1.05 | 0.295 | 0.619 | 1.000 | 4449 | tags=38%, list=34%, signal=56% |
| 185 | AMINO\_ACID\_METABOLIC\_PROCESS |  | 73 | -0.21 | -1.05 | 0.352 | 0.628 | 1.000 | 2254 | tags=26%, list=17%, signal=31% |
| 186 | ACTIN\_FILAMENT\_ORGANIZATION |  | 21 | -0.28 | -1.05 | 0.407 | 0.625 | 1.000 | 2580 | tags=33%, list=20%, signal=41% |
| 187 | ORGANIC\_ACID\_TRANSPORT |  | 39 | -0.24 | -1.04 | 0.362 | 0.630 | 1.000 | 922 | tags=18%, list=7%, signal=19% |
| 188 | RESPONSE\_TO\_CHEMICAL\_STIMULUS |  | 271 | -0.17 | -1.04 | 0.332 | 0.636 | 1.000 | 1672 | tags=18%, list=13%, signal=20% |
| 189 | CARBOXYLIC\_ACID\_TRANSPORT |  | 39 | -0.24 | -1.04 | 0.385 | 0.637 | 1.000 | 922 | tags=18%, list=7%, signal=19% |
| 190 | POSITIVE\_REGULATION\_OF\_DNA\_BINDING |  | 18 | -0.30 | -1.04 | 0.414 | 0.639 | 1.000 | 4731 | tags=61%, list=36%, signal=96% |
| 191 | NEUROGENESIS |  | 75 | -0.21 | -1.03 | 0.408 | 0.640 | 1.000 | 3285 | tags=31%, list=25%, signal=41% |
| 192 | PROTEIN\_AUTOPROCESSING |  | 24 | -0.27 | -1.03 | 0.385 | 0.640 | 1.000 | 3929 | tags=42%, list=30%, signal=59% |
| 193 | REGULATION\_OF\_CYTOKINE\_BIOSYNTHETIC\_PROCESS |  | 31 | -0.25 | -1.03 | 0.391 | 0.645 | 1.000 | 2786 | tags=32%, list=21%, signal=41% |
| 194 | REGULATION\_OF\_TRANSCRIPTION |  | 498 | -0.15 | -1.03 | 0.371 | 0.647 | 1.000 | 2767 | tags=24%, list=21%, signal=30% |
| 195 | CELLULAR\_COMPONENT\_ASSEMBLY |  | 272 | -0.17 | -1.02 | 0.357 | 0.653 | 1.000 | 2860 | tags=26%, list=22%, signal=33% |
| 196 | DETECTION\_OF\_STIMULUS\_INVOLVED\_IN\_SENSORY\_PERCEPTION |  | 15 | -0.31 | -1.02 | 0.439 | 0.655 | 1.000 | 9012 | tags=100%, list=69%, signal=321% |
| 197 | CELLULAR\_PROTEIN\_COMPLEX\_ASSEMBLY |  | 28 | -0.26 | -1.02 | 0.427 | 0.653 | 1.000 | 2695 | tags=29%, list=21%, signal=36% |
| 198 | MACROMOLECULE\_BIOSYNTHETIC\_PROCESS |  | 267 | -0.17 | -1.02 | 0.409 | 0.651 | 1.000 | 3051 | tags=27%, list=23%, signal=35% |
| 199 | PROTEIN\_AMINO\_ACID\_AUTOPHOSPHORYLATION |  | 24 | -0.27 | -1.02 | 0.439 | 0.651 | 1.000 | 3929 | tags=42%, list=30%, signal=59% |
| 200 | AMINO\_ACID\_CATABOLIC\_PROCESS |  | 23 | -0.28 | -1.02 | 0.421 | 0.656 | 1.000 | 2219 | tags=30%, list=17%, signal=37% |
| 201 | CELLULAR\_HOMEOSTASIS |  | 121 | -0.18 | -1.02 | 0.403 | 0.654 | 1.000 | 4489 | tags=45%, list=34%, signal=67% |
| 202 | NEGATIVE\_REGULATION\_OF\_DEVELOPMENTAL\_PROCESS |  | 177 | -0.18 | -1.02 | 0.415 | 0.656 | 1.000 | 2239 | tags=23%, list=17%, signal=28% |
| 203 | PHAGOCYTOSIS |  | 16 | -0.31 | -1.01 | 0.424 | 0.669 | 1.000 | 4891 | tags=63%, list=37%, signal=100% |
| 204 | REGULATION\_OF\_JNK\_ACTIVITY |  | 18 | -0.29 | -1.00 | 0.444 | 0.678 | 1.000 | 1749 | tags=28%, list=13%, signal=32% |
| 205 | VITAMIN\_METABOLIC\_PROCESS |  | 15 | -0.31 | -1.00 | 0.428 | 0.681 | 1.000 | 4051 | tags=53%, list=31%, signal=77% |
| 206 | RHYTHMIC\_PROCESS |  | 23 | -0.27 | -1.00 | 0.457 | 0.687 | 1.000 | 1962 | tags=26%, list=15%, signal=31% |
| 207 | NERVOUS\_SYSTEM\_DEVELOPMENT |  | 328 | -0.16 | -1.00 | 0.473 | 0.687 | 1.000 | 4207 | tags=35%, list=32%, signal=50% |
| 208 | FATTY\_ACID\_OXIDATION |  | 17 | -0.29 | -1.00 | 0.457 | 0.691 | 1.000 | 4240 | tags=53%, list=32%, signal=78% |
| 209 | PROTEIN\_SECRETION |  | 28 | -0.25 | -0.99 | 0.447 | 0.690 | 1.000 | 3504 | tags=36%, list=27%, signal=49% |
| 210 | MAPKKK\_CASCADE\_GO\_0000165 |  | 90 | -0.19 | -0.99 | 0.472 | 0.687 | 1.000 | 1875 | tags=20%, list=14%, signal=23% |
| 211 | AMINO\_ACID\_AND\_DERIVATIVE\_METABOLIC\_PROCESS |  | 96 | -0.19 | -0.99 | 0.465 | 0.695 | 1.000 | 2254 | tags=24%, list=17%, signal=29% |
| 212 | HEART\_DEVELOPMENT |  | 33 | -0.25 | -0.99 | 0.474 | 0.694 | 1.000 | 3539 | tags=36%, list=27%, signal=50% |
| 213 | POSITIVE\_REGULATION\_OF\_NUCLEOBASENUCLEOSIDENUCLEOTIDE\_AND\_NUCLEIC\_ACID\_METABOLIC\_PROCESS |  | 134 | -0.18 | -0.99 | 0.475 | 0.692 | 1.000 | 2964 | tags=27%, list=23%, signal=34% |
| 214 | CHEMICAL\_HOMEOSTASIS |  | 136 | -0.18 | -0.99 | 0.467 | 0.689 | 1.000 | 3087 | tags=28%, list=24%, signal=36% |
| 215 | POSITIVE\_REGULATION\_OF\_CELLULAR\_COMPONENT\_ORGANIZATION\_AND\_BIOGENESIS |  | 28 | -0.25 | -0.99 | 0.488 | 0.690 | 1.000 | 3699 | tags=39%, list=28%, signal=55% |
| 216 | TISSUE\_DEVELOPMENT |  | 126 | -0.18 | -0.99 | 0.483 | 0.689 | 1.000 | 3849 | tags=36%, list=29%, signal=50% |
| 217 | CELL\_MIGRATION |  | 82 | -0.19 | -0.99 | 0.488 | 0.688 | 1.000 | 2720 | tags=24%, list=21%, signal=31% |
| 218 | NEGATIVE\_REGULATION\_OF\_CELL\_DIFFERENTIATION |  | 24 | -0.26 | -0.98 | 0.477 | 0.707 | 1.000 | 2532 | tags=25%, list=19%, signal=31% |
| 219 | REGULATION\_OF\_MYELOID\_CELL\_DIFFERENTIATION |  | 19 | -0.28 | -0.98 | 0.490 | 0.704 | 1.000 | 2767 | tags=32%, list=21%, signal=40% |
| 220 | TRANSLATION |  | 149 | -0.17 | -0.97 | 0.534 | 0.709 | 1.000 | 3051 | tags=29%, list=23%, signal=37% |
| 221 | MACROMOLECULAR\_COMPLEX\_ASSEMBLY |  | 254 | -0.16 | -0.97 | 0.545 | 0.711 | 1.000 | 2720 | tags=24%, list=21%, signal=30% |
| 222 | REGULATION\_OF\_PHOSPHORYLATION |  | 42 | -0.22 | -0.96 | 0.511 | 0.734 | 1.000 | 2086 | tags=24%, list=16%, signal=28% |
| 223 | AMINE\_CATABOLIC\_PROCESS |  | 25 | -0.25 | -0.96 | 0.506 | 0.736 | 1.000 | 2219 | tags=28%, list=17%, signal=34% |
| 224 | SKELETAL\_MUSCLE\_DEVELOPMENT |  | 28 | -0.24 | -0.96 | 0.531 | 0.745 | 1.000 | 3387 | tags=43%, list=26%, signal=58% |
| 225 | ANATOMICAL\_STRUCTURE\_MORPHOGENESIS |  | 336 | -0.15 | -0.95 | 0.612 | 0.748 | 1.000 | 3294 | tags=28%, list=25%, signal=36% |
| 226 | CELL\_CELL\_ADHESION |  | 72 | -0.19 | -0.95 | 0.543 | 0.750 | 1.000 | 4872 | tags=50%, list=37%, signal=79% |
| 227 | HOMEOSTATIC\_PROCESS |  | 179 | -0.16 | -0.95 | 0.592 | 0.757 | 1.000 | 3087 | tags=27%, list=24%, signal=35% |
| 228 | NITROGEN\_COMPOUND\_CATABOLIC\_PROCESS |  | 27 | -0.25 | -0.95 | 0.500 | 0.754 | 1.000 | 2219 | tags=26%, list=17%, signal=31% |
| 229 | REGULATION\_OF\_BINDING |  | 46 | -0.21 | -0.94 | 0.506 | 0.758 | 1.000 | 2490 | tags=28%, list=19%, signal=35% |
| 230 | RESPONSE\_TO\_NUTRIENT |  | 17 | -0.28 | -0.93 | 0.540 | 0.785 | 1.000 | 2246 | tags=29%, list=17%, signal=35% |
| 231 | SULFUR\_METABOLIC\_PROCESS |  | 30 | -0.24 | -0.93 | 0.561 | 0.795 | 1.000 | 2831 | tags=30%, list=22%, signal=38% |
| 232 | POSITIVE\_REGULATION\_OF\_TRANSCRIPTION\_FROM\_RNA\_POLYMERASE\_II\_PROMOTER |  | 60 | -0.19 | -0.93 | 0.588 | 0.793 | 1.000 | 2850 | tags=28%, list=22%, signal=36% |
| 233 | REGULATION\_OF\_G\_PROTEIN\_COUPLED\_RECEPTOR\_PROTEIN\_SIGNALING\_PATHWAY |  | 23 | -0.25 | -0.92 | 0.570 | 0.815 | 1.000 | 1123 | tags=17%, list=9%, signal=19% |
| 234 | SODIUM\_ION\_TRANSPORT |  | 17 | -0.27 | -0.92 | 0.555 | 0.811 | 1.000 | 9524 | tags=100%, list=73%, signal=367% |
| 235 | VESICLE\_MEDIATED\_TRANSPORT |  | 174 | -0.15 | -0.91 | 0.688 | 0.819 | 1.000 | 3747 | tags=33%, list=29%, signal=45% |
| 236 | AXON\_GUIDANCE |  | 18 | -0.27 | -0.90 | 0.595 | 0.845 | 1.000 | 3032 | tags=33%, list=23%, signal=43% |
| 237 | POSITIVE\_REGULATION\_OF\_CASPASE\_ACTIVITY |  | 28 | -0.23 | -0.90 | 0.619 | 0.855 | 1.000 | 1836 | tags=25%, list=14%, signal=29% |
| 238 | REGULATION\_OF\_CELLULAR\_COMPONENT\_ORGANIZATION\_AND\_BIOGENESIS |  | 102 | -0.17 | -0.90 | 0.709 | 0.852 | 1.000 | 3699 | tags=32%, list=28%, signal=45% |
| 239 | REGULATION\_OF\_PROTEIN\_SECRETION |  | 19 | -0.26 | -0.90 | 0.617 | 0.849 | 1.000 | 2763 | tags=32%, list=21%, signal=40% |
| 240 | GOLGI\_VESICLE\_TRANSPORT |  | 42 | -0.20 | -0.90 | 0.622 | 0.846 | 1.000 | 4001 | tags=40%, list=31%, signal=58% |
| 241 | CENTRAL\_NERVOUS\_SYSTEM\_DEVELOPMENT |  | 105 | -0.17 | -0.90 | 0.712 | 0.845 | 1.000 | 4327 | tags=39%, list=33%, signal=58% |
| 242 | REGULATION\_OF\_TRANSLATIONAL\_INITIATION |  | 25 | -0.24 | -0.89 | 0.620 | 0.847 | 1.000 | 1140 | tags=20%, list=9%, signal=22% |
| 243 | CYCLIC\_NUCLEOTIDE\_MEDIATED\_SIGNALING |  | 97 | -0.17 | -0.89 | 0.690 | 0.844 | 1.000 | 1207 | tags=12%, list=9%, signal=14% |
| 244 | SECRETION\_BY\_CELL |  | 100 | -0.17 | -0.89 | 0.704 | 0.841 | 1.000 | 4489 | tags=41%, list=34%, signal=62% |
| 245 | ACTIVATION\_OF\_PROTEIN\_KINASE\_ACTIVITY |  | 23 | -0.24 | -0.89 | 0.581 | 0.848 | 1.000 | 4591 | tags=39%, list=35%, signal=60% |
| 246 | REGULATION\_OF\_PROTEIN\_MODIFICATION\_PROCESS |  | 37 | -0.21 | -0.89 | 0.634 | 0.848 | 1.000 | 2455 | tags=24%, list=19%, signal=30% |
| 247 | RESPONSE\_TO\_OXIDATIVE\_STRESS |  | 38 | -0.21 | -0.88 | 0.699 | 0.873 | 1.000 | 1635 | tags=24%, list=12%, signal=27% |
| 248 | EPIDERMIS\_DEVELOPMENT |  | 66 | -0.18 | -0.87 | 0.700 | 0.872 | 1.000 | 1146 | tags=17%, list=9%, signal=18% |
| 249 | AEROBIC\_RESPIRATION |  | 15 | -0.27 | -0.87 | 0.665 | 0.870 | 1.000 | 825 | tags=20%, list=6%, signal=21% |
| 250 | G\_PROTEIN\_SIGNALING\_COUPLED\_TO\_CYCLIC\_NUCLEOTIDE\_SECOND\_MESSENGER |  | 96 | -0.17 | -0.87 | 0.722 | 0.868 | 1.000 | 1207 | tags=13%, list=9%, signal=14% |
| 251 | T\_CELL\_PROLIFERATION |  | 17 | -0.26 | -0.87 | 0.630 | 0.867 | 1.000 | 2786 | tags=35%, list=21%, signal=45% |
| 252 | CELL\_CYCLE\_ARREST\_GO\_0007050 |  | 52 | -0.19 | -0.87 | 0.693 | 0.870 | 1.000 | 2163 | tags=25%, list=17%, signal=30% |
| 253 | REGULATION\_OF\_GROWTH |  | 48 | -0.20 | -0.87 | 0.695 | 0.869 | 1.000 | 4190 | tags=40%, list=32%, signal=58% |
| 254 | CELLULAR\_MORPHOGENESIS\_DURING\_DIFFERENTIATION |  | 38 | -0.20 | -0.87 | 0.671 | 0.868 | 1.000 | 3709 | tags=32%, list=28%, signal=44% |
| 255 | REGULATION\_OF\_TRANSCRIPTION\_FACTOR\_ACTIVITY |  | 30 | -0.22 | -0.86 | 0.672 | 0.871 | 1.000 | 4301 | tags=47%, list=33%, signal=69% |
| 256 | RESPONSE\_TO\_NUTRIENT\_LEVELS |  | 27 | -0.22 | -0.86 | 0.638 | 0.871 | 1.000 | 2465 | tags=26%, list=19%, signal=32% |
| 257 | AMINE\_METABOLIC\_PROCESS |  | 128 | -0.16 | -0.86 | 0.767 | 0.871 | 1.000 | 3292 | tags=28%, list=25%, signal=37% |
| 258 | PHOSPHOINOSITIDE\_METABOLIC\_PROCESS |  | 25 | -0.23 | -0.86 | 0.675 | 0.872 | 1.000 | 4522 | tags=52%, list=35%, signal=79% |
| 259 | POSITIVE\_REGULATION\_OF\_BINDING |  | 19 | -0.25 | -0.86 | 0.655 | 0.876 | 1.000 | 4731 | tags=58%, list=36%, signal=91% |
| 260 | ENDOSOME\_TRANSPORT |  | 22 | -0.23 | -0.85 | 0.665 | 0.879 | 1.000 | 3924 | tags=41%, list=30%, signal=58% |
| 261 | REGULATION\_OF\_TRANSLATION |  | 76 | -0.17 | -0.85 | 0.776 | 0.876 | 1.000 | 2490 | tags=24%, list=19%, signal=29% |
| 262 | EXTRACELLULAR\_STRUCTURE\_ORGANIZATION\_AND\_BIOGENESIS |  | 23 | -0.23 | -0.85 | 0.668 | 0.881 | 1.000 | 3387 | tags=39%, list=26%, signal=53% |
| 263 | CELL\_CELL\_SIGNALING |  | 372 | -0.13 | -0.85 | 0.926 | 0.879 | 1.000 | 4203 | tags=34%, list=32%, signal=48% |
| 264 | SPHINGOLIPID\_METABOLIC\_PROCESS |  | 23 | -0.23 | -0.84 | 0.718 | 0.892 | 1.000 | 3247 | tags=35%, list=25%, signal=46% |
| 265 | CARBOHYDRATE\_BIOSYNTHETIC\_PROCESS |  | 35 | -0.20 | -0.84 | 0.740 | 0.892 | 1.000 | 4026 | tags=37%, list=31%, signal=53% |
| 266 | REGULATION\_OF\_DNA\_BINDING |  | 36 | -0.19 | -0.83 | 0.739 | 0.913 | 1.000 | 2490 | tags=28%, list=19%, signal=34% |
| 267 | POSITIVE\_REGULATION\_OF\_JNK\_ACTIVITY |  | 16 | -0.24 | -0.81 | 0.730 | 0.945 | 1.000 | 1749 | tags=25%, list=13%, signal=29% |
| 268 | SECOND\_MESSENGER\_MEDIATED\_SIGNALING |  | 139 | -0.14 | -0.80 | 0.932 | 0.955 | 1.000 | 2062 | tags=16%, list=16%, signal=19% |
| 269 | G\_PROTEIN\_COUPLED\_RECEPTOR\_PROTEIN\_SIGNALING\_PATHWAY |  | 300 | -0.13 | -0.79 | 0.972 | 0.965 | 1.000 | 4606 | tags=36%, list=35%, signal=54% |
| 270 | POSITIVE\_REGULATION\_OF\_TRANSCRIPTIONDNA\_DEPENDENT |  | 105 | -0.15 | -0.79 | 0.917 | 0.963 | 1.000 | 4486 | tags=39%, list=34%, signal=59% |
| 271 | INSULIN\_RECEPTOR\_SIGNALING\_PATHWAY |  | 16 | -0.23 | -0.79 | 0.759 | 0.961 | 1.000 | 3078 | tags=31%, list=24%, signal=41% |
| 272 | G\_PROTEIN\_SIGNALING\_COUPLED\_TO\_IP3\_SECOND\_MESSENGERPHOSPHOLIPASE\_C\_ACTIVATING |  | 39 | -0.19 | -0.79 | 0.840 | 0.959 | 1.000 | 2506 | tags=23%, list=19%, signal=28% |
| 273 | AMINE\_BIOSYNTHETIC\_PROCESS |  | 15 | -0.24 | -0.79 | 0.743 | 0.958 | 1.000 | 332 | tags=13%, list=3%, signal=14% |
| 274 | NEGATIVE\_REGULATION\_OF\_CELLULAR\_COMPONENT\_ORGANIZATION\_AND\_BIOGENESIS |  | 26 | -0.20 | -0.78 | 0.829 | 0.965 | 1.000 | 1381 | tags=15%, list=11%, signal=17% |
| 275 | RESPONSE\_TO\_EXTRACELLULAR\_STIMULUS |  | 29 | -0.20 | -0.78 | 0.847 | 0.964 | 1.000 | 2465 | tags=24%, list=19%, signal=30% |
| 276 | REPRODUCTIVE\_PROCESS |  | 133 | -0.14 | -0.78 | 0.931 | 0.961 | 1.000 | 3842 | tags=34%, list=29%, signal=47% |
| 277 | NEGATIVE\_REGULATION\_OF\_CELLULAR\_PROTEIN\_METABOLIC\_PROCESS |  | 41 | -0.17 | -0.76 | 0.862 | 0.977 | 1.000 | 2832 | tags=24%, list=22%, signal=31% |
| 278 | BRAIN\_DEVELOPMENT |  | 39 | -0.18 | -0.76 | 0.839 | 0.975 | 1.000 | 3907 | tags=38%, list=30%, signal=55% |
| 279 | PROTEIN\_LOCALIZATION |  | 184 | -0.13 | -0.76 | 0.971 | 0.976 | 1.000 | 3556 | tags=27%, list=27%, signal=37% |
| 280 | NEGATIVE\_REGULATION\_OF\_GROWTH |  | 35 | -0.18 | -0.76 | 0.850 | 0.974 | 1.000 | 4190 | tags=40%, list=32%, signal=59% |
| 281 | REGULATION\_OF\_MUSCLE\_CONTRACTION |  | 18 | -0.22 | -0.75 | 0.805 | 0.975 | 1.000 | 2631 | tags=33%, list=20%, signal=42% |
| 282 | POSITIVE\_REGULATION\_OF\_RNA\_METABOLIC\_PROCESS |  | 107 | -0.14 | -0.74 | 0.955 | 0.985 | 1.000 | 4486 | tags=38%, list=34%, signal=58% |
| 283 | METAL\_ION\_TRANSPORT |  | 102 | -0.14 | -0.74 | 0.947 | 0.985 | 1.000 | 4664 | tags=40%, list=36%, signal=62% |
| 284 | G\_PROTEIN\_SIGNALING\_ADENYLATE\_CYCLASE\_ACTIVATING\_PATHWAY |  | 24 | -0.19 | -0.73 | 0.861 | 0.996 | 1.000 | 1207 | tags=13%, list=9%, signal=14% |
| 285 | POSITIVE\_REGULATION\_OF\_TRANSPORT |  | 18 | -0.21 | -0.73 | 0.841 | 0.993 | 1.000 | 4891 | tags=56%, list=37%, signal=89% |
| 286 | PROTEIN\_POLYMERIZATION |  | 17 | -0.21 | -0.72 | 0.854 | 0.993 | 1.000 | 418 | tags=12%, list=3%, signal=12% |
| 287 | CARBOHYDRATE\_METABOLIC\_PROCESS |  | 152 | -0.13 | -0.72 | 0.986 | 0.990 | 1.000 | 4061 | tags=32%, list=31%, signal=46% |
| 288 | POTASSIUM\_ION\_TRANSPORT |  | 52 | -0.16 | -0.72 | 0.911 | 0.987 | 1.000 | 4664 | tags=40%, list=36%, signal=62% |
| 289 | CARBOHYDRATE\_CATABOLIC\_PROCESS |  | 20 | -0.20 | -0.71 | 0.877 | 0.994 | 1.000 | 4026 | tags=35%, list=31%, signal=50% |
| 290 | CELLULAR\_CARBOHYDRATE\_CATABOLIC\_PROCESS |  | 20 | -0.20 | -0.71 | 0.850 | 0.991 | 1.000 | 4026 | tags=35%, list=31%, signal=50% |
| 291 | CATION\_TRANSPORT |  | 130 | -0.13 | -0.71 | 0.984 | 0.990 | 1.000 | 4664 | tags=40%, list=36%, signal=62% |
| 292 | NEGATIVE\_REGULATION\_OF\_PROTEIN\_METABOLIC\_PROCESS |  | 44 | -0.16 | -0.71 | 0.936 | 0.988 | 1.000 | 2832 | tags=23%, list=22%, signal=29% |
| 293 | SECRETORY\_PATHWAY |  | 72 | -0.14 | -0.71 | 0.951 | 0.987 | 1.000 | 4489 | tags=40%, list=34%, signal=61% |
| 294 | REGULATION\_OF\_CYTOKINE\_PRODUCTION |  | 21 | -0.19 | -0.70 | 0.873 | 0.985 | 1.000 | 3090 | tags=29%, list=24%, signal=37% |
| 295 | ESTABLISHMENT\_AND\_OR\_MAINTENANCE\_OF\_CELL\_POLARITY |  | 19 | -0.20 | -0.70 | 0.874 | 0.982 | 1.000 | 1481 | tags=16%, list=11%, signal=18% |
| 296 | DI\_\_\_TRI\_VALENT\_INORGANIC\_CATION\_TRANSPORT |  | 27 | -0.18 | -0.70 | 0.906 | 0.979 | 1.000 | 1114 | tags=15%, list=9%, signal=16% |
| 297 | PHOSPHOINOSITIDE\_MEDIATED\_SIGNALING |  | 42 | -0.16 | -0.70 | 0.906 | 0.976 | 1.000 | 2506 | tags=21%, list=19%, signal=26% |
| 298 | ION\_TRANSPORT |  | 165 | -0.12 | -0.70 | 0.995 | 0.973 | 1.000 | 4670 | tags=39%, list=36%, signal=60% |
| 299 | REGULATION\_OF\_CELL\_MIGRATION |  | 23 | -0.19 | -0.70 | 0.885 | 0.973 | 1.000 | 4852 | tags=43%, list=37%, signal=69% |
| 300 | SECRETION |  | 157 | -0.12 | -0.68 | 0.993 | 0.981 | 1.000 | 4792 | tags=41%, list=37%, signal=64% |
| 301 | NEGATIVE\_REGULATION\_OF\_MULTICELLULAR\_ORGANISMAL\_PROCESS |  | 27 | -0.18 | -0.68 | 0.897 | 0.984 | 1.000 | 2105 | tags=22%, list=16%, signal=26% |
| 302 | EXCRETION |  | 35 | -0.16 | -0.66 | 0.932 | 0.993 | 1.000 | 2317 | tags=20%, list=18%, signal=24% |
| 303 | INORGANIC\_ANION\_TRANSPORT |  | 16 | -0.20 | -0.65 | 0.918 | 0.994 | 1.000 | 582 | tags=13%, list=4%, signal=13% |
| 304 | PATTERN\_SPECIFICATION\_PROCESS |  | 27 | -0.16 | -0.65 | 0.947 | 0.991 | 1.000 | 959 | tags=11%, list=7%, signal=12% |
| 305 | LIPID\_HOMEOSTASIS |  | 15 | -0.20 | -0.64 | 0.921 | 0.991 | 1.000 | 917 | tags=13%, list=7%, signal=14% |
| 306 | NUCLEOTIDE\_EXCISION\_REPAIR |  | 19 | -0.18 | -0.62 | 0.950 | 0.998 | 1.000 | 2283 | tags=21%, list=17%, signal=25% |
| 307 | DETECTION\_OF\_ABIOTIC\_STIMULUS |  | 16 | -0.18 | -0.59 | 0.953 | 1.000 | 1.000 | 5167 | tags=50%, list=39%, signal=83% |
| 308 | AMINO\_SUGAR\_METABOLIC\_PROCESS |  | 15 | -0.18 | -0.58 | 0.952 | 1.000 | 1.000 | 4362 | tags=40%, list=33%, signal=60% |
| 309 | REGULATION\_OF\_CELL\_GROWTH |  | 39 | -0.13 | -0.56 | 0.993 | 1.000 | 1.000 | 4190 | tags=33%, list=32%, signal=49% |
| 310 | CALCIUM\_ION\_TRANSPORT |  | 23 | -0.15 | -0.56 | 0.968 | 1.000 | 1.000 | 1114 | tags=13%, list=9%, signal=14% |
| 311 | RESPONSE\_TO\_LIGHT\_STIMULUS |  | 40 | -0.13 | -0.55 | 0.988 | 1.000 | 1.000 | 2854 | tags=23%, list=22%, signal=29% |
| 312 | FEMALE\_GAMETE\_GENERATION |  | 15 | -0.17 | -0.53 | 0.987 | 1.000 | 1.000 | 10913 | tags=100%, list=83%, signal=600% |
| 313 | REGULATION\_OF\_ACTION\_POTENTIAL |  | 16 | -0.15 | -0.49 | 0.996 | 1.000 | 1.000 | 3635 | tags=31%, list=28%, signal=43% |
| 314 | MONOVALENT\_INORGANIC\_CATION\_TRANSPORT |  | 83 | -0.09 | -0.47 | 1.000 | 0.998 | 1.000 | 4664 | tags=36%, list=36%, signal=56% |
Table: Gene sets enriched in phenotype **na**[plain text format]****

  
