# Supplementary material for: Multiple-input multiple-output causal strategies for gene selection
Source: BMC Bioinformatics. 2011 Nov 25;12:458. doi: 10.1186/1471-2105-12-458 (PMC3323860; doi:10.1186/1471-2105-12-458)
Supplement: Additional file 3 — Archive containing the output files computed by the preranked GSEA for λ ∈ {0.6,0.7,0.8,0.9,1.0,2.0} (GSEA_MIMO_part2.zip). [file 1471-2105-12-458-S3.ZIP › mFS09_entrez_mimo.GseaPreranked.1316039282297/gsea_report_for_na_pos_1316039282297.html]

Report for na\_pos 1316039282297 [GSEA]

| GS  follow link to MSigDB | GS DETAILS | SIZE | ES | NES | NOM p-val | FDR q-val | FWER p-val | RANK AT MAX | LEADING EDGE || 1 | CELL\_CYCLE\_PROCESS |  | 169 | 0.49 | 2.68 | 0.000 | 0.000 | 0.000 | 2330 | tags=47%, list=18%, signal=56% |
| 2 | MITOTIC\_CELL\_CYCLE |  | 134 | 0.50 | 2.67 | 0.000 | 0.000 | 0.000 | 2330 | tags=48%, list=18%, signal=58% |
| 3 | M\_PHASE |  | 98 | 0.53 | 2.65 | 0.000 | 0.000 | 0.000 | 2324 | tags=51%, list=18%, signal=62% |
| 4 | MITOSIS |  | 70 | 0.55 | 2.63 | 0.000 | 0.000 | 0.000 | 1470 | tags=47%, list=11%, signal=53% |
| 5 | M\_PHASE\_OF\_MITOTIC\_CELL\_CYCLE |  | 72 | 0.56 | 2.63 | 0.000 | 0.000 | 0.000 | 2324 | tags=54%, list=18%, signal=65% |
| 6 | CELL\_CYCLE\_PHASE |  | 152 | 0.46 | 2.52 | 0.000 | 0.000 | 0.000 | 2330 | tags=45%, list=18%, signal=54% |
| 7 | DNA\_REPLICATION |  | 97 | 0.48 | 2.44 | 0.000 | 0.000 | 0.001 | 2914 | tags=47%, list=22%, signal=61% |
| 8 | DNA\_METABOLIC\_PROCESS |  | 240 | 0.41 | 2.41 | 0.000 | 0.000 | 0.001 | 3249 | tags=47%, list=25%, signal=61% |
| 9 | CELL\_CYCLE\_GO\_0007049 |  | 277 | 0.41 | 2.40 | 0.000 | 0.000 | 0.001 | 2364 | tags=40%, list=18%, signal=48% |
| 10 | SISTER\_CHROMATID\_SEGREGATION |  | 16 | 0.73 | 2.37 | 0.000 | 0.000 | 0.001 | 959 | tags=56%, list=7%, signal=61% |
| 11 | MITOTIC\_SISTER\_CHROMATID\_SEGREGATION |  | 15 | 0.75 | 2.37 | 0.000 | 0.000 | 0.001 | 959 | tags=60%, list=7%, signal=65% |
| 12 | CELL\_CYCLE\_CHECKPOINT\_GO\_0000075 |  | 45 | 0.53 | 2.32 | 0.000 | 0.000 | 0.001 | 1976 | tags=53%, list=15%, signal=63% |
| 13 | DNA\_REPAIR |  | 118 | 0.44 | 2.29 | 0.000 | 0.000 | 0.002 | 2963 | tags=48%, list=23%, signal=62% |
| 14 | DNA\_DEPENDENT\_DNA\_REPLICATION |  | 52 | 0.50 | 2.27 | 0.000 | 0.000 | 0.004 | 3216 | tags=56%, list=25%, signal=74% |
| 15 | REGULATION\_OF\_MITOSIS |  | 33 | 0.57 | 2.27 | 0.000 | 0.000 | 0.004 | 1029 | tags=45%, list=8%, signal=49% |
| 16 | CHROMOSOME\_SEGREGATION |  | 28 | 0.58 | 2.26 | 0.000 | 0.000 | 0.004 | 959 | tags=46%, list=7%, signal=50% |
| 17 | RESPONSE\_TO\_DNA\_DAMAGE\_STIMULUS |  | 153 | 0.41 | 2.22 | 0.000 | 0.000 | 0.009 | 2963 | tags=46%, list=23%, signal=58% |
| 18 | RNA\_SPLICING |  | 74 | 0.46 | 2.21 | 0.000 | 0.000 | 0.009 | 3386 | tags=53%, list=26%, signal=71% |
| 19 | RESPONSE\_TO\_ENDOGENOUS\_STIMULUS |  | 182 | 0.38 | 2.17 | 0.000 | 0.001 | 0.017 | 3338 | tags=46%, list=25%, signal=61% |
| 20 | NUCLEOTIDE\_BIOSYNTHETIC\_PROCESS |  | 17 | 0.65 | 2.15 | 0.000 | 0.001 | 0.024 | 1359 | tags=53%, list=10%, signal=59% |
| 21 | RNA\_PROCESSING |  | 138 | 0.39 | 2.10 | 0.000 | 0.001 | 0.038 | 3299 | tags=49%, list=25%, signal=64% |
| 22 | MICROTUBULE\_CYTOSKELETON\_ORGANIZATION\_AND\_BIOGENESIS |  | 31 | 0.53 | 2.08 | 0.000 | 0.002 | 0.051 | 2713 | tags=55%, list=21%, signal=69% |
| 23 | DNA\_INTEGRITY\_CHECKPOINT |  | 22 | 0.59 | 2.08 | 0.000 | 0.002 | 0.052 | 1962 | tags=59%, list=15%, signal=69% |
| 24 | DNA\_REPLICATION\_INITIATION |  | 15 | 0.66 | 2.07 | 0.000 | 0.002 | 0.057 | 2644 | tags=80%, list=20%, signal=100% |
| 25 | MITOTIC\_CELL\_CYCLE\_CHECKPOINT |  | 19 | 0.58 | 2.02 | 0.004 | 0.004 | 0.118 | 1976 | tags=53%, list=15%, signal=62% |
| 26 | MITOCHONDRION\_ORGANIZATION\_AND\_BIOGENESIS |  | 42 | 0.47 | 2.00 | 0.000 | 0.004 | 0.128 | 3234 | tags=50%, list=25%, signal=66% |
| 27 | PROTEIN\_FOLDING |  | 55 | 0.44 | 2.00 | 0.000 | 0.004 | 0.129 | 3206 | tags=51%, list=24%, signal=67% |
| 28 | DOUBLE\_STRAND\_BREAK\_REPAIR |  | 21 | 0.56 | 1.98 | 0.004 | 0.005 | 0.155 | 1962 | tags=52%, list=15%, signal=62% |
| 29 | REGULATION\_OF\_MITOTIC\_CELL\_CYCLE |  | 19 | 0.57 | 1.98 | 0.002 | 0.005 | 0.162 | 1497 | tags=47%, list=11%, signal=53% |
| 30 | REGULATION\_OF\_CELL\_CYCLE |  | 161 | 0.36 | 1.98 | 0.000 | 0.005 | 0.163 | 1976 | tags=35%, list=15%, signal=40% |
| 31 | MRNA\_METABOLIC\_PROCESS |  | 72 | 0.41 | 1.97 | 0.000 | 0.005 | 0.168 | 3299 | tags=50%, list=25%, signal=66% |
| 32 | COENZYME\_METABOLIC\_PROCESS |  | 35 | 0.48 | 1.95 | 0.002 | 0.006 | 0.210 | 3338 | tags=49%, list=25%, signal=65% |
| 33 | TRNA\_METABOLIC\_PROCESS |  | 15 | 0.61 | 1.94 | 0.004 | 0.006 | 0.226 | 2576 | tags=67%, list=20%, signal=83% |
| 34 | INTERPHASE\_OF\_MITOTIC\_CELL\_CYCLE |  | 57 | 0.42 | 1.93 | 0.000 | 0.007 | 0.259 | 3349 | tags=51%, list=26%, signal=68% |
| 35 | NUCLEOBASENUCLEOSIDENUCLEOTIDE\_AND\_NUCLEIC\_ACID\_TRANSPORT |  | 26 | 0.50 | 1.92 | 0.002 | 0.007 | 0.265 | 2241 | tags=46%, list=17%, signal=56% |
| 36 | INTERPHASE |  | 63 | 0.41 | 1.91 | 0.000 | 0.008 | 0.310 | 3349 | tags=49%, list=26%, signal=66% |
| 37 | COFACTOR\_BIOSYNTHETIC\_PROCESS |  | 21 | 0.53 | 1.88 | 0.002 | 0.011 | 0.397 | 1476 | tags=38%, list=11%, signal=43% |
| 38 | REGULATION\_OF\_DNA\_REPLICATION |  | 18 | 0.55 | 1.87 | 0.009 | 0.012 | 0.441 | 1962 | tags=50%, list=15%, signal=59% |
| 39 | MRNA\_PROCESSING\_GO\_0006397 |  | 61 | 0.41 | 1.86 | 0.002 | 0.011 | 0.441 | 3104 | tags=46%, list=24%, signal=60% |
| 40 | REGULATION\_OF\_CYCLIN\_DEPENDENT\_PROTEIN\_KINASE\_ACTIVITY |  | 40 | 0.44 | 1.86 | 0.005 | 0.011 | 0.447 | 2428 | tags=48%, list=19%, signal=58% |
| 41 | CHROMOSOME\_ORGANIZATION\_AND\_BIOGENESIS |  | 107 | 0.36 | 1.83 | 0.000 | 0.014 | 0.537 | 2764 | tags=38%, list=21%, signal=48% |
| 42 | REGULATION\_OF\_DNA\_METABOLIC\_PROCESS |  | 40 | 0.43 | 1.83 | 0.002 | 0.014 | 0.548 | 2134 | tags=45%, list=16%, signal=54% |
| 43 | MITOCHONDRIAL\_TRANSPORT |  | 18 | 0.54 | 1.82 | 0.002 | 0.015 | 0.568 | 1476 | tags=44%, list=11%, signal=50% |
| 44 | TRANSCRIPTION\_INITIATION\_FROM\_RNA\_POLYMERASE\_II\_PROMOTER |  | 27 | 0.49 | 1.82 | 0.006 | 0.015 | 0.576 | 2352 | tags=44%, list=18%, signal=54% |
| 45 | DNA\_DAMAGE\_CHECKPOINT |  | 19 | 0.53 | 1.80 | 0.004 | 0.017 | 0.632 | 1962 | tags=53%, list=15%, signal=62% |
| 46 | COFACTOR\_METABOLIC\_PROCESS |  | 51 | 0.40 | 1.78 | 0.002 | 0.019 | 0.670 | 3395 | tags=43%, list=26%, signal=58% |
| 47 | G1\_S\_TRANSITION\_OF\_MITOTIC\_CELL\_CYCLE |  | 23 | 0.48 | 1.78 | 0.000 | 0.019 | 0.690 | 3264 | tags=52%, list=25%, signal=69% |
| 48 | NUCLEAR\_EXPORT |  | 26 | 0.47 | 1.73 | 0.007 | 0.028 | 0.812 | 4261 | tags=58%, list=33%, signal=85% |
| 49 | DNA\_DAMAGE\_RESPONSESIGNAL\_TRANSDUCTION |  | 34 | 0.44 | 1.73 | 0.002 | 0.028 | 0.820 | 1962 | tags=44%, list=15%, signal=52% |
| 50 | PROTEIN\_MODIFICATION\_BY\_SMALL\_PROTEIN\_CONJUGATION |  | 35 | 0.43 | 1.73 | 0.004 | 0.027 | 0.821 | 2496 | tags=40%, list=19%, signal=49% |
| 51 | BIOPOLYMER\_CATABOLIC\_PROCESS |  | 103 | 0.34 | 1.72 | 0.000 | 0.029 | 0.850 | 3543 | tags=43%, list=27%, signal=58% |
| 52 | RNA\_EXPORT\_FROM\_NUCLEUS |  | 17 | 0.51 | 1.71 | 0.020 | 0.031 | 0.881 | 4611 | tags=71%, list=35%, signal=109% |
| 53 | UBIQUITIN\_CYCLE |  | 40 | 0.41 | 1.69 | 0.011 | 0.034 | 0.908 | 2496 | tags=38%, list=19%, signal=46% |
| 54 | PROTEIN\_CATABOLIC\_PROCESS |  | 60 | 0.37 | 1.66 | 0.009 | 0.043 | 0.951 | 3543 | tags=42%, list=27%, signal=57% |
| 55 | PROTEIN\_UBIQUITINATION |  | 32 | 0.41 | 1.66 | 0.014 | 0.044 | 0.959 | 2496 | tags=38%, list=19%, signal=46% |
| 56 | CYTOKINESIS |  | 17 | 0.50 | 1.65 | 0.023 | 0.044 | 0.960 | 993 | tags=35%, list=8%, signal=38% |
| 57 | MACROMOLECULE\_CATABOLIC\_PROCESS |  | 120 | 0.31 | 1.64 | 0.003 | 0.047 | 0.971 | 3543 | tags=39%, list=27%, signal=53% |
| 58 | CELLULAR\_PROTEIN\_CATABOLIC\_PROCESS |  | 50 | 0.37 | 1.62 | 0.018 | 0.053 | 0.983 | 3543 | tags=42%, list=27%, signal=57% |
| 59 | BASE\_EXCISION\_REPAIR |  | 16 | 0.49 | 1.62 | 0.019 | 0.054 | 0.986 | 2853 | tags=44%, list=22%, signal=56% |
| 60 | PROTEIN\_DNA\_COMPLEX\_ASSEMBLY |  | 45 | 0.37 | 1.62 | 0.010 | 0.053 | 0.986 | 2697 | tags=40%, list=21%, signal=50% |
| 61 | TRANSCRIPTION\_INITIATION |  | 33 | 0.40 | 1.58 | 0.029 | 0.067 | 0.998 | 2352 | tags=39%, list=18%, signal=48% |
| 62 | NUCLEAR\_TRANSPORT |  | 77 | 0.32 | 1.57 | 0.007 | 0.074 | 0.998 | 4261 | tags=47%, list=33%, signal=69% |
| 63 | DNA\_PACKAGING |  | 29 | 0.40 | 1.55 | 0.033 | 0.080 | 0.999 | 2935 | tags=45%, list=22%, signal=58% |
| 64 | DNA\_RECOMBINATION |  | 45 | 0.36 | 1.55 | 0.020 | 0.082 | 0.999 | 1797 | tags=33%, list=14%, signal=39% |
| 65 | NUCLEOCYTOPLASMIC\_TRANSPORT |  | 77 | 0.32 | 1.55 | 0.014 | 0.082 | 0.999 | 4261 | tags=47%, list=33%, signal=69% |
| 66 | MEIOTIC\_CELL\_CYCLE |  | 31 | 0.40 | 1.54 | 0.026 | 0.086 | 0.999 | 2713 | tags=42%, list=21%, signal=53% |
| 67 | CELL\_DIVISION |  | 19 | 0.45 | 1.53 | 0.044 | 0.086 | 0.999 | 993 | tags=32%, list=8%, signal=34% |
| 68 | CELLULAR\_MACROMOLECULE\_CATABOLIC\_PROCESS |  | 90 | 0.30 | 1.53 | 0.015 | 0.090 | 1.000 | 2241 | tags=29%, list=17%, signal=35% |
| 69 | NUCLEOTIDE\_METABOLIC\_PROCESS |  | 36 | 0.38 | 1.53 | 0.038 | 0.089 | 1.000 | 2520 | tags=39%, list=19%, signal=48% |
| 70 | ONE\_CARBON\_COMPOUND\_METABOLIC\_PROCESS |  | 24 | 0.42 | 1.51 | 0.037 | 0.097 | 1.000 | 3914 | tags=58%, list=30%, signal=83% |
| 71 | MICROTUBULE\_BASED\_PROCESS |  | 75 | 0.32 | 1.51 | 0.012 | 0.096 | 1.000 | 2866 | tags=36%, list=22%, signal=46% |
| 72 | ORGANELLE\_ORGANIZATION\_AND\_BIOGENESIS |  | 407 | 0.24 | 1.50 | 0.000 | 0.098 | 1.000 | 3202 | tags=33%, list=24%, signal=42% |
| 73 | NUCLEOBASENUCLEOSIDE\_AND\_NUCLEOTIDE\_METABOLIC\_PROCESS |  | 46 | 0.35 | 1.50 | 0.014 | 0.101 | 1.000 | 1359 | tags=28%, list=10%, signal=31% |
| 74 | MEIOSIS\_I |  | 19 | 0.43 | 1.48 | 0.043 | 0.111 | 1.000 | 1797 | tags=37%, list=14%, signal=43% |
| 75 | CELLULAR\_COMPONENT\_DISASSEMBLY |  | 31 | 0.38 | 1.48 | 0.044 | 0.111 | 1.000 | 3046 | tags=42%, list=23%, signal=55% |
| 76 | ESTABLISHMENT\_OF\_ORGANELLE\_LOCALIZATION |  | 16 | 0.46 | 1.45 | 0.064 | 0.128 | 1.000 | 901 | tags=38%, list=7%, signal=40% |
| 77 | APOPTOTIC\_NUCLEAR\_CHANGES |  | 17 | 0.43 | 1.44 | 0.074 | 0.135 | 1.000 | 1438 | tags=35%, list=11%, signal=40% |
| 78 | VIRAL\_INFECTIOUS\_CYCLE |  | 29 | 0.37 | 1.42 | 0.065 | 0.153 | 1.000 | 1645 | tags=34%, list=13%, signal=39% |
| 79 | ORGANELLE\_LOCALIZATION |  | 21 | 0.40 | 1.42 | 0.086 | 0.152 | 1.000 | 901 | tags=29%, list=7%, signal=31% |
| 80 | CHROMATIN\_ASSEMBLY\_OR\_DISASSEMBLY |  | 25 | 0.38 | 1.40 | 0.084 | 0.167 | 1.000 | 2935 | tags=48%, list=22%, signal=62% |
| 81 | NEGATIVE\_REGULATION\_OF\_DNA\_METABOLIC\_PROCESS |  | 16 | 0.42 | 1.39 | 0.096 | 0.179 | 1.000 | 2578 | tags=50%, list=20%, signal=62% |
| 82 | VIRAL\_REPRODUCTIVE\_PROCESS |  | 33 | 0.35 | 1.37 | 0.095 | 0.196 | 1.000 | 1645 | tags=33%, list=13%, signal=38% |
| 83 | ALCOHOL\_METABOLIC\_PROCESS |  | 82 | 0.28 | 1.37 | 0.063 | 0.204 | 1.000 | 4330 | tags=44%, list=33%, signal=65% |
| 84 | RESPONSE\_TO\_HYPOXIA |  | 27 | 0.35 | 1.32 | 0.131 | 0.261 | 1.000 | 2434 | tags=33%, list=19%, signal=41% |
| 85 | VIRAL\_REPRODUCTION |  | 38 | 0.32 | 1.32 | 0.112 | 0.266 | 1.000 | 1894 | tags=32%, list=14%, signal=37% |
| 86 | VIRAL\_GENOME\_REPLICATION |  | 20 | 0.38 | 1.31 | 0.105 | 0.267 | 1.000 | 1645 | tags=35%, list=13%, signal=40% |
| 87 | ESTABLISHMENT\_AND\_OR\_MAINTENANCE\_OF\_CHROMATIN\_ARCHITECTURE |  | 65 | 0.28 | 1.30 | 0.102 | 0.279 | 1.000 | 2935 | tags=35%, list=22%, signal=45% |
| 88 | INTRACELLULAR\_TRANSPORT |  | 248 | 0.22 | 1.29 | 0.037 | 0.291 | 1.000 | 3668 | tags=35%, list=28%, signal=47% |
| 89 | NEGATIVE\_REGULATION\_OF\_CATALYTIC\_ACTIVITY |  | 61 | 0.28 | 1.29 | 0.108 | 0.295 | 1.000 | 3198 | tags=39%, list=24%, signal=52% |
| 90 | CHROMATIN\_REMODELING |  | 21 | 0.36 | 1.28 | 0.160 | 0.310 | 1.000 | 2935 | tags=43%, list=22%, signal=55% |
| 91 | RESPONSE\_TO\_ORGANIC\_SUBSTANCE |  | 27 | 0.34 | 1.27 | 0.152 | 0.317 | 1.000 | 2949 | tags=37%, list=23%, signal=48% |
| 92 | RNA\_CATABOLIC\_PROCESS |  | 20 | 0.37 | 1.27 | 0.169 | 0.320 | 1.000 | 3158 | tags=50%, list=24%, signal=66% |
| 93 | RESPONSE\_TO\_ABIOTIC\_STIMULUS |  | 79 | 0.26 | 1.26 | 0.107 | 0.329 | 1.000 | 3338 | tags=37%, list=25%, signal=49% |
| 94 | APOPTOTIC\_PROGRAM |  | 56 | 0.28 | 1.26 | 0.125 | 0.329 | 1.000 | 3187 | tags=38%, list=24%, signal=49% |
| 95 | ESTABLISHMENT\_OF\_CELLULAR\_LOCALIZATION |  | 311 | 0.21 | 1.26 | 0.057 | 0.327 | 1.000 | 3770 | tags=34%, list=29%, signal=47% |
| 96 | G1\_PHASE |  | 15 | 0.39 | 1.26 | 0.183 | 0.326 | 1.000 | 3137 | tags=47%, list=24%, signal=61% |
| 97 | NEGATIVE\_REGULATION\_OF\_BINDING |  | 16 | 0.39 | 1.26 | 0.190 | 0.323 | 1.000 | 2339 | tags=44%, list=18%, signal=53% |
| 98 | HETEROCYCLE\_METABOLIC\_PROCESS |  | 26 | 0.34 | 1.26 | 0.162 | 0.323 | 1.000 | 1476 | tags=23%, list=11%, signal=26% |
| 99 | CYTOSKELETON\_DEPENDENT\_INTRACELLULAR\_TRANSPORT |  | 25 | 0.34 | 1.25 | 0.170 | 0.333 | 1.000 | 4169 | tags=52%, list=32%, signal=76% |
| 100 | RESPONSE\_TO\_STRESS |  | 467 | 0.20 | 1.23 | 0.046 | 0.365 | 1.000 | 3053 | tags=29%, list=23%, signal=36% |
| 101 | CELLULAR\_LOCALIZATION |  | 323 | 0.20 | 1.23 | 0.064 | 0.368 | 1.000 | 3937 | tags=35%, list=30%, signal=49% |
| 102 | RIBONUCLEOPROTEIN\_COMPLEX\_BIOGENESIS\_AND\_ASSEMBLY |  | 68 | 0.26 | 1.21 | 0.170 | 0.399 | 1.000 | 3685 | tags=40%, list=28%, signal=55% |
| 103 | RESPONSE\_TO\_HORMONE\_STIMULUS |  | 26 | 0.31 | 1.20 | 0.219 | 0.411 | 1.000 | 4920 | tags=58%, list=38%, signal=92% |
| 104 | DNA\_CATABOLIC\_PROCESS |  | 21 | 0.35 | 1.20 | 0.216 | 0.410 | 1.000 | 3470 | tags=43%, list=27%, signal=58% |
| 105 | TRANSCRIPTION\_FROM\_RNA\_POLYMERASE\_III\_PROMOTER |  | 18 | 0.36 | 1.18 | 0.254 | 0.451 | 1.000 | 3648 | tags=56%, list=28%, signal=77% |
| 106 | REGULATION\_OF\_HYDROLASE\_ACTIVITY |  | 65 | 0.25 | 1.17 | 0.205 | 0.455 | 1.000 | 3187 | tags=34%, list=24%, signal=45% |
| 107 | INDUCTION\_OF\_APOPTOSIS\_BY\_EXTRACELLULAR\_SIGNALS |  | 25 | 0.32 | 1.17 | 0.267 | 0.464 | 1.000 | 2595 | tags=36%, list=20%, signal=45% |
| 108 | CELLULAR\_RESPIRATION |  | 19 | 0.34 | 1.17 | 0.248 | 0.460 | 1.000 | 2677 | tags=37%, list=20%, signal=46% |
| 109 | CATABOLIC\_PROCESS |  | 201 | 0.20 | 1.15 | 0.172 | 0.497 | 1.000 | 3596 | tags=32%, list=27%, signal=44% |
| 110 | REGULATION\_OF\_GENE\_EXPRESSION\_EPIGENETIC |  | 27 | 0.30 | 1.15 | 0.263 | 0.501 | 1.000 | 3249 | tags=41%, list=25%, signal=54% |
| 111 | LIPID\_BIOSYNTHETIC\_PROCESS |  | 84 | 0.24 | 1.15 | 0.249 | 0.501 | 1.000 | 1638 | tags=21%, list=13%, signal=24% |
| 112 | MEIOTIC\_RECOMBINATION |  | 16 | 0.36 | 1.15 | 0.259 | 0.497 | 1.000 | 1797 | tags=31%, list=14%, signal=36% |
| 113 | NITROGEN\_COMPOUND\_BIOSYNTHETIC\_PROCESS |  | 25 | 0.30 | 1.14 | 0.275 | 0.499 | 1.000 | 1549 | tags=24%, list=12%, signal=27% |
| 114 | TRANSCRIPTION\_FROM\_RNA\_POLYMERASE\_II\_PROMOTER |  | 428 | 0.18 | 1.13 | 0.141 | 0.519 | 1.000 | 2666 | tags=24%, list=20%, signal=29% |
| 115 | CELLULAR\_CATABOLIC\_PROCESS |  | 189 | 0.20 | 1.12 | 0.208 | 0.544 | 1.000 | 3596 | tags=32%, list=27%, signal=43% |
| 116 | CHROMATIN\_MODIFICATION |  | 46 | 0.26 | 1.12 | 0.278 | 0.541 | 1.000 | 2935 | tags=33%, list=22%, signal=42% |
| 117 | REGULATION\_OF\_KINASE\_ACTIVITY |  | 135 | 0.21 | 1.12 | 0.248 | 0.541 | 1.000 | 4095 | tags=39%, list=31%, signal=57% |
| 118 | CELL\_STRUCTURE\_DISASSEMBLY\_DURING\_APOPTOSIS |  | 17 | 0.34 | 1.12 | 0.322 | 0.537 | 1.000 | 1200 | tags=24%, list=9%, signal=26% |
| 119 | GLUTAMATE\_SIGNALING\_PATHWAY |  | 17 | 0.33 | 1.12 | 0.298 | 0.537 | 1.000 | 2859 | tags=29%, list=22%, signal=38% |
| 120 | REGULATION\_OF\_CATALYTIC\_ACTIVITY |  | 238 | 0.19 | 1.11 | 0.197 | 0.545 | 1.000 | 3198 | tags=29%, list=24%, signal=37% |
| 121 | REGULATION\_OF\_TRANSFERASE\_ACTIVITY |  | 137 | 0.20 | 1.10 | 0.280 | 0.570 | 1.000 | 1813 | tags=20%, list=14%, signal=23% |
| 122 | NEGATIVE\_REGULATION\_OF\_DNA\_BINDING |  | 15 | 0.35 | 1.10 | 0.340 | 0.566 | 1.000 | 3046 | tags=47%, list=23%, signal=61% |
| 123 | NUCLEAR\_ORGANIZATION\_AND\_BIOGENESIS |  | 23 | 0.31 | 1.10 | 0.325 | 0.565 | 1.000 | 3046 | tags=39%, list=23%, signal=51% |
| 124 | CHROMATIN\_ASSEMBLY |  | 16 | 0.34 | 1.10 | 0.324 | 0.567 | 1.000 | 2935 | tags=44%, list=22%, signal=56% |
| 125 | NEURON\_APOPTOSIS |  | 15 | 0.34 | 1.10 | 0.321 | 0.563 | 1.000 | 1406 | tags=27%, list=11%, signal=30% |
| 126 | CELLULAR\_RESPONSE\_TO\_STIMULUS |  | 17 | 0.33 | 1.09 | 0.331 | 0.561 | 1.000 | 2949 | tags=35%, list=23%, signal=45% |
| 127 | REGULATION\_OF\_PROTEIN\_KINASE\_ACTIVITY |  | 133 | 0.20 | 1.09 | 0.284 | 0.565 | 1.000 | 4095 | tags=39%, list=31%, signal=56% |
| 128 | NEGATIVE\_REGULATION\_OF\_TRANSPORT |  | 18 | 0.32 | 1.09 | 0.336 | 0.569 | 1.000 | 3554 | tags=44%, list=27%, signal=61% |
| 129 | NUCLEAR\_IMPORT |  | 47 | 0.25 | 1.08 | 0.297 | 0.574 | 1.000 | 3640 | tags=36%, list=28%, signal=50% |
| 130 | CYTOSKELETON\_ORGANIZATION\_AND\_BIOGENESIS |  | 182 | 0.19 | 1.08 | 0.301 | 0.594 | 1.000 | 2755 | tags=26%, list=21%, signal=33% |
| 131 | CELLULAR\_BIOSYNTHETIC\_PROCESS |  | 273 | 0.18 | 1.07 | 0.285 | 0.597 | 1.000 | 1572 | tags=18%, list=12%, signal=20% |
| 132 | GAMETE\_GENERATION |  | 92 | 0.21 | 1.06 | 0.325 | 0.624 | 1.000 | 3792 | tags=35%, list=29%, signal=49% |
| 133 | NEGATIVE\_REGULATION\_OF\_APOPTOSIS |  | 136 | 0.20 | 1.06 | 0.345 | 0.632 | 1.000 | 1512 | tags=19%, list=12%, signal=21% |
| 134 | MICROTUBULE\_BASED\_MOVEMENT |  | 16 | 0.33 | 1.05 | 0.394 | 0.642 | 1.000 | 2636 | tags=38%, list=20%, signal=47% |
| 135 | OXYGEN\_AND\_REACTIVE\_OXYGEN\_SPECIES\_METABOLIC\_PROCESS |  | 18 | 0.31 | 1.05 | 0.390 | 0.639 | 1.000 | 2908 | tags=39%, list=22%, signal=50% |
| 136 | REGULATION\_OF\_PROTEIN\_STABILITY |  | 17 | 0.32 | 1.04 | 0.391 | 0.649 | 1.000 | 4066 | tags=41%, list=31%, signal=60% |
| 137 | RNA\_SPLICINGVIA\_TRANSESTERIFICATION\_REACTIONS |  | 27 | 0.28 | 1.04 | 0.377 | 0.649 | 1.000 | 2638 | tags=30%, list=20%, signal=37% |
| 138 | NEGATIVE\_REGULATION\_OF\_PROGRAMMED\_CELL\_DEATH |  | 137 | 0.19 | 1.04 | 0.397 | 0.655 | 1.000 | 1512 | tags=19%, list=12%, signal=21% |
| 139 | REGULATION\_OF\_MOLECULAR\_FUNCTION |  | 275 | 0.17 | 1.04 | 0.343 | 0.651 | 1.000 | 3198 | tags=28%, list=24%, signal=36% |
| 140 | PIGMENT\_BIOSYNTHETIC\_PROCESS |  | 17 | 0.31 | 1.04 | 0.416 | 0.656 | 1.000 | 1476 | tags=24%, list=11%, signal=26% |
| 141 | INTERACTION\_WITH\_HOST |  | 15 | 0.32 | 1.03 | 0.423 | 0.660 | 1.000 | 1428 | tags=27%, list=11%, signal=30% |
| 142 | DNA\_DAMAGE\_RESPONSESIGNAL\_TRANSDUCTION\_RESULTING\_IN\_INDUCTION\_OF\_APOPTOSIS |  | 15 | 0.32 | 1.03 | 0.405 | 0.673 | 1.000 | 1166 | tags=27%, list=9%, signal=29% |
| 143 | MORPHOGENESIS\_OF\_AN\_EPITHELIUM |  | 15 | 0.32 | 1.01 | 0.435 | 0.698 | 1.000 | 3695 | tags=47%, list=28%, signal=65% |
| 144 | CARBOHYDRATE\_TRANSPORT |  | 17 | 0.31 | 1.01 | 0.435 | 0.696 | 1.000 | 2378 | tags=29%, list=18%, signal=36% |
| 145 | NEGATIVE\_REGULATION\_OF\_TRANSFERASE\_ACTIVITY |  | 27 | 0.27 | 1.00 | 0.473 | 0.728 | 1.000 | 3198 | tags=37%, list=24%, signal=49% |
| 146 | STEROID\_HORMONE\_RECEPTOR\_SIGNALING\_PATHWAY |  | 18 | 0.30 | 1.00 | 0.450 | 0.726 | 1.000 | 1168 | tags=22%, list=9%, signal=24% |
| 147 | PROTEIN\_IMPORT |  | 58 | 0.22 | 1.00 | 0.455 | 0.727 | 1.000 | 4126 | tags=36%, list=32%, signal=53% |
| 148 | RESPONSE\_TO\_TEMPERATURE\_STIMULUS |  | 16 | 0.31 | 0.99 | 0.480 | 0.730 | 1.000 | 3338 | tags=44%, list=25%, signal=59% |
| 149 | SECONDARY\_METABOLIC\_PROCESS |  | 23 | 0.27 | 0.99 | 0.471 | 0.726 | 1.000 | 1476 | tags=22%, list=11%, signal=24% |
| 150 | INTRACELLULAR\_RECEPTOR\_MEDIATED\_SIGNALING\_PATHWAY |  | 18 | 0.30 | 0.99 | 0.477 | 0.733 | 1.000 | 1168 | tags=22%, list=9%, signal=24% |
| 151 | STEROID\_METABOLIC\_PROCESS |  | 66 | 0.22 | 0.99 | 0.492 | 0.730 | 1.000 | 4307 | tags=42%, list=33%, signal=63% |
| 152 | CELL\_PROJECTION\_BIOGENESIS |  | 20 | 0.29 | 0.99 | 0.448 | 0.727 | 1.000 | 4153 | tags=45%, list=32%, signal=66% |
| 153 | MACROMOLECULE\_LOCALIZATION |  | 202 | 0.17 | 0.99 | 0.494 | 0.725 | 1.000 | 4176 | tags=35%, list=32%, signal=51% |
| 154 | GENERATION\_OF\_A\_SIGNAL\_INVOLVED\_IN\_CELL\_CELL\_SIGNALING |  | 25 | 0.27 | 0.98 | 0.488 | 0.737 | 1.000 | 2310 | tags=28%, list=18%, signal=34% |
| 155 | REGULATION\_OF\_TRANSCRIPTION\_FROM\_RNA\_POLYMERASE\_II\_PROMOTER |  | 267 | 0.16 | 0.98 | 0.501 | 0.741 | 1.000 | 2373 | tags=22%, list=18%, signal=26% |
| 156 | ENERGY\_DERIVATION\_BY\_OXIDATION\_OF\_ORGANIC\_COMPOUNDS |  | 37 | 0.24 | 0.98 | 0.487 | 0.738 | 1.000 | 2183 | tags=24%, list=17%, signal=29% |
| 157 | PROTEIN\_AMINO\_ACID\_O\_LINKED\_GLYCOSYLATION |  | 18 | 0.28 | 0.98 | 0.492 | 0.737 | 1.000 | 2842 | tags=39%, list=22%, signal=50% |
| 158 | PIGMENT\_METABOLIC\_PROCESS |  | 18 | 0.29 | 0.98 | 0.487 | 0.733 | 1.000 | 1476 | tags=22%, list=11%, signal=25% |
| 159 | INTRACELLULAR\_PROTEIN\_TRANSPORT |  | 127 | 0.18 | 0.97 | 0.537 | 0.747 | 1.000 | 3668 | tags=31%, list=28%, signal=42% |
| 160 | STEROID\_BIOSYNTHETIC\_PROCESS |  | 22 | 0.27 | 0.97 | 0.513 | 0.750 | 1.000 | 4177 | tags=55%, list=32%, signal=80% |
| 161 | PROGRAMMED\_CELL\_DEATH |  | 393 | 0.15 | 0.96 | 0.568 | 0.762 | 1.000 | 1538 | tags=16%, list=12%, signal=17% |
| 162 | APOPTOSIS\_GO |  | 392 | 0.15 | 0.96 | 0.583 | 0.767 | 1.000 | 1538 | tags=16%, list=12%, signal=17% |
| 163 | PROTEIN\_TRANSPORT |  | 139 | 0.18 | 0.95 | 0.574 | 0.768 | 1.000 | 3770 | tags=32%, list=29%, signal=44% |
| 164 | PROTEIN\_TARGETING |  | 94 | 0.19 | 0.95 | 0.560 | 0.784 | 1.000 | 4176 | tags=35%, list=32%, signal=51% |
| 165 | REGULATION\_OF\_PROGRAMMED\_CELL\_DEATH |  | 313 | 0.16 | 0.95 | 0.617 | 0.781 | 1.000 | 1512 | tags=16%, list=12%, signal=18% |
| 166 | DIGESTION |  | 42 | 0.22 | 0.94 | 0.536 | 0.800 | 1.000 | 3028 | tags=24%, list=23%, signal=31% |
| 167 | COVALENT\_CHROMATIN\_MODIFICATION |  | 22 | 0.26 | 0.93 | 0.547 | 0.801 | 1.000 | 3687 | tags=41%, list=28%, signal=57% |
| 168 | REGULATION\_OF\_APOPTOSIS |  | 312 | 0.16 | 0.93 | 0.652 | 0.806 | 1.000 | 1512 | tags=16%, list=12%, signal=18% |
| 169 | BIOSYNTHETIC\_PROCESS |  | 402 | 0.15 | 0.93 | 0.689 | 0.804 | 1.000 | 1674 | tags=16%, list=13%, signal=18% |
| 170 | PROTEIN\_RNA\_COMPLEX\_ASSEMBLY |  | 55 | 0.21 | 0.92 | 0.579 | 0.813 | 1.000 | 3685 | tags=36%, list=28%, signal=50% |
| 171 | INDUCTION\_OF\_APOPTOSIS\_BY\_INTRACELLULAR\_SIGNALS |  | 23 | 0.25 | 0.92 | 0.576 | 0.809 | 1.000 | 3264 | tags=35%, list=25%, signal=46% |
| 172 | NEGATIVE\_REGULATION\_OF\_CELL\_ADHESION |  | 16 | 0.29 | 0.92 | 0.558 | 0.822 | 1.000 | 3366 | tags=44%, list=26%, signal=59% |
| 173 | REGULATION\_OF\_NEUROTRANSMITTER\_LEVELS |  | 23 | 0.25 | 0.91 | 0.556 | 0.828 | 1.000 | 1150 | tags=17%, list=9%, signal=19% |
| 174 | REGULATION\_OF\_TRANSPORT |  | 57 | 0.20 | 0.91 | 0.597 | 0.843 | 1.000 | 4261 | tags=42%, list=33%, signal=62% |
| 175 | PROTEIN\_IMPORT\_INTO\_NUCLEUS |  | 45 | 0.21 | 0.90 | 0.622 | 0.841 | 1.000 | 4126 | tags=38%, list=32%, signal=55% |
| 176 | AROMATIC\_COMPOUND\_METABOLIC\_PROCESS |  | 26 | 0.23 | 0.89 | 0.628 | 0.865 | 1.000 | 414 | tags=15%, list=3%, signal=16% |
| 177 | SEXUAL\_REPRODUCTION |  | 109 | 0.17 | 0.89 | 0.660 | 0.869 | 1.000 | 3792 | tags=31%, list=29%, signal=44% |
| 178 | POSITIVE\_REGULATION\_OF\_CELL\_CYCLE |  | 15 | 0.28 | 0.88 | 0.637 | 0.885 | 1.000 | 1550 | tags=27%, list=12%, signal=30% |
| 179 | REGULATION\_OF\_RNA\_METABOLIC\_PROCESS |  | 417 | 0.14 | 0.88 | 0.832 | 0.887 | 1.000 | 2374 | tags=20%, list=18%, signal=24% |
| 180 | REGULATION\_OF\_NUCLEOCYTOPLASMIC\_TRANSPORT |  | 19 | 0.25 | 0.88 | 0.653 | 0.888 | 1.000 | 1263 | tags=21%, list=10%, signal=23% |
| 181 | ENERGY\_RESERVE\_METABOLIC\_PROCESS |  | 15 | 0.28 | 0.87 | 0.631 | 0.891 | 1.000 | 1249 | tags=20%, list=10%, signal=22% |
| 182 | REGULATION\_OF\_TRANSCRIPTIONDNA\_DEPENDENT |  | 412 | 0.14 | 0.87 | 0.863 | 0.893 | 1.000 | 2374 | tags=20%, list=18%, signal=24% |
| 183 | TRANSMISSION\_OF\_NERVE\_IMPULSE |  | 167 | 0.16 | 0.87 | 0.750 | 0.889 | 1.000 | 2525 | tags=20%, list=19%, signal=25% |
| 184 | EMBRYONIC\_DEVELOPMENT |  | 46 | 0.20 | 0.87 | 0.668 | 0.885 | 1.000 | 2998 | tags=26%, list=23%, signal=34% |
| 185 | SPLICEOSOME\_ASSEMBLY |  | 17 | 0.26 | 0.86 | 0.646 | 0.909 | 1.000 | 3299 | tags=35%, list=25%, signal=47% |
| 186 | PHOSPHOINOSITIDE\_BIOSYNTHETIC\_PROCESS |  | 21 | 0.25 | 0.85 | 0.668 | 0.908 | 1.000 | 1004 | tags=19%, list=8%, signal=21% |
| 187 | RESPONSE\_TO\_RADIATION |  | 52 | 0.19 | 0.85 | 0.709 | 0.908 | 1.000 | 3216 | tags=31%, list=25%, signal=41% |
| 188 | SYNAPTIC\_TRANSMISSION |  | 154 | 0.15 | 0.85 | 0.800 | 0.911 | 1.000 | 2489 | tags=19%, list=19%, signal=24% |
| 189 | PROTEOLYSIS |  | 170 | 0.15 | 0.85 | 0.803 | 0.909 | 1.000 | 3850 | tags=32%, list=29%, signal=45% |
| 190 | REGULATION\_OF\_INTRACELLULAR\_TRANSPORT |  | 22 | 0.24 | 0.84 | 0.677 | 0.918 | 1.000 | 4261 | tags=45%, list=33%, signal=67% |
| 191 | CALCIUM\_INDEPENDENT\_CELL\_CELL\_ADHESION |  | 16 | 0.26 | 0.83 | 0.671 | 0.928 | 1.000 | 3552 | tags=38%, list=27%, signal=51% |
| 192 | MEMBRANE\_LIPID\_BIOSYNTHETIC\_PROCESS |  | 41 | 0.19 | 0.83 | 0.752 | 0.930 | 1.000 | 1549 | tags=17%, list=12%, signal=19% |
| 193 | PROTEIN\_HOMOOLIGOMERIZATION |  | 19 | 0.24 | 0.83 | 0.709 | 0.931 | 1.000 | 794 | tags=16%, list=6%, signal=17% |
| 194 | DEVELOPMENT\_OF\_PRIMARY\_SEXUAL\_CHARACTERISTICS |  | 25 | 0.22 | 0.82 | 0.730 | 0.932 | 1.000 | 2998 | tags=28%, list=23%, signal=36% |
| 195 | MEMBRANE\_FUSION |  | 27 | 0.22 | 0.82 | 0.731 | 0.929 | 1.000 | 3543 | tags=37%, list=27%, signal=51% |
| 196 | NITROGEN\_COMPOUND\_METABOLIC\_PROCESS |  | 141 | 0.15 | 0.82 | 0.846 | 0.928 | 1.000 | 1764 | tags=16%, list=13%, signal=19% |
| 197 | CASPASE\_ACTIVATION |  | 24 | 0.22 | 0.81 | 0.725 | 0.948 | 1.000 | 3046 | tags=33%, list=23%, signal=43% |
| 198 | ESTABLISHMENT\_OF\_PROTEIN\_LOCALIZATION |  | 166 | 0.15 | 0.81 | 0.901 | 0.946 | 1.000 | 4176 | tags=33%, list=32%, signal=48% |
| 199 | ECTODERM\_DEVELOPMENT |  | 75 | 0.17 | 0.80 | 0.840 | 0.946 | 1.000 | 2464 | tags=24%, list=19%, signal=29% |
| 200 | HISTONE\_MODIFICATION |  | 21 | 0.22 | 0.79 | 0.738 | 0.967 | 1.000 | 3687 | tags=38%, list=28%, signal=53% |
| 201 | REGULATION\_OF\_SECRETION |  | 35 | 0.19 | 0.79 | 0.796 | 0.965 | 1.000 | 2362 | tags=26%, list=18%, signal=31% |
| 202 | TRANSLATIONAL\_INITIATION |  | 33 | 0.20 | 0.79 | 0.807 | 0.960 | 1.000 | 3595 | tags=36%, list=27%, signal=50% |
| 203 | PHOSPHOLIPID\_BIOSYNTHETIC\_PROCESS |  | 35 | 0.19 | 0.78 | 0.815 | 0.963 | 1.000 | 1549 | tags=17%, list=12%, signal=19% |
| 204 | EXOCYTOSIS |  | 22 | 0.22 | 0.78 | 0.776 | 0.959 | 1.000 | 10232 | tags=100%, list=78%, signal=457% |
| 205 | LIPID\_TRANSPORT |  | 27 | 0.21 | 0.78 | 0.807 | 0.959 | 1.000 | 1924 | tags=22%, list=15%, signal=26% |
| 206 | RESPONSE\_TO\_UV |  | 22 | 0.22 | 0.77 | 0.780 | 0.961 | 1.000 | 3216 | tags=36%, list=25%, signal=48% |
| 207 | REPRODUCTION |  | 215 | 0.13 | 0.77 | 0.973 | 0.958 | 1.000 | 3815 | tags=30%, list=29%, signal=41% |
| 208 | NEGATIVE\_REGULATION\_OF\_CELLULAR\_BIOSYNTHETIC\_PROCESS |  | 25 | 0.21 | 0.77 | 0.806 | 0.959 | 1.000 | 3188 | tags=28%, list=24%, signal=37% |
| 209 | GLYCEROPHOSPHOLIPID\_BIOSYNTHETIC\_PROCESS |  | 27 | 0.20 | 0.77 | 0.800 | 0.957 | 1.000 | 1004 | tags=15%, list=8%, signal=16% |
| 210 | NEGATIVE\_REGULATION\_OF\_CELL\_CYCLE |  | 72 | 0.16 | 0.76 | 0.887 | 0.960 | 1.000 | 2076 | tags=19%, list=16%, signal=23% |
| 211 | SENSORY\_PERCEPTION |  | 163 | 0.14 | 0.76 | 0.950 | 0.963 | 1.000 | 5064 | tags=42%, list=39%, signal=67% |
| 212 | POSITIVE\_REGULATION\_OF\_HYDROLASE\_ACTIVITY |  | 45 | 0.17 | 0.75 | 0.890 | 0.965 | 1.000 | 3046 | tags=27%, list=23%, signal=35% |
| 213 | CELLULAR\_CARBOHYDRATE\_METABOLIC\_PROCESS |  | 106 | 0.14 | 0.75 | 0.930 | 0.965 | 1.000 | 2163 | tags=17%, list=17%, signal=20% |
| 214 | NEGATIVE\_REGULATION\_OF\_BIOSYNTHETIC\_PROCESS |  | 26 | 0.20 | 0.73 | 0.835 | 0.977 | 1.000 | 3188 | tags=27%, list=24%, signal=36% |
| 215 | HOMEOSTASIS\_OF\_NUMBER\_OF\_CELLS |  | 20 | 0.21 | 0.73 | 0.841 | 0.977 | 1.000 | 2330 | tags=25%, list=18%, signal=30% |
| 216 | REGULATION\_OF\_CELL\_ADHESION |  | 31 | 0.19 | 0.73 | 0.868 | 0.973 | 1.000 | 4184 | tags=42%, list=32%, signal=61% |
| 217 | LIPOPROTEIN\_METABOLIC\_PROCESS |  | 30 | 0.19 | 0.72 | 0.871 | 0.974 | 1.000 | 1924 | tags=20%, list=15%, signal=23% |
| 218 | EPIDERMAL\_GROWTH\_FACTOR\_RECEPTOR\_SIGNALING\_PATHWAY |  | 18 | 0.21 | 0.72 | 0.836 | 0.975 | 1.000 | 4075 | tags=39%, list=31%, signal=56% |
| 219 | JNK\_CASCADE |  | 44 | 0.17 | 0.71 | 0.912 | 0.978 | 1.000 | 4082 | tags=39%, list=31%, signal=56% |
| 220 | STRESS\_ACTIVATED\_PROTEIN\_KINASE\_SIGNALING\_PATHWAY |  | 45 | 0.16 | 0.71 | 0.929 | 0.977 | 1.000 | 4082 | tags=38%, list=31%, signal=55% |
| 221 | NEUROLOGICAL\_SYSTEM\_PROCESS |  | 328 | 0.12 | 0.71 | 1.000 | 0.973 | 1.000 | 5117 | tags=40%, list=39%, signal=64% |
| 222 | REGULATION\_OF\_CATABOLIC\_PROCESS |  | 15 | 0.22 | 0.70 | 0.856 | 0.975 | 1.000 | 4278 | tags=47%, list=33%, signal=69% |
| 223 | GLUCOSE\_METABOLIC\_PROCESS |  | 27 | 0.18 | 0.69 | 0.895 | 0.975 | 1.000 | 4330 | tags=37%, list=33%, signal=55% |
| 224 | RHO\_PROTEIN\_SIGNAL\_TRANSDUCTION |  | 30 | 0.18 | 0.68 | 0.906 | 0.984 | 1.000 | 2706 | tags=23%, list=21%, signal=29% |
| 225 | ANION\_TRANSPORT |  | 27 | 0.18 | 0.66 | 0.931 | 0.988 | 1.000 | 1476 | tags=15%, list=11%, signal=17% |
| 226 | BIOGENIC\_AMINE\_METABOLIC\_PROCESS |  | 16 | 0.20 | 0.66 | 0.914 | 0.988 | 1.000 | 1549 | tags=19%, list=12%, signal=21% |
| 227 | PROTEIN\_AMINO\_ACID\_LIPIDATION |  | 21 | 0.19 | 0.65 | 0.929 | 0.986 | 1.000 | 1004 | tags=14%, list=8%, signal=15% |
| 228 | TUBE\_DEVELOPMENT |  | 15 | 0.20 | 0.63 | 0.931 | 0.991 | 1.000 | 3695 | tags=33%, list=28%, signal=46% |
| 229 | LIPOPROTEIN\_BIOSYNTHETIC\_PROCESS |  | 23 | 0.18 | 0.63 | 0.945 | 0.990 | 1.000 | 3880 | tags=35%, list=30%, signal=49% |
| 230 | NEGATIVE\_REGULATION\_OF\_TRANSLATION |  | 19 | 0.19 | 0.62 | 0.947 | 0.986 | 1.000 | 3188 | tags=26%, list=24%, signal=35% |
| 231 | ADENYLATE\_CYCLASE\_ACTIVATION |  | 18 | 0.18 | 0.61 | 0.954 | 0.986 | 1.000 | 4563 | tags=39%, list=35%, signal=60% |
| 232 | FEEDING\_BEHAVIOR |  | 20 | 0.18 | 0.61 | 0.956 | 0.983 | 1.000 | 5009 | tags=45%, list=38%, signal=73% |
| 233 | REGULATION\_OF\_HEART\_CONTRACTION |  | 24 | 0.15 | 0.53 | 0.988 | 0.997 | 1.000 | 3932 | tags=33%, list=30%, signal=48% |
| 234 | PEROXISOME\_ORGANIZATION\_AND\_BIOGENESIS |  | 15 | 0.14 | 0.43 | 1.000 | 0.999 | 1.000 | 11275 | tags=100%, list=86%, signal=720% |
Table: Gene sets enriched in phenotype **na**[plain text format]****

  
