# Supplementary material for: Multiple-input multiple-output causal strategies for gene selection
Source: BMC Bioinformatics. 2011 Nov 25;12:458. doi: 10.1186/1471-2105-12-458 (PMC3323860; doi:10.1186/1471-2105-12-458)
Supplement: Additional file 3 — Archive containing the output files computed by the preranked GSEA for λ ∈ {0.6,0.7,0.8,0.9,1.0,2.0} (GSEA_MIMO_part2.zip). [file 1471-2105-12-458-S3.ZIP › mFS10_entrez_mimo.GseaPreranked.1316039488125/gsea_report_for_na_neg_1316039488125.html]

Report for na\_neg 1316039488125 [GSEA]

| GS  follow link to MSigDB | GS DETAILS | SIZE | ES | NES | NOM p-val | FDR q-val | FWER p-val | RANK AT MAX | LEADING EDGE || 1 | IMMUNE\_RESPONSE |  | 212 | -0.41 | -2.42 | 0.000 | 0.003 | 0.002 | 3492 | tags=48%, list=27%, signal=65% |
| 2 | DEFENSE\_RESPONSE |  | 238 | -0.39 | -2.41 | 0.000 | 0.001 | 0.002 | 3474 | tags=44%, list=27%, signal=58% |
| 3 | IMMUNE\_SYSTEM\_PROCESS |  | 298 | -0.37 | -2.31 | 0.000 | 0.002 | 0.005 | 2772 | tags=39%, list=21%, signal=48% |
| 4 | POSITIVE\_REGULATION\_OF\_IMMUNE\_RESPONSE |  | 24 | -0.58 | -2.19 | 0.000 | 0.006 | 0.018 | 2819 | tags=58%, list=22%, signal=74% |
| 5 | INFLAMMATORY\_RESPONSE |  | 115 | -0.40 | -2.18 | 0.000 | 0.005 | 0.019 | 3087 | tags=43%, list=24%, signal=55% |
| 6 | REGULATION\_OF\_IMMUNE\_RESPONSE |  | 28 | -0.52 | -2.10 | 0.000 | 0.011 | 0.049 | 3223 | tags=57%, list=25%, signal=76% |
| 7 | POSITIVE\_REGULATION\_OF\_MULTICELLULAR\_ORGANISMAL\_PROCESS |  | 56 | -0.45 | -2.09 | 0.000 | 0.010 | 0.052 | 3227 | tags=50%, list=25%, signal=66% |
| 8 | CELLULAR\_DEFENSE\_RESPONSE |  | 54 | -0.46 | -2.09 | 0.000 | 0.009 | 0.056 | 3412 | tags=48%, list=26%, signal=65% |
| 9 | RESPONSE\_TO\_WOUNDING |  | 171 | -0.36 | -2.08 | 0.000 | 0.009 | 0.063 | 3263 | tags=41%, list=25%, signal=54% |
| 10 | POSITIVE\_REGULATION\_OF\_IMMUNE\_SYSTEM\_PROCESS |  | 44 | -0.47 | -2.07 | 0.000 | 0.009 | 0.067 | 3227 | tags=50%, list=25%, signal=66% |
| 11 | REGULATION\_OF\_IMMUNE\_SYSTEM\_PROCESS |  | 57 | -0.44 | -2.06 | 0.000 | 0.009 | 0.073 | 3227 | tags=51%, list=25%, signal=67% |
| 12 | ADAPTIVE\_IMMUNE\_RESPONSE\_GO\_0002460 |  | 22 | -0.51 | -1.85 | 0.014 | 0.064 | 0.453 | 3037 | tags=50%, list=23%, signal=65% |
| 13 | HEMOPOIETIC\_OR\_LYMPHOID\_ORGAN\_DEVELOPMENT |  | 71 | -0.37 | -1.81 | 0.000 | 0.077 | 0.539 | 2764 | tags=39%, list=21%, signal=50% |
| 14 | ADAPTIVE\_IMMUNE\_RESPONSE |  | 23 | -0.48 | -1.81 | 0.004 | 0.074 | 0.555 | 3037 | tags=48%, list=23%, signal=62% |
| 15 | HEMOPOIESIS |  | 69 | -0.37 | -1.80 | 0.002 | 0.075 | 0.591 | 2764 | tags=39%, list=21%, signal=49% |
| 16 | REGULATION\_OF\_MULTICELLULAR\_ORGANISMAL\_PROCESS |  | 131 | -0.32 | -1.77 | 0.000 | 0.085 | 0.663 | 3227 | tags=40%, list=25%, signal=52% |
| 17 | RECEPTOR\_MEDIATED\_ENDOCYTOSIS |  | 31 | -0.45 | -1.77 | 0.007 | 0.081 | 0.663 | 1856 | tags=35%, list=14%, signal=41% |
| 18 | IMMUNE\_EFFECTOR\_PROCESS |  | 34 | -0.43 | -1.76 | 0.007 | 0.081 | 0.693 | 3223 | tags=56%, list=25%, signal=74% |
| 19 | IMMUNE\_SYSTEM\_DEVELOPMENT |  | 75 | -0.35 | -1.75 | 0.000 | 0.085 | 0.721 | 2764 | tags=39%, list=21%, signal=49% |
| 20 | RESPONSE\_TO\_EXTERNAL\_STIMULUS |  | 278 | -0.28 | -1.74 | 0.000 | 0.088 | 0.748 | 2230 | tags=28%, list=17%, signal=33% |
| 21 | LYMPHOCYTE\_ACTIVATION |  | 54 | -0.37 | -1.73 | 0.000 | 0.094 | 0.793 | 3227 | tags=46%, list=25%, signal=61% |
| 22 | POSITIVE\_REGULATION\_OF\_RESPONSE\_TO\_STIMULUS |  | 35 | -0.40 | -1.68 | 0.017 | 0.135 | 0.899 | 2819 | tags=46%, list=22%, signal=58% |
| 23 | REGULATION\_OF\_DEFENSE\_RESPONSE |  | 15 | -0.52 | -1.67 | 0.025 | 0.134 | 0.908 | 3471 | tags=60%, list=27%, signal=82% |
| 24 | JAK\_STAT\_CASCADE |  | 26 | -0.43 | -1.67 | 0.019 | 0.134 | 0.918 | 1752 | tags=35%, list=13%, signal=40% |
| 25 | CELL\_ACTIVATION |  | 64 | -0.34 | -1.67 | 0.002 | 0.129 | 0.918 | 3512 | tags=47%, list=27%, signal=64% |
| 26 | T\_CELL\_ACTIVATION |  | 39 | -0.38 | -1.65 | 0.013 | 0.137 | 0.931 | 3227 | tags=44%, list=25%, signal=58% |
| 27 | LEUKOCYTE\_DIFFERENTIATION |  | 34 | -0.40 | -1.64 | 0.004 | 0.138 | 0.939 | 3107 | tags=47%, list=24%, signal=62% |
| 28 | LIPID\_CATABOLIC\_PROCESS |  | 34 | -0.39 | -1.64 | 0.013 | 0.142 | 0.947 | 3952 | tags=56%, list=30%, signal=80% |
| 29 | LEUKOCYTE\_ACTIVATION |  | 59 | -0.34 | -1.62 | 0.007 | 0.154 | 0.965 | 3227 | tags=44%, list=25%, signal=58% |
| 30 | HUMORAL\_IMMUNE\_RESPONSE |  | 30 | -0.41 | -1.62 | 0.020 | 0.149 | 0.965 | 4340 | tags=67%, list=33%, signal=100% |
| 31 | MULTI\_ORGANISM\_PROCESS |  | 137 | -0.28 | -1.57 | 0.000 | 0.197 | 0.989 | 3743 | tags=44%, list=29%, signal=61% |
| 32 | LYMPHOCYTE\_DIFFERENTIATION |  | 23 | -0.43 | -1.56 | 0.025 | 0.205 | 0.992 | 3107 | tags=52%, list=24%, signal=68% |
| 33 | B\_CELL\_ACTIVATION |  | 17 | -0.46 | -1.56 | 0.043 | 0.211 | 0.997 | 3037 | tags=59%, list=23%, signal=76% |
| 34 | REGULATION\_OF\_CELL\_DIFFERENTIATION |  | 48 | -0.35 | -1.55 | 0.011 | 0.220 | 0.998 | 3107 | tags=38%, list=24%, signal=49% |
| 35 | ENZYME\_LINKED\_RECEPTOR\_PROTEIN\_SIGNALING\_PATHWAY |  | 128 | -0.27 | -1.52 | 0.005 | 0.250 | 0.999 | 1477 | tags=22%, list=11%, signal=24% |
| 36 | NEGATIVE\_REGULATION\_OF\_SIGNAL\_TRANSDUCTION |  | 31 | -0.38 | -1.52 | 0.041 | 0.248 | 0.999 | 2734 | tags=35%, list=21%, signal=45% |
| 37 | CELL\_SUBSTRATE\_ADHESION |  | 36 | -0.36 | -1.52 | 0.032 | 0.244 | 0.999 | 2424 | tags=36%, list=19%, signal=44% |
| 38 | ACTIN\_CYTOSKELETON\_ORGANIZATION\_AND\_BIOGENESIS |  | 90 | -0.29 | -1.50 | 0.017 | 0.261 | 0.999 | 2901 | tags=32%, list=22%, signal=41% |
| 39 | REGULATION\_OF\_RESPONSE\_TO\_STIMULUS |  | 49 | -0.33 | -1.50 | 0.020 | 0.259 | 0.999 | 3223 | tags=45%, list=25%, signal=59% |
| 40 | TRANSFORMING\_GROWTH\_FACTOR\_BETA\_RECEPTOR\_SIGNALING\_PATHWAY |  | 34 | -0.36 | -1.50 | 0.038 | 0.260 | 0.999 | 1794 | tags=32%, list=14%, signal=37% |
| 41 | PEPTIDYL\_TYROSINE\_MODIFICATION |  | 23 | -0.41 | -1.48 | 0.045 | 0.273 | 1.000 | 1400 | tags=26%, list=11%, signal=29% |
| 42 | POSITIVE\_REGULATION\_OF\_SIGNAL\_TRANSDUCTION |  | 97 | -0.28 | -1.47 | 0.009 | 0.289 | 1.000 | 2819 | tags=34%, list=22%, signal=43% |
| 43 | MAINTENANCE\_OF\_LOCALIZATION |  | 21 | -0.41 | -1.47 | 0.050 | 0.288 | 1.000 | 2669 | tags=38%, list=20%, signal=48% |
| 44 | DETECTION\_OF\_STIMULUS |  | 36 | -0.35 | -1.46 | 0.042 | 0.288 | 1.000 | 4707 | tags=56%, list=36%, signal=87% |
| 45 | CATION\_HOMEOSTASIS |  | 94 | -0.28 | -1.46 | 0.019 | 0.283 | 1.000 | 3282 | tags=37%, list=25%, signal=49% |
| 46 | REGULATION\_OF\_LYMPHOCYTE\_ACTIVATION |  | 31 | -0.37 | -1.46 | 0.056 | 0.280 | 1.000 | 3227 | tags=45%, list=25%, signal=60% |
| 47 | REGULATION\_OF\_PROTEIN\_AMINO\_ACID\_PHOSPHORYLATION |  | 23 | -0.40 | -1.45 | 0.065 | 0.287 | 1.000 | 2424 | tags=35%, list=19%, signal=43% |
| 48 | CELLULAR\_LIPID\_CATABOLIC\_PROCESS |  | 31 | -0.36 | -1.45 | 0.057 | 0.282 | 1.000 | 3952 | tags=52%, list=30%, signal=74% |
| 49 | PROTEIN\_AMINO\_ACID\_N\_LINKED\_GLYCOSYLATION |  | 27 | -0.37 | -1.45 | 0.049 | 0.289 | 1.000 | 2560 | tags=37%, list=20%, signal=46% |
| 50 | REGULATION\_OF\_SIGNAL\_TRANSDUCTION |  | 173 | -0.25 | -1.44 | 0.019 | 0.296 | 1.000 | 3241 | tags=34%, list=25%, signal=44% |
| 51 | CELLULAR\_CATION\_HOMEOSTASIS |  | 91 | -0.28 | -1.44 | 0.035 | 0.294 | 1.000 | 3282 | tags=37%, list=25%, signal=50% |
| 52 | PROTEIN\_KINASE\_CASCADE |  | 239 | -0.24 | -1.43 | 0.008 | 0.295 | 1.000 | 2517 | tags=28%, list=19%, signal=34% |
| 53 | CYTOKINE\_AND\_CHEMOKINE\_MEDIATED\_SIGNALING\_PATHWAY |  | 19 | -0.41 | -1.43 | 0.065 | 0.295 | 1.000 | 1552 | tags=32%, list=12%, signal=36% |
| 54 | TRANSMEMBRANE\_RECEPTOR\_PROTEIN\_SERINE\_THREONINE\_KINASE\_SIGNALING\_PATHWAY |  | 42 | -0.33 | -1.42 | 0.060 | 0.311 | 1.000 | 1794 | tags=29%, list=14%, signal=33% |
| 55 | REGULATION\_OF\_T\_CELL\_ACTIVATION |  | 25 | -0.37 | -1.42 | 0.048 | 0.309 | 1.000 | 3227 | tags=44%, list=25%, signal=58% |
| 56 | PROTEIN\_COMPLEX\_ASSEMBLY |  | 157 | -0.25 | -1.41 | 0.015 | 0.314 | 1.000 | 3030 | tags=32%, list=23%, signal=41% |
| 57 | POSITIVE\_REGULATION\_OF\_LYMPHOCYTE\_ACTIVATION |  | 23 | -0.38 | -1.41 | 0.067 | 0.314 | 1.000 | 3227 | tags=43%, list=25%, signal=58% |
| 58 | POSITIVE\_REGULATION\_OF\_CELL\_DIFFERENTIATION |  | 21 | -0.39 | -1.41 | 0.090 | 0.309 | 1.000 | 3107 | tags=48%, list=24%, signal=62% |
| 59 | FEMALE\_PREGNANCY |  | 42 | -0.32 | -1.41 | 0.066 | 0.307 | 1.000 | 3743 | tags=50%, list=29%, signal=70% |
| 60 | RESPONSE\_TO\_OTHER\_ORGANISM |  | 69 | -0.29 | -1.41 | 0.053 | 0.302 | 1.000 | 2149 | tags=30%, list=16%, signal=36% |
| 61 | POSITIVE\_REGULATION\_OF\_PHOSPHATE\_METABOLIC\_PROCESS |  | 23 | -0.38 | -1.40 | 0.065 | 0.302 | 1.000 | 1656 | tags=30%, list=13%, signal=35% |
| 62 | REGULATION\_OF\_ANGIOGENESIS |  | 24 | -0.37 | -1.40 | 0.086 | 0.304 | 1.000 | 2133 | tags=42%, list=16%, signal=50% |
| 63 | SMALL\_GTPASE\_MEDIATED\_SIGNAL\_TRANSDUCTION |  | 77 | -0.28 | -1.40 | 0.026 | 0.301 | 1.000 | 3224 | tags=38%, list=25%, signal=50% |
| 64 | INNATE\_IMMUNE\_RESPONSE |  | 19 | -0.39 | -1.40 | 0.090 | 0.296 | 1.000 | 5029 | tags=74%, list=38%, signal=119% |
| 65 | CELL\_MATRIX\_ADHESION |  | 35 | -0.34 | -1.40 | 0.068 | 0.294 | 1.000 | 2424 | tags=34%, list=19%, signal=42% |
| 66 | MESODERM\_DEVELOPMENT |  | 22 | -0.38 | -1.40 | 0.092 | 0.294 | 1.000 | 4673 | tags=59%, list=36%, signal=92% |
| 67 | TRANSMEMBRANE\_RECEPTOR\_PROTEIN\_TYROSINE\_KINASE\_SIGNALING\_PATHWAY |  | 76 | -0.28 | -1.38 | 0.046 | 0.309 | 1.000 | 1662 | tags=22%, list=13%, signal=25% |
| 68 | GROWTH |  | 59 | -0.29 | -1.38 | 0.053 | 0.305 | 1.000 | 4176 | tags=44%, list=32%, signal=64% |
| 69 | REGULATION\_OF\_CYTOSKELETON\_ORGANIZATION\_AND\_BIOGENESIS |  | 26 | -0.36 | -1.38 | 0.082 | 0.301 | 1.000 | 2728 | tags=35%, list=21%, signal=44% |
| 70 | WOUND\_HEALING |  | 49 | -0.30 | -1.37 | 0.067 | 0.327 | 1.000 | 3512 | tags=39%, list=27%, signal=53% |
| 71 | FATTY\_ACID\_METABOLIC\_PROCESS |  | 56 | -0.29 | -1.36 | 0.063 | 0.327 | 1.000 | 3952 | tags=48%, list=30%, signal=69% |
| 72 | PEPTIDYL\_TYROSINE\_PHOSPHORYLATION |  | 21 | -0.37 | -1.36 | 0.106 | 0.339 | 1.000 | 1400 | tags=24%, list=11%, signal=27% |
| 73 | CYTOKINE\_PRODUCTION |  | 61 | -0.29 | -1.35 | 0.051 | 0.351 | 1.000 | 2793 | tags=34%, list=21%, signal=44% |
| 74 | POSITIVE\_REGULATION\_OF\_CYTOKINE\_BIOSYNTHETIC\_PROCESS |  | 21 | -0.37 | -1.35 | 0.116 | 0.350 | 1.000 | 2772 | tags=43%, list=21%, signal=54% |
| 75 | COAGULATION |  | 41 | -0.31 | -1.34 | 0.080 | 0.352 | 1.000 | 3512 | tags=39%, list=27%, signal=53% |
| 76 | BLOOD\_COAGULATION |  | 41 | -0.31 | -1.34 | 0.111 | 0.350 | 1.000 | 3512 | tags=39%, list=27%, signal=53% |
| 77 | PROTEIN\_AMINO\_ACID\_PHOSPHORYLATION |  | 231 | -0.22 | -1.33 | 0.015 | 0.358 | 1.000 | 3939 | tags=36%, list=30%, signal=51% |
| 78 | REGULATION\_OF\_ANATOMICAL\_STRUCTURE\_MORPHOGENESIS |  | 17 | -0.39 | -1.33 | 0.120 | 0.361 | 1.000 | 3220 | tags=35%, list=25%, signal=47% |
| 79 | RESPONSE\_TO\_VIRUS |  | 45 | -0.30 | -1.32 | 0.101 | 0.374 | 1.000 | 2149 | tags=33%, list=16%, signal=40% |
| 80 | REGULATION\_OF\_CELL\_PROLIFERATION |  | 275 | -0.21 | -1.31 | 0.013 | 0.387 | 1.000 | 2534 | tags=25%, list=19%, signal=31% |
| 81 | GLYCOPROTEIN\_METABOLIC\_PROCESS |  | 82 | -0.26 | -1.31 | 0.060 | 0.388 | 1.000 | 3571 | tags=37%, list=27%, signal=50% |
| 82 | MONOCARBOXYLIC\_ACID\_METABOLIC\_PROCESS |  | 77 | -0.26 | -1.31 | 0.059 | 0.385 | 1.000 | 3972 | tags=44%, list=30%, signal=63% |
| 83 | RAS\_PROTEIN\_SIGNAL\_TRANSDUCTION |  | 55 | -0.28 | -1.30 | 0.100 | 0.391 | 1.000 | 3163 | tags=38%, list=24%, signal=50% |
| 84 | POSITIVE\_REGULATION\_OF\_CELL\_PROLIFERATION |  | 129 | -0.23 | -1.30 | 0.049 | 0.401 | 1.000 | 1689 | tags=22%, list=13%, signal=25% |
| 85 | ACTIN\_FILAMENT\_BASED\_PROCESS |  | 99 | -0.25 | -1.29 | 0.078 | 0.408 | 1.000 | 2901 | tags=29%, list=22%, signal=37% |
| 86 | ACTIN\_POLYMERIZATION\_AND\_OR\_DEPOLYMERIZATION |  | 20 | -0.36 | -1.29 | 0.154 | 0.406 | 1.000 | 2728 | tags=30%, list=21%, signal=38% |
| 87 | MUSCLE\_DEVELOPMENT |  | 85 | -0.25 | -1.29 | 0.073 | 0.410 | 1.000 | 3430 | tags=40%, list=26%, signal=54% |
| 88 | POSITIVE\_REGULATION\_OF\_PROTEIN\_AMINO\_ACID\_PHOSPHORYLATION |  | 15 | -0.39 | -1.28 | 0.142 | 0.412 | 1.000 | 1656 | tags=33%, list=13%, signal=38% |
| 89 | RESPONSE\_TO\_BACTERIUM |  | 22 | -0.34 | -1.27 | 0.159 | 0.432 | 1.000 | 3840 | tags=45%, list=29%, signal=64% |
| 90 | PHOSPHOLIPID\_METABOLIC\_PROCESS |  | 63 | -0.26 | -1.26 | 0.103 | 0.447 | 1.000 | 2872 | tags=33%, list=22%, signal=42% |
| 91 | DEFENSE\_RESPONSE\_TO\_BACTERIUM |  | 16 | -0.38 | -1.26 | 0.162 | 0.446 | 1.000 | 3529 | tags=44%, list=27%, signal=60% |
| 92 | REGULATION\_OF\_ORGANELLE\_ORGANIZATION\_AND\_BIOGENESIS |  | 35 | -0.30 | -1.26 | 0.167 | 0.456 | 1.000 | 2921 | tags=34%, list=22%, signal=44% |
| 93 | POSITIVE\_REGULATION\_OF\_CELLULAR\_PROTEIN\_METABOLIC\_PROCESS |  | 61 | -0.26 | -1.26 | 0.123 | 0.453 | 1.000 | 2475 | tags=31%, list=19%, signal=38% |
| 94 | POSITIVE\_REGULATION\_OF\_TRANSLATION |  | 28 | -0.31 | -1.26 | 0.157 | 0.449 | 1.000 | 2772 | tags=39%, list=21%, signal=50% |
| 95 | ACTIVATION\_OF\_NF\_KAPPAB\_TRANSCRIPTION\_FACTOR |  | 15 | -0.39 | -1.25 | 0.168 | 0.455 | 1.000 | 4014 | tags=60%, list=31%, signal=86% |
| 96 | AMINO\_ACID\_TRANSPORT |  | 25 | -0.33 | -1.25 | 0.172 | 0.459 | 1.000 | 515 | tags=20%, list=4%, signal=21% |
| 97 | REGULATION\_OF\_PROTEIN\_IMPORT\_INTO\_NUCLEUS |  | 15 | -0.38 | -1.25 | 0.176 | 0.455 | 1.000 | 1301 | tags=27%, list=10%, signal=30% |
| 98 | REGULATION\_OF\_BODY\_FLUID\_LEVELS |  | 55 | -0.27 | -1.24 | 0.133 | 0.465 | 1.000 | 3625 | tags=38%, list=28%, signal=53% |
| 99 | POSITIVE\_REGULATION\_OF\_PROTEIN\_METABOLIC\_PROCESS |  | 63 | -0.26 | -1.24 | 0.143 | 0.461 | 1.000 | 2921 | tags=35%, list=22%, signal=45% |
| 100 | BEHAVIOR |  | 136 | -0.22 | -1.24 | 0.118 | 0.457 | 1.000 | 3988 | tags=38%, list=30%, signal=53% |
| 101 | REGULATION\_OF\_BLOOD\_PRESSURE |  | 22 | -0.33 | -1.24 | 0.180 | 0.463 | 1.000 | 3158 | tags=36%, list=24%, signal=48% |
| 102 | REGULATION\_OF\_I\_KAPPAB\_KINASE\_NF\_KAPPAB\_CASCADE |  | 72 | -0.25 | -1.23 | 0.127 | 0.464 | 1.000 | 2812 | tags=33%, list=21%, signal=42% |
| 103 | HEMOSTASIS |  | 46 | -0.28 | -1.23 | 0.129 | 0.462 | 1.000 | 3512 | tags=37%, list=27%, signal=50% |
| 104 | G\_PROTEIN\_SIGNALING\_COUPLED\_TO\_CAMP\_NUCLEOTIDE\_SECOND\_MESSENGER |  | 62 | -0.26 | -1.23 | 0.152 | 0.466 | 1.000 | 1898 | tags=21%, list=14%, signal=24% |
| 105 | GENERATION\_OF\_NEURONS |  | 65 | -0.25 | -1.23 | 0.123 | 0.470 | 1.000 | 3236 | tags=32%, list=25%, signal=43% |
| 106 | CAMP\_MEDIATED\_SIGNALING |  | 63 | -0.26 | -1.23 | 0.145 | 0.465 | 1.000 | 1898 | tags=21%, list=14%, signal=24% |
| 107 | I\_KAPPAB\_KINASE\_NF\_KAPPAB\_CASCADE |  | 88 | -0.24 | -1.22 | 0.133 | 0.468 | 1.000 | 2812 | tags=32%, list=21%, signal=40% |
| 108 | CELL\_RECOGNITION |  | 16 | -0.37 | -1.22 | 0.213 | 0.469 | 1.000 | 2767 | tags=38%, list=21%, signal=47% |
| 109 | ANATOMICAL\_STRUCTURE\_FORMATION |  | 52 | -0.26 | -1.22 | 0.166 | 0.467 | 1.000 | 1806 | tags=27%, list=14%, signal=31% |
| 110 | POSITIVE\_REGULATION\_OF\_PHOSPHORYLATION |  | 21 | -0.34 | -1.22 | 0.205 | 0.466 | 1.000 | 1656 | tags=29%, list=13%, signal=33% |
| 111 | LIPID\_METABOLIC\_PROCESS |  | 283 | -0.19 | -1.22 | 0.075 | 0.464 | 1.000 | 3964 | tags=39%, list=30%, signal=55% |
| 112 | ICOSANOID\_METABOLIC\_PROCESS |  | 16 | -0.38 | -1.22 | 0.226 | 0.463 | 1.000 | 3169 | tags=44%, list=24%, signal=58% |
| 113 | MYELOID\_CELL\_DIFFERENTIATION |  | 35 | -0.29 | -1.20 | 0.193 | 0.491 | 1.000 | 2747 | tags=31%, list=21%, signal=40% |
| 114 | NEURON\_DIFFERENTIATION |  | 58 | -0.26 | -1.20 | 0.159 | 0.490 | 1.000 | 3220 | tags=31%, list=25%, signal=41% |
| 115 | PROTEIN\_OLIGOMERIZATION |  | 37 | -0.29 | -1.20 | 0.185 | 0.487 | 1.000 | 2165 | tags=27%, list=17%, signal=32% |
| 116 | NEGATIVE\_REGULATION\_OF\_TRANSCRIPTION |  | 166 | -0.21 | -1.20 | 0.130 | 0.491 | 1.000 | 2724 | tags=28%, list=21%, signal=35% |
| 117 | POSITIVE\_REGULATION\_OF\_I\_KAPPAB\_KINASE\_NF\_KAPPAB\_CASCADE |  | 67 | -0.24 | -1.20 | 0.177 | 0.491 | 1.000 | 2812 | tags=33%, list=21%, signal=42% |
| 118 | POSITIVE\_REGULATION\_OF\_T\_CELL\_ACTIVATION |  | 20 | -0.33 | -1.19 | 0.238 | 0.497 | 1.000 | 3227 | tags=40%, list=25%, signal=53% |
| 119 | REGULATION\_OF\_MAPKKK\_CASCADE |  | 19 | -0.35 | -1.19 | 0.233 | 0.493 | 1.000 | 2146 | tags=32%, list=16%, signal=38% |
| 120 | ORGAN\_MORPHOGENESIS |  | 131 | -0.21 | -1.19 | 0.128 | 0.490 | 1.000 | 1394 | tags=18%, list=11%, signal=20% |
| 121 | PROTEIN\_AMINO\_ACID\_DEPHOSPHORYLATION |  | 60 | -0.25 | -1.19 | 0.179 | 0.488 | 1.000 | 1867 | tags=22%, list=14%, signal=25% |
| 122 | ANGIOGENESIS |  | 44 | -0.27 | -1.19 | 0.194 | 0.489 | 1.000 | 3153 | tags=39%, list=24%, signal=51% |
| 123 | CYTOKINE\_BIOSYNTHETIC\_PROCESS |  | 34 | -0.29 | -1.19 | 0.211 | 0.488 | 1.000 | 2772 | tags=35%, list=21%, signal=45% |
| 124 | DEVELOPMENTAL\_MATURATION |  | 18 | -0.35 | -1.18 | 0.233 | 0.507 | 1.000 | 2969 | tags=39%, list=23%, signal=50% |
| 125 | MUSCLE\_CELL\_DIFFERENTIATION |  | 21 | -0.32 | -1.18 | 0.236 | 0.504 | 1.000 | 3430 | tags=48%, list=26%, signal=64% |
| 126 | REGULATION\_OF\_PROTEIN\_METABOLIC\_PROCESS |  | 150 | -0.21 | -1.18 | 0.145 | 0.503 | 1.000 | 2819 | tags=28%, list=22%, signal=35% |
| 127 | RESPONSE\_TO\_BIOTIC\_STIMULUS |  | 103 | -0.22 | -1.17 | 0.174 | 0.515 | 1.000 | 2149 | tags=26%, list=16%, signal=31% |
| 128 | AMINE\_TRANSPORT |  | 36 | -0.28 | -1.17 | 0.235 | 0.511 | 1.000 | 700 | tags=17%, list=5%, signal=18% |
| 129 | NEGATIVE\_REGULATION\_OF\_CELL\_PROLIFERATION |  | 145 | -0.20 | -1.17 | 0.149 | 0.516 | 1.000 | 2534 | tags=26%, list=19%, signal=31% |
| 130 | DEPHOSPHORYLATION |  | 67 | -0.23 | -1.17 | 0.197 | 0.514 | 1.000 | 1867 | tags=21%, list=14%, signal=24% |
| 131 | PHOSPHORYLATION |  | 262 | -0.19 | -1.16 | 0.119 | 0.514 | 1.000 | 2453 | tags=24%, list=19%, signal=29% |
| 132 | PROTEIN\_PROCESSING |  | 41 | -0.27 | -1.16 | 0.229 | 0.535 | 1.000 | 3914 | tags=39%, list=30%, signal=55% |
| 133 | ION\_HOMEOSTASIS |  | 112 | -0.21 | -1.15 | 0.167 | 0.535 | 1.000 | 3066 | tags=31%, list=23%, signal=40% |
| 134 | POSITIVE\_REGULATION\_OF\_TRANSFERASE\_ACTIVITY |  | 71 | -0.23 | -1.15 | 0.221 | 0.544 | 1.000 | 2881 | tags=28%, list=22%, signal=36% |
| 135 | ACTIVATION\_OF\_MAPK\_ACTIVITY |  | 33 | -0.28 | -1.15 | 0.245 | 0.541 | 1.000 | 1713 | tags=27%, list=13%, signal=31% |
| 136 | REGULATION\_OF\_CELLULAR\_PROTEIN\_METABOLIC\_PROCESS |  | 139 | -0.20 | -1.15 | 0.179 | 0.539 | 1.000 | 2475 | tags=25%, list=19%, signal=31% |
| 137 | LOCOMOTORY\_BEHAVIOR |  | 84 | -0.23 | -1.15 | 0.234 | 0.535 | 1.000 | 2090 | tags=24%, list=16%, signal=28% |
| 138 | REGULATION\_OF\_MAP\_KINASE\_ACTIVITY |  | 56 | -0.25 | -1.15 | 0.231 | 0.532 | 1.000 | 1908 | tags=27%, list=15%, signal=31% |
| 139 | RESPONSE\_TO\_DRUG |  | 21 | -0.32 | -1.14 | 0.253 | 0.535 | 1.000 | 3187 | tags=43%, list=24%, signal=57% |
| 140 | NEURON\_DEVELOPMENT |  | 49 | -0.25 | -1.14 | 0.212 | 0.533 | 1.000 | 3702 | tags=35%, list=28%, signal=48% |
| 141 | STRIATED\_MUSCLE\_DEVELOPMENT |  | 36 | -0.27 | -1.14 | 0.261 | 0.535 | 1.000 | 3430 | tags=44%, list=26%, signal=60% |
| 142 | DETECTION\_OF\_EXTERNAL\_STIMULUS |  | 18 | -0.34 | -1.14 | 0.265 | 0.537 | 1.000 | 3767 | tags=39%, list=29%, signal=55% |
| 143 | BONE\_REMODELING |  | 28 | -0.30 | -1.14 | 0.275 | 0.534 | 1.000 | 2797 | tags=32%, list=21%, signal=41% |
| 144 | GENERATION\_OF\_PRECURSOR\_METABOLITES\_AND\_ENERGY |  | 120 | -0.21 | -1.14 | 0.197 | 0.532 | 1.000 | 2201 | tags=24%, list=17%, signal=29% |
| 145 | REGULATION\_OF\_DEVELOPMENTAL\_PROCESS |  | 387 | -0.17 | -1.13 | 0.114 | 0.539 | 1.000 | 3223 | tags=30%, list=25%, signal=39% |
| 146 | CYTOKINE\_METABOLIC\_PROCESS |  | 35 | -0.27 | -1.13 | 0.258 | 0.542 | 1.000 | 2772 | tags=34%, list=21%, signal=43% |
| 147 | HORMONE\_METABOLIC\_PROCESS |  | 29 | -0.29 | -1.13 | 0.262 | 0.546 | 1.000 | 3288 | tags=45%, list=25%, signal=60% |
| 148 | PEPTIDYL\_AMINO\_ACID\_MODIFICATION |  | 47 | -0.25 | -1.13 | 0.243 | 0.548 | 1.000 | 2424 | tags=28%, list=19%, signal=34% |
| 149 | GLYCEROPHOSPHOLIPID\_METABOLIC\_PROCESS |  | 39 | -0.26 | -1.12 | 0.295 | 0.546 | 1.000 | 4495 | tags=51%, list=34%, signal=78% |
| 150 | NEURITE\_DEVELOPMENT |  | 41 | -0.26 | -1.12 | 0.275 | 0.549 | 1.000 | 3702 | tags=34%, list=28%, signal=47% |
| 151 | POSITIVE\_REGULATION\_OF\_DEVELOPMENTAL\_PROCESS |  | 197 | -0.19 | -1.12 | 0.209 | 0.552 | 1.000 | 3915 | tags=40%, list=30%, signal=56% |
| 152 | POSITIVE\_REGULATION\_OF\_SECRETION |  | 18 | -0.32 | -1.11 | 0.298 | 0.585 | 1.000 | 4799 | tags=61%, list=37%, signal=96% |
| 153 | POSITIVE\_REGULATION\_OF\_CELLULAR\_METABOLIC\_PROCESS |  | 196 | -0.19 | -1.10 | 0.227 | 0.600 | 1.000 | 2935 | tags=28%, list=22%, signal=36% |
| 154 | ORGANIC\_ACID\_METABOLIC\_PROCESS |  | 162 | -0.19 | -1.10 | 0.249 | 0.599 | 1.000 | 3972 | tags=38%, list=30%, signal=53% |
| 155 | NEGATIVE\_REGULATION\_OF\_NUCLEOBASENUCLEOSIDENUCLEOTIDE\_AND\_NUCLEIC\_ACID\_METABOLIC\_PROCESS |  | 185 | -0.18 | -1.10 | 0.243 | 0.598 | 1.000 | 2724 | tags=27%, list=21%, signal=34% |
| 156 | NEGATIVE\_REGULATION\_OF\_METABOLIC\_PROCESS |  | 232 | -0.18 | -1.10 | 0.234 | 0.596 | 1.000 | 2728 | tags=25%, list=21%, signal=32% |
| 157 | POSITIVE\_REGULATION\_OF\_METABOLIC\_PROCESS |  | 201 | -0.18 | -1.10 | 0.236 | 0.592 | 1.000 | 2935 | tags=28%, list=22%, signal=35% |
| 158 | MEMBRANE\_LIPID\_METABOLIC\_PROCESS |  | 85 | -0.21 | -1.09 | 0.278 | 0.591 | 1.000 | 3399 | tags=35%, list=26%, signal=47% |
| 159 | SKELETAL\_DEVELOPMENT |  | 91 | -0.21 | -1.09 | 0.305 | 0.588 | 1.000 | 3430 | tags=34%, list=26%, signal=46% |
| 160 | VASCULATURE\_DEVELOPMENT |  | 50 | -0.24 | -1.09 | 0.302 | 0.584 | 1.000 | 1806 | tags=24%, list=14%, signal=28% |
| 161 | CARBOXYLIC\_ACID\_METABOLIC\_PROCESS |  | 160 | -0.19 | -1.09 | 0.252 | 0.583 | 1.000 | 3972 | tags=38%, list=30%, signal=54% |
| 162 | ANTI\_APOPTOSIS |  | 107 | -0.20 | -1.09 | 0.271 | 0.581 | 1.000 | 2215 | tags=26%, list=17%, signal=31% |
| 163 | ELECTRON\_TRANSPORT\_GO\_0006118 |  | 50 | -0.24 | -1.09 | 0.291 | 0.586 | 1.000 | 2201 | tags=26%, list=17%, signal=31% |
| 164 | CELL\_MATURATION |  | 16 | -0.33 | -1.09 | 0.347 | 0.584 | 1.000 | 2969 | tags=38%, list=23%, signal=48% |
| 165 | CYTOKINE\_SECRETION |  | 15 | -0.34 | -1.09 | 0.342 | 0.582 | 1.000 | 2767 | tags=40%, list=21%, signal=51% |
| 166 | MEMBRANE\_ORGANIZATION\_AND\_BIOGENESIS |  | 124 | -0.19 | -1.08 | 0.262 | 0.589 | 1.000 | 3707 | tags=35%, list=28%, signal=49% |
| 167 | AXONOGENESIS |  | 33 | -0.27 | -1.08 | 0.333 | 0.590 | 1.000 | 3702 | tags=36%, list=28%, signal=51% |
| 168 | CELLULAR\_LIPID\_METABOLIC\_PROCESS |  | 220 | -0.18 | -1.08 | 0.247 | 0.587 | 1.000 | 3964 | tags=38%, list=30%, signal=54% |
| 169 | POST\_TRANSLATIONAL\_PROTEIN\_MODIFICATION |  | 409 | -0.16 | -1.08 | 0.210 | 0.590 | 1.000 | 2477 | tags=22%, list=19%, signal=27% |
| 170 | POSITIVE\_REGULATION\_OF\_TRANSCRIPTION |  | 124 | -0.19 | -1.08 | 0.276 | 0.589 | 1.000 | 2935 | tags=27%, list=22%, signal=35% |
| 171 | NEGATIVE\_REGULATION\_OF\_RNA\_METABOLIC\_PROCESS |  | 114 | -0.20 | -1.08 | 0.319 | 0.592 | 1.000 | 2724 | tags=27%, list=21%, signal=34% |
| 172 | POSITIVE\_REGULATION\_OF\_MAP\_KINASE\_ACTIVITY |  | 39 | -0.26 | -1.08 | 0.329 | 0.590 | 1.000 | 1713 | tags=26%, list=13%, signal=29% |
| 173 | POSITIVE\_REGULATION\_OF\_TRANSCRIPTION\_FACTOR\_ACTIVITY |  | 17 | -0.31 | -1.08 | 0.346 | 0.590 | 1.000 | 4014 | tags=53%, list=31%, signal=76% |
| 174 | POSITIVE\_REGULATION\_OF\_PROTEIN\_MODIFICATION\_PROCESS |  | 24 | -0.28 | -1.07 | 0.327 | 0.591 | 1.000 | 1656 | tags=25%, list=13%, signal=29% |
| 175 | TISSUE\_REMODELING |  | 29 | -0.27 | -1.07 | 0.361 | 0.595 | 1.000 | 2797 | tags=31%, list=21%, signal=39% |
| 176 | POSITIVE\_REGULATION\_OF\_CATALYTIC\_ACTIVITY |  | 139 | -0.19 | -1.07 | 0.282 | 0.603 | 1.000 | 2334 | tags=22%, list=18%, signal=26% |
| 177 | NEGATIVE\_REGULATION\_OF\_TRANSCRIPTION\_DNA\_DEPENDENT |  | 114 | -0.20 | -1.06 | 0.320 | 0.607 | 1.000 | 2724 | tags=27%, list=21%, signal=34% |
| 178 | GLYCOPROTEIN\_BIOSYNTHETIC\_PROCESS |  | 67 | -0.22 | -1.06 | 0.351 | 0.616 | 1.000 | 3719 | tags=36%, list=28%, signal=50% |
| 179 | NEGATIVE\_REGULATION\_OF\_CELLULAR\_METABOLIC\_PROCESS |  | 229 | -0.17 | -1.06 | 0.282 | 0.617 | 1.000 | 2728 | tags=25%, list=21%, signal=31% |
| 180 | NEUROGENESIS |  | 75 | -0.21 | -1.06 | 0.347 | 0.617 | 1.000 | 3236 | tags=31%, list=25%, signal=41% |
| 181 | CELL\_PROLIFERATION\_GO\_0008283 |  | 466 | -0.16 | -1.06 | 0.273 | 0.616 | 1.000 | 2553 | tags=23%, list=20%, signal=28% |
| 182 | AMINO\_ACID\_DERIVATIVE\_METABOLIC\_PROCESS |  | 23 | -0.28 | -1.05 | 0.371 | 0.616 | 1.000 | 4072 | tags=48%, list=31%, signal=69% |
| 183 | ORGANIC\_ACID\_TRANSPORT |  | 39 | -0.24 | -1.05 | 0.353 | 0.625 | 1.000 | 903 | tags=18%, list=7%, signal=19% |
| 184 | REGULATION\_OF\_BIOLOGICAL\_QUALITY |  | 364 | -0.16 | -1.05 | 0.300 | 0.627 | 1.000 | 3412 | tags=28%, list=26%, signal=37% |
| 185 | NEGATIVE\_REGULATION\_OF\_TRANSCRIPTION\_FROM\_RNA\_POLYMERASE\_II\_PROMOTER |  | 76 | -0.21 | -1.05 | 0.357 | 0.623 | 1.000 | 2293 | tags=25%, list=18%, signal=30% |
| 186 | CARBOXYLIC\_ACID\_TRANSPORT |  | 39 | -0.24 | -1.05 | 0.381 | 0.626 | 1.000 | 903 | tags=18%, list=7%, signal=19% |
| 187 | REGULATION\_OF\_TRANSCRIPTION |  | 498 | -0.16 | -1.04 | 0.312 | 0.630 | 1.000 | 2747 | tags=24%, list=21%, signal=30% |
| 188 | REGULATION\_OF\_CYTOKINE\_BIOSYNTHETIC\_PROCESS |  | 31 | -0.26 | -1.04 | 0.378 | 0.633 | 1.000 | 2772 | tags=32%, list=21%, signal=41% |
| 189 | PROTEIN\_AUTOPROCESSING |  | 24 | -0.27 | -1.04 | 0.383 | 0.640 | 1.000 | 5305 | tags=58%, list=41%, signal=98% |
| 190 | AMINO\_ACID\_METABOLIC\_PROCESS |  | 73 | -0.21 | -1.03 | 0.372 | 0.647 | 1.000 | 2290 | tags=26%, list=17%, signal=31% |
| 191 | RESPONSE\_TO\_CHEMICAL\_STIMULUS |  | 271 | -0.17 | -1.03 | 0.374 | 0.648 | 1.000 | 1694 | tags=18%, list=13%, signal=20% |
| 192 | CELLULAR\_PROTEIN\_COMPLEX\_ASSEMBLY |  | 28 | -0.26 | -1.03 | 0.411 | 0.652 | 1.000 | 936 | tags=18%, list=7%, signal=19% |
| 193 | NEGATIVE\_REGULATION\_OF\_DEVELOPMENTAL\_PROCESS |  | 177 | -0.18 | -1.03 | 0.383 | 0.655 | 1.000 | 2217 | tags=23%, list=17%, signal=28% |
| 194 | POSITIVE\_REGULATION\_OF\_DNA\_BINDING |  | 18 | -0.30 | -1.03 | 0.419 | 0.654 | 1.000 | 4800 | tags=61%, list=37%, signal=96% |
| 195 | PROTEIN\_AMINO\_ACID\_AUTOPHOSPHORYLATION |  | 24 | -0.27 | -1.02 | 0.434 | 0.653 | 1.000 | 5305 | tags=58%, list=41%, signal=98% |
| 196 | MACROMOLECULE\_BIOSYNTHETIC\_PROCESS |  | 267 | -0.17 | -1.02 | 0.401 | 0.651 | 1.000 | 2959 | tags=27%, list=23%, signal=34% |
| 197 | DETECTION\_OF\_STIMULUS\_INVOLVED\_IN\_SENSORY\_PERCEPTION |  | 15 | -0.31 | -1.02 | 0.441 | 0.656 | 1.000 | 9017 | tags=100%, list=69%, signal=321% |
| 198 | NERVOUS\_SYSTEM\_DEVELOPMENT |  | 328 | -0.16 | -1.02 | 0.401 | 0.657 | 1.000 | 4161 | tags=35%, list=32%, signal=50% |
| 199 | CELLULAR\_COMPONENT\_ASSEMBLY |  | 272 | -0.17 | -1.02 | 0.366 | 0.657 | 1.000 | 2832 | tags=26%, list=22%, signal=32% |
| 200 | MYOBLAST\_DIFFERENTIATION |  | 16 | -0.30 | -1.01 | 0.440 | 0.678 | 1.000 | 3430 | tags=50%, list=26%, signal=68% |
| 201 | PHAGOCYTOSIS |  | 16 | -0.31 | -1.01 | 0.424 | 0.675 | 1.000 | 5234 | tags=69%, list=40%, signal=114% |
| 202 | REGULATION\_OF\_JNK\_ACTIVITY |  | 18 | -0.29 | -1.01 | 0.438 | 0.674 | 1.000 | 1713 | tags=28%, list=13%, signal=32% |
| 203 | RHYTHMIC\_PROCESS |  | 23 | -0.27 | -1.01 | 0.450 | 0.679 | 1.000 | 1937 | tags=26%, list=15%, signal=31% |
| 204 | POSITIVE\_REGULATION\_OF\_NUCLEOBASENUCLEOSIDENUCLEOTIDE\_AND\_NUCLEIC\_ACID\_METABOLIC\_PROCESS |  | 134 | -0.18 | -1.00 | 0.453 | 0.679 | 1.000 | 2935 | tags=27%, list=22%, signal=34% |
| 205 | HEART\_DEVELOPMENT |  | 33 | -0.25 | -1.00 | 0.445 | 0.677 | 1.000 | 3457 | tags=36%, list=26%, signal=49% |
| 206 | CHEMICAL\_HOMEOSTASIS |  | 136 | -0.18 | -1.00 | 0.442 | 0.678 | 1.000 | 3066 | tags=28%, list=23%, signal=36% |
| 207 | AMINO\_ACID\_CATABOLIC\_PROCESS |  | 23 | -0.27 | -1.00 | 0.452 | 0.677 | 1.000 | 2290 | tags=30%, list=17%, signal=37% |
| 208 | ACTIN\_FILAMENT\_ORGANIZATION |  | 21 | -0.27 | -1.00 | 0.455 | 0.681 | 1.000 | 2728 | tags=33%, list=21%, signal=42% |
| 209 | POSITIVE\_REGULATION\_OF\_CELLULAR\_COMPONENT\_ORGANIZATION\_AND\_BIOGENESIS |  | 28 | -0.25 | -1.00 | 0.475 | 0.683 | 1.000 | 3707 | tags=39%, list=28%, signal=55% |
| 210 | CELLULAR\_HOMEOSTASIS |  | 121 | -0.18 | -0.99 | 0.467 | 0.685 | 1.000 | 4555 | tags=45%, list=35%, signal=68% |
| 211 | PROTEIN\_SECRETION |  | 28 | -0.25 | -0.99 | 0.450 | 0.685 | 1.000 | 3525 | tags=36%, list=27%, signal=49% |
| 212 | NEGATIVE\_REGULATION\_OF\_CELL\_DIFFERENTIATION |  | 24 | -0.26 | -0.99 | 0.463 | 0.699 | 1.000 | 2498 | tags=25%, list=19%, signal=31% |
| 213 | FATTY\_ACID\_OXIDATION |  | 17 | -0.29 | -0.99 | 0.474 | 0.697 | 1.000 | 4232 | tags=53%, list=32%, signal=78% |
| 214 | REGULATION\_OF\_MYELOID\_CELL\_DIFFERENTIATION |  | 19 | -0.28 | -0.99 | 0.471 | 0.698 | 1.000 | 2747 | tags=32%, list=21%, signal=40% |
| 215 | MAPKKK\_CASCADE\_GO\_0000165 |  | 90 | -0.19 | -0.98 | 0.501 | 0.707 | 1.000 | 1908 | tags=20%, list=15%, signal=23% |
| 216 | REGULATION\_OF\_PHOSPHORYLATION |  | 42 | -0.22 | -0.98 | 0.489 | 0.711 | 1.000 | 2424 | tags=26%, list=19%, signal=32% |
| 217 | AMINO\_ACID\_AND\_DERIVATIVE\_METABOLIC\_PROCESS |  | 96 | -0.18 | -0.97 | 0.500 | 0.718 | 1.000 | 2290 | tags=24%, list=17%, signal=29% |
| 218 | TISSUE\_DEVELOPMENT |  | 126 | -0.18 | -0.97 | 0.510 | 0.715 | 1.000 | 3939 | tags=37%, list=30%, signal=52% |
| 219 | TRANSLATION |  | 149 | -0.17 | -0.97 | 0.552 | 0.731 | 1.000 | 2633 | tags=26%, list=20%, signal=32% |
| 220 | VITAMIN\_METABOLIC\_PROCESS |  | 15 | -0.30 | -0.96 | 0.492 | 0.734 | 1.000 | 564 | tags=20%, list=4%, signal=21% |
| 221 | HOMEOSTATIC\_PROCESS |  | 179 | -0.17 | -0.96 | 0.553 | 0.742 | 1.000 | 3066 | tags=27%, list=23%, signal=35% |
| 222 | REGULATION\_OF\_BINDING |  | 46 | -0.21 | -0.96 | 0.496 | 0.752 | 1.000 | 2475 | tags=28%, list=19%, signal=35% |
| 223 | MACROMOLECULAR\_COMPLEX\_ASSEMBLY |  | 254 | -0.16 | -0.95 | 0.597 | 0.755 | 1.000 | 2711 | tags=24%, list=21%, signal=30% |
| 224 | CELL\_MIGRATION |  | 82 | -0.19 | -0.95 | 0.573 | 0.762 | 1.000 | 2424 | tags=22%, list=19%, signal=27% |
| 225 | CELL\_CELL\_ADHESION |  | 72 | -0.19 | -0.95 | 0.543 | 0.761 | 1.000 | 4560 | tags=46%, list=35%, signal=70% |
| 226 | AMINE\_CATABOLIC\_PROCESS |  | 25 | -0.25 | -0.94 | 0.531 | 0.772 | 1.000 | 2290 | tags=28%, list=17%, signal=34% |
| 227 | RESPONSE\_TO\_NUTRIENT |  | 17 | -0.28 | -0.94 | 0.531 | 0.772 | 1.000 | 2230 | tags=29%, list=17%, signal=35% |
| 228 | SKELETAL\_MUSCLE\_DEVELOPMENT |  | 28 | -0.24 | -0.94 | 0.555 | 0.782 | 1.000 | 3430 | tags=43%, list=26%, signal=58% |
| 229 | SULFUR\_METABOLIC\_PROCESS |  | 30 | -0.24 | -0.93 | 0.554 | 0.785 | 1.000 | 2844 | tags=30%, list=22%, signal=38% |
| 230 | NITROGEN\_COMPOUND\_CATABOLIC\_PROCESS |  | 27 | -0.24 | -0.93 | 0.526 | 0.795 | 1.000 | 2290 | tags=26%, list=17%, signal=31% |
| 231 | VESICLE\_MEDIATED\_TRANSPORT |  | 174 | -0.16 | -0.93 | 0.649 | 0.796 | 1.000 | 3958 | tags=35%, list=30%, signal=50% |
| 232 | ANATOMICAL\_STRUCTURE\_MORPHOGENESIS |  | 336 | -0.14 | -0.92 | 0.700 | 0.807 | 1.000 | 3278 | tags=27%, list=25%, signal=35% |
| 233 | GOLGI\_VESICLE\_TRANSPORT |  | 42 | -0.21 | -0.91 | 0.592 | 0.823 | 1.000 | 3957 | tags=40%, list=30%, signal=58% |
| 234 | POSITIVE\_REGULATION\_OF\_CASPASE\_ACTIVITY |  | 28 | -0.23 | -0.91 | 0.588 | 0.823 | 1.000 | 1818 | tags=25%, list=14%, signal=29% |
| 235 | REGULATION\_OF\_TRANSCRIPTIONDNA\_DEPENDENT |  | 412 | -0.14 | -0.91 | 0.795 | 0.820 | 1.000 | 2747 | tags=24%, list=21%, signal=29% |
| 236 | REGULATION\_OF\_G\_PROTEIN\_COUPLED\_RECEPTOR\_PROTEIN\_SIGNALING\_PATHWAY |  | 23 | -0.25 | -0.91 | 0.575 | 0.821 | 1.000 | 1134 | tags=17%, list=9%, signal=19% |
| 237 | POSITIVE\_REGULATION\_OF\_TRANSCRIPTION\_FROM\_RNA\_POLYMERASE\_II\_PROMOTER |  | 60 | -0.19 | -0.91 | 0.628 | 0.825 | 1.000 | 2894 | tags=28%, list=22%, signal=36% |
| 238 | AXON\_GUIDANCE |  | 18 | -0.27 | -0.91 | 0.589 | 0.826 | 1.000 | 3644 | tags=39%, list=28%, signal=54% |
| 239 | CENTRAL\_NERVOUS\_SYSTEM\_DEVELOPMENT |  | 105 | -0.17 | -0.90 | 0.691 | 0.836 | 1.000 | 4223 | tags=38%, list=32%, signal=56% |
| 240 | REGULATION\_OF\_CELLULAR\_COMPONENT\_ORGANIZATION\_AND\_BIOGENESIS |  | 102 | -0.17 | -0.90 | 0.706 | 0.841 | 1.000 | 3707 | tags=32%, list=28%, signal=45% |
| 241 | REGULATION\_OF\_PROTEIN\_SECRETION |  | 19 | -0.26 | -0.90 | 0.613 | 0.838 | 1.000 | 2767 | tags=32%, list=21%, signal=40% |
| 242 | REGULATION\_OF\_PROTEIN\_MODIFICATION\_PROCESS |  | 37 | -0.22 | -0.90 | 0.615 | 0.836 | 1.000 | 2424 | tags=24%, list=19%, signal=30% |
| 243 | REGULATION\_OF\_TRANSLATIONAL\_INITIATION |  | 25 | -0.24 | -0.90 | 0.610 | 0.833 | 1.000 | 1140 | tags=20%, list=9%, signal=22% |
| 244 | SODIUM\_ION\_TRANSPORT |  | 17 | -0.27 | -0.90 | 0.600 | 0.833 | 1.000 | 9608 | tags=100%, list=73%, signal=375% |
| 245 | T\_CELL\_PROLIFERATION |  | 17 | -0.26 | -0.89 | 0.595 | 0.838 | 1.000 | 2772 | tags=35%, list=21%, signal=45% |
| 246 | SECRETION\_BY\_CELL |  | 100 | -0.17 | -0.89 | 0.709 | 0.837 | 1.000 | 4407 | tags=40%, list=34%, signal=60% |
| 247 | AEROBIC\_RESPIRATION |  | 15 | -0.28 | -0.89 | 0.634 | 0.847 | 1.000 | 786 | tags=20%, list=6%, signal=21% |
| 248 | CELL\_CYCLE\_ARREST\_GO\_0007050 |  | 52 | -0.19 | -0.88 | 0.667 | 0.852 | 1.000 | 2133 | tags=25%, list=16%, signal=30% |
| 249 | REGULATION\_OF\_TRANSCRIPTION\_FACTOR\_ACTIVITY |  | 30 | -0.22 | -0.88 | 0.644 | 0.855 | 1.000 | 4274 | tags=47%, list=33%, signal=69% |
| 250 | CYCLIC\_NUCLEOTIDE\_MEDIATED\_SIGNALING |  | 97 | -0.17 | -0.88 | 0.718 | 0.852 | 1.000 | 1214 | tags=12%, list=9%, signal=14% |
| 251 | AMINE\_METABOLIC\_PROCESS |  | 128 | -0.16 | -0.88 | 0.721 | 0.850 | 1.000 | 3538 | tags=30%, list=27%, signal=41% |
| 252 | CARBOHYDRATE\_BIOSYNTHETIC\_PROCESS |  | 35 | -0.21 | -0.88 | 0.650 | 0.847 | 1.000 | 3972 | tags=37%, list=30%, signal=53% |
| 253 | REGULATION\_OF\_GROWTH |  | 48 | -0.20 | -0.88 | 0.670 | 0.846 | 1.000 | 4176 | tags=40%, list=32%, signal=58% |
| 254 | EPIDERMIS\_DEVELOPMENT |  | 66 | -0.18 | -0.88 | 0.700 | 0.847 | 1.000 | 1136 | tags=17%, list=9%, signal=18% |
| 255 | PHOSPHOINOSITIDE\_METABOLIC\_PROCESS |  | 25 | -0.23 | -0.87 | 0.657 | 0.857 | 1.000 | 4495 | tags=52%, list=34%, signal=79% |
| 256 | CELLULAR\_MORPHOGENESIS\_DURING\_DIFFERENTIATION |  | 38 | -0.20 | -0.87 | 0.675 | 0.864 | 1.000 | 3702 | tags=32%, list=28%, signal=44% |
| 257 | REGULATION\_OF\_TRANSLATION |  | 76 | -0.17 | -0.86 | 0.754 | 0.867 | 1.000 | 2475 | tags=24%, list=19%, signal=29% |
| 258 | G\_PROTEIN\_SIGNALING\_COUPLED\_TO\_CYCLIC\_NUCLEOTIDE\_SECOND\_MESSENGER |  | 96 | -0.17 | -0.86 | 0.753 | 0.870 | 1.000 | 1214 | tags=13%, list=9%, signal=14% |
| 259 | RESPONSE\_TO\_NUTRIENT\_LEVELS |  | 27 | -0.22 | -0.85 | 0.643 | 0.878 | 1.000 | 2524 | tags=26%, list=19%, signal=32% |
| 260 | POSITIVE\_REGULATION\_OF\_BINDING |  | 19 | -0.25 | -0.85 | 0.655 | 0.879 | 1.000 | 4014 | tags=47%, list=31%, signal=68% |
| 261 | EXTRACELLULAR\_STRUCTURE\_ORGANIZATION\_AND\_BIOGENESIS |  | 23 | -0.23 | -0.85 | 0.661 | 0.876 | 1.000 | 3367 | tags=39%, list=26%, signal=53% |
| 262 | ACTIVATION\_OF\_PROTEIN\_KINASE\_ACTIVITY |  | 23 | -0.23 | -0.85 | 0.639 | 0.878 | 1.000 | 4719 | tags=39%, list=36%, signal=61% |
| 263 | ENDOSOME\_TRANSPORT |  | 22 | -0.23 | -0.85 | 0.670 | 0.884 | 1.000 | 3942 | tags=41%, list=30%, signal=58% |
| 264 | REGULATION\_OF\_DNA\_BINDING |  | 36 | -0.20 | -0.83 | 0.729 | 0.904 | 1.000 | 2475 | tags=28%, list=19%, signal=34% |
| 265 | SPHINGOLIPID\_METABOLIC\_PROCESS |  | 23 | -0.22 | -0.83 | 0.733 | 0.905 | 1.000 | 3267 | tags=35%, list=25%, signal=46% |
| 266 | CELL\_CELL\_SIGNALING |  | 372 | -0.13 | -0.83 | 0.951 | 0.910 | 1.000 | 3731 | tags=29%, list=29%, signal=39% |
| 267 | INSULIN\_RECEPTOR\_SIGNALING\_PATHWAY |  | 16 | -0.24 | -0.81 | 0.722 | 0.935 | 1.000 | 3040 | tags=31%, list=23%, signal=41% |
| 268 | POSITIVE\_REGULATION\_OF\_JNK\_ACTIVITY |  | 16 | -0.24 | -0.81 | 0.721 | 0.932 | 1.000 | 1713 | tags=25%, list=13%, signal=29% |
| 269 | SECOND\_MESSENGER\_MEDIATED\_SIGNALING |  | 139 | -0.14 | -0.80 | 0.927 | 0.949 | 1.000 | 2038 | tags=16%, list=16%, signal=19% |
| 270 | NEGATIVE\_REGULATION\_OF\_CELLULAR\_COMPONENT\_ORGANIZATION\_AND\_BIOGENESIS |  | 26 | -0.21 | -0.80 | 0.787 | 0.953 | 1.000 | 1342 | tags=15%, list=10%, signal=17% |
| 271 | POSITIVE\_REGULATION\_OF\_TRANSCRIPTIONDNA\_DEPENDENT |  | 105 | -0.15 | -0.79 | 0.917 | 0.957 | 1.000 | 4319 | tags=37%, list=33%, signal=55% |
| 272 | G\_PROTEIN\_SIGNALING\_COUPLED\_TO\_IP3\_SECOND\_MESSENGERPHOSPHOLIPASE\_C\_ACTIVATING |  | 39 | -0.19 | -0.79 | 0.838 | 0.955 | 1.000 | 2493 | tags=23%, list=19%, signal=28% |
| 273 | AMINE\_BIOSYNTHETIC\_PROCESS |  | 15 | -0.24 | -0.79 | 0.739 | 0.954 | 1.000 | 318 | tags=13%, list=2%, signal=14% |
| 274 | REPRODUCTIVE\_PROCESS |  | 133 | -0.14 | -0.78 | 0.928 | 0.963 | 1.000 | 3743 | tags=33%, list=29%, signal=46% |
| 275 | G\_PROTEIN\_COUPLED\_RECEPTOR\_PROTEIN\_SIGNALING\_PATHWAY |  | 300 | -0.12 | -0.78 | 0.992 | 0.960 | 1.000 | 4555 | tags=35%, list=35%, signal=53% |
| 276 | BRAIN\_DEVELOPMENT |  | 39 | -0.18 | -0.78 | 0.808 | 0.959 | 1.000 | 4134 | tags=41%, list=32%, signal=60% |
| 277 | NEGATIVE\_REGULATION\_OF\_GROWTH |  | 35 | -0.19 | -0.77 | 0.833 | 0.965 | 1.000 | 4176 | tags=40%, list=32%, signal=59% |
| 278 | NEGATIVE\_REGULATION\_OF\_CELLULAR\_PROTEIN\_METABOLIC\_PROCESS |  | 41 | -0.17 | -0.77 | 0.853 | 0.964 | 1.000 | 2819 | tags=24%, list=22%, signal=31% |
| 279 | RESPONSE\_TO\_EXTRACELLULAR\_STIMULUS |  | 29 | -0.19 | -0.77 | 0.858 | 0.963 | 1.000 | 2524 | tags=24%, list=19%, signal=30% |
| 280 | PROTEIN\_LOCALIZATION |  | 184 | -0.13 | -0.76 | 0.971 | 0.971 | 1.000 | 3571 | tags=27%, list=27%, signal=37% |
| 281 | METAL\_ION\_TRANSPORT |  | 102 | -0.14 | -0.76 | 0.940 | 0.969 | 1.000 | 4867 | tags=43%, list=37%, signal=68% |
| 282 | POSITIVE\_REGULATION\_OF\_RNA\_METABOLIC\_PROCESS |  | 107 | -0.14 | -0.75 | 0.953 | 0.981 | 1.000 | 4319 | tags=36%, list=33%, signal=54% |
| 283 | REGULATION\_OF\_MUSCLE\_CONTRACTION |  | 18 | -0.22 | -0.74 | 0.827 | 0.990 | 1.000 | 2669 | tags=33%, list=20%, signal=42% |
| 284 | POTASSIUM\_ION\_TRANSPORT |  | 52 | -0.16 | -0.74 | 0.896 | 0.988 | 1.000 | 4637 | tags=40%, list=35%, signal=62% |
| 285 | CARBOHYDRATE\_CATABOLIC\_PROCESS |  | 20 | -0.21 | -0.73 | 0.858 | 0.993 | 1.000 | 3972 | tags=35%, list=30%, signal=50% |
| 286 | CELLULAR\_CARBOHYDRATE\_CATABOLIC\_PROCESS |  | 20 | -0.21 | -0.73 | 0.827 | 0.990 | 1.000 | 3972 | tags=35%, list=30%, signal=50% |
| 287 | G\_PROTEIN\_SIGNALING\_ADENYLATE\_CYCLASE\_ACTIVATING\_PATHWAY |  | 24 | -0.19 | -0.73 | 0.861 | 0.987 | 1.000 | 1214 | tags=13%, list=9%, signal=14% |
| 288 | PROTEIN\_POLYMERIZATION |  | 17 | -0.21 | -0.72 | 0.856 | 0.987 | 1.000 | 448 | tags=12%, list=3%, signal=12% |
| 289 | CARBOHYDRATE\_METABOLIC\_PROCESS |  | 152 | -0.13 | -0.72 | 0.986 | 0.984 | 1.000 | 4683 | tags=39%, list=36%, signal=60% |
| 290 | POSITIVE\_REGULATION\_OF\_TRANSPORT |  | 18 | -0.21 | -0.72 | 0.845 | 0.981 | 1.000 | 4923 | tags=56%, list=38%, signal=89% |
| 291 | CATION\_TRANSPORT |  | 130 | -0.13 | -0.72 | 0.977 | 0.978 | 1.000 | 4637 | tags=40%, list=35%, signal=61% |
| 292 | REGULATION\_OF\_CYTOKINE\_PRODUCTION |  | 21 | -0.20 | -0.72 | 0.865 | 0.977 | 1.000 | 3037 | tags=29%, list=23%, signal=37% |
| 293 | ESTABLISHMENT\_AND\_OR\_MAINTENANCE\_OF\_CELL\_POLARITY |  | 19 | -0.21 | -0.72 | 0.862 | 0.974 | 1.000 | 1449 | tags=16%, list=11%, signal=18% |
| 294 | NEGATIVE\_REGULATION\_OF\_PROTEIN\_METABOLIC\_PROCESS |  | 44 | -0.16 | -0.71 | 0.931 | 0.975 | 1.000 | 2819 | tags=23%, list=22%, signal=29% |
| 295 | ION\_TRANSPORT |  | 165 | -0.12 | -0.71 | 0.995 | 0.978 | 1.000 | 4658 | tags=39%, list=36%, signal=60% |
| 296 | PHOSPHOINOSITIDE\_MEDIATED\_SIGNALING |  | 42 | -0.16 | -0.71 | 0.901 | 0.975 | 1.000 | 2493 | tags=21%, list=19%, signal=26% |
| 297 | DI\_\_\_TRI\_VALENT\_INORGANIC\_CATION\_TRANSPORT |  | 27 | -0.18 | -0.70 | 0.906 | 0.976 | 1.000 | 1110 | tags=15%, list=8%, signal=16% |
| 298 | SECRETION |  | 157 | -0.12 | -0.70 | 0.993 | 0.973 | 1.000 | 3659 | tags=29%, list=28%, signal=40% |
| 299 | SECRETORY\_PATHWAY |  | 72 | -0.14 | -0.70 | 0.960 | 0.974 | 1.000 | 4954 | tags=46%, list=38%, signal=73% |
| 300 | NEGATIVE\_REGULATION\_OF\_MULTICELLULAR\_ORGANISMAL\_PROCESS |  | 27 | -0.18 | -0.68 | 0.890 | 0.981 | 1.000 | 2079 | tags=22%, list=16%, signal=26% |
| 301 | PATTERN\_SPECIFICATION\_PROCESS |  | 27 | -0.16 | -0.65 | 0.943 | 1.000 | 1.000 | 956 | tags=11%, list=7%, signal=12% |
| 302 | INORGANIC\_ANION\_TRANSPORT |  | 16 | -0.19 | -0.65 | 0.920 | 0.998 | 1.000 | 574 | tags=13%, list=4%, signal=13% |
| 303 | REGULATION\_OF\_CELL\_MIGRATION |  | 23 | -0.17 | -0.64 | 0.943 | 0.998 | 1.000 | 5042 | tags=43%, list=39%, signal=71% |
| 304 | NUCLEOTIDE\_EXCISION\_REPAIR |  | 19 | -0.18 | -0.64 | 0.943 | 0.995 | 1.000 | 4091 | tags=37%, list=31%, signal=54% |
| 305 | EXCRETION |  | 35 | -0.15 | -0.63 | 0.954 | 0.994 | 1.000 | 2360 | tags=20%, list=18%, signal=24% |
| 306 | LIPID\_HOMEOSTASIS |  | 15 | -0.19 | -0.63 | 0.935 | 0.994 | 1.000 | 972 | tags=13%, list=7%, signal=14% |
| 307 | DETECTION\_OF\_ABIOTIC\_STIMULUS |  | 16 | -0.18 | -0.61 | 0.936 | 0.998 | 1.000 | 5108 | tags=50%, list=39%, signal=82% |
| 308 | AMINO\_SUGAR\_METABOLIC\_PROCESS |  | 15 | -0.18 | -0.59 | 0.948 | 1.000 | 1.000 | 4329 | tags=40%, list=33%, signal=60% |
| 309 | REGULATION\_OF\_CELL\_GROWTH |  | 39 | -0.13 | -0.57 | 0.993 | 1.000 | 1.000 | 4176 | tags=33%, list=32%, signal=49% |
| 310 | CALCIUM\_ION\_TRANSPORT |  | 23 | -0.15 | -0.56 | 0.968 | 1.000 | 1.000 | 1110 | tags=13%, list=8%, signal=14% |
| 311 | RESPONSE\_TO\_LIGHT\_STIMULUS |  | 40 | -0.13 | -0.56 | 0.986 | 0.999 | 1.000 | 2829 | tags=23%, list=22%, signal=29% |
| 312 | FEMALE\_GAMETE\_GENERATION |  | 15 | -0.16 | -0.53 | 0.987 | 1.000 | 1.000 | 10940 | tags=100%, list=84%, signal=608% |
| 313 | MONOVALENT\_INORGANIC\_CATION\_TRANSPORT |  | 83 | -0.10 | -0.49 | 1.000 | 1.000 | 1.000 | 4637 | tags=36%, list=35%, signal=56% |
| 314 | REGULATION\_OF\_ACTION\_POTENTIAL |  | 16 | -0.15 | -0.48 | 0.996 | 0.998 | 1.000 | 3635 | tags=31%, list=28%, signal=43% |
Table: Gene sets enriched in phenotype **na**[plain text format]****

  
