# Supplementary material for: Multiple-input multiple-output causal strategies for gene selection
Source: BMC Bioinformatics. 2011 Nov 25;12:458. doi: 10.1186/1471-2105-12-458 (PMC3323860; doi:10.1186/1471-2105-12-458)
Supplement: Additional file 3 — Archive containing the output files computed by the preranked GSEA for λ ∈ {0.6,0.7,0.8,0.9,1.0,2.0} (GSEA_MIMO_part2.zip). [file 1471-2105-12-458-S3.ZIP › mFS10_entrez_mimo.GseaPreranked.1316039488125/gsea_report_for_na_pos_1316039488125.html]

Report for na\_pos 1316039488125 [GSEA]

| GS  follow link to MSigDB | GS DETAILS | SIZE | ES | NES | NOM p-val | FDR q-val | FWER p-val | RANK AT MAX | LEADING EDGE || 1 | CELL\_CYCLE\_PROCESS |  | 169 | 0.48 | 2.66 | 0.000 | 0.000 | 0.000 | 2360 | tags=47%, list=18%, signal=56% |
| 2 | MITOTIC\_CELL\_CYCLE |  | 134 | 0.49 | 2.65 | 0.000 | 0.000 | 0.000 | 2360 | tags=48%, list=18%, signal=58% |
| 3 | M\_PHASE |  | 98 | 0.52 | 2.63 | 0.000 | 0.000 | 0.000 | 2360 | tags=51%, list=18%, signal=62% |
| 4 | M\_PHASE\_OF\_MITOTIC\_CELL\_CYCLE |  | 72 | 0.55 | 2.61 | 0.000 | 0.000 | 0.000 | 2360 | tags=54%, list=18%, signal=66% |
| 5 | MITOSIS |  | 70 | 0.55 | 2.59 | 0.000 | 0.000 | 0.000 | 1553 | tags=47%, list=12%, signal=53% |
| 6 | CELL\_CYCLE\_PHASE |  | 152 | 0.46 | 2.50 | 0.000 | 0.000 | 0.000 | 2360 | tags=45%, list=18%, signal=54% |
| 7 | DNA\_REPLICATION |  | 97 | 0.47 | 2.43 | 0.000 | 0.000 | 0.001 | 3207 | tags=49%, list=24%, signal=65% |
| 8 | DNA\_METABOLIC\_PROCESS |  | 240 | 0.41 | 2.40 | 0.000 | 0.000 | 0.001 | 3207 | tags=47%, list=24%, signal=61% |
| 9 | CELL\_CYCLE\_GO\_0007049 |  | 277 | 0.41 | 2.40 | 0.000 | 0.000 | 0.001 | 2360 | tags=40%, list=18%, signal=48% |
| 10 | SISTER\_CHROMATID\_SEGREGATION |  | 16 | 0.73 | 2.36 | 0.000 | 0.000 | 0.001 | 995 | tags=56%, list=8%, signal=61% |
| 11 | MITOTIC\_SISTER\_CHROMATID\_SEGREGATION |  | 15 | 0.75 | 2.35 | 0.000 | 0.000 | 0.001 | 995 | tags=60%, list=8%, signal=65% |
| 12 | CELL\_CYCLE\_CHECKPOINT\_GO\_0000075 |  | 45 | 0.53 | 2.30 | 0.000 | 0.000 | 0.002 | 2020 | tags=53%, list=15%, signal=63% |
| 13 | DNA\_REPAIR |  | 118 | 0.44 | 2.29 | 0.000 | 0.000 | 0.002 | 2966 | tags=48%, list=23%, signal=62% |
| 14 | DNA\_DEPENDENT\_DNA\_REPLICATION |  | 52 | 0.50 | 2.28 | 0.000 | 0.000 | 0.004 | 3207 | tags=56%, list=24%, signal=74% |
| 15 | REGULATION\_OF\_MITOSIS |  | 33 | 0.57 | 2.27 | 0.000 | 0.000 | 0.004 | 1008 | tags=45%, list=8%, signal=49% |
| 16 | CHROMOSOME\_SEGREGATION |  | 28 | 0.58 | 2.24 | 0.000 | 0.000 | 0.006 | 995 | tags=46%, list=8%, signal=50% |
| 17 | RESPONSE\_TO\_DNA\_DAMAGE\_STIMULUS |  | 153 | 0.40 | 2.22 | 0.000 | 0.000 | 0.009 | 2966 | tags=46%, list=23%, signal=58% |
| 18 | RNA\_SPLICING |  | 74 | 0.45 | 2.20 | 0.000 | 0.000 | 0.010 | 3283 | tags=51%, list=25%, signal=68% |
| 19 | RESPONSE\_TO\_ENDOGENOUS\_STIMULUS |  | 182 | 0.39 | 2.17 | 0.000 | 0.001 | 0.016 | 3327 | tags=46%, list=25%, signal=61% |
| 20 | NUCLEOTIDE\_BIOSYNTHETIC\_PROCESS |  | 17 | 0.64 | 2.12 | 0.000 | 0.001 | 0.028 | 1446 | tags=53%, list=11%, signal=59% |
| 21 | RNA\_PROCESSING |  | 138 | 0.40 | 2.11 | 0.000 | 0.001 | 0.032 | 3283 | tags=49%, list=25%, signal=64% |
| 22 | MICROTUBULE\_CYTOSKELETON\_ORGANIZATION\_AND\_BIOGENESIS |  | 31 | 0.53 | 2.08 | 0.000 | 0.002 | 0.051 | 2715 | tags=55%, list=21%, signal=69% |
| 23 | DNA\_REPLICATION\_INITIATION |  | 15 | 0.66 | 2.08 | 0.000 | 0.002 | 0.053 | 2614 | tags=80%, list=20%, signal=100% |
| 24 | DNA\_INTEGRITY\_CHECKPOINT |  | 22 | 0.58 | 2.06 | 0.000 | 0.002 | 0.066 | 2020 | tags=59%, list=15%, signal=70% |
| 25 | PROTEIN\_FOLDING |  | 55 | 0.44 | 2.01 | 0.000 | 0.004 | 0.124 | 3371 | tags=53%, list=26%, signal=71% |
| 26 | MITOCHONDRION\_ORGANIZATION\_AND\_BIOGENESIS |  | 42 | 0.47 | 2.00 | 0.000 | 0.004 | 0.127 | 3258 | tags=50%, list=25%, signal=66% |
| 27 | MITOTIC\_CELL\_CYCLE\_CHECKPOINT |  | 19 | 0.58 | 2.00 | 0.004 | 0.004 | 0.130 | 2020 | tags=53%, list=15%, signal=62% |
| 28 | REGULATION\_OF\_CELL\_CYCLE |  | 161 | 0.36 | 2.00 | 0.000 | 0.004 | 0.136 | 2021 | tags=35%, list=15%, signal=41% |
| 29 | MRNA\_METABOLIC\_PROCESS |  | 72 | 0.41 | 1.98 | 0.000 | 0.005 | 0.163 | 3283 | tags=50%, list=25%, signal=66% |
| 30 | DOUBLE\_STRAND\_BREAK\_REPAIR |  | 21 | 0.56 | 1.97 | 0.004 | 0.005 | 0.174 | 2020 | tags=52%, list=15%, signal=62% |
| 31 | REGULATION\_OF\_MITOTIC\_CELL\_CYCLE |  | 19 | 0.57 | 1.96 | 0.002 | 0.005 | 0.183 | 1549 | tags=47%, list=12%, signal=54% |
| 32 | COENZYME\_METABOLIC\_PROCESS |  | 35 | 0.48 | 1.95 | 0.002 | 0.006 | 0.214 | 3327 | tags=49%, list=25%, signal=65% |
| 33 | TRNA\_METABOLIC\_PROCESS |  | 15 | 0.61 | 1.94 | 0.004 | 0.006 | 0.223 | 2536 | tags=67%, list=19%, signal=83% |
| 34 | INTERPHASE\_OF\_MITOTIC\_CELL\_CYCLE |  | 57 | 0.42 | 1.91 | 0.000 | 0.008 | 0.288 | 3368 | tags=51%, list=26%, signal=68% |
| 35 | NUCLEOBASENUCLEOSIDENUCLEOTIDE\_AND\_NUCLEIC\_ACID\_TRANSPORT |  | 26 | 0.50 | 1.91 | 0.002 | 0.008 | 0.312 | 2284 | tags=46%, list=17%, signal=56% |
| 36 | INTERPHASE |  | 63 | 0.41 | 1.90 | 0.000 | 0.009 | 0.336 | 3368 | tags=49%, list=26%, signal=66% |
| 37 | MRNA\_PROCESSING\_GO\_0006397 |  | 61 | 0.41 | 1.87 | 0.002 | 0.012 | 0.439 | 3283 | tags=48%, list=25%, signal=63% |
| 38 | COFACTOR\_BIOSYNTHETIC\_PROCESS |  | 21 | 0.53 | 1.86 | 0.002 | 0.012 | 0.441 | 1523 | tags=38%, list=12%, signal=43% |
| 39 | REGULATION\_OF\_DNA\_REPLICATION |  | 18 | 0.54 | 1.84 | 0.009 | 0.013 | 0.495 | 2020 | tags=50%, list=15%, signal=59% |
| 40 | TRANSCRIPTION\_INITIATION\_FROM\_RNA\_POLYMERASE\_II\_PROMOTER |  | 27 | 0.49 | 1.84 | 0.006 | 0.014 | 0.519 | 2283 | tags=44%, list=17%, signal=54% |
| 41 | REGULATION\_OF\_CYCLIN\_DEPENDENT\_PROTEIN\_KINASE\_ACTIVITY |  | 40 | 0.44 | 1.84 | 0.005 | 0.014 | 0.521 | 2512 | tags=48%, list=19%, signal=59% |
| 42 | MITOCHONDRIAL\_TRANSPORT |  | 18 | 0.54 | 1.82 | 0.002 | 0.015 | 0.570 | 1498 | tags=44%, list=11%, signal=50% |
| 43 | CHROMOSOME\_ORGANIZATION\_AND\_BIOGENESIS |  | 107 | 0.35 | 1.82 | 0.000 | 0.015 | 0.576 | 2789 | tags=38%, list=21%, signal=48% |
| 44 | REGULATION\_OF\_DNA\_METABOLIC\_PROCESS |  | 40 | 0.43 | 1.82 | 0.002 | 0.015 | 0.576 | 2151 | tags=45%, list=16%, signal=54% |
| 45 | COFACTOR\_METABOLIC\_PROCESS |  | 51 | 0.40 | 1.79 | 0.002 | 0.018 | 0.647 | 3373 | tags=43%, list=26%, signal=58% |
| 46 | G1\_S\_TRANSITION\_OF\_MITOTIC\_CELL\_CYCLE |  | 23 | 0.48 | 1.78 | 0.000 | 0.019 | 0.673 | 3243 | tags=52%, list=25%, signal=69% |
| 47 | DNA\_DAMAGE\_CHECKPOINT |  | 19 | 0.52 | 1.77 | 0.004 | 0.020 | 0.694 | 2020 | tags=53%, list=15%, signal=62% |
| 48 | NUCLEAR\_EXPORT |  | 26 | 0.47 | 1.73 | 0.007 | 0.028 | 0.818 | 4298 | tags=58%, list=33%, signal=86% |
| 49 | PROTEIN\_MODIFICATION\_BY\_SMALL\_PROTEIN\_CONJUGATION |  | 35 | 0.42 | 1.71 | 0.007 | 0.033 | 0.879 | 2562 | tags=40%, list=20%, signal=50% |
| 50 | DNA\_DAMAGE\_RESPONSESIGNAL\_TRANSDUCTION |  | 34 | 0.43 | 1.71 | 0.004 | 0.033 | 0.880 | 2020 | tags=44%, list=15%, signal=52% |
| 51 | RNA\_EXPORT\_FROM\_NUCLEUS |  | 17 | 0.51 | 1.70 | 0.020 | 0.033 | 0.886 | 4642 | tags=71%, list=35%, signal=109% |
| 52 | BIOPOLYMER\_CATABOLIC\_PROCESS |  | 103 | 0.33 | 1.68 | 0.000 | 0.039 | 0.931 | 3537 | tags=42%, list=27%, signal=57% |
| 53 | UBIQUITIN\_CYCLE |  | 40 | 0.40 | 1.67 | 0.011 | 0.041 | 0.944 | 2562 | tags=38%, list=20%, signal=46% |
| 54 | CYTOKINESIS |  | 17 | 0.50 | 1.66 | 0.023 | 0.044 | 0.957 | 986 | tags=35%, list=8%, signal=38% |
| 55 | PROTEIN\_CATABOLIC\_PROCESS |  | 60 | 0.36 | 1.65 | 0.009 | 0.044 | 0.959 | 2186 | tags=32%, list=17%, signal=38% |
| 56 | PROTEIN\_UBIQUITINATION |  | 32 | 0.41 | 1.63 | 0.014 | 0.050 | 0.975 | 2562 | tags=38%, list=20%, signal=47% |
| 57 | CELLULAR\_PROTEIN\_CATABOLIC\_PROCESS |  | 50 | 0.37 | 1.63 | 0.018 | 0.052 | 0.981 | 3528 | tags=42%, list=27%, signal=57% |
| 58 | PROTEIN\_DNA\_COMPLEX\_ASSEMBLY |  | 45 | 0.38 | 1.63 | 0.010 | 0.052 | 0.982 | 2962 | tags=42%, list=23%, signal=54% |
| 59 | BASE\_EXCISION\_REPAIR |  | 16 | 0.48 | 1.61 | 0.023 | 0.058 | 0.988 | 2918 | tags=44%, list=22%, signal=56% |
| 60 | TRANSCRIPTION\_INITIATION |  | 33 | 0.41 | 1.60 | 0.027 | 0.059 | 0.991 | 2283 | tags=39%, list=17%, signal=48% |
| 61 | MACROMOLECULE\_CATABOLIC\_PROCESS |  | 120 | 0.30 | 1.60 | 0.005 | 0.059 | 0.991 | 3537 | tags=38%, list=27%, signal=52% |
| 62 | NUCLEAR\_TRANSPORT |  | 77 | 0.32 | 1.56 | 0.007 | 0.078 | 0.999 | 4169 | tags=45%, list=32%, signal=66% |
| 63 | CELL\_DIVISION |  | 19 | 0.46 | 1.54 | 0.042 | 0.087 | 0.999 | 986 | tags=32%, list=8%, signal=34% |
| 64 | MEIOTIC\_CELL\_CYCLE |  | 31 | 0.40 | 1.54 | 0.024 | 0.086 | 0.999 | 3401 | tags=48%, list=26%, signal=65% |
| 65 | NUCLEOCYTOPLASMIC\_TRANSPORT |  | 77 | 0.32 | 1.54 | 0.016 | 0.086 | 0.999 | 4169 | tags=45%, list=32%, signal=66% |
| 66 | NUCLEOTIDE\_METABOLIC\_PROCESS |  | 36 | 0.38 | 1.53 | 0.036 | 0.090 | 1.000 | 2494 | tags=39%, list=19%, signal=48% |
| 67 | DNA\_RECOMBINATION |  | 45 | 0.36 | 1.53 | 0.029 | 0.090 | 1.000 | 2151 | tags=36%, list=16%, signal=42% |
| 68 | DNA\_PACKAGING |  | 29 | 0.40 | 1.52 | 0.035 | 0.090 | 1.000 | 3021 | tags=45%, list=23%, signal=58% |
| 69 | ONE\_CARBON\_COMPOUND\_METABOLIC\_PROCESS |  | 24 | 0.42 | 1.51 | 0.037 | 0.095 | 1.000 | 3874 | tags=58%, list=30%, signal=83% |
| 70 | CELLULAR\_MACROMOLECULE\_CATABOLIC\_PROCESS |  | 90 | 0.30 | 1.51 | 0.022 | 0.099 | 1.000 | 2284 | tags=29%, list=17%, signal=35% |
| 71 | MICROTUBULE\_BASED\_PROCESS |  | 75 | 0.32 | 1.50 | 0.014 | 0.100 | 1.000 | 2715 | tags=35%, list=21%, signal=43% |
| 72 | ORGANELLE\_ORGANIZATION\_AND\_BIOGENESIS |  | 407 | 0.24 | 1.49 | 0.000 | 0.105 | 1.000 | 3191 | tags=32%, list=24%, signal=42% |
| 73 | NUCLEOBASENUCLEOSIDE\_AND\_NUCLEOTIDE\_METABOLIC\_PROCESS |  | 46 | 0.34 | 1.47 | 0.025 | 0.122 | 1.000 | 1446 | tags=28%, list=11%, signal=32% |
| 74 | APOPTOTIC\_NUCLEAR\_CHANGES |  | 17 | 0.44 | 1.46 | 0.070 | 0.124 | 1.000 | 1413 | tags=35%, list=11%, signal=40% |
| 75 | ESTABLISHMENT\_OF\_ORGANELLE\_LOCALIZATION |  | 16 | 0.45 | 1.45 | 0.064 | 0.131 | 1.000 | 895 | tags=38%, list=7%, signal=40% |
| 76 | CELLULAR\_COMPONENT\_DISASSEMBLY |  | 31 | 0.37 | 1.45 | 0.053 | 0.131 | 1.000 | 3161 | tags=42%, list=24%, signal=55% |
| 77 | VIRAL\_INFECTIOUS\_CYCLE |  | 29 | 0.37 | 1.43 | 0.065 | 0.151 | 1.000 | 1628 | tags=34%, list=12%, signal=39% |
| 78 | MEIOSIS\_I |  | 19 | 0.42 | 1.43 | 0.056 | 0.149 | 1.000 | 1947 | tags=37%, list=15%, signal=43% |
| 79 | ORGANELLE\_LOCALIZATION |  | 21 | 0.40 | 1.42 | 0.086 | 0.152 | 1.000 | 895 | tags=29%, list=7%, signal=31% |
| 80 | NEGATIVE\_REGULATION\_OF\_DNA\_METABOLIC\_PROCESS |  | 16 | 0.42 | 1.39 | 0.096 | 0.186 | 1.000 | 2578 | tags=50%, list=20%, signal=62% |
| 81 | VIRAL\_REPRODUCTIVE\_PROCESS |  | 33 | 0.35 | 1.38 | 0.095 | 0.193 | 1.000 | 1628 | tags=33%, list=12%, signal=38% |
| 82 | CHROMATIN\_ASSEMBLY\_OR\_DISASSEMBLY |  | 25 | 0.37 | 1.37 | 0.091 | 0.198 | 1.000 | 3021 | tags=48%, list=23%, signal=62% |
| 83 | ALCOHOL\_METABOLIC\_PROCESS |  | 82 | 0.27 | 1.34 | 0.071 | 0.240 | 1.000 | 3828 | tags=38%, list=29%, signal=53% |
| 84 | VIRAL\_REPRODUCTION |  | 38 | 0.32 | 1.33 | 0.102 | 0.255 | 1.000 | 1857 | tags=32%, list=14%, signal=37% |
| 85 | RESPONSE\_TO\_HYPOXIA |  | 27 | 0.35 | 1.32 | 0.131 | 0.256 | 1.000 | 2450 | tags=33%, list=19%, signal=41% |
| 86 | VIRAL\_GENOME\_REPLICATION |  | 20 | 0.38 | 1.32 | 0.102 | 0.257 | 1.000 | 1628 | tags=35%, list=12%, signal=40% |
| 87 | INTRACELLULAR\_TRANSPORT |  | 248 | 0.22 | 1.29 | 0.037 | 0.299 | 1.000 | 3721 | tags=35%, list=28%, signal=48% |
| 88 | ESTABLISHMENT\_AND\_OR\_MAINTENANCE\_OF\_CHROMATIN\_ARCHITECTURE |  | 65 | 0.28 | 1.28 | 0.119 | 0.309 | 1.000 | 2789 | tags=34%, list=21%, signal=43% |
| 89 | CHROMATIN\_REMODELING |  | 21 | 0.36 | 1.28 | 0.162 | 0.318 | 1.000 | 2362 | tags=38%, list=18%, signal=46% |
| 90 | NEGATIVE\_REGULATION\_OF\_CATALYTIC\_ACTIVITY |  | 61 | 0.28 | 1.27 | 0.119 | 0.322 | 1.000 | 3247 | tags=39%, list=25%, signal=52% |
| 91 | NEGATIVE\_REGULATION\_OF\_BINDING |  | 16 | 0.39 | 1.27 | 0.178 | 0.322 | 1.000 | 2302 | tags=44%, list=18%, signal=53% |
| 92 | ESTABLISHMENT\_OF\_CELLULAR\_LOCALIZATION |  | 311 | 0.21 | 1.27 | 0.053 | 0.326 | 1.000 | 3721 | tags=34%, list=28%, signal=46% |
| 93 | APOPTOTIC\_PROGRAM |  | 56 | 0.28 | 1.27 | 0.125 | 0.324 | 1.000 | 3414 | tags=39%, list=26%, signal=53% |
| 94 | G1\_PHASE |  | 15 | 0.39 | 1.25 | 0.191 | 0.343 | 1.000 | 3129 | tags=47%, list=24%, signal=61% |
| 95 | RESPONSE\_TO\_ORGANIC\_SUBSTANCE |  | 27 | 0.33 | 1.25 | 0.186 | 0.343 | 1.000 | 3008 | tags=37%, list=23%, signal=48% |
| 96 | HETEROCYCLE\_METABOLIC\_PROCESS |  | 26 | 0.34 | 1.25 | 0.170 | 0.343 | 1.000 | 1498 | tags=23%, list=11%, signal=26% |
| 97 | RNA\_CATABOLIC\_PROCESS |  | 20 | 0.36 | 1.24 | 0.198 | 0.355 | 1.000 | 3274 | tags=50%, list=25%, signal=67% |
| 98 | RESPONSE\_TO\_STRESS |  | 467 | 0.20 | 1.24 | 0.037 | 0.357 | 1.000 | 3008 | tags=29%, list=23%, signal=36% |
| 99 | CELLULAR\_LOCALIZATION |  | 323 | 0.20 | 1.23 | 0.064 | 0.361 | 1.000 | 3721 | tags=33%, list=28%, signal=45% |
| 100 | CYTOSKELETON\_DEPENDENT\_INTRACELLULAR\_TRANSPORT |  | 25 | 0.34 | 1.23 | 0.187 | 0.361 | 1.000 | 4251 | tags=52%, list=32%, signal=77% |
| 101 | RESPONSE\_TO\_ABIOTIC\_STIMULUS |  | 79 | 0.25 | 1.23 | 0.120 | 0.359 | 1.000 | 3393 | tags=37%, list=26%, signal=49% |
| 102 | RESPONSE\_TO\_HORMONE\_STIMULUS |  | 26 | 0.32 | 1.21 | 0.208 | 0.389 | 1.000 | 4912 | tags=58%, list=38%, signal=92% |
| 103 | RIBONUCLEOPROTEIN\_COMPLEX\_BIOGENESIS\_AND\_ASSEMBLY |  | 68 | 0.26 | 1.21 | 0.172 | 0.396 | 1.000 | 3350 | tags=37%, list=26%, signal=49% |
| 104 | DNA\_CATABOLIC\_PROCESS |  | 21 | 0.35 | 1.19 | 0.230 | 0.433 | 1.000 | 3537 | tags=43%, list=27%, signal=59% |
| 105 | TRANSCRIPTION\_FROM\_RNA\_POLYMERASE\_III\_PROMOTER |  | 18 | 0.36 | 1.18 | 0.252 | 0.444 | 1.000 | 3617 | tags=56%, list=28%, signal=77% |
| 106 | INDUCTION\_OF\_APOPTOSIS\_BY\_EXTRACELLULAR\_SIGNALS |  | 25 | 0.32 | 1.17 | 0.267 | 0.461 | 1.000 | 2587 | tags=36%, list=20%, signal=45% |
| 107 | CELLULAR\_RESPIRATION |  | 19 | 0.34 | 1.16 | 0.258 | 0.479 | 1.000 | 2693 | tags=37%, list=21%, signal=46% |
| 108 | LIPID\_BIOSYNTHETIC\_PROCESS |  | 84 | 0.24 | 1.16 | 0.221 | 0.475 | 1.000 | 1598 | tags=21%, list=12%, signal=24% |
| 109 | REGULATION\_OF\_HYDROLASE\_ACTIVITY |  | 65 | 0.25 | 1.16 | 0.221 | 0.473 | 1.000 | 3247 | tags=34%, list=25%, signal=45% |
| 110 | TRANSCRIPTION\_FROM\_RNA\_POLYMERASE\_II\_PROMOTER |  | 428 | 0.18 | 1.14 | 0.123 | 0.513 | 1.000 | 2763 | tags=25%, list=21%, signal=30% |
| 111 | NITROGEN\_COMPOUND\_BIOSYNTHETIC\_PROCESS |  | 25 | 0.30 | 1.14 | 0.277 | 0.510 | 1.000 | 1540 | tags=24%, list=12%, signal=27% |
| 112 | CELL\_STRUCTURE\_DISASSEMBLY\_DURING\_APOPTOSIS |  | 17 | 0.34 | 1.14 | 0.306 | 0.522 | 1.000 | 1197 | tags=24%, list=9%, signal=26% |
| 113 | CATABOLIC\_PROCESS |  | 201 | 0.20 | 1.13 | 0.191 | 0.523 | 1.000 | 3580 | tags=32%, list=27%, signal=43% |
| 114 | CELLULAR\_CATABOLIC\_PROCESS |  | 189 | 0.20 | 1.13 | 0.195 | 0.528 | 1.000 | 3580 | tags=32%, list=27%, signal=43% |
| 115 | REGULATION\_OF\_KINASE\_ACTIVITY |  | 135 | 0.21 | 1.13 | 0.238 | 0.532 | 1.000 | 2283 | tags=24%, list=17%, signal=28% |
| 116 | GLUTAMATE\_SIGNALING\_PATHWAY |  | 17 | 0.34 | 1.12 | 0.294 | 0.537 | 1.000 | 4173 | tags=41%, list=32%, signal=60% |
| 117 | CHROMATIN\_MODIFICATION |  | 46 | 0.26 | 1.11 | 0.285 | 0.555 | 1.000 | 2362 | tags=28%, list=18%, signal=34% |
| 118 | REGULATION\_OF\_TRANSFERASE\_ACTIVITY |  | 137 | 0.20 | 1.11 | 0.259 | 0.557 | 1.000 | 2283 | tags=23%, list=17%, signal=28% |
| 119 | REGULATION\_OF\_GENE\_EXPRESSION\_EPIGENETIC |  | 27 | 0.29 | 1.11 | 0.287 | 0.558 | 1.000 | 3750 | tags=44%, list=29%, signal=62% |
| 120 | MEIOTIC\_RECOMBINATION |  | 16 | 0.35 | 1.11 | 0.295 | 0.554 | 1.000 | 3401 | tags=44%, list=26%, signal=59% |
| 121 | NEGATIVE\_REGULATION\_OF\_DNA\_BINDING |  | 15 | 0.34 | 1.10 | 0.344 | 0.582 | 1.000 | 2302 | tags=40%, list=18%, signal=48% |
| 122 | REGULATION\_OF\_CATALYTIC\_ACTIVITY |  | 238 | 0.19 | 1.09 | 0.230 | 0.586 | 1.000 | 3190 | tags=28%, list=24%, signal=37% |
| 123 | REGULATION\_OF\_PROTEIN\_KINASE\_ACTIVITY |  | 133 | 0.20 | 1.09 | 0.285 | 0.583 | 1.000 | 2283 | tags=23%, list=17%, signal=28% |
| 124 | CELLULAR\_RESPONSE\_TO\_STIMULUS |  | 17 | 0.32 | 1.09 | 0.331 | 0.580 | 1.000 | 3008 | tags=35%, list=23%, signal=46% |
| 125 | CYTOSKELETON\_ORGANIZATION\_AND\_BIOGENESIS |  | 182 | 0.19 | 1.09 | 0.274 | 0.584 | 1.000 | 2735 | tags=26%, list=21%, signal=33% |
| 126 | NUCLEAR\_ORGANIZATION\_AND\_BIOGENESIS |  | 23 | 0.30 | 1.09 | 0.340 | 0.585 | 1.000 | 1413 | tags=26%, list=11%, signal=29% |
| 127 | NEURON\_APOPTOSIS |  | 15 | 0.33 | 1.08 | 0.333 | 0.593 | 1.000 | 1488 | tags=27%, list=11%, signal=30% |
| 128 | NUCLEAR\_IMPORT |  | 47 | 0.25 | 1.08 | 0.303 | 0.599 | 1.000 | 4112 | tags=40%, list=31%, signal=59% |
| 129 | CELLULAR\_BIOSYNTHETIC\_PROCESS |  | 273 | 0.18 | 1.07 | 0.290 | 0.614 | 1.000 | 1647 | tags=18%, list=13%, signal=21% |
| 130 | CHROMATIN\_ASSEMBLY |  | 16 | 0.33 | 1.07 | 0.358 | 0.616 | 1.000 | 3021 | tags=44%, list=23%, signal=57% |
| 131 | NEGATIVE\_REGULATION\_OF\_APOPTOSIS |  | 136 | 0.20 | 1.06 | 0.326 | 0.618 | 1.000 | 1628 | tags=20%, list=12%, signal=22% |
| 132 | MICROTUBULE\_BASED\_MOVEMENT |  | 16 | 0.33 | 1.06 | 0.385 | 0.624 | 1.000 | 2619 | tags=38%, list=20%, signal=47% |
| 133 | GAMETE\_GENERATION |  | 92 | 0.21 | 1.06 | 0.329 | 0.625 | 1.000 | 3778 | tags=35%, list=29%, signal=49% |
| 134 | OXYGEN\_AND\_REACTIVE\_OXYGEN\_SPECIES\_METABOLIC\_PROCESS |  | 18 | 0.31 | 1.05 | 0.386 | 0.635 | 1.000 | 2925 | tags=39%, list=22%, signal=50% |
| 135 | NEGATIVE\_REGULATION\_OF\_TRANSPORT |  | 18 | 0.31 | 1.05 | 0.376 | 0.635 | 1.000 | 3668 | tags=44%, list=28%, signal=62% |
| 136 | RNA\_SPLICINGVIA\_TRANSESTERIFICATION\_REACTIONS |  | 27 | 0.28 | 1.05 | 0.367 | 0.635 | 1.000 | 2605 | tags=30%, list=20%, signal=37% |
| 137 | NEGATIVE\_REGULATION\_OF\_PROGRAMMED\_CELL\_DEATH |  | 137 | 0.20 | 1.05 | 0.380 | 0.638 | 1.000 | 1628 | tags=20%, list=12%, signal=22% |
| 138 | REGULATION\_OF\_PROTEIN\_STABILITY |  | 17 | 0.32 | 1.04 | 0.391 | 0.643 | 1.000 | 4054 | tags=41%, list=31%, signal=60% |
| 139 | REGULATION\_OF\_MOLECULAR\_FUNCTION |  | 275 | 0.17 | 1.03 | 0.359 | 0.668 | 1.000 | 2314 | tags=21%, list=18%, signal=26% |
| 140 | MORPHOGENESIS\_OF\_AN\_EPITHELIUM |  | 15 | 0.32 | 1.03 | 0.427 | 0.676 | 1.000 | 3656 | tags=47%, list=28%, signal=65% |
| 141 | PIGMENT\_BIOSYNTHETIC\_PROCESS |  | 17 | 0.31 | 1.03 | 0.425 | 0.673 | 1.000 | 1498 | tags=24%, list=11%, signal=27% |
| 142 | CARBOHYDRATE\_TRANSPORT |  | 17 | 0.31 | 1.03 | 0.425 | 0.674 | 1.000 | 2337 | tags=29%, list=18%, signal=36% |
| 143 | INTERACTION\_WITH\_HOST |  | 15 | 0.32 | 1.02 | 0.443 | 0.691 | 1.000 | 1467 | tags=27%, list=11%, signal=30% |
| 144 | STEROID\_HORMONE\_RECEPTOR\_SIGNALING\_PATHWAY |  | 18 | 0.30 | 1.01 | 0.436 | 0.701 | 1.000 | 1118 | tags=22%, list=9%, signal=24% |
| 145 | NEGATIVE\_REGULATION\_OF\_TRANSFERASE\_ACTIVITY |  | 27 | 0.27 | 1.01 | 0.466 | 0.707 | 1.000 | 3190 | tags=37%, list=24%, signal=49% |
| 146 | REGULATION\_OF\_TRANSCRIPTION\_FROM\_RNA\_POLYMERASE\_II\_PROMOTER |  | 267 | 0.17 | 1.01 | 0.432 | 0.709 | 1.000 | 2323 | tags=22%, list=18%, signal=26% |
| 147 | DNA\_DAMAGE\_RESPONSESIGNAL\_TRANSDUCTION\_RESULTING\_IN\_INDUCTION\_OF\_APOPTOSIS |  | 15 | 0.31 | 1.00 | 0.431 | 0.708 | 1.000 | 1234 | tags=27%, list=9%, signal=29% |
| 148 | INTRACELLULAR\_RECEPTOR\_MEDIATED\_SIGNALING\_PATHWAY |  | 18 | 0.30 | 1.00 | 0.461 | 0.710 | 1.000 | 1118 | tags=22%, list=9%, signal=24% |
| 149 | PROTEIN\_IMPORT |  | 58 | 0.22 | 1.00 | 0.451 | 0.712 | 1.000 | 4112 | tags=36%, list=31%, signal=53% |
| 150 | GENERATION\_OF\_A\_SIGNAL\_INVOLVED\_IN\_CELL\_CELL\_SIGNALING |  | 25 | 0.27 | 0.99 | 0.474 | 0.726 | 1.000 | 2285 | tags=28%, list=17%, signal=34% |
| 151 | CELL\_PROJECTION\_BIOGENESIS |  | 20 | 0.29 | 0.99 | 0.447 | 0.724 | 1.000 | 4565 | tags=50%, list=35%, signal=77% |
| 152 | ENERGY\_DERIVATION\_BY\_OXIDATION\_OF\_ORGANIC\_COMPOUNDS |  | 37 | 0.24 | 0.99 | 0.472 | 0.724 | 1.000 | 2148 | tags=24%, list=16%, signal=29% |
| 153 | SECONDARY\_METABOLIC\_PROCESS |  | 23 | 0.27 | 0.99 | 0.482 | 0.731 | 1.000 | 1498 | tags=22%, list=11%, signal=25% |
| 154 | MACROMOLECULE\_LOCALIZATION |  | 202 | 0.17 | 0.98 | 0.498 | 0.732 | 1.000 | 3934 | tags=33%, list=30%, signal=46% |
| 155 | STEROID\_METABOLIC\_PROCESS |  | 66 | 0.21 | 0.97 | 0.518 | 0.753 | 1.000 | 4207 | tags=41%, list=32%, signal=60% |
| 156 | PROGRAMMED\_CELL\_DEATH |  | 393 | 0.16 | 0.97 | 0.536 | 0.749 | 1.000 | 1512 | tags=16%, list=12%, signal=17% |
| 157 | RESPONSE\_TO\_TEMPERATURE\_STIMULUS |  | 16 | 0.30 | 0.97 | 0.508 | 0.752 | 1.000 | 3393 | tags=44%, list=26%, signal=59% |
| 158 | PROTEIN\_AMINO\_ACID\_O\_LINKED\_GLYCOSYLATION |  | 18 | 0.28 | 0.97 | 0.494 | 0.747 | 1.000 | 2902 | tags=39%, list=22%, signal=50% |
| 159 | STEROID\_BIOSYNTHETIC\_PROCESS |  | 22 | 0.27 | 0.97 | 0.506 | 0.744 | 1.000 | 1598 | tags=27%, list=12%, signal=31% |
| 160 | APOPTOSIS\_GO |  | 392 | 0.16 | 0.97 | 0.541 | 0.742 | 1.000 | 1512 | tags=16%, list=12%, signal=17% |
| 161 | PIGMENT\_METABOLIC\_PROCESS |  | 18 | 0.29 | 0.97 | 0.501 | 0.742 | 1.000 | 1498 | tags=22%, list=11%, signal=25% |
| 162 | INTRACELLULAR\_PROTEIN\_TRANSPORT |  | 127 | 0.18 | 0.96 | 0.560 | 0.754 | 1.000 | 3682 | tags=31%, list=28%, signal=42% |
| 163 | REGULATION\_OF\_PROGRAMMED\_CELL\_DEATH |  | 313 | 0.16 | 0.95 | 0.596 | 0.768 | 1.000 | 1497 | tags=16%, list=11%, signal=18% |
| 164 | PROTEIN\_TARGETING |  | 94 | 0.19 | 0.95 | 0.550 | 0.781 | 1.000 | 4169 | tags=35%, list=32%, signal=51% |
| 165 | BIOSYNTHETIC\_PROCESS |  | 402 | 0.15 | 0.94 | 0.645 | 0.787 | 1.000 | 1647 | tags=16%, list=13%, signal=18% |
| 166 | PROTEIN\_TRANSPORT |  | 139 | 0.18 | 0.94 | 0.605 | 0.793 | 1.000 | 3798 | tags=32%, list=29%, signal=44% |
| 167 | REGULATION\_OF\_APOPTOSIS |  | 312 | 0.16 | 0.94 | 0.626 | 0.788 | 1.000 | 1497 | tags=16%, list=11%, signal=18% |
| 168 | INDUCTION\_OF\_APOPTOSIS\_BY\_INTRACELLULAR\_SIGNALS |  | 23 | 0.25 | 0.93 | 0.566 | 0.811 | 1.000 | 3243 | tags=35%, list=25%, signal=46% |
| 169 | COVALENT\_CHROMATIN\_MODIFICATION |  | 22 | 0.26 | 0.93 | 0.561 | 0.814 | 1.000 | 3716 | tags=41%, list=28%, signal=57% |
| 170 | NEGATIVE\_REGULATION\_OF\_CELL\_ADHESION |  | 16 | 0.29 | 0.93 | 0.550 | 0.810 | 1.000 | 3359 | tags=44%, list=26%, signal=59% |
| 171 | DIGESTION |  | 42 | 0.22 | 0.92 | 0.569 | 0.829 | 1.000 | 3068 | tags=24%, list=23%, signal=31% |
| 172 | PROTEIN\_RNA\_COMPLEX\_ASSEMBLY |  | 55 | 0.20 | 0.92 | 0.599 | 0.827 | 1.000 | 3709 | tags=36%, list=28%, signal=51% |
| 173 | PROTEIN\_IMPORT\_INTO\_NUCLEUS |  | 45 | 0.21 | 0.91 | 0.617 | 0.841 | 1.000 | 4112 | tags=38%, list=31%, signal=55% |
| 174 | REGULATION\_OF\_TRANSPORT |  | 57 | 0.20 | 0.90 | 0.613 | 0.858 | 1.000 | 4121 | tags=40%, list=31%, signal=59% |
| 175 | REGULATION\_OF\_NEUROTRANSMITTER\_LEVELS |  | 23 | 0.25 | 0.89 | 0.595 | 0.871 | 1.000 | 1204 | tags=17%, list=9%, signal=19% |
| 176 | POSITIVE\_REGULATION\_OF\_CELL\_CYCLE |  | 15 | 0.28 | 0.89 | 0.615 | 0.871 | 1.000 | 1524 | tags=27%, list=12%, signal=30% |
| 177 | EMBRYONIC\_DEVELOPMENT |  | 46 | 0.20 | 0.89 | 0.629 | 0.873 | 1.000 | 2983 | tags=26%, list=23%, signal=34% |
| 178 | SEXUAL\_REPRODUCTION |  | 109 | 0.17 | 0.89 | 0.677 | 0.875 | 1.000 | 3778 | tags=31%, list=29%, signal=43% |
| 179 | REGULATION\_OF\_NUCLEOCYTOPLASMIC\_TRANSPORT |  | 19 | 0.26 | 0.88 | 0.644 | 0.875 | 1.000 | 1244 | tags=21%, list=10%, signal=23% |
| 180 | AROMATIC\_COMPOUND\_METABOLIC\_PROCESS |  | 26 | 0.23 | 0.88 | 0.641 | 0.877 | 1.000 | 446 | tags=15%, list=3%, signal=16% |
| 181 | REGULATION\_OF\_RNA\_METABOLIC\_PROCESS |  | 417 | 0.14 | 0.88 | 0.833 | 0.878 | 1.000 | 2339 | tags=20%, list=18%, signal=23% |
| 182 | TRANSMISSION\_OF\_NERVE\_IMPULSE |  | 167 | 0.16 | 0.88 | 0.727 | 0.875 | 1.000 | 2493 | tags=20%, list=19%, signal=25% |
| 183 | RESPONSE\_TO\_OXIDATIVE\_STRESS |  | 38 | 0.21 | 0.88 | 0.655 | 0.872 | 1.000 | 3362 | tags=39%, list=26%, signal=53% |
| 184 | ENERGY\_RESERVE\_METABOLIC\_PROCESS |  | 15 | 0.27 | 0.87 | 0.637 | 0.882 | 1.000 | 1251 | tags=20%, list=10%, signal=22% |
| 185 | PHOSPHOINOSITIDE\_BIOSYNTHETIC\_PROCESS |  | 21 | 0.25 | 0.86 | 0.651 | 0.896 | 1.000 | 983 | tags=19%, list=8%, signal=21% |
| 186 | SPLICEOSOME\_ASSEMBLY |  | 17 | 0.26 | 0.85 | 0.649 | 0.908 | 1.000 | 3283 | tags=35%, list=25%, signal=47% |
| 187 | SYNAPTIC\_TRANSMISSION |  | 154 | 0.15 | 0.85 | 0.780 | 0.903 | 1.000 | 2461 | tags=19%, list=19%, signal=24% |
| 188 | RESPONSE\_TO\_RADIATION |  | 52 | 0.19 | 0.85 | 0.711 | 0.911 | 1.000 | 3207 | tags=31%, list=24%, signal=41% |
| 189 | CALCIUM\_INDEPENDENT\_CELL\_CELL\_ADHESION |  | 16 | 0.26 | 0.84 | 0.665 | 0.921 | 1.000 | 3495 | tags=38%, list=27%, signal=51% |
| 190 | NITROGEN\_COMPOUND\_METABOLIC\_PROCESS |  | 141 | 0.16 | 0.84 | 0.808 | 0.920 | 1.000 | 1722 | tags=16%, list=13%, signal=19% |
| 191 | MEMBRANE\_LIPID\_BIOSYNTHETIC\_PROCESS |  | 41 | 0.20 | 0.84 | 0.739 | 0.916 | 1.000 | 1540 | tags=17%, list=12%, signal=19% |
| 192 | PROTEIN\_HOMOOLIGOMERIZATION |  | 19 | 0.24 | 0.84 | 0.687 | 0.912 | 1.000 | 757 | tags=16%, list=6%, signal=17% |
| 193 | PROTEOLYSIS |  | 170 | 0.15 | 0.84 | 0.828 | 0.915 | 1.000 | 3816 | tags=32%, list=29%, signal=44% |
| 194 | MEMBRANE\_FUSION |  | 27 | 0.22 | 0.83 | 0.718 | 0.924 | 1.000 | 3528 | tags=37%, list=27%, signal=51% |
| 195 | DEVELOPMENT\_OF\_PRIMARY\_SEXUAL\_CHARACTERISTICS |  | 25 | 0.22 | 0.83 | 0.722 | 0.920 | 1.000 | 2983 | tags=28%, list=23%, signal=36% |
| 196 | REGULATION\_OF\_INTRACELLULAR\_TRANSPORT |  | 22 | 0.23 | 0.83 | 0.725 | 0.920 | 1.000 | 4298 | tags=45%, list=33%, signal=68% |
| 197 | REGULATION\_OF\_SECRETION |  | 35 | 0.20 | 0.81 | 0.773 | 0.951 | 1.000 | 2326 | tags=26%, list=18%, signal=31% |
| 198 | ECTODERM\_DEVELOPMENT |  | 75 | 0.17 | 0.81 | 0.836 | 0.947 | 1.000 | 2450 | tags=24%, list=19%, signal=29% |
| 199 | ESTABLISHMENT\_OF\_PROTEIN\_LOCALIZATION |  | 166 | 0.14 | 0.80 | 0.911 | 0.951 | 1.000 | 4192 | tags=33%, list=32%, signal=48% |
| 200 | TRANSLATIONAL\_INITIATION |  | 33 | 0.20 | 0.79 | 0.793 | 0.961 | 1.000 | 3576 | tags=36%, list=27%, signal=50% |
| 201 | PHOSPHOLIPID\_BIOSYNTHETIC\_PROCESS |  | 35 | 0.20 | 0.79 | 0.801 | 0.959 | 1.000 | 1540 | tags=17%, list=12%, signal=19% |
| 202 | CASPASE\_ACTIVATION |  | 24 | 0.21 | 0.79 | 0.778 | 0.964 | 1.000 | 3161 | tags=33%, list=24%, signal=44% |
| 203 | NEGATIVE\_REGULATION\_OF\_CELL\_CYCLE |  | 72 | 0.16 | 0.78 | 0.852 | 0.961 | 1.000 | 2021 | tags=19%, list=15%, signal=23% |
| 204 | REPRODUCTION |  | 215 | 0.14 | 0.78 | 0.964 | 0.958 | 1.000 | 3778 | tags=30%, list=29%, signal=41% |
| 205 | LIPID\_TRANSPORT |  | 27 | 0.21 | 0.78 | 0.801 | 0.954 | 1.000 | 1915 | tags=22%, list=15%, signal=26% |
| 206 | HISTONE\_MODIFICATION |  | 21 | 0.22 | 0.78 | 0.749 | 0.953 | 1.000 | 3716 | tags=38%, list=28%, signal=53% |
| 207 | NEGATIVE\_REGULATION\_OF\_CELLULAR\_BIOSYNTHETIC\_PROCESS |  | 25 | 0.21 | 0.78 | 0.796 | 0.951 | 1.000 | 3176 | tags=28%, list=24%, signal=37% |
| 208 | CELLULAR\_CARBOHYDRATE\_METABOLIC\_PROCESS |  | 106 | 0.15 | 0.78 | 0.892 | 0.950 | 1.000 | 2099 | tags=17%, list=16%, signal=20% |
| 209 | RESPONSE\_TO\_UV |  | 22 | 0.22 | 0.78 | 0.780 | 0.947 | 1.000 | 3207 | tags=36%, list=24%, signal=48% |
| 210 | GLYCEROPHOSPHOLIPID\_BIOSYNTHETIC\_PROCESS |  | 27 | 0.20 | 0.78 | 0.788 | 0.942 | 1.000 | 983 | tags=15%, list=8%, signal=16% |
| 211 | EXOCYTOSIS |  | 22 | 0.21 | 0.77 | 0.796 | 0.949 | 1.000 | 10290 | tags=100%, list=79%, signal=467% |
| 212 | SENSORY\_PERCEPTION |  | 163 | 0.14 | 0.76 | 0.947 | 0.949 | 1.000 | 5039 | tags=42%, list=38%, signal=67% |
| 213 | NEGATIVE\_REGULATION\_OF\_BIOSYNTHETIC\_PROCESS |  | 26 | 0.20 | 0.74 | 0.822 | 0.970 | 1.000 | 3176 | tags=27%, list=24%, signal=35% |
| 214 | HOMEOSTASIS\_OF\_NUMBER\_OF\_CELLS |  | 20 | 0.21 | 0.74 | 0.828 | 0.970 | 1.000 | 2286 | tags=25%, list=17%, signal=30% |
| 215 | LIPOPROTEIN\_METABOLIC\_PROCESS |  | 30 | 0.19 | 0.73 | 0.867 | 0.977 | 1.000 | 1915 | tags=20%, list=15%, signal=23% |
| 216 | REGULATION\_OF\_CELL\_ADHESION |  | 31 | 0.19 | 0.73 | 0.868 | 0.975 | 1.000 | 4210 | tags=42%, list=32%, signal=62% |
| 217 | NEUROLOGICAL\_SYSTEM\_PROCESS |  | 328 | 0.12 | 0.73 | 0.997 | 0.971 | 1.000 | 5077 | tags=40%, list=39%, signal=64% |
| 218 | EPIDERMAL\_GROWTH\_FACTOR\_RECEPTOR\_SIGNALING\_PATHWAY |  | 18 | 0.22 | 0.72 | 0.821 | 0.969 | 1.000 | 4029 | tags=39%, list=31%, signal=56% |
| 219 | POSITIVE\_REGULATION\_OF\_HYDROLASE\_ACTIVITY |  | 45 | 0.16 | 0.72 | 0.923 | 0.966 | 1.000 | 3161 | tags=27%, list=24%, signal=35% |
| 220 | REGULATION\_OF\_CATABOLIC\_PROCESS |  | 15 | 0.23 | 0.71 | 0.834 | 0.971 | 1.000 | 2099 | tags=27%, list=16%, signal=32% |
| 221 | JNK\_CASCADE |  | 44 | 0.17 | 0.70 | 0.919 | 0.976 | 1.000 | 4083 | tags=39%, list=31%, signal=56% |
| 222 | STRESS\_ACTIVATED\_PROTEIN\_KINASE\_SIGNALING\_PATHWAY |  | 45 | 0.16 | 0.70 | 0.936 | 0.975 | 1.000 | 4083 | tags=38%, list=31%, signal=55% |
| 223 | RHO\_PROTEIN\_SIGNAL\_TRANSDUCTION |  | 30 | 0.18 | 0.69 | 0.887 | 0.975 | 1.000 | 3901 | tags=33%, list=30%, signal=47% |
| 224 | GLUCOSE\_METABOLIC\_PROCESS |  | 27 | 0.18 | 0.69 | 0.905 | 0.974 | 1.000 | 4350 | tags=37%, list=33%, signal=55% |
| 225 | PROTEIN\_AMINO\_ACID\_LIPIDATION |  | 21 | 0.19 | 0.66 | 0.915 | 0.991 | 1.000 | 983 | tags=14%, list=8%, signal=15% |
| 226 | BIOGENIC\_AMINE\_METABOLIC\_PROCESS |  | 16 | 0.20 | 0.65 | 0.915 | 0.989 | 1.000 | 1540 | tags=19%, list=12%, signal=21% |
| 227 | ANION\_TRANSPORT |  | 27 | 0.17 | 0.65 | 0.942 | 0.985 | 1.000 | 1498 | tags=15%, list=11%, signal=17% |
| 228 | TUBE\_DEVELOPMENT |  | 15 | 0.20 | 0.65 | 0.921 | 0.984 | 1.000 | 3656 | tags=33%, list=28%, signal=46% |
| 229 | NEGATIVE\_REGULATION\_OF\_TRANSLATION |  | 19 | 0.19 | 0.64 | 0.925 | 0.984 | 1.000 | 3176 | tags=26%, list=24%, signal=35% |
| 230 | LIPOPROTEIN\_BIOSYNTHETIC\_PROCESS |  | 23 | 0.18 | 0.64 | 0.941 | 0.981 | 1.000 | 3855 | tags=35%, list=29%, signal=49% |
| 231 | FEEDING\_BEHAVIOR |  | 20 | 0.18 | 0.61 | 0.956 | 0.987 | 1.000 | 5017 | tags=45%, list=38%, signal=73% |
| 232 | ADENYLATE\_CYCLASE\_ACTIVATION |  | 18 | 0.18 | 0.61 | 0.954 | 0.984 | 1.000 | 4586 | tags=39%, list=35%, signal=60% |
| 233 | REGULATION\_OF\_HEART\_CONTRACTION |  | 24 | 0.15 | 0.53 | 0.988 | 0.997 | 1.000 | 3939 | tags=33%, list=30%, signal=48% |
| 234 | PEROXISOME\_ORGANIZATION\_AND\_BIOGENESIS |  | 15 | 0.14 | 0.43 | 1.000 | 0.999 | 1.000 | 11279 | tags=100%, list=86%, signal=722% |
Table: Gene sets enriched in phenotype **na**[plain text format]****

  
