# Supplementary material for: Multiple-input multiple-output causal strategies for gene selection
Source: BMC Bioinformatics. 2011 Nov 25;12:458. doi: 10.1186/1471-2105-12-458 (PMC3323860; doi:10.1186/1471-2105-12-458)
Supplement: Additional file 3 — Archive containing the output files computed by the preranked GSEA for λ ∈ {0.6,0.7,0.8,0.9,1.0,2.0} (GSEA_MIMO_part2.zip). [file 1471-2105-12-458-S3.ZIP › mFS20_entrez_mimo.GseaPreranked.1316039690234/gsea_report_for_na_neg_1316039690234.html]

Report for na\_neg 1316039690234 [GSEA]

| GS  follow link to MSigDB | GS DETAILS | SIZE | ES | NES | NOM p-val | FDR q-val | FWER p-val | RANK AT MAX | LEADING EDGE || 1 | DEFENSE\_RESPONSE |  | 238 | -0.42 | -2.54 | 0.000 | 0.000 | 0.000 | 3451 | tags=44%, list=26%, signal=58% |
| 2 | IMMUNE\_RESPONSE |  | 212 | -0.41 | -2.46 | 0.000 | 0.000 | 0.000 | 3451 | tags=48%, list=26%, signal=64% |
| 3 | IMMUNE\_SYSTEM\_PROCESS |  | 298 | -0.38 | -2.38 | 0.000 | 0.001 | 0.002 | 2866 | tags=40%, list=22%, signal=50% |
| 4 | REGULATION\_OF\_IMMUNE\_RESPONSE |  | 28 | -0.57 | -2.30 | 0.000 | 0.002 | 0.006 | 2833 | tags=57%, list=22%, signal=73% |
| 5 | POSITIVE\_REGULATION\_OF\_IMMUNE\_RESPONSE |  | 24 | -0.60 | -2.26 | 0.000 | 0.003 | 0.010 | 2833 | tags=58%, list=22%, signal=74% |
| 6 | POSITIVE\_REGULATION\_OF\_IMMUNE\_SYSTEM\_PROCESS |  | 44 | -0.51 | -2.25 | 0.000 | 0.002 | 0.010 | 3408 | tags=52%, list=26%, signal=70% |
| 7 | INFLAMMATORY\_RESPONSE |  | 115 | -0.41 | -2.22 | 0.000 | 0.002 | 0.013 | 2811 | tags=39%, list=21%, signal=49% |
| 8 | REGULATION\_OF\_IMMUNE\_SYSTEM\_PROCESS |  | 57 | -0.46 | -2.16 | 0.000 | 0.004 | 0.026 | 3445 | tags=53%, list=26%, signal=71% |
| 9 | CELLULAR\_DEFENSE\_RESPONSE |  | 54 | -0.46 | -2.11 | 0.000 | 0.007 | 0.048 | 2900 | tags=43%, list=22%, signal=54% |
| 10 | POSITIVE\_REGULATION\_OF\_MULTICELLULAR\_ORGANISMAL\_PROCESS |  | 56 | -0.45 | -2.10 | 0.000 | 0.007 | 0.051 | 3515 | tags=50%, list=27%, signal=68% |
| 11 | RESPONSE\_TO\_WOUNDING |  | 171 | -0.37 | -2.10 | 0.000 | 0.006 | 0.052 | 3230 | tags=40%, list=25%, signal=52% |
| 12 | ADAPTIVE\_IMMUNE\_RESPONSE\_GO\_0002460 |  | 22 | -0.52 | -1.88 | 0.011 | 0.046 | 0.352 | 2833 | tags=50%, list=22%, signal=64% |
| 13 | REGULATION\_OF\_MULTICELLULAR\_ORGANISMAL\_PROCESS |  | 131 | -0.33 | -1.85 | 0.000 | 0.057 | 0.442 | 3729 | tags=45%, list=28%, signal=62% |
| 14 | LYMPHOCYTE\_ACTIVATION |  | 54 | -0.40 | -1.85 | 0.000 | 0.054 | 0.447 | 2949 | tags=44%, list=23%, signal=57% |
| 15 | ADAPTIVE\_IMMUNE\_RESPONSE |  | 23 | -0.49 | -1.85 | 0.002 | 0.051 | 0.450 | 2833 | tags=48%, list=22%, signal=61% |
| 16 | IMMUNE\_EFFECTOR\_PROCESS |  | 34 | -0.45 | -1.85 | 0.000 | 0.048 | 0.455 | 3671 | tags=62%, list=28%, signal=86% |
| 17 | IMMUNE\_SYSTEM\_DEVELOPMENT |  | 75 | -0.37 | -1.84 | 0.000 | 0.048 | 0.476 | 2949 | tags=41%, list=23%, signal=53% |
| 18 | HEMOPOIETIC\_OR\_LYMPHOID\_ORGAN\_DEVELOPMENT |  | 71 | -0.37 | -1.84 | 0.000 | 0.046 | 0.477 | 2949 | tags=41%, list=23%, signal=52% |
| 19 | HEMOPOIESIS |  | 69 | -0.37 | -1.83 | 0.002 | 0.047 | 0.499 | 2949 | tags=41%, list=23%, signal=52% |
| 20 | CELL\_ACTIVATION |  | 64 | -0.37 | -1.81 | 0.000 | 0.052 | 0.558 | 2976 | tags=44%, list=23%, signal=56% |
| 21 | RESPONSE\_TO\_EXTERNAL\_STIMULUS |  | 278 | -0.29 | -1.79 | 0.000 | 0.057 | 0.614 | 3263 | tags=35%, list=25%, signal=45% |
| 22 | RECEPTOR\_MEDIATED\_ENDOCYTOSIS |  | 31 | -0.46 | -1.79 | 0.007 | 0.056 | 0.625 | 1848 | tags=35%, list=14%, signal=41% |
| 23 | T\_CELL\_ACTIVATION |  | 39 | -0.41 | -1.76 | 0.002 | 0.067 | 0.705 | 2949 | tags=41%, list=23%, signal=53% |
| 24 | LEUKOCYTE\_ACTIVATION |  | 59 | -0.37 | -1.74 | 0.004 | 0.075 | 0.757 | 2949 | tags=42%, list=23%, signal=54% |
| 25 | POSITIVE\_REGULATION\_OF\_RESPONSE\_TO\_STIMULUS |  | 35 | -0.41 | -1.74 | 0.009 | 0.074 | 0.767 | 2833 | tags=46%, list=22%, signal=58% |
| 26 | LEUKOCYTE\_DIFFERENTIATION |  | 34 | -0.42 | -1.73 | 0.002 | 0.072 | 0.772 | 2949 | tags=47%, list=23%, signal=61% |
| 27 | REGULATION\_OF\_DEFENSE\_RESPONSE |  | 15 | -0.53 | -1.72 | 0.019 | 0.080 | 0.821 | 3451 | tags=60%, list=26%, signal=81% |
| 28 | HUMORAL\_IMMUNE\_RESPONSE |  | 30 | -0.42 | -1.68 | 0.013 | 0.105 | 0.897 | 3842 | tags=63%, list=29%, signal=89% |
| 29 | JAK\_STAT\_CASCADE |  | 26 | -0.43 | -1.67 | 0.019 | 0.105 | 0.906 | 2580 | tags=38%, list=20%, signal=48% |
| 30 | REGULATION\_OF\_RESPONSE\_TO\_STIMULUS |  | 49 | -0.37 | -1.65 | 0.002 | 0.118 | 0.932 | 3451 | tags=49%, list=26%, signal=66% |
| 31 | LYMPHOCYTE\_DIFFERENTIATION |  | 23 | -0.45 | -1.65 | 0.013 | 0.115 | 0.933 | 2949 | tags=52%, list=23%, signal=67% |
| 32 | B\_CELL\_ACTIVATION |  | 17 | -0.48 | -1.63 | 0.027 | 0.126 | 0.950 | 2811 | tags=59%, list=21%, signal=75% |
| 33 | POSITIVE\_REGULATION\_OF\_SIGNAL\_TRANSDUCTION |  | 97 | -0.31 | -1.63 | 0.007 | 0.123 | 0.951 | 2772 | tags=35%, list=21%, signal=44% |
| 34 | MULTI\_ORGANISM\_PROCESS |  | 137 | -0.29 | -1.62 | 0.000 | 0.131 | 0.963 | 3632 | tags=42%, list=28%, signal=58% |
| 35 | DETECTION\_OF\_STIMULUS |  | 36 | -0.38 | -1.59 | 0.015 | 0.155 | 0.984 | 4710 | tags=61%, list=36%, signal=95% |
| 36 | CELL\_SUBSTRATE\_ADHESION |  | 36 | -0.37 | -1.59 | 0.017 | 0.154 | 0.984 | 2211 | tags=36%, list=17%, signal=43% |
| 37 | REGULATION\_OF\_SIGNAL\_TRANSDUCTION |  | 173 | -0.27 | -1.59 | 0.000 | 0.152 | 0.984 | 2772 | tags=31%, list=21%, signal=39% |
| 38 | NEGATIVE\_REGULATION\_OF\_SIGNAL\_TRANSDUCTION |  | 31 | -0.39 | -1.54 | 0.041 | 0.206 | 0.999 | 2709 | tags=35%, list=21%, signal=45% |
| 39 | REGULATION\_OF\_LYMPHOCYTE\_ACTIVATION |  | 31 | -0.39 | -1.53 | 0.040 | 0.208 | 0.999 | 2949 | tags=42%, list=23%, signal=54% |
| 40 | INNATE\_IMMUNE\_RESPONSE |  | 19 | -0.43 | -1.53 | 0.041 | 0.211 | 0.999 | 3445 | tags=53%, list=26%, signal=71% |
| 41 | CYTOKINE\_AND\_CHEMOKINE\_MEDIATED\_SIGNALING\_PATHWAY |  | 19 | -0.44 | -1.51 | 0.034 | 0.227 | 0.999 | 1278 | tags=32%, list=10%, signal=35% |
| 42 | ENZYME\_LINKED\_RECEPTOR\_PROTEIN\_SIGNALING\_PATHWAY |  | 128 | -0.27 | -1.51 | 0.005 | 0.222 | 0.999 | 1918 | tags=24%, list=15%, signal=28% |
| 43 | CATION\_HOMEOSTASIS |  | 94 | -0.29 | -1.51 | 0.011 | 0.222 | 0.999 | 3974 | tags=45%, list=30%, signal=64% |
| 44 | MAINTENANCE\_OF\_LOCALIZATION |  | 21 | -0.43 | -1.51 | 0.043 | 0.220 | 0.999 | 3460 | tags=43%, list=26%, signal=58% |
| 45 | REGULATION\_OF\_T\_CELL\_ACTIVATION |  | 25 | -0.39 | -1.50 | 0.032 | 0.222 | 0.999 | 3408 | tags=44%, list=26%, signal=59% |
| 46 | CELLULAR\_CATION\_HOMEOSTASIS |  | 91 | -0.29 | -1.50 | 0.014 | 0.225 | 0.999 | 3974 | tags=45%, list=30%, signal=64% |
| 47 | PROTEIN\_AMINO\_ACID\_N\_LINKED\_GLYCOSYLATION |  | 27 | -0.38 | -1.49 | 0.030 | 0.228 | 0.999 | 1970 | tags=33%, list=15%, signal=39% |
| 48 | PEPTIDYL\_TYROSINE\_MODIFICATION |  | 23 | -0.41 | -1.49 | 0.044 | 0.227 | 0.999 | 2211 | tags=30%, list=17%, signal=37% |
| 49 | RESPONSE\_TO\_OTHER\_ORGANISM |  | 69 | -0.31 | -1.49 | 0.015 | 0.229 | 1.000 | 3632 | tags=43%, list=28%, signal=60% |
| 50 | RESPONSE\_TO\_BACTERIUM |  | 22 | -0.40 | -1.49 | 0.044 | 0.224 | 1.000 | 3240 | tags=45%, list=25%, signal=60% |
| 51 | POSITIVE\_REGULATION\_OF\_LYMPHOCYTE\_ACTIVATION |  | 23 | -0.40 | -1.49 | 0.040 | 0.221 | 1.000 | 3408 | tags=43%, list=26%, signal=59% |
| 52 | LIPID\_CATABOLIC\_PROCESS |  | 34 | -0.36 | -1.48 | 0.041 | 0.217 | 1.000 | 3955 | tags=53%, list=30%, signal=76% |
| 53 | REGULATION\_OF\_CYTOSKELETON\_ORGANIZATION\_AND\_BIOGENESIS |  | 26 | -0.38 | -1.48 | 0.043 | 0.220 | 1.000 | 2211 | tags=31%, list=17%, signal=37% |
| 54 | REGULATION\_OF\_CELL\_DIFFERENTIATION |  | 48 | -0.33 | -1.48 | 0.022 | 0.216 | 1.000 | 3425 | tags=38%, list=26%, signal=51% |
| 55 | CELL\_MATRIX\_ADHESION |  | 35 | -0.35 | -1.47 | 0.035 | 0.218 | 1.000 | 2211 | tags=34%, list=17%, signal=41% |
| 56 | REGULATION\_OF\_PROTEIN\_AMINO\_ACID\_PHOSPHORYLATION |  | 23 | -0.40 | -1.47 | 0.058 | 0.219 | 1.000 | 2211 | tags=35%, list=17%, signal=42% |
| 57 | ACTIVATION\_OF\_NF\_KAPPAB\_TRANSCRIPTION\_FACTOR |  | 15 | -0.46 | -1.47 | 0.071 | 0.222 | 1.000 | 3884 | tags=67%, list=30%, signal=95% |
| 58 | ACTIN\_CYTOSKELETON\_ORGANIZATION\_AND\_BIOGENESIS |  | 90 | -0.28 | -1.46 | 0.019 | 0.226 | 1.000 | 2551 | tags=29%, list=19%, signal=36% |
| 59 | ACTIN\_POLYMERIZATION\_AND\_OR\_DEPOLYMERIZATION |  | 20 | -0.41 | -1.46 | 0.074 | 0.227 | 1.000 | 817 | tags=20%, list=6%, signal=21% |
| 60 | POSITIVE\_REGULATION\_OF\_PHOSPHATE\_METABOLIC\_PROCESS |  | 23 | -0.39 | -1.45 | 0.047 | 0.238 | 1.000 | 772 | tags=26%, list=6%, signal=28% |
| 61 | PROTEIN\_KINASE\_CASCADE |  | 239 | -0.24 | -1.44 | 0.007 | 0.251 | 1.000 | 2580 | tags=27%, list=20%, signal=33% |
| 62 | GLYCOPROTEIN\_METABOLIC\_PROCESS |  | 82 | -0.28 | -1.44 | 0.020 | 0.248 | 1.000 | 3769 | tags=40%, list=29%, signal=56% |
| 63 | POSITIVE\_REGULATION\_OF\_CELL\_DIFFERENTIATION |  | 21 | -0.40 | -1.43 | 0.081 | 0.249 | 1.000 | 4469 | tags=62%, list=34%, signal=94% |
| 64 | REGULATION\_OF\_ANATOMICAL\_STRUCTURE\_MORPHOGENESIS |  | 17 | -0.42 | -1.43 | 0.086 | 0.247 | 1.000 | 3657 | tags=41%, list=28%, signal=57% |
| 65 | DEFENSE\_RESPONSE\_TO\_BACTERIUM |  | 16 | -0.43 | -1.43 | 0.079 | 0.244 | 1.000 | 2970 | tags=44%, list=23%, signal=57% |
| 66 | TRANSFORMING\_GROWTH\_FACTOR\_BETA\_RECEPTOR\_SIGNALING\_PATHWAY |  | 34 | -0.35 | -1.43 | 0.065 | 0.241 | 1.000 | 3008 | tags=41%, list=23%, signal=53% |
| 67 | TRANSMEMBRANE\_RECEPTOR\_PROTEIN\_SERINE\_THREONINE\_KINASE\_SIGNALING\_PATHWAY |  | 42 | -0.33 | -1.43 | 0.055 | 0.242 | 1.000 | 3008 | tags=38%, list=23%, signal=49% |
| 68 | REGULATION\_OF\_ANGIOGENESIS |  | 24 | -0.38 | -1.43 | 0.068 | 0.238 | 1.000 | 1894 | tags=42%, list=14%, signal=49% |
| 69 | PROTEIN\_COMPLEX\_ASSEMBLY |  | 157 | -0.25 | -1.42 | 0.012 | 0.240 | 1.000 | 2751 | tags=29%, list=21%, signal=37% |
| 70 | POSITIVE\_REGULATION\_OF\_CELL\_PROLIFERATION |  | 129 | -0.25 | -1.40 | 0.018 | 0.270 | 1.000 | 1530 | tags=22%, list=12%, signal=24% |
| 71 | TRANSMEMBRANE\_RECEPTOR\_PROTEIN\_TYROSINE\_KINASE\_SIGNALING\_PATHWAY |  | 76 | -0.28 | -1.40 | 0.045 | 0.271 | 1.000 | 1918 | tags=24%, list=15%, signal=28% |
| 72 | PEPTIDYL\_TYROSINE\_PHOSPHORYLATION |  | 21 | -0.38 | -1.38 | 0.095 | 0.290 | 1.000 | 2211 | tags=29%, list=17%, signal=34% |
| 73 | RESPONSE\_TO\_VIRUS |  | 45 | -0.31 | -1.38 | 0.061 | 0.294 | 1.000 | 893 | tags=24%, list=7%, signal=26% |
| 74 | POSITIVE\_REGULATION\_OF\_CYTOKINE\_BIOSYNTHETIC\_PROCESS |  | 21 | -0.38 | -1.37 | 0.104 | 0.312 | 1.000 | 2667 | tags=43%, list=20%, signal=54% |
| 75 | REGULATION\_OF\_CELL\_PROLIFERATION |  | 275 | -0.22 | -1.36 | 0.012 | 0.318 | 1.000 | 1984 | tags=22%, list=15%, signal=26% |
| 76 | REGULATION\_OF\_PROTEIN\_IMPORT\_INTO\_NUCLEUS |  | 15 | -0.42 | -1.36 | 0.109 | 0.323 | 1.000 | 2319 | tags=33%, list=18%, signal=40% |
| 77 | POSITIVE\_REGULATION\_OF\_CELLULAR\_PROTEIN\_METABOLIC\_PROCESS |  | 61 | -0.28 | -1.35 | 0.070 | 0.323 | 1.000 | 2690 | tags=34%, list=21%, signal=43% |
| 78 | CYTOKINE\_PRODUCTION |  | 61 | -0.29 | -1.35 | 0.050 | 0.323 | 1.000 | 2833 | tags=34%, list=22%, signal=44% |
| 79 | I\_KAPPAB\_KINASE\_NF\_KAPPAB\_CASCADE |  | 88 | -0.26 | -1.34 | 0.056 | 0.340 | 1.000 | 2709 | tags=31%, list=21%, signal=38% |
| 80 | CELL\_RECOGNITION |  | 16 | -0.40 | -1.32 | 0.143 | 0.388 | 1.000 | 3558 | tags=44%, list=27%, signal=60% |
| 81 | POSITIVE\_REGULATION\_OF\_I\_KAPPAB\_KINASE\_NF\_KAPPAB\_CASCADE |  | 67 | -0.27 | -1.31 | 0.087 | 0.395 | 1.000 | 2567 | tags=31%, list=20%, signal=39% |
| 82 | REGULATION\_OF\_MAPKKK\_CASCADE |  | 19 | -0.38 | -1.31 | 0.148 | 0.397 | 1.000 | 1673 | tags=32%, list=13%, signal=36% |
| 83 | POSITIVE\_REGULATION\_OF\_TRANSCRIPTION\_FACTOR\_ACTIVITY |  | 17 | -0.38 | -1.31 | 0.154 | 0.393 | 1.000 | 3884 | tags=59%, list=30%, signal=84% |
| 84 | REGULATION\_OF\_I\_KAPPAB\_KINASE\_NF\_KAPPAB\_CASCADE |  | 72 | -0.26 | -1.30 | 0.089 | 0.394 | 1.000 | 2709 | tags=32%, list=21%, signal=40% |
| 85 | BEHAVIOR |  | 136 | -0.23 | -1.30 | 0.073 | 0.400 | 1.000 | 3875 | tags=37%, list=30%, signal=52% |
| 86 | CELLULAR\_LIPID\_CATABOLIC\_PROCESS |  | 31 | -0.32 | -1.29 | 0.134 | 0.403 | 1.000 | 3955 | tags=48%, list=30%, signal=69% |
| 87 | REGULATION\_OF\_ORGANELLE\_ORGANIZATION\_AND\_BIOGENESIS |  | 35 | -0.31 | -1.29 | 0.137 | 0.402 | 1.000 | 2211 | tags=29%, list=17%, signal=34% |
| 88 | POSITIVE\_REGULATION\_OF\_PROTEIN\_METABOLIC\_PROCESS |  | 63 | -0.27 | -1.29 | 0.103 | 0.399 | 1.000 | 2690 | tags=33%, list=21%, signal=42% |
| 89 | PHOSPHOLIPID\_METABOLIC\_PROCESS |  | 63 | -0.26 | -1.29 | 0.098 | 0.409 | 1.000 | 2953 | tags=33%, list=23%, signal=43% |
| 90 | MYELOID\_CELL\_DIFFERENTIATION |  | 35 | -0.31 | -1.28 | 0.121 | 0.408 | 1.000 | 2557 | tags=31%, list=20%, signal=39% |
| 91 | POSITIVE\_REGULATION\_OF\_TRANSLATION |  | 28 | -0.32 | -1.28 | 0.137 | 0.404 | 1.000 | 2667 | tags=39%, list=20%, signal=49% |
| 92 | POSITIVE\_REGULATION\_OF\_T\_CELL\_ACTIVATION |  | 20 | -0.36 | -1.28 | 0.159 | 0.416 | 1.000 | 3408 | tags=40%, list=26%, signal=54% |
| 93 | PROTEIN\_OLIGOMERIZATION |  | 37 | -0.31 | -1.27 | 0.115 | 0.417 | 1.000 | 2034 | tags=27%, list=16%, signal=32% |
| 94 | MESODERM\_DEVELOPMENT |  | 22 | -0.35 | -1.27 | 0.172 | 0.427 | 1.000 | 2999 | tags=36%, list=23%, signal=47% |
| 95 | ACTIN\_FILAMENT\_BASED\_PROCESS |  | 99 | -0.24 | -1.27 | 0.102 | 0.425 | 1.000 | 2551 | tags=26%, list=19%, signal=32% |
| 96 | WOUND\_HEALING |  | 49 | -0.28 | -1.26 | 0.125 | 0.433 | 1.000 | 3230 | tags=35%, list=25%, signal=46% |
| 97 | POSITIVE\_REGULATION\_OF\_DNA\_BINDING |  | 18 | -0.37 | -1.26 | 0.168 | 0.430 | 1.000 | 3884 | tags=56%, list=30%, signal=79% |
| 98 | ICOSANOID\_METABOLIC\_PROCESS |  | 16 | -0.39 | -1.26 | 0.204 | 0.428 | 1.000 | 1604 | tags=31%, list=12%, signal=36% |
| 99 | LOCOMOTORY\_BEHAVIOR |  | 84 | -0.25 | -1.25 | 0.118 | 0.434 | 1.000 | 1974 | tags=24%, list=15%, signal=28% |
| 100 | ACTIVATION\_OF\_MAPK\_ACTIVITY |  | 33 | -0.31 | -1.25 | 0.148 | 0.431 | 1.000 | 1410 | tags=27%, list=11%, signal=30% |
| 101 | POSITIVE\_REGULATION\_OF\_PHOSPHORYLATION |  | 21 | -0.35 | -1.25 | 0.183 | 0.440 | 1.000 | 761 | tags=24%, list=6%, signal=25% |
| 102 | SMALL\_GTPASE\_MEDIATED\_SIGNAL\_TRANSDUCTION |  | 77 | -0.25 | -1.24 | 0.108 | 0.459 | 1.000 | 3601 | tags=38%, list=28%, signal=52% |
| 103 | ION\_HOMEOSTASIS |  | 112 | -0.23 | -1.24 | 0.087 | 0.455 | 1.000 | 3974 | tags=41%, list=30%, signal=58% |
| 104 | MONOCARBOXYLIC\_ACID\_METABOLIC\_PROCESS |  | 77 | -0.25 | -1.24 | 0.115 | 0.454 | 1.000 | 4215 | tags=44%, list=32%, signal=65% |
| 105 | ANATOMICAL\_STRUCTURE\_FORMATION |  | 52 | -0.26 | -1.23 | 0.153 | 0.459 | 1.000 | 1954 | tags=29%, list=15%, signal=34% |
| 106 | ANGIOGENESIS |  | 44 | -0.28 | -1.23 | 0.159 | 0.468 | 1.000 | 1894 | tags=30%, list=14%, signal=34% |
| 107 | CYTOKINE\_BIOSYNTHETIC\_PROCESS |  | 34 | -0.30 | -1.23 | 0.181 | 0.467 | 1.000 | 2667 | tags=35%, list=20%, signal=44% |
| 108 | POSITIVE\_REGULATION\_OF\_PROTEIN\_AMINO\_ACID\_PHOSPHORYLATION |  | 15 | -0.37 | -1.22 | 0.188 | 0.470 | 1.000 | 743 | tags=27%, list=6%, signal=28% |
| 109 | GROWTH |  | 59 | -0.26 | -1.22 | 0.159 | 0.470 | 1.000 | 4113 | tags=39%, list=31%, signal=57% |
| 110 | PROTEIN\_AMINO\_ACID\_PHOSPHORYLATION |  | 231 | -0.20 | -1.22 | 0.061 | 0.467 | 1.000 | 2368 | tags=23%, list=18%, signal=27% |
| 111 | RAS\_PROTEIN\_SIGNAL\_TRANSDUCTION |  | 55 | -0.27 | -1.22 | 0.157 | 0.465 | 1.000 | 2986 | tags=35%, list=23%, signal=45% |
| 112 | FEMALE\_PREGNANCY |  | 42 | -0.28 | -1.22 | 0.148 | 0.463 | 1.000 | 3630 | tags=43%, list=28%, signal=59% |
| 113 | G\_PROTEIN\_SIGNALING\_COUPLED\_TO\_CAMP\_NUCLEOTIDE\_SECOND\_MESSENGER |  | 62 | -0.25 | -1.21 | 0.168 | 0.473 | 1.000 | 1989 | tags=23%, list=15%, signal=27% |
| 114 | REGULATION\_OF\_CELLULAR\_PROTEIN\_METABOLIC\_PROCESS |  | 139 | -0.22 | -1.21 | 0.105 | 0.477 | 1.000 | 2699 | tags=27%, list=21%, signal=34% |
| 115 | CAMP\_MEDIATED\_SIGNALING |  | 63 | -0.25 | -1.21 | 0.159 | 0.478 | 1.000 | 1989 | tags=22%, list=15%, signal=26% |
| 116 | MUSCLE\_DEVELOPMENT |  | 85 | -0.24 | -1.20 | 0.129 | 0.485 | 1.000 | 3674 | tags=41%, list=28%, signal=57% |
| 117 | FATTY\_ACID\_METABOLIC\_PROCESS |  | 56 | -0.26 | -1.20 | 0.181 | 0.481 | 1.000 | 4202 | tags=46%, list=32%, signal=68% |
| 118 | POSITIVE\_REGULATION\_OF\_TRANSFERASE\_ACTIVITY |  | 71 | -0.24 | -1.20 | 0.154 | 0.481 | 1.000 | 2189 | tags=24%, list=17%, signal=29% |
| 119 | LIPID\_METABOLIC\_PROCESS |  | 283 | -0.19 | -1.20 | 0.093 | 0.477 | 1.000 | 2186 | tags=23%, list=17%, signal=27% |
| 120 | POSITIVE\_REGULATION\_OF\_DEVELOPMENTAL\_PROCESS |  | 197 | -0.20 | -1.20 | 0.101 | 0.476 | 1.000 | 3005 | tags=31%, list=23%, signal=40% |
| 121 | REGULATION\_OF\_DEVELOPMENTAL\_PROCESS |  | 387 | -0.18 | -1.20 | 0.049 | 0.473 | 1.000 | 3890 | tags=37%, list=30%, signal=51% |
| 122 | RESPONSE\_TO\_BIOTIC\_STIMULUS |  | 103 | -0.23 | -1.20 | 0.143 | 0.473 | 1.000 | 1906 | tags=24%, list=15%, signal=28% |
| 123 | MEMBRANE\_ORGANIZATION\_AND\_BIOGENESIS |  | 124 | -0.22 | -1.20 | 0.133 | 0.470 | 1.000 | 3256 | tags=33%, list=25%, signal=44% |
| 124 | BONE\_REMODELING |  | 28 | -0.31 | -1.20 | 0.219 | 0.469 | 1.000 | 3727 | tags=43%, list=28%, signal=60% |
| 125 | GLYCOPROTEIN\_BIOSYNTHETIC\_PROCESS |  | 67 | -0.25 | -1.20 | 0.183 | 0.468 | 1.000 | 3769 | tags=39%, list=29%, signal=54% |
| 126 | BLOOD\_COAGULATION |  | 41 | -0.27 | -1.19 | 0.211 | 0.468 | 1.000 | 3531 | tags=37%, list=27%, signal=50% |
| 127 | COAGULATION |  | 41 | -0.27 | -1.19 | 0.186 | 0.464 | 1.000 | 3531 | tags=37%, list=27%, signal=50% |
| 128 | REGULATION\_OF\_BODY\_FLUID\_LEVELS |  | 55 | -0.26 | -1.19 | 0.179 | 0.470 | 1.000 | 3262 | tags=35%, list=25%, signal=46% |
| 129 | REGULATION\_OF\_BLOOD\_PRESSURE |  | 22 | -0.32 | -1.19 | 0.222 | 0.477 | 1.000 | 3188 | tags=36%, list=24%, signal=48% |
| 130 | REGULATION\_OF\_PROTEIN\_METABOLIC\_PROCESS |  | 150 | -0.21 | -1.18 | 0.138 | 0.477 | 1.000 | 2699 | tags=27%, list=21%, signal=33% |
| 131 | POSITIVE\_REGULATION\_OF\_CELLULAR\_COMPONENT\_ORGANIZATION\_AND\_BIOGENESIS |  | 28 | -0.30 | -1.18 | 0.213 | 0.484 | 1.000 | 3157 | tags=36%, list=24%, signal=47% |
| 132 | POSITIVE\_REGULATION\_OF\_MAP\_KINASE\_ACTIVITY |  | 39 | -0.28 | -1.18 | 0.217 | 0.483 | 1.000 | 1460 | tags=26%, list=11%, signal=29% |
| 133 | CELLULAR\_PROTEIN\_COMPLEX\_ASSEMBLY |  | 28 | -0.30 | -1.18 | 0.214 | 0.481 | 1.000 | 632 | tags=18%, list=5%, signal=19% |
| 134 | VASCULATURE\_DEVELOPMENT |  | 50 | -0.25 | -1.17 | 0.197 | 0.487 | 1.000 | 1894 | tags=26%, list=14%, signal=30% |
| 135 | CYTOKINE\_METABOLIC\_PROCESS |  | 35 | -0.28 | -1.17 | 0.221 | 0.491 | 1.000 | 2667 | tags=34%, list=20%, signal=43% |
| 136 | PROTEIN\_AUTOPROCESSING |  | 24 | -0.31 | -1.16 | 0.250 | 0.513 | 1.000 | 4738 | tags=58%, list=36%, signal=91% |
| 137 | DEVELOPMENTAL\_MATURATION |  | 18 | -0.34 | -1.15 | 0.252 | 0.522 | 1.000 | 2393 | tags=33%, list=18%, signal=41% |
| 138 | POSITIVE\_REGULATION\_OF\_CATALYTIC\_ACTIVITY |  | 139 | -0.20 | -1.15 | 0.160 | 0.523 | 1.000 | 2265 | tags=22%, list=17%, signal=26% |
| 139 | GENERATION\_OF\_NEURONS |  | 65 | -0.24 | -1.15 | 0.204 | 0.522 | 1.000 | 3060 | tags=29%, list=23%, signal=38% |
| 140 | PROTEIN\_PROCESSING |  | 41 | -0.26 | -1.15 | 0.232 | 0.521 | 1.000 | 4738 | tags=51%, list=36%, signal=80% |
| 141 | DETECTION\_OF\_EXTERNAL\_STIMULUS |  | 18 | -0.34 | -1.15 | 0.252 | 0.521 | 1.000 | 4614 | tags=50%, list=35%, signal=77% |
| 142 | NEGATIVE\_REGULATION\_OF\_CELL\_PROLIFERATION |  | 145 | -0.20 | -1.15 | 0.157 | 0.519 | 1.000 | 1984 | tags=21%, list=15%, signal=25% |
| 143 | AMINO\_ACID\_TRANSPORT |  | 25 | -0.30 | -1.15 | 0.257 | 0.522 | 1.000 | 867 | tags=20%, list=7%, signal=21% |
| 144 | PROTEIN\_AMINO\_ACID\_AUTOPHOSPHORYLATION |  | 24 | -0.31 | -1.14 | 0.275 | 0.522 | 1.000 | 4738 | tags=58%, list=36%, signal=91% |
| 145 | POSITIVE\_REGULATION\_OF\_CELLULAR\_METABOLIC\_PROCESS |  | 196 | -0.20 | -1.14 | 0.160 | 0.524 | 1.000 | 2690 | tags=27%, list=21%, signal=33% |
| 146 | RESPONSE\_TO\_DRUG |  | 21 | -0.32 | -1.14 | 0.252 | 0.528 | 1.000 | 2984 | tags=38%, list=23%, signal=49% |
| 147 | ORGAN\_MORPHOGENESIS |  | 131 | -0.20 | -1.14 | 0.184 | 0.532 | 1.000 | 1338 | tags=18%, list=10%, signal=19% |
| 148 | POSITIVE\_REGULATION\_OF\_TRANSCRIPTION |  | 124 | -0.21 | -1.14 | 0.196 | 0.529 | 1.000 | 3301 | tags=31%, list=25%, signal=41% |
| 149 | PHOSPHORYLATION |  | 262 | -0.19 | -1.13 | 0.168 | 0.539 | 1.000 | 2368 | tags=22%, list=18%, signal=26% |
| 150 | SKELETAL\_DEVELOPMENT |  | 91 | -0.22 | -1.13 | 0.249 | 0.544 | 1.000 | 3816 | tags=38%, list=29%, signal=54% |
| 151 | HEMOSTASIS |  | 46 | -0.26 | -1.13 | 0.259 | 0.543 | 1.000 | 3230 | tags=33%, list=25%, signal=43% |
| 152 | POSITIVE\_REGULATION\_OF\_PROTEIN\_MODIFICATION\_PROCESS |  | 24 | -0.30 | -1.12 | 0.273 | 0.549 | 1.000 | 772 | tags=21%, list=6%, signal=22% |
| 153 | POSITIVE\_REGULATION\_OF\_METABOLIC\_PROCESS |  | 201 | -0.19 | -1.12 | 0.197 | 0.549 | 1.000 | 2690 | tags=26%, list=21%, signal=32% |
| 154 | AMINE\_TRANSPORT |  | 36 | -0.27 | -1.12 | 0.277 | 0.552 | 1.000 | 1898 | tags=22%, list=14%, signal=26% |
| 155 | REGULATION\_OF\_PHOSPHORYLATION |  | 42 | -0.25 | -1.11 | 0.287 | 0.557 | 1.000 | 2211 | tags=26%, list=17%, signal=31% |
| 156 | CHEMICAL\_HOMEOSTASIS |  | 136 | -0.20 | -1.11 | 0.244 | 0.564 | 1.000 | 3974 | tags=38%, list=30%, signal=54% |
| 157 | MACROMOLECULE\_BIOSYNTHETIC\_PROCESS |  | 267 | -0.18 | -1.11 | 0.201 | 0.564 | 1.000 | 2780 | tags=26%, list=21%, signal=32% |
| 158 | TISSUE\_REMODELING |  | 29 | -0.28 | -1.11 | 0.305 | 0.566 | 1.000 | 3727 | tags=41%, list=28%, signal=58% |
| 159 | CELLULAR\_COMPONENT\_ASSEMBLY |  | 272 | -0.18 | -1.10 | 0.212 | 0.569 | 1.000 | 2316 | tags=23%, list=18%, signal=27% |
| 160 | PEPTIDYL\_AMINO\_ACID\_MODIFICATION |  | 47 | -0.25 | -1.10 | 0.262 | 0.567 | 1.000 | 2211 | tags=26%, list=17%, signal=31% |
| 161 | CELLULAR\_HOMEOSTASIS |  | 121 | -0.20 | -1.10 | 0.228 | 0.568 | 1.000 | 3974 | tags=39%, list=30%, signal=55% |
| 162 | CYTOKINE\_SECRETION |  | 15 | -0.35 | -1.10 | 0.327 | 0.567 | 1.000 | 1860 | tags=33%, list=14%, signal=39% |
| 163 | NEGATIVE\_REGULATION\_OF\_TRANSCRIPTION |  | 166 | -0.19 | -1.10 | 0.219 | 0.564 | 1.000 | 2051 | tags=22%, list=16%, signal=25% |
| 164 | MEMBRANE\_LIPID\_METABOLIC\_PROCESS |  | 85 | -0.22 | -1.10 | 0.275 | 0.571 | 1.000 | 3710 | tags=38%, list=28%, signal=52% |
| 165 | HORMONE\_METABOLIC\_PROCESS |  | 29 | -0.28 | -1.10 | 0.302 | 0.571 | 1.000 | 3994 | tags=52%, list=31%, signal=74% |
| 166 | NEURON\_DEVELOPMENT |  | 49 | -0.24 | -1.09 | 0.286 | 0.574 | 1.000 | 3060 | tags=29%, list=23%, signal=37% |
| 167 | ORGANIC\_ACID\_METABOLIC\_PROCESS |  | 162 | -0.19 | -1.09 | 0.252 | 0.573 | 1.000 | 4233 | tags=40%, list=32%, signal=59% |
| 168 | REGULATION\_OF\_TRANSCRIPTION\_FACTOR\_ACTIVITY |  | 30 | -0.27 | -1.09 | 0.305 | 0.572 | 1.000 | 4099 | tags=50%, list=31%, signal=73% |
| 169 | CARBOXYLIC\_ACID\_METABOLIC\_PROCESS |  | 160 | -0.19 | -1.09 | 0.265 | 0.577 | 1.000 | 4233 | tags=41%, list=32%, signal=59% |
| 170 | NEURON\_DIFFERENTIATION |  | 58 | -0.23 | -1.09 | 0.298 | 0.578 | 1.000 | 3310 | tags=29%, list=25%, signal=39% |
| 171 | REGULATION\_OF\_CYTOKINE\_BIOSYNTHETIC\_PROCESS |  | 31 | -0.26 | -1.08 | 0.329 | 0.599 | 1.000 | 2667 | tags=32%, list=20%, signal=40% |
| 172 | GLYCEROPHOSPHOLIPID\_METABOLIC\_PROCESS |  | 39 | -0.25 | -1.08 | 0.349 | 0.598 | 1.000 | 4437 | tags=49%, list=34%, signal=73% |
| 173 | POSITIVE\_REGULATION\_OF\_BINDING |  | 19 | -0.31 | -1.07 | 0.353 | 0.601 | 1.000 | 3884 | tags=53%, list=30%, signal=75% |
| 174 | REGULATION\_OF\_TRANSCRIPTION |  | 498 | -0.16 | -1.07 | 0.221 | 0.603 | 1.000 | 3650 | tags=32%, list=28%, signal=42% |
| 175 | REGULATION\_OF\_BIOLOGICAL\_QUALITY |  | 364 | -0.17 | -1.07 | 0.247 | 0.599 | 1.000 | 3994 | tags=34%, list=31%, signal=47% |
| 176 | POST\_TRANSLATIONAL\_PROTEIN\_MODIFICATION |  | 409 | -0.16 | -1.07 | 0.241 | 0.605 | 1.000 | 2738 | tags=24%, list=21%, signal=29% |
| 177 | TRANSLATION |  | 149 | -0.19 | -1.07 | 0.324 | 0.605 | 1.000 | 2564 | tags=26%, list=20%, signal=32% |
| 178 | ORGANIC\_ACID\_TRANSPORT |  | 39 | -0.25 | -1.06 | 0.334 | 0.617 | 1.000 | 867 | tags=18%, list=7%, signal=19% |
| 179 | POSITIVE\_REGULATION\_OF\_SECRETION |  | 18 | -0.31 | -1.06 | 0.338 | 0.613 | 1.000 | 1379 | tags=22%, list=11%, signal=25% |
| 180 | CARBOXYLIC\_ACID\_TRANSPORT |  | 39 | -0.25 | -1.06 | 0.361 | 0.623 | 1.000 | 867 | tags=18%, list=7%, signal=19% |
| 181 | NEGATIVE\_REGULATION\_OF\_CELL\_DIFFERENTIATION |  | 24 | -0.28 | -1.05 | 0.370 | 0.628 | 1.000 | 2239 | tags=25%, list=17%, signal=30% |
| 182 | POSITIVE\_REGULATION\_OF\_NUCLEOBASENUCLEOSIDENUCLEOTIDE\_AND\_NUCLEIC\_ACID\_METABOLIC\_PROCESS |  | 134 | -0.19 | -1.05 | 0.333 | 0.628 | 1.000 | 3301 | tags=30%, list=25%, signal=40% |
| 183 | PROTEIN\_AMINO\_ACID\_DEPHOSPHORYLATION |  | 60 | -0.22 | -1.05 | 0.346 | 0.634 | 1.000 | 1197 | tags=17%, list=9%, signal=18% |
| 184 | ANTI\_APOPTOSIS |  | 107 | -0.19 | -1.05 | 0.339 | 0.636 | 1.000 | 1817 | tags=22%, list=14%, signal=26% |
| 185 | CARBOHYDRATE\_BIOSYNTHETIC\_PROCESS |  | 35 | -0.25 | -1.05 | 0.362 | 0.633 | 1.000 | 4215 | tags=40%, list=32%, signal=59% |
| 186 | CELL\_PROLIFERATION\_GO\_0008283 |  | 466 | -0.16 | -1.05 | 0.287 | 0.632 | 1.000 | 1986 | tags=19%, list=15%, signal=21% |
| 187 | PHOSPHOINOSITIDE\_METABOLIC\_PROCESS |  | 25 | -0.28 | -1.04 | 0.394 | 0.634 | 1.000 | 2780 | tags=36%, list=21%, signal=46% |
| 188 | REGULATION\_OF\_JNK\_ACTIVITY |  | 18 | -0.30 | -1.04 | 0.394 | 0.631 | 1.000 | 1410 | tags=28%, list=11%, signal=31% |
| 189 | HOMEOSTATIC\_PROCESS |  | 179 | -0.18 | -1.04 | 0.358 | 0.630 | 1.000 | 3974 | tags=36%, list=30%, signal=51% |
| 190 | MACROMOLECULAR\_COMPLEX\_ASSEMBLY |  | 254 | -0.17 | -1.04 | 0.345 | 0.631 | 1.000 | 2306 | tags=22%, list=18%, signal=26% |
| 191 | MUSCLE\_CELL\_DIFFERENTIATION |  | 21 | -0.29 | -1.04 | 0.422 | 0.641 | 1.000 | 2393 | tags=33%, list=18%, signal=41% |
| 192 | NEGATIVE\_REGULATION\_OF\_DEVELOPMENTAL\_PROCESS |  | 177 | -0.18 | -1.03 | 0.369 | 0.645 | 1.000 | 2902 | tags=28%, list=22%, signal=36% |
| 193 | STRIATED\_MUSCLE\_DEVELOPMENT |  | 36 | -0.25 | -1.03 | 0.389 | 0.644 | 1.000 | 3115 | tags=39%, list=24%, signal=51% |
| 194 | REGULATION\_OF\_MYELOID\_CELL\_DIFFERENTIATION |  | 19 | -0.30 | -1.03 | 0.419 | 0.642 | 1.000 | 2557 | tags=32%, list=20%, signal=39% |
| 195 | GENERATION\_OF\_PRECURSOR\_METABOLITES\_AND\_ENERGY |  | 120 | -0.19 | -1.03 | 0.380 | 0.641 | 1.000 | 2209 | tags=23%, list=17%, signal=27% |
| 196 | POSITIVE\_REGULATION\_OF\_CASPASE\_ACTIVITY |  | 28 | -0.27 | -1.03 | 0.382 | 0.639 | 1.000 | 1719 | tags=25%, list=13%, signal=29% |
| 197 | CELLULAR\_LIPID\_METABOLIC\_PROCESS |  | 220 | -0.17 | -1.03 | 0.378 | 0.647 | 1.000 | 4202 | tags=39%, list=32%, signal=57% |
| 198 | RESPONSE\_TO\_NUTRIENT |  | 17 | -0.30 | -1.03 | 0.404 | 0.645 | 1.000 | 2014 | tags=29%, list=15%, signal=35% |
| 199 | REGULATION\_OF\_MAP\_KINASE\_ACTIVITY |  | 56 | -0.22 | -1.03 | 0.401 | 0.643 | 1.000 | 1460 | tags=21%, list=11%, signal=24% |
| 200 | VITAMIN\_METABOLIC\_PROCESS |  | 15 | -0.32 | -1.03 | 0.409 | 0.640 | 1.000 | 3740 | tags=47%, list=29%, signal=65% |
| 201 | NEGATIVE\_REGULATION\_OF\_METABOLIC\_PROCESS |  | 232 | -0.17 | -1.03 | 0.381 | 0.641 | 1.000 | 2051 | tags=20%, list=16%, signal=23% |
| 202 | RHYTHMIC\_PROCESS |  | 23 | -0.27 | -1.02 | 0.419 | 0.640 | 1.000 | 1795 | tags=26%, list=14%, signal=30% |
| 203 | T\_CELL\_PROLIFERATION |  | 17 | -0.30 | -1.02 | 0.400 | 0.651 | 1.000 | 2833 | tags=35%, list=22%, signal=45% |
| 204 | NEURITE\_DEVELOPMENT |  | 41 | -0.23 | -1.02 | 0.411 | 0.649 | 1.000 | 3025 | tags=27%, list=23%, signal=35% |
| 205 | CELL\_MATURATION |  | 16 | -0.30 | -1.02 | 0.426 | 0.653 | 1.000 | 2393 | tags=31%, list=18%, signal=38% |
| 206 | INTERACTION\_WITH\_HOST |  | 15 | -0.31 | -1.01 | 0.441 | 0.653 | 1.000 | 2117 | tags=33%, list=16%, signal=40% |
| 207 | AMINO\_ACID\_CATABOLIC\_PROCESS |  | 23 | -0.28 | -1.01 | 0.434 | 0.651 | 1.000 | 1128 | tags=22%, list=9%, signal=24% |
| 208 | AMINO\_ACID\_METABOLIC\_PROCESS |  | 73 | -0.21 | -1.01 | 0.414 | 0.653 | 1.000 | 1342 | tags=19%, list=10%, signal=21% |
| 209 | RESPONSE\_TO\_CHEMICAL\_STIMULUS |  | 271 | -0.16 | -1.01 | 0.425 | 0.665 | 1.000 | 2190 | tags=20%, list=17%, signal=24% |
| 210 | NEUROGENESIS |  | 75 | -0.20 | -1.01 | 0.452 | 0.663 | 1.000 | 3060 | tags=28%, list=23%, signal=36% |
| 211 | CELL\_CYCLE\_ARREST\_GO\_0007050 |  | 52 | -0.21 | -1.00 | 0.451 | 0.677 | 1.000 | 1894 | tags=25%, list=14%, signal=29% |
| 212 | REGULATION\_OF\_BINDING |  | 46 | -0.22 | -1.00 | 0.456 | 0.686 | 1.000 | 2721 | tags=30%, list=21%, signal=38% |
| 213 | REGULATION\_OF\_DNA\_BINDING |  | 36 | -0.23 | -1.00 | 0.448 | 0.684 | 1.000 | 4099 | tags=47%, list=31%, signal=69% |
| 214 | DEPHOSPHORYLATION |  | 67 | -0.20 | -0.99 | 0.453 | 0.683 | 1.000 | 1722 | tags=18%, list=13%, signal=21% |
| 215 | NEGATIVE\_REGULATION\_OF\_CELLULAR\_METABOLIC\_PROCESS |  | 229 | -0.16 | -0.99 | 0.482 | 0.685 | 1.000 | 2051 | tags=20%, list=16%, signal=23% |
| 216 | NEGATIVE\_REGULATION\_OF\_TRANSCRIPTION\_FROM\_RNA\_POLYMERASE\_II\_PROMOTER |  | 76 | -0.20 | -0.99 | 0.436 | 0.686 | 1.000 | 1865 | tags=21%, list=14%, signal=24% |
| 217 | NERVOUS\_SYSTEM\_DEVELOPMENT |  | 328 | -0.16 | -0.99 | 0.494 | 0.689 | 1.000 | 3643 | tags=30%, list=28%, signal=40% |
| 218 | PHAGOCYTOSIS |  | 16 | -0.30 | -0.98 | 0.465 | 0.695 | 1.000 | 5110 | tags=63%, list=39%, signal=102% |
| 219 | SULFUR\_METABOLIC\_PROCESS |  | 30 | -0.25 | -0.98 | 0.496 | 0.716 | 1.000 | 2964 | tags=30%, list=23%, signal=39% |
| 220 | NEGATIVE\_REGULATION\_OF\_NUCLEOBASENUCLEOSIDENUCLEOTIDE\_AND\_NUCLEIC\_ACID\_METABOLIC\_PROCESS |  | 185 | -0.16 | -0.98 | 0.523 | 0.714 | 1.000 | 2051 | tags=21%, list=16%, signal=24% |
| 221 | FATTY\_ACID\_OXIDATION |  | 17 | -0.29 | -0.97 | 0.493 | 0.725 | 1.000 | 3015 | tags=41%, list=23%, signal=53% |
| 222 | HEART\_DEVELOPMENT |  | 33 | -0.24 | -0.97 | 0.511 | 0.725 | 1.000 | 3425 | tags=36%, list=26%, signal=49% |
| 223 | GOLGI\_VESICLE\_TRANSPORT |  | 42 | -0.22 | -0.97 | 0.479 | 0.732 | 1.000 | 4547 | tags=48%, list=35%, signal=73% |
| 224 | AMINE\_CATABOLIC\_PROCESS |  | 25 | -0.26 | -0.96 | 0.500 | 0.731 | 1.000 | 1128 | tags=20%, list=9%, signal=22% |
| 225 | MAPKKK\_CASCADE\_GO\_0000165 |  | 90 | -0.19 | -0.96 | 0.522 | 0.731 | 1.000 | 1783 | tags=19%, list=14%, signal=22% |
| 226 | NEGATIVE\_REGULATION\_OF\_RNA\_METABOLIC\_PROCESS |  | 114 | -0.18 | -0.96 | 0.534 | 0.730 | 1.000 | 1954 | tags=20%, list=15%, signal=24% |
| 227 | REGULATION\_OF\_CELLULAR\_COMPONENT\_ORGANIZATION\_AND\_BIOGENESIS |  | 102 | -0.18 | -0.96 | 0.537 | 0.730 | 1.000 | 1158 | tags=15%, list=9%, signal=16% |
| 228 | REGULATION\_OF\_PROTEIN\_SECRETION |  | 19 | -0.28 | -0.96 | 0.511 | 0.728 | 1.000 | 1860 | tags=26%, list=14%, signal=31% |
| 229 | REGULATION\_OF\_PROTEIN\_MODIFICATION\_PROCESS |  | 37 | -0.23 | -0.95 | 0.530 | 0.741 | 1.000 | 2211 | tags=24%, list=17%, signal=29% |
| 230 | NITROGEN\_COMPOUND\_CATABOLIC\_PROCESS |  | 27 | -0.25 | -0.95 | 0.494 | 0.744 | 1.000 | 1128 | tags=19%, list=9%, signal=20% |
| 231 | NEGATIVE\_REGULATION\_OF\_TRANSCRIPTION\_DNA\_DEPENDENT |  | 114 | -0.18 | -0.95 | 0.541 | 0.746 | 1.000 | 1954 | tags=20%, list=15%, signal=24% |
| 232 | ELECTRON\_TRANSPORT\_GO\_0006118 |  | 50 | -0.21 | -0.95 | 0.545 | 0.742 | 1.000 | 1629 | tags=20%, list=12%, signal=23% |
| 233 | APOPTOSIS\_GO |  | 392 | -0.15 | -0.95 | 0.639 | 0.747 | 1.000 | 2873 | tags=26%, list=22%, signal=32% |
| 234 | ACTIN\_FILAMENT\_ORGANIZATION |  | 21 | -0.26 | -0.95 | 0.541 | 0.744 | 1.000 | 2211 | tags=29%, list=17%, signal=34% |
| 235 | VESICLE\_MEDIATED\_TRANSPORT |  | 174 | -0.16 | -0.95 | 0.587 | 0.742 | 1.000 | 3689 | tags=32%, list=28%, signal=44% |
| 236 | CENTRAL\_NERVOUS\_SYSTEM\_DEVELOPMENT |  | 105 | -0.18 | -0.95 | 0.569 | 0.739 | 1.000 | 4229 | tags=39%, list=32%, signal=57% |
| 237 | BIOSYNTHETIC\_PROCESS |  | 402 | -0.14 | -0.95 | 0.653 | 0.739 | 1.000 | 2735 | tags=23%, list=21%, signal=28% |
| 238 | TISSUE\_DEVELOPMENT |  | 126 | -0.17 | -0.94 | 0.603 | 0.750 | 1.000 | 3838 | tags=36%, list=29%, signal=50% |
| 239 | CELL\_CELL\_ADHESION |  | 72 | -0.19 | -0.94 | 0.563 | 0.748 | 1.000 | 4815 | tags=50%, list=37%, signal=79% |
| 240 | PROGRAMMED\_CELL\_DEATH |  | 393 | -0.15 | -0.94 | 0.680 | 0.748 | 1.000 | 2873 | tags=25%, list=22%, signal=32% |
| 241 | REGULATION\_OF\_TRANSCRIPTIONDNA\_DEPENDENT |  | 412 | -0.14 | -0.94 | 0.677 | 0.750 | 1.000 | 2670 | tags=23%, list=20%, signal=28% |
| 242 | DETECTION\_OF\_STIMULUS\_INVOLVED\_IN\_SENSORY\_PERCEPTION |  | 15 | -0.29 | -0.93 | 0.555 | 0.757 | 1.000 | 9360 | tags=100%, list=71%, signal=350% |
| 243 | SECRETION\_BY\_CELL |  | 100 | -0.18 | -0.93 | 0.608 | 0.759 | 1.000 | 4547 | tags=42%, list=35%, signal=64% |
| 244 | REGULATION\_OF\_TRANSLATIONAL\_INITIATION |  | 25 | -0.24 | -0.93 | 0.557 | 0.764 | 1.000 | 1148 | tags=20%, list=9%, signal=22% |
| 245 | AMINO\_ACID\_DERIVATIVE\_METABOLIC\_PROCESS |  | 23 | -0.25 | -0.92 | 0.559 | 0.785 | 1.000 | 2526 | tags=30%, list=19%, signal=38% |
| 246 | RESPONSE\_TO\_NUTRIENT\_LEVELS |  | 27 | -0.23 | -0.92 | 0.572 | 0.789 | 1.000 | 2014 | tags=22%, list=15%, signal=26% |
| 247 | ENDOSOME\_TRANSPORT |  | 22 | -0.25 | -0.91 | 0.573 | 0.790 | 1.000 | 1754 | tags=23%, list=13%, signal=26% |
| 248 | REGULATION\_OF\_RNA\_METABOLIC\_PROCESS |  | 417 | -0.14 | -0.91 | 0.825 | 0.797 | 1.000 | 2670 | tags=23%, list=20%, signal=28% |
| 249 | CELL\_MIGRATION |  | 82 | -0.18 | -0.90 | 0.680 | 0.807 | 1.000 | 2715 | tags=23%, list=21%, signal=29% |
| 250 | REGULATION\_OF\_NUCLEOCYTOPLASMIC\_TRANSPORT |  | 19 | -0.25 | -0.89 | 0.589 | 0.846 | 1.000 | 1261 | tags=21%, list=10%, signal=23% |
| 251 | POSITIVE\_REGULATION\_OF\_TRANSCRIPTION\_FROM\_RNA\_POLYMERASE\_II\_PROMOTER |  | 60 | -0.19 | -0.89 | 0.668 | 0.847 | 1.000 | 3301 | tags=32%, list=25%, signal=42% |
| 252 | REGULATION\_OF\_TRANSLATION |  | 76 | -0.18 | -0.88 | 0.712 | 0.845 | 1.000 | 2667 | tags=25%, list=20%, signal=31% |
| 253 | POTASSIUM\_ION\_TRANSPORT |  | 52 | -0.19 | -0.88 | 0.653 | 0.843 | 1.000 | 4417 | tags=42%, list=34%, signal=64% |
| 254 | PROTEIN\_SECRETION |  | 28 | -0.22 | -0.88 | 0.674 | 0.846 | 1.000 | 4056 | tags=36%, list=31%, signal=52% |
| 255 | AXONOGENESIS |  | 33 | -0.22 | -0.88 | 0.662 | 0.849 | 1.000 | 3025 | tags=27%, list=23%, signal=35% |
| 256 | AMINE\_METABOLIC\_PROCESS |  | 128 | -0.16 | -0.88 | 0.720 | 0.850 | 1.000 | 3478 | tags=30%, list=27%, signal=40% |
| 257 | POSITIVE\_REGULATION\_OF\_HYDROLASE\_ACTIVITY |  | 45 | -0.20 | -0.87 | 0.668 | 0.853 | 1.000 | 1852 | tags=20%, list=14%, signal=23% |
| 258 | NEGATIVE\_REGULATION\_OF\_CELLULAR\_COMPONENT\_ORGANIZATION\_AND\_BIOGENESIS |  | 26 | -0.23 | -0.87 | 0.657 | 0.850 | 1.000 | 1148 | tags=15%, list=9%, signal=17% |
| 259 | AMINO\_ACID\_AND\_DERIVATIVE\_METABOLIC\_PROCESS |  | 96 | -0.16 | -0.86 | 0.755 | 0.868 | 1.000 | 2526 | tags=24%, list=19%, signal=29% |
| 260 | ESTABLISHMENT\_AND\_OR\_MAINTENANCE\_OF\_CELL\_POLARITY |  | 19 | -0.25 | -0.86 | 0.663 | 0.875 | 1.000 | 1158 | tags=16%, list=9%, signal=17% |
| 261 | EPIDERMIS\_DEVELOPMENT |  | 66 | -0.18 | -0.86 | 0.723 | 0.877 | 1.000 | 1426 | tags=18%, list=11%, signal=20% |
| 262 | METAL\_ION\_TRANSPORT |  | 102 | -0.16 | -0.85 | 0.764 | 0.880 | 1.000 | 4617 | tags=42%, list=35%, signal=65% |
| 263 | POSITIVE\_REGULATION\_OF\_JNK\_ACTIVITY |  | 16 | -0.25 | -0.85 | 0.643 | 0.877 | 1.000 | 1410 | tags=25%, list=11%, signal=28% |
| 264 | SECRETORY\_PATHWAY |  | 72 | -0.17 | -0.85 | 0.747 | 0.881 | 1.000 | 4547 | tags=44%, list=35%, signal=68% |
| 265 | SECOND\_MESSENGER\_MEDIATED\_SIGNALING |  | 139 | -0.15 | -0.84 | 0.862 | 0.889 | 1.000 | 1989 | tags=17%, list=15%, signal=20% |
| 266 | CYCLIC\_NUCLEOTIDE\_MEDIATED\_SIGNALING |  | 97 | -0.16 | -0.84 | 0.786 | 0.889 | 1.000 | 1700 | tags=15%, list=13%, signal=18% |
| 267 | POSITIVE\_REGULATION\_OF\_TRANSCRIPTIONDNA\_DEPENDENT |  | 105 | -0.16 | -0.84 | 0.824 | 0.887 | 1.000 | 3635 | tags=30%, list=28%, signal=42% |
| 268 | CELL\_CELL\_SIGNALING |  | 372 | -0.13 | -0.84 | 0.939 | 0.887 | 1.000 | 4448 | tags=38%, list=34%, signal=56% |
| 269 | REGULATION\_OF\_G\_PROTEIN\_COUPLED\_RECEPTOR\_PROTEIN\_SIGNALING\_PATHWAY |  | 23 | -0.23 | -0.84 | 0.729 | 0.887 | 1.000 | 1294 | tags=17%, list=10%, signal=19% |
| 270 | ECTODERM\_DEVELOPMENT |  | 75 | -0.16 | -0.83 | 0.792 | 0.899 | 1.000 | 1426 | tags=17%, list=11%, signal=19% |
| 271 | AROMATIC\_COMPOUND\_METABOLIC\_PROCESS |  | 26 | -0.22 | -0.83 | 0.733 | 0.900 | 1.000 | 1705 | tags=23%, list=13%, signal=26% |
| 272 | RESPONSE\_TO\_EXTRACELLULAR\_STIMULUS |  | 29 | -0.21 | -0.83 | 0.756 | 0.897 | 1.000 | 2014 | tags=21%, list=15%, signal=24% |
| 273 | G\_PROTEIN\_SIGNALING\_COUPLED\_TO\_CYCLIC\_NUCLEOTIDE\_SECOND\_MESSENGER |  | 96 | -0.16 | -0.82 | 0.829 | 0.903 | 1.000 | 1700 | tags=16%, list=13%, signal=18% |
| 274 | PROTEIN\_LOCALIZATION |  | 184 | -0.14 | -0.82 | 0.911 | 0.901 | 1.000 | 3507 | tags=27%, list=27%, signal=36% |
| 275 | PROTEOLYSIS |  | 170 | -0.14 | -0.82 | 0.871 | 0.905 | 1.000 | 2740 | tags=23%, list=21%, signal=29% |
| 276 | G\_PROTEIN\_COUPLED\_RECEPTOR\_PROTEIN\_SIGNALING\_PATHWAY |  | 300 | -0.13 | -0.82 | 0.941 | 0.903 | 1.000 | 3890 | tags=30%, list=30%, signal=42% |
| 277 | SPHINGOLIPID\_METABOLIC\_PROCESS |  | 23 | -0.22 | -0.81 | 0.763 | 0.923 | 1.000 | 3710 | tags=39%, list=28%, signal=55% |
| 278 | POSITIVE\_REGULATION\_OF\_RNA\_METABOLIC\_PROCESS |  | 107 | -0.15 | -0.80 | 0.873 | 0.922 | 1.000 | 3635 | tags=30%, list=28%, signal=41% |
| 279 | G\_PROTEIN\_SIGNALING\_COUPLED\_TO\_IP3\_SECOND\_MESSENGERPHOSPHOLIPASE\_C\_ACTIVATING |  | 39 | -0.19 | -0.80 | 0.806 | 0.919 | 1.000 | 2325 | tags=23%, list=18%, signal=28% |
| 280 | SKELETAL\_MUSCLE\_DEVELOPMENT |  | 28 | -0.20 | -0.80 | 0.776 | 0.916 | 1.000 | 3115 | tags=36%, list=24%, signal=47% |
| 281 | REGULATION\_OF\_INTRACELLULAR\_TRANSPORT |  | 22 | -0.22 | -0.80 | 0.757 | 0.926 | 1.000 | 1261 | tags=18%, list=10%, signal=20% |
| 282 | CELLULAR\_CARBOHYDRATE\_CATABOLIC\_PROCESS |  | 20 | -0.22 | -0.79 | 0.755 | 0.926 | 1.000 | 3740 | tags=35%, list=29%, signal=49% |
| 283 | CARBOHYDRATE\_CATABOLIC\_PROCESS |  | 20 | -0.22 | -0.79 | 0.767 | 0.925 | 1.000 | 3740 | tags=35%, list=29%, signal=49% |
| 284 | SODIUM\_ION\_TRANSPORT |  | 17 | -0.24 | -0.79 | 0.752 | 0.923 | 1.000 | 4952 | tags=53%, list=38%, signal=85% |
| 285 | CARBOHYDRATE\_METABOLIC\_PROCESS |  | 152 | -0.14 | -0.79 | 0.929 | 0.922 | 1.000 | 4296 | tags=36%, list=33%, signal=52% |
| 286 | NUCLEOTIDE\_EXCISION\_REPAIR |  | 19 | -0.22 | -0.79 | 0.798 | 0.927 | 1.000 | 3635 | tags=37%, list=28%, signal=51% |
| 287 | MYOBLAST\_DIFFERENTIATION |  | 16 | -0.24 | -0.78 | 0.763 | 0.924 | 1.000 | 3089 | tags=38%, list=24%, signal=49% |
| 288 | AMINE\_BIOSYNTHETIC\_PROCESS |  | 15 | -0.24 | -0.78 | 0.745 | 0.923 | 1.000 | 234 | tags=13%, list=2%, signal=14% |
| 289 | ACTIVATION\_OF\_PROTEIN\_KINASE\_ACTIVITY |  | 23 | -0.21 | -0.77 | 0.774 | 0.936 | 1.000 | 2189 | tags=17%, list=17%, signal=21% |
| 290 | NEGATIVE\_REGULATION\_OF\_MULTICELLULAR\_ORGANISMAL\_PROCESS |  | 27 | -0.20 | -0.76 | 0.815 | 0.949 | 1.000 | 1860 | tags=22%, list=14%, signal=26% |
| 291 | DETECTION\_OF\_ABIOTIC\_STIMULUS |  | 16 | -0.23 | -0.75 | 0.794 | 0.957 | 1.000 | 4614 | tags=50%, list=35%, signal=77% |
| 292 | AXON\_GUIDANCE |  | 18 | -0.22 | -0.75 | 0.812 | 0.956 | 1.000 | 3939 | tags=39%, list=30%, signal=56% |
| 293 | CATION\_TRANSPORT |  | 130 | -0.14 | -0.75 | 0.954 | 0.954 | 1.000 | 4617 | tags=40%, list=35%, signal=61% |
| 294 | ION\_TRANSPORT |  | 165 | -0.13 | -0.75 | 0.977 | 0.951 | 1.000 | 4016 | tags=33%, list=31%, signal=47% |
| 295 | PHOSPHOINOSITIDE\_MEDIATED\_SIGNALING |  | 42 | -0.17 | -0.74 | 0.865 | 0.957 | 1.000 | 1897 | tags=19%, list=14%, signal=22% |
| 296 | NEGATIVE\_REGULATION\_OF\_CELL\_CYCLE |  | 72 | -0.15 | -0.73 | 0.950 | 0.964 | 1.000 | 2291 | tags=21%, list=18%, signal=25% |
| 297 | SECRETION |  | 157 | -0.13 | -0.73 | 0.979 | 0.961 | 1.000 | 4547 | tags=39%, list=35%, signal=59% |
| 298 | EXTRACELLULAR\_STRUCTURE\_ORGANIZATION\_AND\_BIOGENESIS |  | 23 | -0.20 | -0.73 | 0.843 | 0.961 | 1.000 | 3310 | tags=35%, list=25%, signal=46% |
| 299 | REGULATION\_OF\_CELL\_MIGRATION |  | 23 | -0.20 | -0.71 | 0.877 | 0.974 | 1.000 | 4172 | tags=35%, list=32%, signal=51% |
| 300 | POSITIVE\_REGULATION\_OF\_TRANSPORT |  | 18 | -0.21 | -0.71 | 0.860 | 0.973 | 1.000 | 5110 | tags=56%, list=39%, signal=91% |
| 301 | REGULATION\_OF\_GROWTH |  | 48 | -0.16 | -0.70 | 0.928 | 0.975 | 1.000 | 4500 | tags=38%, list=34%, signal=57% |
| 302 | BRAIN\_DEVELOPMENT |  | 39 | -0.17 | -0.70 | 0.917 | 0.972 | 1.000 | 3945 | tags=36%, list=30%, signal=51% |
| 303 | PROTEIN\_POLYMERIZATION |  | 17 | -0.21 | -0.70 | 0.873 | 0.974 | 1.000 | 2035 | tags=18%, list=16%, signal=21% |
| 304 | REGULATION\_OF\_CYTOKINE\_PRODUCTION |  | 21 | -0.19 | -0.68 | 0.901 | 0.986 | 1.000 | 3729 | tags=33%, list=28%, signal=47% |
| 305 | REGULATION\_OF\_MUSCLE\_CONTRACTION |  | 18 | -0.20 | -0.67 | 0.913 | 0.986 | 1.000 | 3246 | tags=39%, list=25%, signal=52% |
| 306 | STRESS\_ACTIVATED\_PROTEIN\_KINASE\_SIGNALING\_PATHWAY |  | 45 | -0.15 | -0.67 | 0.943 | 0.987 | 1.000 | 1410 | tags=16%, list=11%, signal=17% |
| 307 | DI\_\_\_TRI\_VALENT\_INORGANIC\_CATION\_TRANSPORT |  | 27 | -0.17 | -0.66 | 0.930 | 0.985 | 1.000 | 2850 | tags=26%, list=22%, signal=33% |
| 308 | G\_PROTEIN\_SIGNALING\_ADENYLATE\_CYCLASE\_ACTIVATING\_PATHWAY |  | 24 | -0.17 | -0.66 | 0.911 | 0.982 | 1.000 | 1379 | tags=13%, list=11%, signal=14% |
| 309 | INSULIN\_RECEPTOR\_SIGNALING\_PATHWAY |  | 16 | -0.20 | -0.66 | 0.916 | 0.981 | 1.000 | 1894 | tags=19%, list=14%, signal=22% |
| 310 | INORGANIC\_ANION\_TRANSPORT |  | 16 | -0.20 | -0.66 | 0.910 | 0.979 | 1.000 | 3262 | tags=31%, list=25%, signal=42% |
| 311 | RESPONSE\_TO\_LIGHT\_STIMULUS |  | 40 | -0.15 | -0.65 | 0.952 | 0.977 | 1.000 | 3692 | tags=33%, list=28%, signal=45% |
| 312 | NEGATIVE\_REGULATION\_OF\_GROWTH |  | 35 | -0.16 | -0.65 | 0.944 | 0.977 | 1.000 | 4113 | tags=34%, list=31%, signal=50% |
| 313 | CALCIUM\_ION\_TRANSPORT |  | 23 | -0.17 | -0.63 | 0.911 | 0.980 | 1.000 | 2850 | tags=26%, list=22%, signal=33% |
| 314 | MONOVALENT\_INORGANIC\_CATION\_TRANSPORT |  | 83 | -0.12 | -0.61 | 0.993 | 0.988 | 1.000 | 4551 | tags=39%, list=35%, signal=59% |
| 315 | EXCRETION |  | 35 | -0.14 | -0.59 | 0.978 | 0.990 | 1.000 | 3928 | tags=34%, list=30%, signal=49% |
| 316 | ADENYLATE\_CYCLASE\_ACTIVATION |  | 18 | -0.16 | -0.54 | 0.982 | 0.999 | 1.000 | 11062 | tags=100%, list=85%, signal=644% |
| 317 | LIPID\_HOMEOSTASIS |  | 15 | -0.16 | -0.53 | 0.982 | 0.996 | 1.000 | 3851 | tags=33%, list=29%, signal=47% |
| 318 | PEROXISOME\_ORGANIZATION\_AND\_BIOGENESIS |  | 15 | -0.15 | -0.48 | 0.994 | 0.998 | 1.000 | 4099 | tags=33%, list=31%, signal=48% |
Table: Gene sets enriched in phenotype **na**[plain text format]****

  
