# Supplementary material for: Multiple-input multiple-output causal strategies for gene selection
Source: BMC Bioinformatics. 2011 Nov 25;12:458. doi: 10.1186/1471-2105-12-458 (PMC3323860; doi:10.1186/1471-2105-12-458)
Supplement: Additional file 3 — Archive containing the output files computed by the preranked GSEA for λ ∈ {0.6,0.7,0.8,0.9,1.0,2.0} (GSEA_MIMO_part2.zip). [file 1471-2105-12-458-S3.ZIP › mFS20_entrez_mimo.GseaPreranked.1316039690234/gsea_report_for_na_pos_1316039690234.html]

Report for na\_pos 1316039690234 [GSEA]

| GS  follow link to MSigDB | GS DETAILS | SIZE | ES | NES | NOM p-val | FDR q-val | FWER p-val | RANK AT MAX | LEADING EDGE || 1 | MITOTIC\_CELL\_CYCLE |  | 134 | 0.48 | 2.56 | 0.000 | 0.000 | 0.000 | 3071 | tags=52%, list=23%, signal=68% |
| 2 | CELL\_CYCLE\_PROCESS |  | 169 | 0.46 | 2.54 | 0.000 | 0.000 | 0.000 | 3071 | tags=51%, list=23%, signal=66% |
| 3 | M\_PHASE\_OF\_MITOTIC\_CELL\_CYCLE |  | 72 | 0.54 | 2.54 | 0.000 | 0.000 | 0.000 | 2779 | tags=57%, list=21%, signal=72% |
| 4 | MITOSIS |  | 70 | 0.53 | 2.53 | 0.000 | 0.000 | 0.000 | 1527 | tags=46%, list=12%, signal=51% |
| 5 | M\_PHASE |  | 98 | 0.49 | 2.46 | 0.000 | 0.000 | 0.001 | 2779 | tags=52%, list=21%, signal=66% |
| 6 | CELL\_CYCLE\_PHASE |  | 152 | 0.44 | 2.40 | 0.000 | 0.000 | 0.001 | 3071 | tags=49%, list=23%, signal=64% |
| 7 | REGULATION\_OF\_MITOSIS |  | 33 | 0.58 | 2.31 | 0.000 | 0.000 | 0.001 | 798 | tags=45%, list=6%, signal=48% |
| 8 | CELL\_CYCLE\_GO\_0007049 |  | 277 | 0.38 | 2.26 | 0.000 | 0.000 | 0.005 | 1898 | tags=35%, list=14%, signal=40% |
| 9 | DNA\_METABOLIC\_PROCESS |  | 240 | 0.38 | 2.25 | 0.000 | 0.001 | 0.006 | 3182 | tags=44%, list=24%, signal=57% |
| 10 | DNA\_REPLICATION |  | 97 | 0.44 | 2.24 | 0.000 | 0.000 | 0.006 | 3143 | tags=46%, list=24%, signal=61% |
| 11 | SISTER\_CHROMATID\_SEGREGATION |  | 16 | 0.69 | 2.24 | 0.000 | 0.000 | 0.006 | 1418 | tags=56%, list=11%, signal=63% |
| 12 | MITOTIC\_SISTER\_CHROMATID\_SEGREGATION |  | 15 | 0.71 | 2.23 | 0.000 | 0.000 | 0.006 | 1418 | tags=60%, list=11%, signal=67% |
| 13 | CHROMOSOME\_SEGREGATION |  | 28 | 0.57 | 2.20 | 0.000 | 0.001 | 0.010 | 3852 | tags=75%, list=29%, signal=106% |
| 14 | DNA\_DEPENDENT\_DNA\_REPLICATION |  | 52 | 0.49 | 2.19 | 0.000 | 0.001 | 0.010 | 2746 | tags=50%, list=21%, signal=63% |
| 15 | DNA\_REPAIR |  | 118 | 0.41 | 2.13 | 0.000 | 0.001 | 0.025 | 2963 | tags=45%, list=23%, signal=58% |
| 16 | CELL\_CYCLE\_CHECKPOINT\_GO\_0000075 |  | 45 | 0.49 | 2.13 | 0.000 | 0.001 | 0.025 | 2282 | tags=51%, list=17%, signal=62% |
| 17 | DNA\_REPLICATION\_INITIATION |  | 15 | 0.66 | 2.08 | 0.000 | 0.002 | 0.053 | 2572 | tags=80%, list=20%, signal=99% |
| 18 | RNA\_SPLICING |  | 74 | 0.43 | 2.08 | 0.000 | 0.002 | 0.056 | 3189 | tags=47%, list=24%, signal=62% |
| 19 | MICROTUBULE\_CYTOSKELETON\_ORGANIZATION\_AND\_BIOGENESIS |  | 31 | 0.53 | 2.06 | 0.000 | 0.002 | 0.061 | 2779 | tags=55%, list=21%, signal=69% |
| 20 | RESPONSE\_TO\_DNA\_DAMAGE\_STIMULUS |  | 153 | 0.37 | 2.04 | 0.000 | 0.003 | 0.089 | 2963 | tags=42%, list=23%, signal=54% |
| 21 | RNA\_PROCESSING |  | 138 | 0.38 | 2.01 | 0.000 | 0.004 | 0.120 | 3189 | tags=45%, list=24%, signal=59% |
| 22 | RESPONSE\_TO\_ENDOGENOUS\_STIMULUS |  | 182 | 0.36 | 2.00 | 0.000 | 0.005 | 0.126 | 3650 | tags=46%, list=28%, signal=63% |
| 23 | MITOCHONDRION\_ORGANIZATION\_AND\_BIOGENESIS |  | 42 | 0.46 | 1.98 | 0.000 | 0.006 | 0.162 | 2377 | tags=40%, list=18%, signal=49% |
| 24 | MITOTIC\_CELL\_CYCLE\_CHECKPOINT |  | 19 | 0.57 | 1.97 | 0.005 | 0.006 | 0.172 | 577 | tags=42%, list=4%, signal=44% |
| 25 | REGULATION\_OF\_CYCLIN\_DEPENDENT\_PROTEIN\_KINASE\_ACTIVITY |  | 40 | 0.46 | 1.94 | 0.002 | 0.008 | 0.220 | 1789 | tags=43%, list=14%, signal=49% |
| 26 | TRANSCRIPTION\_INITIATION\_FROM\_RNA\_POLYMERASE\_II\_PROMOTER |  | 27 | 0.52 | 1.94 | 0.002 | 0.008 | 0.226 | 1922 | tags=44%, list=15%, signal=52% |
| 27 | REGULATION\_OF\_CELL\_CYCLE |  | 161 | 0.35 | 1.92 | 0.000 | 0.010 | 0.280 | 1789 | tags=32%, list=14%, signal=37% |
| 28 | PROTEIN\_FOLDING |  | 55 | 0.42 | 1.91 | 0.000 | 0.010 | 0.308 | 3177 | tags=49%, list=24%, signal=65% |
| 29 | COENZYME\_METABOLIC\_PROCESS |  | 35 | 0.47 | 1.89 | 0.002 | 0.012 | 0.366 | 3337 | tags=49%, list=25%, signal=65% |
| 30 | MRNA\_METABOLIC\_PROCESS |  | 72 | 0.39 | 1.88 | 0.000 | 0.013 | 0.392 | 2957 | tags=44%, list=23%, signal=57% |
| 31 | NUCLEOTIDE\_BIOSYNTHETIC\_PROCESS |  | 17 | 0.57 | 1.88 | 0.004 | 0.013 | 0.401 | 1390 | tags=47%, list=11%, signal=53% |
| 32 | DOUBLE\_STRAND\_BREAK\_REPAIR |  | 21 | 0.53 | 1.86 | 0.008 | 0.015 | 0.463 | 1884 | tags=48%, list=14%, signal=56% |
| 33 | NUCLEOBASENUCLEOSIDENUCLEOTIDE\_AND\_NUCLEIC\_ACID\_TRANSPORT |  | 26 | 0.48 | 1.85 | 0.006 | 0.016 | 0.490 | 2975 | tags=50%, list=23%, signal=65% |
| 34 | INTERPHASE\_OF\_MITOTIC\_CELL\_CYCLE |  | 57 | 0.39 | 1.80 | 0.007 | 0.021 | 0.613 | 4095 | tags=56%, list=31%, signal=81% |
| 35 | MITOCHONDRIAL\_TRANSPORT |  | 18 | 0.54 | 1.79 | 0.005 | 0.023 | 0.643 | 1737 | tags=44%, list=13%, signal=51% |
| 36 | REGULATION\_OF\_MITOTIC\_CELL\_CYCLE |  | 19 | 0.52 | 1.79 | 0.009 | 0.022 | 0.645 | 2188 | tags=47%, list=17%, signal=57% |
| 37 | MRNA\_PROCESSING\_GO\_0006397 |  | 61 | 0.39 | 1.79 | 0.002 | 0.022 | 0.652 | 2506 | tags=39%, list=19%, signal=48% |
| 38 | COFACTOR\_METABOLIC\_PROCESS |  | 51 | 0.40 | 1.78 | 0.002 | 0.023 | 0.678 | 3337 | tags=43%, list=25%, signal=58% |
| 39 | TRNA\_METABOLIC\_PROCESS |  | 15 | 0.56 | 1.77 | 0.013 | 0.024 | 0.704 | 2918 | tags=67%, list=22%, signal=86% |
| 40 | INTERPHASE |  | 63 | 0.38 | 1.77 | 0.000 | 0.024 | 0.712 | 4095 | tags=54%, list=31%, signal=78% |
| 41 | REGULATION\_OF\_DNA\_REPLICATION |  | 18 | 0.52 | 1.75 | 0.015 | 0.027 | 0.755 | 1574 | tags=44%, list=12%, signal=50% |
| 42 | DNA\_INTEGRITY\_CHECKPOINT |  | 22 | 0.49 | 1.74 | 0.006 | 0.028 | 0.779 | 1975 | tags=50%, list=15%, signal=59% |
| 43 | G1\_S\_TRANSITION\_OF\_MITOTIC\_CELL\_CYCLE |  | 23 | 0.47 | 1.74 | 0.004 | 0.028 | 0.784 | 2940 | tags=48%, list=22%, signal=62% |
| 44 | CYTOKINESIS |  | 17 | 0.52 | 1.74 | 0.007 | 0.028 | 0.797 | 2542 | tags=47%, list=19%, signal=58% |
| 45 | REGULATION\_OF\_DNA\_METABOLIC\_PROCESS |  | 40 | 0.41 | 1.72 | 0.002 | 0.032 | 0.848 | 2617 | tags=48%, list=20%, signal=59% |
| 46 | NUCLEAR\_EXPORT |  | 26 | 0.46 | 1.71 | 0.007 | 0.033 | 0.865 | 2975 | tags=42%, list=23%, signal=55% |
| 47 | TRANSCRIPTION\_INITIATION |  | 33 | 0.43 | 1.70 | 0.008 | 0.036 | 0.891 | 1922 | tags=39%, list=15%, signal=46% |
| 48 | PROTEIN\_MODIFICATION\_BY\_SMALL\_PROTEIN\_CONJUGATION |  | 35 | 0.41 | 1.68 | 0.009 | 0.042 | 0.928 | 1799 | tags=34%, list=14%, signal=40% |
| 49 | PROTEIN\_CATABOLIC\_PROCESS |  | 60 | 0.37 | 1.67 | 0.009 | 0.043 | 0.937 | 3499 | tags=42%, list=27%, signal=57% |
| 50 | CHROMOSOME\_ORGANIZATION\_AND\_BIOGENESIS |  | 107 | 0.32 | 1.67 | 0.004 | 0.043 | 0.944 | 2692 | tags=36%, list=21%, signal=44% |
| 51 | CELLULAR\_PROTEIN\_CATABOLIC\_PROCESS |  | 50 | 0.38 | 1.66 | 0.011 | 0.045 | 0.953 | 3499 | tags=44%, list=27%, signal=60% |
| 52 | BIOPOLYMER\_CATABOLIC\_PROCESS |  | 103 | 0.32 | 1.65 | 0.002 | 0.049 | 0.962 | 3171 | tags=38%, list=24%, signal=50% |
| 53 | CELL\_DIVISION |  | 19 | 0.48 | 1.64 | 0.020 | 0.052 | 0.972 | 904 | tags=32%, list=7%, signal=34% |
| 54 | RNA\_EXPORT\_FROM\_NUCLEUS |  | 17 | 0.49 | 1.63 | 0.028 | 0.052 | 0.974 | 5095 | tags=76%, list=39%, signal=125% |
| 55 | APOPTOTIC\_NUCLEAR\_CHANGES |  | 17 | 0.49 | 1.63 | 0.024 | 0.051 | 0.974 | 1198 | tags=35%, list=9%, signal=39% |
| 56 | PROTEIN\_UBIQUITINATION |  | 32 | 0.40 | 1.62 | 0.014 | 0.055 | 0.985 | 1621 | tags=31%, list=12%, signal=36% |
| 57 | COFACTOR\_BIOSYNTHETIC\_PROCESS |  | 21 | 0.44 | 1.57 | 0.036 | 0.079 | 0.998 | 1737 | tags=33%, list=13%, signal=38% |
| 58 | MACROMOLECULE\_CATABOLIC\_PROCESS |  | 120 | 0.29 | 1.56 | 0.005 | 0.083 | 0.999 | 3317 | tags=36%, list=25%, signal=48% |
| 59 | PROTEIN\_DNA\_COMPLEX\_ASSEMBLY |  | 45 | 0.36 | 1.55 | 0.018 | 0.086 | 0.999 | 2077 | tags=33%, list=16%, signal=39% |
| 60 | BASE\_EXCISION\_REPAIR |  | 16 | 0.46 | 1.52 | 0.050 | 0.104 | 1.000 | 2288 | tags=38%, list=17%, signal=45% |
| 61 | UBIQUITIN\_CYCLE |  | 40 | 0.36 | 1.51 | 0.028 | 0.108 | 1.000 | 2529 | tags=35%, list=19%, signal=43% |
| 62 | DNA\_DAMAGE\_CHECKPOINT |  | 19 | 0.44 | 1.49 | 0.052 | 0.125 | 1.000 | 3842 | tags=63%, list=29%, signal=89% |
| 63 | CELLULAR\_MACROMOLECULE\_CATABOLIC\_PROCESS |  | 90 | 0.29 | 1.48 | 0.028 | 0.131 | 1.000 | 3560 | tags=39%, list=27%, signal=53% |
| 64 | DNA\_DAMAGE\_RESPONSESIGNAL\_TRANSDUCTION |  | 34 | 0.38 | 1.47 | 0.053 | 0.131 | 1.000 | 1975 | tags=38%, list=15%, signal=45% |
| 65 | NUCLEOTIDE\_METABOLIC\_PROCESS |  | 36 | 0.36 | 1.46 | 0.047 | 0.137 | 1.000 | 2549 | tags=39%, list=19%, signal=48% |
| 66 | MEIOTIC\_CELL\_CYCLE |  | 31 | 0.38 | 1.45 | 0.050 | 0.149 | 1.000 | 3050 | tags=45%, list=23%, signal=59% |
| 67 | MICROTUBULE\_BASED\_PROCESS |  | 75 | 0.30 | 1.44 | 0.028 | 0.159 | 1.000 | 2779 | tags=33%, list=21%, signal=42% |
| 68 | ESTABLISHMENT\_OF\_ORGANELLE\_LOCALIZATION |  | 16 | 0.45 | 1.44 | 0.069 | 0.157 | 1.000 | 881 | tags=38%, list=7%, signal=40% |
| 69 | DNA\_PACKAGING |  | 29 | 0.37 | 1.43 | 0.072 | 0.158 | 1.000 | 2077 | tags=34%, list=16%, signal=41% |
| 70 | NUCLEAR\_TRANSPORT |  | 77 | 0.29 | 1.43 | 0.032 | 0.156 | 1.000 | 3628 | tags=36%, list=28%, signal=50% |
| 71 | NEGATIVE\_REGULATION\_OF\_DNA\_METABOLIC\_PROCESS |  | 16 | 0.44 | 1.42 | 0.082 | 0.164 | 1.000 | 1574 | tags=44%, list=12%, signal=50% |
| 72 | ORGANELLE\_ORGANIZATION\_AND\_BIOGENESIS |  | 407 | 0.23 | 1.42 | 0.003 | 0.169 | 1.000 | 2918 | tags=29%, list=22%, signal=36% |
| 73 | ALCOHOL\_METABOLIC\_PROCESS |  | 82 | 0.29 | 1.42 | 0.049 | 0.168 | 1.000 | 3778 | tags=39%, list=29%, signal=55% |
| 74 | NUCLEOCYTOPLASMIC\_TRANSPORT |  | 77 | 0.29 | 1.41 | 0.040 | 0.168 | 1.000 | 3628 | tags=36%, list=28%, signal=50% |
| 75 | DNA\_RECOMBINATION |  | 45 | 0.33 | 1.41 | 0.068 | 0.172 | 1.000 | 1000 | tags=27%, list=8%, signal=29% |
| 76 | G1\_PHASE |  | 15 | 0.43 | 1.40 | 0.084 | 0.173 | 1.000 | 3071 | tags=53%, list=23%, signal=70% |
| 77 | NUCLEOBASENUCLEOSIDE\_AND\_NUCLEOTIDE\_METABOLIC\_PROCESS |  | 46 | 0.33 | 1.40 | 0.058 | 0.172 | 1.000 | 2549 | tags=35%, list=19%, signal=43% |
| 78 | VIRAL\_INFECTIOUS\_CYCLE |  | 29 | 0.36 | 1.40 | 0.075 | 0.176 | 1.000 | 1718 | tags=34%, list=13%, signal=40% |
| 79 | ORGANELLE\_LOCALIZATION |  | 21 | 0.39 | 1.40 | 0.093 | 0.175 | 1.000 | 881 | tags=29%, list=7%, signal=31% |
| 80 | CELLULAR\_COMPONENT\_DISASSEMBLY |  | 31 | 0.36 | 1.39 | 0.075 | 0.180 | 1.000 | 3175 | tags=39%, list=24%, signal=51% |
| 81 | VIRAL\_GENOME\_REPLICATION |  | 20 | 0.40 | 1.39 | 0.075 | 0.181 | 1.000 | 1523 | tags=35%, list=12%, signal=40% |
| 82 | RESPONSE\_TO\_HYPOXIA |  | 27 | 0.36 | 1.38 | 0.098 | 0.184 | 1.000 | 1309 | tags=26%, list=10%, signal=29% |
| 83 | ONE\_CARBON\_COMPOUND\_METABOLIC\_PROCESS |  | 24 | 0.38 | 1.38 | 0.071 | 0.185 | 1.000 | 3820 | tags=54%, list=29%, signal=76% |
| 84 | NEGATIVE\_REGULATION\_OF\_BINDING |  | 16 | 0.40 | 1.31 | 0.143 | 0.272 | 1.000 | 2253 | tags=44%, list=17%, signal=53% |
| 85 | VIRAL\_REPRODUCTION |  | 38 | 0.31 | 1.30 | 0.125 | 0.290 | 1.000 | 1886 | tags=32%, list=14%, signal=37% |
| 86 | APOPTOTIC\_PROGRAM |  | 56 | 0.29 | 1.30 | 0.102 | 0.290 | 1.000 | 2917 | tags=34%, list=22%, signal=43% |
| 87 | VIRAL\_REPRODUCTIVE\_PROCESS |  | 33 | 0.33 | 1.29 | 0.143 | 0.294 | 1.000 | 1886 | tags=33%, list=14%, signal=39% |
| 88 | CELL\_STRUCTURE\_DISASSEMBLY\_DURING\_APOPTOSIS |  | 17 | 0.38 | 1.26 | 0.171 | 0.341 | 1.000 | 1198 | tags=24%, list=9%, signal=26% |
| 89 | RESPONSE\_TO\_HORMONE\_STIMULUS |  | 26 | 0.33 | 1.26 | 0.167 | 0.346 | 1.000 | 4972 | tags=58%, list=38%, signal=93% |
| 90 | CYTOSKELETON\_DEPENDENT\_INTRACELLULAR\_TRANSPORT |  | 25 | 0.34 | 1.26 | 0.163 | 0.344 | 1.000 | 2728 | tags=36%, list=21%, signal=45% |
| 91 | TRANSCRIPTION\_FROM\_RNA\_POLYMERASE\_III\_PROMOTER |  | 18 | 0.38 | 1.25 | 0.189 | 0.346 | 1.000 | 4384 | tags=72%, list=33%, signal=108% |
| 92 | RESPONSE\_TO\_ORGANIC\_SUBSTANCE |  | 27 | 0.33 | 1.25 | 0.181 | 0.344 | 1.000 | 2458 | tags=33%, list=19%, signal=41% |
| 93 | REGULATION\_OF\_HYDROLASE\_ACTIVITY |  | 65 | 0.26 | 1.24 | 0.146 | 0.372 | 1.000 | 2573 | tags=29%, list=20%, signal=36% |
| 94 | RNA\_CATABOLIC\_PROCESS |  | 20 | 0.36 | 1.24 | 0.202 | 0.369 | 1.000 | 2720 | tags=45%, list=21%, signal=57% |
| 95 | NUCLEAR\_ORGANIZATION\_AND\_BIOGENESIS |  | 23 | 0.35 | 1.23 | 0.185 | 0.368 | 1.000 | 1198 | tags=26%, list=9%, signal=29% |
| 96 | MEIOSIS\_I |  | 19 | 0.36 | 1.23 | 0.207 | 0.367 | 1.000 | 3477 | tags=47%, list=27%, signal=64% |
| 97 | REGULATION\_OF\_PROTEIN\_KINASE\_ACTIVITY |  | 133 | 0.23 | 1.21 | 0.115 | 0.419 | 1.000 | 1457 | tags=20%, list=11%, signal=22% |
| 98 | MORPHOGENESIS\_OF\_AN\_EPITHELIUM |  | 15 | 0.38 | 1.21 | 0.229 | 0.415 | 1.000 | 3777 | tags=53%, list=29%, signal=75% |
| 99 | NEGATIVE\_REGULATION\_OF\_CATALYTIC\_ACTIVITY |  | 61 | 0.26 | 1.21 | 0.163 | 0.412 | 1.000 | 2676 | tags=33%, list=20%, signal=41% |
| 100 | INTRACELLULAR\_TRANSPORT |  | 248 | 0.20 | 1.19 | 0.129 | 0.449 | 1.000 | 3309 | tags=29%, list=25%, signal=39% |
| 101 | ESTABLISHMENT\_OF\_CELLULAR\_LOCALIZATION |  | 311 | 0.20 | 1.19 | 0.126 | 0.445 | 1.000 | 3000 | tags=26%, list=23%, signal=33% |
| 102 | REGULATION\_OF\_KINASE\_ACTIVITY |  | 135 | 0.22 | 1.18 | 0.156 | 0.444 | 1.000 | 1457 | tags=19%, list=11%, signal=21% |
| 103 | REGULATION\_OF\_TRANSFERASE\_ACTIVITY |  | 137 | 0.22 | 1.18 | 0.149 | 0.447 | 1.000 | 1457 | tags=19%, list=11%, signal=21% |
| 104 | TRANSCRIPTION\_FROM\_RNA\_POLYMERASE\_II\_PROMOTER |  | 428 | 0.19 | 1.18 | 0.074 | 0.443 | 1.000 | 3033 | tags=27%, list=23%, signal=34% |
| 105 | CELLULAR\_RESPONSE\_TO\_STIMULUS |  | 17 | 0.35 | 1.16 | 0.265 | 0.477 | 1.000 | 2202 | tags=29%, list=17%, signal=35% |
| 106 | CATABOLIC\_PROCESS |  | 201 | 0.20 | 1.15 | 0.170 | 0.499 | 1.000 | 3317 | tags=30%, list=25%, signal=39% |
| 107 | CELLULAR\_LOCALIZATION |  | 323 | 0.19 | 1.15 | 0.123 | 0.496 | 1.000 | 3000 | tags=26%, list=23%, signal=33% |
| 108 | DNA\_CATABOLIC\_PROCESS |  | 21 | 0.33 | 1.15 | 0.267 | 0.495 | 1.000 | 2167 | tags=29%, list=17%, signal=34% |
| 109 | OXYGEN\_AND\_REACTIVE\_OXYGEN\_SPECIES\_METABOLIC\_PROCESS |  | 18 | 0.34 | 1.15 | 0.255 | 0.492 | 1.000 | 1198 | tags=28%, list=9%, signal=31% |
| 110 | RESPONSE\_TO\_STRESS |  | 467 | 0.18 | 1.15 | 0.128 | 0.489 | 1.000 | 3019 | tags=27%, list=23%, signal=34% |
| 111 | NEGATIVE\_REGULATION\_OF\_DNA\_BINDING |  | 15 | 0.36 | 1.14 | 0.301 | 0.503 | 1.000 | 2253 | tags=40%, list=17%, signal=48% |
| 112 | HETEROCYCLE\_METABOLIC\_PROCESS |  | 26 | 0.31 | 1.14 | 0.266 | 0.500 | 1.000 | 1737 | tags=23%, list=13%, signal=27% |
| 113 | RIBONUCLEOPROTEIN\_COMPLEX\_BIOGENESIS\_AND\_ASSEMBLY |  | 68 | 0.24 | 1.14 | 0.231 | 0.506 | 1.000 | 3189 | tags=34%, list=24%, signal=44% |
| 114 | GENERATION\_OF\_A\_SIGNAL\_INVOLVED\_IN\_CELL\_CELL\_SIGNALING |  | 25 | 0.31 | 1.13 | 0.268 | 0.506 | 1.000 | 2986 | tags=36%, list=23%, signal=47% |
| 115 | CELLULAR\_CATABOLIC\_PROCESS |  | 189 | 0.20 | 1.13 | 0.198 | 0.514 | 1.000 | 3317 | tags=30%, list=25%, signal=39% |
| 116 | REGULATION\_OF\_CATALYTIC\_ACTIVITY |  | 238 | 0.19 | 1.11 | 0.197 | 0.553 | 1.000 | 2676 | tags=24%, list=20%, signal=30% |
| 117 | GLUTAMATE\_SIGNALING\_PATHWAY |  | 17 | 0.33 | 1.11 | 0.301 | 0.551 | 1.000 | 4418 | tags=47%, list=34%, signal=71% |
| 118 | CELLULAR\_RESPIRATION |  | 19 | 0.32 | 1.11 | 0.312 | 0.555 | 1.000 | 2778 | tags=37%, list=21%, signal=47% |
| 119 | CELL\_PROJECTION\_BIOGENESIS |  | 20 | 0.32 | 1.10 | 0.311 | 0.563 | 1.000 | 4302 | tags=50%, list=33%, signal=74% |
| 120 | ESTABLISHMENT\_AND\_OR\_MAINTENANCE\_OF\_CHROMATIN\_ARCHITECTURE |  | 65 | 0.24 | 1.09 | 0.309 | 0.578 | 1.000 | 2692 | tags=29%, list=21%, signal=37% |
| 121 | PROTEIN\_AMINO\_ACID\_O\_LINKED\_GLYCOSYLATION |  | 18 | 0.32 | 1.09 | 0.323 | 0.577 | 1.000 | 1315 | tags=28%, list=10%, signal=31% |
| 122 | RESPONSE\_TO\_ABIOTIC\_STIMULUS |  | 79 | 0.22 | 1.09 | 0.290 | 0.593 | 1.000 | 4629 | tags=49%, list=35%, signal=76% |
| 123 | CYTOSKELETON\_ORGANIZATION\_AND\_BIOGENESIS |  | 182 | 0.19 | 1.08 | 0.279 | 0.590 | 1.000 | 2869 | tags=27%, list=22%, signal=34% |
| 124 | EMBRYONIC\_DEVELOPMENT |  | 46 | 0.25 | 1.08 | 0.334 | 0.605 | 1.000 | 1984 | tags=22%, list=15%, signal=26% |
| 125 | LIPID\_BIOSYNTHETIC\_PROCESS |  | 84 | 0.22 | 1.07 | 0.339 | 0.609 | 1.000 | 1420 | tags=19%, list=11%, signal=21% |
| 126 | CELLULAR\_BIOSYNTHETIC\_PROCESS |  | 273 | 0.18 | 1.07 | 0.283 | 0.605 | 1.000 | 3459 | tags=31%, list=26%, signal=41% |
| 127 | REGULATION\_OF\_MOLECULAR\_FUNCTION |  | 275 | 0.18 | 1.07 | 0.264 | 0.603 | 1.000 | 2676 | tags=24%, list=20%, signal=30% |
| 128 | MICROTUBULE\_BASED\_MOVEMENT |  | 16 | 0.33 | 1.07 | 0.371 | 0.603 | 1.000 | 2728 | tags=38%, list=21%, signal=47% |
| 129 | INDUCTION\_OF\_APOPTOSIS\_BY\_EXTRACELLULAR\_SIGNALS |  | 25 | 0.29 | 1.06 | 0.375 | 0.626 | 1.000 | 2770 | tags=36%, list=21%, signal=46% |
| 130 | NEGATIVE\_REGULATION\_OF\_APOPTOSIS |  | 136 | 0.20 | 1.06 | 0.333 | 0.621 | 1.000 | 1504 | tags=19%, list=11%, signal=21% |
| 131 | STEROID\_BIOSYNTHETIC\_PROCESS |  | 22 | 0.30 | 1.06 | 0.379 | 0.632 | 1.000 | 1220 | tags=27%, list=9%, signal=30% |
| 132 | CARBOHYDRATE\_TRANSPORT |  | 17 | 0.32 | 1.05 | 0.385 | 0.638 | 1.000 | 2093 | tags=29%, list=16%, signal=35% |
| 133 | CHROMATIN\_REMODELING |  | 21 | 0.30 | 1.05 | 0.390 | 0.645 | 1.000 | 3175 | tags=38%, list=24%, signal=50% |
| 134 | STEROID\_METABOLIC\_PROCESS |  | 66 | 0.23 | 1.04 | 0.378 | 0.648 | 1.000 | 3716 | tags=38%, list=28%, signal=53% |
| 135 | NEGATIVE\_REGULATION\_OF\_PROGRAMMED\_CELL\_DEATH |  | 137 | 0.20 | 1.04 | 0.387 | 0.645 | 1.000 | 1504 | tags=19%, list=11%, signal=21% |
| 136 | NITROGEN\_COMPOUND\_BIOSYNTHETIC\_PROCESS |  | 25 | 0.28 | 1.04 | 0.407 | 0.642 | 1.000 | 1706 | tags=24%, list=13%, signal=28% |
| 137 | RNA\_SPLICINGVIA\_TRANSESTERIFICATION\_REACTIONS |  | 27 | 0.27 | 1.03 | 0.404 | 0.682 | 1.000 | 2609 | tags=30%, list=20%, signal=37% |
| 138 | CHROMATIN\_ASSEMBLY\_OR\_DISASSEMBLY |  | 25 | 0.27 | 1.02 | 0.425 | 0.695 | 1.000 | 3964 | tags=48%, list=30%, signal=69% |
| 139 | CHROMATIN\_MODIFICATION |  | 46 | 0.23 | 1.01 | 0.417 | 0.706 | 1.000 | 2692 | tags=28%, list=21%, signal=35% |
| 140 | REGULATION\_OF\_PROTEIN\_STABILITY |  | 17 | 0.31 | 1.01 | 0.437 | 0.705 | 1.000 | 3974 | tags=41%, list=30%, signal=59% |
| 141 | STEROID\_HORMONE\_RECEPTOR\_SIGNALING\_PATHWAY |  | 18 | 0.30 | 1.01 | 0.446 | 0.723 | 1.000 | 1065 | tags=22%, list=8%, signal=24% |
| 142 | POSITIVE\_REGULATION\_OF\_CELL\_CYCLE |  | 15 | 0.32 | 1.00 | 0.477 | 0.739 | 1.000 | 1244 | tags=27%, list=10%, signal=29% |
| 143 | INTRACELLULAR\_RECEPTOR\_MEDIATED\_SIGNALING\_PATHWAY |  | 18 | 0.30 | 0.99 | 0.470 | 0.742 | 1.000 | 1065 | tags=22%, list=8%, signal=24% |
| 144 | NEGATIVE\_REGULATION\_OF\_TRANSFERASE\_ACTIVITY |  | 27 | 0.26 | 0.99 | 0.482 | 0.739 | 1.000 | 1457 | tags=22%, list=11%, signal=25% |
| 145 | COVALENT\_CHROMATIN\_MODIFICATION |  | 22 | 0.28 | 0.99 | 0.452 | 0.739 | 1.000 | 1350 | tags=23%, list=10%, signal=25% |
| 146 | REGULATION\_OF\_TRANSCRIPTION\_FROM\_RNA\_POLYMERASE\_II\_PROMOTER |  | 267 | 0.16 | 0.99 | 0.474 | 0.741 | 1.000 | 3120 | tags=26%, list=24%, signal=34% |
| 147 | INDUCTION\_OF\_APOPTOSIS\_BY\_INTRACELLULAR\_SIGNALS |  | 23 | 0.27 | 0.98 | 0.509 | 0.754 | 1.000 | 2940 | tags=35%, list=22%, signal=45% |
| 148 | GAMETE\_GENERATION |  | 92 | 0.20 | 0.98 | 0.502 | 0.769 | 1.000 | 3762 | tags=35%, list=29%, signal=48% |
| 149 | MEIOTIC\_RECOMBINATION |  | 16 | 0.30 | 0.98 | 0.499 | 0.764 | 1.000 | 3477 | tags=44%, list=27%, signal=59% |
| 150 | NUCLEAR\_IMPORT |  | 47 | 0.22 | 0.97 | 0.495 | 0.786 | 1.000 | 4058 | tags=36%, list=31%, signal=52% |
| 151 | REGULATION\_OF\_GENE\_EXPRESSION\_EPIGENETIC |  | 27 | 0.25 | 0.96 | 0.509 | 0.788 | 1.000 | 3989 | tags=44%, list=30%, signal=64% |
| 152 | REGULATION\_OF\_PROGRAMMED\_CELL\_DEATH |  | 313 | 0.15 | 0.94 | 0.630 | 0.842 | 1.000 | 2986 | tags=25%, list=23%, signal=32% |
| 153 | RESPONSE\_TO\_OXIDATIVE\_STRESS |  | 38 | 0.22 | 0.93 | 0.567 | 0.864 | 1.000 | 3289 | tags=39%, list=25%, signal=53% |
| 154 | REGULATION\_OF\_APOPTOSIS |  | 312 | 0.16 | 0.93 | 0.666 | 0.873 | 1.000 | 2986 | tags=25%, list=23%, signal=32% |
| 155 | ENERGY\_DERIVATION\_BY\_OXIDATION\_OF\_ORGANIC\_COMPOUNDS |  | 37 | 0.23 | 0.93 | 0.558 | 0.868 | 1.000 | 1916 | tags=22%, list=15%, signal=25% |
| 156 | PIGMENT\_BIOSYNTHETIC\_PROCESS |  | 17 | 0.28 | 0.92 | 0.578 | 0.871 | 1.000 | 1737 | tags=24%, list=13%, signal=27% |
| 157 | PROTEIN\_HOMOOLIGOMERIZATION |  | 19 | 0.26 | 0.92 | 0.543 | 0.870 | 1.000 | 507 | tags=16%, list=4%, signal=16% |
| 158 | DEVELOPMENT\_OF\_PRIMARY\_SEXUAL\_CHARACTERISTICS |  | 25 | 0.25 | 0.92 | 0.578 | 0.878 | 1.000 | 3267 | tags=32%, list=25%, signal=43% |
| 159 | SEXUAL\_REPRODUCTION |  | 109 | 0.18 | 0.92 | 0.591 | 0.873 | 1.000 | 4454 | tags=41%, list=34%, signal=62% |
| 160 | ANATOMICAL\_STRUCTURE\_MORPHOGENESIS |  | 336 | 0.15 | 0.91 | 0.732 | 0.900 | 1.000 | 2728 | tags=22%, list=21%, signal=27% |
| 161 | MACROMOLECULE\_LOCALIZATION |  | 202 | 0.16 | 0.90 | 0.705 | 0.902 | 1.000 | 2803 | tags=22%, list=21%, signal=28% |
| 162 | MEMBRANE\_LIPID\_BIOSYNTHETIC\_PROCESS |  | 41 | 0.21 | 0.89 | 0.642 | 0.921 | 1.000 | 1954 | tags=20%, list=15%, signal=23% |
| 163 | TRANSMISSION\_OF\_NERVE\_IMPULSE |  | 167 | 0.16 | 0.89 | 0.694 | 0.916 | 1.000 | 3794 | tags=31%, list=29%, signal=42% |
| 164 | PHOSPHOINOSITIDE\_BIOSYNTHETIC\_PROCESS |  | 21 | 0.26 | 0.89 | 0.593 | 0.915 | 1.000 | 877 | tags=19%, list=7%, signal=20% |
| 165 | PIGMENT\_METABOLIC\_PROCESS |  | 18 | 0.26 | 0.89 | 0.612 | 0.913 | 1.000 | 3270 | tags=33%, list=25%, signal=44% |
| 166 | NEURON\_APOPTOSIS |  | 15 | 0.27 | 0.89 | 0.640 | 0.918 | 1.000 | 1198 | tags=20%, list=9%, signal=22% |
| 167 | NEGATIVE\_REGULATION\_OF\_CELL\_ADHESION |  | 16 | 0.28 | 0.89 | 0.615 | 0.916 | 1.000 | 2458 | tags=31%, list=19%, signal=38% |
| 168 | PROTEIN\_RNA\_COMPLEX\_ASSEMBLY |  | 55 | 0.20 | 0.88 | 0.667 | 0.917 | 1.000 | 3585 | tags=35%, list=27%, signal=47% |
| 169 | MEMBRANE\_FUSION |  | 27 | 0.23 | 0.88 | 0.647 | 0.918 | 1.000 | 2447 | tags=30%, list=19%, signal=36% |
| 170 | SECONDARY\_METABOLIC\_PROCESS |  | 23 | 0.24 | 0.88 | 0.633 | 0.913 | 1.000 | 1737 | tags=22%, list=13%, signal=25% |
| 171 | PROTEIN\_IMPORT |  | 58 | 0.19 | 0.87 | 0.657 | 0.934 | 1.000 | 4067 | tags=33%, list=31%, signal=47% |
| 172 | ENERGY\_RESERVE\_METABOLIC\_PROCESS |  | 15 | 0.27 | 0.85 | 0.659 | 0.967 | 1.000 | 1254 | tags=20%, list=10%, signal=22% |
| 173 | REGULATION\_OF\_NEUROTRANSMITTER\_LEVELS |  | 23 | 0.23 | 0.85 | 0.655 | 0.966 | 1.000 | 278 | tags=13%, list=2%, signal=13% |
| 174 | REGULATION\_OF\_TRANSPORT |  | 57 | 0.19 | 0.84 | 0.740 | 0.979 | 1.000 | 3259 | tags=30%, list=25%, signal=40% |
| 175 | SYNAPTIC\_TRANSMISSION |  | 154 | 0.15 | 0.84 | 0.806 | 0.976 | 1.000 | 2725 | tags=21%, list=21%, signal=27% |
| 176 | NEGATIVE\_REGULATION\_OF\_TRANSPORT |  | 18 | 0.25 | 0.84 | 0.690 | 0.983 | 1.000 | 3259 | tags=33%, list=25%, signal=44% |
| 177 | RESPONSE\_TO\_TEMPERATURE\_STIMULUS |  | 16 | 0.26 | 0.83 | 0.689 | 0.984 | 1.000 | 4581 | tags=56%, list=35%, signal=86% |
| 178 | INTRACELLULAR\_PROTEIN\_TRANSPORT |  | 127 | 0.15 | 0.83 | 0.847 | 0.981 | 1.000 | 4215 | tags=33%, list=32%, signal=48% |
| 179 | DIGESTION |  | 42 | 0.20 | 0.83 | 0.744 | 0.979 | 1.000 | 2729 | tags=21%, list=21%, signal=27% |
| 180 | PROTEIN\_TARGETING |  | 94 | 0.16 | 0.83 | 0.827 | 0.982 | 1.000 | 4215 | tags=33%, list=32%, signal=48% |
| 181 | BIOGENIC\_AMINE\_METABOLIC\_PROCESS |  | 16 | 0.25 | 0.82 | 0.710 | 0.983 | 1.000 | 4573 | tags=56%, list=35%, signal=86% |
| 182 | RHO\_PROTEIN\_SIGNAL\_TRANSDUCTION |  | 30 | 0.21 | 0.82 | 0.727 | 0.991 | 1.000 | 3342 | tags=30%, list=26%, signal=40% |
| 183 | REGULATION\_OF\_CELL\_ADHESION |  | 31 | 0.21 | 0.82 | 0.741 | 0.987 | 1.000 | 2458 | tags=26%, list=19%, signal=32% |
| 184 | SPLICEOSOME\_ASSEMBLY |  | 17 | 0.25 | 0.82 | 0.720 | 0.984 | 1.000 | 3189 | tags=35%, list=24%, signal=47% |
| 185 | REGULATION\_OF\_CATABOLIC\_PROCESS |  | 15 | 0.26 | 0.82 | 0.707 | 0.979 | 1.000 | 2097 | tags=27%, list=16%, signal=32% |
| 186 | NITROGEN\_COMPOUND\_METABOLIC\_PROCESS |  | 141 | 0.15 | 0.82 | 0.863 | 0.975 | 1.000 | 2148 | tags=18%, list=16%, signal=22% |
| 187 | CALCIUM\_INDEPENDENT\_CELL\_CELL\_ADHESION |  | 16 | 0.25 | 0.81 | 0.706 | 0.972 | 1.000 | 4125 | tags=44%, list=32%, signal=64% |
| 188 | REPRODUCTION |  | 215 | 0.14 | 0.81 | 0.925 | 0.967 | 1.000 | 3887 | tags=33%, list=30%, signal=46% |
| 189 | PROTEIN\_TRANSPORT |  | 139 | 0.15 | 0.81 | 0.853 | 0.963 | 1.000 | 4215 | tags=33%, list=32%, signal=48% |
| 190 | GLYCEROPHOSPHOLIPID\_BIOSYNTHETIC\_PROCESS |  | 27 | 0.21 | 0.81 | 0.727 | 0.967 | 1.000 | 877 | tags=15%, list=7%, signal=16% |
| 191 | LIPID\_TRANSPORT |  | 27 | 0.22 | 0.81 | 0.753 | 0.965 | 1.000 | 1817 | tags=22%, list=14%, signal=26% |
| 192 | REGULATION\_OF\_SECRETION |  | 35 | 0.20 | 0.81 | 0.777 | 0.963 | 1.000 | 2076 | tags=23%, list=16%, signal=27% |
| 193 | TRANSLATIONAL\_INITIATION |  | 33 | 0.20 | 0.80 | 0.783 | 0.967 | 1.000 | 3585 | tags=36%, list=27%, signal=50% |
| 194 | NEUROLOGICAL\_SYSTEM\_PROCESS |  | 328 | 0.13 | 0.80 | 0.966 | 0.965 | 1.000 | 4827 | tags=38%, list=37%, signal=59% |
| 195 | HISTONE\_MODIFICATION |  | 21 | 0.23 | 0.80 | 0.732 | 0.961 | 1.000 | 1350 | tags=19%, list=10%, signal=21% |
| 196 | AEROBIC\_RESPIRATION |  | 15 | 0.25 | 0.79 | 0.747 | 0.963 | 1.000 | 2778 | tags=33%, list=21%, signal=42% |
| 197 | DNA\_DAMAGE\_RESPONSESIGNAL\_TRANSDUCTION\_RESULTING\_IN\_INDUCTION\_OF\_APOPTOSIS |  | 15 | 0.25 | 0.79 | 0.766 | 0.963 | 1.000 | 1975 | tags=27%, list=15%, signal=31% |
| 198 | CASPASE\_ACTIVATION |  | 24 | 0.21 | 0.79 | 0.765 | 0.958 | 1.000 | 2917 | tags=29%, list=22%, signal=37% |
| 199 | NEGATIVE\_REGULATION\_OF\_CELLULAR\_BIOSYNTHETIC\_PROCESS |  | 25 | 0.21 | 0.78 | 0.793 | 0.968 | 1.000 | 1905 | tags=20%, list=15%, signal=23% |
| 200 | PROTEIN\_IMPORT\_INTO\_NUCLEUS |  | 45 | 0.18 | 0.78 | 0.841 | 0.970 | 1.000 | 4058 | tags=33%, list=31%, signal=48% |
| 201 | RESPONSE\_TO\_RADIATION |  | 52 | 0.17 | 0.77 | 0.844 | 0.975 | 1.000 | 3877 | tags=37%, list=30%, signal=52% |
| 202 | PHOSPHOLIPID\_BIOSYNTHETIC\_PROCESS |  | 35 | 0.19 | 0.77 | 0.844 | 0.973 | 1.000 | 1672 | tags=17%, list=13%, signal=20% |
| 203 | NEGATIVE\_REGULATION\_OF\_BIOSYNTHETIC\_PROCESS |  | 26 | 0.20 | 0.76 | 0.807 | 0.974 | 1.000 | 1905 | tags=19%, list=15%, signal=22% |
| 204 | LIPOPROTEIN\_METABOLIC\_PROCESS |  | 30 | 0.20 | 0.76 | 0.818 | 0.971 | 1.000 | 1817 | tags=20%, list=14%, signal=23% |
| 205 | SENSORY\_PERCEPTION |  | 163 | 0.14 | 0.76 | 0.952 | 0.973 | 1.000 | 5046 | tags=43%, list=39%, signal=69% |
| 206 | CHROMATIN\_ASSEMBLY |  | 16 | 0.23 | 0.75 | 0.804 | 0.981 | 1.000 | 3964 | tags=44%, list=30%, signal=63% |
| 207 | TUBE\_DEVELOPMENT |  | 15 | 0.23 | 0.74 | 0.809 | 0.982 | 1.000 | 3458 | tags=33%, list=26%, signal=45% |
| 208 | HOMEOSTASIS\_OF\_NUMBER\_OF\_CELLS |  | 20 | 0.21 | 0.74 | 0.827 | 0.979 | 1.000 | 3458 | tags=35%, list=26%, signal=47% |
| 209 | CELLULAR\_CARBOHYDRATE\_METABOLIC\_PROCESS |  | 106 | 0.14 | 0.74 | 0.938 | 0.977 | 1.000 | 2221 | tags=17%, list=17%, signal=20% |
| 210 | ESTABLISHMENT\_OF\_PROTEIN\_LOCALIZATION |  | 166 | 0.13 | 0.71 | 0.990 | 1.000 | 1.000 | 4215 | tags=31%, list=32%, signal=46% |
| 211 | REPRODUCTIVE\_PROCESS |  | 133 | 0.13 | 0.71 | 0.978 | 0.998 | 1.000 | 4036 | tags=33%, list=31%, signal=47% |
| 212 | RESPONSE\_TO\_UV |  | 22 | 0.19 | 0.69 | 0.879 | 1.000 | 1.000 | 3451 | tags=36%, list=26%, signal=49% |
| 213 | PROTEIN\_AMINO\_ACID\_LIPIDATION |  | 21 | 0.20 | 0.68 | 0.884 | 1.000 | 1.000 | 877 | tags=14%, list=7%, signal=15% |
| 214 | NEGATIVE\_REGULATION\_OF\_CELLULAR\_PROTEIN\_METABOLIC\_PROCESS |  | 41 | 0.16 | 0.68 | 0.938 | 1.000 | 1.000 | 2299 | tags=20%, list=18%, signal=24% |
| 215 | CELLULAR\_MORPHOGENESIS\_DURING\_DIFFERENTIATION |  | 38 | 0.16 | 0.66 | 0.933 | 1.000 | 1.000 | 2926 | tags=21%, list=22%, signal=27% |
| 216 | NEGATIVE\_REGULATION\_OF\_PROTEIN\_METABOLIC\_PROCESS |  | 44 | 0.15 | 0.65 | 0.963 | 1.000 | 1.000 | 2299 | tags=18%, list=18%, signal=22% |
| 217 | JNK\_CASCADE |  | 44 | 0.15 | 0.65 | 0.951 | 1.000 | 1.000 | 4675 | tags=45%, list=36%, signal=70% |
| 218 | FEEDING\_BEHAVIOR |  | 20 | 0.19 | 0.64 | 0.934 | 1.000 | 1.000 | 4948 | tags=45%, list=38%, signal=72% |
| 219 | EXOCYTOSIS |  | 22 | 0.18 | 0.64 | 0.910 | 1.000 | 1.000 | 9800 | tags=95%, list=75%, signal=379% |
| 220 | PATTERN\_SPECIFICATION\_PROCESS |  | 27 | 0.17 | 0.64 | 0.930 | 1.000 | 1.000 | 2988 | tags=26%, list=23%, signal=34% |
| 221 | AMINO\_SUGAR\_METABOLIC\_PROCESS |  | 15 | 0.19 | 0.63 | 0.948 | 1.000 | 1.000 | 2947 | tags=27%, list=23%, signal=34% |
| 222 | LIPOPROTEIN\_BIOSYNTHETIC\_PROCESS |  | 23 | 0.18 | 0.62 | 0.948 | 1.000 | 1.000 | 3822 | tags=35%, list=29%, signal=49% |
| 223 | ANION\_TRANSPORT |  | 27 | 0.16 | 0.61 | 0.973 | 1.000 | 1.000 | 2260 | tags=19%, list=17%, signal=22% |
| 224 | EPIDERMAL\_GROWTH\_FACTOR\_RECEPTOR\_SIGNALING\_PATHWAY |  | 18 | 0.17 | 0.58 | 0.948 | 1.000 | 1.000 | 3829 | tags=33%, list=29%, signal=47% |
| 225 | NEGATIVE\_REGULATION\_OF\_TRANSLATION |  | 19 | 0.17 | 0.58 | 0.969 | 1.000 | 1.000 | 3437 | tags=26%, list=26%, signal=36% |
| 226 | REGULATION\_OF\_ACTION\_POTENTIAL |  | 16 | 0.16 | 0.54 | 0.986 | 1.000 | 1.000 | 2856 | tags=25%, list=22%, signal=32% |
| 227 | FEMALE\_GAMETE\_GENERATION |  | 15 | 0.17 | 0.53 | 0.976 | 1.000 | 1.000 | 3050 | tags=27%, list=23%, signal=35% |
| 228 | REGULATION\_OF\_HEART\_CONTRACTION |  | 24 | 0.14 | 0.49 | 0.992 | 1.000 | 1.000 | 11291 | tags=100%, list=86%, signal=726% |
| 229 | GLUCOSE\_METABOLIC\_PROCESS |  | 27 | 0.12 | 0.46 | 1.000 | 1.000 | 1.000 | 4877 | tags=37%, list=37%, signal=59% |
| 230 | REGULATION\_OF\_CELL\_GROWTH |  | 39 | 0.10 | 0.40 | 1.000 | 1.000 | 1.000 | 5238 | tags=41%, list=40%, signal=68% |
Table: Gene sets enriched in phenotype **na**[plain text format]****

  
