# Supplementary material for: Multiple-input multiple-output causal strategies for gene selection
Source: BMC Bioinformatics. 2011 Nov 25;12:458. doi: 10.1186/1471-2105-12-458 (PMC3323860; doi:10.1186/1471-2105-12-458)
Supplement: Additional file 3 — Archive containing the output files computed by the preranked GSEA for λ ∈ {0.6,0.7,0.8,0.9,1.0,2.0} (GSEA_MIMO_part2.zip). [file 1471-2105-12-458-S3.ZIP › mFS06_entrez_mimo.GseaPreranked.1316038724366/gsea_report_for_na_pos_1316038724366.html]

Report for na\_pos 1316038724366 [GSEA]

| GS  follow link to MSigDB | GS DETAILS | SIZE | ES | NES | NOM p-val | FDR q-val | FWER p-val | RANK AT MAX | LEADING EDGE || 1 | M\_PHASE |  | 98 | 0.54 | 2.70 | 0.000 | 0.000 | 0.000 | 2517 | tags=53%, list=19%, signal=65% |
| 2 | MITOTIC\_CELL\_CYCLE |  | 134 | 0.50 | 2.69 | 0.000 | 0.000 | 0.000 | 2748 | tags=51%, list=21%, signal=65% |
| 3 | M\_PHASE\_OF\_MITOTIC\_CELL\_CYCLE |  | 72 | 0.57 | 2.69 | 0.000 | 0.000 | 0.000 | 2517 | tags=57%, list=19%, signal=70% |
| 4 | CELL\_CYCLE\_PROCESS |  | 169 | 0.48 | 2.67 | 0.000 | 0.000 | 0.000 | 2517 | tags=48%, list=19%, signal=59% |
| 5 | MITOSIS |  | 70 | 0.56 | 2.67 | 0.000 | 0.000 | 0.000 | 2517 | tags=56%, list=19%, signal=69% |
| 6 | CELL\_CYCLE\_PHASE |  | 152 | 0.46 | 2.53 | 0.000 | 0.000 | 0.000 | 2748 | tags=48%, list=21%, signal=60% |
| 7 | DNA\_REPLICATION |  | 97 | 0.49 | 2.52 | 0.000 | 0.000 | 0.000 | 2852 | tags=48%, list=22%, signal=61% |
| 8 | DNA\_METABOLIC\_PROCESS |  | 240 | 0.41 | 2.42 | 0.000 | 0.000 | 0.001 | 2907 | tags=45%, list=22%, signal=56% |
| 9 | CELL\_CYCLE\_GO\_0007049 |  | 277 | 0.41 | 2.42 | 0.000 | 0.000 | 0.001 | 2525 | tags=42%, list=19%, signal=51% |
| 10 | SISTER\_CHROMATID\_SEGREGATION |  | 16 | 0.74 | 2.41 | 0.000 | 0.000 | 0.001 | 812 | tags=56%, list=6%, signal=60% |
| 11 | MITOTIC\_SISTER\_CHROMATID\_SEGREGATION |  | 15 | 0.76 | 2.41 | 0.000 | 0.000 | 0.001 | 812 | tags=60%, list=6%, signal=64% |
| 12 | CHROMOSOME\_SEGREGATION |  | 28 | 0.60 | 2.33 | 0.000 | 0.000 | 0.001 | 812 | tags=46%, list=6%, signal=49% |
| 13 | DNA\_DEPENDENT\_DNA\_REPLICATION |  | 52 | 0.52 | 2.32 | 0.000 | 0.000 | 0.001 | 2852 | tags=54%, list=22%, signal=69% |
| 14 | CELL\_CYCLE\_CHECKPOINT\_GO\_0000075 |  | 45 | 0.53 | 2.31 | 0.000 | 0.000 | 0.001 | 2042 | tags=53%, list=16%, signal=63% |
| 15 | DNA\_REPAIR |  | 118 | 0.44 | 2.29 | 0.000 | 0.000 | 0.002 | 3260 | tags=51%, list=25%, signal=67% |
| 16 | REGULATION\_OF\_MITOSIS |  | 33 | 0.57 | 2.27 | 0.000 | 0.000 | 0.004 | 1910 | tags=52%, list=15%, signal=60% |
| 17 | RESPONSE\_TO\_DNA\_DAMAGE\_STIMULUS |  | 153 | 0.41 | 2.23 | 0.000 | 0.000 | 0.007 | 2440 | tags=42%, list=19%, signal=51% |
| 18 | RNA\_SPLICING |  | 74 | 0.46 | 2.22 | 0.000 | 0.000 | 0.009 | 3365 | tags=53%, list=26%, signal=71% |
| 19 | RESPONSE\_TO\_ENDOGENOUS\_STIMULUS |  | 182 | 0.39 | 2.17 | 0.000 | 0.001 | 0.017 | 3260 | tags=46%, list=25%, signal=60% |
| 20 | NUCLEOTIDE\_BIOSYNTHETIC\_PROCESS |  | 17 | 0.65 | 2.16 | 0.000 | 0.001 | 0.017 | 1363 | tags=53%, list=10%, signal=59% |
| 21 | RNA\_PROCESSING |  | 138 | 0.40 | 2.13 | 0.000 | 0.001 | 0.025 | 3365 | tags=50%, list=26%, signal=67% |
| 22 | DNA\_INTEGRITY\_CHECKPOINT |  | 22 | 0.59 | 2.08 | 0.000 | 0.002 | 0.054 | 2042 | tags=59%, list=16%, signal=70% |
| 23 | MICROTUBULE\_CYTOSKELETON\_ORGANIZATION\_AND\_BIOGENESIS |  | 31 | 0.53 | 2.08 | 0.000 | 0.002 | 0.054 | 2707 | tags=55%, list=21%, signal=69% |
| 24 | DNA\_REPLICATION\_INITIATION |  | 15 | 0.65 | 2.05 | 0.000 | 0.003 | 0.077 | 2737 | tags=80%, list=21%, signal=101% |
| 25 | MITOTIC\_CELL\_CYCLE\_CHECKPOINT |  | 19 | 0.59 | 2.05 | 0.002 | 0.003 | 0.086 | 1910 | tags=53%, list=15%, signal=62% |
| 26 | PROTEIN\_FOLDING |  | 55 | 0.44 | 2.02 | 0.000 | 0.003 | 0.114 | 3182 | tags=51%, list=24%, signal=67% |
| 27 | MRNA\_METABOLIC\_PROCESS |  | 72 | 0.42 | 2.01 | 0.000 | 0.003 | 0.118 | 3345 | tags=51%, list=26%, signal=69% |
| 28 | MITOCHONDRION\_ORGANIZATION\_AND\_BIOGENESIS |  | 42 | 0.47 | 2.00 | 0.000 | 0.004 | 0.132 | 3172 | tags=50%, list=24%, signal=66% |
| 29 | DOUBLE\_STRAND\_BREAK\_REPAIR |  | 21 | 0.57 | 2.00 | 0.004 | 0.004 | 0.132 | 1870 | tags=52%, list=14%, signal=61% |
| 30 | REGULATION\_OF\_MITOTIC\_CELL\_CYCLE |  | 19 | 0.58 | 1.99 | 0.002 | 0.004 | 0.139 | 1351 | tags=47%, list=10%, signal=53% |
| 31 | REGULATION\_OF\_CELL\_CYCLE |  | 161 | 0.36 | 1.97 | 0.000 | 0.005 | 0.185 | 1635 | tags=32%, list=12%, signal=36% |
| 32 | TRNA\_METABOLIC\_PROCESS |  | 15 | 0.61 | 1.94 | 0.004 | 0.006 | 0.225 | 2687 | tags=67%, list=21%, signal=84% |
| 33 | COENZYME\_METABOLIC\_PROCESS |  | 35 | 0.48 | 1.94 | 0.002 | 0.006 | 0.230 | 3404 | tags=49%, list=26%, signal=65% |
| 34 | INTERPHASE\_OF\_MITOTIC\_CELL\_CYCLE |  | 57 | 0.42 | 1.92 | 0.000 | 0.008 | 0.280 | 3377 | tags=51%, list=26%, signal=68% |
| 35 | MRNA\_PROCESSING\_GO\_0006397 |  | 61 | 0.42 | 1.92 | 0.002 | 0.008 | 0.284 | 3345 | tags=49%, list=26%, signal=66% |
| 36 | NUCLEOBASENUCLEOSIDENUCLEOTIDE\_AND\_NUCLEIC\_ACID\_TRANSPORT |  | 26 | 0.50 | 1.91 | 0.002 | 0.008 | 0.313 | 2188 | tags=46%, list=17%, signal=55% |
| 37 | INTERPHASE |  | 63 | 0.41 | 1.90 | 0.000 | 0.009 | 0.334 | 3377 | tags=49%, list=26%, signal=66% |
| 38 | COFACTOR\_BIOSYNTHETIC\_PROCESS |  | 21 | 0.53 | 1.89 | 0.002 | 0.010 | 0.381 | 1458 | tags=38%, list=11%, signal=43% |
| 39 | REGULATION\_OF\_DNA\_METABOLIC\_PROCESS |  | 40 | 0.45 | 1.87 | 0.000 | 0.011 | 0.425 | 2060 | tags=45%, list=16%, signal=53% |
| 40 | REGULATION\_OF\_CYCLIN\_DEPENDENT\_PROTEIN\_KINASE\_ACTIVITY |  | 40 | 0.45 | 1.87 | 0.005 | 0.011 | 0.437 | 2675 | tags=50%, list=20%, signal=63% |
| 41 | CHROMOSOME\_ORGANIZATION\_AND\_BIOGENESIS |  | 107 | 0.36 | 1.85 | 0.000 | 0.012 | 0.467 | 2857 | tags=39%, list=22%, signal=50% |
| 42 | REGULATION\_OF\_DNA\_REPLICATION |  | 18 | 0.55 | 1.85 | 0.009 | 0.012 | 0.472 | 2042 | tags=50%, list=16%, signal=59% |
| 43 | MITOCHONDRIAL\_TRANSPORT |  | 18 | 0.54 | 1.81 | 0.004 | 0.016 | 0.587 | 1430 | tags=44%, list=11%, signal=50% |
| 44 | G1\_S\_TRANSITION\_OF\_MITOTIC\_CELL\_CYCLE |  | 23 | 0.49 | 1.81 | 0.000 | 0.017 | 0.611 | 2675 | tags=48%, list=20%, signal=60% |
| 45 | DNA\_DAMAGE\_CHECKPOINT |  | 19 | 0.53 | 1.79 | 0.004 | 0.018 | 0.639 | 2042 | tags=53%, list=16%, signal=62% |
| 46 | PROTEIN\_MODIFICATION\_BY\_SMALL\_PROTEIN\_CONJUGATION |  | 35 | 0.44 | 1.78 | 0.002 | 0.019 | 0.679 | 2253 | tags=40%, list=17%, signal=48% |
| 47 | TRANSCRIPTION\_INITIATION\_FROM\_RNA\_POLYMERASE\_II\_PROMOTER |  | 27 | 0.47 | 1.76 | 0.008 | 0.022 | 0.717 | 2525 | tags=44%, list=19%, signal=55% |
| 48 | UBIQUITIN\_CYCLE |  | 40 | 0.42 | 1.76 | 0.005 | 0.022 | 0.727 | 2253 | tags=38%, list=17%, signal=45% |
| 49 | COFACTOR\_METABOLIC\_PROCESS |  | 51 | 0.40 | 1.76 | 0.002 | 0.021 | 0.728 | 3462 | tags=43%, list=26%, signal=58% |
| 50 | PROTEIN\_CATABOLIC\_PROCESS |  | 60 | 0.38 | 1.74 | 0.004 | 0.025 | 0.800 | 2254 | tags=33%, list=17%, signal=40% |
| 51 | DNA\_DAMAGE\_RESPONSESIGNAL\_TRANSDUCTION |  | 34 | 0.44 | 1.72 | 0.002 | 0.028 | 0.836 | 2413 | tags=47%, list=18%, signal=58% |
| 52 | CELLULAR\_PROTEIN\_CATABOLIC\_PROCESS |  | 50 | 0.40 | 1.72 | 0.007 | 0.028 | 0.848 | 2254 | tags=34%, list=17%, signal=41% |
| 53 | BIOPOLYMER\_CATABOLIC\_PROCESS |  | 103 | 0.34 | 1.72 | 0.000 | 0.028 | 0.858 | 3316 | tags=41%, list=25%, signal=54% |
| 54 | PROTEIN\_UBIQUITINATION |  | 32 | 0.43 | 1.71 | 0.011 | 0.030 | 0.880 | 2253 | tags=38%, list=17%, signal=45% |
| 55 | DNA\_RECOMBINATION |  | 45 | 0.40 | 1.69 | 0.007 | 0.032 | 0.905 | 1436 | tags=33%, list=11%, signal=37% |
| 56 | PROTEIN\_DNA\_COMPLEX\_ASSEMBLY |  | 45 | 0.39 | 1.68 | 0.004 | 0.036 | 0.928 | 2525 | tags=40%, list=19%, signal=49% |
| 57 | NUCLEAR\_EXPORT |  | 26 | 0.45 | 1.68 | 0.009 | 0.036 | 0.932 | 4225 | tags=58%, list=32%, signal=85% |
| 58 | RNA\_EXPORT\_FROM\_NUCLEUS |  | 17 | 0.50 | 1.66 | 0.026 | 0.041 | 0.957 | 4543 | tags=71%, list=35%, signal=108% |
| 59 | MACROMOLECULE\_CATABOLIC\_PROCESS |  | 120 | 0.31 | 1.64 | 0.003 | 0.046 | 0.972 | 3352 | tags=38%, list=26%, signal=50% |
| 60 | DNA\_PACKAGING |  | 29 | 0.43 | 1.64 | 0.022 | 0.046 | 0.974 | 2703 | tags=45%, list=21%, signal=56% |
| 61 | CYTOKINESIS |  | 17 | 0.49 | 1.64 | 0.025 | 0.046 | 0.974 | 1029 | tags=35%, list=8%, signal=38% |
| 62 | BASE\_EXCISION\_REPAIR |  | 16 | 0.49 | 1.63 | 0.017 | 0.046 | 0.976 | 2700 | tags=44%, list=21%, signal=55% |
| 63 | MEIOSIS\_I |  | 19 | 0.47 | 1.62 | 0.017 | 0.051 | 0.986 | 1436 | tags=37%, list=11%, signal=41% |
| 64 | ONE\_CARBON\_COMPOUND\_METABOLIC\_PROCESS |  | 24 | 0.45 | 1.60 | 0.024 | 0.055 | 0.990 | 2842 | tags=50%, list=22%, signal=64% |
| 65 | MEIOTIC\_CELL\_CYCLE |  | 31 | 0.41 | 1.57 | 0.017 | 0.068 | 0.998 | 2707 | tags=42%, list=21%, signal=53% |
| 66 | NUCLEAR\_TRANSPORT |  | 77 | 0.32 | 1.57 | 0.007 | 0.069 | 0.998 | 3257 | tags=38%, list=25%, signal=50% |
| 67 | CELLULAR\_MACROMOLECULE\_CATABOLIC\_PROCESS |  | 90 | 0.31 | 1.57 | 0.007 | 0.069 | 0.998 | 2337 | tags=30%, list=18%, signal=36% |
| 68 | NUCLEOCYTOPLASMIC\_TRANSPORT |  | 77 | 0.32 | 1.54 | 0.014 | 0.079 | 0.999 | 3257 | tags=38%, list=25%, signal=50% |
| 69 | TRANSCRIPTION\_INITIATION |  | 33 | 0.39 | 1.53 | 0.034 | 0.084 | 0.999 | 2525 | tags=39%, list=19%, signal=49% |
| 70 | ORGANELLE\_ORGANIZATION\_AND\_BIOGENESIS |  | 407 | 0.25 | 1.53 | 0.000 | 0.083 | 0.999 | 3260 | tags=34%, list=25%, signal=43% |
| 71 | MICROTUBULE\_BASED\_PROCESS |  | 75 | 0.32 | 1.53 | 0.007 | 0.085 | 1.000 | 2758 | tags=36%, list=21%, signal=45% |
| 72 | NUCLEOTIDE\_METABOLIC\_PROCESS |  | 36 | 0.38 | 1.52 | 0.038 | 0.085 | 1.000 | 1363 | tags=31%, list=10%, signal=34% |
| 73 | CELL\_DIVISION |  | 19 | 0.45 | 1.51 | 0.050 | 0.094 | 1.000 | 1029 | tags=32%, list=8%, signal=34% |
| 74 | NUCLEOBASENUCLEOSIDE\_AND\_NUCLEOTIDE\_METABOLIC\_PROCESS |  | 46 | 0.35 | 1.50 | 0.014 | 0.096 | 1.000 | 1363 | tags=28%, list=10%, signal=31% |
| 75 | APOPTOTIC\_NUCLEAR\_CHANGES |  | 17 | 0.45 | 1.50 | 0.061 | 0.097 | 1.000 | 2675 | tags=47%, list=20%, signal=59% |
| 76 | CHROMATIN\_ASSEMBLY\_OR\_DISASSEMBLY |  | 25 | 0.40 | 1.49 | 0.053 | 0.100 | 1.000 | 2703 | tags=48%, list=21%, signal=60% |
| 77 | ESTABLISHMENT\_OF\_ORGANELLE\_LOCALIZATION |  | 16 | 0.46 | 1.46 | 0.064 | 0.122 | 1.000 | 907 | tags=38%, list=7%, signal=40% |
| 78 | CELLULAR\_COMPONENT\_DISASSEMBLY |  | 31 | 0.37 | 1.44 | 0.057 | 0.133 | 1.000 | 2675 | tags=39%, list=20%, signal=49% |
| 79 | ORGANELLE\_LOCALIZATION |  | 21 | 0.40 | 1.43 | 0.081 | 0.145 | 1.000 | 907 | tags=29%, list=7%, signal=31% |
| 80 | VIRAL\_INFECTIOUS\_CYCLE |  | 29 | 0.37 | 1.43 | 0.065 | 0.143 | 1.000 | 1102 | tags=31%, list=8%, signal=34% |
| 81 | NEGATIVE\_REGULATION\_OF\_DNA\_METABOLIC\_PROCESS |  | 16 | 0.43 | 1.41 | 0.094 | 0.161 | 1.000 | 2562 | tags=50%, list=20%, signal=62% |
| 82 | VIRAL\_REPRODUCTIVE\_PROCESS |  | 33 | 0.35 | 1.37 | 0.104 | 0.203 | 1.000 | 1690 | tags=33%, list=13%, signal=38% |
| 83 | ALCOHOL\_METABOLIC\_PROCESS |  | 82 | 0.28 | 1.37 | 0.062 | 0.202 | 1.000 | 4445 | tags=45%, list=34%, signal=68% |
| 84 | ESTABLISHMENT\_AND\_OR\_MAINTENANCE\_OF\_CHROMATIN\_ARCHITECTURE |  | 65 | 0.29 | 1.36 | 0.067 | 0.208 | 1.000 | 2778 | tags=35%, list=21%, signal=45% |
| 85 | NEGATIVE\_REGULATION\_OF\_BINDING |  | 16 | 0.42 | 1.36 | 0.114 | 0.209 | 1.000 | 2675 | tags=50%, list=20%, signal=63% |
| 86 | CHROMATIN\_REMODELING |  | 21 | 0.38 | 1.33 | 0.127 | 0.239 | 1.000 | 2703 | tags=43%, list=21%, signal=54% |
| 87 | RESPONSE\_TO\_HYPOXIA |  | 27 | 0.35 | 1.32 | 0.128 | 0.249 | 1.000 | 2372 | tags=33%, list=18%, signal=41% |
| 88 | RESPONSE\_TO\_ORGANIC\_SUBSTANCE |  | 27 | 0.35 | 1.32 | 0.123 | 0.246 | 1.000 | 2808 | tags=37%, list=21%, signal=47% |
| 89 | INTRACELLULAR\_TRANSPORT |  | 248 | 0.22 | 1.32 | 0.031 | 0.255 | 1.000 | 3263 | tags=32%, list=25%, signal=42% |
| 90 | APOPTOTIC\_PROGRAM |  | 56 | 0.29 | 1.30 | 0.099 | 0.271 | 1.000 | 3919 | tags=46%, list=30%, signal=66% |
| 91 | REGULATION\_OF\_GENE\_EXPRESSION\_EPIGENETIC |  | 27 | 0.34 | 1.30 | 0.119 | 0.278 | 1.000 | 2842 | tags=41%, list=22%, signal=52% |
| 92 | VIRAL\_GENOME\_REPLICATION |  | 20 | 0.37 | 1.30 | 0.114 | 0.276 | 1.000 | 1690 | tags=35%, list=13%, signal=40% |
| 93 | VIRAL\_REPRODUCTION |  | 38 | 0.31 | 1.29 | 0.126 | 0.283 | 1.000 | 2002 | tags=32%, list=15%, signal=37% |
| 94 | G1\_PHASE |  | 15 | 0.40 | 1.29 | 0.165 | 0.281 | 1.000 | 533 | tags=27%, list=4%, signal=28% |
| 95 | MEIOTIC\_RECOMBINATION |  | 16 | 0.40 | 1.28 | 0.165 | 0.294 | 1.000 | 1436 | tags=31%, list=11%, signal=35% |
| 96 | CYTOSKELETON\_DEPENDENT\_INTRACELLULAR\_TRANSPORT |  | 25 | 0.35 | 1.28 | 0.144 | 0.294 | 1.000 | 3990 | tags=52%, list=30%, signal=75% |
| 97 | ESTABLISHMENT\_OF\_CELLULAR\_LOCALIZATION |  | 311 | 0.21 | 1.27 | 0.048 | 0.299 | 1.000 | 3263 | tags=30%, list=25%, signal=39% |
| 98 | RESPONSE\_TO\_ABIOTIC\_STIMULUS |  | 79 | 0.26 | 1.27 | 0.097 | 0.297 | 1.000 | 3404 | tags=37%, list=26%, signal=49% |
| 99 | HETEROCYCLE\_METABOLIC\_PROCESS |  | 26 | 0.35 | 1.27 | 0.152 | 0.302 | 1.000 | 1458 | tags=23%, list=11%, signal=26% |
| 100 | RNA\_CATABOLIC\_PROCESS |  | 20 | 0.37 | 1.26 | 0.172 | 0.303 | 1.000 | 3071 | tags=50%, list=23%, signal=65% |
| 101 | NEGATIVE\_REGULATION\_OF\_CATALYTIC\_ACTIVITY |  | 61 | 0.28 | 1.26 | 0.122 | 0.308 | 1.000 | 2696 | tags=34%, list=21%, signal=43% |
| 102 | CELLULAR\_LOCALIZATION |  | 323 | 0.20 | 1.25 | 0.059 | 0.331 | 1.000 | 3263 | tags=30%, list=25%, signal=39% |
| 103 | RESPONSE\_TO\_STRESS |  | 467 | 0.20 | 1.24 | 0.035 | 0.328 | 1.000 | 2908 | tags=28%, list=22%, signal=35% |
| 104 | RIBONUCLEOPROTEIN\_COMPLEX\_BIOGENESIS\_AND\_ASSEMBLY |  | 68 | 0.26 | 1.22 | 0.155 | 0.367 | 1.000 | 3668 | tags=40%, list=28%, signal=55% |
| 105 | DNA\_CATABOLIC\_PROCESS |  | 21 | 0.35 | 1.22 | 0.197 | 0.373 | 1.000 | 3260 | tags=43%, list=25%, signal=57% |
| 106 | NEGATIVE\_REGULATION\_OF\_DNA\_BINDING |  | 15 | 0.38 | 1.20 | 0.248 | 0.405 | 1.000 | 2675 | tags=47%, list=20%, signal=59% |
| 107 | NUCLEAR\_ORGANIZATION\_AND\_BIOGENESIS |  | 23 | 0.33 | 1.19 | 0.216 | 0.412 | 1.000 | 2675 | tags=39%, list=20%, signal=49% |
| 108 | CHROMATIN\_ASSEMBLY |  | 16 | 0.37 | 1.18 | 0.265 | 0.441 | 1.000 | 2703 | tags=44%, list=21%, signal=55% |
| 109 | CELLULAR\_RESPIRATION |  | 19 | 0.34 | 1.17 | 0.244 | 0.450 | 1.000 | 2667 | tags=37%, list=20%, signal=46% |
| 110 | TRANSCRIPTION\_FROM\_RNA\_POLYMERASE\_III\_PROMOTER |  | 18 | 0.35 | 1.17 | 0.270 | 0.454 | 1.000 | 3748 | tags=56%, list=29%, signal=78% |
| 111 | CHROMATIN\_MODIFICATION |  | 46 | 0.27 | 1.17 | 0.220 | 0.456 | 1.000 | 2778 | tags=33%, list=21%, signal=41% |
| 112 | CELL\_STRUCTURE\_DISASSEMBLY\_DURING\_APOPTOSIS |  | 17 | 0.35 | 1.16 | 0.280 | 0.457 | 1.000 | 2675 | tags=35%, list=20%, signal=44% |
| 113 | INDUCTION\_OF\_APOPTOSIS\_BY\_EXTRACELLULAR\_SIGNALS |  | 25 | 0.32 | 1.16 | 0.274 | 0.456 | 1.000 | 2618 | tags=36%, list=20%, signal=45% |
| 114 | TRANSCRIPTION\_FROM\_RNA\_POLYMERASE\_II\_PROMOTER |  | 428 | 0.18 | 1.15 | 0.102 | 0.468 | 1.000 | 3118 | tags=28%, list=24%, signal=35% |
| 115 | REGULATION\_OF\_HYDROLASE\_ACTIVITY |  | 65 | 0.24 | 1.14 | 0.233 | 0.486 | 1.000 | 3175 | tags=34%, list=24%, signal=44% |
| 116 | NITROGEN\_COMPOUND\_BIOSYNTHETIC\_PROCESS |  | 25 | 0.30 | 1.14 | 0.274 | 0.484 | 1.000 | 1589 | tags=24%, list=12%, signal=27% |
| 117 | CELLULAR\_RESPONSE\_TO\_STIMULUS |  | 17 | 0.34 | 1.13 | 0.294 | 0.509 | 1.000 | 4288 | tags=53%, list=33%, signal=79% |
| 118 | NUCLEAR\_IMPORT |  | 47 | 0.26 | 1.13 | 0.258 | 0.507 | 1.000 | 3257 | tags=34%, list=25%, signal=45% |
| 119 | RESPONSE\_TO\_HORMONE\_STIMULUS |  | 26 | 0.30 | 1.13 | 0.298 | 0.511 | 1.000 | 4957 | tags=58%, list=38%, signal=93% |
| 120 | REGULATION\_OF\_CATALYTIC\_ACTIVITY |  | 238 | 0.19 | 1.13 | 0.181 | 0.510 | 1.000 | 3307 | tags=30%, list=25%, signal=39% |
| 121 | REGULATION\_OF\_KINASE\_ACTIVITY |  | 135 | 0.21 | 1.12 | 0.240 | 0.510 | 1.000 | 2277 | tags=24%, list=17%, signal=28% |
| 122 | NEURON\_APOPTOSIS |  | 15 | 0.35 | 1.12 | 0.286 | 0.507 | 1.000 | 1222 | tags=27%, list=9%, signal=29% |
| 123 | CATABOLIC\_PROCESS |  | 201 | 0.20 | 1.12 | 0.215 | 0.513 | 1.000 | 3659 | tags=32%, list=28%, signal=44% |
| 124 | GLUTAMATE\_SIGNALING\_PATHWAY |  | 17 | 0.33 | 1.12 | 0.294 | 0.510 | 1.000 | 2981 | tags=29%, list=23%, signal=38% |
| 125 | CELLULAR\_CATABOLIC\_PROCESS |  | 189 | 0.20 | 1.11 | 0.227 | 0.523 | 1.000 | 2675 | tags=25%, list=20%, signal=31% |
| 126 | NEGATIVE\_REGULATION\_OF\_TRANSPORT |  | 18 | 0.33 | 1.11 | 0.311 | 0.525 | 1.000 | 3528 | tags=44%, list=27%, signal=61% |
| 127 | REGULATION\_OF\_TRANSFERASE\_ACTIVITY |  | 137 | 0.20 | 1.11 | 0.264 | 0.529 | 1.000 | 2277 | tags=23%, list=17%, signal=28% |
| 128 | LIPID\_BIOSYNTHETIC\_PROCESS |  | 84 | 0.23 | 1.10 | 0.303 | 0.546 | 1.000 | 1756 | tags=21%, list=13%, signal=25% |
| 129 | REGULATION\_OF\_PROTEIN\_KINASE\_ACTIVITY |  | 133 | 0.21 | 1.10 | 0.280 | 0.546 | 1.000 | 4457 | tags=44%, list=34%, signal=65% |
| 130 | INTERACTION\_WITH\_HOST |  | 15 | 0.34 | 1.08 | 0.361 | 0.594 | 1.000 | 1311 | tags=27%, list=10%, signal=30% |
| 131 | CYTOSKELETON\_ORGANIZATION\_AND\_BIOGENESIS |  | 182 | 0.19 | 1.07 | 0.314 | 0.610 | 1.000 | 2823 | tags=27%, list=22%, signal=34% |
| 132 | OXYGEN\_AND\_REACTIVE\_OXYGEN\_SPECIES\_METABOLIC\_PROCESS |  | 18 | 0.32 | 1.06 | 0.368 | 0.615 | 1.000 | 3400 | tags=44%, list=26%, signal=60% |
| 133 | DNA\_DAMAGE\_RESPONSESIGNAL\_TRANSDUCTION\_RESULTING\_IN\_INDUCTION\_OF\_APOPTOSIS |  | 15 | 0.33 | 1.05 | 0.372 | 0.639 | 1.000 | 1091 | tags=27%, list=8%, signal=29% |
| 134 | REGULATION\_OF\_MOLECULAR\_FUNCTION |  | 275 | 0.18 | 1.05 | 0.318 | 0.635 | 1.000 | 2696 | tags=24%, list=21%, signal=30% |
| 135 | REGULATION\_OF\_PROTEIN\_STABILITY |  | 17 | 0.32 | 1.05 | 0.385 | 0.633 | 1.000 | 4088 | tags=41%, list=31%, signal=60% |
| 136 | CELLULAR\_BIOSYNTHETIC\_PROCESS |  | 273 | 0.18 | 1.05 | 0.334 | 0.638 | 1.000 | 1624 | tags=18%, list=12%, signal=20% |
| 137 | PIGMENT\_BIOSYNTHETIC\_PROCESS |  | 17 | 0.31 | 1.04 | 0.403 | 0.646 | 1.000 | 1458 | tags=24%, list=11%, signal=26% |
| 138 | NEGATIVE\_REGULATION\_OF\_APOPTOSIS |  | 136 | 0.19 | 1.04 | 0.376 | 0.651 | 1.000 | 1530 | tags=19%, list=12%, signal=21% |
| 139 | INTRACELLULAR\_PROTEIN\_TRANSPORT |  | 127 | 0.19 | 1.04 | 0.377 | 0.657 | 1.000 | 3320 | tags=29%, list=25%, signal=39% |
| 140 | RNA\_SPLICINGVIA\_TRANSESTERIFICATION\_REACTIONS |  | 27 | 0.27 | 1.04 | 0.390 | 0.654 | 1.000 | 3616 | tags=37%, list=28%, signal=51% |
| 141 | GAMETE\_GENERATION |  | 92 | 0.21 | 1.03 | 0.392 | 0.678 | 1.000 | 3823 | tags=34%, list=29%, signal=47% |
| 142 | PROTEIN\_IMPORT |  | 58 | 0.23 | 1.02 | 0.419 | 0.678 | 1.000 | 3257 | tags=29%, list=25%, signal=39% |
| 143 | NEGATIVE\_REGULATION\_OF\_PROGRAMMED\_CELL\_DEATH |  | 137 | 0.19 | 1.02 | 0.426 | 0.676 | 1.000 | 1530 | tags=19%, list=12%, signal=21% |
| 144 | MACROMOLECULE\_LOCALIZATION |  | 202 | 0.18 | 1.02 | 0.425 | 0.688 | 1.000 | 3320 | tags=28%, list=25%, signal=37% |
| 145 | SECONDARY\_METABOLIC\_PROCESS |  | 23 | 0.27 | 1.00 | 0.458 | 0.716 | 1.000 | 1458 | tags=22%, list=11%, signal=24% |
| 146 | DIGESTION |  | 42 | 0.24 | 1.00 | 0.417 | 0.713 | 1.000 | 2896 | tags=24%, list=22%, signal=30% |
| 147 | RESPONSE\_TO\_TEMPERATURE\_STIMULUS |  | 16 | 0.31 | 1.00 | 0.465 | 0.712 | 1.000 | 3404 | tags=44%, list=26%, signal=59% |
| 148 | PROTEIN\_TARGETING |  | 94 | 0.20 | 1.00 | 0.457 | 0.711 | 1.000 | 3320 | tags=29%, list=25%, signal=38% |
| 149 | MICROTUBULE\_BASED\_MOVEMENT |  | 16 | 0.31 | 1.00 | 0.454 | 0.707 | 1.000 | 2758 | tags=38%, list=21%, signal=47% |
| 150 | PROTEIN\_TRANSPORT |  | 139 | 0.19 | 1.00 | 0.474 | 0.703 | 1.000 | 3320 | tags=29%, list=25%, signal=38% |
| 151 | NEGATIVE\_REGULATION\_OF\_TRANSFERASE\_ACTIVITY |  | 27 | 0.26 | 1.00 | 0.476 | 0.707 | 1.000 | 2696 | tags=33%, list=21%, signal=42% |
| 152 | STEROID\_BIOSYNTHETIC\_PROCESS |  | 22 | 0.28 | 0.99 | 0.476 | 0.715 | 1.000 | 4138 | tags=55%, list=32%, signal=80% |
| 153 | CARBOHYDRATE\_TRANSPORT |  | 17 | 0.30 | 0.99 | 0.461 | 0.710 | 1.000 | 2446 | tags=29%, list=19%, signal=36% |
| 154 | MORPHOGENESIS\_OF\_AN\_EPITHELIUM |  | 15 | 0.31 | 0.99 | 0.457 | 0.716 | 1.000 | 3763 | tags=47%, list=29%, signal=65% |
| 155 | PIGMENT\_METABOLIC\_PROCESS |  | 18 | 0.29 | 0.99 | 0.470 | 0.718 | 1.000 | 1458 | tags=22%, list=11%, signal=25% |
| 156 | REGULATION\_OF\_PROGRAMMED\_CELL\_DEATH |  | 313 | 0.16 | 0.99 | 0.503 | 0.715 | 1.000 | 1766 | tags=18%, list=13%, signal=20% |
| 157 | PROGRAMMED\_CELL\_DEATH |  | 393 | 0.16 | 0.99 | 0.489 | 0.712 | 1.000 | 1716 | tags=17%, list=13%, signal=19% |
| 158 | PROTEIN\_AMINO\_ACID\_O\_LINKED\_GLYCOSYLATION |  | 18 | 0.29 | 0.98 | 0.476 | 0.710 | 1.000 | 2716 | tags=39%, list=21%, signal=49% |
| 159 | APOPTOSIS\_GO |  | 392 | 0.16 | 0.98 | 0.511 | 0.712 | 1.000 | 1716 | tags=17%, list=13%, signal=19% |
| 160 | REGULATION\_OF\_TRANSCRIPTION\_FROM\_RNA\_POLYMERASE\_II\_PROMOTER |  | 267 | 0.16 | 0.98 | 0.489 | 0.709 | 1.000 | 2525 | tags=23%, list=19%, signal=28% |
| 161 | REGULATION\_OF\_NEUROTRANSMITTER\_LEVELS |  | 23 | 0.27 | 0.97 | 0.474 | 0.722 | 1.000 | 1002 | tags=17%, list=8%, signal=19% |
| 162 | REGULATION\_OF\_APOPTOSIS |  | 312 | 0.16 | 0.97 | 0.535 | 0.727 | 1.000 | 1766 | tags=18%, list=13%, signal=20% |
| 163 | CELL\_PROJECTION\_BIOGENESIS |  | 20 | 0.28 | 0.97 | 0.471 | 0.725 | 1.000 | 4185 | tags=45%, list=32%, signal=66% |
| 164 | STEROID\_HORMONE\_RECEPTOR\_SIGNALING\_PATHWAY |  | 18 | 0.29 | 0.97 | 0.497 | 0.722 | 1.000 | 1293 | tags=22%, list=10%, signal=25% |
| 165 | STEROID\_METABOLIC\_PROCESS |  | 66 | 0.21 | 0.97 | 0.522 | 0.720 | 1.000 | 4138 | tags=39%, list=32%, signal=57% |
| 166 | INTRACELLULAR\_RECEPTOR\_MEDIATED\_SIGNALING\_PATHWAY |  | 18 | 0.29 | 0.96 | 0.516 | 0.738 | 1.000 | 1293 | tags=22%, list=10%, signal=25% |
| 167 | ENERGY\_DERIVATION\_BY\_OXIDATION\_OF\_ORGANIC\_COMPOUNDS |  | 37 | 0.24 | 0.96 | 0.508 | 0.741 | 1.000 | 2667 | tags=27%, list=20%, signal=34% |
| 168 | GENERATION\_OF\_A\_SIGNAL\_INVOLVED\_IN\_CELL\_CELL\_SIGNALING |  | 25 | 0.26 | 0.95 | 0.535 | 0.755 | 1.000 | 2388 | tags=28%, list=18%, signal=34% |
| 169 | PROTEIN\_IMPORT\_INTO\_NUCLEUS |  | 45 | 0.22 | 0.95 | 0.539 | 0.764 | 1.000 | 3257 | tags=31%, list=25%, signal=41% |
| 170 | COVALENT\_CHROMATIN\_MODIFICATION |  | 22 | 0.27 | 0.94 | 0.530 | 0.762 | 1.000 | 3651 | tags=41%, list=28%, signal=57% |
| 171 | PROTEIN\_RNA\_COMPLEX\_ASSEMBLY |  | 55 | 0.21 | 0.93 | 0.561 | 0.785 | 1.000 | 3668 | tags=36%, list=28%, signal=50% |
| 172 | RESPONSE\_TO\_OXIDATIVE\_STRESS |  | 38 | 0.22 | 0.93 | 0.568 | 0.800 | 1.000 | 3428 | tags=42%, list=26%, signal=57% |
| 173 | REGULATION\_OF\_TRANSPORT |  | 57 | 0.21 | 0.93 | 0.553 | 0.796 | 1.000 | 4213 | tags=42%, list=32%, signal=62% |
| 174 | AROMATIC\_COMPOUND\_METABOLIC\_PROCESS |  | 26 | 0.24 | 0.93 | 0.566 | 0.794 | 1.000 | 334 | tags=15%, list=3%, signal=16% |
| 175 | BIOSYNTHETIC\_PROCESS |  | 402 | 0.15 | 0.92 | 0.727 | 0.803 | 1.000 | 1791 | tags=17%, list=14%, signal=19% |
| 176 | INDUCTION\_OF\_APOPTOSIS\_BY\_INTRACELLULAR\_SIGNALS |  | 23 | 0.24 | 0.89 | 0.612 | 0.866 | 1.000 | 3377 | tags=35%, list=26%, signal=47% |
| 177 | NEGATIVE\_REGULATION\_OF\_CELL\_ADHESION |  | 16 | 0.28 | 0.89 | 0.612 | 0.873 | 1.000 | 3414 | tags=44%, list=26%, signal=59% |
| 178 | ENERGY\_RESERVE\_METABOLIC\_PROCESS |  | 15 | 0.28 | 0.88 | 0.618 | 0.891 | 1.000 | 1252 | tags=20%, list=10%, signal=22% |
| 179 | REGULATION\_OF\_RNA\_METABOLIC\_PROCESS |  | 417 | 0.14 | 0.87 | 0.857 | 0.902 | 1.000 | 2961 | tags=24%, list=23%, signal=30% |
| 180 | SEXUAL\_REPRODUCTION |  | 109 | 0.17 | 0.87 | 0.723 | 0.910 | 1.000 | 3823 | tags=30%, list=29%, signal=42% |
| 181 | POSITIVE\_REGULATION\_OF\_CELL\_CYCLE |  | 15 | 0.27 | 0.87 | 0.651 | 0.907 | 1.000 | 507 | tags=20%, list=4%, signal=21% |
| 182 | REGULATION\_OF\_INTRACELLULAR\_TRANSPORT |  | 22 | 0.24 | 0.86 | 0.651 | 0.904 | 1.000 | 4213 | tags=45%, list=32%, signal=67% |
| 183 | RESPONSE\_TO\_RADIATION |  | 52 | 0.19 | 0.86 | 0.693 | 0.907 | 1.000 | 3257 | tags=31%, list=25%, signal=41% |
| 184 | SPLICEOSOME\_ASSEMBLY |  | 17 | 0.26 | 0.86 | 0.645 | 0.907 | 1.000 | 3345 | tags=35%, list=26%, signal=47% |
| 185 | REGULATION\_OF\_NUCLEOCYTOPLASMIC\_TRANSPORT |  | 19 | 0.25 | 0.86 | 0.666 | 0.904 | 1.000 | 1304 | tags=21%, list=10%, signal=23% |
| 186 | AEROBIC\_RESPIRATION |  | 15 | 0.27 | 0.85 | 0.665 | 0.916 | 1.000 | 2667 | tags=33%, list=20%, signal=42% |
| 187 | REGULATION\_OF\_TRANSCRIPTIONDNA\_DEPENDENT |  | 412 | 0.14 | 0.85 | 0.900 | 0.916 | 1.000 | 2961 | tags=24%, list=23%, signal=29% |
| 188 | TRANSMISSION\_OF\_NERVE\_IMPULSE |  | 167 | 0.15 | 0.85 | 0.803 | 0.917 | 1.000 | 2621 | tags=20%, list=20%, signal=25% |
| 189 | PHOSPHOINOSITIDE\_BIOSYNTHETIC\_PROCESS |  | 21 | 0.24 | 0.83 | 0.705 | 0.954 | 1.000 | 1088 | tags=19%, list=8%, signal=21% |
| 190 | PROTEOLYSIS |  | 170 | 0.15 | 0.82 | 0.854 | 0.953 | 1.000 | 3909 | tags=32%, list=30%, signal=46% |
| 191 | SYNAPTIC\_TRANSMISSION |  | 154 | 0.15 | 0.82 | 0.860 | 0.952 | 1.000 | 2596 | tags=19%, list=20%, signal=24% |
| 192 | CELL\_CYCLE\_ARREST\_GO\_0007050 |  | 52 | 0.18 | 0.82 | 0.774 | 0.952 | 1.000 | 3726 | tags=38%, list=28%, signal=54% |
| 193 | DEVELOPMENT\_OF\_PRIMARY\_SEXUAL\_CHARACTERISTICS |  | 25 | 0.22 | 0.82 | 0.743 | 0.949 | 1.000 | 3028 | tags=28%, list=23%, signal=36% |
| 194 | ESTABLISHMENT\_OF\_PROTEIN\_LOCALIZATION |  | 166 | 0.15 | 0.82 | 0.879 | 0.949 | 1.000 | 3320 | tags=26%, list=25%, signal=34% |
| 195 | MEMBRANE\_FUSION |  | 27 | 0.22 | 0.81 | 0.741 | 0.951 | 1.000 | 3578 | tags=37%, list=27%, signal=51% |
| 196 | CALCIUM\_INDEPENDENT\_CELL\_CELL\_ADHESION |  | 16 | 0.25 | 0.81 | 0.705 | 0.947 | 1.000 | 3731 | tags=38%, list=29%, signal=52% |
| 197 | EMBRYONIC\_DEVELOPMENT |  | 46 | 0.19 | 0.81 | 0.777 | 0.942 | 1.000 | 3028 | tags=26%, list=23%, signal=34% |
| 198 | MEMBRANE\_LIPID\_BIOSYNTHETIC\_PROCESS |  | 41 | 0.19 | 0.80 | 0.800 | 0.961 | 1.000 | 1589 | tags=17%, list=12%, signal=19% |
| 199 | HISTONE\_MODIFICATION |  | 21 | 0.23 | 0.80 | 0.731 | 0.957 | 1.000 | 3651 | tags=38%, list=28%, signal=53% |
| 200 | EXOCYTOSIS |  | 22 | 0.22 | 0.79 | 0.764 | 0.968 | 1.000 | 10208 | tags=100%, list=78%, signal=453% |
| 201 | RESPONSE\_TO\_UV |  | 22 | 0.22 | 0.77 | 0.780 | 0.987 | 1.000 | 3257 | tags=36%, list=25%, signal=48% |
| 202 | EPIDERMAL\_GROWTH\_FACTOR\_RECEPTOR\_SIGNALING\_PATHWAY |  | 18 | 0.23 | 0.77 | 0.754 | 0.983 | 1.000 | 4457 | tags=44%, list=34%, signal=67% |
| 203 | LIPID\_TRANSPORT |  | 27 | 0.21 | 0.76 | 0.830 | 0.992 | 1.000 | 1966 | tags=22%, list=15%, signal=26% |
| 204 | TRANSLATIONAL\_INITIATION |  | 33 | 0.19 | 0.76 | 0.837 | 0.988 | 1.000 | 3668 | tags=36%, list=28%, signal=50% |
| 205 | PHOSPHOLIPID\_BIOSYNTHETIC\_PROCESS |  | 35 | 0.19 | 0.76 | 0.864 | 0.992 | 1.000 | 1589 | tags=17%, list=12%, signal=19% |
| 206 | SENSORY\_PERCEPTION |  | 163 | 0.14 | 0.75 | 0.959 | 0.996 | 1.000 | 5181 | tags=43%, list=40%, signal=70% |
| 207 | CASPASE\_ACTIVATION |  | 24 | 0.20 | 0.75 | 0.826 | 0.995 | 1.000 | 3175 | tags=33%, list=24%, signal=44% |
| 208 | REPRODUCTION |  | 215 | 0.13 | 0.74 | 0.980 | 0.995 | 1.000 | 3841 | tags=29%, list=29%, signal=40% |
| 209 | CELLULAR\_CARBOHYDRATE\_METABOLIC\_PROCESS |  | 106 | 0.14 | 0.74 | 0.934 | 0.991 | 1.000 | 2828 | tags=21%, list=22%, signal=26% |
| 210 | GLYCEROPHOSPHOLIPID\_BIOSYNTHETIC\_PROCESS |  | 27 | 0.20 | 0.74 | 0.845 | 0.988 | 1.000 | 1088 | tags=15%, list=8%, signal=16% |
| 211 | REGULATION\_OF\_SECRETION |  | 35 | 0.18 | 0.73 | 0.877 | 0.995 | 1.000 | 2477 | tags=26%, list=19%, signal=32% |
| 212 | NEGATIVE\_REGULATION\_OF\_CELL\_CYCLE |  | 72 | 0.15 | 0.73 | 0.933 | 0.990 | 1.000 | 2367 | tags=21%, list=18%, signal=25% |
| 213 | JNK\_CASCADE |  | 44 | 0.17 | 0.72 | 0.896 | 0.993 | 1.000 | 4101 | tags=39%, list=31%, signal=56% |
| 214 | STRESS\_ACTIVATED\_PROTEIN\_KINASE\_SIGNALING\_PATHWAY |  | 45 | 0.17 | 0.72 | 0.915 | 0.990 | 1.000 | 4101 | tags=38%, list=31%, signal=55% |
| 215 | REGULATION\_OF\_CATABOLIC\_PROCESS |  | 15 | 0.23 | 0.72 | 0.824 | 0.988 | 1.000 | 4202 | tags=47%, list=32%, signal=69% |
| 216 | NEGATIVE\_REGULATION\_OF\_CELLULAR\_BIOSYNTHETIC\_PROCESS |  | 25 | 0.19 | 0.72 | 0.895 | 0.984 | 1.000 | 3251 | tags=28%, list=25%, signal=37% |
| 217 | LIPOPROTEIN\_METABOLIC\_PROCESS |  | 30 | 0.18 | 0.71 | 0.886 | 0.990 | 1.000 | 1966 | tags=20%, list=15%, signal=23% |
| 218 | HOMEOSTASIS\_OF\_NUMBER\_OF\_CELLS |  | 20 | 0.20 | 0.71 | 0.880 | 0.987 | 1.000 | 2445 | tags=25%, list=19%, signal=31% |
| 219 | REGULATION\_OF\_CELL\_ADHESION |  | 31 | 0.18 | 0.70 | 0.894 | 0.983 | 1.000 | 4151 | tags=42%, list=32%, signal=61% |
| 220 | GLUCOSE\_METABOLIC\_PROCESS |  | 27 | 0.18 | 0.70 | 0.889 | 0.982 | 1.000 | 4896 | tags=44%, list=37%, signal=71% |
| 221 | POSITIVE\_REGULATION\_OF\_HYDROLASE\_ACTIVITY |  | 45 | 0.16 | 0.68 | 0.957 | 0.993 | 1.000 | 3175 | tags=27%, list=24%, signal=35% |
| 222 | NEGATIVE\_REGULATION\_OF\_BIOSYNTHETIC\_PROCESS |  | 26 | 0.18 | 0.68 | 0.901 | 0.990 | 1.000 | 3251 | tags=27%, list=25%, signal=36% |
| 223 | ANION\_TRANSPORT |  | 27 | 0.18 | 0.68 | 0.917 | 0.988 | 1.000 | 1463 | tags=15%, list=11%, signal=17% |
| 224 | NEUROLOGICAL\_SYSTEM\_PROCESS |  | 328 | 0.11 | 0.68 | 1.000 | 0.984 | 1.000 | 4817 | tags=35%, list=37%, signal=54% |
| 225 | BIOGENIC\_AMINE\_METABOLIC\_PROCESS |  | 16 | 0.20 | 0.66 | 0.910 | 0.988 | 1.000 | 1589 | tags=19%, list=12%, signal=21% |
| 226 | PROTEIN\_AMINO\_ACID\_LIPIDATION |  | 21 | 0.18 | 0.63 | 0.949 | 1.000 | 1.000 | 1088 | tags=14%, list=8%, signal=16% |
| 227 | RHO\_PROTEIN\_SIGNAL\_TRANSDUCTION |  | 30 | 0.16 | 0.62 | 0.940 | 1.000 | 1.000 | 2770 | tags=23%, list=21%, signal=30% |
| 228 | FEEDING\_BEHAVIOR |  | 20 | 0.18 | 0.61 | 0.956 | 1.000 | 1.000 | 4995 | tags=45%, list=38%, signal=73% |
| 229 | ADENYLATE\_CYCLASE\_ACTIVATION |  | 18 | 0.18 | 0.61 | 0.956 | 0.996 | 1.000 | 4527 | tags=39%, list=35%, signal=59% |
| 230 | LIPOPROTEIN\_BIOSYNTHETIC\_PROCESS |  | 23 | 0.17 | 0.60 | 0.959 | 0.994 | 1.000 | 3942 | tags=35%, list=30%, signal=50% |
| 231 | TUBE\_DEVELOPMENT |  | 15 | 0.19 | 0.60 | 0.953 | 0.991 | 1.000 | 3763 | tags=33%, list=29%, signal=47% |
| 232 | NEGATIVE\_REGULATION\_OF\_TRANSLATION |  | 19 | 0.17 | 0.55 | 0.976 | 0.998 | 1.000 | 3251 | tags=26%, list=25%, signal=35% |
| 233 | REGULATION\_OF\_HEART\_CONTRACTION |  | 24 | 0.14 | 0.52 | 0.988 | 0.999 | 1.000 | 3931 | tags=33%, list=30%, signal=48% |
| 234 | DETECTION\_OF\_ABIOTIC\_STIMULUS |  | 16 | 0.15 | 0.50 | 0.985 | 0.996 | 1.000 | 69 | tags=6%, list=1%, signal=6% |
Table: Gene sets enriched in phenotype **na**[plain text format]****

  
