# Supplementary material for: Multiple-input multiple-output causal strategies for gene selection
Source: BMC Bioinformatics. 2011 Nov 25;12:458. doi: 10.1186/1471-2105-12-458 (PMC3323860; doi:10.1186/1471-2105-12-458)
Supplement: Additional file 3 — Archive containing the output files computed by the preranked GSEA for λ ∈ {0.6,0.7,0.8,0.9,1.0,2.0} (GSEA_MIMO_part2.zip). [file 1471-2105-12-458-S3.ZIP › mFS07_entrez_mimo.GseaPreranked.1316038907112/gsea_report_for_na_neg_1316038907112.html]

Report for na\_neg 1316038907112 [GSEA]

| GS  follow link to MSigDB | GS DETAILS | SIZE | ES | NES | NOM p-val | FDR q-val | FWER p-val | RANK AT MAX | LEADING EDGE || 1 | IMMUNE\_RESPONSE |  | 212 | -0.40 | -2.37 | 0.000 | 0.004 | 0.003 | 3289 | tags=46%, list=25%, signal=60% |
| 2 | DEFENSE\_RESPONSE |  | 238 | -0.38 | -2.30 | 0.000 | 0.004 | 0.006 | 3425 | tags=42%, list=26%, signal=56% |
| 3 | IMMUNE\_SYSTEM\_PROCESS |  | 298 | -0.36 | -2.29 | 0.000 | 0.003 | 0.006 | 2861 | tags=40%, list=22%, signal=50% |
| 4 | POSITIVE\_REGULATION\_OF\_IMMUNE\_RESPONSE |  | 24 | -0.57 | -2.15 | 0.000 | 0.010 | 0.029 | 2861 | tags=58%, list=22%, signal=75% |
| 5 | INFLAMMATORY\_RESPONSE |  | 115 | -0.39 | -2.10 | 0.000 | 0.013 | 0.050 | 3168 | tags=43%, list=24%, signal=56% |
| 6 | REGULATION\_OF\_IMMUNE\_RESPONSE |  | 28 | -0.52 | -2.09 | 0.000 | 0.013 | 0.058 | 4688 | tags=75%, list=36%, signal=117% |
| 7 | POSITIVE\_REGULATION\_OF\_MULTICELLULAR\_ORGANISMAL\_PROCESS |  | 56 | -0.45 | -2.08 | 0.000 | 0.012 | 0.062 | 3179 | tags=50%, list=24%, signal=66% |
| 8 | POSITIVE\_REGULATION\_OF\_IMMUNE\_SYSTEM\_PROCESS |  | 44 | -0.46 | -2.04 | 0.000 | 0.015 | 0.091 | 3179 | tags=50%, list=24%, signal=66% |
| 9 | CELLULAR\_DEFENSE\_RESPONSE |  | 54 | -0.44 | -2.04 | 0.000 | 0.014 | 0.093 | 3425 | tags=48%, list=26%, signal=65% |
| 10 | RESPONSE\_TO\_WOUNDING |  | 171 | -0.35 | -2.02 | 0.000 | 0.016 | 0.115 | 3173 | tags=40%, list=24%, signal=52% |
| 11 | REGULATION\_OF\_IMMUNE\_SYSTEM\_PROCESS |  | 57 | -0.42 | -1.97 | 0.000 | 0.022 | 0.170 | 3179 | tags=49%, list=24%, signal=65% |
| 12 | ADAPTIVE\_IMMUNE\_RESPONSE\_GO\_0002460 |  | 22 | -0.50 | -1.81 | 0.014 | 0.087 | 0.559 | 3168 | tags=50%, list=24%, signal=66% |
| 13 | HEMOPOIETIC\_OR\_LYMPHOID\_ORGAN\_DEVELOPMENT |  | 71 | -0.36 | -1.77 | 0.000 | 0.105 | 0.660 | 2822 | tags=39%, list=22%, signal=50% |
| 14 | ADAPTIVE\_IMMUNE\_RESPONSE |  | 23 | -0.47 | -1.77 | 0.006 | 0.099 | 0.664 | 3168 | tags=48%, list=24%, signal=63% |
| 15 | HEMOPOIESIS |  | 69 | -0.36 | -1.76 | 0.002 | 0.100 | 0.698 | 2822 | tags=39%, list=22%, signal=50% |
| 16 | REGULATION\_OF\_MULTICELLULAR\_ORGANISMAL\_PROCESS |  | 131 | -0.32 | -1.76 | 0.000 | 0.097 | 0.706 | 3444 | tags=41%, list=26%, signal=55% |
| 17 | RECEPTOR\_MEDIATED\_ENDOCYTOSIS |  | 31 | -0.44 | -1.72 | 0.009 | 0.125 | 0.813 | 2006 | tags=35%, list=15%, signal=42% |
| 18 | IMMUNE\_SYSTEM\_DEVELOPMENT |  | 75 | -0.34 | -1.72 | 0.000 | 0.119 | 0.818 | 2822 | tags=39%, list=22%, signal=49% |
| 19 | LYMPHOCYTE\_ACTIVATION |  | 54 | -0.36 | -1.71 | 0.000 | 0.125 | 0.842 | 3179 | tags=46%, list=24%, signal=61% |
| 20 | RESPONSE\_TO\_EXTERNAL\_STIMULUS |  | 278 | -0.27 | -1.70 | 0.000 | 0.127 | 0.859 | 3188 | tags=34%, list=24%, signal=44% |
| 21 | IMMUNE\_EFFECTOR\_PROCESS |  | 34 | -0.41 | -1.70 | 0.009 | 0.121 | 0.859 | 2769 | tags=50%, list=21%, signal=63% |
| 22 | JAK\_STAT\_CASCADE |  | 26 | -0.43 | -1.68 | 0.019 | 0.133 | 0.895 | 1598 | tags=35%, list=12%, signal=39% |
| 23 | LIPID\_CATABOLIC\_PROCESS |  | 34 | -0.40 | -1.66 | 0.011 | 0.148 | 0.927 | 3960 | tags=56%, list=30%, signal=80% |
| 24 | REGULATION\_OF\_DEFENSE\_RESPONSE |  | 15 | -0.51 | -1.66 | 0.027 | 0.143 | 0.928 | 3508 | tags=60%, list=27%, signal=82% |
| 25 | HUMORAL\_IMMUNE\_RESPONSE |  | 30 | -0.42 | -1.66 | 0.013 | 0.138 | 0.928 | 3188 | tags=53%, list=24%, signal=70% |
| 26 | POSITIVE\_REGULATION\_OF\_RESPONSE\_TO\_STIMULUS |  | 35 | -0.39 | -1.64 | 0.017 | 0.150 | 0.944 | 2861 | tags=46%, list=22%, signal=58% |
| 27 | REGULATION\_OF\_CELL\_DIFFERENTIATION |  | 48 | -0.36 | -1.63 | 0.007 | 0.157 | 0.959 | 4632 | tags=56%, list=35%, signal=87% |
| 28 | T\_CELL\_ACTIVATION |  | 39 | -0.38 | -1.62 | 0.020 | 0.158 | 0.962 | 3179 | tags=44%, list=24%, signal=57% |
| 29 | LEUKOCYTE\_DIFFERENTIATION |  | 34 | -0.39 | -1.61 | 0.004 | 0.160 | 0.966 | 2812 | tags=44%, list=21%, signal=56% |
| 30 | LEUKOCYTE\_ACTIVATION |  | 59 | -0.34 | -1.60 | 0.009 | 0.166 | 0.974 | 3179 | tags=44%, list=24%, signal=58% |
| 31 | CELL\_ACTIVATION |  | 64 | -0.33 | -1.58 | 0.007 | 0.191 | 0.989 | 3508 | tags=45%, list=27%, signal=62% |
| 32 | TRANSFORMING\_GROWTH\_FACTOR\_BETA\_RECEPTOR\_SIGNALING\_PATHWAY |  | 34 | -0.38 | -1.58 | 0.025 | 0.187 | 0.989 | 3125 | tags=44%, list=24%, signal=58% |
| 33 | ACTIN\_CYTOSKELETON\_ORGANIZATION\_AND\_BIOGENESIS |  | 90 | -0.30 | -1.56 | 0.013 | 0.213 | 0.997 | 2914 | tags=33%, list=22%, signal=43% |
| 34 | ENZYME\_LINKED\_RECEPTOR\_PROTEIN\_SIGNALING\_PATHWAY |  | 128 | -0.28 | -1.55 | 0.005 | 0.217 | 0.998 | 1860 | tags=24%, list=14%, signal=28% |
| 35 | LYMPHOCYTE\_DIFFERENTIATION |  | 23 | -0.42 | -1.53 | 0.034 | 0.239 | 0.999 | 3179 | tags=52%, list=24%, signal=69% |
| 36 | TRANSMEMBRANE\_RECEPTOR\_PROTEIN\_SERINE\_THREONINE\_KINASE\_SIGNALING\_PATHWAY |  | 42 | -0.35 | -1.52 | 0.022 | 0.247 | 0.999 | 3125 | tags=40%, list=24%, signal=53% |
| 37 | B\_CELL\_ACTIVATION |  | 17 | -0.44 | -1.52 | 0.048 | 0.249 | 0.999 | 3168 | tags=59%, list=24%, signal=78% |
| 38 | POSITIVE\_REGULATION\_OF\_CELL\_DIFFERENTIATION |  | 21 | -0.41 | -1.49 | 0.051 | 0.276 | 0.999 | 4632 | tags=71%, list=35%, signal=110% |
| 39 | NEGATIVE\_REGULATION\_OF\_SIGNAL\_TRANSDUCTION |  | 31 | -0.37 | -1.48 | 0.048 | 0.287 | 1.000 | 2751 | tags=35%, list=21%, signal=45% |
| 40 | MULTI\_ORGANISM\_PROCESS |  | 137 | -0.26 | -1.48 | 0.005 | 0.284 | 1.000 | 3762 | tags=42%, list=29%, signal=59% |
| 41 | CELLULAR\_LIPID\_CATABOLIC\_PROCESS |  | 31 | -0.36 | -1.48 | 0.051 | 0.278 | 1.000 | 3960 | tags=52%, list=30%, signal=74% |
| 42 | REGULATION\_OF\_RESPONSE\_TO\_STIMULUS |  | 49 | -0.33 | -1.48 | 0.024 | 0.274 | 1.000 | 3983 | tags=53%, list=30%, signal=76% |
| 43 | PROTEIN\_AMINO\_ACID\_N\_LINKED\_GLYCOSYLATION |  | 27 | -0.37 | -1.47 | 0.045 | 0.282 | 1.000 | 2433 | tags=37%, list=19%, signal=45% |
| 44 | CELL\_SUBSTRATE\_ADHESION |  | 36 | -0.35 | -1.47 | 0.041 | 0.279 | 1.000 | 2506 | tags=36%, list=19%, signal=45% |
| 45 | POSITIVE\_REGULATION\_OF\_SIGNAL\_TRANSDUCTION |  | 97 | -0.28 | -1.47 | 0.009 | 0.274 | 1.000 | 2344 | tags=31%, list=18%, signal=37% |
| 46 | MAINTENANCE\_OF\_LOCALIZATION |  | 21 | -0.41 | -1.46 | 0.052 | 0.280 | 1.000 | 2565 | tags=38%, list=20%, signal=47% |
| 47 | PEPTIDYL\_TYROSINE\_MODIFICATION |  | 23 | -0.40 | -1.46 | 0.051 | 0.285 | 1.000 | 1467 | tags=26%, list=11%, signal=29% |
| 48 | INNATE\_IMMUNE\_RESPONSE |  | 19 | -0.41 | -1.45 | 0.060 | 0.292 | 1.000 | 4851 | tags=74%, list=37%, signal=117% |
| 49 | REGULATION\_OF\_PROTEIN\_AMINO\_ACID\_PHOSPHORYLATION |  | 23 | -0.39 | -1.45 | 0.065 | 0.288 | 1.000 | 2506 | tags=35%, list=19%, signal=43% |
| 50 | POSITIVE\_REGULATION\_OF\_PHOSPHATE\_METABOLIC\_PROCESS |  | 23 | -0.39 | -1.45 | 0.048 | 0.286 | 1.000 | 1535 | tags=30%, list=12%, signal=34% |
| 51 | REGULATION\_OF\_ANGIOGENESIS |  | 24 | -0.39 | -1.44 | 0.063 | 0.284 | 1.000 | 2550 | tags=46%, list=19%, signal=57% |
| 52 | REGULATION\_OF\_LYMPHOCYTE\_ACTIVATION |  | 31 | -0.36 | -1.44 | 0.059 | 0.283 | 1.000 | 3179 | tags=45%, list=24%, signal=60% |
| 53 | CATION\_HOMEOSTASIS |  | 94 | -0.27 | -1.44 | 0.023 | 0.283 | 1.000 | 3188 | tags=36%, list=24%, signal=47% |
| 54 | CYTOKINE\_AND\_CHEMOKINE\_MEDIATED\_SIGNALING\_PATHWAY |  | 19 | -0.41 | -1.43 | 0.065 | 0.290 | 1.000 | 2355 | tags=37%, list=18%, signal=45% |
| 55 | DETECTION\_OF\_STIMULUS |  | 36 | -0.34 | -1.42 | 0.053 | 0.298 | 1.000 | 4855 | tags=56%, list=37%, signal=88% |
| 56 | REGULATION\_OF\_SIGNAL\_TRANSDUCTION |  | 173 | -0.24 | -1.41 | 0.022 | 0.310 | 1.000 | 3575 | tags=36%, list=27%, signal=49% |
| 57 | SMALL\_GTPASE\_MEDIATED\_SIGNAL\_TRANSDUCTION |  | 77 | -0.28 | -1.41 | 0.024 | 0.308 | 1.000 | 3485 | tags=40%, list=27%, signal=55% |
| 58 | CELLULAR\_CATION\_HOMEOSTASIS |  | 91 | -0.27 | -1.41 | 0.037 | 0.308 | 1.000 | 3188 | tags=36%, list=24%, signal=48% |
| 59 | REGULATION\_OF\_CYTOSKELETON\_ORGANIZATION\_AND\_BIOGENESIS |  | 26 | -0.36 | -1.40 | 0.069 | 0.313 | 1.000 | 2506 | tags=35%, list=19%, signal=43% |
| 60 | MESODERM\_DEVELOPMENT |  | 22 | -0.38 | -1.40 | 0.088 | 0.311 | 1.000 | 4053 | tags=50%, list=31%, signal=72% |
| 61 | PROTEIN\_KINASE\_CASCADE |  | 239 | -0.23 | -1.40 | 0.010 | 0.308 | 1.000 | 2353 | tags=26%, list=18%, signal=32% |
| 62 | PROTEIN\_AMINO\_ACID\_PHOSPHORYLATION |  | 231 | -0.23 | -1.40 | 0.005 | 0.311 | 1.000 | 2934 | tags=29%, list=22%, signal=37% |
| 63 | WOUND\_HEALING |  | 49 | -0.31 | -1.40 | 0.056 | 0.308 | 1.000 | 3698 | tags=41%, list=28%, signal=57% |
| 64 | COAGULATION |  | 41 | -0.32 | -1.39 | 0.056 | 0.307 | 1.000 | 3698 | tags=41%, list=28%, signal=58% |
| 65 | REGULATION\_OF\_T\_CELL\_ACTIVATION |  | 25 | -0.36 | -1.39 | 0.063 | 0.305 | 1.000 | 3179 | tags=44%, list=24%, signal=58% |
| 66 | BLOOD\_COAGULATION |  | 41 | -0.32 | -1.39 | 0.079 | 0.302 | 1.000 | 3698 | tags=41%, list=28%, signal=58% |
| 67 | FATTY\_ACID\_METABOLIC\_PROCESS |  | 56 | -0.30 | -1.39 | 0.057 | 0.303 | 1.000 | 4094 | tags=50%, list=31%, signal=72% |
| 68 | RESPONSE\_TO\_OTHER\_ORGANISM |  | 69 | -0.28 | -1.39 | 0.064 | 0.299 | 1.000 | 2144 | tags=30%, list=16%, signal=36% |
| 69 | POSITIVE\_REGULATION\_OF\_LYMPHOCYTE\_ACTIVATION |  | 23 | -0.37 | -1.39 | 0.071 | 0.295 | 1.000 | 3179 | tags=43%, list=24%, signal=57% |
| 70 | TRANSMEMBRANE\_RECEPTOR\_PROTEIN\_TYROSINE\_KINASE\_SIGNALING\_PATHWAY |  | 76 | -0.28 | -1.38 | 0.046 | 0.297 | 1.000 | 1372 | tags=21%, list=10%, signal=23% |
| 71 | PROTEIN\_COMPLEX\_ASSEMBLY |  | 157 | -0.24 | -1.37 | 0.020 | 0.309 | 1.000 | 2833 | tags=30%, list=22%, signal=38% |
| 72 | ACTIN\_POLYMERIZATION\_AND\_OR\_DEPOLYMERIZATION |  | 20 | -0.38 | -1.37 | 0.118 | 0.319 | 1.000 | 2301 | tags=30%, list=18%, signal=36% |
| 73 | FEMALE\_PREGNANCY |  | 42 | -0.31 | -1.35 | 0.101 | 0.343 | 1.000 | 3911 | tags=50%, list=30%, signal=71% |
| 74 | CELL\_MATRIX\_ADHESION |  | 35 | -0.32 | -1.35 | 0.084 | 0.343 | 1.000 | 2506 | tags=34%, list=19%, signal=42% |
| 75 | ACTIN\_FILAMENT\_BASED\_PROCESS |  | 99 | -0.26 | -1.34 | 0.046 | 0.354 | 1.000 | 2914 | tags=30%, list=22%, signal=39% |
| 76 | POSITIVE\_REGULATION\_OF\_CYTOKINE\_BIOSYNTHETIC\_PROCESS |  | 21 | -0.37 | -1.34 | 0.116 | 0.356 | 1.000 | 2817 | tags=43%, list=22%, signal=55% |
| 77 | GROWTH |  | 59 | -0.28 | -1.34 | 0.089 | 0.352 | 1.000 | 4083 | tags=42%, list=31%, signal=61% |
| 78 | POSITIVE\_REGULATION\_OF\_PROTEIN\_AMINO\_ACID\_PHOSPHORYLATION |  | 15 | -0.41 | -1.33 | 0.108 | 0.357 | 1.000 | 1535 | tags=33%, list=12%, signal=38% |
| 79 | CYTOKINE\_PRODUCTION |  | 61 | -0.29 | -1.33 | 0.055 | 0.356 | 1.000 | 2817 | tags=34%, list=22%, signal=44% |
| 80 | PEPTIDYL\_TYROSINE\_PHOSPHORYLATION |  | 21 | -0.36 | -1.33 | 0.127 | 0.361 | 1.000 | 1467 | tags=24%, list=11%, signal=27% |
| 81 | GLYCOPROTEIN\_METABOLIC\_PROCESS |  | 82 | -0.26 | -1.32 | 0.053 | 0.370 | 1.000 | 4141 | tags=43%, list=32%, signal=62% |
| 82 | RESPONSE\_TO\_VIRUS |  | 45 | -0.29 | -1.30 | 0.107 | 0.399 | 1.000 | 3151 | tags=42%, list=24%, signal=55% |
| 83 | MUSCLE\_DEVELOPMENT |  | 85 | -0.26 | -1.30 | 0.071 | 0.401 | 1.000 | 3295 | tags=39%, list=25%, signal=52% |
| 84 | REGULATION\_OF\_CELL\_PROLIFERATION |  | 275 | -0.21 | -1.30 | 0.013 | 0.400 | 1.000 | 2526 | tags=25%, list=19%, signal=31% |
| 85 | MONOCARBOXYLIC\_ACID\_METABOLIC\_PROCESS |  | 77 | -0.26 | -1.29 | 0.068 | 0.405 | 1.000 | 4117 | tags=45%, list=31%, signal=66% |
| 86 | RAS\_PROTEIN\_SIGNAL\_TRANSDUCTION |  | 55 | -0.28 | -1.28 | 0.115 | 0.430 | 1.000 | 3414 | tags=40%, list=26%, signal=54% |
| 87 | REGULATION\_OF\_BODY\_FLUID\_LEVELS |  | 55 | -0.27 | -1.28 | 0.118 | 0.434 | 1.000 | 3763 | tags=40%, list=29%, signal=56% |
| 88 | REGULATION\_OF\_ANATOMICAL\_STRUCTURE\_MORPHOGENESIS |  | 17 | -0.37 | -1.27 | 0.142 | 0.435 | 1.000 | 3301 | tags=35%, list=25%, signal=47% |
| 89 | HEMOSTASIS |  | 46 | -0.29 | -1.27 | 0.105 | 0.447 | 1.000 | 3698 | tags=39%, list=28%, signal=54% |
| 90 | AMINE\_TRANSPORT |  | 36 | -0.31 | -1.26 | 0.142 | 0.450 | 1.000 | 2514 | tags=28%, list=19%, signal=34% |
| 91 | AMINO\_ACID\_TRANSPORT |  | 25 | -0.33 | -1.26 | 0.162 | 0.445 | 1.000 | 445 | tags=20%, list=3%, signal=21% |
| 92 | POSITIVE\_REGULATION\_OF\_CELL\_PROLIFERATION |  | 129 | -0.23 | -1.26 | 0.087 | 0.451 | 1.000 | 1447 | tags=20%, list=11%, signal=22% |
| 93 | POSITIVE\_REGULATION\_OF\_PHOSPHORYLATION |  | 21 | -0.35 | -1.26 | 0.179 | 0.451 | 1.000 | 1535 | tags=29%, list=12%, signal=32% |
| 94 | PROTEIN\_AMINO\_ACID\_DEPHOSPHORYLATION |  | 60 | -0.27 | -1.26 | 0.136 | 0.447 | 1.000 | 1359 | tags=20%, list=10%, signal=22% |
| 95 | ANATOMICAL\_STRUCTURE\_FORMATION |  | 52 | -0.27 | -1.25 | 0.136 | 0.449 | 1.000 | 2550 | tags=33%, list=19%, signal=40% |
| 96 | BEHAVIOR |  | 136 | -0.22 | -1.25 | 0.111 | 0.452 | 1.000 | 3693 | tags=35%, list=28%, signal=49% |
| 97 | CELL\_RECOGNITION |  | 16 | -0.38 | -1.25 | 0.205 | 0.447 | 1.000 | 4391 | tags=56%, list=34%, signal=85% |
| 98 | DEPHOSPHORYLATION |  | 67 | -0.25 | -1.25 | 0.129 | 0.454 | 1.000 | 1359 | tags=19%, list=10%, signal=22% |
| 99 | POSITIVE\_REGULATION\_OF\_TRANSLATION |  | 28 | -0.31 | -1.24 | 0.166 | 0.452 | 1.000 | 2817 | tags=39%, list=22%, signal=50% |
| 100 | REGULATION\_OF\_BLOOD\_PRESSURE |  | 22 | -0.34 | -1.24 | 0.179 | 0.448 | 1.000 | 3744 | tags=41%, list=29%, signal=57% |
| 101 | REGULATION\_OF\_I\_KAPPAB\_KINASE\_NF\_KAPPAB\_CASCADE |  | 72 | -0.25 | -1.24 | 0.123 | 0.456 | 1.000 | 3643 | tags=42%, list=28%, signal=57% |
| 102 | REGULATION\_OF\_ORGANELLE\_ORGANIZATION\_AND\_BIOGENESIS |  | 35 | -0.30 | -1.24 | 0.179 | 0.453 | 1.000 | 2506 | tags=31%, list=19%, signal=39% |
| 103 | PHOSPHOLIPID\_METABOLIC\_PROCESS |  | 63 | -0.25 | -1.23 | 0.127 | 0.458 | 1.000 | 2898 | tags=33%, list=22%, signal=43% |
| 104 | ANGIOGENESIS |  | 44 | -0.28 | -1.23 | 0.157 | 0.460 | 1.000 | 2550 | tags=34%, list=19%, signal=42% |
| 105 | RESPONSE\_TO\_DRUG |  | 21 | -0.34 | -1.23 | 0.189 | 0.461 | 1.000 | 2825 | tags=43%, list=22%, signal=55% |
| 106 | POSITIVE\_REGULATION\_OF\_CELLULAR\_PROTEIN\_METABOLIC\_PROCESS |  | 61 | -0.25 | -1.23 | 0.144 | 0.459 | 1.000 | 2312 | tags=30%, list=18%, signal=36% |
| 107 | MUSCLE\_CELL\_DIFFERENTIATION |  | 21 | -0.34 | -1.23 | 0.198 | 0.456 | 1.000 | 3295 | tags=48%, list=25%, signal=64% |
| 108 | G\_PROTEIN\_SIGNALING\_COUPLED\_TO\_CAMP\_NUCLEOTIDE\_SECOND\_MESSENGER |  | 62 | -0.26 | -1.22 | 0.162 | 0.469 | 1.000 | 1978 | tags=21%, list=15%, signal=25% |
| 109 | POSITIVE\_REGULATION\_OF\_PROTEIN\_METABOLIC\_PROCESS |  | 63 | -0.25 | -1.22 | 0.157 | 0.466 | 1.000 | 2552 | tags=32%, list=19%, signal=39% |
| 110 | ACTIVATION\_OF\_NF\_KAPPAB\_TRANSCRIPTION\_FACTOR |  | 15 | -0.38 | -1.22 | 0.190 | 0.462 | 1.000 | 4113 | tags=60%, list=31%, signal=87% |
| 111 | NEGATIVE\_REGULATION\_OF\_TRANSCRIPTION |  | 166 | -0.21 | -1.22 | 0.107 | 0.459 | 1.000 | 2759 | tags=28%, list=21%, signal=35% |
| 112 | CAMP\_MEDIATED\_SIGNALING |  | 63 | -0.25 | -1.22 | 0.152 | 0.457 | 1.000 | 1978 | tags=21%, list=15%, signal=24% |
| 113 | REGULATION\_OF\_PROTEIN\_IMPORT\_INTO\_NUCLEUS |  | 15 | -0.37 | -1.22 | 0.204 | 0.455 | 1.000 | 1322 | tags=27%, list=10%, signal=30% |
| 114 | GENERATION\_OF\_NEURONS |  | 65 | -0.25 | -1.22 | 0.132 | 0.453 | 1.000 | 3428 | tags=34%, list=26%, signal=46% |
| 115 | PHOSPHORYLATION |  | 262 | -0.20 | -1.22 | 0.084 | 0.449 | 1.000 | 2934 | tags=28%, list=22%, signal=35% |
| 116 | ICOSANOID\_METABOLIC\_PROCESS |  | 16 | -0.38 | -1.22 | 0.226 | 0.447 | 1.000 | 3125 | tags=44%, list=24%, signal=57% |
| 117 | PROTEIN\_OLIGOMERIZATION |  | 37 | -0.30 | -1.22 | 0.158 | 0.446 | 1.000 | 2833 | tags=32%, list=22%, signal=41% |
| 118 | LIPID\_METABOLIC\_PROCESS |  | 283 | -0.19 | -1.21 | 0.088 | 0.456 | 1.000 | 4205 | tags=41%, list=32%, signal=60% |
| 119 | POSITIVE\_REGULATION\_OF\_TRANSFERASE\_ACTIVITY |  | 71 | -0.25 | -1.21 | 0.138 | 0.460 | 1.000 | 2514 | tags=27%, list=19%, signal=33% |
| 120 | NEURON\_DIFFERENTIATION |  | 58 | -0.26 | -1.21 | 0.158 | 0.458 | 1.000 | 3428 | tags=33%, list=26%, signal=44% |
| 121 | RESPONSE\_TO\_BACTERIUM |  | 22 | -0.33 | -1.20 | 0.222 | 0.459 | 1.000 | 4037 | tags=45%, list=31%, signal=66% |
| 122 | DEVELOPMENTAL\_MATURATION |  | 18 | -0.35 | -1.20 | 0.205 | 0.458 | 1.000 | 2909 | tags=39%, list=22%, signal=50% |
| 123 | REGULATION\_OF\_PROTEIN\_METABOLIC\_PROCESS |  | 150 | -0.21 | -1.20 | 0.106 | 0.462 | 1.000 | 2552 | tags=27%, list=19%, signal=33% |
| 124 | ORGAN\_MORPHOGENESIS |  | 131 | -0.21 | -1.20 | 0.116 | 0.459 | 1.000 | 1528 | tags=19%, list=12%, signal=21% |
| 125 | DEFENSE\_RESPONSE\_TO\_BACTERIUM |  | 16 | -0.36 | -1.19 | 0.210 | 0.465 | 1.000 | 3762 | tags=44%, list=29%, signal=61% |
| 126 | POSITIVE\_REGULATION\_OF\_I\_KAPPAB\_KINASE\_NF\_KAPPAB\_CASCADE |  | 67 | -0.24 | -1.19 | 0.180 | 0.475 | 1.000 | 2344 | tags=30%, list=18%, signal=36% |
| 127 | BONE\_REMODELING |  | 28 | -0.30 | -1.17 | 0.241 | 0.510 | 1.000 | 2751 | tags=32%, list=21%, signal=41% |
| 128 | POSITIVE\_REGULATION\_OF\_SECRETION |  | 18 | -0.34 | -1.17 | 0.248 | 0.508 | 1.000 | 4911 | tags=67%, list=38%, signal=107% |
| 129 | POSITIVE\_REGULATION\_OF\_T\_CELL\_ACTIVATION |  | 20 | -0.33 | -1.17 | 0.256 | 0.515 | 1.000 | 3179 | tags=40%, list=24%, signal=53% |
| 130 | I\_KAPPAB\_KINASE\_NF\_KAPPAB\_CASCADE |  | 88 | -0.22 | -1.16 | 0.179 | 0.520 | 1.000 | 2976 | tags=33%, list=23%, signal=42% |
| 131 | MYELOID\_CELL\_DIFFERENTIATION |  | 35 | -0.28 | -1.16 | 0.237 | 0.516 | 1.000 | 2822 | tags=31%, list=22%, signal=40% |
| 132 | CYTOKINE\_BIOSYNTHETIC\_PROCESS |  | 34 | -0.28 | -1.16 | 0.224 | 0.518 | 1.000 | 2817 | tags=35%, list=22%, signal=45% |
| 133 | SKELETAL\_DEVELOPMENT |  | 91 | -0.22 | -1.16 | 0.204 | 0.519 | 1.000 | 3037 | tags=32%, list=23%, signal=41% |
| 134 | STRIATED\_MUSCLE\_DEVELOPMENT |  | 36 | -0.28 | -1.16 | 0.239 | 0.518 | 1.000 | 3444 | tags=44%, list=26%, signal=60% |
| 135 | REGULATION\_OF\_MAPKKK\_CASCADE |  | 19 | -0.34 | -1.16 | 0.273 | 0.516 | 1.000 | 2290 | tags=32%, list=17%, signal=38% |
| 136 | PROTEIN\_PROCESSING |  | 41 | -0.27 | -1.16 | 0.224 | 0.514 | 1.000 | 3950 | tags=39%, list=30%, signal=56% |
| 137 | NEGATIVE\_REGULATION\_OF\_CELL\_PROLIFERATION |  | 145 | -0.20 | -1.16 | 0.150 | 0.511 | 1.000 | 2523 | tags=26%, list=19%, signal=31% |
| 138 | RESPONSE\_TO\_BIOTIC\_STIMULUS |  | 103 | -0.22 | -1.15 | 0.191 | 0.519 | 1.000 | 2144 | tags=26%, list=16%, signal=31% |
| 139 | GENERATION\_OF\_PRECURSOR\_METABOLITES\_AND\_ENERGY |  | 120 | -0.21 | -1.15 | 0.182 | 0.520 | 1.000 | 3096 | tags=31%, list=24%, signal=40% |
| 140 | REGULATION\_OF\_MAP\_KINASE\_ACTIVITY |  | 56 | -0.25 | -1.15 | 0.231 | 0.519 | 1.000 | 1895 | tags=27%, list=14%, signal=31% |
| 141 | REGULATION\_OF\_CELLULAR\_PROTEIN\_METABOLIC\_PROCESS |  | 139 | -0.20 | -1.15 | 0.177 | 0.520 | 1.000 | 2552 | tags=26%, list=19%, signal=32% |
| 142 | VASCULATURE\_DEVELOPMENT |  | 50 | -0.25 | -1.15 | 0.224 | 0.517 | 1.000 | 2550 | tags=30%, list=19%, signal=37% |
| 143 | NEURON\_DEVELOPMENT |  | 49 | -0.25 | -1.14 | 0.219 | 0.541 | 1.000 | 3428 | tags=33%, list=26%, signal=44% |
| 144 | ION\_HOMEOSTASIS |  | 112 | -0.21 | -1.13 | 0.193 | 0.546 | 1.000 | 3188 | tags=32%, list=24%, signal=42% |
| 145 | NEGATIVE\_REGULATION\_OF\_METABOLIC\_PROCESS |  | 232 | -0.18 | -1.13 | 0.182 | 0.555 | 1.000 | 2592 | tags=25%, list=20%, signal=31% |
| 146 | LOCOMOTORY\_BEHAVIOR |  | 84 | -0.22 | -1.12 | 0.252 | 0.560 | 1.000 | 2270 | tags=25%, list=17%, signal=30% |
| 147 | NEGATIVE\_REGULATION\_OF\_NUCLEOBASENUCLEOSIDENUCLEOTIDE\_AND\_NUCLEIC\_ACID\_METABOLIC\_PROCESS |  | 185 | -0.19 | -1.12 | 0.187 | 0.557 | 1.000 | 2592 | tags=26%, list=20%, signal=33% |
| 148 | REGULATION\_OF\_DEVELOPMENTAL\_PROCESS |  | 387 | -0.17 | -1.12 | 0.133 | 0.560 | 1.000 | 3301 | tags=31%, list=25%, signal=41% |
| 149 | CELL\_MATURATION |  | 16 | -0.34 | -1.12 | 0.305 | 0.556 | 1.000 | 2909 | tags=38%, list=22%, signal=48% |
| 150 | POST\_TRANSLATIONAL\_PROTEIN\_MODIFICATION |  | 409 | -0.17 | -1.12 | 0.126 | 0.559 | 1.000 | 2934 | tags=26%, list=22%, signal=32% |
| 151 | MEMBRANE\_ORGANIZATION\_AND\_BIOGENESIS |  | 124 | -0.20 | -1.12 | 0.224 | 0.558 | 1.000 | 3861 | tags=38%, list=29%, signal=53% |
| 152 | NEGATIVE\_REGULATION\_OF\_RNA\_METABOLIC\_PROCESS |  | 114 | -0.20 | -1.12 | 0.248 | 0.560 | 1.000 | 2759 | tags=28%, list=21%, signal=35% |
| 153 | GLYCOPROTEIN\_BIOSYNTHETIC\_PROCESS |  | 67 | -0.23 | -1.12 | 0.248 | 0.557 | 1.000 | 4141 | tags=42%, list=32%, signal=61% |
| 154 | POSITIVE\_REGULATION\_OF\_PROTEIN\_MODIFICATION\_PROCESS |  | 24 | -0.30 | -1.11 | 0.280 | 0.558 | 1.000 | 1535 | tags=25%, list=12%, signal=28% |
| 155 | ORGANIC\_ACID\_METABOLIC\_PROCESS |  | 162 | -0.19 | -1.11 | 0.235 | 0.567 | 1.000 | 4342 | tags=42%, list=33%, signal=62% |
| 156 | CARBOXYLIC\_ACID\_METABOLIC\_PROCESS |  | 160 | -0.19 | -1.11 | 0.230 | 0.569 | 1.000 | 4342 | tags=43%, list=33%, signal=63% |
| 157 | REGULATION\_OF\_MYELOID\_CELL\_DIFFERENTIATION |  | 19 | -0.32 | -1.11 | 0.300 | 0.567 | 1.000 | 4597 | tags=58%, list=35%, signal=89% |
| 158 | TISSUE\_REMODELING |  | 29 | -0.28 | -1.11 | 0.307 | 0.565 | 1.000 | 2751 | tags=31%, list=21%, signal=39% |
| 159 | ACTIVATION\_OF\_MAPK\_ACTIVITY |  | 33 | -0.27 | -1.10 | 0.302 | 0.563 | 1.000 | 1841 | tags=27%, list=14%, signal=32% |
| 160 | AMINO\_ACID\_DERIVATIVE\_METABOLIC\_PROCESS |  | 23 | -0.30 | -1.10 | 0.299 | 0.560 | 1.000 | 3983 | tags=48%, list=30%, signal=69% |
| 161 | CYTOKINE\_METABOLIC\_PROCESS |  | 35 | -0.27 | -1.10 | 0.290 | 0.558 | 1.000 | 2817 | tags=34%, list=22%, signal=44% |
| 162 | PROTEIN\_AUTOPROCESSING |  | 24 | -0.29 | -1.10 | 0.305 | 0.555 | 1.000 | 5117 | tags=58%, list=39%, signal=96% |
| 163 | NEGATIVE\_REGULATION\_OF\_TRANSCRIPTION\_DNA\_DEPENDENT |  | 114 | -0.20 | -1.10 | 0.257 | 0.556 | 1.000 | 2759 | tags=28%, list=21%, signal=35% |
| 164 | CYTOKINE\_SECRETION |  | 15 | -0.35 | -1.10 | 0.331 | 0.561 | 1.000 | 3465 | tags=47%, list=26%, signal=63% |
| 165 | GLYCEROPHOSPHOLIPID\_METABOLIC\_PROCESS |  | 39 | -0.26 | -1.10 | 0.326 | 0.563 | 1.000 | 4591 | tags=51%, list=35%, signal=79% |
| 166 | NEURITE\_DEVELOPMENT |  | 41 | -0.25 | -1.09 | 0.318 | 0.569 | 1.000 | 3428 | tags=32%, list=26%, signal=43% |
| 167 | ELECTRON\_TRANSPORT\_GO\_0006118 |  | 50 | -0.24 | -1.09 | 0.287 | 0.570 | 1.000 | 2203 | tags=26%, list=17%, signal=31% |
| 168 | NEGATIVE\_REGULATION\_OF\_TRANSCRIPTION\_FROM\_RNA\_POLYMERASE\_II\_PROMOTER |  | 76 | -0.22 | -1.09 | 0.284 | 0.567 | 1.000 | 2759 | tags=29%, list=21%, signal=36% |
| 169 | PROTEIN\_AMINO\_ACID\_AUTOPHOSPHORYLATION |  | 24 | -0.29 | -1.09 | 0.350 | 0.565 | 1.000 | 5117 | tags=58%, list=39%, signal=96% |
| 170 | DETECTION\_OF\_EXTERNAL\_STIMULUS |  | 18 | -0.32 | -1.09 | 0.331 | 0.565 | 1.000 | 4028 | tags=39%, list=31%, signal=56% |
| 171 | NEGATIVE\_REGULATION\_OF\_CELLULAR\_METABOLIC\_PROCESS |  | 229 | -0.18 | -1.09 | 0.221 | 0.566 | 1.000 | 2592 | tags=25%, list=20%, signal=30% |
| 172 | PEPTIDYL\_AMINO\_ACID\_MODIFICATION |  | 47 | -0.24 | -1.09 | 0.304 | 0.565 | 1.000 | 2506 | tags=28%, list=19%, signal=34% |
| 173 | HORMONE\_METABOLIC\_PROCESS |  | 29 | -0.27 | -1.09 | 0.312 | 0.563 | 1.000 | 3439 | tags=45%, list=26%, signal=61% |
| 174 | POSITIVE\_REGULATION\_OF\_DEVELOPMENTAL\_PROCESS |  | 197 | -0.18 | -1.08 | 0.277 | 0.564 | 1.000 | 4203 | tags=43%, list=32%, signal=62% |
| 175 | PHAGOCYTOSIS |  | 16 | -0.33 | -1.08 | 0.327 | 0.574 | 1.000 | 4189 | tags=56%, list=32%, signal=83% |
| 176 | POSITIVE\_REGULATION\_OF\_CELLULAR\_METABOLIC\_PROCESS |  | 196 | -0.18 | -1.08 | 0.260 | 0.571 | 1.000 | 2822 | tags=27%, list=22%, signal=34% |
| 177 | POSITIVE\_REGULATION\_OF\_METABOLIC\_PROCESS |  | 201 | -0.18 | -1.08 | 0.273 | 0.572 | 1.000 | 2822 | tags=27%, list=22%, signal=34% |
| 178 | MEMBRANE\_LIPID\_METABOLIC\_PROCESS |  | 85 | -0.21 | -1.07 | 0.318 | 0.583 | 1.000 | 3546 | tags=36%, list=27%, signal=50% |
| 179 | POSITIVE\_REGULATION\_OF\_TRANSCRIPTION |  | 124 | -0.19 | -1.07 | 0.298 | 0.581 | 1.000 | 2822 | tags=27%, list=22%, signal=34% |
| 180 | CELL\_PROLIFERATION\_GO\_0008283 |  | 466 | -0.16 | -1.07 | 0.246 | 0.578 | 1.000 | 2593 | tags=24%, list=20%, signal=28% |
| 181 | POSITIVE\_REGULATION\_OF\_CATALYTIC\_ACTIVITY |  | 139 | -0.19 | -1.07 | 0.273 | 0.578 | 1.000 | 2514 | tags=23%, list=19%, signal=28% |
| 182 | FATTY\_ACID\_OXIDATION |  | 17 | -0.32 | -1.07 | 0.336 | 0.578 | 1.000 | 3482 | tags=47%, list=27%, signal=64% |
| 183 | CELL\_MIGRATION |  | 82 | -0.21 | -1.07 | 0.344 | 0.581 | 1.000 | 2526 | tags=24%, list=19%, signal=30% |
| 184 | REGULATION\_OF\_BIOLOGICAL\_QUALITY |  | 364 | -0.17 | -1.07 | 0.266 | 0.582 | 1.000 | 3763 | tags=31%, list=29%, signal=43% |
| 185 | ACTIN\_FILAMENT\_ORGANIZATION |  | 21 | -0.29 | -1.06 | 0.378 | 0.581 | 1.000 | 2506 | tags=33%, list=19%, signal=41% |
| 186 | PROTEIN\_SECRETION |  | 28 | -0.27 | -1.06 | 0.351 | 0.584 | 1.000 | 4348 | tags=46%, list=33%, signal=69% |
| 187 | MYOBLAST\_DIFFERENTIATION |  | 16 | -0.32 | -1.06 | 0.374 | 0.583 | 1.000 | 3295 | tags=50%, list=25%, signal=67% |
| 188 | AMINO\_ACID\_CATABOLIC\_PROCESS |  | 23 | -0.29 | -1.05 | 0.373 | 0.597 | 1.000 | 2063 | tags=30%, list=16%, signal=36% |
| 189 | AMINO\_ACID\_METABOLIC\_PROCESS |  | 73 | -0.21 | -1.05 | 0.343 | 0.602 | 1.000 | 2239 | tags=26%, list=17%, signal=31% |
| 190 | ANTI\_APOPTOSIS |  | 107 | -0.19 | -1.05 | 0.333 | 0.601 | 1.000 | 2139 | tags=25%, list=16%, signal=30% |
| 191 | AXONOGENESIS |  | 33 | -0.26 | -1.05 | 0.397 | 0.598 | 1.000 | 3428 | tags=33%, list=26%, signal=45% |
| 192 | CELLULAR\_LIPID\_METABOLIC\_PROCESS |  | 220 | -0.17 | -1.05 | 0.342 | 0.605 | 1.000 | 4115 | tags=39%, list=31%, signal=56% |
| 193 | NEUROGENESIS |  | 75 | -0.21 | -1.04 | 0.398 | 0.624 | 1.000 | 3428 | tags=32%, list=26%, signal=43% |
| 194 | RESPONSE\_TO\_CHEMICAL\_STIMULUS |  | 271 | -0.17 | -1.04 | 0.349 | 0.627 | 1.000 | 1731 | tags=18%, list=13%, signal=20% |
| 195 | VITAMIN\_METABOLIC\_PROCESS |  | 15 | -0.32 | -1.04 | 0.386 | 0.625 | 1.000 | 4438 | tags=60%, list=34%, signal=91% |
| 196 | POSITIVE\_REGULATION\_OF\_TRANSCRIPTION\_FACTOR\_ACTIVITY |  | 17 | -0.30 | -1.04 | 0.396 | 0.623 | 1.000 | 4113 | tags=53%, list=31%, signal=77% |
| 197 | REGULATION\_OF\_TRANSCRIPTION |  | 498 | -0.16 | -1.03 | 0.337 | 0.622 | 1.000 | 2822 | tags=25%, list=22%, signal=31% |
| 198 | POSITIVE\_REGULATION\_OF\_MAP\_KINASE\_ACTIVITY |  | 39 | -0.25 | -1.03 | 0.398 | 0.625 | 1.000 | 1841 | tags=26%, list=14%, signal=30% |
| 199 | MACROMOLECULE\_BIOSYNTHETIC\_PROCESS |  | 267 | -0.17 | -1.03 | 0.385 | 0.624 | 1.000 | 2906 | tags=27%, list=22%, signal=33% |
| 200 | MAPKKK\_CASCADE\_GO\_0000165 |  | 90 | -0.20 | -1.03 | 0.408 | 0.632 | 1.000 | 2353 | tags=23%, list=18%, signal=28% |
| 201 | ORGANIC\_ACID\_TRANSPORT |  | 39 | -0.24 | -1.03 | 0.385 | 0.631 | 1.000 | 960 | tags=18%, list=7%, signal=19% |
| 202 | DETECTION\_OF\_STIMULUS\_INVOLVED\_IN\_SENSORY\_PERCEPTION |  | 15 | -0.31 | -1.02 | 0.437 | 0.635 | 1.000 | 9009 | tags=100%, list=69%, signal=320% |
| 203 | CARBOXYLIC\_ACID\_TRANSPORT |  | 39 | -0.24 | -1.02 | 0.412 | 0.635 | 1.000 | 960 | tags=18%, list=7%, signal=19% |
| 204 | NERVOUS\_SYSTEM\_DEVELOPMENT |  | 328 | -0.16 | -1.02 | 0.397 | 0.637 | 1.000 | 4076 | tags=34%, list=31%, signal=48% |
| 205 | CELLULAR\_COMPONENT\_ASSEMBLY |  | 272 | -0.17 | -1.02 | 0.368 | 0.640 | 1.000 | 2909 | tags=26%, list=22%, signal=33% |
| 206 | NEGATIVE\_REGULATION\_OF\_CELL\_DIFFERENTIATION |  | 24 | -0.27 | -1.02 | 0.434 | 0.645 | 1.000 | 3037 | tags=29%, list=23%, signal=38% |
| 207 | REGULATION\_OF\_CYTOKINE\_BIOSYNTHETIC\_PROCESS |  | 31 | -0.25 | -1.01 | 0.411 | 0.646 | 1.000 | 2817 | tags=32%, list=22%, signal=41% |
| 208 | TISSUE\_DEVELOPMENT |  | 126 | -0.18 | -1.01 | 0.431 | 0.645 | 1.000 | 1698 | tags=19%, list=13%, signal=22% |
| 209 | CELLULAR\_PROTEIN\_COMPLEX\_ASSEMBLY |  | 28 | -0.26 | -1.00 | 0.455 | 0.665 | 1.000 | 2701 | tags=29%, list=21%, signal=36% |
| 210 | TRANSLATION |  | 149 | -0.18 | -1.00 | 0.478 | 0.662 | 1.000 | 2704 | tags=27%, list=21%, signal=33% |
| 211 | AMINE\_CATABOLIC\_PROCESS |  | 25 | -0.26 | -1.00 | 0.433 | 0.667 | 1.000 | 2063 | tags=28%, list=16%, signal=33% |
| 212 | AMINO\_ACID\_AND\_DERIVATIVE\_METABOLIC\_PROCESS |  | 96 | -0.19 | -0.99 | 0.458 | 0.681 | 1.000 | 2250 | tags=24%, list=17%, signal=29% |
| 213 | NEGATIVE\_REGULATION\_OF\_DEVELOPMENTAL\_PROCESS |  | 177 | -0.17 | -0.99 | 0.470 | 0.681 | 1.000 | 3301 | tags=31%, list=25%, signal=41% |
| 214 | REGULATION\_OF\_JNK\_ACTIVITY |  | 18 | -0.29 | -0.99 | 0.457 | 0.679 | 1.000 | 1841 | tags=28%, list=14%, signal=32% |
| 215 | RHYTHMIC\_PROCESS |  | 23 | -0.26 | -0.99 | 0.480 | 0.687 | 1.000 | 1998 | tags=26%, list=15%, signal=31% |
| 216 | NITROGEN\_COMPOUND\_CATABOLIC\_PROCESS |  | 27 | -0.26 | -0.99 | 0.453 | 0.691 | 1.000 | 2063 | tags=26%, list=16%, signal=31% |
| 217 | HEART\_DEVELOPMENT |  | 33 | -0.24 | -0.98 | 0.481 | 0.693 | 1.000 | 3983 | tags=39%, list=30%, signal=56% |
| 218 | SODIUM\_ION\_TRANSPORT |  | 17 | -0.29 | -0.98 | 0.460 | 0.699 | 1.000 | 5080 | tags=59%, list=39%, signal=96% |
| 219 | CHEMICAL\_HOMEOSTASIS |  | 136 | -0.18 | -0.98 | 0.494 | 0.708 | 1.000 | 3188 | tags=29%, list=24%, signal=38% |
| 220 | POSITIVE\_REGULATION\_OF\_DNA\_BINDING |  | 18 | -0.28 | -0.98 | 0.479 | 0.707 | 1.000 | 4905 | tags=61%, list=37%, signal=98% |
| 221 | CELL\_CELL\_ADHESION |  | 72 | -0.19 | -0.96 | 0.514 | 0.733 | 1.000 | 4774 | tags=49%, list=36%, signal=76% |
| 222 | MACROMOLECULAR\_COMPLEX\_ASSEMBLY |  | 254 | -0.16 | -0.96 | 0.566 | 0.734 | 1.000 | 2833 | tags=25%, list=22%, signal=32% |
| 223 | POSITIVE\_REGULATION\_OF\_NUCLEOBASENUCLEOSIDENUCLEOTIDE\_AND\_NUCLEIC\_ACID\_METABOLIC\_PROCESS |  | 134 | -0.17 | -0.96 | 0.522 | 0.738 | 1.000 | 3015 | tags=27%, list=23%, signal=35% |
| 224 | ANATOMICAL\_STRUCTURE\_MORPHOGENESIS |  | 336 | -0.15 | -0.96 | 0.609 | 0.739 | 1.000 | 3328 | tags=28%, list=25%, signal=37% |
| 225 | CELLULAR\_HOMEOSTASIS |  | 121 | -0.17 | -0.96 | 0.561 | 0.743 | 1.000 | 3228 | tags=30%, list=25%, signal=39% |
| 226 | SKELETAL\_MUSCLE\_DEVELOPMENT |  | 28 | -0.24 | -0.95 | 0.540 | 0.758 | 1.000 | 3444 | tags=43%, list=26%, signal=58% |
| 227 | POSITIVE\_REGULATION\_OF\_TRANSCRIPTION\_FROM\_RNA\_POLYMERASE\_II\_PROMOTER |  | 60 | -0.20 | -0.95 | 0.551 | 0.760 | 1.000 | 4597 | tags=47%, list=35%, signal=72% |
| 228 | AXON\_GUIDANCE |  | 18 | -0.28 | -0.94 | 0.525 | 0.771 | 1.000 | 2960 | tags=33%, list=23%, signal=43% |
| 229 | HOMEOSTATIC\_PROCESS |  | 179 | -0.16 | -0.94 | 0.615 | 0.772 | 1.000 | 3228 | tags=28%, list=25%, signal=37% |
| 230 | ACTIVATION\_OF\_PROTEIN\_KINASE\_ACTIVITY |  | 23 | -0.25 | -0.93 | 0.516 | 0.786 | 1.000 | 4428 | tags=39%, list=34%, signal=59% |
| 231 | REGULATION\_OF\_PHOSPHORYLATION |  | 42 | -0.21 | -0.93 | 0.559 | 0.786 | 1.000 | 2506 | tags=26%, list=19%, signal=32% |
| 232 | REGULATION\_OF\_G\_PROTEIN\_COUPLED\_RECEPTOR\_PROTEIN\_SIGNALING\_PATHWAY |  | 23 | -0.25 | -0.93 | 0.546 | 0.786 | 1.000 | 1097 | tags=17%, list=8%, signal=19% |
| 233 | POSITIVE\_REGULATION\_OF\_CELLULAR\_COMPONENT\_ORGANIZATION\_AND\_BIOGENESIS |  | 28 | -0.24 | -0.93 | 0.560 | 0.786 | 1.000 | 4155 | tags=43%, list=32%, signal=63% |
| 234 | SECRETION\_BY\_CELL |  | 100 | -0.17 | -0.92 | 0.628 | 0.805 | 1.000 | 4590 | tags=43%, list=35%, signal=66% |
| 235 | REGULATION\_OF\_TRANSCRIPTIONDNA\_DEPENDENT |  | 412 | -0.14 | -0.92 | 0.778 | 0.816 | 1.000 | 2822 | tags=25%, list=22%, signal=30% |
| 236 | CYCLIC\_NUCLEOTIDE\_MEDIATED\_SIGNALING |  | 97 | -0.17 | -0.91 | 0.638 | 0.815 | 1.000 | 1203 | tags=12%, list=9%, signal=14% |
| 237 | REGULATION\_OF\_BINDING |  | 46 | -0.20 | -0.91 | 0.583 | 0.816 | 1.000 | 2552 | tags=28%, list=19%, signal=35% |
| 238 | SULFUR\_METABOLIC\_PROCESS |  | 30 | -0.23 | -0.91 | 0.604 | 0.824 | 1.000 | 2827 | tags=30%, list=22%, signal=38% |
| 239 | RESPONSE\_TO\_NUTRIENT |  | 17 | -0.27 | -0.91 | 0.573 | 0.822 | 1.000 | 2305 | tags=29%, list=18%, signal=36% |
| 240 | RESPONSE\_TO\_OXIDATIVE\_STRESS |  | 38 | -0.21 | -0.89 | 0.654 | 0.854 | 1.000 | 1579 | tags=24%, list=12%, signal=27% |
| 241 | G\_PROTEIN\_SIGNALING\_COUPLED\_TO\_CYCLIC\_NUCLEOTIDE\_SECOND\_MESSENGER |  | 96 | -0.17 | -0.89 | 0.696 | 0.851 | 1.000 | 1203 | tags=13%, list=9%, signal=14% |
| 242 | REGULATION\_OF\_PROTEIN\_SECRETION |  | 19 | -0.26 | -0.89 | 0.620 | 0.853 | 1.000 | 2769 | tags=32%, list=21%, signal=40% |
| 243 | REGULATION\_OF\_PROTEIN\_MODIFICATION\_PROCESS |  | 37 | -0.21 | -0.89 | 0.634 | 0.858 | 1.000 | 1535 | tags=19%, list=12%, signal=21% |
| 244 | REGULATION\_OF\_TRANSLATIONAL\_INITIATION |  | 25 | -0.23 | -0.88 | 0.628 | 0.865 | 1.000 | 1146 | tags=20%, list=9%, signal=22% |
| 245 | RESPONSE\_TO\_NUTRIENT\_LEVELS |  | 27 | -0.22 | -0.88 | 0.626 | 0.873 | 1.000 | 2345 | tags=26%, list=18%, signal=32% |
| 246 | VESICLE\_MEDIATED\_TRANSPORT |  | 174 | -0.15 | -0.88 | 0.790 | 0.876 | 1.000 | 3880 | tags=33%, list=30%, signal=47% |
| 247 | EPIDERMIS\_DEVELOPMENT |  | 66 | -0.18 | -0.87 | 0.700 | 0.877 | 1.000 | 1698 | tags=20%, list=13%, signal=23% |
| 248 | REGULATION\_OF\_CELLULAR\_COMPONENT\_ORGANIZATION\_AND\_BIOGENESIS |  | 102 | -0.17 | -0.87 | 0.771 | 0.880 | 1.000 | 3617 | tags=31%, list=28%, signal=43% |
| 249 | ENDOSOME\_TRANSPORT |  | 22 | -0.24 | -0.87 | 0.636 | 0.880 | 1.000 | 3861 | tags=41%, list=29%, signal=58% |
| 250 | CENTRAL\_NERVOUS\_SYSTEM\_DEVELOPMENT |  | 105 | -0.16 | -0.87 | 0.781 | 0.882 | 1.000 | 4387 | tags=39%, list=34%, signal=58% |
| 251 | GOLGI\_VESICLE\_TRANSPORT |  | 42 | -0.20 | -0.87 | 0.681 | 0.881 | 1.000 | 4294 | tags=43%, list=33%, signal=64% |
| 252 | AMINE\_METABOLIC\_PROCESS |  | 128 | -0.16 | -0.86 | 0.766 | 0.883 | 1.000 | 2971 | tags=26%, list=23%, signal=33% |
| 253 | CELL\_CELL\_SIGNALING |  | 372 | -0.13 | -0.86 | 0.892 | 0.882 | 1.000 | 4225 | tags=34%, list=32%, signal=49% |
| 254 | POSITIVE\_REGULATION\_OF\_CASPASE\_ACTIVITY |  | 28 | -0.22 | -0.86 | 0.677 | 0.880 | 1.000 | 1872 | tags=25%, list=14%, signal=29% |
| 255 | CELLULAR\_MORPHOGENESIS\_DURING\_DIFFERENTIATION |  | 38 | -0.20 | -0.85 | 0.701 | 0.901 | 1.000 | 3428 | tags=29%, list=26%, signal=39% |
| 256 | SPHINGOLIPID\_METABOLIC\_PROCESS |  | 23 | -0.23 | -0.85 | 0.703 | 0.898 | 1.000 | 3228 | tags=35%, list=25%, signal=46% |
| 257 | ECTODERM\_DEVELOPMENT |  | 75 | -0.17 | -0.85 | 0.764 | 0.906 | 1.000 | 1447 | tags=17%, list=11%, signal=19% |
| 258 | REGULATION\_OF\_TRANSCRIPTION\_FACTOR\_ACTIVITY |  | 30 | -0.21 | -0.83 | 0.733 | 0.926 | 1.000 | 4357 | tags=47%, list=33%, signal=70% |
| 259 | EXTRACELLULAR\_STRUCTURE\_ORGANIZATION\_AND\_BIOGENESIS |  | 23 | -0.22 | -0.83 | 0.690 | 0.924 | 1.000 | 3444 | tags=39%, list=26%, signal=53% |
| 260 | T\_CELL\_PROLIFERATION |  | 17 | -0.25 | -0.83 | 0.691 | 0.923 | 1.000 | 2817 | tags=35%, list=22%, signal=45% |
| 261 | PHOSPHOINOSITIDE\_METABOLIC\_PROCESS |  | 25 | -0.22 | -0.83 | 0.707 | 0.923 | 1.000 | 4591 | tags=52%, list=35%, signal=80% |
| 262 | REGULATION\_OF\_TRANSLATION |  | 76 | -0.17 | -0.82 | 0.825 | 0.931 | 1.000 | 2896 | tags=26%, list=22%, signal=34% |
| 263 | POSITIVE\_REGULATION\_OF\_TRANSCRIPTIONDNA\_DEPENDENT |  | 105 | -0.15 | -0.82 | 0.880 | 0.932 | 1.000 | 4632 | tags=42%, list=35%, signal=64% |
| 264 | NITROGEN\_COMPOUND\_METABOLIC\_PROCESS |  | 141 | -0.15 | -0.82 | 0.872 | 0.932 | 1.000 | 2971 | tags=25%, list=23%, signal=32% |
| 265 | INSULIN\_RECEPTOR\_SIGNALING\_PATHWAY |  | 16 | -0.24 | -0.82 | 0.699 | 0.929 | 1.000 | 3644 | tags=38%, list=28%, signal=52% |
| 266 | REGULATION\_OF\_GROWTH |  | 48 | -0.18 | -0.82 | 0.792 | 0.932 | 1.000 | 4285 | tags=40%, list=33%, signal=59% |
| 267 | POSITIVE\_REGULATION\_OF\_BINDING |  | 19 | -0.23 | -0.81 | 0.725 | 0.940 | 1.000 | 4113 | tags=47%, list=31%, signal=69% |
| 268 | SECOND\_MESSENGER\_MEDIATED\_SIGNALING |  | 139 | -0.14 | -0.80 | 0.915 | 0.950 | 1.000 | 2092 | tags=16%, list=16%, signal=19% |
| 269 | REGULATION\_OF\_DNA\_BINDING |  | 36 | -0.19 | -0.80 | 0.777 | 0.949 | 1.000 | 2552 | tags=28%, list=19%, signal=34% |
| 270 | PROTEIN\_LOCALIZATION |  | 184 | -0.14 | -0.80 | 0.947 | 0.952 | 1.000 | 3575 | tags=28%, list=27%, signal=38% |
| 271 | RESPONSE\_TO\_EXTRACELLULAR\_STIMULUS |  | 29 | -0.20 | -0.80 | 0.812 | 0.949 | 1.000 | 2345 | tags=24%, list=18%, signal=29% |
| 272 | POSITIVE\_REGULATION\_OF\_JNK\_ACTIVITY |  | 16 | -0.24 | -0.79 | 0.753 | 0.953 | 1.000 | 1841 | tags=25%, list=14%, signal=29% |
| 273 | REGULATION\_OF\_MUSCLE\_CONTRACTION |  | 18 | -0.23 | -0.79 | 0.760 | 0.954 | 1.000 | 1828 | tags=28%, list=14%, signal=32% |
| 274 | REPRODUCTIVE\_PROCESS |  | 133 | -0.14 | -0.79 | 0.921 | 0.951 | 1.000 | 3922 | tags=35%, list=30%, signal=49% |
| 275 | AMINE\_BIOSYNTHETIC\_PROCESS |  | 15 | -0.24 | -0.78 | 0.751 | 0.963 | 1.000 | 371 | tags=13%, list=3%, signal=14% |
| 276 | CARBOHYDRATE\_METABOLIC\_PROCESS |  | 152 | -0.14 | -0.78 | 0.938 | 0.962 | 1.000 | 4141 | tags=34%, list=32%, signal=49% |
| 277 | POSITIVE\_REGULATION\_OF\_RNA\_METABOLIC\_PROCESS |  | 107 | -0.14 | -0.77 | 0.925 | 0.964 | 1.000 | 4632 | tags=41%, list=35%, signal=63% |
| 278 | G\_PROTEIN\_SIGNALING\_COUPLED\_TO\_IP3\_SECOND\_MESSENGERPHOSPHOLIPASE\_C\_ACTIVATING |  | 39 | -0.18 | -0.77 | 0.856 | 0.963 | 1.000 | 2564 | tags=23%, list=20%, signal=29% |
| 279 | CARBOHYDRATE\_BIOSYNTHETIC\_PROCESS |  | 35 | -0.18 | -0.76 | 0.854 | 0.977 | 1.000 | 4117 | tags=37%, list=31%, signal=54% |
| 280 | METAL\_ION\_TRANSPORT |  | 102 | -0.14 | -0.76 | 0.937 | 0.977 | 1.000 | 5080 | tags=46%, list=39%, signal=75% |
| 281 | REGULATION\_OF\_CELL\_MIGRATION |  | 23 | -0.21 | -0.75 | 0.814 | 0.978 | 1.000 | 4688 | tags=43%, list=36%, signal=68% |
| 282 | G\_PROTEIN\_COUPLED\_RECEPTOR\_PROTEIN\_SIGNALING\_PATHWAY |  | 300 | -0.12 | -0.75 | 0.997 | 0.977 | 1.000 | 4795 | tags=37%, list=37%, signal=57% |
| 283 | NEGATIVE\_REGULATION\_OF\_CELLULAR\_COMPONENT\_ORGANIZATION\_AND\_BIOGENESIS |  | 26 | -0.20 | -0.75 | 0.859 | 0.977 | 1.000 | 1443 | tags=15%, list=11%, signal=17% |
| 284 | BRAIN\_DEVELOPMENT |  | 39 | -0.18 | -0.74 | 0.861 | 0.983 | 1.000 | 3952 | tags=38%, list=30%, signal=55% |
| 285 | NEGATIVE\_REGULATION\_OF\_CELLULAR\_PROTEIN\_METABOLIC\_PROCESS |  | 41 | -0.17 | -0.74 | 0.896 | 0.982 | 1.000 | 2861 | tags=24%, list=22%, signal=31% |
| 286 | POSITIVE\_REGULATION\_OF\_TRANSPORT |  | 18 | -0.21 | -0.73 | 0.835 | 0.989 | 1.000 | 4851 | tags=56%, list=37%, signal=88% |
| 287 | G\_PROTEIN\_SIGNALING\_ADENYLATE\_CYCLASE\_ACTIVATING\_PATHWAY |  | 24 | -0.19 | -0.73 | 0.861 | 0.991 | 1.000 | 1203 | tags=13%, list=9%, signal=14% |
| 288 | PROTEIN\_POLYMERIZATION |  | 17 | -0.21 | -0.73 | 0.854 | 0.988 | 1.000 | 361 | tags=12%, list=3%, signal=12% |
| 289 | DI\_\_\_TRI\_VALENT\_INORGANIC\_CATION\_TRANSPORT |  | 27 | -0.18 | -0.71 | 0.902 | 0.999 | 1.000 | 1108 | tags=15%, list=8%, signal=16% |
| 290 | CATION\_TRANSPORT |  | 130 | -0.13 | -0.71 | 0.984 | 0.997 | 1.000 | 4800 | tags=42%, list=37%, signal=65% |
| 291 | SECRETORY\_PATHWAY |  | 72 | -0.14 | -0.71 | 0.951 | 0.995 | 1.000 | 4590 | tags=42%, list=35%, signal=64% |
| 292 | NEGATIVE\_REGULATION\_OF\_GROWTH |  | 35 | -0.17 | -0.70 | 0.908 | 0.997 | 1.000 | 4285 | tags=40%, list=33%, signal=59% |
| 293 | EXCRETION |  | 35 | -0.17 | -0.69 | 0.921 | 1.000 | 1.000 | 2247 | tags=20%, list=17%, signal=24% |
| 294 | SECRETION |  | 157 | -0.12 | -0.69 | 0.993 | 0.998 | 1.000 | 4911 | tags=43%, list=38%, signal=67% |
| 295 | ION\_TRANSPORT |  | 165 | -0.12 | -0.69 | 0.995 | 0.995 | 1.000 | 4800 | tags=41%, list=37%, signal=63% |
| 296 | NEGATIVE\_REGULATION\_OF\_PROTEIN\_METABOLIC\_PROCESS |  | 44 | -0.15 | -0.69 | 0.945 | 0.994 | 1.000 | 2861 | tags=23%, list=22%, signal=29% |
| 297 | POTASSIUM\_ION\_TRANSPORT |  | 52 | -0.15 | -0.69 | 0.951 | 0.991 | 1.000 | 5051 | tags=44%, list=39%, signal=72% |
| 298 | PHOSPHOINOSITIDE\_MEDIATED\_SIGNALING |  | 42 | -0.16 | -0.69 | 0.919 | 0.989 | 1.000 | 2564 | tags=21%, list=20%, signal=27% |
| 299 | CARBOHYDRATE\_CATABOLIC\_PROCESS |  | 20 | -0.19 | -0.68 | 0.905 | 0.987 | 1.000 | 4117 | tags=35%, list=31%, signal=51% |
| 300 | CELLULAR\_CARBOHYDRATE\_CATABOLIC\_PROCESS |  | 20 | -0.19 | -0.68 | 0.876 | 0.984 | 1.000 | 4117 | tags=35%, list=31%, signal=51% |
| 301 | REGULATION\_OF\_CYTOKINE\_PRODUCTION |  | 21 | -0.19 | -0.68 | 0.891 | 0.982 | 1.000 | 3168 | tags=29%, list=24%, signal=38% |
| 302 | PATTERN\_SPECIFICATION\_PROCESS |  | 27 | -0.17 | -0.67 | 0.924 | 0.985 | 1.000 | 5525 | tags=59%, list=42%, signal=102% |
| 303 | ESTABLISHMENT\_AND\_OR\_MAINTENANCE\_OF\_CELL\_POLARITY |  | 19 | -0.19 | -0.67 | 0.910 | 0.982 | 1.000 | 1557 | tags=16%, list=12%, signal=18% |
| 304 | LIPID\_HOMEOSTASIS |  | 15 | -0.21 | -0.67 | 0.901 | 0.983 | 1.000 | 811 | tags=13%, list=6%, signal=14% |
| 305 | NEGATIVE\_REGULATION\_OF\_MULTICELLULAR\_ORGANISMAL\_PROCESS |  | 27 | -0.17 | -0.66 | 0.914 | 0.983 | 1.000 | 2149 | tags=22%, list=16%, signal=27% |
| 306 | INORGANIC\_ANION\_TRANSPORT |  | 16 | -0.20 | -0.66 | 0.909 | 0.982 | 1.000 | 588 | tags=13%, list=4%, signal=13% |
| 307 | NUCLEOTIDE\_EXCISION\_REPAIR |  | 19 | -0.18 | -0.64 | 0.936 | 0.986 | 1.000 | 2205 | tags=21%, list=17%, signal=25% |
| 308 | AMINO\_SUGAR\_METABOLIC\_PROCESS |  | 15 | -0.17 | -0.57 | 0.954 | 1.000 | 1.000 | 4422 | tags=40%, list=34%, signal=60% |
| 309 | CALCIUM\_ION\_TRANSPORT |  | 23 | -0.15 | -0.57 | 0.966 | 1.000 | 1.000 | 1108 | tags=13%, list=8%, signal=14% |
| 310 | FEMALE\_GAMETE\_GENERATION |  | 15 | -0.17 | -0.55 | 0.983 | 1.000 | 1.000 | 10852 | tags=100%, list=83%, signal=584% |
| 311 | DETECTION\_OF\_ABIOTIC\_STIMULUS |  | 16 | -0.16 | -0.54 | 0.983 | 1.000 | 1.000 | 5346 | tags=50%, list=41%, signal=84% |
| 312 | RESPONSE\_TO\_LIGHT\_STIMULUS |  | 40 | -0.12 | -0.54 | 0.991 | 1.000 | 1.000 | 2552 | tags=20%, list=19%, signal=25% |
| 313 | REGULATION\_OF\_CELL\_GROWTH |  | 39 | -0.12 | -0.51 | 0.998 | 1.000 | 1.000 | 4285 | tags=33%, list=33%, signal=49% |
| 314 | REGULATION\_OF\_ACTION\_POTENTIAL |  | 16 | -0.15 | -0.50 | 0.992 | 1.000 | 1.000 | 3636 | tags=31%, list=28%, signal=43% |
| 315 | MONOVALENT\_INORGANIC\_CATION\_TRANSPORT |  | 83 | -0.10 | -0.50 | 1.000 | 0.997 | 1.000 | 5080 | tags=42%, list=39%, signal=68% |
Table: Gene sets enriched in phenotype **na**[plain text format]****

  
