# Supplementary material for: Multiple-input multiple-output causal strategies for gene selection
Source: BMC Bioinformatics. 2011 Nov 25;12:458. doi: 10.1186/1471-2105-12-458 (PMC3323860; doi:10.1186/1471-2105-12-458)
Supplement: Additional file 3 — Archive containing the output files computed by the preranked GSEA for λ ∈ {0.6,0.7,0.8,0.9,1.0,2.0} (GSEA_MIMO_part2.zip). [file 1471-2105-12-458-S3.ZIP › mFS08_entrez_mimo.GseaPreranked.1316039081375/gsea_report_for_na_pos_1316039081375.html]

Report for na\_pos 1316039081375 [GSEA]

| GS  follow link to MSigDB | GS DETAILS | SIZE | ES | NES | NOM p-val | FDR q-val | FWER p-val | RANK AT MAX | LEADING EDGE || 1 | M\_PHASE\_OF\_MITOTIC\_CELL\_CYCLE |  | 72 | 0.56 | 2.66 | 0.000 | 0.000 | 0.000 | 2726 | tags=58%, list=21%, signal=73% |
| 2 | CELL\_CYCLE\_PROCESS |  | 169 | 0.48 | 2.66 | 0.000 | 0.000 | 0.000 | 2377 | tags=47%, list=18%, signal=56% |
| 3 | M\_PHASE |  | 98 | 0.53 | 2.66 | 0.000 | 0.000 | 0.000 | 2328 | tags=51%, list=18%, signal=62% |
| 4 | MITOTIC\_CELL\_CYCLE |  | 134 | 0.49 | 2.65 | 0.000 | 0.000 | 0.000 | 2377 | tags=48%, list=18%, signal=58% |
| 5 | MITOSIS |  | 70 | 0.56 | 2.64 | 0.000 | 0.000 | 0.000 | 2726 | tags=57%, list=21%, signal=72% |
| 6 | CELL\_CYCLE\_PHASE |  | 152 | 0.46 | 2.51 | 0.000 | 0.000 | 0.000 | 2377 | tags=45%, list=18%, signal=54% |
| 7 | DNA\_REPLICATION |  | 97 | 0.48 | 2.47 | 0.000 | 0.000 | 0.001 | 2851 | tags=47%, list=22%, signal=60% |
| 8 | DNA\_METABOLIC\_PROCESS |  | 240 | 0.41 | 2.41 | 0.000 | 0.000 | 0.001 | 3227 | tags=47%, list=25%, signal=61% |
| 9 | CELL\_CYCLE\_GO\_0007049 |  | 277 | 0.41 | 2.40 | 0.000 | 0.000 | 0.001 | 2414 | tags=41%, list=18%, signal=49% |
| 10 | SISTER\_CHROMATID\_SEGREGATION |  | 16 | 0.74 | 2.39 | 0.000 | 0.000 | 0.001 | 912 | tags=56%, list=7%, signal=60% |
| 11 | MITOTIC\_SISTER\_CHROMATID\_SEGREGATION |  | 15 | 0.75 | 2.38 | 0.000 | 0.000 | 0.001 | 912 | tags=60%, list=7%, signal=64% |
| 12 | CELL\_CYCLE\_CHECKPOINT\_GO\_0000075 |  | 45 | 0.53 | 2.33 | 0.000 | 0.000 | 0.001 | 1965 | tags=53%, list=15%, signal=63% |
| 13 | DNA\_REPAIR |  | 118 | 0.44 | 2.29 | 0.000 | 0.000 | 0.003 | 2973 | tags=48%, list=23%, signal=62% |
| 14 | CHROMOSOME\_SEGREGATION |  | 28 | 0.59 | 2.28 | 0.000 | 0.000 | 0.004 | 912 | tags=46%, list=7%, signal=50% |
| 15 | DNA\_DEPENDENT\_DNA\_REPLICATION |  | 52 | 0.51 | 2.28 | 0.000 | 0.000 | 0.004 | 2992 | tags=54%, list=23%, signal=70% |
| 16 | REGULATION\_OF\_MITOSIS |  | 33 | 0.57 | 2.26 | 0.000 | 0.000 | 0.004 | 1056 | tags=45%, list=8%, signal=49% |
| 17 | RNA\_SPLICING |  | 74 | 0.46 | 2.24 | 0.000 | 0.000 | 0.006 | 3325 | tags=53%, list=25%, signal=70% |
| 18 | RESPONSE\_TO\_DNA\_DAMAGE\_STIMULUS |  | 153 | 0.41 | 2.22 | 0.000 | 0.000 | 0.009 | 2973 | tags=46%, list=23%, signal=59% |
| 19 | NUCLEOTIDE\_BIOSYNTHETIC\_PROCESS |  | 17 | 0.66 | 2.17 | 0.000 | 0.001 | 0.013 | 1272 | tags=53%, list=10%, signal=59% |
| 20 | RESPONSE\_TO\_ENDOGENOUS\_STIMULUS |  | 182 | 0.38 | 2.16 | 0.000 | 0.001 | 0.017 | 3404 | tags=47%, list=26%, signal=62% |
| 21 | RNA\_PROCESSING |  | 138 | 0.40 | 2.12 | 0.000 | 0.001 | 0.027 | 3325 | tags=49%, list=25%, signal=65% |
| 22 | DNA\_INTEGRITY\_CHECKPOINT |  | 22 | 0.59 | 2.09 | 0.000 | 0.002 | 0.047 | 1965 | tags=59%, list=15%, signal=69% |
| 23 | MICROTUBULE\_CYTOSKELETON\_ORGANIZATION\_AND\_BIOGENESIS |  | 31 | 0.53 | 2.08 | 0.000 | 0.002 | 0.051 | 2712 | tags=55%, list=21%, signal=69% |
| 24 | DNA\_REPLICATION\_INITIATION |  | 15 | 0.65 | 2.06 | 0.000 | 0.002 | 0.061 | 2675 | tags=80%, list=20%, signal=100% |
| 25 | MITOTIC\_CELL\_CYCLE\_CHECKPOINT |  | 19 | 0.59 | 2.03 | 0.004 | 0.003 | 0.102 | 1946 | tags=53%, list=15%, signal=62% |
| 26 | PROTEIN\_FOLDING |  | 55 | 0.44 | 2.01 | 0.000 | 0.004 | 0.122 | 3184 | tags=51%, list=24%, signal=67% |
| 27 | MITOCHONDRION\_ORGANIZATION\_AND\_BIOGENESIS |  | 42 | 0.47 | 2.00 | 0.000 | 0.004 | 0.130 | 3212 | tags=50%, list=25%, signal=66% |
| 28 | DOUBLE\_STRAND\_BREAK\_REPAIR |  | 21 | 0.57 | 2.00 | 0.004 | 0.004 | 0.138 | 1901 | tags=52%, list=15%, signal=61% |
| 29 | REGULATION\_OF\_MITOTIC\_CELL\_CYCLE |  | 19 | 0.57 | 1.99 | 0.002 | 0.004 | 0.148 | 1446 | tags=47%, list=11%, signal=53% |
| 30 | MRNA\_METABOLIC\_PROCESS |  | 72 | 0.42 | 1.98 | 0.000 | 0.004 | 0.152 | 3121 | tags=49%, list=24%, signal=63% |
| 31 | REGULATION\_OF\_CELL\_CYCLE |  | 161 | 0.36 | 1.98 | 0.000 | 0.004 | 0.154 | 1965 | tags=35%, list=15%, signal=40% |
| 32 | COENZYME\_METABOLIC\_PROCESS |  | 35 | 0.48 | 1.94 | 0.002 | 0.006 | 0.225 | 3366 | tags=49%, list=26%, signal=65% |
| 33 | TRNA\_METABOLIC\_PROCESS |  | 15 | 0.61 | 1.94 | 0.004 | 0.006 | 0.226 | 2613 | tags=67%, list=20%, signal=83% |
| 34 | INTERPHASE\_OF\_MITOTIC\_CELL\_CYCLE |  | 57 | 0.42 | 1.93 | 0.000 | 0.007 | 0.249 | 3342 | tags=51%, list=26%, signal=68% |
| 35 | NUCLEOBASENUCLEOSIDENUCLEOTIDE\_AND\_NUCLEIC\_ACID\_TRANSPORT |  | 26 | 0.50 | 1.93 | 0.002 | 0.007 | 0.250 | 2195 | tags=46%, list=17%, signal=55% |
| 36 | INTERPHASE |  | 63 | 0.41 | 1.91 | 0.000 | 0.008 | 0.288 | 3342 | tags=49%, list=26%, signal=66% |
| 37 | COFACTOR\_BIOSYNTHETIC\_PROCESS |  | 21 | 0.53 | 1.88 | 0.002 | 0.010 | 0.386 | 1461 | tags=38%, list=11%, signal=43% |
| 38 | REGULATION\_OF\_DNA\_REPLICATION |  | 18 | 0.55 | 1.87 | 0.009 | 0.012 | 0.438 | 1965 | tags=50%, list=15%, signal=59% |
| 39 | MRNA\_PROCESSING\_GO\_0006397 |  | 61 | 0.40 | 1.86 | 0.002 | 0.012 | 0.445 | 3121 | tags=46%, list=24%, signal=60% |
| 40 | REGULATION\_OF\_CYCLIN\_DEPENDENT\_PROTEIN\_KINASE\_ACTIVITY |  | 40 | 0.44 | 1.86 | 0.005 | 0.011 | 0.447 | 2414 | tags=48%, list=18%, signal=58% |
| 41 | CHROMOSOME\_ORGANIZATION\_AND\_BIOGENESIS |  | 107 | 0.36 | 1.85 | 0.000 | 0.012 | 0.477 | 2845 | tags=39%, list=22%, signal=50% |
| 42 | REGULATION\_OF\_DNA\_METABOLIC\_PROCESS |  | 40 | 0.44 | 1.84 | 0.000 | 0.013 | 0.500 | 2105 | tags=45%, list=16%, signal=53% |
| 43 | MITOCHONDRIAL\_TRANSPORT |  | 18 | 0.54 | 1.82 | 0.004 | 0.015 | 0.574 | 1461 | tags=44%, list=11%, signal=50% |
| 44 | DNA\_DAMAGE\_CHECKPOINT |  | 19 | 0.53 | 1.80 | 0.004 | 0.017 | 0.624 | 1965 | tags=53%, list=15%, signal=62% |
| 45 | TRANSCRIPTION\_INITIATION\_FROM\_RNA\_POLYMERASE\_II\_PROMOTER |  | 27 | 0.48 | 1.80 | 0.006 | 0.017 | 0.630 | 2414 | tags=44%, list=18%, signal=54% |
| 46 | COFACTOR\_METABOLIC\_PROCESS |  | 51 | 0.40 | 1.77 | 0.002 | 0.020 | 0.697 | 3423 | tags=43%, list=26%, signal=58% |
| 47 | G1\_S\_TRANSITION\_OF\_MITOTIC\_CELL\_CYCLE |  | 23 | 0.48 | 1.77 | 0.000 | 0.021 | 0.713 | 3299 | tags=52%, list=25%, signal=70% |
| 48 | PROTEIN\_MODIFICATION\_BY\_SMALL\_PROTEIN\_CONJUGATION |  | 35 | 0.43 | 1.75 | 0.004 | 0.024 | 0.768 | 2418 | tags=40%, list=18%, signal=49% |
| 49 | DNA\_DAMAGE\_RESPONSESIGNAL\_TRANSDUCTION |  | 34 | 0.44 | 1.73 | 0.002 | 0.027 | 0.809 | 1965 | tags=44%, list=15%, signal=52% |
| 50 | NUCLEAR\_EXPORT |  | 26 | 0.47 | 1.72 | 0.007 | 0.028 | 0.833 | 4233 | tags=58%, list=32%, signal=85% |
| 51 | UBIQUITIN\_CYCLE |  | 40 | 0.41 | 1.72 | 0.009 | 0.029 | 0.852 | 2418 | tags=38%, list=18%, signal=46% |
| 52 | BIOPOLYMER\_CATABOLIC\_PROCESS |  | 103 | 0.34 | 1.71 | 0.000 | 0.031 | 0.876 | 3560 | tags=43%, list=27%, signal=58% |
| 53 | RNA\_EXPORT\_FROM\_NUCLEUS |  | 17 | 0.51 | 1.69 | 0.020 | 0.033 | 0.904 | 4588 | tags=71%, list=35%, signal=109% |
| 54 | PROTEIN\_UBIQUITINATION |  | 32 | 0.42 | 1.67 | 0.012 | 0.038 | 0.934 | 2418 | tags=38%, list=18%, signal=46% |
| 55 | PROTEIN\_CATABOLIC\_PROCESS |  | 60 | 0.37 | 1.67 | 0.009 | 0.038 | 0.935 | 2418 | tags=33%, list=18%, signal=41% |
| 56 | CELLULAR\_PROTEIN\_CATABOLIC\_PROCESS |  | 50 | 0.38 | 1.65 | 0.015 | 0.044 | 0.962 | 2418 | tags=34%, list=18%, signal=42% |
| 57 | PROTEIN\_DNA\_COMPLEX\_ASSEMBLY |  | 45 | 0.38 | 1.65 | 0.010 | 0.044 | 0.962 | 2604 | tags=40%, list=20%, signal=50% |
| 58 | CYTOKINESIS |  | 17 | 0.50 | 1.65 | 0.025 | 0.044 | 0.964 | 1003 | tags=35%, list=8%, signal=38% |
| 59 | MACROMOLECULE\_CATABOLIC\_PROCESS |  | 120 | 0.31 | 1.63 | 0.003 | 0.048 | 0.975 | 3560 | tags=39%, list=27%, signal=53% |
| 60 | BASE\_EXCISION\_REPAIR |  | 16 | 0.49 | 1.63 | 0.017 | 0.050 | 0.982 | 2801 | tags=44%, list=21%, signal=56% |
| 61 | DNA\_RECOMBINATION |  | 45 | 0.37 | 1.60 | 0.015 | 0.058 | 0.990 | 1655 | tags=33%, list=13%, signal=38% |
| 62 | DNA\_PACKAGING |  | 29 | 0.41 | 1.58 | 0.028 | 0.065 | 0.997 | 2845 | tags=45%, list=22%, signal=57% |
| 63 | NUCLEAR\_TRANSPORT |  | 77 | 0.32 | 1.58 | 0.007 | 0.068 | 0.998 | 3427 | tags=39%, list=26%, signal=52% |
| 64 | TRANSCRIPTION\_INITIATION |  | 33 | 0.40 | 1.56 | 0.032 | 0.074 | 0.998 | 2414 | tags=39%, list=18%, signal=48% |
| 65 | NUCLEOCYTOPLASMIC\_TRANSPORT |  | 77 | 0.32 | 1.56 | 0.014 | 0.077 | 0.999 | 3427 | tags=39%, list=26%, signal=52% |
| 66 | MEIOTIC\_CELL\_CYCLE |  | 31 | 0.40 | 1.55 | 0.024 | 0.079 | 0.999 | 2712 | tags=42%, list=21%, signal=53% |
| 67 | NUCLEOTIDE\_METABOLIC\_PROCESS |  | 36 | 0.38 | 1.54 | 0.036 | 0.082 | 0.999 | 1272 | tags=31%, list=10%, signal=34% |
| 68 | MICROTUBULE\_BASED\_PROCESS |  | 75 | 0.33 | 1.53 | 0.005 | 0.085 | 0.999 | 2783 | tags=36%, list=21%, signal=45% |
| 69 | MEIOSIS\_I |  | 19 | 0.45 | 1.53 | 0.035 | 0.085 | 1.000 | 1655 | tags=37%, list=13%, signal=42% |
| 70 | CELLULAR\_MACROMOLECULE\_CATABOLIC\_PROCESS |  | 90 | 0.31 | 1.53 | 0.015 | 0.085 | 1.000 | 2418 | tags=30%, list=18%, signal=37% |
| 71 | NUCLEOBASENUCLEOSIDE\_AND\_NUCLEOTIDE\_METABOLIC\_PROCESS |  | 46 | 0.36 | 1.53 | 0.008 | 0.084 | 1.000 | 1272 | tags=28%, list=10%, signal=31% |
| 72 | CELL\_DIVISION |  | 19 | 0.45 | 1.53 | 0.046 | 0.085 | 1.000 | 1003 | tags=32%, list=8%, signal=34% |
| 73 | ORGANELLE\_ORGANIZATION\_AND\_BIOGENESIS |  | 407 | 0.24 | 1.52 | 0.000 | 0.089 | 1.000 | 2809 | tags=30%, list=21%, signal=37% |
| 74 | ONE\_CARBON\_COMPOUND\_METABOLIC\_PROCESS |  | 24 | 0.42 | 1.52 | 0.035 | 0.088 | 1.000 | 3123 | tags=50%, list=24%, signal=66% |
| 75 | CELLULAR\_COMPONENT\_DISASSEMBLY |  | 31 | 0.38 | 1.47 | 0.046 | 0.114 | 1.000 | 3038 | tags=42%, list=23%, signal=54% |
| 76 | ESTABLISHMENT\_OF\_ORGANELLE\_LOCALIZATION |  | 16 | 0.46 | 1.46 | 0.064 | 0.127 | 1.000 | 907 | tags=38%, list=7%, signal=40% |
| 77 | APOPTOTIC\_NUCLEAR\_CHANGES |  | 17 | 0.44 | 1.45 | 0.072 | 0.127 | 1.000 | 2910 | tags=47%, list=22%, signal=60% |
| 78 | CHROMATIN\_ASSEMBLY\_OR\_DISASSEMBLY |  | 25 | 0.39 | 1.44 | 0.069 | 0.139 | 1.000 | 2845 | tags=48%, list=22%, signal=61% |
| 79 | ORGANELLE\_LOCALIZATION |  | 21 | 0.40 | 1.42 | 0.084 | 0.150 | 1.000 | 907 | tags=29%, list=7%, signal=31% |
| 80 | VIRAL\_INFECTIOUS\_CYCLE |  | 29 | 0.37 | 1.42 | 0.065 | 0.152 | 1.000 | 1661 | tags=34%, list=13%, signal=39% |
| 81 | NEGATIVE\_REGULATION\_OF\_DNA\_METABOLIC\_PROCESS |  | 16 | 0.43 | 1.40 | 0.096 | 0.174 | 1.000 | 2577 | tags=50%, list=20%, signal=62% |
| 82 | VIRAL\_REPRODUCTIVE\_PROCESS |  | 33 | 0.35 | 1.37 | 0.097 | 0.200 | 1.000 | 1661 | tags=33%, list=13%, signal=38% |
| 83 | ALCOHOL\_METABOLIC\_PROCESS |  | 82 | 0.28 | 1.37 | 0.062 | 0.204 | 1.000 | 4336 | tags=44%, list=33%, signal=65% |
| 84 | ESTABLISHMENT\_AND\_OR\_MAINTENANCE\_OF\_CHROMATIN\_ARCHITECTURE |  | 65 | 0.29 | 1.34 | 0.083 | 0.241 | 1.000 | 2845 | tags=35%, list=22%, signal=45% |
| 85 | RESPONSE\_TO\_HYPOXIA |  | 27 | 0.35 | 1.32 | 0.131 | 0.259 | 1.000 | 2417 | tags=33%, list=18%, signal=41% |
| 86 | VIRAL\_REPRODUCTION |  | 38 | 0.31 | 1.31 | 0.121 | 0.272 | 1.000 | 1922 | tags=32%, list=15%, signal=37% |
| 87 | VIRAL\_GENOME\_REPLICATION |  | 20 | 0.38 | 1.31 | 0.109 | 0.274 | 1.000 | 1661 | tags=35%, list=13%, signal=40% |
| 88 | CHROMATIN\_REMODELING |  | 21 | 0.37 | 1.30 | 0.150 | 0.284 | 1.000 | 2845 | tags=43%, list=22%, signal=55% |
| 89 | NEGATIVE\_REGULATION\_OF\_BINDING |  | 16 | 0.40 | 1.29 | 0.159 | 0.288 | 1.000 | 2910 | tags=50%, list=22%, signal=64% |
| 90 | RESPONSE\_TO\_ORGANIC\_SUBSTANCE |  | 27 | 0.34 | 1.29 | 0.141 | 0.290 | 1.000 | 2898 | tags=37%, list=22%, signal=47% |
| 91 | RNA\_CATABOLIC\_PROCESS |  | 20 | 0.37 | 1.29 | 0.152 | 0.289 | 1.000 | 3053 | tags=50%, list=23%, signal=65% |
| 92 | INTRACELLULAR\_TRANSPORT |  | 248 | 0.22 | 1.29 | 0.037 | 0.286 | 1.000 | 3675 | tags=35%, list=28%, signal=47% |
| 93 | NEGATIVE\_REGULATION\_OF\_CATALYTIC\_ACTIVITY |  | 61 | 0.28 | 1.28 | 0.110 | 0.292 | 1.000 | 3222 | tags=39%, list=25%, signal=52% |
| 94 | APOPTOTIC\_PROGRAM |  | 56 | 0.28 | 1.27 | 0.123 | 0.313 | 1.000 | 3135 | tags=38%, list=24%, signal=49% |
| 95 | HETEROCYCLE\_METABOLIC\_PROCESS |  | 26 | 0.34 | 1.26 | 0.157 | 0.321 | 1.000 | 1461 | tags=23%, list=11%, signal=26% |
| 96 | G1\_PHASE |  | 15 | 0.39 | 1.26 | 0.183 | 0.318 | 1.000 | 3150 | tags=47%, list=24%, signal=61% |
| 97 | RESPONSE\_TO\_ABIOTIC\_STIMULUS |  | 79 | 0.26 | 1.26 | 0.107 | 0.316 | 1.000 | 3366 | tags=37%, list=26%, signal=49% |
| 98 | CYTOSKELETON\_DEPENDENT\_INTRACELLULAR\_TRANSPORT |  | 25 | 0.34 | 1.26 | 0.163 | 0.319 | 1.000 | 4113 | tags=52%, list=31%, signal=76% |
| 99 | ESTABLISHMENT\_OF\_CELLULAR\_LOCALIZATION |  | 311 | 0.21 | 1.26 | 0.057 | 0.317 | 1.000 | 3734 | tags=34%, list=29%, signal=46% |
| 100 | RESPONSE\_TO\_STRESS |  | 467 | 0.20 | 1.23 | 0.040 | 0.357 | 1.000 | 2987 | tags=28%, list=23%, signal=36% |
| 101 | CELLULAR\_LOCALIZATION |  | 323 | 0.20 | 1.22 | 0.064 | 0.372 | 1.000 | 3734 | tags=33%, list=29%, signal=45% |
| 102 | RIBONUCLEOPROTEIN\_COMPLEX\_BIOGENESIS\_AND\_ASSEMBLY |  | 68 | 0.26 | 1.22 | 0.155 | 0.382 | 1.000 | 3667 | tags=40%, list=28%, signal=55% |
| 103 | DNA\_CATABOLIC\_PROCESS |  | 21 | 0.35 | 1.21 | 0.207 | 0.402 | 1.000 | 3404 | tags=43%, list=26%, signal=58% |
| 104 | MEIOTIC\_RECOMBINATION |  | 16 | 0.37 | 1.20 | 0.226 | 0.419 | 1.000 | 1655 | tags=31%, list=13%, signal=36% |
| 105 | REGULATION\_OF\_GENE\_EXPRESSION\_EPIGENETIC |  | 27 | 0.31 | 1.19 | 0.222 | 0.419 | 1.000 | 3123 | tags=41%, list=24%, signal=53% |
| 106 | REGULATION\_OF\_HYDROLASE\_ACTIVITY |  | 65 | 0.25 | 1.18 | 0.200 | 0.438 | 1.000 | 3135 | tags=34%, list=24%, signal=44% |
| 107 | RESPONSE\_TO\_HORMONE\_STIMULUS |  | 26 | 0.31 | 1.17 | 0.248 | 0.451 | 1.000 | 4947 | tags=58%, list=38%, signal=93% |
| 108 | TRANSCRIPTION\_FROM\_RNA\_POLYMERASE\_III\_PROMOTER |  | 18 | 0.35 | 1.17 | 0.266 | 0.452 | 1.000 | 3689 | tags=56%, list=28%, signal=77% |
| 109 | CELLULAR\_RESPIRATION |  | 19 | 0.34 | 1.17 | 0.244 | 0.450 | 1.000 | 2671 | tags=37%, list=20%, signal=46% |
| 110 | INDUCTION\_OF\_APOPTOSIS\_BY\_EXTRACELLULAR\_SIGNALS |  | 25 | 0.32 | 1.17 | 0.267 | 0.451 | 1.000 | 2596 | tags=36%, list=20%, signal=45% |
| 111 | CHROMATIN\_MODIFICATION |  | 46 | 0.26 | 1.15 | 0.241 | 0.497 | 1.000 | 2845 | tags=33%, list=22%, signal=42% |
| 112 | NITROGEN\_COMPOUND\_BIOSYNTHETIC\_PROCESS |  | 25 | 0.30 | 1.15 | 0.273 | 0.497 | 1.000 | 1554 | tags=24%, list=12%, signal=27% |
| 113 | TRANSCRIPTION\_FROM\_RNA\_POLYMERASE\_II\_PROMOTER |  | 428 | 0.18 | 1.14 | 0.129 | 0.507 | 1.000 | 2644 | tags=24%, list=20%, signal=29% |
| 114 | CATABOLIC\_PROCESS |  | 201 | 0.20 | 1.14 | 0.181 | 0.503 | 1.000 | 3619 | tags=32%, list=28%, signal=44% |
| 115 | NEGATIVE\_REGULATION\_OF\_DNA\_BINDING |  | 15 | 0.36 | 1.14 | 0.304 | 0.506 | 1.000 | 2910 | tags=47%, list=22%, signal=60% |
| 116 | NUCLEAR\_ORGANIZATION\_AND\_BIOGENESIS |  | 23 | 0.32 | 1.13 | 0.283 | 0.508 | 1.000 | 2910 | tags=39%, list=22%, signal=50% |
| 117 | LIPID\_BIOSYNTHETIC\_PROCESS |  | 84 | 0.23 | 1.13 | 0.265 | 0.517 | 1.000 | 1679 | tags=21%, list=13%, signal=24% |
| 118 | CHROMATIN\_ASSEMBLY |  | 16 | 0.35 | 1.13 | 0.306 | 0.516 | 1.000 | 2845 | tags=44%, list=22%, signal=56% |
| 119 | CELL\_STRUCTURE\_DISASSEMBLY\_DURING\_APOPTOSIS |  | 17 | 0.34 | 1.13 | 0.316 | 0.516 | 1.000 | 2910 | tags=35%, list=22%, signal=45% |
| 120 | REGULATION\_OF\_KINASE\_ACTIVITY |  | 135 | 0.21 | 1.12 | 0.242 | 0.518 | 1.000 | 1655 | tags=20%, list=13%, signal=23% |
| 121 | GLUTAMATE\_SIGNALING\_PATHWAY |  | 17 | 0.33 | 1.12 | 0.299 | 0.530 | 1.000 | 2902 | tags=29%, list=22%, signal=38% |
| 122 | REGULATION\_OF\_TRANSFERASE\_ACTIVITY |  | 137 | 0.20 | 1.11 | 0.259 | 0.540 | 1.000 | 1655 | tags=20%, list=13%, signal=22% |
| 123 | CELLULAR\_CATABOLIC\_PROCESS |  | 189 | 0.20 | 1.11 | 0.230 | 0.536 | 1.000 | 3619 | tags=32%, list=28%, signal=43% |
| 124 | NEURON\_APOPTOSIS |  | 15 | 0.34 | 1.11 | 0.308 | 0.536 | 1.000 | 1332 | tags=27%, list=10%, signal=30% |
| 125 | REGULATION\_OF\_CATALYTIC\_ACTIVITY |  | 238 | 0.19 | 1.10 | 0.220 | 0.561 | 1.000 | 3222 | tags=29%, list=25%, signal=37% |
| 126 | CELLULAR\_RESPONSE\_TO\_STIMULUS |  | 17 | 0.33 | 1.10 | 0.331 | 0.560 | 1.000 | 2898 | tags=35%, list=22%, signal=45% |
| 127 | CYTOSKELETON\_ORGANIZATION\_AND\_BIOGENESIS |  | 182 | 0.19 | 1.09 | 0.265 | 0.556 | 1.000 | 2783 | tags=27%, list=21%, signal=34% |
| 128 | NEGATIVE\_REGULATION\_OF\_TRANSPORT |  | 18 | 0.33 | 1.09 | 0.331 | 0.553 | 1.000 | 3551 | tags=44%, list=27%, signal=61% |
| 129 | REGULATION\_OF\_PROTEIN\_KINASE\_ACTIVITY |  | 133 | 0.20 | 1.09 | 0.289 | 0.562 | 1.000 | 4411 | tags=43%, list=34%, signal=64% |
| 130 | NUCLEAR\_IMPORT |  | 47 | 0.25 | 1.08 | 0.297 | 0.579 | 1.000 | 3397 | tags=34%, list=26%, signal=46% |
| 131 | CELLULAR\_BIOSYNTHETIC\_PROCESS |  | 273 | 0.18 | 1.07 | 0.299 | 0.618 | 1.000 | 1589 | tags=18%, list=12%, signal=20% |
| 132 | NEGATIVE\_REGULATION\_OF\_APOPTOSIS |  | 136 | 0.20 | 1.05 | 0.346 | 0.640 | 1.000 | 1510 | tags=19%, list=12%, signal=21% |
| 133 | GAMETE\_GENERATION |  | 92 | 0.21 | 1.05 | 0.338 | 0.639 | 1.000 | 3830 | tags=35%, list=29%, signal=49% |
| 134 | OXYGEN\_AND\_REACTIVE\_OXYGEN\_SPECIES\_METABOLIC\_PROCESS |  | 18 | 0.31 | 1.05 | 0.390 | 0.643 | 1.000 | 2883 | tags=39%, list=22%, signal=50% |
| 135 | REGULATION\_OF\_PROTEIN\_STABILITY |  | 17 | 0.32 | 1.05 | 0.389 | 0.645 | 1.000 | 4071 | tags=41%, list=31%, signal=60% |
| 136 | INTERACTION\_WITH\_HOST |  | 15 | 0.33 | 1.05 | 0.401 | 0.646 | 1.000 | 1394 | tags=27%, list=11%, signal=30% |
| 137 | DNA\_DAMAGE\_RESPONSESIGNAL\_TRANSDUCTION\_RESULTING\_IN\_INDUCTION\_OF\_APOPTOSIS |  | 15 | 0.33 | 1.04 | 0.387 | 0.646 | 1.000 | 1104 | tags=27%, list=8%, signal=29% |
| 138 | PIGMENT\_BIOSYNTHETIC\_PROCESS |  | 17 | 0.31 | 1.04 | 0.406 | 0.651 | 1.000 | 1461 | tags=24%, list=11%, signal=26% |
| 139 | RNA\_SPLICINGVIA\_TRANSESTERIFICATION\_REACTIONS |  | 27 | 0.28 | 1.04 | 0.388 | 0.651 | 1.000 | 2664 | tags=30%, list=20%, signal=37% |
| 140 | NEGATIVE\_REGULATION\_OF\_PROGRAMMED\_CELL\_DEATH |  | 137 | 0.19 | 1.04 | 0.402 | 0.652 | 1.000 | 1510 | tags=19%, list=12%, signal=21% |
| 141 | MICROTUBULE\_BASED\_MOVEMENT |  | 16 | 0.32 | 1.04 | 0.417 | 0.652 | 1.000 | 2677 | tags=38%, list=20%, signal=47% |
| 142 | REGULATION\_OF\_MOLECULAR\_FUNCTION |  | 275 | 0.17 | 1.03 | 0.354 | 0.648 | 1.000 | 2414 | tags=22%, list=18%, signal=27% |
| 143 | CARBOHYDRATE\_TRANSPORT |  | 17 | 0.30 | 1.01 | 0.447 | 0.723 | 1.000 | 2400 | tags=29%, list=18%, signal=36% |
| 144 | MORPHOGENESIS\_OF\_AN\_EPITHELIUM |  | 15 | 0.31 | 1.01 | 0.446 | 0.719 | 1.000 | 3718 | tags=47%, list=28%, signal=65% |
| 145 | SECONDARY\_METABOLIC\_PROCESS |  | 23 | 0.27 | 1.00 | 0.464 | 0.728 | 1.000 | 1461 | tags=22%, list=11%, signal=24% |
| 146 | RESPONSE\_TO\_TEMPERATURE\_STIMULUS |  | 16 | 0.31 | 1.00 | 0.475 | 0.738 | 1.000 | 3366 | tags=44%, list=26%, signal=59% |
| 147 | PROTEIN\_IMPORT |  | 58 | 0.22 | 0.99 | 0.459 | 0.736 | 1.000 | 4148 | tags=36%, list=32%, signal=53% |
| 148 | MACROMOLECULE\_LOCALIZATION |  | 202 | 0.17 | 0.99 | 0.479 | 0.731 | 1.000 | 3442 | tags=29%, list=26%, signal=38% |
| 149 | NEGATIVE\_REGULATION\_OF\_TRANSFERASE\_ACTIVITY |  | 27 | 0.26 | 0.99 | 0.486 | 0.734 | 1.000 | 3210 | tags=37%, list=25%, signal=49% |
| 150 | STEROID\_HORMONE\_RECEPTOR\_SIGNALING\_PATHWAY |  | 18 | 0.29 | 0.99 | 0.471 | 0.737 | 1.000 | 1213 | tags=22%, list=9%, signal=24% |
| 151 | REGULATION\_OF\_TRANSCRIPTION\_FROM\_RNA\_POLYMERASE\_II\_PROMOTER |  | 267 | 0.17 | 0.99 | 0.480 | 0.735 | 1.000 | 2467 | tags=22%, list=19%, signal=27% |
| 152 | STEROID\_METABOLIC\_PROCESS |  | 66 | 0.21 | 0.99 | 0.501 | 0.730 | 1.000 | 4324 | tags=42%, list=33%, signal=63% |
| 153 | CELL\_PROJECTION\_BIOGENESIS |  | 20 | 0.29 | 0.98 | 0.456 | 0.733 | 1.000 | 4157 | tags=45%, list=32%, signal=66% |
| 154 | INTRACELLULAR\_PROTEIN\_TRANSPORT |  | 127 | 0.18 | 0.98 | 0.519 | 0.732 | 1.000 | 3442 | tags=29%, list=26%, signal=39% |
| 155 | PIGMENT\_METABOLIC\_PROCESS |  | 18 | 0.29 | 0.98 | 0.481 | 0.731 | 1.000 | 1461 | tags=22%, list=11%, signal=25% |
| 156 | INTRACELLULAR\_RECEPTOR\_MEDIATED\_SIGNALING\_PATHWAY |  | 18 | 0.29 | 0.98 | 0.490 | 0.735 | 1.000 | 1213 | tags=22%, list=9%, signal=24% |
| 157 | PROTEIN\_AMINO\_ACID\_O\_LINKED\_GLYCOSYLATION |  | 18 | 0.28 | 0.98 | 0.488 | 0.732 | 1.000 | 2802 | tags=39%, list=21%, signal=49% |
| 158 | STEROID\_BIOSYNTHETIC\_PROCESS |  | 22 | 0.28 | 0.98 | 0.497 | 0.732 | 1.000 | 4158 | tags=55%, list=32%, signal=80% |
| 159 | GENERATION\_OF\_A\_SIGNAL\_INVOLVED\_IN\_CELL\_CELL\_SIGNALING |  | 25 | 0.27 | 0.97 | 0.515 | 0.751 | 1.000 | 2341 | tags=28%, list=18%, signal=34% |
| 160 | ENERGY\_DERIVATION\_BY\_OXIDATION\_OF\_ORGANIC\_COMPOUNDS |  | 37 | 0.24 | 0.97 | 0.495 | 0.749 | 1.000 | 2217 | tags=24%, list=17%, signal=29% |
| 161 | DIGESTION |  | 42 | 0.23 | 0.97 | 0.480 | 0.746 | 1.000 | 2965 | tags=24%, list=23%, signal=31% |
| 162 | PROGRAMMED\_CELL\_DEATH |  | 393 | 0.15 | 0.96 | 0.578 | 0.760 | 1.000 | 1911 | tags=18%, list=15%, signal=20% |
| 163 | REGULATION\_OF\_PROGRAMMED\_CELL\_DEATH |  | 313 | 0.16 | 0.96 | 0.590 | 0.760 | 1.000 | 1890 | tags=18%, list=14%, signal=21% |
| 164 | PROTEIN\_TRANSPORT |  | 139 | 0.18 | 0.96 | 0.569 | 0.760 | 1.000 | 3774 | tags=32%, list=29%, signal=44% |
| 165 | APOPTOSIS\_GO |  | 392 | 0.15 | 0.96 | 0.587 | 0.755 | 1.000 | 1911 | tags=18%, list=15%, signal=20% |
| 166 | PROTEIN\_TARGETING |  | 94 | 0.19 | 0.95 | 0.548 | 0.766 | 1.000 | 3442 | tags=29%, list=26%, signal=39% |
| 167 | COVALENT\_CHROMATIN\_MODIFICATION |  | 22 | 0.27 | 0.94 | 0.532 | 0.778 | 1.000 | 3656 | tags=41%, list=28%, signal=57% |
| 168 | REGULATION\_OF\_APOPTOSIS |  | 312 | 0.16 | 0.94 | 0.624 | 0.776 | 1.000 | 1890 | tags=18%, list=14%, signal=21% |
| 169 | REGULATION\_OF\_NEUROTRANSMITTER\_LEVELS |  | 23 | 0.26 | 0.94 | 0.533 | 0.790 | 1.000 | 1096 | tags=17%, list=8%, signal=19% |
| 170 | PROTEIN\_RNA\_COMPLEX\_ASSEMBLY |  | 55 | 0.21 | 0.93 | 0.566 | 0.795 | 1.000 | 3667 | tags=36%, list=28%, signal=50% |
| 171 | BIOSYNTHETIC\_PROCESS |  | 402 | 0.15 | 0.92 | 0.719 | 0.811 | 1.000 | 1734 | tags=16%, list=13%, signal=18% |
| 172 | REGULATION\_OF\_TRANSPORT |  | 57 | 0.20 | 0.92 | 0.571 | 0.823 | 1.000 | 4233 | tags=42%, list=32%, signal=62% |
| 173 | INDUCTION\_OF\_APOPTOSIS\_BY\_INTRACELLULAR\_SIGNALS |  | 23 | 0.25 | 0.91 | 0.593 | 0.825 | 1.000 | 3299 | tags=35%, list=25%, signal=46% |
| 174 | NEGATIVE\_REGULATION\_OF\_CELL\_ADHESION |  | 16 | 0.28 | 0.91 | 0.583 | 0.840 | 1.000 | 3383 | tags=44%, list=26%, signal=59% |
| 175 | AROMATIC\_COMPOUND\_METABOLIC\_PROCESS |  | 26 | 0.24 | 0.91 | 0.601 | 0.836 | 1.000 | 379 | tags=15%, list=3%, signal=16% |
| 176 | PROTEIN\_IMPORT\_INTO\_NUCLEUS |  | 45 | 0.21 | 0.90 | 0.629 | 0.851 | 1.000 | 4148 | tags=38%, list=32%, signal=55% |
| 177 | SEXUAL\_REPRODUCTION |  | 109 | 0.17 | 0.88 | 0.680 | 0.882 | 1.000 | 3830 | tags=31%, list=29%, signal=44% |
| 178 | ENERGY\_RESERVE\_METABOLIC\_PROCESS |  | 15 | 0.28 | 0.88 | 0.624 | 0.900 | 1.000 | 1248 | tags=20%, list=10%, signal=22% |
| 179 | POSITIVE\_REGULATION\_OF\_CELL\_CYCLE |  | 15 | 0.28 | 0.87 | 0.646 | 0.902 | 1.000 | 1575 | tags=27%, list=12%, signal=30% |
| 180 | REGULATION\_OF\_RNA\_METABOLIC\_PROCESS |  | 417 | 0.14 | 0.87 | 0.855 | 0.897 | 1.000 | 2421 | tags=20%, list=18%, signal=24% |
| 181 | REGULATION\_OF\_NUCLEOCYTOPLASMIC\_TRANSPORT |  | 19 | 0.25 | 0.87 | 0.657 | 0.896 | 1.000 | 1277 | tags=21%, list=10%, signal=23% |
| 182 | TRANSMISSION\_OF\_NERVE\_IMPULSE |  | 167 | 0.16 | 0.86 | 0.763 | 0.915 | 1.000 | 2560 | tags=20%, list=20%, signal=25% |
| 183 | SPLICEOSOME\_ASSEMBLY |  | 17 | 0.26 | 0.86 | 0.644 | 0.910 | 1.000 | 3307 | tags=35%, list=25%, signal=47% |
| 184 | RESPONSE\_TO\_RADIATION |  | 52 | 0.19 | 0.86 | 0.705 | 0.914 | 1.000 | 3227 | tags=31%, list=25%, signal=41% |
| 185 | REGULATION\_OF\_INTRACELLULAR\_TRANSPORT |  | 22 | 0.24 | 0.85 | 0.665 | 0.916 | 1.000 | 4233 | tags=45%, list=32%, signal=67% |
| 186 | AEROBIC\_RESPIRATION |  | 15 | 0.27 | 0.85 | 0.660 | 0.913 | 1.000 | 2671 | tags=33%, list=20%, signal=42% |
| 187 | PROTEOLYSIS |  | 170 | 0.15 | 0.85 | 0.800 | 0.913 | 1.000 | 3846 | tags=32%, list=29%, signal=45% |
| 188 | EMBRYONIC\_DEVELOPMENT |  | 46 | 0.19 | 0.85 | 0.708 | 0.911 | 1.000 | 3011 | tags=26%, list=23%, signal=34% |
| 189 | PHOSPHOINOSITIDE\_BIOSYNTHETIC\_PROCESS |  | 21 | 0.24 | 0.85 | 0.677 | 0.910 | 1.000 | 1022 | tags=19%, list=8%, signal=21% |
| 190 | SYNAPTIC\_TRANSMISSION |  | 154 | 0.15 | 0.84 | 0.809 | 0.918 | 1.000 | 2520 | tags=19%, list=19%, signal=24% |
| 191 | CALCIUM\_INDEPENDENT\_CELL\_CELL\_ADHESION |  | 16 | 0.25 | 0.83 | 0.685 | 0.945 | 1.000 | 3617 | tags=38%, list=28%, signal=52% |
| 192 | MEMBRANE\_LIPID\_BIOSYNTHETIC\_PROCESS |  | 41 | 0.19 | 0.82 | 0.768 | 0.947 | 1.000 | 1554 | tags=17%, list=12%, signal=19% |
| 193 | DEVELOPMENT\_OF\_PRIMARY\_SEXUAL\_CHARACTERISTICS |  | 25 | 0.22 | 0.82 | 0.741 | 0.944 | 1.000 | 3011 | tags=28%, list=23%, signal=36% |
| 194 | MEMBRANE\_FUSION |  | 27 | 0.22 | 0.82 | 0.733 | 0.944 | 1.000 | 3560 | tags=37%, list=27%, signal=51% |
| 195 | PROTEIN\_HOMOOLIGOMERIZATION |  | 19 | 0.23 | 0.81 | 0.748 | 0.955 | 1.000 | 844 | tags=16%, list=6%, signal=17% |
| 196 | NITROGEN\_COMPOUND\_METABOLIC\_PROCESS |  | 141 | 0.15 | 0.81 | 0.875 | 0.957 | 1.000 | 1800 | tags=16%, list=14%, signal=19% |
| 197 | EXOCYTOSIS |  | 22 | 0.22 | 0.80 | 0.743 | 0.958 | 1.000 | 10158 | tags=100%, list=78%, signal=446% |
| 198 | ESTABLISHMENT\_OF\_PROTEIN\_LOCALIZATION |  | 166 | 0.14 | 0.80 | 0.913 | 0.958 | 1.000 | 4190 | tags=33%, list=32%, signal=48% |
| 199 | HISTONE\_MODIFICATION |  | 21 | 0.23 | 0.80 | 0.731 | 0.958 | 1.000 | 3656 | tags=38%, list=28%, signal=53% |
| 200 | ECTODERM\_DEVELOPMENT |  | 75 | 0.17 | 0.79 | 0.857 | 0.959 | 1.000 | 2500 | tags=24%, list=19%, signal=29% |
| 201 | CASPASE\_ACTIVATION |  | 24 | 0.21 | 0.79 | 0.763 | 0.958 | 1.000 | 3074 | tags=33%, list=23%, signal=43% |
| 202 | TRANSLATIONAL\_INITIATION |  | 33 | 0.20 | 0.78 | 0.815 | 0.975 | 1.000 | 3622 | tags=36%, list=28%, signal=50% |
| 203 | LIPID\_TRANSPORT |  | 27 | 0.21 | 0.78 | 0.807 | 0.973 | 1.000 | 1930 | tags=22%, list=15%, signal=26% |
| 204 | PHOSPHOLIPID\_BIOSYNTHETIC\_PROCESS |  | 35 | 0.19 | 0.78 | 0.836 | 0.969 | 1.000 | 1554 | tags=17%, list=12%, signal=19% |
| 205 | RESPONSE\_TO\_UV |  | 22 | 0.22 | 0.77 | 0.780 | 0.966 | 1.000 | 3227 | tags=36%, list=25%, signal=48% |
| 206 | REGULATION\_OF\_SECRETION |  | 35 | 0.19 | 0.77 | 0.834 | 0.974 | 1.000 | 2406 | tags=26%, list=18%, signal=31% |
| 207 | GLYCEROPHOSPHOLIPID\_BIOSYNTHETIC\_PROCESS |  | 27 | 0.20 | 0.76 | 0.805 | 0.976 | 1.000 | 1022 | tags=15%, list=8%, signal=16% |
| 208 | SENSORY\_PERCEPTION |  | 163 | 0.14 | 0.76 | 0.947 | 0.971 | 1.000 | 5183 | tags=44%, list=40%, signal=71% |
| 209 | REPRODUCTION |  | 215 | 0.13 | 0.76 | 0.975 | 0.970 | 1.000 | 3494 | tags=27%, list=27%, signal=36% |
| 210 | NEGATIVE\_REGULATION\_OF\_CELLULAR\_BIOSYNTHETIC\_PROCESS |  | 25 | 0.20 | 0.75 | 0.833 | 0.971 | 1.000 | 3211 | tags=28%, list=25%, signal=37% |
| 211 | NEGATIVE\_REGULATION\_OF\_CELL\_CYCLE |  | 72 | 0.15 | 0.74 | 0.921 | 0.986 | 1.000 | 1901 | tags=18%, list=15%, signal=21% |
| 212 | POSITIVE\_REGULATION\_OF\_HYDROLASE\_ACTIVITY |  | 45 | 0.17 | 0.73 | 0.907 | 0.987 | 1.000 | 3074 | tags=27%, list=23%, signal=35% |
| 213 | REGULATION\_OF\_CELL\_ADHESION |  | 31 | 0.19 | 0.72 | 0.872 | 0.992 | 1.000 | 4167 | tags=42%, list=32%, signal=61% |
| 214 | CELLULAR\_CARBOHYDRATE\_METABOLIC\_PROCESS |  | 106 | 0.14 | 0.72 | 0.956 | 0.991 | 1.000 | 2736 | tags=20%, list=21%, signal=25% |
| 215 | LIPOPROTEIN\_METABOLIC\_PROCESS |  | 30 | 0.19 | 0.72 | 0.879 | 0.987 | 1.000 | 1930 | tags=20%, list=15%, signal=23% |
| 216 | HOMEOSTASIS\_OF\_NUMBER\_OF\_CELLS |  | 20 | 0.21 | 0.72 | 0.849 | 0.983 | 1.000 | 2377 | tags=25%, list=18%, signal=30% |
| 217 | NEGATIVE\_REGULATION\_OF\_BIOSYNTHETIC\_PROCESS |  | 26 | 0.19 | 0.72 | 0.857 | 0.980 | 1.000 | 3211 | tags=27%, list=25%, signal=36% |
| 218 | EPIDERMAL\_GROWTH\_FACTOR\_RECEPTOR\_SIGNALING\_PATHWAY |  | 18 | 0.21 | 0.72 | 0.838 | 0.976 | 1.000 | 4105 | tags=39%, list=31%, signal=57% |
| 219 | JNK\_CASCADE |  | 44 | 0.17 | 0.71 | 0.903 | 0.973 | 1.000 | 4086 | tags=39%, list=31%, signal=56% |
| 220 | STRESS\_ACTIVATED\_PROTEIN\_KINASE\_SIGNALING\_PATHWAY |  | 45 | 0.17 | 0.71 | 0.928 | 0.971 | 1.000 | 4086 | tags=38%, list=31%, signal=55% |
| 221 | REGULATION\_OF\_CATABOLIC\_PROCESS |  | 15 | 0.22 | 0.70 | 0.841 | 0.975 | 1.000 | 4253 | tags=47%, list=32%, signal=69% |
| 222 | NEUROLOGICAL\_SYSTEM\_PROCESS |  | 328 | 0.11 | 0.70 | 1.000 | 0.974 | 1.000 | 4686 | tags=34%, list=36%, signal=52% |
| 223 | GLUCOSE\_METABOLIC\_PROCESS |  | 27 | 0.18 | 0.69 | 0.904 | 0.978 | 1.000 | 4336 | tags=37%, list=33%, signal=55% |
| 224 | ANION\_TRANSPORT |  | 27 | 0.18 | 0.67 | 0.921 | 0.988 | 1.000 | 1461 | tags=15%, list=11%, signal=17% |
| 225 | BIOGENIC\_AMINE\_METABOLIC\_PROCESS |  | 16 | 0.20 | 0.66 | 0.912 | 0.989 | 1.000 | 1554 | tags=19%, list=12%, signal=21% |
| 226 | RHO\_PROTEIN\_SIGNAL\_TRANSDUCTION |  | 30 | 0.17 | 0.66 | 0.915 | 0.986 | 1.000 | 2722 | tags=23%, list=21%, signal=29% |
| 227 | PROTEIN\_AMINO\_ACID\_LIPIDATION |  | 21 | 0.19 | 0.65 | 0.935 | 0.989 | 1.000 | 1022 | tags=14%, list=8%, signal=15% |
| 228 | TUBE\_DEVELOPMENT |  | 15 | 0.19 | 0.62 | 0.943 | 0.996 | 1.000 | 3718 | tags=33%, list=28%, signal=47% |
| 229 | LIPOPROTEIN\_BIOSYNTHETIC\_PROCESS |  | 23 | 0.17 | 0.62 | 0.950 | 0.994 | 1.000 | 3905 | tags=35%, list=30%, signal=49% |
| 230 | ADENYLATE\_CYCLASE\_ACTIVATION |  | 18 | 0.18 | 0.61 | 0.954 | 0.991 | 1.000 | 4548 | tags=39%, list=35%, signal=60% |
| 231 | FEEDING\_BEHAVIOR |  | 20 | 0.18 | 0.61 | 0.956 | 0.988 | 1.000 | 5007 | tags=45%, list=38%, signal=73% |
| 232 | NEGATIVE\_REGULATION\_OF\_TRANSLATION |  | 19 | 0.18 | 0.60 | 0.955 | 0.986 | 1.000 | 3211 | tags=26%, list=25%, signal=35% |
| 233 | REGULATION\_OF\_HEART\_CONTRACTION |  | 24 | 0.15 | 0.53 | 0.990 | 0.998 | 1.000 | 3930 | tags=33%, list=30%, signal=48% |
| 234 | PEROXISOME\_ORGANIZATION\_AND\_BIOGENESIS |  | 15 | 0.14 | 0.44 | 1.000 | 0.999 | 1.000 | 454 | tags=7%, list=3%, signal=7% |
Table: Gene sets enriched in phenotype **na**[plain text format]****

  
